# Multi-omics peripheral and core regions of cancer

**DOI:** 10.1038/s41540-022-00258-1

**Published:** 2022-11-29

**Authors:** Bingbo Wang, Xianan Dong, Jie Hu, Lin Gao

**Affiliations:** School of Computer Science and Technology, Xidian University, Xi’an, 710071 China

**Keywords:** Software, Statistics

## Abstract

Thousands of genes are perturbed by cancer, and these disturbances can be seen in transcriptome, methylation, somatic mutation, and copy number variation omics studies. Understanding their connectivity patterns as an omnigenic neighbourhood in a molecular interaction network (interactome) is a key step towards advancing knowledge of the molecular mechanisms underlying cancers. Here, we introduce a unified connectivity line (*CLine*) to pinpoint omics-specific omnigenic patterns across 15 curated cancers. Taking advantage of the universality of *CLine*, we distinguish the peripheral and core genes for each omics aspect. We propose a network-based framework, multi-omics periphery and core (MOPC), to combine peripheral and core genes from different omics into a button-like structure. On the basis of network proximity, we provide evidence that core genes tend to be specifically perturbed in one omics, but the peripheral genes are diversely perturbed in multiple omics. And the core of one omics is regulated by multiple omics peripheries. Finally, we take the MOPC as an omnigenic neighbourhood, describe its characteristics, and explore its relative contribution to network-based mechanisms of cancer. We were able to present how multi-omics perturbations percolate through the human interactome and contribute to an integrated periphery and core.

## Introduction

Complex diseases result from the interaction of multiple molecular processes^1–6^. Genes rarely work alone. Instead, they are often involved in complex pathways, interacting with other genes and, combined with environmental factors, affecting diseases. To study how disease-related genes interact, studying the relationship between genotype and phenotype in biological networks is necessary. Genes associated with diseases affect each other in *cis*-regulatory or *trans*-regulatory fashions, and their relationships can be modelled as a regulatory network^7^. Genes related to specific diseases tend to cluster in the network neighbourhood, which gives rise to the concept of disease modules^6^, which usually consist of dozens of genes. To accurately identify the network’s disease modules, researchers have developed the connectivity-based DIAMOnD^8^ and C3 algorithms^9^. Both algorithms determine the candidate genes to be imported to connect scattered pathogenic genes and obtain connected disease modules.

Over the past decade, genome-wide association studies (GWAS) have identified pathogenic variants for hundreds of diseases and found that the heritability of most complex diseases is caused by many common variants with small effects, and a small number of rare variants with relatively large effects^10^. For a trait or disease, rare variation only explains a small portion of the heritability, and the heritability explained by the genome variation is much higher than that explained by the rare variation. This phenomenon is called “missing heritability”^11^. Most of the missing heritability results from many small-effect common variations that are not significant under the current sample size^12–16^. All genes active in disease-related tissues affect disease risk, and these genes are widespread across the genome. As the number of genes becomes very large, the contribution of each gene becomes correspondingly smaller, which leads to the limit of Fisher’s famous “infinitesimal model”^12,17^. Therefore, some researchers have proposed a new perspective on understanding complex diseases:^7^ from polygenic to omnigenic.

In the omnigenic model, core genes and peripheral genes play distinct roles in diseases^12^. The number of core genes is small and their variations are critical, which directly affects disease development. In contrast, the number of peripheral genes is large and their variations are moderate, which affects disease risk indirectly through trans-effects on the core genes. There are two key proposals: (1) most genes expressed in disease-related cells may affect the core genes through regulation; and (2) almost all disease heritability is determined by the variation in near peripheral genes^12^. Boyle et al. anticipate that significant phenotypic differences between species are driven by small effects being accumulated, while differences in larger effects may be an exception^7^. This is in line with the thinking of quantitative genetics since Darwin, that evolutionary adaptation mainly comes from many genes with small effects^18–20^. After the concept of the omnigenic model was proposed, understanding of the disease neighbourhood has developed from a mesoscopic partial module to a macroscale omnigenic model.

Some studies have published evidence supporting complex traits with the omnigenic model^21–23^, and others have suggested that this model may underestimate the biological complexity of common diseases^18^. Some key questions still need to be solved, such as: how to define the core genes, what percentage of peripheral genes are accounted for, and whether we can infer the role of peripheral genes from their relationship to core genes. Studies have proposed methods to define and identify core genes from the perspective of genetic and topological characteristics. Ratnakumar et al. proposed a method to identify candidate core genes by combining GWAS hits with the protein–protein interaction (PPI) network^24^. Wang et al. detected the peripheral and core regions of disease based on the significance of the local maxima of connectivity between the differentially expressed genes in the human interactome, and applied it to the comorbidity and drug recommendations for COVID-19^25,26^. The latest development in the genetic architecture of schizophrenia indicates that the omnigenic model may underlie the risk for the disorder^27^, and the association between rare and common variants implicated in psychiatric disease risk constitutes a potentially general phenomenon occurring more widely in complex genetic disorders^28^. O’Connor et al. found that for most complex traits, the genes and loci with the most critical biological effects are often different from those with the strongest common-variant associations^29^. Sinnott-Armstrong et al. found three molecular traits that are highly polygenic, with thousands of variants scattered across the genome, leading to trait variance^30^.

Cancer is caused by the dysfunction of genes and their interactions, rather than the mutation or abnormality of a single gene^1,31,32^. At present, some studies on the polygenic model of cancer are represented by disease modules and driver pathways^33–36^. Cheng et al.^37^ identified the relative network configuration of the drug target module related to the disease module using the network proximity, which helps to detect potentially effective paired drug combinations for cancer. Based on biological pathways and network information, many complex calculation methods have been developed to facilitate detection of cancer-driven variants and pathways^38^. For example, methods exist that use known pathways from public databases^39^, such as HotNet2^40^ based on networks, and Dendrix^41^ based on high exclusivity of variants. Some studies also apply cancer pathways to cancer classification^42,43^. Although cancer pathway analysis has become a powerful tool in cancer genomics, our knowledge about oncogenic pathways or modules remains incomplete. In the past few years, great progress has been made in understanding the molecular changes in cancer development. The latest advances in high-throughput sequencing technologies have provided new ideas for cancer genome-wide research and have greatly enriched The Cancer Genome Atlas database (TCGA)^44^ of cancer multi-omics data, which covers four types of omics technologies: transcriptome differential expression (transcriptome), DNA differential methylation (methylation), somatic mutation, and copy number variation (CNV).

Based on multi-omics data, Ding et al. found (1) somatic driver mutations, germline pathogenic variants, and their interactions in tumours; (2) the tumour genome and epigenome’s influence on the transcriptome and proteome; and (3) the relationship between the tumour and the microenvironment^45^. Using multi-omics technologies, Bhattacharya et al. performed transcriptome-wide association studies^46^ and Duan et al.^47^ analysed cancer subtypes. Shi et al.^48^ have developed a novel algorithm, Iterative Clique Enumeration (ICE), for identifying relatively independent maximal cliques as co-expression modules and a module-based approach to the analysis of gene expression data. Zhang et al.^49^ proposed a method, iMCMC, to identify mutated core cancer modules. Yang et al. revealed common and specific cancer patterns by analysing pan-cancer DNA methylation^50^. Vandin et al. detected significantly mutated pathways in cancer^51^. Sánchez-Vega et al.^52^ analysed the mechanisms and patterns of somatic alterations in ten canonical pathways (containing 246 genes).

At present, research on cancer multi-omics remains in the polygenic graph pattern. The abundant multi-omics cancer data can accelerate the development of cancer biology and related technologies to provide us with opportunities to study cancer’s omnigenic graph pattern. Analysing cancer’s omnigenic pattern is an important way to comprehensively understand cancer’s molecular mechanisms and eliminate the prejudices of single data research. For the research on the multi-omics omnigenic pattern of cancer, some unresolved problems remain: (1) Are the omnigenic patterns omics specific? (2) Does the omics-specific omnigenic pattern have universality across cancers? (3) How do the peripheral and core regions from multi-omics of a specific cancer affect each other, and what is the proximity between them in the network?

In this study, we curated transcriptome, methylation, somatic mutation, and CNV omics datasets from 15 cancers, and depicted the connective properties between the genes perturbed in each omics aspect in the human interactome. We developed a unified network-based *CLine* (Connectivity Line) to pinpoint omics-specific omnigenic patterns across cancers. We observed that the omnigenic patterns present bimodal, fragmented, unimodal, and steepest descent patterns, respectively, in these four omics aspects. Furthermore, these omics-specific patterns have universality in 66.7%, 86.7%, 93.3%, and 93.3% of cancers, respectively. From an omnigenic perspective, we distinguished between the omics-specific peripheral and core regions, and explored their differential scales and connectivity for cancers. Then, we provided network-based framework multi-omics periphery and core (MOPC) for integrated analysis of cancers. We determined that core genes are specific while peripheral genes are shared between multiple omics aspects. Meanwhile, peripheral genes irregularly surround and regulate other omics cores. The integrated multi-omics neighbourhood of cancer displays a button-like structure. Finally, we take MOPC as an omnigenic neighbourhood. Its characteristics are described through biological profile verification, pathway enrichment analysis, eQTL regulatory relationship analysis, and cancer relationship quantification. We explored the relative contribution of MOPC to the commonalities between cancers, and explained cancer–cancer relationships. MOPC provides a network-based, and practical tool for omnigenic analysis of cancers.

## Results

### Omics-specific omnigenic patterns of cancer

To study cancer’s omnigenic pattern, we curated cancer multi-omics data from UCSC Xena^53^, including a publicly-available samples of four types of omics aspects (transcriptome differential expression, DNA differential methylation, somatic mutation, and copy number variation) for 15 cancers (see Supplementary Table 1). We used indicator fold change to measure the degree of each gene’s perturbation in the transcriptome and methylation, and indicator frequency to measure the gene’s perturbation in the somatic mutation and CNV (Supplementary Table 3). To ensure experimental accuracy and feasibility, we selected the top 25% of the perturbed genes in each omics aspect (a wide range of about 4000 genes, Supplementary Table 2) for subsequent analysis. Furthermore, we analysed the structural properties of these thousands of genes perturbed in a certain study from the perspective of connectivity significance in the network (human interactome, Supplementary Table 4) to get an Omics-Specific Omnigenic Pattern (OSOP). The omnigenic pattern was constructed based on the wave mode of the connectivity significance (Largest Connected Component, LCC z-score) of genes as different degrees of perturbation were considered. We developed a unified network-based framework, *CLine* (Connectivity Line, see ‘Methods’), to pinpoint the omics-specific omnigenic pattern. *CLine* shows gene connectivity in regular fluctuation with the varying perturbation degrees in the network.

**Fig. 1:**
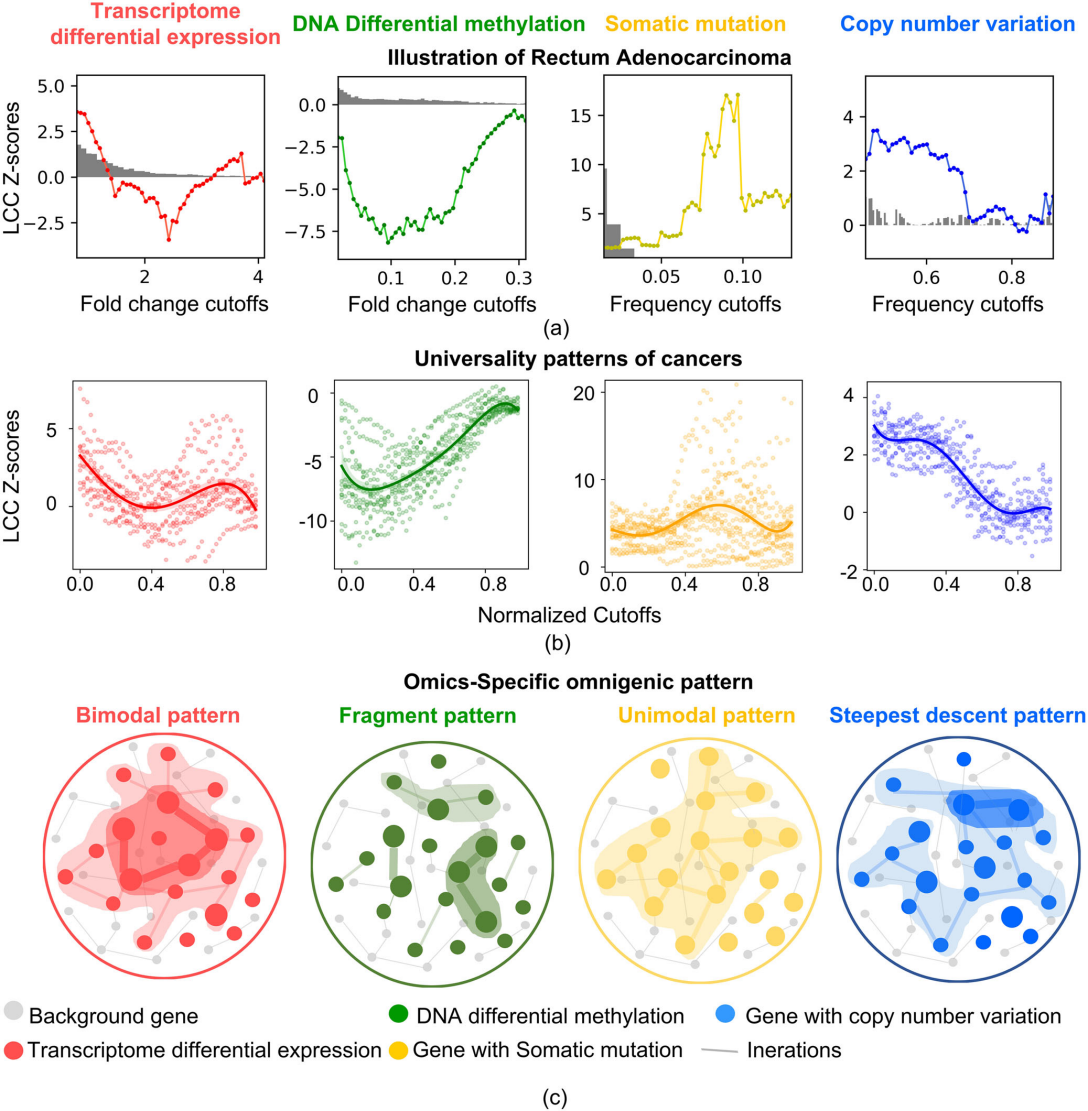
Omics-specific omnigenic pattern of cancer. **a** Network-based Connectivity Line, *CLine*. Illustration of Rectum Adenocarcinoma (READ). The perturbed genes in the transcriptome, methylation, somatic mutation and CNV aspects present an omics-specific omnigenic pattern. The abscissa is the cutoffs of fold change and frequency index, and the ordinate calculates the connectivity significance LCC z-score. i. The *CLine* of the transcriptome aspect forms a bimodal pattern. In the low perturbation cutoff part (*f*_*i*_ = 0.8), the LCC z-score of 3674 genes forms the first local peak in the line (z-score = 3.58). In the high perturbation cutoff part (*f*_*i*_ = 3.7), the LCC z-score of 147 genes forms the second local peak (z-score = 1.28). ii. The *CLine* of the methylation aspect presents a fragmented pattern, and all LCC z-scores are below 0 regardless of the low or high perturbation cutoffs. iii. The *CLine* of the somatic mutation aspect presents a unimodal pattern. When *f*_*i*_ = 0.09, the LCC z-score = 17.03, which forms the only peak in the line. iv. The *CLine* of CNV aspect presents a steepest descent pattern. When the cutoffs slightly increase from 0.68 to 0.70, LCC z-scores drop from 1.92 (significant connectivity) to 0.3 (quasi-random) in the high perturbation cutoff part. The grey histograms represent the distribution of the number of genes under different cutoffs. **b** Universality of pattern. Least squares fitting was used to construct a uniform curve across cancers (*UCurve*), highlighted by heavy continuous lines in the normalised coordinates. The red *UCurve* fits bimodal *CLine* in 66.7% of cancers; the green *UCurve* fits the fragment *CLine* in 86.7% of cancers; the yellow *UCurve* fits the unimodal *CLine* in 93.3% of cancers; and the blue *UCurve* fits the steepest descent *CLine* in 93.3% of cancers (details in Supplementary Figs. 1–4). **c** Different omics aspects of cancer show perturbations of the biological network in different ways, forming four omics-specific neighbourhoods, each of which exhibits different connectivity characteristics. Both the node sizes and thickness of edges are proportional to the strength of the perturbation. We highlighted the weakly-perturbed peripheral region with light shades, and the highly-perturbed core region with dark shades.

We took rectum adenocarcinoma (READ) as a typical example, and selected the top differentially-expressed genes (DEGs, 4033 genes). As Fig. 1a shows in red, the *CLine* forms a ‘bimodal pattern’. In the low perturbation part (log_2_(FC) cutoff = 0.8), the curve forms the first local peak. An LCC with significant size (LCC z-score = 3.58) is formed by 3674 genes that are weakly perturbed by READ, suggesting that they form a statistically-detectable connected region in the network. In the high perturbation part (log_2_(FC) cutoff = 3.7), *CLine* forms a second local peak. A statistically-detectable connected region (LCC z-score = 1.28) is also formed by 147 genes that are highly perturbed by READ. These two regions correspond to the peripheral and core regions, respectively, from an omnigenic perspective. *CLine*’s wave mode, which we called an omnigenic pattern, interprets the formation of the peripheral and core regions of the DEGs, and indicates their identifiability in the network.

For the other three omics aspects, DNA differential methylation (methylation), somatic mutation, and copy number variation (CNV), we observed that the methylation *CLine* conforms to a ‘fragment pattern’, the somatic mutation *CLine* shows a ‘unimodal pattern’, and the CNV *CLine* presents a ‘steepest descent pattern’. In methylation, all LCC z-scores were <0 in the whole *CLine*, indicating that the connectivity of the differentially-methylated genes (DMGs) was, unexpectedly, worse than random. Even if different scales with different cutoffs were considered, the DMGs tended to be scattered and avoid interaction, showing a fragmented pattern. In the weakly-perturbed region, as the fold change cutoffs progressively increase, the LCC z-score decreases from −1.95 to the minimum of −8.16 (log_2_(FC) cutoff = 0.095). Then the curve gradually rises and reaches the global maximum z-score of −0.34 in the highly-perturbed region (log_2_(FC) cutoff = 0.29). This characteristic wave mode of *CLine*, with an upward trend but significantly lower throughout, indicates the omnigenic pattern of the methylation is fragmented. The weakly-perturbed genes form a fragmented peripheral region. Comparatively highly perturbed genes form a quasi-random connected core region.

The *CLine* of somatic mutation has a unimodal pattern. As the perturbation degree cutoffs go from low to high, the LCC z-scores increase and then decrease, showing a single peak. Initially, the low-frequency cutoff is 0.015 and the LCC z-score is 1.56. The LCC z-score gradually rises to a maximum of 17.03 at the high perturbed frequency cutoff of 0.09. Afterwards, the LCC z-score continues to reduce to a lower level (z-score = 6.89 at a frequency cutoff of 0.13). Forming one dominant peak in the highly perturbed part, this characteristic of *CLine* indicates the omnigenic pattern of the somatic mutation is unimodal. The highly-perturbed genes form a remarkable connected core region.

CNV’s *CLine* has the steepest descent pattern. At the beginning, when the low-frequency cutoff = 0.46, the LCC z-score = 2.44. As the cutoff increases, the LCC z-scores remain steady. When the cutoffs slightly increase from 0.68 to 0.70, the LCC z-scores drop precipitously from 1.92 (significant connectivity) to 0.3 (quasi-random), and a step phase change occurs at a high cutoff. This characteristic *CLine* wave mode indicates that CNV’s omnigenic pattern has the steepest descent. A well-connected peripheral region exists at low perturbed cutoffs. As connectivity abruptly reduces, the disconnected core region is hard to discover at a high perturbed cutoff.

### Universal omnigenic patterns across cancers

We used the *CLine* framework across 15 cancers in four omics aspects, 60 tests overall. We applied four rigorous criteria to decide whether *CLine* in a test agreed with the corresponding omnigenic pattern’s wave mode. The key indicator d_*ratio*_ (see ‘Methods’) quantified the *CLine* curve’s amplitude ratio at low, medium, and high perturbation degree cutoffs. Then, we achieved agreement rates of 66.7%, 86.7%, 93.3%, and 93.3% for the *CLine* of 15 cancers in the transcriptome, methylation, somatic mutation, and CNV studies, respectively (Supplementary Figs. 1–4, Supplementary Table 5). This suggests that most of the cancers conformed to the corresponding common wave mode in *CLine*. OSOP presents its universality, that is, omics aspects have specific universal connectivity patterns across cancers. To denoise and highlight OSOP, we constructed a Uniform Curve across the cancers based on the fitting curve (*UCurve*, Fig. 1b, Supplementary Figs. 1–4) to indicate the shared wave mode of multiple *CLine*s. For providing uniform coordinates for multiple cancers, we normalised their perturbation degree cutoffs (see ‘Methods’). Figure 1c visually describes the corresponding disease neighbourhoods.

### Distinction of peripheral and core regions

From the omnigenic perspective, Boyle et al.^7^ proposed that diseases are directly affected by a few core genes and indirectly affected by many peripheral genes. Therefore, the disease neighbourhood structurally comprises peripheral and core regions. Any expressed peripheral gene influences disease by regulating core genes. Relatively speaking, core genes produce strong disease perturbations while peripheral genes produce weak perturbations. The conceptual distinction between peripheral and core regions in the human interactome is useful for understanding cancers. We used the network-based *UCurve* framework to further distinguish the peripheral and core regions. First, we selected the LCC of perturbed genes corresponding to a high cutoff with the local maximum LCC z-score in the *UCurve* as the core region. Core genes produce strong perturbations and form a local connected subnetwork. For core regions, the LCC was selected for transcriptome omics aspect corresponding to the second peak, for somatic mutation omics aspect corresponding to the single peak, and for fragmented highly perturbed genes in methylation and CNV omics aspects, the LCCs were selected with maximum z-scores in the high perturbation cutoff part. Next, we identified peripheral regions based on genes that were widespread in the human interactome and were either cancer-related or showed connectivity. In practice, to facilitate subsequent research and reduce computational complexity, we uniformly selected the LCC formed by the top 1500 genes (see Supplementary Materials) as the cancer neighbourhood, where several criteria were met: (i) *CLine* identified them with a low perturbation cutoff, and significant connectivity is guaranteed, except for methylation aspect; (ii) biological enrichment analysis (see Supplementary Materials) revealed their association with cancer; and (iii) these 1500 genes, providing a wide range across the whole genome, were perturbed to a certain degree regardless of the omics aspect (details of perturbation degree in Table 1). Finally, by the LCC of the top 1500 genes, the peripheral region was defined as the rest, after removing the core. Then, according to *UCurve*, we obtained the peripheral and core regions for each of the four omics aspects for 15 cancers (Supplementary Tables 1, 6).

**Table 1:** Degree of perturbation of the omnigenic neighbourhood in multi-omics aspects.

Cancers	Transcriptome |log_2_(FC)|^a^	Methylation |log_2_(FC)|^a^	Somatic mutation Frequence^a^	CNV Frequence^a^
BLCA	7.732, 1.279	0.459, 0.141	0.508, 0.025	0.730, 0.583
BRCA	8.063, 1.344	0.411, 0.113	0.317, 0.007	0.779, 0.640
CHOL	10.639, 2.546	0.473, 0.060	0.333, 0.028	0.861, 0.639
COAD	9.577, 1.360	0.531, 0.141	0.719, 0.037	0.727, 0.574
ESCA	6.333, 1.119	0.376, 0.088	0.870, 0.016	0.815, 0.690
HNSC	7.418, 1.196	0.429, 0.120	0.713, 0.016	0.761, 0.580
KIRC	8.251, 1.501	0.369, 0.074	0.535, 0.009	0.896, 0.345
KIRP	7.349, 1.330	0.407, 0.064	0.143, 0.012	0.701, 0.601
LIHC	9.258, 1.123	0.422, 0.136	0.332, 0.035	0.770, 0.565
LUAD	9.016, 1.419	0.346, 0.092	0.521, 0.028	0.758, 0.622
LUSC	9.900, 1.944	0.418, 0.113	0.792, 0.034	0.912, 0.774
PRAD	6.801, 0.915	0.413, 0.100	0.142, 0.006	0.622, 0.230
READ	10.104, 1.438	0.587, 0.131	0.852, 0.025	0.897, 0.661
THCA	8.645, 0.912	0.374, 0.028	0.589, 0.002	0.186, 0.058
UCEC	7.625, 1.634	0.522, 0.135	0.649, 0.048	0.481, 0.336

^a^Maximum and minimum values.

As previously shown, not all *CLines* agreed with the criteria of the corresponding OSOP’s wave mode, possibly because of the small sample size, uneven distribution, or noise in the data. For example, the failed *CLine* of cholangiocarcinoma (CHOL), based on the somatic mutation data, is usually caused by too few samples and an uneven data distribution (Supplementary Fig. 3, Supplementary Table 1). The failed *CLine* of lung adenocarcinoma (LUAD) obtained by CNV (Supplementary Fig. 4) increased suddenly and rose sharply to a sky-high LCC z-score when it approached the highest cutoffs. This noise is caused by a highly-perturbed motif (intrinsic triplet), which delays the steepest descent pattern. In such cases, we used the cwDTW algorithm^54^ to map a failed *CLine* to the corresponding uniformed *UCurve* (Supplementary Fig. 5). cwDTW solves the problem of end-to-end mapping between two signals, based on continuous wavelet transforms (CWT) and dynamic time warping (DTW). Thus, extended by cwDTW, our *UCurve* shows comprehensive advantages in exploring peripheral and core regions for all cancers: (i) a regular curve across multiple cancers is constructed in unified coordinates and highlighted OSOP; (ii) cwDTW extends the adaptability of *UCurve*; and (iii) the core region is the LCC corresponding to a high perturbation cutoff with the local maximum z-score in the *UCurve*. This is a consolidated method of defining the core, thus eliminating data differences while maintaining various core scales across cancers (sizes of cores shown in Table 2).

**Table 2:** The size of the core and peripheral regions of 15 cancers in four omics aspects.

Cancers	Transcriptome^a^	Methylation^a^	Somatic mutation^a^	CNV^a^	Multi-omics neighbourhood^a^
BLCA	16, 1012	21, 666	25, 1081	8, 952	70, 3273
BRCA	36, 969	9, 733	16, 1077	451, 524	503, 2822
CHOL	24, 877	10, 830	42, 1058	23, 961	93, 3266
COAD	12, 899	20, 664	23, 1018	41, 951	95, 3038
ESCA	7, 1001	9, 639	13, 989	8, 928	35, 3133
HNSC	19, 952	21, 573	16, 1049	134, 785	187, 2948
KIRC	17, 892	7, 643	6, 1114	42, 956	69, 3164
KIRP	18, 838	5, 700	18, 981	279, 709	317, 2826
LIHC	60, 950	10, 592	30, 1035	448, 535	539, 2688
LUAD	47, 841	15, 692	14, 950	361, 607	426, 2600
LUSC	10, 926	7, 599	117, 845	8, 972	142, 2949
PRAD	145, 745	10, 666	26, 1020	8, 977	185, 2967
READ	16, 885	9, 684	22, 972	75, 902	121, 2969
THCA	11, 959	7, 778	15, 1029	112, 888	144, 3177
UCEC	20, 921	10, 732	12, 1053	13, 989	55, 3238

^a^Number of core genes, number of peripheral genes.

**Fig. 2:**
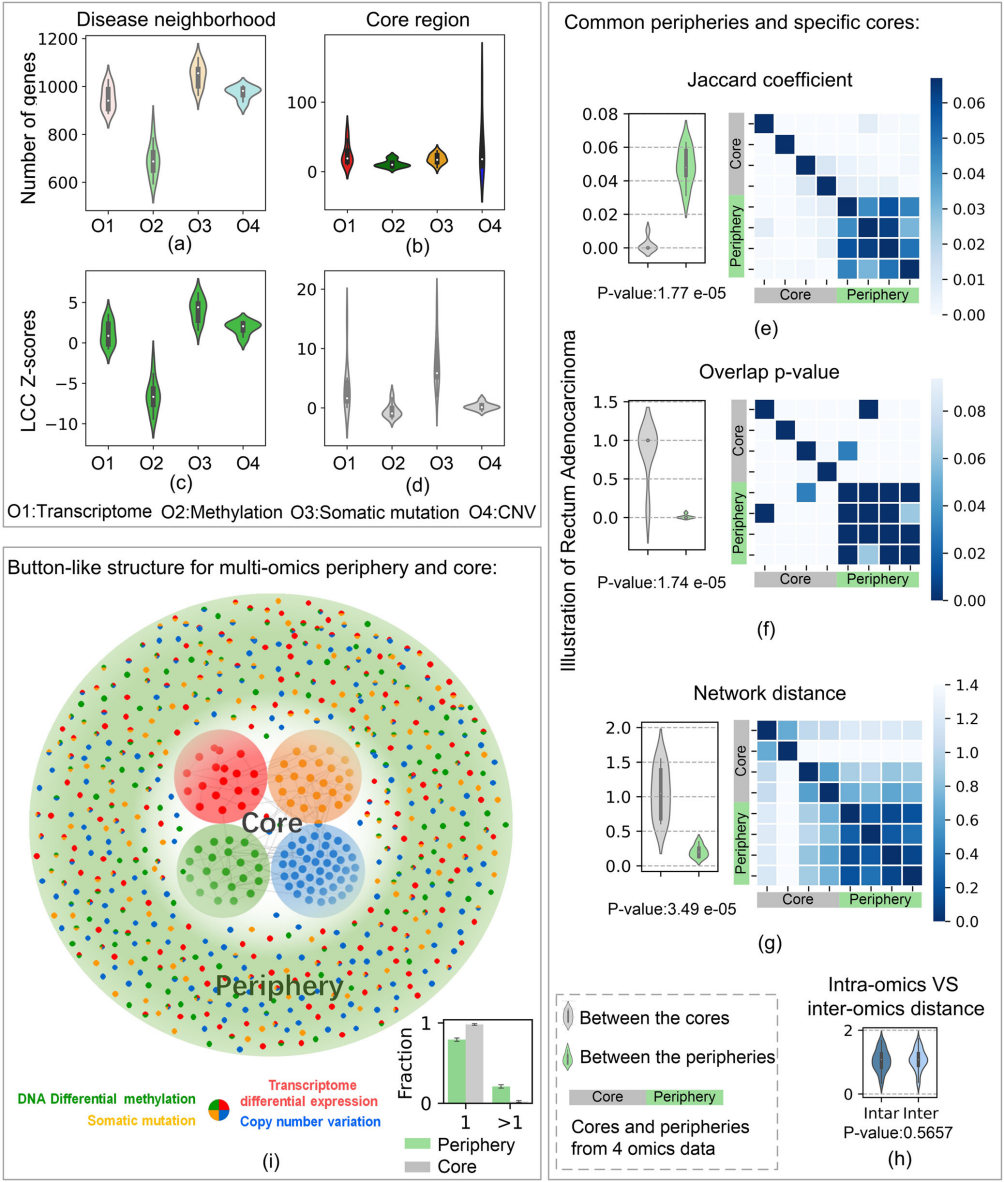
Relationship between multi-omics periphery and core. **a**–**d** Distinction of peripheral and core regions of four omics aspects (O1, O2, O3, and O4). **a** The violin chart shows the scales of the neighbourhoods (Table 2) across 15 cancers. The average sizes are 942, 691, 1044, and 976, respectively (median: 936, 687, 1046, and 983). **b** Of these, the scales of the core regions average 31, 11, 26, and 134, respectively (median: 18, 10, 18, and 42). **c** We show the LCC z-scores of neighbourhoods across 15 cancers. The average sizes are 0.88, −5.67, 4.01, and 1.93, respectively (median: 0.76, −6.37, 4.2, and 2.08). **d** LCC z-scores of cores shown with average values of 2.53, −0.62, 7.83, and 0.63 (median, 1.09, −1.03, 7.38, and 0.21). **e**–**g** Common peripheries and specific cores. Taking READ as an example, four peripheral and four core regions were obtained from different omics aspects. Network proximity: Jaccard coefficient (JAC), Overlap *p*-value (hypergeometric test), and network distance between any two regions are shown in the heat maps. The overlap between the peripheral region is large (average JAC = 0.048, *p*-value = 0.01) and the distance is close ($s_{AB}$ = 0.21), while the core regions are basically non-overlapping (average JAC = 0.017, *p*-value = 0.25) and far away ($s_{AB}$ = 1.05). Correspondingly, grey or green violins display the network proximity values among cores or among peripheries. We noticed significant differences between these violin pairs (Mann–Whitney U test *p*-values, 1.77 × 10^−5^, 1.74 × 10^−5^, and 3.49 × 10^−5^). **h** The blue violin describes the network distance between the core and peripheral regions of the same omics aspect; the light blue violin collects the network distance between the core and peripheral regions from different omics aspects. There is no significant difference between these two sets (Mann–Whitney U test *p*-value, 0.5657). **i** Button-like structure of the omnigenic neighbourhood. The peripheral regions (highlighted in light green) are common, and the core regions (highlighted in four colours) are specific to the four omics aspects. A node is marked with multiple colours, indicating that it is perturbed in multiple omics aspects. The bar chart shows the number of omics aspects in which a peripheral or core gene is perturbed. The results indicate that most core genes (98%) are perturbed in specific omics aspects, and a certain percentage of peripheral genes (21%) and a few core genes (2%) tend to be perturbed in multiple omics aspects. The error bars indicate the 95% confidence intervals.

Furthermore, we thoroughly analysed the scale and connectivity significance of the peripheral and core regions. First, the average sizes of neighbourhoods across cancers in the transcriptome, methylation, somatic mutation, and CNV studies were 942, 691, 1044, and 976, respectively (Fig. 2a). The cancer neighbourhood’s smallest scale in the methylation aspect indicates weak regulation between methylation sites. DNA methylation is an epigenetic mechanism and mainly serves as a repressive or activating mark for gene expression. The average sizes of the four omics cores were 31, 11, 26, and 134, respectively (Fig. 2b). Then, we compared the connectivity significance of the cancer neighbourhoods and core regions between different omics aspects. The average LCC z-scores of neighbourhoods across the 15 cancers were 0.88, −5.67, 4.01, and 1.93, respectively (Fig. 2c), and the average LCC z-scores of the cores were 2.53, −0.62, 7.83, and 0.63, respectively (Fig. 2d). Among them, cancer neighbourhoods in the somatic mutation and CNV studies were more detectable, while the cancer neighbourhood in the methylation study was completely randomly distributed across the network. In addition, core regions in the transcriptome and somatic mutation studies formed detectable connected subgraphs, and core regions in the methylation and CNV studies tended to scatter randomly. Therefore, different strategies should be adopted to analyse the disease neighbourhoods of different omics studies. Our *CLine* and its uniform *UCurve* determined common structural properties across cancers and discriminated the differential connectivity pattern between multiple omics studies.

### Relationship between multi-omics periphery and core regions

The ultimate aim of these omnigenic patterns is to integrate multiple omics aspects and provide a network-based platform for characterising the MOPC of cancer. The *UCurve* framework has helped us construct omics-specific peripheral and core regions. However, the relationship between these omics aspects remains unclear, hampering further integrated analysis based on MOPC.

In this respect, we quantified the relationship between the multi-omics core and peripheral regions based on their network proximity^1^. Gene sets that were proximal in the network tended to have similar biological functions and pathogenicity. We used three indicators (see ‘Methods’) to quantify network proximity and show relationships between different regions: (1) the Jaccard coefficient; (2) the statistical significance *p*-value of overlap; and (3) the distance in the network. These indicators quantified the amount of overlap between these regions, whether the degree of overlap was significantly higher than random, and the shortest-path proximity in the network, respectively. Taking rectum adenocarcinoma as an example, we found a lower overlap and longer distance between the core regions than expected, indicating that core regions tend to be highly and specifically perturbed in one omics aspect. In addition, the large overlap and short distance between the peripheral regions of different omics aspects indicated that peripheral regions tend to be weakly but diversely perturbed in multiple omics aspects (Fig. 2e–g). After performing tests for all cancers (see Supplementary Figs. 7–9), we discovered that the high perturbation of core genes usually observed in one omics aspect has independent characteristics, while peripheral genes can be simultaneously perturbed in multiple omics aspects.

Furthermore, to test whether the core region of an omics aspect is significantly close to the peripheral region of the same omics aspect, we compared the network distance between the core and peripheral regions (Fig. 2h). We found that the network distance between the intra-omics core and peripheral regions is almost indistinguishable from the inter-omics values (Fig. 2h, Mann–Whitney U Test *p*-value = 0.5657). We observed the phenomenon that peripheral genes not only surround their own core genes, but are widespread in the network, likewise regulating core genes of other omics aspects. Thus, we found some relationships between peripheral and core regions: (i) core genes are specific between multiple omics aspects, and core genes are highly perturbed in one omics aspect; (ii) peripheral genes are weakly perturbed, but are influenced in multiple omics aspects; and (iii) omics perturbations interact with each other, and peripheral genes irregularly surround and regulate core genes of multiple omics aspects. This result shows the intricate regulatory interactions among and between omics perturbations.

Based on these observations, we proposed a visual button-like structure to describe the multi-omics neighbourhood in an integrated way. The disc is shared by the peripheral regions, and the four holes correspond to the independent cores of different omics aspects (Fig. 2i). The button-like structure characterises cancer’s integrated MOPC, where the periphery and core comprise the peripheral and core regions, respectively, from the four omics aspects. Furthermore, we compared the number of omics aspects involved in each peripheral and core gene set (Fig. 2i), and showed that 21% of peripheral and 2% of core genes are perturbed concurrently in multiple omics aspects. This means that cancer tends to affect different genes in different omics aspects, and the trend is more pronounced as the degree of perturbation gets bigger. We provided the integrated MOPC as a multi-omics neighbourhood (Table 2), which offers insights for network-based mechanisms of cancer.

### Applications of multi-omics periphery and core in cancers

We take the MOPC as an omnigenic neighbourhood. To study how the MOPC describes cancer’s biological characteristics, we performed biological profile verification, enrichment analysis, eQTL regulatory relationship analysis, and cancer relationship research.

**Fig. 3:**
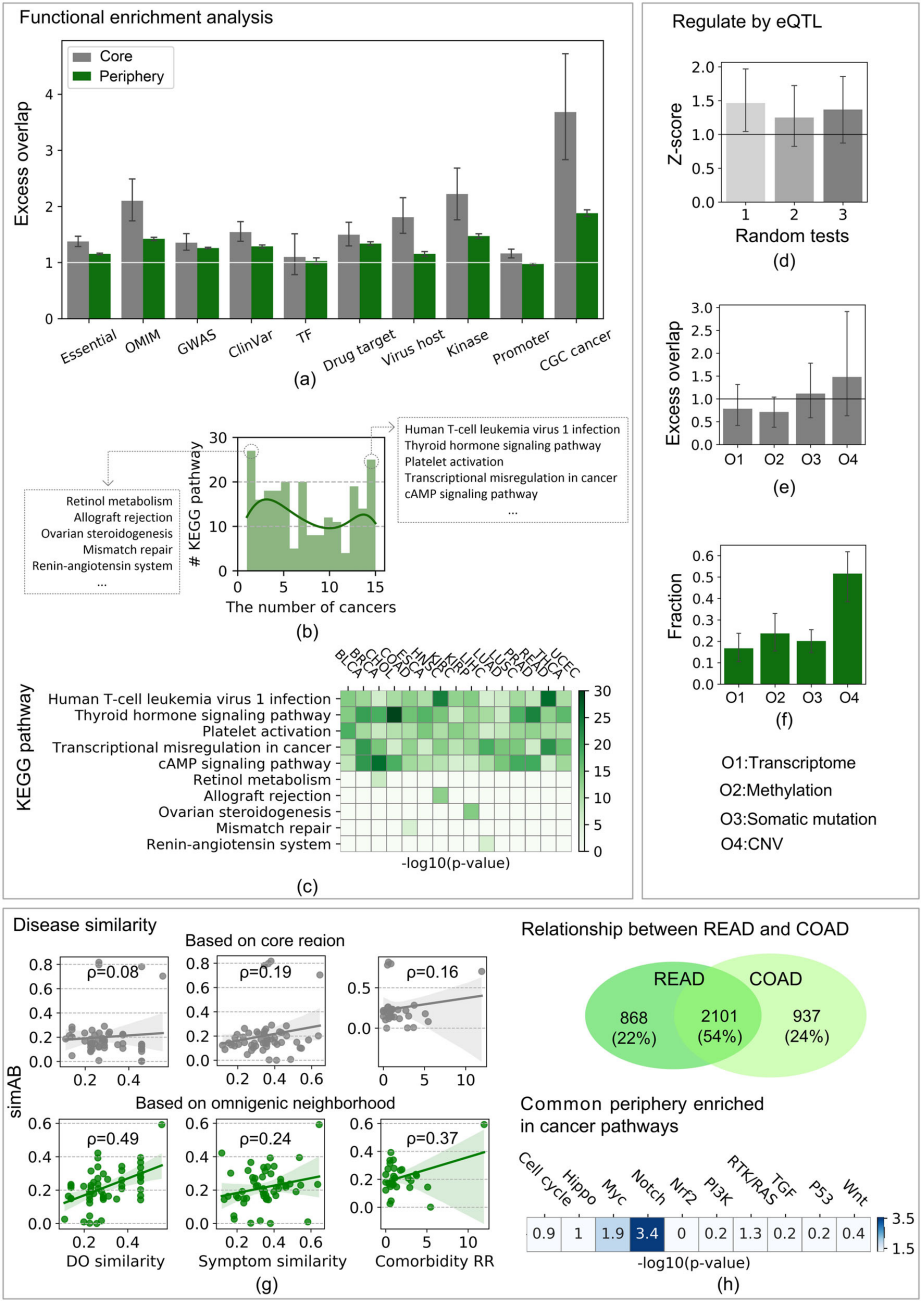
Characteristics of multi-omics periphery and core (MOPC). **a** Functional profiles. The excess overlap between the peripheral and core region was calculated with ten biological functional datasets. The items with index excess overlap >1 (excess white line) mark the functional profiles of the peripheral and core regions. The error bar corresponds to the fluctuation of the results across 15 cancers. The results of peripheral regions (excess overlap >1, green bar) are slightly weaker than those of the core regions (grey bar), showing that their functionality should not be ignored. **b** The distribution of the number of cancer MOPCs enriched in KEGG pathways. **c** Enriched pathways shared or specific among cancer MOPCs. The ten representative pathways are focused on and their enrichment results are displayed (−log_10_(*p*-value), hypergeometric test). **d**–**f** Regulatory relationship analysis in eQTL, based on *cis-*eQTL and *trans-*eQTL in the PancanQTL database (see ‘Methods’). **d** We counted the number of core genes directly regulated by peripheral genes. The z-score represents the statistical significance compared with that of 1000 random experiments. **e** We counted the number of core genes regulated by eQTL. The excess overlap values between the cores and the ground truth *egene* (see ‘Methods’) are given. The CNV (O4) core has the most excess overlap with *egene* (mean, 1.5). **f** We counted the number of peripheral genes, which regulate core genes, and showed the proportion of peripheral genes that are responsible. The periphery of CNV (O4) accounts for the largest proportion (average, 0.52). **g** The omnigenic neighbourhood portrays cancer similarity. We used simAB to calculate the relationship between cancers (‘Methods’), where the grey image represents the result of the cancer similarity analysis based on core genes, and the green image represents the result of the cancer similarity analysis based on the omnigenic neighbourhood. The points represent the similarities between cancers. We verified the results by comparison with DO similarity, symptom similarity, and comorbidity RR. The fitted line is the Pearson correlation coefficient between the predicted and known similarity between cancers. The shading indicates the 95% confidence interval. In the three similarity verification experiments, based on the omnigenic neighbourhood, the Pearson correlation coefficient increased by 6.12-fold, 1.26-fold, and 2.31-fold, respectively. **h** Common mechanism of rectum adenocarcinoma (READ) and colon adenocarcinoma (COAD) in the periphery. The Venn diagram shows the number of overlapping peripheral genes between COAD and READ (2101, Jaccard coefficient = 0.54). The heat map is also used to show the enrichment results (−log_10_(*p*-value), hypergeometric test) on the shared peripheral genes in ten classic cancer signalling pathways^42^ that frequently undergo gene variations. The error bars indicate the 95% confidence intervals.

First, to mark the functional profiles of the peripheral and core regions, we analysed the excess overlap between genes in ten biological datasets (Table 3, Supplementary Table 7) and the peripheral and core genes (see ‘Methods’, Fig. 3a). The average values across the cancers all exceeded 1, indicating that genes in the MOPC have biological significance. A significant excessive overlap of peripheral genes on OMIM, GWAS, and ClinVar indicated that they are potentially disease-related genes, and the significant excessive overlap on drug targets indicated that they can be screened for cancer treatment. In addition, the excessive overlap of peripheral genes on the Cancer Gene Census (CGC) was very high (average excess overlap = 1.88), which shows that peripheral genes likely lead to cancer development.

**Table 3:** Datasets for biological profile verification.

Dataset	Gene number	source
Essential	7935	DEG: http://tubic.tju.edu.cn/deg/
OMIM	2266	OMIM: https://omim.org/
GWAS^74^	6271	The new NHGRI-EBI Catalogue of published genome-wide association studies (GWAS Catalogue). PMID: 27899670
ClinVar^75^	5428	ClinVar: Public Archive of Relationships Among Sequence Variation and Human Phenotype. PMID: 24234437
TF^76^	1610	The human transcription factors. PMID: 29425488
Drug target^37^	2256	Network-based prediction of drug combinations. PMID: 30867426
Virus host	947	CCSB: http://interactome.dfci.harvard.edu/V_hostome
Kinase	514	http://kinase.com/human/kinome
Promoter^77^	3934	A high-resolution map of active promoters in the human genome. PMID: 15988478
CGC cancer^78^	555	A census of human cancer genes. PMID: 14993899

Second, we performed KEGG enrichment analysis on the MOPC, by the Over-Representation Analysis (ORA) in ConsensusPathDB (http://consensuspathdb.org/). We collected 225 KEGG pathways enriched in cancers (hypergeometric test *p*-value < 0.01, Supplementary Table 8) and showed the distribution of cancers involved in each pathway (Fig. 3b). Either specific (29% ≤ 3 cancers) or corresponding (54% ≥ 7 cancers) pathways existed between cancers. In particular, 27 pathways were enriched in a specific cancer while 25 pathways were involved in all cancers. Among them, we found generalised cancer mechanisms, including human T-cell leukaemia virus 1 infection^55^, the thyroid hormone signalling pathway^56^, platelet activation^57^, transcriptional misregulation in cancer, and the cAMP signalling pathway^58^. For specific mechanisms, we found that retinol metabolism reduces cholangiocarcinoma risk^59^, allograft rejection occurs in kidney renal clear cell carcinoma^60^, ovarian steroidogenesis inhibits liver hepatocellular carcinoma^61^, *MSH* and *MLH1* gene alterations in mismatch repair increase the oesophageal carcinoma risk^62^, and lung adenocarcinoma^63^ influences renin–angiotensin system gene expression. We visualised these representative pathways (−log_10_(*p*-value), hypergeometric test) in Fig. 3c. This result shows that different cancers participate in some common and specific pathways, suggesting that different cancers participate in the same pathway through shared peripheral regions, which establishes relationships between cancers. Similar results exist for GO terms and reactome pathways (see Supplementary Fig. 10a–d and Supplementary Table 8).

To explore the regulatory relationship between peripheral and core genes in the MOPC, we used the PancanQTL database’s eQTL cancer data^64^, including significant data on cis-eQTL and trans-eQTL regulatory effects. The significantly regulated genes are called *egene* as the ground truth. For 15 cancers, the number of core genes regulated by peripheral genes was higher than random genes (z-scores >1 in Fig. 3d), indicating that the cancer periphery tends to work by regulating the core. Furthermore, to determine differences in the degree to which eQTL regulates the cores, we calculated the excess overlap between the cores and *egene*, and found that multiple omics cores were subject to eQTL. The CNV core was among the most regulated regions (Fig. 3e). Finally, to determine the roles of different peripheries in core regulation, we calculated the proportion of peripheral genes occupying the eQTL. The results show that the variation that regulated the core genes was mainly occupied by the methylation and CNV peripheries (Fig. 3f). Through these experiments, we observed that, in the cancer neighbourhood, the variation in the peripheral genes significantly regulates core gene expression levels, which directly affects cancer. Core genes account for only a few of the total number of genes, so the role of peripheral genes should not be ignored in understanding cancer.

Finally, we hypothesised that cancers describe their similarities through their peripheries. We applied the MOPC to obtain their relationship. We first obtained three convincing cancer similarity datasets for verification, including disease ontology (DO) similarity^65^, symptom similarity^66^, and comorbidity data^67^ (see ‘Methods’). We screened these three types of similarity data based on the 15 cancer multi-omics aspects, and obtained 55 pairs of DO similarity values among 11 cancers, 54 pairs of symptom similarity values among 11 cancers, and 29 pairs of comorbidity relative risk (RR) values among 10 cancers. Moreover, we used two methods to predict cancer similarity based on MOPC: (1) disease similarity *simAB*, and (2) Jaccard coefficient (see ‘Methods’). Furthermore, we calculated the Pearson correlation coefficient between the predicted values and the ground truth similarities (DO, symptom similarity, and comorbidity RR). The results showed that the similarity based on MOPC positively correlated with DO similarity, symptom similarity, and comorbidity RR (Fig. 3g, Supplementary Fig. 10e), indicating that MOPC is valid in indicating relationships between cancers. MOPC obtained greater relationship accuracy than just the core because it included the large-scale periphery. MOPC improved the correlation coefficients to 6.12, 1.26 and 2.31 times the ability of the core for each ground truth similarity, respectively. Specifically, we focused on the relationship between colon adenocarcinoma (COAD) and READ. Biologically, they are the same type of colorectal cancer. The main difference is the anatomical location. Therefore, a sophisticated method should give them a very high similarity score. Based on the cores, their similarity ranked fourth, but based on MOPC, their relationship ranked first. This illustrates the great potential of the periphery in predicting the relationship between cancers. We then focused on shared peripheral genes of COAD and READ. As the Venn diagram in Fig. 3h shows, they shared numerous overlapping peripheral genes (2101, 54%). We suggest the pathways underlying their pathology are involved in these overlapped peripheral genes. For further test, we used ten classic cancer signalling pathways that frequently undergo genetic variation^52^, including cell cycle, Hippo, Myc, Notch, Nrf2, PI3K, RTK/RAS, TGF, P53 and Wnt. We calculated the enrichment significance (hypergeometric test *p*-value, see ‘Methods’) of overlapping peripheral genes in signalling pathways. We detected the key oncogenic signalling pathways Myc and Notch, the abnormal activation of which drives colorectal cancer’s carcinogenesis. Stabilising c-Myc promotes colorectal carcinogenesis and glucose metabolism^68^. Meanwhile, therapies have potential in abrogating Notch signalling and, thus, inhibiting colorectal cancer development and progression^69^. The presence of the Myc and Notch signalling pathways in the periphery suggests that the weakly-perturbed periphery also contains significant cancer signalling molecules.

The genetic architecture of diseases describes the number of genomic variants that contribute to risk of disease and their effect size distribution. Cancer is polygenic but some mutations are drivers and have large effect^70^. Therefore, network medicine consortium, based on polygenic model, detects mesoscopic scale module formed by driver genes. Omnigenic model takes it further that most variants contribute to risk of cancer. To present the outstanding characteristics of omnigenic neighbourhood, we compare it with three representative polygenic modules. We gather network-based DIAMOnD^8^ modules, a group of cancer driver genes from Broad Institute of MIT and Harvard^71^ (indicated by Driver(1), Supplementary Table 9) and another group of cancer driver genes from Pan-Cancer Analysis of Whole Genomes (PCAWG) Consortium^72^ (indicated by Driver(2), Supplementary Table 10) (Details see Supplementary Materials section 7).

1. We present the overlap between omnigenic neighbourhood and these three polygenic modules (Supplementary Fig. 11). We find that the induced intermediate DIAMOnd gene set has significant overlap (hypergeometric test *p*-value < 0.01) with peripheries of multiple omics. For Driver(1) and Driver(2) were identified based on somatic mutation, they are only significant overlap with core and periphery of Somatic mutation data. The remarkable thing is that their significant overlap with the periphery are higher than with the core. These all are compatible with the importance of the periphery.
2. We do KEGG pathway enrichment analysis of omnigenic neighbourhood and polygenic modules (Supplementary Fig. 12). It is observed that about 30–50% KEGG pathways, enriched by polygenic modules (hypergeometric test *p*-value < 0.01), are reinforced by omnigenic neighbourhood. Biological functions of polygenic modules need to be supplemented by the periphery. There are functional pathways enriched only by peripheries, and the common pathways which are enriched by several peripheries have an underlying association with cancer (validated in literature). The functional pathways that indirectly affect cancers are embedded in their common peripheries. On the other hand, the significance of enrichment reduces under the removal of one omics data. This indicates that each omics offers its own contribution to the understanding of cancer. For example, if the Methylation core is removed, the multi-omics core will ignore the relationship between cancer and inflammatory mediator regulation of TRP channels (more examples in Supplementary Table 11). Furthermore, we show the functional pathways enriched by a given omics core, and find that other multiple omics peripheries can reinforce these pathways. Such as Transcriptome core is reinforced by peripheries of Transcriptome, Methylation and CNV. This indicated the complex regulatory relationship between omics.
3. We test cancer similarity described by polygenic modules (Supplementary Fig. 13), and verify the results by calculating correlation coefficient with DO similarity, symptom similarity, and comorbidity RR. In 89% (8 in 9) tests, the omnigenic neighbourhood achieves higher correlation coefficients than polygenic modules, present a greater ability to portray the relationship between cancers. Meanwhile, different omics contribute differently to the similarity. Transcriptome and Methylation play a more critical role in portraying the relationship (Supplementary Fig. 14).
4. We do drug targets enrichment analysis of omnigenic neighbourhood (Supplementary Fig. 15). 229 protein targets of 72 approved drugs for 12 cancers (from repoDB database^73^, details in Supplementary Table 12) are harvested as ground truth. Among multi-omics core, DIAMOnD module, Driver(1) and Driver(2), only DIAMOnD module contains significantly more drug targets. Nevertheless, as long as we look at the periphery, considerably more drug targets can be detected. Omnigenic neighbourhood is the region with the largest number (average 70.3 across cancers) and the strongest significance (-log10(p-value) average 5.55, hypergeometric test) of drug targets. Again, different omics contribute differently to drug targets. Transcriptome and Somatic mutation plays a crucial role in portraying more drug targets.

Overall, omnigenic neighbourhood presents three outstanding characteristics than polygenic modules. First, it enhances and identifies underlying functional pathways of cancer. Second, it puts forward a greater ability to portray the relationship between cancers. Finally, it accommodates a greater variety of drug targets, offer a methodological neighbourhood for explaining drug therapeutic effects through the interactome.

## Discussion

We studied the omnigenic pattern, which is constructed based on the wave mode of the connectivity significance of cancer genes while considering different degrees of perturbation. We developed a unified network-based pattern *CLine* that pinpoints the OSOP across 15 cancers.

The mesoscopic scale disease module^1^ focuses on connected subgraphs formed by these strongly cancer-relevant core genes. Previous work^1^ used incomplete PPI networks to explain why disease modules are unconnected. We observed that the connectivity of the cancer-perturbed genes depends on the omics aspects. Therefore, different strategies should be adopted to analyse the disease neighbourhood of different omics studies. Previous methods^8,9,74–76^ based on network proximity could only identify mesoscopic cores in transcriptome and somatic mutation aspects, and to present macroscopic cancer neighbourhoods in somatic mutation and CNV aspects. Our *CLine* and its uniform *UCurve* identify the common structural properties across cancers and discriminate the differential connectivity pattern between multiple omics aspects. We have provided a practical tool for analysing cancers from the omnigenic model in multiple omics studies.

With the huge volume of data from large-scale cancer genomics, an open challenge is to distinguish core regions, conditional on genotypes and expression levels, that have the strongest effects on cancer or with interpretable mechanistic links to cancer formation and progression. The usual assumption is that cancer-associated genes tend to cluster in the same network neighbourhood. In fact, cancer core regions do not correspond to any one well-connected component as observable modules in the present incomplete interactome. They are scattered, forming many separate components. Despite the best curation efforts, the samples and interactome remain incomplete and systematically biased toward multi-omics cancer genes and mechanisms. Therefore, not all *CLines* meet the criteria of the wave of the corresponding OSOP, which may be because of the uneven distribution of sample numbers and incomplete interactome. For example, CHOL’s *CLine* in the somatic mutation does not meet the unimodal pattern, which is largely because of too few samples (sample number 36, Supplementary Fig. 3, Supplementary Table 1), and LUAD’s *CLine* in the CNV does not meet the steepest descent pattern (Supplementary Fig. 4), which may be because of interactome noise in the highly-perturbed region. We selected the LCC of perturbed genes corresponding to a high cutoff with the local maximum LCC z-score in the *UCurve* as the core region. This network-based approach defines and indicates the cancer core. For any *CLine* that did not meet the corresponding criteria, we used the cwDTW algorithm^54^ to map it to the corresponding *UCurve*. This is an adaptive way to deal with problems such as inadequate samples and an incomplete interactome. An alternative method is by reducing the criteria through parameter α in formula 3, which defines the standards of the omnigenic pattern.

Another key problem to be solved in the MOPC is to determine how many distinct peripheral genes contribute to cancer variation. This remains a challenge in our omnigenic pattern. Because of huge differences in sample size, the scales of the regions perturbed by cancers varies greatly under the same pre-set parameters of the statistic model. Therefore, based on bio-enrichment and experience, the LCC of genes with the top 1500 perturbation degrees were selected as the cancer neighbourhood for each omics aspect. Another unsolved problem is that if most peripheral genes act through interactome networks, then what graph pattern mediates their contribution to the core region? Key master regulators, propagation paths, and direct or indirect interactions may all contribute. Moreover, for DNA differential methylation and CNV omics aspects, the highly-perturbed genes did not form a significant connected subgraph. The results are influenced by the choice of the LCC of the gene set corresponding to the local maximum of the high perturbation region of *UCurve* as the core. In particular, if the core genes of the methylated aspect do not tend to influence each other, will they regulate other omics-perturbed genes? The deep-seated relationship needs further exploration. Our pattern also raises questions about the next generation of prediction studies. The role of the omics-specific omnigenic pattern of cancer in predicting driver mutations, pathways, and gene sets (or core modules) that contribute to cancer formation, progression, and precise treatment remains an essential task for fully understanding cancer biology.

We performed data-driven pattern discovery in multi-omics data of cancer according to omnigenic model. The observed pattern is linked to the specific type of data, its distribution and noise. Indeed orthogonal verification experiments are needed to support *Cline* to go forward to a system property of the cancer. For specific disease and independent datasets, developing computational tools based on omnigenic neighbourhood to improve sample classification and drug repurposing will be an open problem in future work.

## Methods

### Material for building model

#### Human interactome

The human interactome was established from the underlying network using experimentally-documented molecular interactions in human cells from the interactome platform^1^. Protein interactions were combined from four sources: (1) binary interactions from two available high-quality yeast-two-hybrid datasets; (2) literature-curated interactions obtained by low-throughput experiments; (3) kinase–substrate pairs; and (4) signalling interactions. The data contained 16,461 genes and 239,305 physical interactions (details in Supplementary Table 4).

#### Multi-omics cancer data

The Cancer Genome Atlas (TCGA) has analysed large cohorts of over 30 human cancers through large-scale genome sequencing and integrated multi-dimensional analyses, covering publicly-available data sets including transcriptome differential expression, DNA differential methylation, somatic mutation, and copy number variation^44^. UCSC Xena was developed as a high-performance visualisation and analysis tool for both large public repositories and private datasets^53^. It organises and redevelops TCGA data, and provides interactive online visualisation of TCGA public data sets, which can help researchers to download multi-omics data of TCGA. We downloaded and used cancer multi-omics data from UCSC Xena, collating publicly-available sample datasets for transcriptome differential expression, DNA differential methylation, somatic mutation, and copy number variation of 15 cancers from TCGA (see Supplementary Materials for data preprocessing, Supplementary Table 3).

### Methods

#### Identification of the omnigenic neighbourhood of cancers

##### Omics-specific omnigenic pattern of cancers

We used the connectivity line (*CLine*) to describe the omnigenic pattern of each cancer omics aspect individually. First, for each cancer, according to the degree of perturbation in a given omics dataset, a set of equally divided cutoffs *fl* of perturbation degree was considered. According to different perturbation degree cutoffs *fi* (*fi* ∈ *fl*), we selected the cancer gene set *S*_*i*_ ($\forall j \in S_i,wj \,>\, fi$) whose perturbation degree *w*_*j*_ of any gene was greater than *fi*. As *fi* progressively increases, the stronger the cancer association of the derived gene set *S*_*i*_. We gradually narrowed down the scope from the weakly-perturbed peripheral genes to the highly-perturbed core genes. The perturbation degree values of all genes in an omics dataset were collected into a set *w*, which was ranked and divided into *t* equal bins (*t* = 50) from minimum to maximum, forming the cutoff list *fl*. Each ordered element *fi* is a perturbation degree cutoff and defined as

1 $$f_i = \min (w) + \frac{{\max (w) - \min (w)}}{t} \times (i + 1), i=0,1,2,...,t - 1,$$

where $\forall fi \in fl(i = 0,1,2...,t - 1)$. We calculated the LCC z-score of gene set *S*_*i*_ ($\forall j \in S_i,w_j \,>\, f_i$), where $w_j \in w$ reflects the perturbation of gene *j*.

Then, size (*S*_*LCC*_) of the largest connected component (LCC) of *S*_*i*_ was used to quantify the connectivity of these cancer genes in the human interactome. By comparing with the sizes (*S*_*rLCC*_) of the LCCs from 1000 random experiments, the statistical z-score was obtained to indicate the significance of connectivity of *S*_*i*_. The LCC *z-score* is given by:

2 $${{{\mathrm{LCC}}}}\,z - score = \frac{{S_{LCC} - \mu (S_{rLCC})}}{{\sigma (S_{rLCC})}},$$

where *μ*(*S*_*rLCC*_) and *σ*(*S*_*rLCC*_) represent the mean and standard deviation of the LCC size obtained from 1000 random experiments, respectively.

Finally, *CLine* was plotted with the vertical as the LCC z-score of *S*_*i*_, the abscissa as the cutoff *fi*, and a line out of 50 points (*t* = 50) (Fig. 1a). *CLine* reflects the wave mode of the connectivity between the perturbed genes corresponding to the change of the perturbation degree cutoffs (results of 15 cancers shown in Supplementary Table 5).

##### Universality of pattern

We defined different criteria for each omics aspect to measure whether the *CLine* agreed with a specific pattern. We divided the cutoffs into three perturbation parts: low (cutoffs of the first quarter), medium (cutoffs of the second and third quarters) and high (cutoffs of the last quarter), and used three sets, L, M, and H, to store LCC z-scores corresponding to these different cutoffs, respectively. The standards that we defined for the connectivity omnigenic patterns of the four omics aspects were as follows:

1. The bimodal pattern of transcriptome aspect: $d_{ratio}(L,M) \,>\, \alpha$ and $d_{ratio}(H,M) \,>\, \alpha$;
2. The fragment pattern of methylation aspect: all z-score < 1.64 (z-score = 1.64, corresponding statistical significance *p*-value = 0.05, under the standard normal distribution);
3. The unimodal pattern of somatic mutation aspect: $d_{ratio}(M,L) \,>\, \alpha$ and $d_{ratio}(M,H) \,>\, \alpha$;
4. The steepest descent pattern of CNV aspect: $d_{ratio}(L,H) \,>\, 2\alpha$;

where the key indicator *d*_*ratio*_ quantifies the amplitude ratio of the curve as:

3 $$d_{ratio}(X,Y) = \frac{{\max (X) - \min (Y)}}{{\max (ALL) - \min (ALL)}},$$

where *X*, *Y* and *ALL* are sets of LCC z-scores. *ALL* fixedly stores the LCC z-scores corresponding to all cutoffs. *X* and *Y* are used to substitute the parts L, M and H. When$d_{ratio}(X,Y) \,>\, \alpha$, it means that the amplitude ratio between parts *X* and *Y* is relative to the overall amplitude of *CLine*, indicating that the maximum value in the *X* set is significantly higher than the minimum value in the *Y* set. The higher parameter *α*, the more stringent the omnigenic pattern. We set the parameter *α* = 0.4 as the threshold of the amplitude ratio in our tests. To highlight the pattern, we used the least square method^77^ to perform polynomial fitting on the *CLine*s that met the criteria to obtain a Uniformed Curve (*UCurve*, Fig. 1b). Before fitting, in each omics aspect, we normalised the cutoff *f*_*i*_ as $\left( {f_i - \min \left( {fl} \right)} \right)/\left( {\max \left( {fl} \right) - \min \left( {fl} \right)} \right)$. Then multiple cancers could be displayed in a uniform coordinate.

In the follow-up, for any *CLine* that did not meet the corresponding criteria, we used cwDTW^54^ to map it to the corresponding *UCurve*. The cwDTW uses CWT (continuous wavelet transforms) to perform continuous wavelet transformation on *CLine* and perform z-score standardisation to obtain a curve similar to the fluctuation law of *UCurve*. At the same time, dynamic time warping (DTW) was used to find the mapping effect, which minimises the sum of the distances of all corresponding points in the two curves to map the *CLine* onto the *UCurve*. We sampled the mapping results to obtain the key points corresponding to the two curves (Supplementary Fig. 5).

#### Network proximity between peripheries and cores

We performed network proximity analysis between the core and peripheral regions of the four omics aspects. We calculated the Jaccard coefficients, the *p*-value of overlap, and the network distance between them.

##### Jaccard coefficient

For sets *A* and *B*, the Jaccard coefficient is the ratio of the size of the same element of *A* and *B* to the size of all elements of *A* and *B*. The Jaccard coefficient is in the range of 0–1. When $J(A,B) = 0$, *A* and *B* do not have the same element. When $J(A,B) = 1$, *A* and *B* are exactly the same. The larger the Jaccard coefficient value, the more identical elements, and the more similar sets *A* and *B* are.

4 $$J(A,B) = \frac{{\left| {A \cap B} \right|}}{{\left| {A \cup B} \right|}}.$$

##### Overlap *p*-value

The hypergeometric distribution is used to calculate the overlap significance of the two sets.

5 $$p(x = k) = \frac{{\left( {\frac{M}{k}} \right)\left( {\frac{{N - M}}{{n - k}}} \right)}}{{\left( {\frac{N}{n}} \right)}},$$

6 $$p - value(k) = \mathop {\sum}\limits_{i \ge k} {p(x = i)} ,$$

where *x* is a random variable and obeys the hypergeometric distribution. *N* is the number of all genes in the network, and *n* and *M* are the number of genes in the two gene sets, respectively. The cumulative function is used to calculate the overlap significant *p*-value of the two gene sets. A *p*-value < 0.05 indicates that the overlap of the two gene sets is significant.

##### Network distance

The average shortest distance *d*_*AB*_ of gene sets *A* and *B* on the network is calculated as follows:

7 $$\left\langle {d_{AB}} \right\rangle = \frac{1}{{\left| A \right| + \left| B \right|}}\left( {\mathop {\sum}\limits_{a \in A} {\mathop{\min}\limits_{b \in B}d(a,b) + \mathop {\sum}\limits_{b \in B} {\mathop{\min}\limits_{a \in A}} } d(a,b)} \right),$$

where *d(a, b)* represents the shortest distance between the two genes *a* and *b* in the network. When *a* and *b* are the same, *d(a, b)* = 0. |*A*| and |*B*| are the sizes of gene sets *A* and *B*, respectively.

The network distance, *s*_*AB*_, can describe the positional relationship of two sets of nodes in the network. The smaller the *s*_*AB*_, the closer the distance of the gene set in the network.

8 $$s_{AB} = \left\langle {d_{AB}} \right\rangle - \frac{{\left\langle {d_{AA}} \right\rangle + \left\langle {d_{BB}} \right\rangle }}{2}.$$

We found that the core genes of the four omics aspects were independent of each other, had little overlap, and were far apart in the network, while the peripheral genes of the four omics aspects were mixed with each other, overlapped more, and were close in the network. In the end, we modelled the observed results as a button-like structure, describing the omnigenic neighbourhood of cancer (Fig. 2i).

#### Biological characteristics of multi-omics periphery and core

##### Verification of biological data sets

We used ten biological datasets (Table 3, details in Supplementary Table 7) to show the enrichment performance of cancer in different biological profiles based on the multi-omics neighbourhood. These ten datasets included essential genes that play a decisive role in human life, disease-related pathogenic genes from OMIM, GWAS^78^, and ClinVar^79^, transcription factors from TF^80^, drug targets^37^, viral hosts, kinases, promoters^81^ and cancer genes from CGC^82^ (see table). These ten datasets reflect a wide range of indicators to measure the importance and biological significance of genes.

We used an *excessive overlap*^10^ to measure whether there was significant overlap between two gene sets. In a network with *N* genes, for gene sets *A* and *B*, excess overlap is defined as:

9 $$excess\,overlap = \frac{{\left| {\frac{{A \cap B}}{B}} \right|}}{{\left| {\frac{{A \cap N}}{N}} \right|}}.$$

The gene set *A* represents one of the ten biological datasets, and *B* represents the core region or the peripheral region in the multi-omics neighbourhood of cancer. When excess overlap >1, it means there is greater overlap than expected randomly, otherwise not.

##### Functional enrichment analysis

We used the over-representation analysis (ORA) method in the online ConsensusPathDB website (http://consensuspathdb.org/) to analyse the functional enrichment of the multi-omics periphery and core in pathways.

##### eQTL regulatory relationship

We used the eQTL data of cancer provided in the PancanQTL database^64^, including significant *cis-*eQTL and *trans-*eQTL regulatory effects (*p*-value < 0.01, estimated by Hardy–Weinberg R package^83^). eQTL provides pair relations set *P*, each relation $\left( {s,g} \right) \in P$ describes the significant regulatory influence of a single nucleotide polymorphisms (SNP) site *s* on Transcriptome expression of gene *g*, among which the set of sites is called *esite* and the set of significantly regulated gene is called *egene*. Five representative cancers were selected in our tests, namely BLCA, COAD, HNSC, READ and UCEC (the *CLines* of these cancers in the four omics aspects all conform to specific patterns).

First, we mapped the SNP sites to genes according to their genome positions. If a SNP *s* in *esite* is located in a gene *g*, denote as ${\mathop{\rm I}\nolimits} \left( {s,g} \right) = 1$ otherwise 0. The number $\left| {\left\{ {g\left| {{\mathop{\rm I}\nolimits} \left( {s,q} \right) = 1,\left( {s,g} \right) \in P,q \in X,g \in Y} \right.} \right\}} \right|$ is used to quantify the regulated amount of gene set Y being affected by gene set X. When there is a SNP site *s* located in the gene *q* of set X ${\mathop{\rm I}\nolimits} \left( {s,q} \right) = 1$, and site *s* has significant regulatory influence on a gene g of set Y $\left( {s,g} \right) \in P$, the regulated amount increases by one. Then we observed the regulated amount of core by peripheral gene sets. For the statistical significance, we randomly selected 1000 gene sets as counterparts of periphery to calculate the z-score (Fig. 3c). We designed three random strategies to obtain a random gene set: (1) Randomly select set with the same size of the periphery; (2) Randomly select connected component in the network with the same size of the periphery; (3) Randomly select gene sets that are consistent with the degree sequence and size of the periphery. Each group has 1000 random experiments. Furthermore, we quantified the regulated amount of omics-specific core genes in eQTL, for each omics aspect, we calculated the excess overlap between the core and *egene*, and found that the somatic mutation core is mostly regulated in eQTL (Fig. 3d). Finally, the number $\left| {\left\{ {q\left| {{\mathop{\rm I}\nolimits} \left( {s,q} \right) = 1,\left( {s,g} \right) \in P,q \in X,g \in Y} \right.} \right\}} \right|$ is used to quantify the regulatory amount of gene set X having over gene set Y. We calculated the regulatory amount proportion of peripheral genes having over the core genes in the four omics aspects, it is found that the variations that regulate core mainly occur in copy number variant periphery (Fig. 3e).

#### Disease similarity analysis

In order to explore the relative contribution of omnigenic neighbourhood to the commonalities between cancers and also explain cancer–cancer relationships, the known and convincing cancer similarity data are from DO similarity^65^, symptom similarity^66^ and comorbidity RR^67^ are used for for verification.

DO similarity: DO similarity data are calculated by the R package DOSim^65^. DOSim provides a simple and direct method to study disease similarity. It calculates the similarity of diseases by using semantic similarity measures in Disease Ontology (DO) to deepen our understanding of the complex pathogenesis of diseases and the relationship between different diseases.

Symptom similarity: Symptom similarity data comes from the Human Symptoms Disease Network (HSDN) based on symptoms^66^. The weight of the link between two diseases quantifies the similarity of their symptoms. Symptoms are crucial in the clinical diagnosis and treatment of diseases. The HSDN is constructed using a large biomedical literature database to study the relationship between the clinical manifestations of the disease and its potential molecular interactions.

Comorbidity RR: We used comorbidity data between genetically related diseases^67^. The degree of comorbidity is quantified by relative risk, which is calculated based on the Medicare database of approximately 13 million patients. Studying the systemic correlation between network interactions and comorbidity can provide opportunities for understanding disease mechanisms and developing treatment methods.

We first mapped 15 cancers to these three datasets, and obtained 55 pairs of DO similarity values among 11 cancers, 54 pairs of symptom similarity values among 11 cancers and 29 pairs of comorbidities among 10 cancers. Then, we used the mapping data to verify the results of cancer–cancer relationship based on omnigenic neighbourhood.

We used two methods to predict cancer similarity.

1. Disease similarity *simAB*. The similarity *simAB* of two cancers A and B is:
  10 $$simAB_{} = 1 - \frac{{\left\langle {d_{AB}} \right\rangle }}{{\left\langle d \right\rangle _{\max }}},$$
  where $\left\langle {d_{AB}} \right\rangle$ is the average shortest distance between cancers A and B in the human interactome. The calculation method is shown in formula (7). $\left\langle d \right\rangle _{\max }$ represents the largest average shortest distance between all cancer pairs. The range of *simAB* is between 0 and 1. The larger the *simAB*, the higher the cancer similarity.
2. Jaccard coefficient: in measuring the similarity between two cancers, the Jaccard coefficient was used to calculate the ratio of overlapping genes to all genes in the gene set of two cancers, as shown in formula (4). The larger the Jaccard coefficient value, the higher the cancer similarity.

## Supplementary information

Supplementary Material

Supplementary Table 1

## Data Availability

Supplementary data are available online at https://github.com/wangbingbo2019/ENCORE-Cancer including: Supplementary Tables 3–8, 11–12.
