# Supplementary material for: Multi-omics peripheral and core regions of cancer
Source: NPJ Syst Biol Appl. 2022 Nov 29;8:47. doi: 10.1038/s41540-022-00258-1 (PMC9707100; doi:10.1038/s41540-022-00258-1)
Supplement: Supplementary file 1 — Supplementary Material [file 41540_2022_258_MOESM1_ESM.pdf]

Supplementary materials

1. Multi-omics data preprocessing of cancer

We used cancer multi-omics data from UCSC Xena[1] to sort out the publicly available sample datasets of 15 cancers from TCGA[2], containing Transcriptome differential expression (Transcriptome), DNA differential methylation (Methylation), Somatic mutation and Copy number variation (CNV) data (Supplementary Table 1). We preprocessed the original multi-omics datasets of the 15 cancers, and some of the original data was preprocessed using the limma R package. The gene variation value is expressed as the absolute value of *logFC* in Transcriptome and Methylation omics aspects, and as the frequency value in somatic mutation and CNV omics aspects. For transcriptome, we used an R package edge[3] to perform differential expression analysis to obtain the *p-value* and *logFC* value of related differentially expressed genes. *LogFC* refers to the logarithm of Fold Change (FC), which is the logarithm of the multiple of the average expression level of a certain gene in disease samples compared with it in normal samples. For methylation, we used the R package limma[4] to obtain the *p-value* and *logFC* value of the relevant differential methylation level. In the subsequent experiments, the differential expression and differential methylation genes (adjust *p value* $\leq$ 0.05) are ranked by the absolute value of *logFC*. For somatic mutation and CNV, we calculated the frequency value of variation in samples. That is, the ratio of the samples with variations in the whole population. The CNV is estimated to be -2, -1, 0, 1, 2, representing the homozygous deletion, single copy deletion, diploid normal copy, low-level copy number amplification, or high-level copy number amplification.

We mapped variation values of genes to the corresponding node perturbation degrees in the network. According to the absolute value of *logFC* and frequency value sorted from high to low, we got the ranking list of nodes based on the perturbation degrees. For each omics aspect, we believe that the top 25% nodes are suitable for subsequent analysis (a wide omnigenic range about 4,000 in the network). Therefore, the details of data preparation results for 15 cancers in four omics aspects of are shown

in Supplementary Table 2 (details in Supplementary Table 3).

Supplementary Table 1. The number of samples of multi-omics data for 15 cancers

cancer	name	Transcriptome	Methylation	Somatic mutation	CNV
**BLCA**	Bladder Urothelial Carcinoma	426	434	411	408
**BRCA**	Breast Invasive Carcinoma	1218	888	791	1080
**CHOL**	Cholangiocarcinoma	45	45	36	36
**COAD**	Colon Adenocarcinoma	329	337	290	451
**ESCA**	Esophageal Carcinoma	198	202	184	184
**HNSC**	Head and Neck Squamous Cell Carcinoma	566	580	507	522
**KIRC**	Kidney Renal Clear Cell Carcinoma	606	480	368	528
**KIRP**	Kidney Papillary Cell Carcinoma	323	321	281	288
**LIHC**	Liver Hepatocellular Carcinoma	423	429	363	370
**LUAD**	Lung Adenocarcinoma	495	492	543	516
**LUSC**	Lung Squamous Cell Carcinoma	553	415	480	504
**PRAD**	Prostate Adenocarcinoma	550	549	495	492
**READ**	Rectum Adenocarcinoma	105	106	90	165
**THCA**	Thyroid Carcinoma	572	571	492	499
**UCEC**	Uterine Corpus Endometrial Carcinoma	201	478	447	539

Supplementary Table 2. The number and the perturbation degree range of the top 25% genes.

<table>
<tr><th>Cancer</th><th colspan="2">Transcriptome</th><th colspan="2">Methylation</th><th colspan="2">Somatic mutation</th><th colspan="2">CNV</th></tr>
<tr><th></th><th>#gene</th><th>Range of |logFC|</th><th>#gene</th><th>Range of |logFC|</th><th>#gene</th><th>Range of Frequence</th><th>#gene</th><th>Range of Frequence</th></tr>
<tr><td>**BLCA**</td><td>4030</td><td>7.73~0.59</td><td>4030</td><td>0.459~0.05</td><td>4665</td><td>0.508~0.01</td><td>4188</td><td>0.73~0.534</td></tr>
<tr><td>**BRCA**</td><td>2433</td><td>8.063~1</td><td>4036</td><td>0.411~0.04</td><td>5098</td><td>0.317~0.00</td><td>4041</td><td>0.779~0.49</td></tr>
<tr><td>**CHOL**</td><td>4030</td><td>10.64~1.12</td><td>1955</td><td>0.473~0.00</td><td>1581</td><td>0.333~0.02</td><td>4493</td><td>0.861~0.47</td></tr>
<tr><td>**COAD**</td><td>4035</td><td>9.58~0.71</td><td>4035</td><td>0.531~0.04</td><td>4329</td><td>0.719~0.02</td><td>4091</td><td>0.727~0.36</td></tr>
<tr><td>**ESCA**</td><td>3850</td><td>6.33~0.33</td><td>3326</td><td>0.376~0.00</td><td>4956</td><td>0.87~0.011</td><td>4125</td><td>0.815~0.60</td></tr>
<tr><td>**HNSC**</td><td>4031</td><td>7.42~0.55</td><td>4031</td><td>0.429~0.04</td><td>4329</td><td>0.713~0.01</td><td>4080</td><td>0.761~0.44</td></tr>
<tr><td>**KIRC**</td><td>4030</td><td>8.251~0.7</td><td>4030</td><td>0.369~0.03</td><td>5986</td><td>0.535~0.00</td><td>4056</td><td>0.896~0.24</td></tr>
<tr><td>**KIRP**</td><td>4034</td><td>7.35~0.63</td><td>4034</td><td>0.407~0.02</td><td>5766</td><td>0.143~0.00</td><td>4184</td><td>0.701~0.36</td></tr>
<tr><td>**LIHC**</td><td>4031</td><td>9.26~0.59</td><td>4031</td><td>0.422~0.05</td><td>5330</td><td>0.332~0.02</td><td>4047</td><td>0.77~0.43</td></tr>
<tr><td>**LUAD**</td><td>4031</td><td>9.016~0.69</td><td>4031</td><td>0.346~0.03</td><td>4354</td><td>0.521~0.01</td><td>4221</td><td>0.758~0.56</td></tr>
<tr><td>**LUSC**</td><td>4036</td><td>9.9~0.952</td><td>4036</td><td>0.418~0.04</td><td>5063</td><td>0.792~0.01</td><td>4045</td><td>0.912~0.65</td></tr>
<tr><td>**PRAD**</td><td>4031</td><td>6.8~0.44</td><td>4031</td><td>0.413~0.03</td><td>4492</td><td>0.142~0.00</td><td>4153</td><td>0.622~0.14</td></tr>
<tr><td>**READ**</td><td>4033</td><td>10.1~0.734</td><td>4033</td><td>0.587~0.01</td><td>7734</td><td>0.852~0.01</td><td>4121</td><td>0.897~0.44</td></tr>
<tr><td>**THCA**</td><td>4031</td><td>8.645~0.41</td><td>4031</td><td>0.374~0.00</td><td>4035</td><td>0.589~0.00</td><td>4365</td><td>0.186~0.03</td></tr>
<tr><td>**UCEC**</td><td>4039</td><td>7.625~0.82</td><td>4039</td><td>0.522~0.05</td><td>4383</td><td>0.649~0.03</td><td>4077</td><td>0.481~0.27</td></tr>
</table>

2. Cutoff optimization

When cutoff *fi* is about to reach the maximum perturbation degree, the number of perturbed genes meeting the cutoff is small, and the LCC formed by these genes is mostly in the single digits, usually manifested as a triplet motif. To indicate the statistical significance, we randomly selected the same small number of genes as a random counterpart in the network. But it is difficult for the randomized few genes to occupy an edge in the network. This leads to connectivity significance LCC z-score value is very susceptible to the existing motif, and presents an enormous value in misleading. We used the following strategies to optimize the maximum cutoff to improve the reliability of z-score:

1) Initialize the maximum cutoff *maxf*;
2) Calculate the size of LCC formed by gene sets that meet the cutoff conditions under different cutoffs;
3) If the sizes of LCCs formed at two consecutive *fi-1*, *fi* are less than or equal to 6, then set *maxf=fi* and use the formula (2) in main text to update the cutoff set *fl*.

3. The rational analysis of selecting the top 1,500 genes with variation as the omnigenic neighborhood.

To further analyze omnigenic pattern of cancer, for each omics aspect, we used *UCurve* to identify the peripheral and core regions. We took the LCC with local maximum z-score in high perturbation part of *UCurve* as the core. Since connectivity patterns are different, we selected the LCC at second peak in Transcriptome, the LCC at the single peak in Somatic mutation, and the LCCs with local maximum z-scores in the high perturbation parts of their *UCurve* in Methylation and CNV. The core region is highly perturbed and significantly connected subgraph in the network. We selected the LCC of the top 1,500 genes as the omnigenic neighbourhood, which contains the core and peripheral genes. Since core has been identified by *UCurve*, the remaining genes are peripheral after removing the core (details of genes in multi-omics periphery and core in Supplementary Table 6).

Compared with core genes, peripheral genes have a smaller impact on disease risk. The important thing is that the number of peripheral genes is very large, reaching few thousands, and the sum of their weak effects may far exceed the genetic contribution of core genes to diseases. Boyle et al. hypothesized that the regulatory network is highly interconnected, so that any expressed peripheral genes may affect the core genes through regulation[5]. Based on the above hypothesis, when selecting the omnigenic neighborhood, we should choose a gene set that has good connectivity, has a non-zero impact on the disease, and satisfies the large number of peripheral genes. In order to facilitate subsequent research and reduce computational complexity, we need to select a gene set that is relatively suitable in size and can reflect the connectivity of a wide range of genes with weak effects. Therefore, when selecting the omnigenic neighborhood, for different omics aspects, we selected the largest connected subgraph formed by the top 1,500 genes in the network. The neighborhood is relatively large in size, has an association with cancer, and can reflect the domain connectivity of weakly-perturbed neighborhoods.

First, we verified that the top 1,500 genes form a subnetwork with significant connectivity and including amounts of weakly-perturbed genes. We conducted a

statistical analysis of connectivity in the network for four omics aspects. 1) In transcriptome, the non-highly perturbed part (below the 3/4 cutoff) corresponds to the LCC $z-score=1.14\pm1.65$. The mean z-score corresponding to the top 1,500 genes of the 15 cancer is 0.88, which is consistent with the z-scores of the non-highly part. 2) In methylation, the non-highly perturbed part (below the 3/4 cutoff) corresponds to the LCC $z-score=-5.97\pm1.57$, showing that the genes are fragmented in the network. The average z-score corresponding to the top 1,500 genes of the 15 cancer is -5.67, which is consistent with the z-score level of the non-highly perturbed parts. 3) In somatic mutation, the non-highly perturbed part (below the 3/4 cutoff) corresponds to the LCC $z-score=5.4\pm2.43$ The average z-score corresponding to the top 1,500 genes of the 15 cancer is 4.02, which is consistent with the z-score level of the non-highly perturbed part. 4) In CNV, the non-highly perturbed part (below the 3/4 cutoff) corresponds to the connectivity significance LCC $z-score=1.85\pm0.49$. The mean z-score corresponding to the top 1,500 genes of the 15 cancer is 1.94, which is consistent with the z-score level of the non-highly perturbed part. The results show that among different omics aspects, the gene set with the top 1,500 variation values can reflect the connectivity of the gene set corresponding to the non-highly perturbed part.

Then, in order to calculate the degree to which the top 1,500 gene sets were perturbed by disease, we conducted a statistical analysis on the variation values of the top 1500 genes in each omics aspect of 15 cancers. The corresponding variation values are shown in Table 1. Among them, in Transcriptome, the maximum and minimum values of the variation log(FC) are 8.45 ± 1.23 and 1.4 ± 0.4 respectively. In Methylation, the maximum and minimum values of variation log(FC) are 0.44 ± 0.07 and 0.1 ± 0.33 respectively. In Somatic mutation, the maximum and minimum mutation frequencies are 0.53 ± 0.23 and 0.22 ± 0.01 respectively. And in CNV, the maximum and minimum variation frequencies are 0.73 ± 0.18 and 0.53 ± 0.19 respectively. The results show that the top 1,500 gene sets with different omics aspects of cancer include high-disturbance core genes and low-disturbance peripheral genes, which are in line with the hypothesis of core and peripheral genes in omnigenic

neighborhood.

Finally, in order to analyze the correlation between cancers and the top 1,500 genes in different omics aspects, we calculated the enrichment of them in five datasets of GWAS, OMIM, CGC, ClinVar and Drug Target (Supplementary Figure 6). The five datasets include disease-related genes, oncogenes and drug targets. We sorted the genes according to their variation value, and calculated the excess overlap between the set of top x (x = 100, 200, ..., 4,000) genes and the biological dataset. When the excess overlap > 1, it means that there is a significant overlap between the gene set and the biological dataset. The vertical line *L* is used to mark the excess overlap between the set of top 1,500 genes and the biological dataset (Supplementary Figure 6). The results show that in four omics aspects, based on five biological datasets, excess overlap has different variation trends with the increase of gene number. 1) **Transcriptome**: The results of the GWAS dataset show an upward trend and then a downward trend. The vertical line *L* corresponds to the maximum value of excess overlap (mean value 1.26). The results of the CGC dataset show an upward trend, and the vertical line *L* corresponds to the rising platform region (average excess overlap = 1.23). The results of OMIM, ClinVar and Drug Target datasets show a downward trend, and the vertical line *L* corresponds to an excess overlap> 1 (mean values are 1.38, 1.16 and 1.41, respectively). 2) **Methylation**: The results of the GWAS dataset show a downward trend, and the excess overlap >1 (average excess overlap = 1.14) corresponding to the vertical line *L*. The results of the OMIM, CGC and ClinVar datasets show an upward trend. Among them, the vertical line *L* corresponding to OMIM is located at the rising inflection point (average excess overlap = 1.12), and the position corresponding to the vertical line *L* of ClinVar rises to the excess overlap greater than 1 (average excess overlap = 1.02). The results of the Drug Target dataset show a trend of rising first and then falling, and the vertical line *L* corresponds to a higher excess overlap (average excess overlap = 1.2). 3) **Somatic mutation**: The results of the four datasets of GWAS, OMIM, CGC and ClinVar show a downward trend, and the excess overlap corresponding to the vertical line L is all greater than 1 (the average value is 1.43, 1.64, 2.45, 1.54, respectively). The results of the Drug Target dataset show an

upward trend and the position corresponding to the vertical line L is the rising plateau area of the curve (excess overlap mean 1.15). 4) **CNV**: The results of the GWAS dataset show a downward trend, and the excess overlap is greater than 1 corresponding to the vertical line *L* (average excess overlap = 1.07). The results of the ClinVar dataset show an upward trend, and the excess overlap corresponding to the vertical line *L* is greater than 1 (average excess overlap = 1.08). The results of OMIM, CGC and Drug Target datasets show a fluctuating trend, and the excess overlap corresponding to the vertical line *L* is all greater than 1 (the average value is1.02, 1.12, 1.04, respectively). The results show that for different cancers in the four omics aspects, the vast majority (95%) of the enrichment results based on the five biological datasets are significant, indicating that the sets of the top 1,500 genes are closely related to the occurrence and development of the disease. These genes may be potentially disease-related pathogenic genes, cancer genes and drug targets.

In summary, among different omics aspects, the top 1,500 gene sets with variation values are of appropriate size, well connectivity, and contain high and low disturbance genes, as well as characteristics closely related to disease. Therefore, we believe that it is reasonable to select the LCC of the disease neighborhood with the top 1,500 genes as the omnigenic neighborhood of cancer in each omics aspect.

4. The enrichment analysis result of GO and Reactome pathway

In order to study the biological characteristics of multi-omics periphery and core in cancers, functional enrichment analysis is usually performed, hoping to discover genes or biological pathways that play a key role in them. Biological pathways have become the first choice for the study of gene function. They can generalize hundreds of genes, proteins or other molecules into known pathways with different functions, reveal and understand the basic molecular mechanisms of biological processes embedded in the periphery and core, reduce the complexity of analysis and improve the explanatory power[6]. In the past decade, pathway enrichment analysis has proven to be a very useful tool for interpreting large-scale transcriptome and proteomics data[7].

Then we introduced the three widely used databases for functional enrichment analysis: 1) The GO (Gene Ontology) database records three GO items according to the function of genes: biological process, molecular function, and cellular component, which can be accessed through http://www.geneontology.org[8]. Through GO function enrichment analysis, it is possible to find out the GO terms that are statistically significantly enriched in the omnigenic neighborhood of cancer, thereby revealing the biological processes involved. 2) KEGG is a resource library of advanced functions and utilities for understanding biological systems, which can be used through the KEGG website http://www.kegg.jp/[7]. KEGG datasets include information about cancer metabolism, signal transduction, cell cycle and other pathways. One of the main goals of KEGG is to discover higher-level system functions of cells and organisms from information at the genomic and molecular level. Through KEGG function enrichment analysis, it is possible to find out the KEGG pathways that are statistically significantly enriched in the omnigenic neighborhood. 3) Reactome Knowledgebase (https://reactome.org) is an improved database that integrates various reactions and biological pathways in the human body compared to the KEGG database. The Reactome Knowledgebase provides molecular details of signal transduction, transport, DNA replication, metabolism and other cellular processes as an ordered network of molecular transformations in a single consistent data model[9].

Reactome functions both as an archive of biological processes and as a tool for discovering functional relationships in data.

To analyze the biological characteristics of omnigenic neighborhood, we used the over-representation analysis (ORA) method in the online ConsensusPathDB website (http://consensuspathdb.org/) to obtain the function of enrichment of MOPC of 15 cancers. The results of functional enrichment using GO[8], KEGG[7] and Reactome[9] are sorted out (Figure 3b,c, Supplementary Figure 10a-d), and the analysis of KEGG results is shown in the main text.

As shown in Supplementary Figure 10a-d, we performed GO term enrichment analysis of biological processes and Reactome pathway enrichment analysis. We collect 1244 GO terms and 836 Reactome pathways that are enriched in 15 cancers (hypergeometric p-value<0.01, Supplementary Table 8) and count the number of cancers involved in each term or pathway (Supplementary Figure 10a, b), respectively. The result show that 270 terms and 220 pathways are enriched in one cancer while 145 terms and 23 pathways are involved in 15 cancers. Among the terms and pathways enriched in 15 cancers, we find some known or evidence-supported terms and pathways associated with cancer. These GO terms including regulation of cell-matrix adhesion[10], regulation of RNA metabolic process[11], endocytosis[12], regulation of GTPase activity[13] and phosphorylation[14]. And these Reactome pathways including L1CAM interactions[15], Signal Transduction[16], Extracellular matrix organization[17], Cell-Cell communication[18] and Semaphorin interactions[19]. For the 10 GO terms and 10 Reactome pathways show in Supplementary Figure 10a, c, we visualized the enrichment significance (-log10(p-value), based on hypergeometric test) of these terms and pathways in 15 cancers (Supplementary Figure 10 b, d).

The results of the enrichment of the omnigenic neighborhood on the GO term and Reactome pathway are combined with the literature verification, fully demonstrating the biological processes and biological pathways covered in the button model. Interestingly, we found that some pathways are collectively enriched by different cancers, and some pathways are only enriched by a specific cancer. This result implies

that different cancers participate in the same pathway through shared peripherals, thereby influencing each other during the disease process, and exhibit the specific functions of different cancers by participating in specific pathways.

5. Comparison with polygenic module

We interpreted the multi-omics data of cancer according to omnigenic model. It is well known that cancer data has been analyzed based on polygenic model[20]. We compare omnigenic neighborhood with three representative polygenic modules: network-based DIAMOnD[21] modules, a group of cancer driver genes (indicated by Driver(1), doi:10.1038/nature12912) and another group of cancer driver genes (indicated by Driver(2), doi.org/10.1038/s41586-020-1969-6). As results, we present 1) the overlap between omnigenic neighbourhood and these three polygenic modules in Supplementary Figure 11; 2) KEGG pathway enrichment analysis of omnigenic neighbourhood in Supplementary Figure 12; 3) the cancer similarity described by polygenic modules in Supplementary Figure 13; 4) cancer similarity under removing one omics data in Supplementary Figure 14; and 5) enrichment analysis of drug targets in Supplementary Figure 15.

DIAMOnD modules. Network-based DIAMOnD, the most advanced algorithm, is used to analyze the connectivity patterns of Disease Associated Proteins (DAPs) and form a disease module with a large number of intermediate proteins, which are known as DIAMOnD proteins. We take multi-omics core as DAPs for a specific cancer, and use the tool DIAMOnD to generate internal interconnected module in the human interactome. For each test, usually the tool introduces 200 DIAMOnD proteins as intermediate proteins. Then, we obtain 15 DIAMOnD modules (average size is 398) as polygenic counterparts for comparison with omnigenic neighbourhood.

Driver(1). Michael S. L. et al.[22] explored cancer genes across 21 tumour types based on somatic point mutations in exome sequences from 4,742 human cancers and their matched normal-tissue samples. We download the list of the 21 tumor types studied, and the significantly mutated genes found by the MutSig suite in each tumor type. By taking the intersection of these 21 tumor types and our 15 cancers, we gathered a group of driver genes for 8 cancers as showed in Supplementary Table 9. These 8 sets of driver genes are considered as the second group polygenic counterparts.

Supplementary Table 9. Cancer driver genes of 15 cancers collected from work of Michael S. L. et al.

Cancers		Number of driver genes
BLCA	Bladder Cancer	34
BRCA	Breast Cancer	37
CHOL	Bile Duct Cancer	---
COAD	Colon Cancer	---
ESCA	Esophageal Cancer	---
HNSC	Head and Neck Cancer	35
KIRC	Kidney Clear Cell Carcinoma	21
KIRP	Kidney Papillary Cell Carcinoma	---
LIHC	Liver Cancer	---
LUAD	Lung Cancer	32
LUSC	Lung Squamous Cell Carcinoma	24
PRAD	Prostate Cancer	6
READ	Rectal Cancer	---
THCA	Thyroid Cancer	---
UCEC	Endometrioid Cancer	73

--- Corresponding cancer type is not found.

Driver(2). Pan-Cancer Analysis of Whole Genomes (PCAWG) Consortium of the International Cancer Genome Consortium (ICGC) and The Cancer Genome Atlas (TCGA) reported the integrative analysis of 2,658 whole-cancer genomes and their matching normal tissues across 38 tumour types[2]. They described that driver mutations in tumour evolution precede most somatic point mutations and affect several cancer-associated genes simultaneously. We obtained the datasets of driver mutations at https://dcc.icgc.org. By taking the intersection with our 15 cancers, we curated a group of driver genes for 13 cancers as showed in Supplementary Table 10. For each test, we introduce top 200 driver genes as polygenic modules. These 13 sets of driver genes are taken as the third group polygenic counterparts.

Supplementary Table 10. Cancer driver genes of 15 cancers collected from PCAWG.

Cancers		Number of mutations	Source
BLCA	Bladder Cancer	9217	https://dcc.icgc.org/projects/BLCA-US/mutations

BRCA	Breast Cancer	8021	https://dcc.icgc.org/projects/BRCA-US/mutations
CHOL	Bile Duct Cancer	---	---
COAD	Colon Cancer	11568	https://dcc.icgc.org/projects/COAD-US/mutations
ESCA	Esophageal Cancer	---	---
HNSC	Head and Neck Cancer	7568	https://dcc.icgc.org/projects/HNSC-US/mutations
KIRC	Kidney Clear Cell Carcinoma	3328	https://dcc.icgc.org/projects/KIRC-US/mutations
KIRP	Kidney Papillary Cell Carcinoma	3529	https://dcc.icgc.org/projects/KIRP-US/mutations
LIHC	Liver Cancer	4854	https://dcc.icgc.org/projects/LIHC-US/mutations
LUAD	Lung Cancer	9954	https://dcc.icgc.org/projects/LUAD-US/mutations
LUSC	Lung Squamous Cell Carcinoma	9578	https://dcc.icgc.org/projects/LUSC-US/mutations
PRAD	Prostate Cancer	3381	https://dcc.icgc.org/projects/PRAD-US/mutations
READ	Rectal Cancer	5248	https://dcc.icgc.org/projects/READ-US/mutations
THCA	Thyroid Cancer	1580	https://dcc.icgc.org/projects/THCA-US/mutations
UCEC	Endometrioid Cancer	15826	https://dcc.icgc.org/projects/UCEC-US/mutations

--- Corresponding cancer type is not found.

References

1. Goldman M et al. 2020 Visualizing and interpreting cancer genomics data via the Xena platform. Nat. Biotechnol. 38. (doi:10.1038/s41587-020-0546-8)

2. Tomczak K, Czerwinska P, Wiznerowicz M. 2015 The Cancer Genome Atlas (TCGA): An immeasurable source of knowledge. Contemp Oncol (Pozn) 19, A68–A77. (10.5114/wo.2014.47136)

3. Leek J, Monsen E, Dabney A, Storey J. 2006 EDGE: Extraction and Analysis of Differential Gene Expression. Bioinformatics 22, 507–508. (doi:10.1093/bioinformatics/btk005)

4. Ritchie M, Phipson B, Wu D, Hu Y, Law C, Shi W, Smyth G. 2015 LIMMA powers differential expression analyses for RNA-sequencing and microarray studies. Nucleic Acids Res. 43. (doi:10.1093/nar/gkv007)

5. Boyle E, Li Y, Pritchard J. 2017 An Expanded View of Complex Traits: From Polygenic to Omnigenic. Cell 169, 1177–1186. (doi:10.1016/j.cell.2017.05.038)

6. Ashburner M et al. 2000 Gene ontology: tool for the unification of biology. The Gene Ontology Consortium. Nat. Genet. 25, 25–29. (doi:10.1038/75556)

7. Kanehisa M, Goto S, Furumichi M, Tanabe M, Hirakawa M. 2010 KEGG for representation and analysis of molecular networks involving diseases and drugs. Nucleic Acids Res. 38, D355-60. (doi:10.1093/nar/gkp896)

8. Shi Z, Derow CK, Zhang B. 2010 Co-expression module analysis reveals biological processes, genomic gain, and regulatory mechanisms associated with breast cancer progression. BMC Syst. Biol. 4, 74. (doi:10.1186/1752-0509-4-74)

9. Jassal B et al. 2019 The reactome pathway knowledgebase. Nucleic Acids Res. 48. (doi:10.1093/nar/gkz1031)

10. Alpha KM, Xu W, Turner CE. 2020 Paxillin family of focal adhesion adaptor proteins and regulation of cancer cell invasion. Int. Rev. Cell Mol. Biol. 355, 1–52. (doi:10.1016/bs.ircmb.2020.05.003)

11. He L, Li H, Wu A, Peng Y, Shu G, Yin G. 2019 Functions of N6-methyladenosine and its role in cancer. Mol. Cancer 18, 176. (doi:10.1186/s12943-019-1109-9)

12. Schmid SL. 2017 Reciprocal regulation of signaling and endocytosis: Implications for the evolving cancer cell. J. Cell Biol. 216, 2623–2632. (doi:10.1083/jcb.201705017)

13. Haga RB, Ridley AJ. 2016 Rho GTPases: Regulation and roles in cancer cell biology. Small GTPases 7, 207–221. (doi:10.1080/21541248.2016.1232583)

14. Singh V, Ram M, Kumar R, Prasad R, Roy BK, Singh KK. 2017 Phosphorylation: Implications in Cancer. Protein J. 36, 1–6. (doi:10.1007/s10930-017-9696-z)

15. Giordano M et al. 2021 L1CAM promotes ovarian cancer stemness and tumor initiation via FGFR1/SRC/STAT3 signaling. J. Exp. Clin. Cancer Res. 40, 319. (doi:10.1186/s13046-021-02117-z)

16. Park JH, Pyun WY, Park HW. 2020 Cancer Metabolism: Phenotype, Signaling and Therapeutic Targets. Cells 9. (doi:10.3390/cells9102308)

17. Walker C, Mojares E, Del Río Hernández A. 2018 Role of Extracellular Matrix in Development and Cancer Progression. Int. J. Mol. Sci. 19. (doi:10.3390/ijms19103028)

18. Wortzel I, Dror S, Kenific CM, Lyden D. 2019 Exosome-Mediated Metastasis: Communication from a Distance. Dev. Cell 49, 347–360. (doi:10.1016/j.devcel.2019.04.011)

19. Gu C, Giraudo E. 2013 The role of semaphorins and their receptors in vascular development and cancer. Exp. Cell Res. 319, 1306–1316. (doi:10.1016/j.yexcr.2013.02.003)

20. Visscher PM, Yengo L, Cox NJ, Wray NR. 2021 Discovery and implications of polygenicity of common diseases. Science 373, 1468–1473. (doi:10.1126/science.abi8206)

21. Ghiassian S, Menche J, Barabasi A-L. 2015 A DIseAse MOdule Detection (DIAMOnD) Algorithm Derived from a Systematic Analysis of Connectivity Patterns of Disease Proteins in the Human Interactome. PLoS Comput. Biol. 11, e1004120. (doi:10.1371/journal.pcbi.1004120)

22. Lawrence MS et al. 2014 Discovery and saturation analysis of cancer genes across 21 tumour types. Nature 505, 495–501. (doi:10.1038/nature12912)

23. Campbell, P. J. et al. 2020 Pan-cancer analysis of whole genomes. Nature 578, 82–93. (doi:10.1038/s41586-020-1969-6)

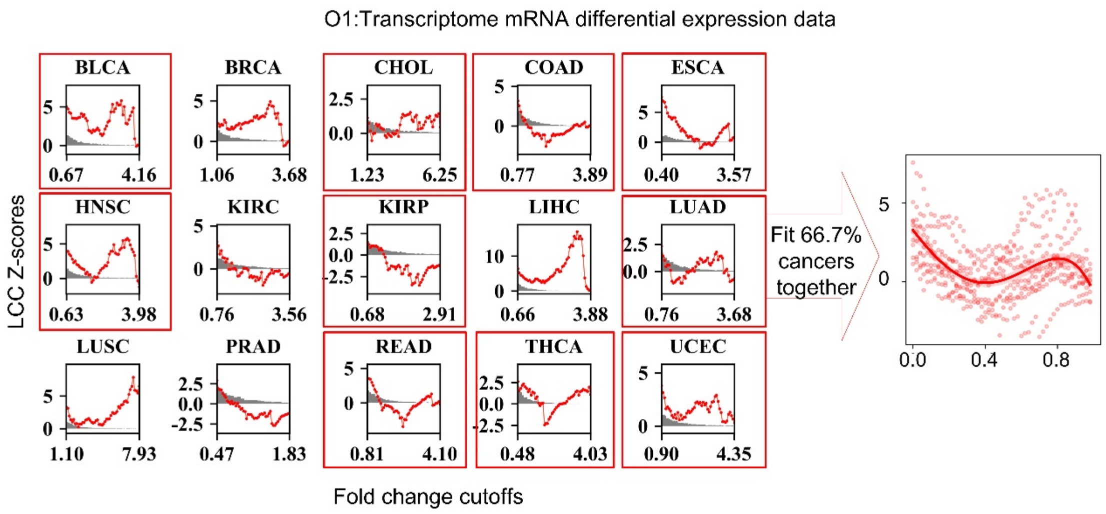

Supplementary Figure 1. Transcriptome omnigenic pattern. Each subplot gives a cancer connectivity line (*CLine*) (Figure 1a), which reflects the change in connectivity of the corresponding set of perturbed genes in the network with the change of fold change cutoff of transcriptome omics aspect. With the results obtained based on transcriptome differential expression, 66.7% *CLine* of cancers (marked with red boxes) meet the bimodal pattern ($d_{ratio}(L,M) > \alpha$ and $d_{ratio}(H,M) > \alpha$, $\alpha = 0.4$) and are universal. Normalize the abscissa of the *CLine* for 66.7% of cancers, and then use the least squares method to fit the points on them to obtain a Uniformed Curve (*UCurve*, right panel).

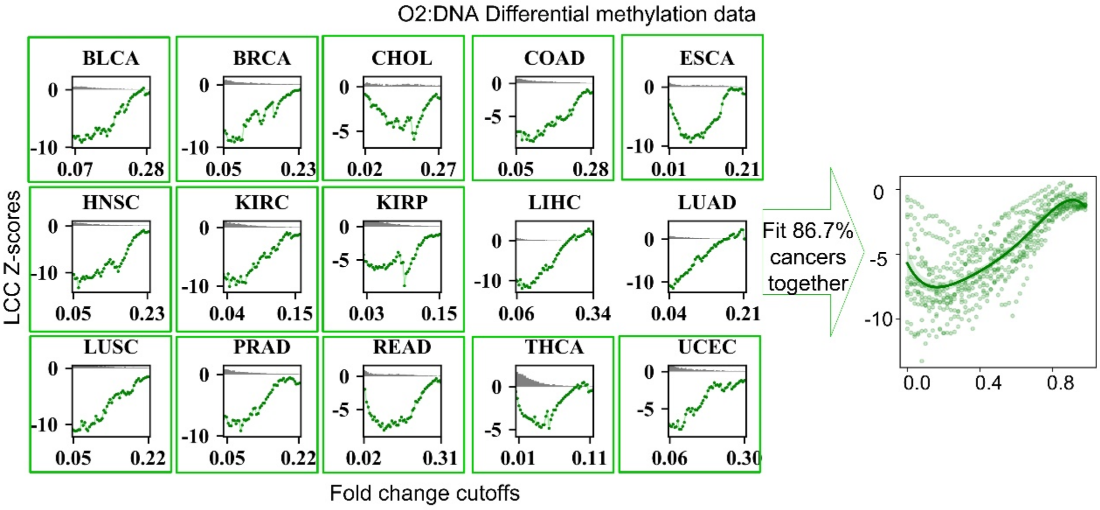

Supplementary Figure 2. Methylation omnigenic pattern. Each subplot gives a cancer connectivity line (*CLine*) (Figure 1a), which reflects the change in connectivity of the corresponding set of perturbed genes in the network with the change of fold change cutoff of methylation omics aspect. In the results obtained based on DNA differential methylation, 86.7% *CLine* of cancers (marked with green boxes) meet the fragment pattern (all z-score < 1.46) and are universal. Normalize the abscissa of the *CLine* for 86.7% of cancers, and then use the least squares method to fit the points on them to obtain a Uniformed Curve (*UCurve*, right panel).

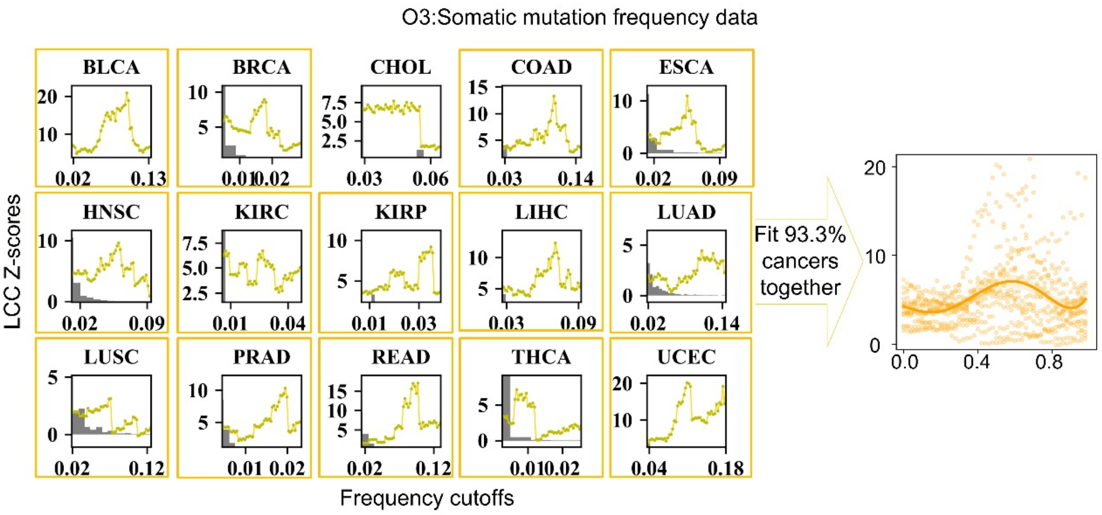

Supplementary Figure 3. Somatic mutation omnigenic pattern. Each subplot gives a cancer connectivity line (*CLine*) (Figure 1a), which reflects the change in connectivity of the corresponding set of perturbed genes in the network with the change of frequence cutoff of somatic mutation. The results obtained based on somatic mutation, 93.3% *CLine* of cancers (marked with yellow boxes) meet the unimodal pattern ($d_{ratio}(M,L) > \alpha$ and $d_{ratio}(M,H) > \alpha$, $\alpha = 0.4$) and are universal. Normalize the abscissa of the *CLine* for 93.3% of cancers, and then use the least squares method to fit the points on them to obtain a Uniformed Curve (*UCurve*, right panel).

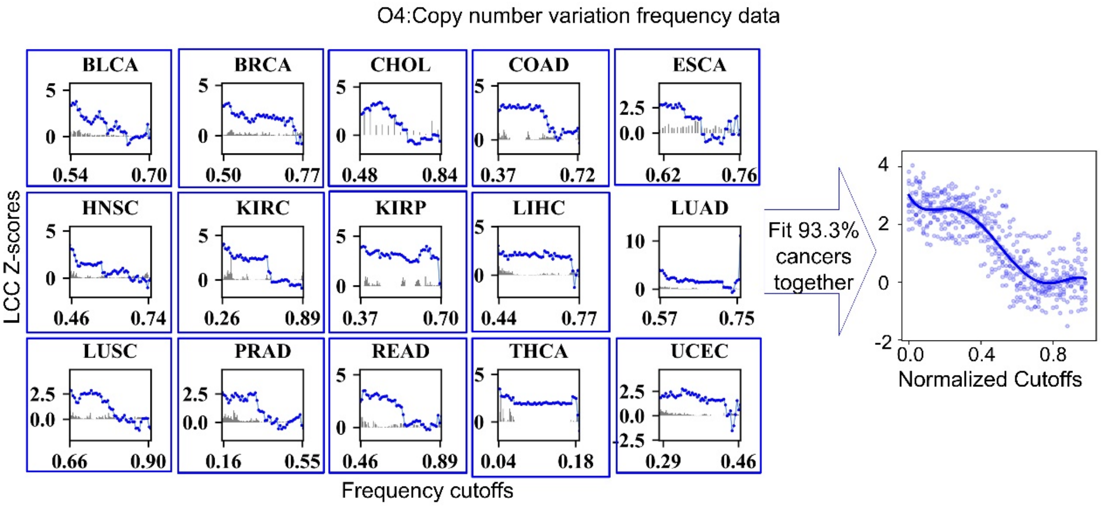

Supplementary Figure 4. CNV omnigenic pattern. Each subplot gives a cancer connectivity line (*CLine*) (Figure 1a), which reflects the change in connectivity of the corresponding set of perturbed genes in the network with the change of frequency cutoff of CNV. The results obtained based on CNV, 93.3% *CLine* of cancers (marked with blue boxes) meet the unimodal pattern ($d_{ratio}(L,H) > 2\alpha$, $\alpha = 0.4$) and are universal. Normalize the abscissa of the *CLine* for 93.3% of cancers, and then use the least squares method to fit the points on them to obtain a Uniformed Curve (*UCurve*, right panel).

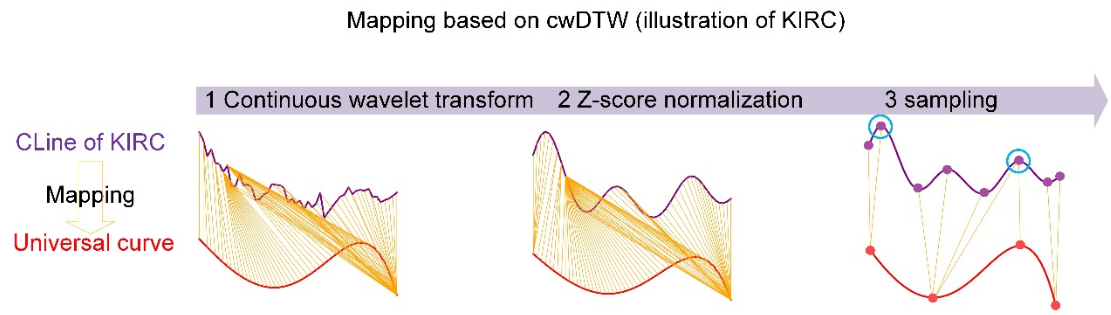

Supplementary Figure 5. Pattern mapping. Use the cwDTW method to map *CLine* to *UCurve*. Taking CLine obtained from transcriptome differential expression of KIRC as an example. KIRC contains 4030 genes in the low-disturbance region, and its connectivity z-score=3.12, but there is no significant connected subgraph formation in the high-disturbance region (maximum z-score = -0.19). Its *CLine* (purple line) does not meet the standard of the bimodal pattern ($d_{ratio}(H,M) = -0.09$). Map this *CLine* to the red *UCurve*. The main steps (method) include: perform continuous wavelet transformation on *CLine*, then normalize, sample the mapping result, and finally output the points corresponding to the double peaks of *UCurve* (highlighted with blue circles), mark the low-disturbance peripheral regions and high-disturbance core regions of KIRC.

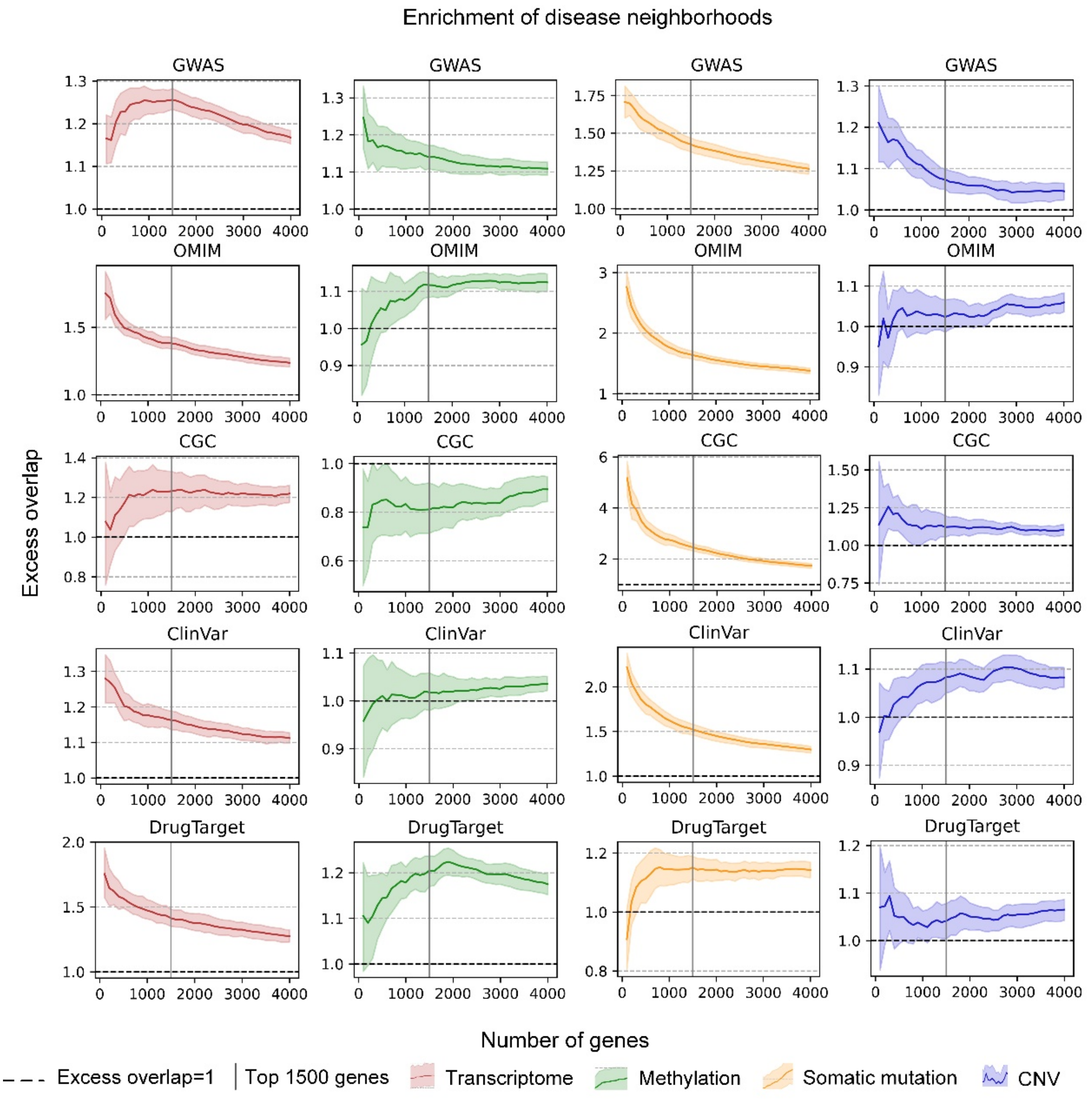

Supplementary Figure 6. Enrichment analysis of disease-perturbed genes. Use GWAS, OMIM, CGC, ClinVar, Drug Target datasets to perform enrichment analysis on the disease neighborhood of a given size. According to the perturbed strength ranking, select the top gene in the ranking list as disease neighborhood. The abscissa is the size of the selected disease neighborhood, and the ordinate is the degree of enrichment between the disease neighborhood and the corresponding dataset (excess overlap, see method). The horizontal dashed line marks the enrichment significance cutoff excess overlap = 1. The four omics aspects of transcriptome, methylation, somatic mutation, and CNV are represented by red, green, yellow, and blue respectively. The solid colored line represents the mean, and the shaded area is the 95% confidence interval. Enrichment of the disease neighborhood composed of top 1500 genes is marked with a solid vertical line.

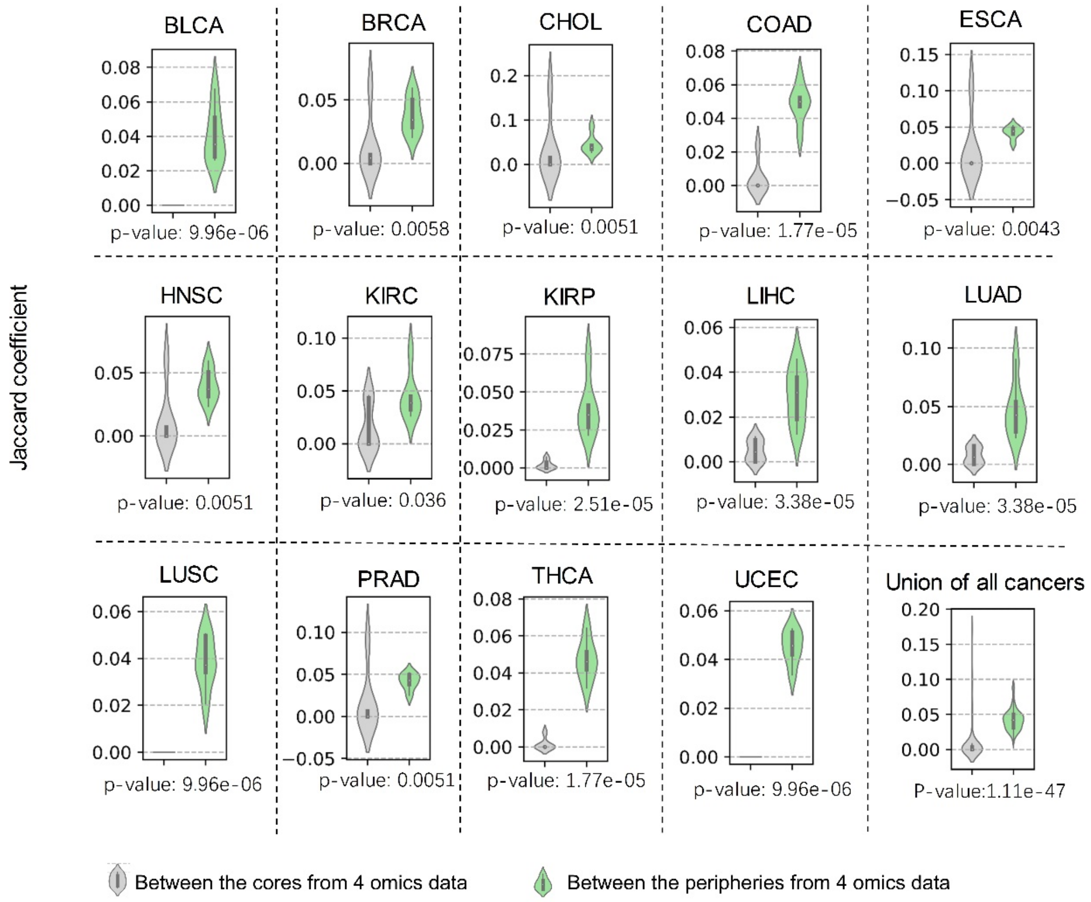

Supplementary Figure 7. Use gray and green violins to show the Jaccard coefficient between the core genes and peripheral genes of 15 cancers. The Mann-Whitney U Test p-value was used to detect the difference between the two sets of data. The error bars indicate the 95% confidence intervals.

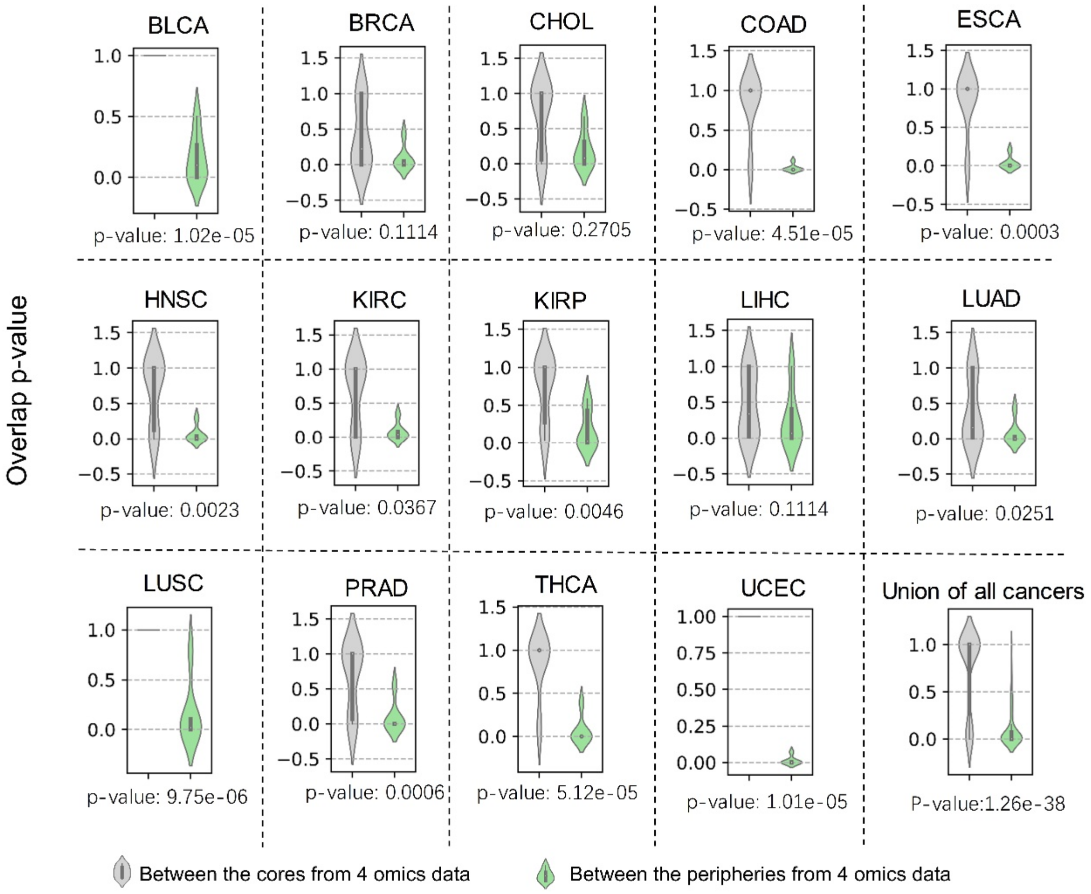

Supplementary Figure 8. Use gray and green violins to show the overlap p-value (hypergeometric test) between the core genes and peripheral genes of 15 cancers. The Mann-Whitney U Test p-value was used to detect the difference between the two sets of data. The error bars indicate the 95% confidence intervals.

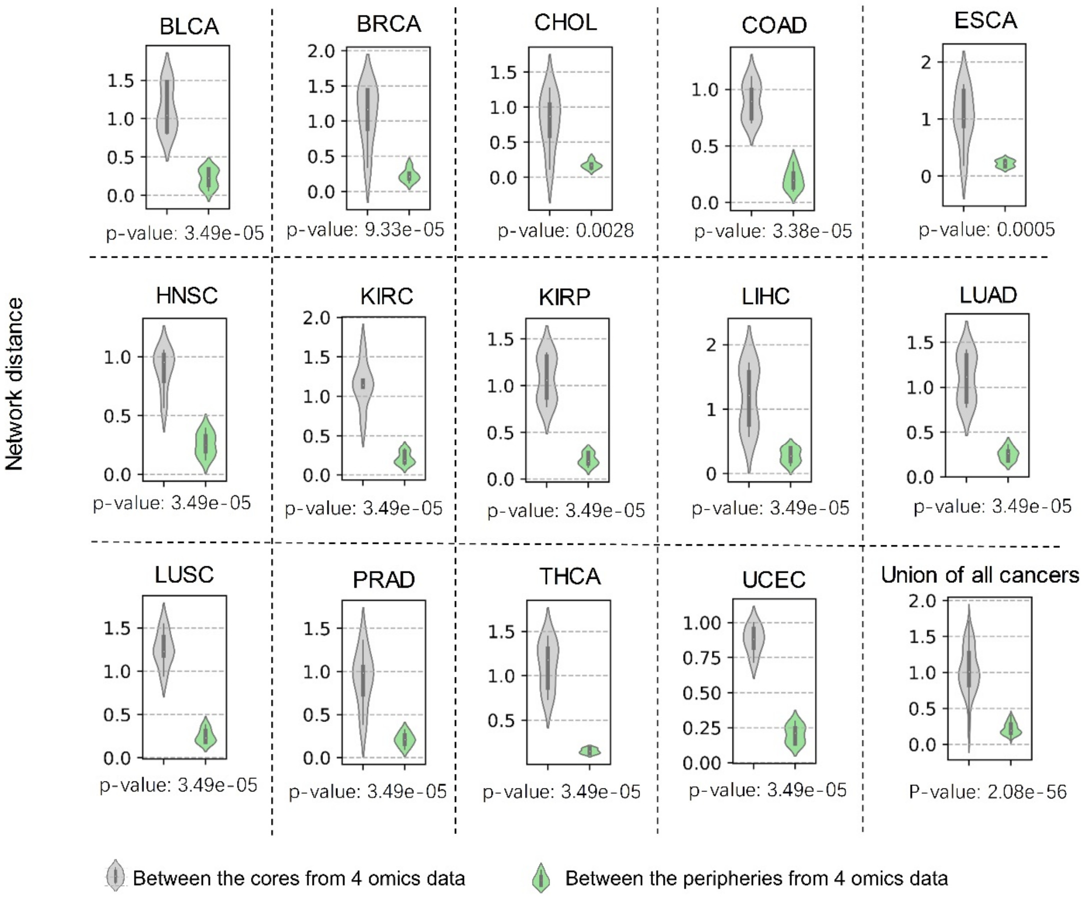

Supplementary Figure 9. Use gray and green violins to show the distance between the core genes and peripheral genes of 15 cancers. The Mann-Whitney U Test p-value was used to detect the difference between the two sets of data. The error bars indicate the 95% confidence intervals.

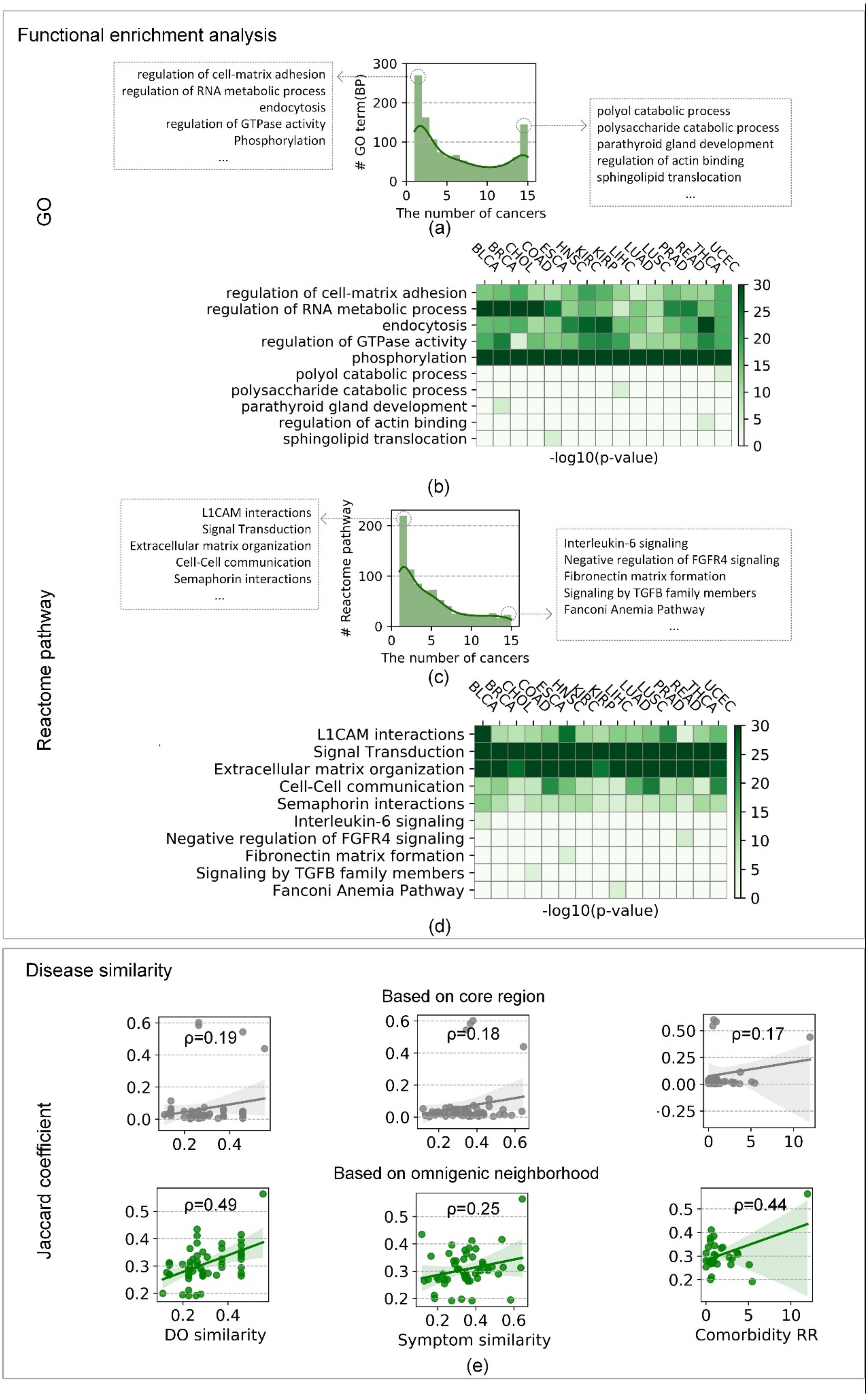

Supplementary Figure 10. (a-d) Functional enrichment analysis. (a, c) The distribution

of the number of cancers enriched by GO term(BP) and Reactome pathway. The left and right dotted boxes in the figure are examples of terms or pathways enriched in one cancers and 15 cancers respectively. (b, d) Focus on the GO terms in (a) and Reactome pathway in (c), show the enrichment result of these terms or pathways with 15 cancers. (e) **Omnigenic neighborhood portrays cancer similarity.** Use Jaccard coefficient to calculate the relationship between cancers (method), where the gray image represents the result of cancer similarity analysis based on core genes, and the green image represents the result of cancer similarity analysis based on omnigenic neighborhood. The points in the figure represent the results of cancer similarity, which is verified by comparison with DO similarity, symptom similarity, and comorbidity RR. The fitted line is the Pearson correlation coefficient between the predicted and known similarity of cancer. The shading indicates the 95% confidence interval. In the three similarity verification experiments, based on omnigenic neighborhood, the Pearson correlation coefficient increased by 2.58, 1.39 and 2.59 fold, respectively.

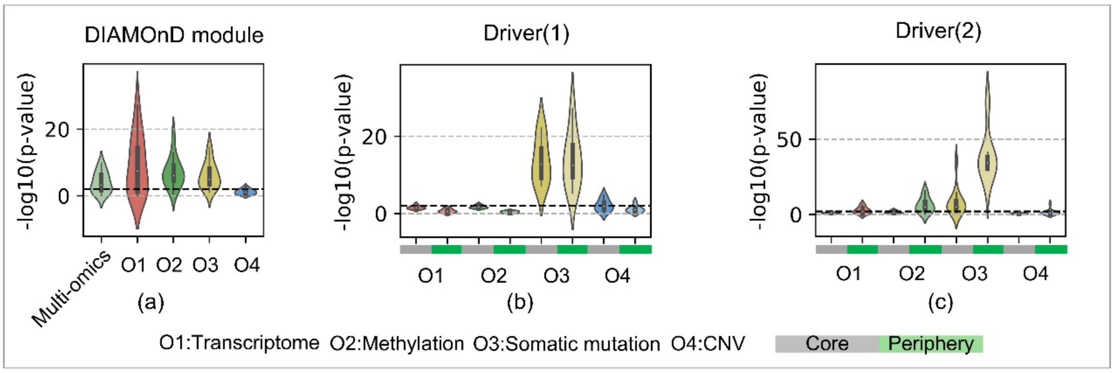

Supplementary Figure 11. Overlap between omnigenic neighbourhood and polygenic module. Three representative modules DIAMOnd, Driver(1) and Driver(2) are curated as counterpart polygenic modules. Four omics aspects are distinguished by O1, O2, O3, and O4, and the core and periphery are marked by grey and green grids respectively. The black dash lines highlight the threshold (p-value=0.05, hypergeometric test) of significance. (a) The violin charts show overlaps between omnigenic periphery and induced intermediate DIAMOnd genes. In 15 cancers, the average significance level (-log10(P-value)) reach 4.03, 9.08, 6.91, 6.15 and 1.19 respectively. (b) The violin charts show overlap between omnigenic neighbourhood and a group of sophisticated cancer driver genes (indicated by Driver(1)). Only the core and periphery of O3 (Somatic mutation) have significant overlap (-log10(P-value): 13.67 and 13.8) with Driver(1). (c) The violin charts show overlap between omnigenic neighbourhood and another group of sophisticated cancer driver genes (indicated by Driver(2)). The periphery from O2 (Methylation), the core and periphery of O3 (Somatic mutation) have significant overlap (-log10(P-value): 6.23, 8.17 and 39.2) with Driver(2). The error bars indicate the 95% confidence intervals.

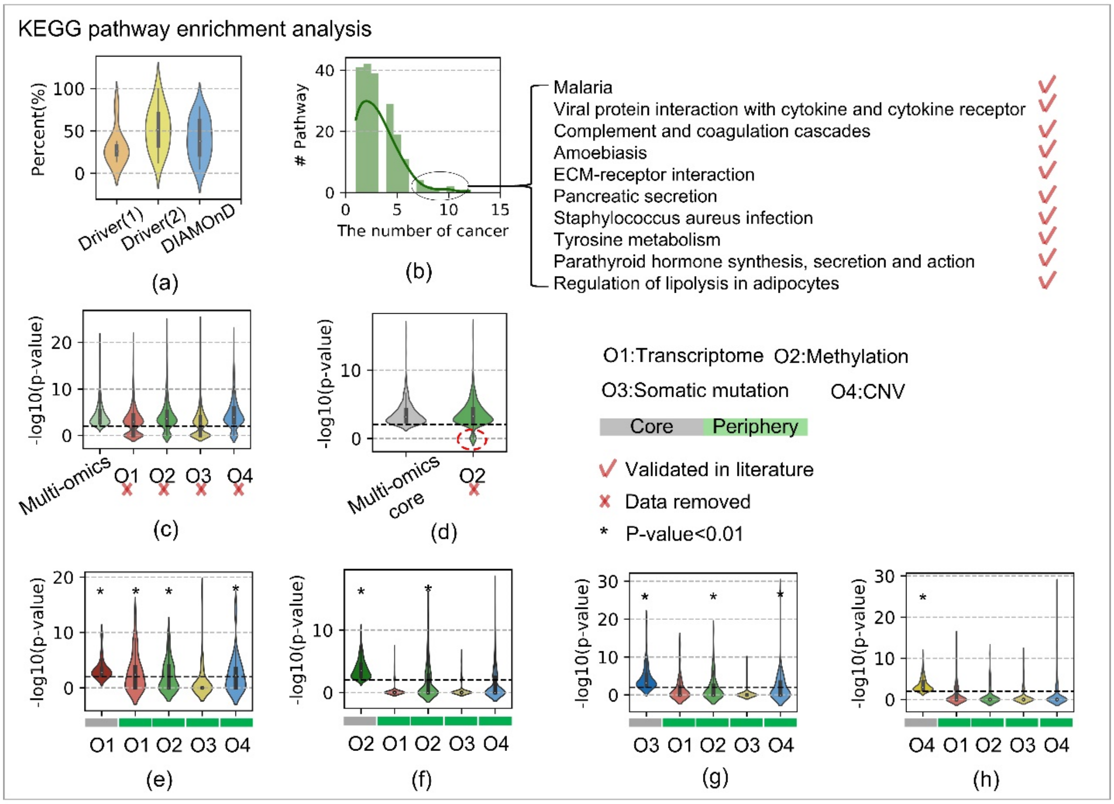

Supplementary Figure 12. KEGG pathway enrichment analysis of omnigenic neighbourhood. All details are gathered in Supplementary Table 11. Four omics aspects are distinguished by O1, O2, O3, and O4, and the core and periphery are marked by grey and green grids respectively. The black dash lines highlight the threshold (p-value=0.01, hypergeometric test) of significance. (a) The violin charts show the percentages of KEGG pathways which are enriched by polygenic modules (p-value<0.01) and further improved by omnigenic neighbourhood. In cancers, average improvement ratios are 31.5%, 52.2% and 41%respectively. (b) The distribution of the number of cancers enriched in KEGG pathway. We indicate the functional pathways enriched only by peripheries in at least 7 cancers. The underlying associations between these pathways and cancer can be validated (100%, highlighted by red check marks) in literature. (c) The violin charts show the enrichment significance in KEGG pathways (average -log10(p-value)= 4.5) of multi-omics omnigenic neighbourhood. The significance of enrichment changes to average 3.05, 4.03, 2.57 and 4.52 when we remove O1, O2, O3 and O4 in our tests respectively. (d) The grey violin chart shows the enrichment significance of multi-omics core. The green violin indicates that after

removing the O2 (Methylation) core, some KEGG pathways loss their underlying association with cancer (red dotted circle), such as inflammatory mediator regulation of TRP channels. (e-f) The violin charts show the enrichment significance in KEGG pathways of core (grey grid) from one omics. Comparing with that, we show the significance levels of these KEGG pathways enriched by peripheries (green grids) from other omics respectively. The error bars indicate the 95% confidence intervals.

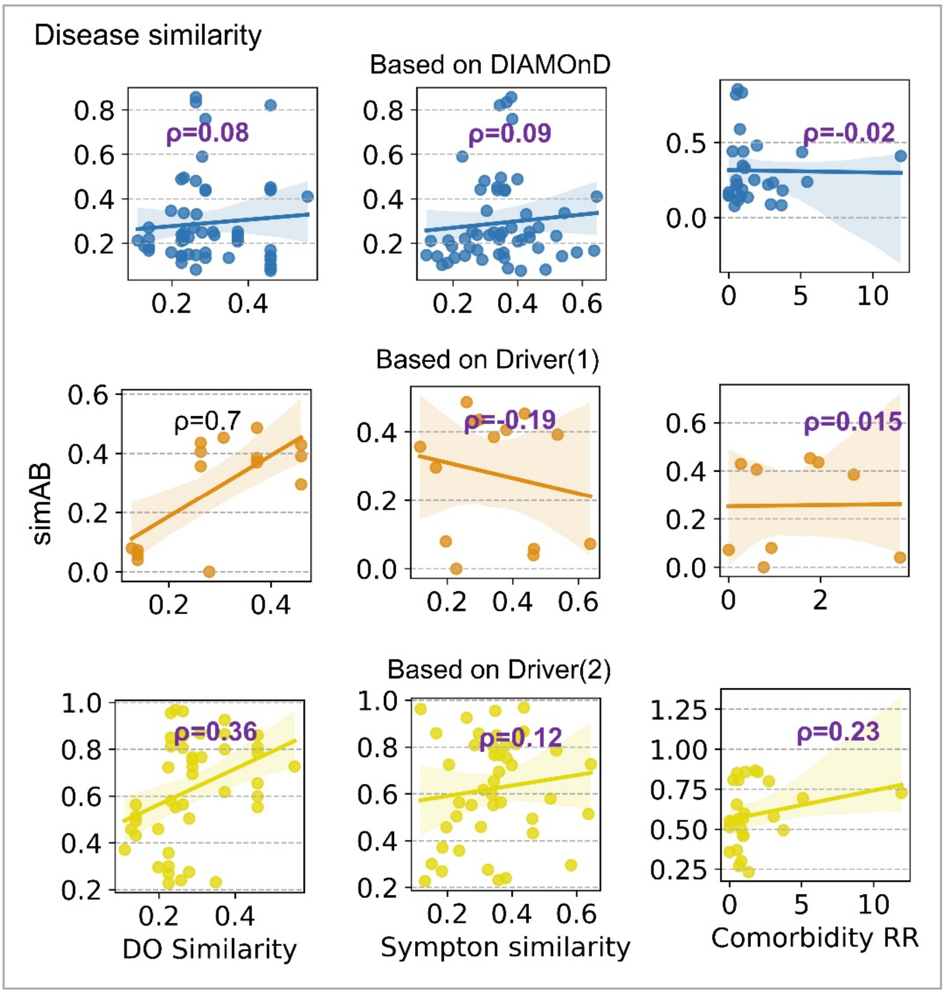

Supplementary Figure 13. The polygenic module portrays cancer similarity. We used simAB to calculate the relationship between cancers (Methods), where the blue image represents the results based on DIAMOnD module, the orange image represents the results based on a group of cancer driver genes (indicated by Driver(1)) , and the yellow image represents the results based on another group of cancer driver genes (indicated by Driver(2)). The points represent the similarities between cancers. We verified the results by comparison with DO similarity, symptom similarity, and comorbidity RR. The fitted line is the Pearson correlation coefficient between the predicted and known similarity between cancers. The shading indicates the 95% confidence interval. In the three similarity verification experiments, 89% (8 in 9 tests, highlighted by purple) correlation coefficients based on polygenic modules are weaker than the results based on the omnigenic neighbourhood.

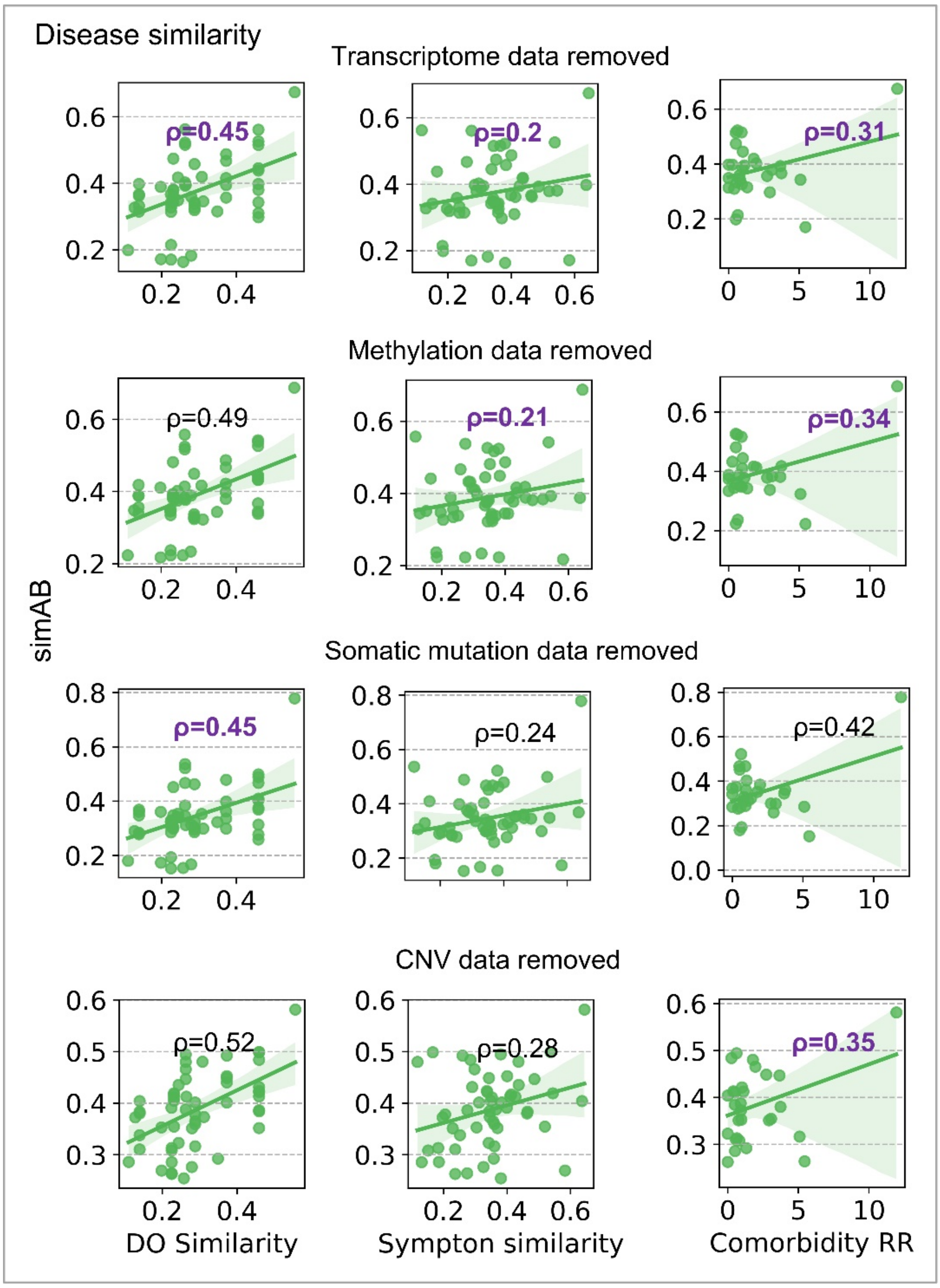

Supplementary Figure 14. Cancer similarity under removing one omics data. We used simAB to calculate the relationship between cancers. When we remove the transcriptome, methylation, somatic mutation and CNV respectively, we calculate the similarities between cancers based on other three omics aspects. We verified the results by comparison with DO similarity, symptom similarity, and comorbidity RR. The fitted line is the Pearson correlation coefficient between the predicted and known similarity between cancers. The shading indicates the 95% confidence interval. After removal of transcriptome and methylation, 83% (5 in 6 tests, highlighted by purple) coefficients are weaker than the results based on the omnigenic neighbourhood.

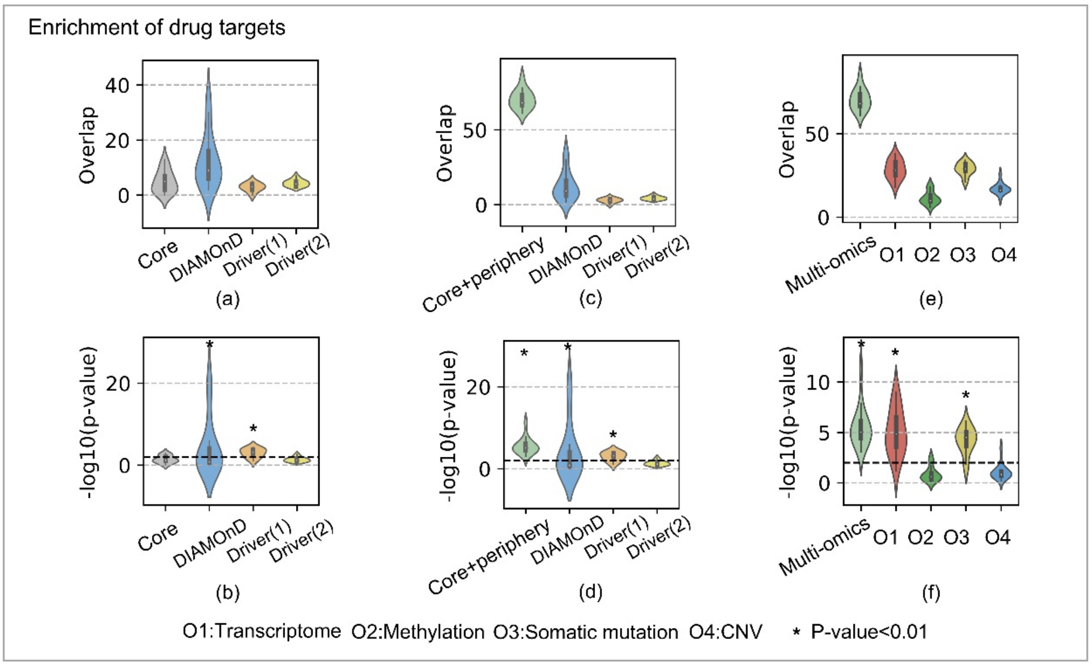

Supplementary Figure 15. Enrichment analysis of drug targets. We collected 229 protein targets of 72 approved drugs for 12 cancers (Details in Supplementary Table 12). Four omics aspects are distinguished by O1, O2, O3, and O4, and the core and periphery are marked by grey and green grids respectively. The black dash lines highlight the threshold (p-value=0.05, hypergeometric test) of significance. (a-b) The violin charts show the numbers (average 4.93, 12.47, 2.88 and 4.38) of drug target in multi-omics core, DIAMOnD, Driver(1) and Driver(2), and their respective statistical significances (-log10(p-value) average 1.45, 4.42, 3.0 and 1.38). (c-d) The violin charts show the number (average 70.3) of drug target in multi-omics core and periphery, and its statistical significance (-log10(p-value) average 5.55). (e-f) The violin charts show the numbers (average 28.4, 11.5, 28.8 and 17.07) of drug target in core and periphery of each O1, O2, O3 and O4, and their statistical significances (-log10(p-value) average 5.0, 0.88, 4.21 and 1.08) respectively. The error bars indicate the 95% confidence intervals.
