# Supplementary material for: Multi-omics peripheral and core regions of cancer
Source: NPJ Syst Biol Appl. 2022 Nov 29;8:47. doi: 10.1038/s41540-022-00258-1 (PMC9707100; doi:10.1038/s41540-022-00258-1)
Supplement: Supplementary file 2 — Supplementary Table 1 [file 41540_2022_258_MOESM2_ESM.pdf]

Supplementary Table 1

			M4
M0	Phyla	Significant with FDR < 0.05	8
		increased in metformin	6
		decreased in metformin	2
	Families	Significant with FDR < 0.05	20
		increased in metformin	10
		decreased in metformin	10
	Genera	Significant with FDR < 0.05	23
		increased in metformin	8
		decreased in metformin	15
	MSPs	Significant with FDR < 0.05	72
		increased in metformin	59
		decreased in metformin	13

Phyla	M4vsM0
Proteobacteria	increased in Metformin
Firmicutes	decreased_in_metformin
Firmicutes	increased in Metformin
Bacteroidetes	decreased in metformin
Verrucomicrobia	increased in Metformin
Actinobacteria	increased in Metformin
Bacteroidetes	increased in Metformin
Candidatus Melainabacteria	increased in Metformin

Family	M4vsM0
Enterobacteriaceae	increased in Metformin
Peptostreptococcaceae	decreased_in_metformin
Lachnospiraceae	increased in Metformin
Clostridiaceae	decreased in metformin
Ruminococcaceae	decreased in metformin
Lachnospiraceae	decreased in metformin
Barnesiellaceae	decreased in metformin
Erysipelotrichaceae	decreased in metformin
Rikenellaceae	decreased in metformin
Clostridiaceae	increased in Metformin
Ruminococcaceae	increased in Metformin
Christensenellaceae	decreased_in_metformin
Akkermansiaceae	increased in Metformin
Bifidobacteriaceae	increased in Metformin
Eubacteriaceae	decreased_in_metformin
Bacteroidaceae	decreased in metformin
Odoribacteraceae	increased_in_Metformin
Erysipelotrichaceae	increased in Metformin
Candidatus Gastranaerophilales	increased in Metformin
Eggerthellaceae	increased in Metformin

Genus	M4vsM0
Escherichia	increased in Metformin
Intestinibacter	decreased_in_metformin
Blautia	increased in Metformin
Clostridium	decreased in metformin
Faecalibacterium	decreased in metformin
Romboutsia	decreased in metformin
Roseburia	decreased in metformin
Ruminococcus	decreased in metformin
Flavonifractor	decreased in metformin
Barnesiella	decreased in metformin
Coprobacillus	decreased in metformin
Alistipes	decreased_in_metformin
Gemmiger	decreased in metformin
Clostridium	increased in Metformin
Ruminococcus	increased_in_Metformin
Akkermansia	increased in Metformin
Bifidobacterium	increased_in_Metformin
Eubacterium	decreased in metformin
Bacteroides	decreased in metformin
Candidatus Gastranaerophilales	increased in Metformin
Anaerotruncus	decreased_in_metformin
Gordonibacter	increased_in_Metformin
Coprobacter	decreased in metformin

MSP	Pvalue	wilcoxon_FDR	abundance_means
msp_0005	4,50E-11	3,59E-08	increased_in_Metformin
msp_0621	2,24E-10	8,92E-08	decreased_in_metformin
msp_0125	6,23E-10	1,65E-07	decreased_in_metformin
msp_0760	2,84E-08	5,65E-06	decreased_in_metformin
msp_0860	9,04E-08	1,44E-05	decreased_in_metformin
msp_0076	1,72E-07	2,29E-05	increased_in_Metformin
msp_0362	3,06E-07	3,48E-05	decreased_in_metformin
msp_0389	4,23E-07	4,21E-05	decreased_in_metformin
msp_0461	5,57E-07	4,94E-05	decreased_in_metformin
msp_0521	3,80E-06	0,000302804	decreased_in_metformin
msp_0422	6,21E-06	0,000450263	decreased_in_metformin
msp_0079	1,07E-05	0,000713398	increased_in_Metformin
msp_0512	2,32E-05	0,001424457	decreased_in_metformin
msp_0449	5,57E-05	0,003171843	decreased_in_metformin
msp_0074	0,000105838	0,004991657	decreased_in_metformin
msp_0244	0,000112735	0,004991657	increased_in_Metformin
msp_0430	0,000108577	0,004991657	decreased_in_metformin
msp_0723	0,000109717	0,004991657	decreased_in_metformin
msp_0655	0,000165916	0,006682001	increased_in_Metformin
msp_1195	0,000167679	0,006682001	decreased_in_metformin
msp_0847	0,000216213	0,008205801	decreased_in_metformin
msp_0596	0,000232427	0,008420196	decreased_in_metformin
msp_0811	0,000253633	0,008788934	decreased_in_metformin
msp_0250	0,000276849	0,008861335	decreased_in_metformin
msp_0314	0,000289077	0,008861335	decreased_in_metformin
msp_1323	0,000289077	0,008861335	decreased_in_metformin
msp_0055	0,000500348	0,0135281	decreased_in_metformin
msp_0059	0,000509213	0,0135281	decreased_in_metformin
msp_0132	0,000496492	0,0135281	decreased_in_metformin
msp_0225	0,000479752	0,0135281	decreased_in_metformin
msp_0456	0,000646557	0,016622769	decreased_in_metformin
msp_0159	0,000730308	0,018189223	decreased_in_metformin
msp_0697	0,000849819	0,019920759	decreased_in_metformin
msp_1302	0,000849819	0,019920759	decreased_in_metformin
msp_0066	0,001049448	0,020910261	increased_in_Metformin
msp_0124	0,001038755	0,020910261	decreased_in_metformin
msp_0152	0,00100007	0,020910261	increased_in_Metformin
msp_0173	0,000958119	0,020910261	decreased_in_metformin
msp_0312	0,000936609	0,020910261	decreased_in_metformin
msp_0820	0,001023428	0,020910261	decreased_in_metformin
msp_0025	0,001187438	0,021410033	increased_in_Metformin
msp_0138	0,001229742	0,021410033	increased_in_Metformin
msp_0227	0,001174177	0,021410033	decreased_in_metformin
msp_0412	0,001202242	0,021410033	decreased_in_metformin
msp_0563	0,001161613	0,021410033	decreased_in_metformin
msp_0761	0,001235711	0,021410033	decreased_in_metformin
msp_0022	0,001681733	0,027387397	decreased_in_metformin
msp_0296	0,001665398	0,027387397	decreased_in_metformin
msp_0827	0,001683792	0,027387397	increased_in_Metformin

msp_0454	0,001828935	0,029153222	decreased_in_metformin
msp_0047	0,00253633	0,031585241	decreased_in_metformin
msp_0158	0,002133185	0,031585241	decreased_in_metformin
msp_0324	0,00233069	0,031585241	decreased_in_metformin
msp_0334	0,002031677	0,031585241	increased_in_Metformin
msp_0407	0,002507688	0,031585241	decreased_in_metformin
msp_0455	0,002181863	0,031585241	increased_in_Metformin
msp_0569	0,002468774	0,031585241	decreased_in_metformin
msp_0676	0,002468774	0,031585241	decreased_in_metformin
msp_0821	0,002270592	0,031585241	decreased_in_metformin
msp_0867	0,002468774	0,031585241	decreased_in_metformin
msp_1290	0,002468774	0,031585241	decreased_in_metformin
msp_1339	0,002181863	0,031585241	increased_in_Metformin
msp_1343	0,002468774	0,031585241	decreased_in_metformin
msp_1589	0,002468774	0,031585241	decreased_in_metformin
msp_0388	0,002848707	0,034929532	decreased_in_metformin
msp_0398	0,003130291	0,037800638	decreased_in_metformin
msp_0467	0,003395611	0,040392563	decreased_in_metformin
msp_0198	0,004194943	0,046435691	decreased_in_metformin
msp_0451	0,004194943	0,046435691	decreased_in_metformin
msp_0699	0,004194943	0,046435691	decreased_in_metformin
msp_0872	0,004194943	0,046435691	decreased_in_metformin
msp_1069	0,004194943	0,046435691	decreased_in_metformin

Genus	Pvalue	wilcoxon_	dysbiosis
Escherichia	4,50E-11	3,59E-08	increased_in_Metformin
Intestinibacter	2,24E-10	8,92E-08	decreased_in_metformin
unclassified Clostridiales	6,23E-10	1,65E-07	decreased_in_metformin
unclassified Clostridiales	2,84E-08	5,65E-06	decreased_in_metformin
Intestinibacter	9,04E-08	1,44E-05	decreased_in_metformin
Blautia	1,72E-07	2,29E-05	increased_in_Metformin
Clostridium	3,06E-07	3,48E-05	decreased_in_metformin
Faecalibacterium	4,23E-07	4,21E-05	decreased_in_metformin
unclassified Clostridiales	5,57E-07	4,94E-05	decreased_in_metformin
unclassified Firmicutes	3,80E-06	0,000303	decreased_in_metformin
Romboutsia	6,21E-06	0,00045	decreased_in_metformin
unclassified Clostridiales	1,07E-05	0,000713	increased_in_Metformin
unclassified Firmicutes	2,32E-05	0,001424	decreased_in_metformin
unclassified	5,57E-05	0,003172	decreased_in_metformin
Roseburia	0,000105838	0,004992	decreased_in_metformin
Blautia	0,000112735	0,004992	increased_in_Metformin
unclassified	0,000108577	0,004992	decreased_in_metformin
unclassified Firmicutes	0,000109717	0,004992	decreased_in_metformin
unclassified Clostridiales	0,000165916	0,006682	increased_in_Metformin
unclassified Firmicutes	0,000167679	0,006682	decreased_in_metformin
unclassified	0,000216213	0,008206	decreased_in_metformin
unclassified Firmicutes	0,000232427	0,00842	decreased_in_metformin
unclassified Firmicutes	0,000253633	0,008789	decreased_in_metformin
Ruminococcus	0,000276849	0,008861	decreased_in_metformin
unclassified Clostridiales	0,000289077	0,008861	decreased_in_metformin
Flavonifractor	0,000289077	0,008861	decreased_in_metformin
unclassified Clostridiales	0,000500348	0,013528	decreased_in_metformin
Barnesiella	0,000509213	0,013528	decreased_in_metformin
Coprobacillus	0,000496492	0,013528	decreased_in_metformin
Alistipes	0,000479752	0,013528	decreased_in_metformin
Gemmiger	0,000646557	0,016623	decreased_in_metformin
unclassified	0,000730308	0,018189	decreased_in_metformin
unclassified Clostridiales	0,000849819	0,019921	decreased_in_metformin
unclassified Firmicutes	0,000849819	0,019921	decreased_in_metformin
Clostridium	0,001049448	0,02091	increased_in_Metformin
unclassified Ruminococcaceae	0,001038755	0,02091	decreased_in_metformin
Ruminococcus	0,00100007	0,02091	increased_in_Metformin
unclassified Christensenellaceae	0,000958119	0,02091	decreased_in_metformin
unclassified Firmicutes	0,000936609	0,02091	decreased_in_metformin
unclassified Clostridiales	0,001023428	0,02091	decreased_in_metformin
Akkermansia	0,001187438	0,02141	increased_in_Metformin
Bifidobacterium	0,001229742	0,02141	increased_in_Metformin
Eubacterium	0,001174177	0,02141	decreased_in_metformin
Bacteroides	0,001202242	0,02141	decreased_in_metformin
Clostridium	0,001161613	0,02141	decreased_in_metformin
unclassified Clostridiales	0,001235711	0,02141	decreased_in_metformin
Bacteroides	0,001681733	0,027387	decreased_in_metformin
unclassified Lachnospiraceae	0,001665398	0,027387	decreased_in_metformin
unclassified Odoribacteraceae	0,001683792	0,027387	increased_in_Metformin
Clostridium	0,001828935	0,029153	decreased_in_metformin
Eubacterium	0,00253633	0,031585	decreased_in_metformin
Eubacterium	0,002133185	0,031585	decreased_in_metformin
Faecalibacterium	0,00233069	0,031585	decreased_in_metformin
unclassified Erysipelotrichaceae	0,002031677	0,031585	increased_in_Metformin
Gemmiger	0,002507688	0,031585	decreased_in_metformin
Candidatus Gastranaerophilales	0,002181863	0,031585	increased_in_Metformin
Anaerotruncus	0,002468774	0,031585	decreased_in_metformin
unclassified	0,002468774	0,031585	decreased_in_metformin
unclassified Firmicutes	0,002270592	0,031585	decreased_in_metformin
unclassified Firmicutes	0,002468774	0,031585	decreased_in_metformin
unclassified Firmicutes	0,002468774	0,031585	decreased_in_metformin
Gordonibacter	0,002181863	0,031585	increased_in_Metformin
unclassified Clostridiales	0,002468774	0,031585	decreased_in_metformin
unclassified	0,002468774	0,031585	decreased_in_metformin
Faecalibacterium	0,002848707	0,03493	decreased_in_metformin
unclassified	0,003130291	0,037801	decreased_in_metformin
unclassified Firmicutes	0,003395611	0,040393	decreased_in_metformin
Coprobacter	0,004194943	0,046436	decreased_in_metformin
Ruminococcus	0,004194943	0,046436	decreased_in_metformin
unclassified Firmicutes	0,004194943	0,046436	decreased_in_metformin
unclassified Clostridiales	0,004194943	0,046436	decreased_in_metformin
unclassified Clostridiales	0,004194943	0,046436	decreased_in_metformin

Family	Pvalue	wilcoxon_	dysbiosis
Enterobacteriaceae	4,50E-11	3,59E-08	increased_in_Metformin
Peptostreptococcaceae	2,24E-10	8,92E-08	decreased_in_metformin
unclassified Clostridiales	6,23E-10	1,65E-07	decreased_in_metformin
unclassified Clostridiales	2,84E-08	5,65E-06	decreased_in_metformin
Peptostreptococcaceae	9,04E-08	1,44E-05	decreased_in_metformin
Lachnospiraceae	1,72E-07	2,29E-05	increased_in_Metformin
Clostridiaceae	3,06E-07	3,48E-05	decreased_in_metformin
Ruminococcaceae	4,23E-07	4,21E-05	decreased_in_metformin
unclassified Clostridiales	5,57E-07	4,94E-05	decreased_in_metformin
unclassified Firmicutes	3,80E-06	0,000303	decreased_in_metformin
Peptostreptococcaceae	6,21E-06	0,00045	decreased_in_metformin
unclassified Clostridiales	1,07E-05	0,000713	increased_in_Metformin
unclassified Firmicutes	2,32E-05	0,001424	decreased_in_metformin
unclassified	5,57E-05	0,003172	decreased_in_metformin
Lachnospiraceae	0,000106	0,004992	decreased_in_metformin
Lachnospiraceae	0,000113	0,004992	increased_in_Metformin
unclassified	0,000109	0,004992	decreased_in_metformin
unclassified Firmicutes	0,00011	0,004992	decreased_in_metformin
unclassified Clostridiales	0,000166	0,006682	increased_in_Metformin
unclassified Firmicutes	0,000168	0,006682	decreased_in_metformin
unclassified	0,000216	0,008206	decreased_in_metformin
unclassified Firmicutes	0,000232	0,00842	decreased_in_metformin
unclassified Firmicutes	0,000254	0,008789	decreased_in_metformin
Ruminococcaceae	0,000277	0,008861	decreased_in_metformin
unclassified Clostridiales	0,000289	0,008861	decreased_in_metformin
unclassified Clostridiales	0,000289	0,008861	decreased_in_metformin
unclassified Clostridiales	0,0005	0,013528	decreased_in_metformin
Barnesiellaceae	0,000509	0,013528	decreased_in_metformin
Erysipelotrichaceae	0,000496	0,013528	decreased_in_metformin
Rikenellaceae	0,00048	0,013528	decreased_in_metformin
Ruminococcaceae	0,000647	0,016623	decreased_in_metformin
unclassified	0,00073	0,018189	decreased_in_metformin
unclassified Clostridiales	0,00085	0,019921	decreased_in_metformin
unclassified Firmicutes	0,00085	0,019921	decreased_in_metformin
Clostridiaceae	0,001049	0,02091	increased_in_Metformin
Ruminococcaceae	0,001039	0,02091	decreased_in_metformin
Ruminococcaceae	0,001	0,02091	increased_in_Metformin
Christensenellaceae	0,000958	0,02091	decreased_in_metformin
unclassified Firmicutes	0,000937	0,02091	decreased_in_metformin
unclassified Clostridiales	0,001023	0,02091	decreased_in_metformin
Akkermansiaceae	0,001187	0,02141	increased_in_Metformin
Bifidobacteriaceae	0,00123	0,02141	increased_in_Metformin
Eubacteriaceae	0,001174	0,02141	decreased_in_metformin
Bacteroidaceae	0,001202	0,02141	decreased_in_metformin
Clostridiaceae	0,001162	0,02141	decreased_in_metformin
unclassified Clostridiales	0,001236	0,02141	decreased_in_metformin
Bacteroidaceae	0,001682	0,027387	decreased_in_metformin
Lachnospiraceae	0,001665	0,027387	decreased_in_metformin
Odoribacteraceae	0,001684	0,027387	increased_in_Metformin
Clostridiaceae	0,001829	0,029153	decreased_in_metformin
Eubacteriaceae	0,002536	0,031585	decreased_in_metformin
Eubacteriaceae	0,002133	0,031585	decreased_in_metformin
Ruminococcaceae	0,002331	0,031585	decreased_in_metformin
Erysipelotrichaceae	0,002032	0,031585	increased_in_Metformin
Ruminococcaceae	0,002508	0,031585	decreased_in_metformin
Candidatus Gastranaerophilales	0,002182	0,031585	increased_in_Metformin
Ruminococcaceae	0,002469	0,031585	decreased_in_metformin
unclassified	0,002469	0,031585	decreased_in_metformin
unclassified Firmicutes	0,002271	0,031585	decreased_in_metformin
unclassified Firmicutes	0,002469	0,031585	decreased_in_metformin
unclassified Firmicutes	0,002469	0,031585	decreased_in_metformin
Eggerthellaceae	0,002182	0,031585	increased_in_Metformin
unclassified Clostridiales	0,002469	0,031585	decreased_in_metformin
unclassified	0,002469	0,031585	decreased_in_metformin
Ruminococcaceae	0,002849	0,03493	decreased_in_metformin
unclassified	0,00313	0,037801	decreased_in_metformin
unclassified Firmicutes	0,003396	0,040393	decreased_in_metformin
Barnesiellaceae	0,004195	0,046436	decreased_in_metformin
Ruminococcaceae	0,004195	0,046436	decreased_in_metformin
unclassified Firmicutes	0,004195	0,046436	decreased_in_metformin
unclassified Clostridiales	0,004195	0,046436	decreased_in_metformin
unclassified Clostridiales	0,004195	0,046436	decreased_in_metformin

Phyla	Pvalue	wilcoxon_	dysbiosis
Proteobacteria	4,50E-11	3,59E-08	increased_in_Metformin
Firmicutes	2,24E-10	8,92E-08	decreased_in_metformin
Firmicutes	6,23E-10	1,65E-07	decreased_in_metformin
Firmicutes	2,84E-08	5,65E-06	decreased_in_metformin
Firmicutes	9,04E-08	1,44E-05	decreased_in_metformin
Firmicutes	1,72E-07	2,29E-05	increased_in_Metformin
Firmicutes	3,06E-07	3,48E-05	decreased_in_metformin
Firmicutes	4,23E-07	4,21E-05	decreased_in_metformin
Firmicutes	5,57E-07	4,94E-05	decreased_in_metformin
Firmicutes	3,80E-06	0,000303	decreased_in_metformin
Firmicutes	6,21E-06	0,00045	decreased_in_metformin
Firmicutes	1,07E-05	0,000713	increased_in_Metformin
Firmicutes	2,32E-05	0,001424	decreased_in_metformin
unclassified	5,57E-05	0,003172	decreased_in_metformin
Firmicutes	0,000106	0,004992	decreased_in_metformin
Firmicutes	0,000113	0,004992	increased_in_Metformin
unclassified	0,000109	0,004992	decreased_in_metformin
Firmicutes	0,00011	0,004992	decreased_in_metformin
Firmicutes	0,000166	0,006682	increased_in_Metformin
Firmicutes	0,000168	0,006682	decreased_in_metformin
unclassified	0,000216	0,008206	decreased_in_metformin
Firmicutes	0,000232	0,00842	decreased_in_metformin
Firmicutes	0,000254	0,008789	decreased_in_metformin
Firmicutes	0,000277	0,008861	decreased_in_metformin
Firmicutes	0,000289	0,008861	decreased_in_metformin
Firmicutes	0,000289	0,008861	decreased_in_metformin
Firmicutes	0,0005	0,013528	decreased_in_metformin
Bacteroidetes	0,000509	0,013528	decreased_in_metformin
Firmicutes	0,000496	0,013528	decreased_in_metformin
Bacteroidetes	0,00048	0,013528	decreased_in_metformin
Firmicutes	0,000647	0,016623	decreased_in_metformin
unclassified	0,00073	0,018189	decreased_in_metformin
Firmicutes	0,00085	0,019921	decreased_in_metformin
Firmicutes	0,00085	0,019921	decreased_in_metformin
Firmicutes	0,001049	0,02091	increased_in_Metformin
Firmicutes	0,001039	0,02091	decreased_in_metformin
Firmicutes	0,001	0,02091	increased_in_Metformin
Firmicutes	0,000958	0,02091	decreased_in_metformin
Firmicutes	0,000937	0,02091	decreased_in_metformin
Firmicutes	0,001023	0,02091	decreased_in_metformin
Verrucomicrobia	0,001187	0,02141	increased_in_Metformin
Actinobacteria	0,00123	0,02141	increased_in_Metformin
Firmicutes	0,001174	0,02141	decreased_in_metformin
Bacteroidetes	0,001202	0,02141	decreased_in_metformin
Firmicutes	0,001162	0,02141	decreased_in_metformin
Firmicutes	0,001236	0,02141	decreased_in_metformin
Bacteroidetes	0,001682	0,027387	decreased_in_metformin
Firmicutes	0,001665	0,027387	decreased_in_metformin
Bacteroidetes	0,001684	0,027387	increased_in_Metformin
Firmicutes	0,001829	0,029153	decreased_in_metformin
Firmicutes	0,002536	0,031585	decreased_in_metformin
Firmicutes	0,002133	0,031585	decreased_in_metformin
Firmicutes	0,002331	0,031585	decreased_in_metformin
Firmicutes	0,002032	0,031585	increased_in_Metformin
Firmicutes	0,002508	0,031585	decreased_in_metformin
Candidatus Melainabacter	0,002182	0,031585	increased_in_Metformin
Firmicutes	0,002469	0,031585	decreased_in_metformin
unclassified	0,002469	0,031585	decreased_in_metformin
Firmicutes	0,002271	0,031585	decreased_in_metformin
Firmicutes	0,002469	0,031585	decreased_in_metformin
Firmicutes	0,002469	0,031585	decreased_in_metformin
Actinobacteria	0,002182	0,031585	increased_in_Metformin
Firmicutes	0,002469	0,031585	decreased_in_metformin
unclassified	0,002469	0,031585	decreased_in_metformin
Firmicutes	0,002849	0,03493	decreased_in_metformin
unclassified	0,00313	0,037801	decreased_in_metformin
Firmicutes	0,003396	0,040393	decreased_in_metformin
Bacteroidetes	0,004195	0,046436	decreased_in_metformin
Firmicutes	0,004195	0,046436	decreased_in_metformin
Firmicutes	0,004195	0,046436	decreased_in_metformin
Firmicutes	0,004195	0,046436	decreased_in_metformin
Firmicutes	0,004195	0,046436	decreased_in_metformin

Supplementary Table 2

M0

Species	Species	corr	CorType
msp_0005	msp_0074	-0.2726598	Negative
msp_0005	msp_0079	-0.4664402	Negative
msp_0005	msp_0362	0.3478552	Positive
msp_0025	msp_0066	-0.3320167	Negative
msp_0025	msp_0076	-0.4755613	Negative
msp_0025	msp_0422	0.3509567	Positive
msp_0025	msp_0811	0.5634897	Positive
msp_0066	msp_0244	-0.2538672	Negative
msp_0066	msp_0811	-0.2726598	Negative
msp_0066	msp_0074	-0.2984557	Negative
msp_0066	msp_0362	-0.3320167	Negative
msp_0066	msp_0760	-0.3814797	Negative
msp_0066	msp_0125	-0.3983866	Negative
msp_0066	msp_0621	-0.4511125	Negative
msp_0066	msp_0422	-0.5058021	Negative
msp_0066	msp_0076	0.5445784	Positive
msp_0074	msp_0655	-0.3693674	Negative
msp_0074	msp_0244	0.3952547	Positive
msp_0074	msp_0760	0.3977302	Positive
msp_0076	msp_0422	-0.4035341	Negative
msp_0076	msp_0760	-0.5056420	Negative
msp_0076	msp_0125	-0.5808999	Negative
msp_0076	msp_0811	-0.6239327	Negative
msp_0076	msp_0079	0.4591453	Positive
msp_0079	msp_0811	-0.4975934	Negative
msp_0079	msp_0760	-0.5324609	Negative
msp_0125	msp_0655	-0.5015950	Negative
msp_0125	msp_0811	0.2475613	Positive
msp_0125	msp_0512	0.3181039	Positive
msp_0125	msp_0362	0.3309859	Positive
msp_0125	msp_0422	0.3676952	Positive
msp_0152	msp_0244	0.2963848	Positive
msp_0152	msp_0621	0.3547463	Positive
msp_0152	msp_0362	0.4667818	Positive
msp_0244	msp_0389	0.4706921	Positive
msp_0250	msp_0621	0.3006957	Positive
msp_0362	msp_0422	0.3294034	Positive
msp_0362	msp_0621	0.5379909	Positive
msp_0389	msp_0422	0.4454114	Positive
msp_0422	msp_0621	0.3209886	Positive
msp_0760	msp_0811	0.4708686	Positive

M4

Species	Species	corr	CorType
msp_0005	msp_0244	-0.5939909	Negative
msp_0005	msp_0079	-0.5046229	Negative
msp_0005	msp_0066	0.3820380	Positive
msp_0025	msp_0389	0.3476127	Positive
msp_0066	msp_0389	-0.5643097	Negative
msp_0066	msp_0125	-0.5229348	Negative
msp_0066	msp_0244	-0.4419999	Negative
msp_0066	msp_0422	-0.3817603	Negative
msp_0076	msp_0250	-0.4530065	Negative
msp_0076	msp_0244	-0.4142650	Negative
msp_0076	msp_0655	-0.3141626	Negative
msp_0076	msp_0138	0.1852153	Positive
msp_0079	msp_0138	-0.5547457	Negative
msp_0079	msp_0655	0.3501638	Positive
msp_0079	msp_0250	0.4365825	Positive
msp_0079	msp_0244	0.6010256	Positive
msp_0125	msp_0152	0.3665624	Positive
msp_0125	msp_0422	0.4444515	Positive
msp_0138	msp_0244	-0.3582897	Negative
msp_0152	msp_0244	0.3447013	Positive
msp_0250	msp_0621	-0.3108638	Negative
msp_0250	msp_0422	-0.2528858	Negative
msp_0250	msp_0655	0.2731918	Positive
msp_0389	msp_0422	0.3565490	Positive
msp_0389	msp_0621	0.4531589	Positive

Same interaction

Lost interaction

New interatcion

M0

Species	Species	correlation	Correlation type
Escherichia coli	Clostridium sp. CAG:7	-0.466440296	Negative
Clostridium sp. AT4	Ruminococcus sp. CAG:60 / Blautia sp. 2789STDY560883	-0.253867247	Negative
Clostridium sp. AT4	Firmicutes bacterium CAG:24 / Clostridium sp. 2789STDY	-0.398386639	Negative
Clostridium sp. AT4	Romboutsia timonensis	-0.505802108	Negative
Firmicutes bacterium CAG:2	[Firmicutes] bacterium CAG:321	0.318103963	Positive
Firmicutes bacterium CAG:2	Romboutsia timonensis	0.367695219	Positive
Ruminococcus faecis	Ruminococcus sp. CAG:60 / Blautia sp. 2789STDY560883	0.296384877	Positive
Ruminococcus lactaris	Intestinibacter bartlettii	0.300695739	Positive
Faecalibacterium prausnitzii	Romboutsia timonensis	0.445411406	Positive

M4

Species	Species	correlation	Correlation type
Escherichia coli	Clostridium sp. CAG:7	-0.504622986568393	Negative
Clostridium sp. AT4	Ruminococcus sp. CAG:60 / Blautia sp. 2789STDY560883	-0.522934877686852	Negative
Clostridium sp. AT4	Firmicutes bacterium CAG:24 / Clostridium sp. 2789STDY	-0.441999971771285	Negative
Clostridium sp. AT4	Romboutsia timonensis	-0.381760379609671	Negative
Firmicutes bacterium CAG:2	[Firmicutes] bacterium CAG:321	0.36656247038735	Positive
Firmicutes bacterium CAG:2	Romboutsia timonensis	0.444451530943913	Positive
Ruminococcus faecis	Ruminococcus sp. CAG:60 / Blautia sp. 2789STDY560883	0.344701336943509	Positive
Ruminococcus lactaris	Intestinibacter bartlettii	-0.310863814342674	Negative
Faecalibacterium prausnitzii	Romboutsia timonensis	0.356549013469671	Positive

M4

Species	Species	Correlation	Correlation type
Escherichia coli	Ruminococcus sp. CAG:60 / Blautia sp. 2789STDY5608836	-0.593990932605943	Negative
Escherichia coli	Clostridium sp. AT4	0.382038038575852	Positive
Akkermansia muciniphila	Faecalibacterium prausnitzii 4	0.347612731075978	Positive
Clostridium sp. AT4	Faecalibacterium prausnitzii 4	-0.564309782761286	Negative
Blautia wexlerae	Ruminococcus lactaris	-0.453006591170668	Negative
Blautia wexlerae	Ruminococcus sp. CAG:60 / Blautia sp. 2789STDY5608836	-0.414265026640942	Negative
Blautia wexlerae	Ruminococcus sp. 2789STDY5608817 / Eubacterium sp. 2789STDY5834872	-0.314162651721888	Negative
Blautia wexlerae	Bifidobacterium pseudocatenulatum	0.185215327412714	Positive
Clostridium sp. CAG:7	Bifidobacterium pseudocatenulatum	-0.554745738835034	Negative
Clostridium sp. CAG:7	Ruminococcus sp. 2789STDY5608817 / Eubacterium sp. 2789STDY5834872	0.350163885809912	Positive
Clostridium sp. CAG:7	Ruminococcus lactaris	0.436582590079039	Positive
Clostridium sp. CAG:7	Ruminococcus sp. CAG:60 / Blautia sp. 2789STDY5608836	0.601025666534806	Positive
Bifidobacterium pseudocatenulatum	Ruminococcus sp. CAG:60 / Blautia sp. 2789STDY5608836	-0.358289790510688	Negative
Ruminococcus lactaris	Romboutsia timonensis	-0.252885817318549	Negative
Ruminococcus lactaris	Ruminococcus sp. 2789STDY5608817 / Eubacterium sp. 2789STDY5834872	0.273191844854909	Positive
Faecalibacterium prausnitzii 4	Intestinibacter bartlettii	0.45315897615201	Positive

Supplementary Table 3

annot1	subsConversion	references
CBM20	Starch,other_storage_CH	DOI: 10.1016/j.celrep.2019.02.090
CBM32	Mucine,host_glycans	DOI: 10.1016/j.celrep.2019.02.090
CBM34	Starch,other_storage_CH	DOI: 10.1016/j.celrep.2019.02.090
CBM48	Starch,other_storage_CH	DOI: 10.1016/j.celrep.2019.02.090
CBM50	Mucine,host_glycans	DOI: 10.1016/j.celrep.2019.02.090
CBM6	Cellulose,hemicellulose,b-glucans	DOI: 10.1016/j.celrep.2019.02.090
CBM77	Pectins_and_mannen	DOI: 10.1016/j.celrep.2019.02.090
GH1	Multiple_polysaccharides	DOI: 10.1016/j.celrep.2019.02.090
GH10	Cellulose,hemicellulose,b-glucans	DOI: 10.1016/j.celrep.2019.02.090
GH105	Pectins_and_mannen	DOI: 10.1016/j.celrep.2019.02.090
GH106	Pectins_and_mannen	DOI: 10.1104/pp.105.072652
GH109	Mucine,host_glycans	DOI: 10.1016/j.celrep.2019.02.090
GH110	Multiple_polysaccharides	DOI: 10.1016/j.celrep.2019.02.090
GH112	Multiple_polysaccharides	DOI: 10.1016/j.celrep.2019.02.090
GH115	Cellulose,hemicellulose,b-glucans	DOI: 10.1016/j.celrep.2019.02.090
GH123	Mucine,host_glycans	DOI: 10.1016/j.celrep.2019.02.090
GH125	Fungal_cell_wall_mannan	DOI: 10.1104/pp.105.072652
GH127	Cellulose,hemicellulose,b-glucans	DOI: 10.1016/j.celrep.2019.02.090
GH13	Starch,other_storage_CH	DOI: 10.1016/j.celrep.2019.02.090
GH13	Starch,other_storage_CH	DOI: 10.1016/j.celrep.2019.02.090
GH13	Starch,other_storage_CH	DOI: 10.1016/j.celrep.2019.02.090
GH13	Starch,other_storage_CH	DOI: 10.1016/j.celrep.2019.02.090
GH13	Starch,other_storage_CH	DOI: 10.1016/j.celrep.2019.02.090
GH13	Starch,other_storage_CH	DOI: 10.1016/j.celrep.2019.02.090
GH13	Starch,other_storage_CH	DOI: 10.1016/j.celrep.2019.02.090
GH13	Starch,other_storage_CH	DOI: 10.1016/j.celrep.2019.02.090
GH13	Starch,other_storage_CH	DOI: 10.1016/j.celrep.2019.02.090
GH13	Starch,other_storage_CH	DOI: 10.1016/j.celrep.2019.02.090
GH13	Starch,other_storage_CH	DOI: 10.1016/j.celrep.2019.02.090
GH13	Starch,other_storage_CH	DOI: 10.1016/j.celrep.2019.02.090
GH133	Starch,other_storage_CH	DOI: 10.1016/j.celrep.2019.02.090
GH15	Starch,other_storage_CH	DOI:10.1371/journal.pone.0129275
GH16	Multiple_polysaccharides	DOI: 10.1016/j.celrep.2019.02.090
GH18	Mucine,host_glycans	DOI: 10.1016/j.celrep.2019.02.090
GH2	Multiple_polysaccharides	DOI: 10.1016/j.celrep.2019.02.090
GH20	Mucine,host_glycans	DOI: 10.1016/j.celrep.2019.02.090
GH23	Multiple_polysaccharides	DOI: 10.1016/j.celrep.2019.02.090
GH25	Multiple_polysaccharides	DOI: 10.1016/j.celrep.2019.02.090
GH26	Cellulose,hemicellulose,b-glucans	DOI: 10.1104/pp.105.072652
GH28	Pectins_and_mannen	DOI: 10.1016/j.celrep.2019.02.090
GH3	Multiple_polysaccharides	DOI: 10.1016/j.celrep.2019.02.090
GH30	Cellulose,hemicellulose,b-glucans	DOI: 10.1016/j.celrep.2019.02.090
GH31	Multiple_polysaccharides	DOI: 10.1016/j.celrep.2019.02.090
GH32	Starch,other_storage_CH	DOI: 10.1016/j.celrep.2019.02.090
GH33	Mucine,host_glycans	DOI: 10.1016/j.celrep.2019.02.090

GH35	Multiple_polysaccharides	DOI: 10.1016/j.celrep.2019.02.090
GH36	Starch,other_storage_CH	DOI: 10.1016/j.celrep.2019.02.090
GH38	Fungal_cell_wall_mannan	DOI: 10.1104/pp.105.072652
GH42	Multiple_polysaccharides	DOI: 10.1016/j.celrep.2019.02.090
GH43	Multiple_polysaccharides	DOI: 10.1016/j.celrep.2019.02.090
GH43	Multiple_polysaccharides	DOI: 10.1016/j.celrep.2019.02.090
GH43	Multiple_polysaccharides	DOI: 10.1016/j.celrep.2019.02.090
GH43	Multiple_polysaccharides	DOI: 10.1016/j.celrep.2019.02.090
GH43	Multiple_polysaccharides	DOI: 10.1016/j.celrep.2019.02.090
GH48	Cellulose,hemicellulose,b-glucans	DOI: 10.1111/1462-2920.12217
GH5	Cellulose,hemicellulose,b-glucans	DOI: 10.1016/j.celrep.2019.02.090
GH55	Cellulose,hemicellulose,b-glucans	DOI:10.1371/journal.pone.0129275
GH65	Carbohydrate transport and metabolism	DOI:10.1371/journal.pone.0129275
GH73	Multiple_polysaccharides	DOI: 10.1016/j.celrep.2019.02.090
GH77	Starch,other_storage_CH	DOI: 10.1016/j.celrep.2019.02.090
GH78	Pectins_and_mannen	DOI: 10.1104/pp.105.072652
GH85	Mucine,host_glycans	DOI: 10.1104/pp.105.072652
GH89	Mucine,host_glycans	DOI: 10.1016/j.celrep.2019.02.090
GH9	Cellulose,hemicellulose,b-glucans	DOI: 10.1104/pp.105.072652
GH92	Pectins_and_mannen	DOI: 10.1016/j.celrep.2019.02.090
GH94	Multiple_polysaccharides	DOI: 10.1016/j.celrep.2019.02.090
GH95	Multiple_polysaccharides	DOI: 10.1016/j.celrep.2019.02.090
GH97	Multiple_polysaccharides	DOI: 10.1016/j.celrep.2019.02.090
GT19	LPS_synthesis	DOI: 10.1016/j.celrep.2019.02.090
GT2	Multiple_polysaccharides	DOI: 10.1016/j.celrep.2019.02.090
GT26	Starch,other_storage_CH	DOI: 10.1016/j.celrep.2019.02.090
GT28	Multiple_polysaccharides	DOI: 10.1016/j.celrep.2019.02.090
GT3	Starch,other_storage_CH	DOI: 10.1016/j.celrep.2019.02.090
GT35	Starch,other_storage_CH	DOI: 10.1016/j.celrep.2019.02.090
GT4	Multiple_polysaccharides	DOI: 10.1016/j.celrep.2019.02.090
GT5	Starch,other_storage_CH	DOI: 10.1016/j.celrep.2019.02.090
GT51	Multiple_polysaccharides	DOI: 10.1016/j.celrep.2019.02.090
GT8	LPS_synthesis	DOI: 10.1016/j.celrep.2019.02.090
GT9	LPS_synthesis	DOI: 10.1016/j.celrep.2019.02.090
PL1	Pectins_and_mannen	DOI: 10.1016/j.celrep.2019.02.090
PL11	Pectins_and_mannen	DOI: 10.1104/pp.105.072652
PL9	Multiple_polysaccharides	DOI: 10.1104/pp.105.072652

Supplementary Table 4

ModelSEED ID	PValue	Wilcox FDR	dysbiosis	External ID	Source
rxn00062	0,000734829	0,045322493	M0	rn00230 (Purine metabolism)	KEGG
rxn00085	0,000605023	0,045322493	M0	rn01230 (Biosynthesis of amino a	KEGG
rxn00085	0,000605023	0,045322493	M0	rn01110 (Biosynthesis of seconda	KEGG
rxn00085	0,000605023	0,045322493	M0	rn00910 (Nitrogen metabolism)	KEGG
rxn00085	0,000605023	0,045322493	M0	rn00250 (Alanine, aspartate and g	KEGG
rxn00132	0,000808753	0,045322493	M0	rn00230 (Purine metabolism)	KEGG
rxn00165	0,000667073	0,045322493	M0	rn01230 (Biosynthesis of amino a	KEGG
rxn00165	0,000667073	0,045322493	M0	rn01200 (Carbon metabolism)	KEGG
rxn00165	0,000667073	0,045322493	M0	rn00260 (Glycine, serine and thre	KEGG
rxn00165	0,000667073	0,045322493	M0	rn01110 (Biosynthesis of seconda	KEGG
rxn00187	0,000605023	0,045322493	M0	rn00250 (Alanine, aspartate and g	KEGG
rxn00187	0,000605023	0,045322493	M0	rn00330 (Arginine and proline me	KEGG
rxn00187	0,000605023	0,045322493	M0	rn01230 (Biosynthesis of amino a	KEGG
rxn00187	0,000605023	0,045322493	M0	rn00630 (Glyoxylate and dicarbox	KEGG
rxn00187	0,000605023	0,045322493	M0	rn00910 (Nitrogen metabolism)	KEGG
rxn00242	0,000889334	0,048543786	M4	rn00230 (Purine metabolism)	KEGG
rxn00301	0,00054825	0,045322493	M0	rn00230 (Purine metabolism)	KEGG
rxn00363	0,000808753	0,045322493	M0	rn00240 (Pyrimidine metabolism)	KEGG
rxn00514	0,00054825	0,045322493	M0	rn00230 (Purine metabolism)	KEGG
rxn00634	7,88E-05	0,012739493	M4	rn00051 (Fructose and mannose	KEGG
rxn00692	0,000267981	0,028880153	M0	rn00670 (One carbon pool by fola	KEGG
rxn00692	0,000267981	0,028880153	M0	rn00680 (Methane metabolism)	KEGG
rxn00692	0,000267981	0,028880153	M0	rn01230 (Biosynthesis of amino a	KEGG
rxn00692	0,000267981	0,028880153	M0	rn00630 (Glyoxylate and dicarbox	KEGG
rxn00692	0,000267981	0,028880153	M0	rn00460 (Cyanoamino acid metab	KEGG
rxn00692	0,000267981	0,028880153	M0	rn01110 (Biosynthesis of seconda	KEGG
rxn00692	0,000267981	0,028880153	M0	rn01200 (Carbon metabolism)	KEGG
rxn00692	0,000267981	0,028880153	M0	rn00260 (Glycine, serine and thre	KEGG
rxn00708	0,000808753	0,045322493	M0	rn00240 (Pyrimidine metabolism)	KEGG
rxn00831	0,000808753	0,045322493	M0	rn00230 (Purine metabolism)	KEGG
rxn00863	9,94E-05	0,01347634	M4	rn01110 (Biosynthesis of seconda	KEGG
rxn00863	9,94E-05	0,01347634	M4	rn00340 (Histidine metabolism)	KEGG
rxn00872	0,000174247	0,021539972	M0	rn01212 (Fatty acid metabolism)	KEGG
rxn00872	0,000174247	0,021539972	M0	rn01200 (Carbon metabolism)	KEGG
rxn00872	0,000174247	0,021539972	M0	rn00071 (Fatty acid degradation)	KEGG
rxn00872	0,000174247	0,021539972	M0	rn00650 (Butanoate metabolism)	KEGG
rxn00913	0,000605023	0,045322493	M0	rn00230 (Purine metabolism)	KEGG
rxn01016	0,000734829	0,045322493	M0	rn00910 (Nitrogen metabolism)	KEGG
rxn01068	0,000330363	0,03386628	M4	rn00260 (Glycine, serine and thre	KEGG
rxn01114	0,000667073	0,045322493	M4	rn00040 (Pentose and glucuronat	KEGG
rxn01256	2,30E-05	0,004883533	M0	rn01230 (Biosynthesis of amino a	KEGG
rxn01256	2,30E-05	0,004883533	M0	rn00400 (Phenylalanine, tyrosine	KEGG
rxn01256	2,30E-05	0,004883533	M0	rn01110 (Biosynthesis of seconda	KEGG
rxn01292	7,84E-06	0,00269585	M4	rn00040 (Pentose and glucuronat	KEGG
rxn01344	0,000156071	0,019877829	M4	rn00710 (Carbon fixation in photo	KEGG
rxn01351	0,00054825	0,045322493	M0	rn00230 (Purine metabolism)	KEGG
rxn01364	0,000199688	0,022683463	M0	rn00400 (Phenylalanine, tyrosine	KEGG
rxn01486	0,000889334	0,048543786	M0	rn00900 (Terpenoid backbone bio	KEGG
rxn01486	0,000889334	0,048543786	M0	rn01110 (Biosynthesis of seconda	KEGG
rxn01670	6,22E-05	0,011366691	M0	rn00760 (Nicotinate and nicotina	KEGG
rxn01742	0,000199688	0,022683463	M0	rn00400 (Phenylalanine, tyrosine	KEGG
rxn01742	0,000199688	0,022683463	M0	rn01110 (Biosynthesis of seconda	KEGG
rxn01763	0,000267981	0,028880153	M4	rn00040 (Pentose and glucuronat	KEGG
rxn01961	0,000808753	0,045322493	M0	rn00230 (Purine metabolism)	KEGG
rxn01961	0,000808753	0,045322493	M0	rn01110 (Biosynthesis of seconda	KEGG
rxn01962	0,00054825	0,045322493	M0	rn00230 (Purine metabolism)	KEGG
rxn02128	8,86E-05	0,013292704	M0	rn00770 (Pantothenate and CoA	KEGG
rxn02159	9,94E-05	0,01347634	M4	rn01110 (Biosynthesis of seconda	KEGG
rxn02159	9,94E-05	0,01347634	M4	rn00340 (Histidine metabolism)	KEGG
rxn02351	0,000194338	0,022683463	M0	rn00310 (Lysine degradation)	KEGG
rxn02400	6,22E-05	0,011366691	M0	rn00760 (Nicotinate and nicotina	KEGG
rxn02450	0,000605023	0,045322493	M4	rn00564 (Glycerophospholipid me	KEGG
rxn02518	0,00054825	0,045322493	M0	rn00230 (Purine metabolism)	KEGG
rxn02972	2,32E-05	0,004883533	M0	rn00120 (Primary bile acid biosyn	KEGG
rxn02988	3,37E-05	0,006750284	M4	rn00760 (Nicotinate and nicotina	KEGG
rxn03047	8,86E-05	0,013292704	M0	rn00770 (Pantothenate and CoA	KEGG
rxn03251	9,94E-05	0,01347634	M0	rn00071 (Fatty acid degradation)	KEGG
rxn03251	9,94E-05	0,01347634	M0	rn01212 (Fatty acid metabolism)	KEGG
rxn03407	0,000605023	0,045322493	M0	rn00550 (Peptidoglycan biosynthe	KEGG
rxn03796	0,000734829	0,045322493	M4	rn00642 (Ethylbenzene degradati	KEGG
rxn03796	0,000734829	0,045322493	M4	rn01220 (Degradation of aromati	KEGG
rxn03866	0,000734829	0,045322493	M4	rn00362 (Benzoate degradation)	KEGG
rxn04786	0,000124818	0,016394075	M4	rn00260 (Glycine, serine and thre	KEGG
rxn04786	0,000124818	0,016394075	M4	rn01230 (Biosynthesis of amino a	KEGG
rxn04786	0,000124818	0,016394075	M4	rn01210 (2-Oxocarboxylic acid me	KEGG
rxn07489	0,000667073	0,045322493	M0	rn00053 (Ascorbate and aldarate	KEGG
rxn07489	0,000667073	0,045322493	M0	rn01110 (Biosynthesis of seconda	KEGG
rxn07681	0,000734829	0,045322493	M4	rn00592 (alpha-Linolenic acid me	KEGG
rxn07685	0,000734829	0,045322493	M4	rn00592 (alpha-Linolenic acid me	KEGG
rxn07689	0,000734829	0,045322493	M4	rn00592 (alpha-Linolenic acid me	KEGG
rxn07880	0,000734829	0,045322493	M4	rn00281 (Geraniol degradation)	KEGG
rxn07880	0,000734829	0,045322493	M4	rn01110 (Biosynthesis of seconda	KEGG
rxn07884	0,000734829	0,045322493	M4	rn00281 (Geraniol degradation)	KEGG
rxn07884	0,000734829	0,045322493	M4	rn01110 (Biosynthesis of seconda	KEGG
rxn13977	8,34E-06	0,00269585	M0	rn00966 (Glucosinolate biosynthe	KEGG
rxn13977	8,34E-06	0,00269585	M0	rn01210 (2-Oxocarboxylic acid me	KEGG
rxn13980	2,32E-05	0,004883533	M0	rn00120 (Primary bile acid biosyn	KEGG
rxn13983	8,34E-06	0,00269585	M0	rn00966 (Glucosinolate biosynthe	KEGG
rxn13983	8,34E-06	0,00269585	M0	rn01210 (2-Oxocarboxylic acid me	KEGG
rxn13995	8,34E-06	0,00269585	M0	rn01210 (2-Oxocarboxylic acid me	KEGG

rxn13995	8,34E-06	0,00269585	M0	rn00966 (Glucosinolate biosynthe	KEGG
rxn14049	2,32E-05	0,004883533	M0	rn00120 (Primary bile acid biosyn	KEGG
rxn14059	8,34E-06	0,00269585	M0	rn00966 (Glucosinolate biosynthe	KEGG
rxn14059	8,34E-06	0,00269585	M0	rn01210 (2-Oxocarboxylic acid me	KEGG
rxn14122	8,34E-06	0,00269585	M0	rn01210 (2-Oxocarboxylic acid me	KEGG
rxn14122	8,34E-06	0,00269585	M0	rn00966 (Glucosinolate biosynthe	KEGG
rxn14156	8,34E-06	0,00269585	M0	rn00966 (Glucosinolate biosynthe	KEGG
rxn14156	8,34E-06	0,00269585	M0	rn01210 (2-Oxocarboxylic acid me	KEGG
rxn14172	8,34E-06	0,00269585	M0	rn00966 (Glucosinolate biosynthe	KEGG
rxn14172	8,34E-06	0,00269585	M0	rn01110 (Biosynthesis of seconda	KEGG
rxn14172	8,34E-06	0,00269585	M0	rn01210 (2-Oxocarboxylic acid me	KEGG
rxn14174	2,32E-05	0,004883533	M0	rn00120 (Primary bile acid biosyn	KEGG
rxn14182	8,34E-06	0,00269585	M0	rn01210 (2-Oxocarboxylic acid me	KEGG
rxn14182	8,34E-06	0,00269585	M0	rn00966 (Glucosinolate biosynthe	KEGG
rxn14244	8,34E-06	0,00269585	M0	rn01210 (2-Oxocarboxylic acid me	KEGG
rxn14244	8,34E-06	0,00269585	M0	rn00966 (Glucosinolate biosynthe	KEGG
rxn14278	8,34E-06	0,00269585	M0	rn00966 (Glucosinolate biosynthe	KEGG
rxn14278	8,34E-06	0,00269585	M0	rn01210 (2-Oxocarboxylic acid me	KEGG
rxn14319	2,32E-05	0,004883533	M0	rn00120 (Primary bile acid biosyn	KEGG

ModelSEED ID	PValue	Wilcox FDR	dysbiosis	External ID	Source
rxn00062	0,000734829	0,045322493	M0	rn00230 (Purine metabolism	KEGG
rxn00085	0,000605023	0,045322493	M0	rn01230 (Biosynthesis of an	KEGG
rxn00085	0,000605023	0,045322493	M0	rn01110 (Biosynthesis of se	KEGG
rxn00085	0,000605023	0,045322493	M0	rn00910 (Nitrogen metabol	KEGG
rxn00085	0,000605023	0,045322493	M0	rn00250 (Alanine, aspartate	KEGG
rxn00132	0,000808753	0,045322493	M0	rn00230 (Purine metabolism	KEGG
rxn00165	0,000667073	0,045322493	M0	rn01230 (Biosynthesis of an	KEGG
rxn00165	0,000667073	0,045322493	M0	rn01200 (Carbon metabolis	KEGG
rxn00165	0,000667073	0,045322493	M0	rn00260 (Glycine, serine an	KEGG
rxn00165	0,000667073	0,045322493	M0	rn01110 (Biosynthesis of se	KEGG
rxn00187	0,000605023	0,045322493	M0	rn00250 (Alanine, aspartate	KEGG
rxn00187	0,000605023	0,045322493	M0	rn00330 (Arginine and proli	KEGG
rxn00187	0,000605023	0,045322493	M0	rn01230 (Biosynthesis of an	KEGG
rxn00187	0,000605023	0,045322493	M0	rn00630 (Glyoxylate and dic	KEGG
rxn00187	0,000605023	0,045322493	M0	rn00910 (Nitrogen metabol	KEGG
rxn00242	0,000889334	0,048543786	M4	rn00230 (Purine metabolism	KEGG
rxn00301	0,00054825	0,045322493	M0	rn00230 (Purine metabolism	KEGG
rxn00363	0,000808753	0,045322493	M0	rn00240 (Pyrimidine metab	KEGG
rxn00514	0,00054825	0,045322493	M0	rn00230 (Purine metabolism	KEGG
rxn00634	7,88E-05	0,012739493	M4	rn00051 (Fructose and man	KEGG
rxn00692	0,000267981	0,028880153	M0	rn00670 (One carbon pool b	KEGG
rxn00692	0,000267981	0,028880153	M0	rn00680 (Methane metabol	KEGG
rxn00692	0,000267981	0,028880153	M0	rn01230 (Biosynthesis of an	KEGG
rxn00692	0,000267981	0,028880153	M0	rn00630 (Glyoxylate and dic	KEGG
rxn00692	0,000267981	0,028880153	M0	rn00460 (Cyanoamino acid	KEGG
rxn00692	0,000267981	0,028880153	M0	rn01110 (Biosynthesis of se	KEGG
rxn00692	0,000267981	0,028880153	M0	rn01200 (Carbon metabolis	KEGG
rxn00692	0,000267981	0,028880153	M0	rn00260 (Glycine, serine an	KEGG
rxn00708	0,000808753	0,045322493	M0	rn00240 (Pyrimidine metab	KEGG
rxn00831	0,000808753	0,045322493	M0	rn00230 (Purine metabolism	KEGG
rxn00863	9,94E-05	0,01347634	M4	rn01110 (Biosynthesis of se	KEGG
rxn00863	9,94E-05	0,01347634	M4	rn00340 (Histidine metabol	KEGG
rxn00872	0,000174247	0,021539972	M0	rn01212 (Fatty acid metabo	KEGG
rxn00872	0,000174247	0,021539972	M0	rn01200 (Carbon metabolis	KEGG
rxn00872	0,000174247	0,021539972	M0	rn00071 (Fatty acid degrada	KEGG
rxn00872	0,000174247	0,021539972	M0	rn00650 (Butanoate metabo	KEGG
rxn00913	0,000605023	0,045322493	M0	rn00230 (Purine metabolism	KEGG
rxn01016	0,000734829	0,045322493	M0	rn00910 (Nitrogen metabol	KEGG
rxn01068	0,000330363	0,03386628	M4	rn00260 (Glycine, serine an	KEGG
rxn01114	0,000667073	0,045322493	M4	rn00040 (Pentose and glucu	KEGG
rxn01256	2,30E-05	0,004883533	M0	rn01230 (Biosynthesis of an	KEGG
rxn01256	2,30E-05	0,004883533	M0	rn00400 (Phenylalanine, tyr	KEGG
rxn01256	2,30E-05	0,004883533	M0	rn01110 (Biosynthesis of se	KEGG
rxn01292	7,84E-06	0,00269585	M4	rn00040 (Pentose and glucu	KEGG
rxn01344	0,000156071	0,019877829	M4	rn00710 (Carbon fixation in	KEGG
rxn01351	0,00054825	0,045322493	M0	rn00230 (Purine metabolism	KEGG
rxn01364	0,000199688	0,022683463	M0	rn00400 (Phenylalanine, tyr	KEGG
rxn01486	0,000889334	0,048543786	M0	rn00900 (Terpenoid backbo	KEGG
rxn01486	0,000889334	0,048543786	M0	rn01110 (Biosynthesis of se	KEGG
rxn01670	6,22E-05	0,011366691	M0	rn00760 (Nicotinate and nic	KEGG
rxn01742	0,000199688	0,022683463	M0	rn00400 (Phenylalanine, tyr	KEGG
rxn01742	0,000199688	0,022683463	M0	rn01110 (Biosynthesis of se	KEGG
rxn01763	0,000267981	0,028880153	M4	rn00040 (Pentose and glucu	KEGG
rxn01961	0,000808753	0,045322493	M0	rn00230 (Purine metabolism	KEGG
rxn01961	0,000808753	0,045322493	M0	rn01110 (Biosynthesis of se	KEGG
rxn01962	0,00054825	0,045322493	M0	rn00230 (Purine metabolism	KEGG
rxn02128	8,86E-05	0,013292704	M0	rn00770 (Pantothenate and	KEGG
rxn02159	9,94E-05	0,01347634	M4	rn01110 (Biosynthesis of se	KEGG
rxn02159	9,94E-05	0,01347634	M4	rn00340 (Histidine metabol	KEGG
rxn02351	0,000194338	0,022683463	M0	rn00310 (Lysine degradatio	KEGG
rxn02400	6,22E-05	0,011366691	M0	rn00760 (Nicotinate and nic	KEGG
rxn02450	0,000605023	0,045322493	M4	rn00564 (Glycerophospholi	KEGG
rxn02518	0,00054825	0,045322493	M0	rn00230 (Purine metabolism	KEGG
rxn02972	2,32E-05	0,004883533	M0	rn00120 (Primary bile acid b	KEGG
rxn02988	3,37E-05	0,006750284	M4	rn00760 (Nicotinate and nic	KEGG
rxn03047	8,86E-05	0,013292704	M0	rn00770 (Pantothenate and	KEGG
rxn03251	9,94E-05	0,01347634	M0	rn00071 (Fatty acid degrada	KEGG
rxn03251	9,94E-05	0,01347634	M0	rn01212 (Fatty acid metabo	KEGG
rxn03407	0,000605023	0,045322493	M0	rn00550 (Peptidoglycan bio	KEGG
rxn03796	0,000734829	0,045322493	M4	rn00642 (Ethylbenzene deg	KEGG
rxn03796	0,000734829	0,045322493	M4	rn01220 (Degradation of ar	KEGG
rxn03866	0,000734829	0,045322493	M4	rn00362 (Benzoate degrada	KEGG
rxn04786	0,000124818	0,016394075	M4	rn00260 (Glycine, serine an	KEGG
rxn04786	0,000124818	0,016394075	M4	rn01230 (Biosynthesis of an	KEGG
rxn04786	0,000124818	0,016394075	M4	rn01210 (2-Oxocarboxylic a	KEGG
rxn07489	0,000667073	0,045322493	M0	rn00053 (Ascorbate and ald	KEGG
rxn07489	0,000667073	0,045322493	M0	rn01110 (Biosynthesis of se	KEGG
rxn07681	0,000734829	0,045322493	M4	rn00592 (alpha-Linolenic ac	KEGG

rxn07685	0,000734829	0,045322493	M4	rn00592 (alpha-Linolenic ac	KEGG
rxn07689	0,000734829	0,045322493	M4	rn00592 (alpha-Linolenic ac	KEGG
rxn07880	0,000734829	0,045322493	M4	rn00281 (Geraniol degradat	KEGG
rxn07880	0,000734829	0,045322493	M4	rn01110 (Biosynthesis of se	KEGG
rxn07884	0,000734829	0,045322493	M4	rn00281 (Geraniol degradat	KEGG
rxn07884	0,000734829	0,045322493	M4	rn01110 (Biosynthesis of se	KEGG
rxn13977	8,34E-06	0,00269585	M0	rn00966 (Glucosinolate bios	KEGG
rxn13977	8,34E-06	0,00269585	M0	rn01210 (2-Oxocarboxylic a	KEGG
rxn13980	2,32E-05	0,004883533	M0	rn00120 (Primary bile acid b	KEGG
rxn13983	8,34E-06	0,00269585	M0	rn00966 (Glucosinolate bios	KEGG
rxn13983	8,34E-06	0,00269585	M0	rn01210 (2-Oxocarboxylic a	KEGG
rxn13995	8,34E-06	0,00269585	M0	rn01210 (2-Oxocarboxylic a	KEGG
rxn13995	8,34E-06	0,00269585	M0	rn00966 (Glucosinolate bios	KEGG
rxn14049	2,32E-05	0,004883533	M0	rn00120 (Primary bile acid b	KEGG
rxn14059	8,34E-06	0,00269585	M0	rn00966 (Glucosinolate bios	KEGG
rxn14059	8,34E-06	0,00269585	M0	rn01210 (2-Oxocarboxylic a	KEGG
rxn14122	8,34E-06	0,00269585	M0	rn01210 (2-Oxocarboxylic a	KEGG
rxn14122	8,34E-06	0,00269585	M0	rn00966 (Glucosinolate bios	KEGG
rxn14156	8,34E-06	0,00269585	M0	rn00966 (Glucosinolate bios	KEGG
rxn14156	8,34E-06	0,00269585	M0	rn01210 (2-Oxocarboxylic a	KEGG
rxn14172	8,34E-06	0,00269585	M0	rn00966 (Glucosinolate bios	KEGG
rxn14172	8,34E-06	0,00269585	M0	rn01110 (Biosynthesis of se	KEGG
rxn14172	8,34E-06	0,00269585	M0	rn01210 (2-Oxocarboxylic a	KEGG
rxn14174	2,32E-05	0,004883533	M0	rn00120 (Primary bile acid b	KEGG
rxn14182	8,34E-06	0,00269585	M0	rn01210 (2-Oxocarboxylic a	KEGG
rxn14182	8,34E-06	0,00269585	M0	rn00966 (Glucosinolate bios	KEGG
rxn14244	8,34E-06	0,00269585	M0	rn01210 (2-Oxocarboxylic a	KEGG
rxn14244	8,34E-06	0,00269585	M0	rn00966 (Glucosinolate bios	KEGG
rxn14278	8,34E-06	0,00269585	M0	rn00966 (Glucosinolate bios	KEGG
rxn14278	8,34E-06	0,00269585	M0	rn01210 (2-Oxocarboxylic a	KEGG
rxn14319	2,32E-05	0,004883533	M0	rn00120 (Primary bile acid b	KEGG

Supplementary Table 5

This document contains all information in order to build the diets.
The first sheet Diet_meals consist of the five diferent diets: high fibre omnivorous, high fibre plant based, ketogenic diet, high protein plant based and high protein omnivorous. Of each diet the products consumed for 3 days are shown. All products contain a food ID number whih is linked to the USDA database.
The second sheet named Diet_per_day . This sheet contains all diets from the second sheet (Diet_meals). However, an average consumption of each diet is calculated over 3 days.
The sheet named Diets_macros contains the macro nutrient information for eeach diet, normalized over a 2000Kcal intake.
The sheet named Diets_micronutrients_normalized contains the flux rate (mmol/gDW) of each diet normalized on a 2000kCal intake

Diet plan		High protein meals/recipes	Food name	Food ID	Food name
Day 1					
[illegible]	Breakfast	[illegible]			
		ham		[illegible]	Ham, sliced, regular [illegible]
		cheese		[illegible]	Cheese, [illegible]
		white bread		[illegible]	Bread, white, commercially prepared, toasted
		butter		[illegible]	Butter, salted
		coffee		[illegible]	Beverages, coffee, brewed, breakfast blend
		dairy milk		[illegible]	Milk, whole, 3.25% milkfat, without added vitamin A and vitamin D
	Lunch	chicken curry and white rice			
		white rice		[illegible]	Rice, white, long-grain, regular, enriched, cooked
		garlic		[illegible]	Garlic, raw
		[illegible]		[illegible]	Onions, [illegible]
		[illegible]		[illegible]	Oil, [illegible]
		salt		[illegible]	Salt, table
		chicken		[illegible]	Chicken, [illegible]
		cooking cream		[illegible]	Cream, fluid, half and half
	Dinner	[illegible]			
		fish		[illegible]	Fish, [illegible]
		[illegible]		[illegible]	[illegible]

[illegible]

HIGH FIBRE PLANT BASED		
Ingredient / food	USDA ID	g/per 1 day
chilli powder	2009	1,666666667
cumin	2014	1,666666667
curry	2015	1,666666667
dill	2016	1,666666667
ginger	2021	1,666666667
paprika powder	2028	1,666666667
pepper	2030	1,666666667
salt	2047	1,666666667
apple cider vinegar	2048	5
rice wine vinegar	2069	3,333333333
olive oil	4053	45
avocado	9037	85
banana	9040	36
lemon juice	9152	1,666666667
lime	9160	1,666666667
mango	9176	35
orange juice w/ fibre	9209	83,33333333
pear	9252	41,66666667
raspberries	9302	40
strawberries	9316	33,33333333
broccoli	11091	65
cabbage	11109	66,66666667
carrot	11124	26,66666667
celery	11143	66,66666667
coriander	11165	8,333333333
cucumber	11205	43,33333333
garlic	11215	5
kale	11233	40
shiitake mushroom	11238	60
mushrooms	11243	60
lettuce	11252	40
mushrooms	11260	50
onion	11282	20
red onion	11286	20
spring onion	11291	20
parsley	11297	8,333333333
peas	11302	25
spinach	11457	43,33333333
courgette	11477	50
sweet potato	11508	66,66666667
tomato	11529	26,66666667
chopped tomatoes	11530	26,66666667
watercress	11591	13,33333333
artichoke	11702	23,33333333
red bellpepper	11951	100
arugula	11959	66,66666667
sesame seeds	12023	23,33333333
soaked raw cashew	12087	5
coconut milk	12118	45
Nuts, macadamia nuts, rav	12131	33,33333333
walnuts	12155	45
flax seeds	12220	45
red wine	14097	40
cooked white wine	14140	11,66666667
black tea	14355	50
lentils	16070	50
soy beens	16111	5
soy sauce	16123	33,33333333
tofu	16129	66,66666667
soy milk	16235	5
chickpeas	16359	66,66666667
whole grain bread	18076	200
whole grain crackers	18235	125
golden caster sugar	19335	30
maple sypup	19353	15
appricot jam	19719	5
oats	20034	40,33333333
brown rice	20041	5
wholegrain flour	20129	15
oats flour	20132	33,33333333
Quinoa, cooked	20137	15
margarine	42307	3
nutritional yeast	43406	21

HIGH FIBRE OMNIVOROUS		
Ingredient / food	USDA ID	g/per 1 day
Butter. salted	1001	15
Cheese. brie	1006	6,67
Cheese. camembert	1007	6,67
Cheese. edam	1018	6,67
Cheese. parmesan. grated	1032	6,67
Cream. fluid. half and half	1049	11,66666667
Egg. whole. raw. fresh	1123	10
Egg. whole. cooked. hard-bo	1129	10
Egg. whole. cooked. scrambl	1132	10
Milk. whole. 3.25% milkfat. v	1211	20
Spices. chili powder	2009	1,666666667
Spices. cumin seed	2014	1,666666667
Spices. curry powder	2015	1,666666667
Spices. dill seed	2016	1,666666667
Spices. ginger. ground	2021	1,666666667
Spices. paprika	2028	1,666666667
Spices. pepper. black	2030	1,666666667
Salt. table	2047	1,666666667
Vinegar. balsamic	2069	3,333333333
Oil. olive. salad or cooking	4053	10
Oil. sunflower. high oleic (70	4584	10
Chicken. broilers or fryers. m	5010	15
Chicken. broilers or fryers. b	5064	15
Sausage. pork. chorizo. link c	7019	16,66666667
Ham. sliced. regular (approxi	7029	16,66666667
Sausage. smoked link sausag	7074	20
Salami. Italian. pork	7926	20
Salami. Italian. pork and bee	7941	16,66666667
Avocados. raw. all commerci	9037	12
Bananas. raw	9040	95
Lemon juice. raw	9152	1,666666667
Lime juice. raw	9160	1,666666667
Mangos. raw	9176	41,66666667
Orange juice. chilled. include	9209	83,33333333
Pears. raw	9252	65
Raspberries. raw	9302	65
Rose-apples. raw	9312	65
Strawberries. raw	9316	40
Cabbage. raw	11109	88
Carrots. raw	11124	88
Coriander (cilantro) leaves. r	11165	8,333333333
Cucumber. with peel. raw	11205	43,33333333
Garlic. raw	11215	5
Kale. raw	11233	45
Lettuce. iceberg (includes cr	11252	40
Onions. raw	11282	20
Onions. yellow. sauteed	11286	20
Onions. spring or scallions (i	11291	20
Parsley. fresh	11297	8,333333333
Peas. edible-podded. frozen	11302	35
Spinach. raw	11457	43,33333333
Sweet potato. cooked. boile	11510	125
Tomatoes. red. ripe. raw. ye	11529	26,66666667
Tomatoes. red. ripe. cooked	11530	26,66666667
Watercress. raw	11591	13,33333333
Artichokes. (globe or french)	11702	23,33333333
Arugula. raw	11959	35
Seeds. sesame seeds. whole	12023	15
Nuts. macadamia nuts. raw	12131	10
Nuts. walnuts. english	12155	25
Seeds. flaxseed	12220	56
Alcoholic Beverage. wine. ta	14097	10
Alcoholic beverage. wine. ta	14140	50
Beverages. tea. black. brewe	14355	50
Fish. salmon. chinook. smok	15077	45
Fish. salmon. pink. raw	15083	15
Fish. tuna. fresh. skipjack. ra	15123	15
Fish. pollock. Alaska. cooked	15267	10
Crustaceans. shrimp. cookec	15271	10
Soy sauce made from soy an	16123	40
Bread. whole-wheat. comme	18076	55
Crackers. whole-wheat	18235	115
Leavening agents. baking po	18371	15
Sugars. granulated	19335	1,666666667
Syrups. maple	19353	5
Rice. brown. medium-grain.	20041	25
Wheat flours. bread. unenric	20129	65
Oat flour. partially debranne	20132	16,66666667
Quinoa. cooked	20137	35
Beef. chuck for stew. separa	23092	35
Margarine-like. butter-marga	42307	85

HIGH PROTEIN PLANT BASED		
Ingredient / food	USDA ID	g/per 1 day
chilli powder	2009	1,5
cumin	2014	1,5
curry	2015	1,5
dill	2016	10
ginger	2021	4
paprika powder	2028	4
pepper	2030	4
salt	2047	4
apple cider vinegar	2048	12
balsamic vinegar	2069	8
olive oil	4053	20
sunflower oil	4584	20
avocado	9037	30
banana	9040	20
lemon juice	9152	11
lime	9160	7,5
orange juice	9209	30
peach	9236	0
apple	9312	25
strawberries	9316	0
Beans snap green cooked bo	11053	70
broccoli	11091	70
brussel sprouts	11098	50
carrots	11124	15
celery	11143	15
coriander	11165	10
cucumber	11205	30
garlic	11215	3,75
pickled ginger	11216	18,75
mushrooms	11243	120
lettuce	11252	31,25
mushrooms	11260	120
onion	11282	20
red onion	11286	11,25
spring onions	11291	6,25
parsley	11297	11,25
peas	11302	100
spinach	11457	12,5
courgette	11477	12,5
tomatoes	11530	20
Beans kidney mature seeds	11713	70
potato	11829	10
red bellpepper	11951	17,5
arugula	11959	20
wasabi	11990	2,5
sesame seeds	12023	10
coconut milk	12118	37,5
Nuts. macadamia nuts. raw	12131	25
Nuts. walnuts. english	12155	20
almond milk	14016	40
red wine	14097	20
cooked white wine	14140	7,5
coffee	14215	62,5
black tea	14355	62,5
chickpeas	16057	70
lentils	16070	70
soy beens	16111	150
soy sauce	16123	3,75
tofu	16129	120
chickpeas	16359	70
white bread	18070	15
non-whole grain crackers	18232	11,25
sugar	19334	0
golden caster sugar	19335	0
maple sypup	19353	0
white rice	20045	10
non-wholegrain flour	20129	21,25

HIGH PROTEIN OMNIVOROUS		
Ingredient / food	USDA ID	g/per 1 day
butter	1001	2
cheese 1	1006	10
cheese 2	1007	10
cheese	1018	10
parmesan chesse	1032	5
cooking cream	1049	10
eggs	1123	50
boiled egg	1129	25
chilli powder	2009	2
cumin	2014	2
curry	2015	2
dill	2016	2
ginger	2021	5
paprika powder	2028	2
pepper	2030	2
salt	2047	2
rice wine vinegar	2069	2
olive oil	4053	15
sunflower oil	4584	15
chicken	5064	45
sausage	7004	15
chorizo	7019	10
ham	7029	10
prosciutto	7941	10
lemon juice	9152	5
lime	9160	2
orange juice	9209	55
strawberries	9316	55
coriander	11165	5
cucumber	11205	45
garlic	11215	5
lettuce	11252	55
onion	11282	25
red onion	11286	10
parsley	11297	5
peas	11302	0
spinach	11457	30
chopped tomatoes	11530	25
potato	11829	20
wasabi	11990	4
sesame seeds	12023	5
beef	13020	15
red wine	14097	35
cooked white wine	14140	5
black tea	14355	85
smoked salmon	15077	15
salmon	15083	95
tuna	15123	95
fish	15267	85
shrimp	15271	95
soy sauce	16123	5
white bread	18070	30
non-whole grain crackers	18232	10
baking powder	18371	1,5
sugar	19335	5
maple sypup	19353	5
white rice	20045	30
non-wholegrain flour	20083	10
muesli	42184	20
margarine	42307	10

KETO == HIGH FAT LOW CARB		
Ingredient / food	USDA ID	g/per 1 day
Cheese, parmesan, hard	1033	7,5
Cream, fluid, light whipping	1052	15
Cream, sour, cultured	1056	12,5
Egg, whole, cooked, scrambled	1132	40
Butter, without salt	1145	12,5
Spices, cinnamon, ground	2010	0,75
Salad dressing, mayonnaise, reg	4025	5
Chicken, broilers or fryers, brea	5057	125
Bacon and beef sticks	7921	30
olives	9195	5
Orange juice, raw (Includes foo	9206	75
Rose-apples, raw	9312	25
Asparagus, raw	11011	20
Celery, raw	11143	25
Cucumber, peeled, raw	11206	25
Lettuce, green leaf, raw	11253	30
Squash, summer, zucchini, inclu	11477	25
Catsup	11935	5
Nuts, macadamia nuts, raw	12131	7,5
Fish, tuna, white, canned in wa	15126	112
avocados, raw, california	9038	25
cheese edam	1018	7,5
Luncheon sausage, pork and be	7090	32,5
salami, italian, pork	7926	12,5
salmon	15076	125

Macronutrients	HIGH FIBRE PLANT BASED	HIGH FIBRE OMNIVOROUS	HIGH PROTEIN PLANT BASED	HIGH PROTEIN OMNIVOROUS	KETO == HIGH FAT LOW CARB
Protein	134,14	140,2235	138,12275	140,0306	83,393075
Total lipid (fat)	211.14	231.8952783	160.84365	96.2718	196.034825
Carbohydrate by difference	467.14	356.80868	242.6602	106.9224	26.7641
Ash	42,34	44,42805433	28,393475	19,0251	14,9632
Energy Kcal	4115.88	3962.7674	2793.0675	1860.065	2179.8775
Water	1638.17	1468.972554	1333.550675	896.2578	485.45615
Adjusted Protein	1,09	0	2,616	0	9120,9575
Energy	17223.87	16584.1682	11690.4925	7784.98	11.21655
Sugars total	112,62	72,08784133	38,09425	31,4671	10,071
Fiber total dietary	106,38	73,00933333	65,27	12,659	0,367835
Fatty acids total trans	2.30	1.54385	0.030825	0.14701	48.732085
Fatty acids total saturated	35,90	61,54609807	27,57019	27,499405	74,4873275
Fatty acids total monounsaturated	88.17	88.55361453	69.1425125	48.00771	64.6235875
Fatty acids total polyunsaturated	70.36	66.38235293	53.448845	13.021285	0.069865
Fatty acids total trans-monoenoic	0,22	0,8259	0,027	0,11364	0,18847
Fatty acids total trans-polyenoic	0.03	0.0918	0.003825	0.03337	

Based on 2000 kCAL	2000	2000	2000	2000	2000
	2,05794	1,9813837	1,39653375	0,9300325	1,08993875

Macronutrients	HIGH FIBRE PLANT BASED		HIGH FIBRE OMNIVOROUS		HIGH PROTEIN PLANT BASED		HIGH PROTEIN OMNIVOROUS		KETO == HIGH FAT LOW CARB	
Protein	65,18155696		70,77049236		98,90398281		150,5652759		76,51170765	
Total lipid (fat)	102.5953138		117.0370375		115.1734786		103.5144471		179.85857	
Carbohydrate by difference	226,9937737		180,0805568		173,7589228		114,9663049		24,55560003	
Ash	20,57275722		22,42274141		20,33139192		20,4563819		13,72847786	
Energy Kcal	2000		2000		2000		2000		2000	
Water	796,0230943		741,3872204		954,9004276		963,6843874		445,3976428	
Adjusted Protein	0,529655869		0		1,873209294		0		8368,321156	
Energy	8369.471413		8369.993253		8371.077677		8370.653714		10.29099112	
Sugars total	54,72509727		36,38257513		27,27771527		33,83440901		9,23996876	
Fiber total dietary	51.69230719		36.84765012		46.73714473		13.61135229		0.337482267	
Fatty acids total trans	1.117015073		0.779177703		0.022072506		0.158069745		44.71084728	
Fatty acids total saturated	17,44642701		31,06218047		19,74187162		29,56821939		68,34083796	
Fatty acids total monounsaturated	42.84335306		44.69281469		49.51009061		51.61938964		59.29102667	
Fatty acids total polyunsaturated	34.18788044		33.50302767		38.27250505		14.00089244		0.064099932	
Fatty acids total trans-monoenoic	0,105688212		0,416829915		0,019333582		0,122189278		0,172917974	
Fatty acids total trans-polyenoic	0.015185088		0.046331258		0.002738924		0.035880467		0	
macro %s										
total grams = 100%	394.77		367.89		387.84		369.05		280.93	
protein %	16,5112472	20%	19,23696225	20%	25,50147094	40%	40,79850873	40%	27,23554992	25%
fat %	25,98858735	30%	31,81321758	30%	29,69640893	30%	28,04919691	30%	64,02349669	70%
carbs %	57.50016545	50%	48.94982017	50%	44.80212013	30%	31.15229436	30%	8.740953389	5%
fibre grams	51,69	37 g	36,85	37 g	46,74		13,61		0,34	
check = 100%	100		100		100		100		100	

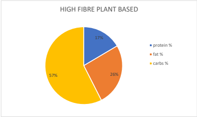

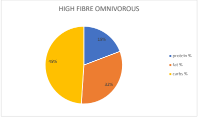

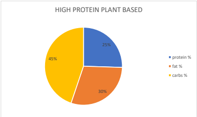

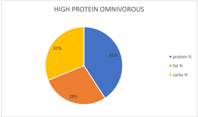

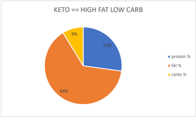

Micronutrients mmol/gDW	iH FIBRE PLANT BA	HIGH FIBRE PLANT BASED	HIGH FIBRE OMNIVOROUS	HIGH FIBRE OMNIVOROUS	HIGH PROTEIN PLANT BASED	HIGH PROTEIN PLANT BASED	HIGH PROTEIN OMNIVOROUS	HIGH PROTEIN OMNIVOROUS	KETO == HIGH FAT LOW CARB	ETO == HIGH FAT LOW CARB
	**normalized**	**1,9724842**	**normalized**	**1,9813837**	**normalized**	**1,4246838**	**normalized**	**0,9300325**		**1,08993875**
Starch	57,40392638	113,2283378	58,47542529	115,8622545	7,413331234	10,56	7,599228831	7,067529788	0,461115322	0,502587458
Sucrose	79,82552593	157,4545887	33,04716602	65,47911609	11,36579476	16,19	36,70927546	34,14081923	6,166862209	6,721502087
Glucose (dextrose)	48,1503658	94,97583576	48,79107281	96,67383638	38,41890203	54,73	29,51281649	27,44787851	9,418717496	10,26582517
Fructose	43,88430158	86,5610915	56,27990564	111,5120877	22,68138474	32,31	29,93537456	27,84087124	9,98604569	10,88417816
Lactose	0	0	1,667603685	3,304162759	0	0,00	0,023559173	0,021910797	3,4596881	3,770848123
Maltose	3,599953305	7,100851015	6,203927648	12,29236112	0,74385084	1,06	1,036603622	0,964075058	0,120616668	0,131464781
Alcohol, ethyl	57,51782899	113,4530089	59,70615871	118,3008097	42,05153235	59,91	96,04238339	89,32253793	0	0
Water	46101,02449	90933,54242	41153,88401	81541,63497	51958,52171	74024,46	53493,44365	49750,64113	24723,71039	26947,33
Caffeine	0,026106621	0,051494897	0,025989361	0,051494897	0,103916275	0,15	0,094127167	0,087541325	0	0
Theobromine	0,002813917	0,005550406	0,002801278	0,005550406	0,004869858	0,01	0,010145549	0,00943569	0	0
Galactose	1,634047332	3,223132544	0,217593194	0,431135608	0,646172207	0,92	0,346163392	0,321943205	0,249542922	0,271986501
Calcium, Ca	21,03131658	41,48393965	24,16882318	47,88771229	26,08432171	37,16	19,37444467	18,01886322	11,41817101	12,44510704
Iron, Fe	0,380463132	0,750457516	0,273094176	0,541104348	0,452939126	0,65	0,314882273	0,292850748	0,125088657	0,136338974
Magnesium, Mg	27,0041752	53,26530892	19,71530206	39,06357814	25,34854471	36,11	14,73405231	13,7031475	6,450313295	7,03044641
Phosphorus, P	51,07039668	100,7355505	57,43673935	113,8042191	57,9350686	82,54	61,79107793	57,46771069	26,8367407	29,25040362
Potassium, K	97,54582895	192,4076064	92,68676035	183,6480362	115,5211428	164,58	88,85304986	82,6362241	39,13583806	42,65566641
Sodium, Na	122,8158118	242,2522483	136,886879	271,2254308	77,76358209	110,79	139,4880927	129,7284595	147,4666867	160,7296562
Zinc, Zn	0,191338958	0,377413072	0,168104299	0,333079117	0,211745672	0,30	0,169343165	0,157494647	0,096190095	0,104841312
Copper, Cu	0,060905707	0,120135545	0,038670231	0,076620566	0,061039652	0,09	0,02490438	0,023161883	0,010036998	0,010939713
Fluoride, F	0,00545584	0,010761559	0,005526587	0,01095029	0,009079757	0,01	0,018990939	0,017662191	0,000590687	0,000643813
Manganese, Mn	0,201365254	0,397189783	0,123129841	0,24396746	0,174969952	0,25	0,093306743	0,086778304	0,045737283	0,049850837
Selenium, Se	0,001425343	0,002811466	0,001148872	0,002276356	0,001226103	0,00	0,002027987	1,89E-03	0,001542495	0,001681225
Retinol	4,07762E-05	8,04E-05	0,001596229	0,003162742	6,17475E-05	0,00	0,001430919	0,001330801	0,000679026	0,000740097
Vitamin A, RAE	0,002970814	0,005859885	0,005957407	0,01180391	0,001410453	0,00	0,002518316	0,002342115	0,002115493	0,002305758
Carotene, beta	0,018094716	0,035691541	0,026126133	0,051765893	0,00786103	0,01	0,006663619	0,006197382	0,009026521	0,009838355
Carotene, alpha	0,001064067	2,10E-03	0,003048039	6,04E-03	0,000788251	0,00	0,000134762	0,000125333	1,57218E-05	1,71358E-05
Vitamin D (D2 + D3)	7,74303E-07	1,53E-06	1,46793E-05	2,90854E-05	1,82008E-06	0,00	4,20587E-05	3,91E-05	8,21671E-06	8,95571E-06
Cryptoxanthin, beta	0,000250727	0,000494556	0,000237546	0,000470669	0,000438917	0,00	0,0004626	0,000430233	0,000105499	0,000114987
Lycopene	0,001415545	0,00279214	0,001409952	0,002793655	0,000796795	0,00	0,001524203	1,42E-03	0,001835092	0,002000138
Lutein + zeaxanthin	0,00724668	0,014293962	0,006070256	1,20E-02	0,003975225	0,01	0,004698011	0,004369302	0,002379986	0,002594039
Tocopherol, beta	0,000608944	0,001201133	0,000492315	0,000975465	0,000230944	0,00	0,000231721	2,16E-04	0,002062569	0,002248074
Tocopherol, gamma	0,038109441	0,07517027	0,034529006	0,068415209	0,011739295	0,02	0,006756031	0,006283328	0,100933121	0,110010919
Tocopherol, delta	0,005440083	0,010730479	0,005141739	0,010187758	0,001526581	0,00	0,001220327	0,001134944	0,029269295	0,031901739
Tocotrienol, alpha	0,000808927	1,60E-03	0,000767382	1,52E-03	0,000640063	0,00	0,000645642	0,000600468	0,000264657	0,00028846
Tocotrienol, beta	1,0494E-05	2,07E-05	5,12102E-06	1,01E-05	0	0,00	0	0	0	0
Tocotrienol, gamma	7,28409E-05	0,000143677	6,26813E-05	0,000124196	8,3072E-05	0,00	0,000172292	1,60E-04	2,79283E-06	3,04401E-06
Tocotrienol, delta	0	0	0	0	0	0,00	0	0	0	0
Vitamin C, total ascorbic acid	1,386362966	2,734579047	0,820930084	1,626577487	1,02291159	1,46	0,538555786	0,500874384	0,202363071	0,220563353
Thiamin	0,016679469	0,032899989	0,00600924	0,011906611	0,006891895	0,01	0,004839523	0,004500914	0,004708818	0,005132323
Riboflavin	0,00873743	0,017234443	0,002832739	0,005612744	0,006296577	0,01	0,004685412	0,004357585	0,00228044	0,002485539
Niacin	0,275321242	0,543066799	0,142130154	0,281614371	0,145872249	0,21	0,344070155	0,319996426	0,142277346	0,155073592
Pantothenic acid	0,028251288	0,05572522	0,019571124	0,038777907	0,027147629	0,04	0,028296419	0,026316589	0,013475158	0,014687097
Vitamin B-6	0,01212025	2,39E-02	0,011491932	2,28E-02	0,010674496	0,02	0,018700876	0,017392422	0,008045314	0,0087689
Vitamin B-12	7,08814E-08	1,40E-07	2,17097E-06	4,30152E-06	2,97579E-07	0,00	7,49265E-06	6,97E-06	3,29236E-06	3,58847E-06
Choline, total	2,178905712	4,30E+00	2,426535048	4,81E+00	2,394715156	3,41	4,630338588	4,306365373	1,517347877	1,653816248
Menaquinone-4	2,05226E-07	4,05E-07	3,35967E-06	6,66E-06	5,68273E-07	0,00	0	0,00E+00	5,15835E-08	5,62229E-08
Dihydrophylloquinone	6,00791E-06	1,19E-05	7,07901E-07	1,40262E-06	1,50329E-05	0,00	4,03755E-07	3,76E-07	2,53323E-08	2,76107E-08
Vitamin K (phylloquinone)	0,001268383	0,002501866	0,001166982	0,00231224	0,000997355	0,00	0,000718977	6,69E-04	0,000756028	0,000824024
Betaine	0,449019094	0,885683068	0,326069499	0,64606879	0,282969292	0,40	0,39916863	0,371239799	0,028617492	0,031191314
Tryptophan	3,31542313	6,539619741	3,802101676	7,533422286	6,13560056	8,74	5,921303172	5,507004392	3,884349779	4,233703343
Threonine	15,34734153	30,27238868	21,25583584	42,11596667	32,36974974	46,12	38,04122803	35,3795784	25,8952283	28,22421276
Isoleucine	15,48588214	30,54565784	21,39125583	42,38428562	32,86399023	46,82	38,2564284	35,57972175	25,79906583	28,11940156
Leucine	26,28860568	51,85385935	35,22413697	69,79253084	54,07098772	77,03	62,31308293	57,9531923	43,97938779	47,93483896
Lysine	14,82156346	29,23529975	26,80573357	53,11244357	38,82309138	55,31	57,04047752	53,04949791	41,06547734	44,75885504
Methionine	5,081131335	10,02245128	9,236432733	18,30091726	8,260893449	11,77	18,71623832	17,40670992	12,66081245	13,79951009
Cystine	3,402703275	6,711778447	3,781186843	7,491981978	5,586418177	7,96	5,152198177	4,791711751	3,32357068	3,622488472
Phenylalanine	14,46173688	28,5255475	16,85547064	33,39715477	28,32724342	40,36	27,24649467	25,34012555	18,62293252	20,29785579
Tyrosine	7,764684029	15,31571656	10,84399597	21,48611686	17,04265964	24,28	20,73732735	19,2863884	14,39535855	15,69005911
Valine	21,83038487	43,06008923	26,73632216	52,97491293	43,42919328	61,87	47,2481103	43,94227814	32,37130585	35,28274064
Arginine	22,08764074	43,56752237	23,64614905	46,85209429	41,2664541	58,79	35,70920206	33,21071847	26,00902483	28,34824401
Histidine	7,928917205	15,63966391	10,98188106	21,75932013	15,91433144	22,67	18,95190611	17,62588862	13,85837595	15,10478096
Alanine	24,50854824	48,34272416	34,83801294	69,02747099	47,19044728	67,23	64,56263127	60,04534537	46,00736674	50,1452118
Aspartic acid	36,31907725	71,63880604	42,72231378	84,64929616	80,85186035	115,19	78,35294388	72,87078428	52,419378	57,13391133
Glutamic acid	75,49885431	148,9202972	84,74316444	167,9087247	117,9453806	168,03	116,0615404	107,9410046	79,80690606	86,98463943
Glycine	30,29062584	59,74778087	40,36222546	79,97305563	53,24748808	75,86	66,34271442	61,70088055	50,31109607	54,83601316
Proline	27,13362903	53,52065455	33,14811116	65,67912715	43,69939747	62,26	47,22418005	43,92002224	32,57891732	35,50902442
Serine	23,01369155	45,39414297	27,1002707	53,69603462	46,07200028	65,64	45,38780733	42,21213592	30,17702231	32,89110597
Hydroxyproline	0,304460991	0,600544494	0,759754339	1,505364864	0	0,00	0,137754496	0,128116159	0	0
Vitamin B-12, added	0	0	0	0	0,000582082	0,00	0,001142354	0,001062426	0	0
Butyric acid	0,146500593	0,288970104	8,584446818	17,009083	0,002628996	0,00	5,734094742	5,332894468	7,328896681	7,988048487
caproic acid	0,309527037	0,61053719	3,814507964	7,558003903	0,288232736	0,41	2,311339886	2,149621212	2,552992898	2,782605888
caprylic acid	2,469651517	4,871348598	1,838247909	3,642274444	2,760339314	3,93	1,187336703	1,104261722	1,219109321	1,32875449
capric acid	1,739652104	3,431436289	3,431817909	6,799748067	2,101843386	2,99	3,025687283	2,813987508	2,506063959	2,731456219
lauric acid	10,95848194	21,61543249	3,132933828	6,207544021	12,57914026	17,92	2,149018622	1,998657162	2,306405032	2,513840217
myristic acid	4,711884285	9,294117304	11,28291324	22,35578038	5,503596996	7,84	11,19116021	10,40814271	11,19532958	12,20222353
palmitic acid	39,55349988	78,01865356	63,57011767	125,956795	39,79828969	56,70	63,76163615	59,30039387	97,30194716	106,0531627
stearic acid	11,85479741	23,38340059	26,73294455	52,96822059	12,7532221	18,17	23,79068747	22,12611254	35,95430913	39,18799476
arachidic acid	1,593924567	3,143991024	0,609448378	1,207551082	1,344679509	1,92	0,297759837	0,276926326	2,741281269	2,98782868
Oleic acid	148,0227473	291,9725302	138,6474018	274,713702	159,7867912	227,65	165,5823871	153,9970014	188,9489878	205,9428236
linoleic acid	95,4766533	188,3261901	83,57926913	165,6026015	116,8318628	166,45	39,11540294	36,37859598	175,3006525	191,066974
α-Linolenic acid	32,54440643	64,19332749	32,91903993	65,22524913	17,02605944	24,26	4,416933752	4,107891939	25,00491915	27,25383032
Arachidonic acid	0,007631637	0,015053283	0,233777641	0,463203207	0,002074788	0,00	0,767734756	0,714018274	0,617131403	0,67263543
Docosahexaenoic	0,003472558	0,006849565	0,359293433	0,711898152	0	0,00	2,110934427	1,963237622	2,634542517	2,871489978
Docosanoic acid	0,563525425	1,111544996	0,3930362	0,77875552	0,819766242	1,17	0,590475559	0,54916146	1,352147043	1,473757458
Myristoleic acid	0,005449898	0,010749838	0,081513585	0,16150969	0,011132079	0,02	0,106259599	0,098824881	0,234781711	0,255897685
Palmitoleic acid	11,34802082	22,38379177	6,756673394	13,38756253	10,89996977	15,53	8,045828755	7,482882231	23,6760723	25,80546865
Stearidonic acid	0	0	0,020997123	0,041603357	0	0,00	0,284153253	0,26427176	0,24603288	0,26816077
Paullinic acid	1,5780687	3,112715578	1,395813248	2,765641619	1,772095871	2,52	2,798311797	2,602520916	3,249815714	3,542100077
Eicosapentaenoic acid (EPA)	0	0	0,216507879	0,428985181	0,000261077	0,00	1,105064609	1,027746001	1,014300478	1,105525395
docosenoic acid	0,134264538	0,26483468	0,276858144	0,548562214	0,124496302	0,18	0,312747427	0,290865271	0,5232678	0,570329852
Docosapentaenoic acid (DPA)	0,001748141	0,003448181	0,068358561	0,135444539	0,000435656	0,00	0,242921406	0,225924802	0,156916859	0,171029765
Phytosterols	0,44207304	0,871982086	0,255558853	0,506360145	0,349484298	0,50	0,290224647	0,269918354	0,198299968	0,216134819
Stigmasterol	0,008476157	0,016719085	0,008132358	0,016113321	0,001530691	0,00	0,000390802	0,000363458	0,109265952	0,119093196
Campesterol	0,0405724	0,080028418	0,035078986	0,06950493	0,01208707	0,02	0	0	0,119868482	0,130649303
Beta-sitosterol	0,227458494	0,448658285	0,112979089	0,223854925	0,114074491	0,16	0,000466682	0,00043403	0,393414217	0,4287974
pentadecanoic acid	0,022936287	0,045241464	0,233177424	0,462013946	0,47256009	0,67	0,679551929	0,632005379	0,318597997	0,347252303
heptadecanoic acid	0,15973967	0,315083975	0,352260307	0,697962831	0,100268431	0,14	0,186774104	0,173705987	0,615116486	0,670439293
tetracosanoic acid	0,181897759	0,358790457	0,090303074	0,178925039	0,140612038	0,20	0,010791805	0,010036729	0,49141173	0,535608687
Palmitoleic acid	0	0	0,002281331	0,004520191	0	0,00	0,030429398	0,028300329	0,013523471	0,014739755
Oleic acid	0,390370035	0,769998726	1,473348515	2,92E+00	0,067092819	0,10	0,401553959	0,373458232	0,178206703	0,194234391
Erucic acid	0	0	0,000223597	0,000443032	0	0,00	0,003016958	2,81E-03	0,030485566	0,033227399
linoleic acid	0,056490829	0,111427267	0,059026457	0,11695406	0,00957314	0,01	0,105241174	0,097877712	0,60930627	0,664106514
linoleic acid	0	0	0,079901667	0,158315862	0	0,00	0,022696821	0,021108782	0,007262604	0,007915793
linoleic acid	0	0	0,026273972	0,052058819	0	0,00	0	0	0	0
linoleic acid	0,015817432	0,031199635	0,107075433	0,212157517	0,002252504	0,00	0,064754952	0,060224209	0,235552079	0,256737338
Nervonic acid	0,049849836	0,098328015	0,060418369	0,119711971	0,009261355	0,01	0,095607393	0,088917983	0,014639484	0,015956141
eicosadienoic acid	0,015447233	0,030469424	0,06947844	0,137663449	0,001279795	0,00	0,099330614	0,092380699	0,116355962	0,126820872
cis-Palmitoleic acid	0,031783824	0,062693091	0,403398784	0,799287775	0,011656475	0,02	1,226980961	1,14113217	0,410960247	0,447921498
cis-oleic acid	7,226422393	14,25400399	17,62800927	34,92785023	1,848879293	2,63	12,7950645	11,89982582	67,15885176	73,19903493
Linoleic acid (LA)	14,03892002	27,69154793	14,80583297	29,33603611	3,13447141	4,47	6,460044449	6,008051289	159,3233499	173,6526928
cis-Erucic acid	0,001871718	0,003691933	0,011776123	0,023333018	0,000233226	0,00	0,136080699	0,126559473	0,040647421	0,044303199
Gamma-linolenic acid (GLA)	0,051346422	0,101280007	0,031811427	0,063030643	0,018037061	0,03	0,040315958	0,037495151	0,548474348	0,597803445
Heptadecenoic acid	0,139409397	0,274982833	0,111750301	0,221420225	0,081946739	0,12	0,15040524	0,139881762	0,240956239	0,262627542
Eicosatrienoic acid (ETE)	0,026686663	0,05263902	0,021963771	0,043518657	0,025432191	0,04	0,108544013	0,10094946	0,023424263	0,025531012
Tridecanoic acid	0,004178492	0,00824201	0,0091853	1,82E-02	0,005206636	0,01	0,018660503	0,017354875	0	0
Pentadecenoic acid	0	0	0,000629857	0,001247988	0	0,00	0,008498543	7,90E-03	0	0
alpha-linolenic acid (ALA)	2,167565551	4,275488802	1,980410861	3,923953799	0,53804264	0,77	0,849106601	0,789696735	22,20031889	24,19698782
Eicosatrienoic acid (ETE)	0	0	0,002140713	0,004241574	0	0,00	0,0284165	0,026428268	0,00748379	0,008156873
Dihomo-gamma-linolenic acid (DGL	0	0	0,004940107	0,009788248	0,000515285	0,00	0,058937926	0,054814186	0	0
Arachidonic acid (AA)	0	0	0	0	0	0,00	0	0	0	0
linolenic acid undifferentiated	0	0	0	0	0	0,00	0	0	0	0
Heneicosapentaenoic acid	0	0	0	0	0	0,00	0	0	0	0
Docosatetraenoic acid	0	0	0,003870289	0,007668527	0	0,00	0,027161449	0,025261031	0,003448893	0,003759082
Vaccenic acid	0	0	0,172599402	0,341985641	0	0,00	0	0	0	0
Vitamin E (alpha-tocopherol)	0,029968355	0,059112106	1,756018296	3,479346029	0,000539408	0,00	1,172984515	1,090913721	1,499181457	1,634015963
Folate, total	0,006882771	0,013576157	0,002285715	0,004528879	0,005253938	0,01	0,002607156	0,00242474	0,000750197	0,000817669

rxn ID	minFlux	max Flux
rxn00001	0	1000
rxn00003	-510,2602619	0
rxn00004	0	0
rxn00006	0	0
rxn00007	0	1000
rxn00011	-510,2602619	0
rxn00014	0	0
rxn00015	0,007702147	0,007702147
rxn00016	0	0
rxn00020	0	0
rxn00022	0	1000
rxn00029	0,00101873	0,00101873
rxn00031	0	0
rxn00044	0	0
rxn00047	0	0
rxn00048	0	0,000509365
rxn00060	0,000254683	0,000254683
rxn00062	0	1000
rxn00063	0	1000
rxn00065	0	1000
rxn00067	0	0
rxn00070	0	0
rxn00076	0	1000
rxn00077	0	0,000510507
rxn00085	-1000	0
rxn00086	0	0
rxn00097	-1000	1000
rxn00100	0,000657835	0,000657835
rxn00101	0	1000
rxn00102	-1000	999,9999646
rxn00103	0	1000
rxn00104	-1000	0
rxn00105	-999,9973972	1000
rxn00106	-1000	0
rxn00107	0	0
rxn00109	0	0
rxn00114	-1000	1000
rxn00117	-1000	1000
rxn00119	0,368989262	1000
rxn00121	-0,000254683	0
rxn00122	0	0,000254683
rxn00124	0	0,000254683
rxn00126	0,000764048	1000
rxn00127	0	0
rxn00131	-1000	1000
rxn00132	0	1000
rxn00133	0	0
rxn00134	0	1000
rxn00137	0	1000

rxn00138	0	1000
rxn00139	-999,9971425	0
rxn00140	0	1000
rxn00141	-999,9994906	0
rxn00143	0,000509365	1000
rxn00148	-1000	0
rxn00151	-1000	0
rxn00152	0	499,9997453
rxn00154	0	510,2602619
rxn00157	-510,2602619	0
rxn00159	-1000	1000
rxn00161	-1000	1000
rxn00162	0	1000
rxn00165	0	500,7015214
rxn00170	0	1000
rxn00171	0	510,9893928
rxn00173	-499,9997453	1000
rxn00174	0	999,9999646
rxn00175	0	1000
rxn00176	0	0
rxn00178	-510,2602619	0
rxn00179	-1000	1000
rxn00183	0	1000
rxn00184	-1000	0
rxn00187	0	1000
rxn00189	0	1000
rxn00190	0	1000
rxn00191	-1000	1000
rxn00192	0	1000
rxn00193	0,031494975	0,031494975
rxn00194	0,000436272	510,2606982
rxn00196	0	0
rxn00198	0	1000
rxn00199	0	1000
rxn00200	0	0
rxn00202	0	0
rxn00205	0	0
rxn00206	0,000254683	500
rxn00208	0	0
rxn00209	0	0,000254683
rxn00211	0	0
rxn00212	0	999,6310107
rxn00213	-1000	1000
rxn00214	0	0
rxn00216	0	1000
rxn00221	0	1000
rxn00222	0	0
rxn00223	-1000	1000
rxn00224	0,000254683	1000
rxn00225	-1000	0

rxn00226	0	0
rxn00231	0	0
rxn00237	-1000	1000
rxn00238	-1000	0
rxn00239	0,238807673	1000
rxn00242	0	1000
rxn00245	0	0
rxn00247	0	1000
rxn00248	-1000	1000
rxn00250	-1000	999,9999646
rxn00254	0	0
rxn00255	0	0
rxn00256	-1000	0
rxn00259	0	0
rxn00260	-1000	1000
rxn00262	0	499,9997453
rxn00272	-1000	1000
rxn00273	0	0
rxn00274	0	0
rxn00275	-1000	1000
rxn00278	-1000	0
rxn00279	0	1000
rxn00283	0,027731841	0,027731841
rxn00285	-1000	1000
rxn00290	-1000	1000
rxn00293	-999,9370101	1000
rxn00295	-1000	999,9370101
rxn00297	0	1000
rxn00298	0	0
rxn00299	0	1,092277686
rxn00300	0	0,000509365
rxn00301	0	999,7611923
rxn00302	0	1,092277686
rxn00303	0	1000
rxn00304	-1000	0
rxn00307	0	0
rxn00313	0	1,135828017
rxn00321	0	0
rxn00322	0	0
rxn00324	-499,9997453	0
rxn00328	0	0
rxn00333	0,000254683	500
rxn00337	0,039451804	2,311107838
rxn00338	0	0,002602787
rxn00340	0	1000
rxn00342	0	1000
rxn00346	0	0,000657835
rxn00347	0	1000
rxn00348	0	0
rxn00350	-0,000254683	-0,000254683

rxn00358	0	0
rxn00359	0	1000
rxn00360	0	1000
rxn00361	0	1000
rxn00362	-1000	1000
rxn00363	0	1000
rxn00364	-999,6310107	1000
rxn00365	0	1000
rxn00367	0	1000
rxn00368	0	1000
rxn00369	0	1000
rxn00371	0	1000
rxn00377	0	0
rxn00379	0	1000
rxn00388	0	0
rxn00391	0	999,9997453
rxn00392	0,000254683	1000
rxn00394	0	1000
rxn00395	0	1000
rxn00405	0	0,045698949
rxn00407	0	1000
rxn00409	-1000	1000
rxn00410	-999,8225103	1000
rxn00411	-1000	0
rxn00412	0	1000
rxn00414	0	1000
rxn00416	0	1000
rxn00420	0	0
rxn00422	-1000	1000
rxn00423	0	500,7015214
rxn00424	-1000	1000
rxn00426	0	0
rxn00427	0	0
rxn00433	0	0
rxn00436	0	1000
rxn00437	0	0
rxn00438	-999,9997453	1000
rxn00440	0	1000
rxn00452	0	999,999236
rxn00453	0	0
rxn00459	-335,0923446	509,1222651
rxn00460	-1000	0
rxn00461	0,031494975	0,031494975
rxn00462	0	510,2602619
rxn00463	0	999,6310107
rxn00467	-2,14558705	1000
rxn00469	0	1000
rxn00470	0	0,045698949
rxn00471	0	2,271656034
rxn00473	0	500,7015214

rxn00474	0	500,7015214
rxn00490	0	0
rxn00493	-2,271656034	0
rxn00499	-11,04350924	0
rxn00500	-11,04350924	0
rxn00503	-2,271656034	0
rxn00504	-2,271656034	0
rxn00505	0	1000
rxn00506	0	510,9893928
rxn00510	0	0
rxn00512	-499,9997453	0
rxn00514	0	0
rxn00515	-1000	1000
rxn00517	-1000	0
rxn00527	-2,271656034	0
rxn00533	3,54043E-05	1000
rxn00536	-1000	1000
rxn00537	0	510,2602619
rxn00539	0	0
rxn00540	0	0
rxn00541	-2,271656034	0
rxn00543	-1000	1000
rxn00545	0	1000
rxn00547	0	1000
rxn00549	0	1000
rxn00551	0	1000
rxn00552	-0,06298995	999,9370101
rxn00553	0	1000
rxn00554	0	1000
rxn00555	0	1000
rxn00556	0	1000
rxn00557	0	1000
rxn00558	-1000	1000
rxn00559	0	0
rxn00565	0	0
rxn00566	0	501,0987211
rxn00567	-0,16	0
rxn00575	0	1000
rxn00577	-1000	0,5
rxn00584	0	0
rxn00585	0	0
rxn00591	0	0
rxn00602	0	1000
rxn00604	-1000	1000
rxn00606	0	0
rxn00607	0	0
rxn00608	0	0
rxn00609	0	0
rxn00610	0	1000
rxn00611	-8,283395975	0

rxn00612	-8,283395975	0
rxn00615	0	1000
rxn00616	0	8,283395975
rxn00620	0	1000
rxn00621	0	1000
rxn00622	0	0
rxn00623	0	0
rxn00624	0	0
rxn00629	0	0
rxn00633	0	1000
rxn00634	0	1000
rxn00641	0	0
rxn00642	0	0
rxn00647	0	0
rxn00649	0	500,7015214
rxn00650	-0,000254683	-0,000254683
rxn00653	0	0
rxn00659	0	0
rxn00661	0	0
rxn00670	0	1000
rxn00673	0	0
rxn00674	0	1000
rxn00675	0	0
rxn00677	-1000	1000
rxn00679	0	0
rxn00684	-1,092277686	0
rxn00685	0	1000
rxn00686	-1,092277686	0
rxn00687	0	1000
rxn00689	0	0
rxn00690	0	1000
rxn00691	0	1000
rxn00692	-0,260507082	500,4410144
rxn00693	0,000509365	0,397709
rxn00695	-1000	1000
rxn00702	0	0
rxn00704	-1000	0,5
rxn00707	0	1000
rxn00708	0	1000
rxn00709	0	1000
rxn00710	0	0,365829146
rxn00711	-999,9971425	0
rxn00712	0	1000
rxn00713	0	1000
rxn00714	0	0
rxn00715	0	1000
rxn00717	0	999,6310107
rxn00719	0	0
rxn00726	0	1,135828017
rxn00727	0	1,135828017

rxn00729	0	0
rxn00735	-1000	0
rxn00738	0	0
rxn00741	0	0
rxn00743	0	1000
rxn00745	0	1000
rxn00747	-4,003239303	334,6136622
rxn00748	0	0
rxn00754	0	0
rxn00756	0	0
rxn00758	0	0
rxn00762	-8,283395975	0
rxn00763	0	0
rxn00765	0	0
rxn00768	0	0
rxn00769	0	0
rxn00770	0,002857469	1000
rxn00772	0	1000
rxn00775	0	0
rxn00777	-335,3394393	0,370156845
rxn00778	-1000	1000
rxn00781	-335,0923446	8,966882519
rxn00784	-0,057583371	1,308245774
rxn00785	-333,8513206	1,783673688
rxn00786	-1000	4,003239303
rxn00789	0	0
rxn00790	-0,000254683	-0,000254683
rxn00791	-1,135828017	0
rxn00792	0	0
rxn00796	0	0
rxn00799	-1000	7,853820248
rxn00800	-1,273742578	999,7290863
rxn00801	0	0
rxn00802	0	1000
rxn00806	0	0
rxn00808	0	1000
rxn00809	-1000	0
rxn00810	-1000	1000
rxn00811	-1000	0
rxn00813	-1000	1000
rxn00814	0	0
rxn00815	0	0
rxn00816	0	0
rxn00817	0	0
rxn00818	0	0
rxn00819	0	0
rxn00827	0	0
rxn00829	0,000690955	0,000690955
rxn00830	6,28141E-05	6,28141E-05
rxn00831	0	999,9971425

rxn00832	0	0
rxn00834	-999,7290863	1000
rxn00836	-999,9971425	0
rxn00838	-1,273742578	999,7290863
rxn00851	0	1000
rxn00853	0	0,045698949
rxn00855	0	0
rxn00858	0	0,045698949
rxn00864	0	1000
rxn00865	0	1000
rxn00867	0	0
rxn00869	0	0
rxn00871	0	1000
rxn00872	-8,282631927	0
rxn00874	0	0
rxn00875	0	1000
rxn00879	0	0
rxn00881	0	0
rxn00882	0	0
rxn00883	0	0
rxn00889	0	0
rxn00890	0	0
rxn00897	-1000	0
rxn00898	0	2,271656034
rxn00902	0	0
rxn00903	-1000	1000
rxn00904	-1000	1000
rxn00907	-999,9994906	1,403552247
rxn00908	-1000	1000
rxn00909	-8,282631927	0,397963683
rxn00910	-8,283395975	0
rxn00912	0	0,000657835
rxn00913	0	1000
rxn00915	-999,9971425	0
rxn00916	-999,7319438	1000
rxn00917	0	1000
rxn00918	0	0
rxn00921	0	0
rxn00925	0	0
rxn00926	0	999,9971425
rxn00927	-1000	1000
rxn00929	-1000	1000
rxn00931	-1000	1000
rxn00938	0	999,9971425
rxn00942	0	1000
rxn00946	0	0
rxn00947	0	1000
rxn00950	0	0
rxn00952	0	0,397199635
rxn00955	0,000509365	1000

rxn00966	0	0
rxn00973	-1000	1000
rxn00974	-1000	1000
rxn00977	0	0
rxn00979	0,000254683	1,092532369
rxn00980	0	0
rxn00983	0	0
rxn00985	-1000	0
rxn00986	0	0
rxn00990	-1000	1000
rxn00991	-0,000690955	-0,000690955
rxn00992	0	0
rxn00994	-1000	8,282631927
rxn00999	0	0
rxn01000	0	2,271656034
rxn01007	0	0
rxn01008	0	0
rxn01011	-500,7015214	0
rxn01013	-500,7015214	0
rxn01015	0	0
rxn01016	0	0
rxn01018	0	0,365829146
rxn01019	0	1000
rxn01021	0	0
rxn01022	0	0
rxn01025	0	0
rxn01027	0	0
rxn01029	0	0,045698949
rxn01037	0	0
rxn01042	0	0
rxn01044	0	0
rxn01052	0	0
rxn01056	-1000	1000
rxn01068	0	0
rxn01069	0	2,271656034
rxn01071	0	0
rxn01073	0	0
rxn01078	0	0
rxn01080	0	0
rxn01089	0	0
rxn01094	0	0
rxn01097	0	0
rxn01098	0	0
rxn01100	-1000	0
rxn01101	0	0
rxn01106	-8,966882519	335,0923446
rxn01107	0	1000
rxn01108	-1000	1000
rxn01109	-1000	1000
rxn01114	0	0,5

rxn01115	0	1000
rxn01116	-1,840457982	667,3698173
rxn01117	0	0
rxn01119	0	0
rxn01122	0	0
rxn01123	0	0
rxn01124	0	0
rxn01132	-1000	0,505
rxn01133	0	0
rxn01137	0	999,9971425
rxn01138	-1000	1000
rxn01139	0	0
rxn01146	0	0
rxn01152	0	0,5
rxn01153	0	0
rxn01169	0	1000
rxn01171	-1000	1000
rxn01199	0	0
rxn01200	0	1000
rxn01201	-510,2609529	-0,000690955
rxn01204	0,000690955	510,2609529
rxn01210	0	0
rxn01211	-999,9997453	1,403552247
rxn01213	6,28141E-05	6,28141E-05
rxn01225	0	999,9971425
rxn01226	-999,9707515	1000
rxn01228	0	0
rxn01231	0	0
rxn01236	-1000	0
rxn01237	0	0
rxn01241	-1000	1000
rxn01242	0	510,2602619
rxn01244	0	0
rxn01245	0	0
rxn01249	-499,9997453	0
rxn01251	0	0
rxn01252	0	499,9997453
rxn01255	0,000254683	2,271910716
rxn01256	0	2,271656034
rxn01257	0	1,092277686
rxn01259	0	0
rxn01265	-999,9997453	0
rxn01268	0	2,271656034
rxn01274	0	0
rxn01275	0	1000
rxn01276	0	0
rxn01277	0	0
rxn01278	0	0
rxn01286	0	0
rxn01290	0	0

rxn01291	0	0
rxn01292	0	0,5
rxn01293	0	0
rxn01297	-999,7319438	999,9971425
rxn01299	-1000	1000
rxn01300	0	2,271656034
rxn01301	-2,271910716	0
rxn01302	-2,271910716	0
rxn01303	0,000254683	0,397454318
rxn01304	0	0
rxn01305	0	0
rxn01308	0	0
rxn01310	-1000	0
rxn01313	0	0
rxn01314	0	0
rxn01316	-1000	0
rxn01321	0	0
rxn01322	0	1000
rxn01329	0	0
rxn01332	0,000254683	2,271910716
rxn01333	-1000	334,4866811
rxn01334	0	1000
rxn01336	0	0
rxn01338	0	0
rxn01339	0	0
rxn01343	0	1000
rxn01346	0	1000
rxn01347	0	1000
rxn01348	0	1000
rxn01351	0	1000
rxn01352	-1000	-0,029248515
rxn01353	-1000	1000
rxn01354	-1000	0
rxn01355	0	1000
rxn01358	-1000	1000
rxn01360	0	499,9997453
rxn01361	-499,9997453	0
rxn01362	-0,365829146	0
rxn01365	0	0
rxn01366	-999,968505	1000
rxn01367	0	0
rxn01368	0	999,6310107
rxn01370	0	1000
rxn01374	0	0
rxn01376	0	0
rxn01377	0	0
rxn01379	0	0
rxn01380	0	0
rxn01386	0	0
rxn01387	-1000	0

rxn01388	-1000	1000
rxn01390	0	0
rxn01392	0	0
rxn01396	0	0,000254683
rxn01405	-0,007702147	-0,007702147
rxn01416	0	0
rxn01423	0	0
rxn01426	0	0
rxn01434	0	1000
rxn01437	0	0
rxn01441	0	0
rxn01445	0	999,9707515
rxn01446	-0,029248515	-0,029248515
rxn01453	-999,999309	0
rxn01457	0	0
rxn01459	0	0
rxn01463	0	1000
rxn01464	0	1000
rxn01465	-0,365829146	0
rxn01466	6,28141E-05	6,28141E-05
rxn01476	0	1000
rxn01480	0	0
rxn01484	0	0
rxn01485	-0,06298995	-0,062989949
rxn01486	0	0
rxn01500	-0,000690955	-0,000690955
rxn01506	0	0
rxn01509	-999,9707515	1000
rxn01510	0	1000
rxn01513	0,028334856	0,028334856
rxn01517	0	1000
rxn01518	0,028334856	1000
rxn01519	0	1000
rxn01521	0	999,9716651
rxn01522	0	0
rxn01530	0	0
rxn01537	0	999,9997453
rxn01539	-1000	0
rxn01544	-999,9971425	0
rxn01545	-1000	1000
rxn01548	-999,9707515	1000
rxn01549	0	0
rxn01562	0	0
rxn01575	-2,271656034	0
rxn01583	0	0
rxn01587	0	0
rxn01594	0	0
rxn01597	0	0
rxn01601	0	1,092277686
rxn01602	0	1,092277686

rxn01603	0	1,092277686
rxn01605	0	0
rxn01610	0	0
rxn01615	0	0
rxn01620	0	0
rxn01621	0	0
rxn01626	0	0
rxn01629	-0,00203746	-0,00203746
rxn01637	-1000	0
rxn01639	0	0
rxn01641	0	0
rxn01642	0	0
rxn01643	-2,311107838	-0,039451804
rxn01644	0,031494975	1,167322992
rxn01646	-1000	999,9971425
rxn01647	0	999,9971425
rxn01648	-1000	1000
rxn01649	-1000	1000
rxn01650	0	0
rxn01652	0	1000
rxn01653	0	1000
rxn01654	0	0
rxn01667	-1000	0
rxn01669	0	999,9973972
rxn01670	0	999,9971425
rxn01675	0	0
rxn01678	0	1000
rxn01679	0	1000
rxn01682	-500,7015214	0
rxn01683	-1000	1000
rxn01684	-1000	1000
rxn01685	0	0
rxn01686	-8,282631927	0
rxn01692	0	0
rxn01693	0	0
rxn01704	0	0
rxn01706	0	1000
rxn01709	0	0
rxn01710	0	0
rxn01722	0	0
rxn01723	0	0
rxn01731	-1000	1000
rxn01734	-1000	1000
rxn01735	0	0
rxn01737	0	0
rxn01739	0,000254683	2,271910716
rxn01740	-2,271910716	-0,000254683
rxn01741	0	0
rxn01748	0	0
rxn01750	0	0

rxn01751	0	0
rxn01757	0	0
rxn01761	0	0
rxn01763	0	0,5
rxn01775	0	0
rxn01784	0	0
rxn01790	-0,000657835	0
rxn01791	0	0,000657835
rxn01799	-0,028334856	0,33749429
rxn01800	0	0,365829146
rxn01802	0	0
rxn01804	0	0
rxn01805	0	0
rxn01807	0	0
rxn01810	0	0
rxn01827	0	0
rxn01829	0	0
rxn01831	0	0
rxn01834	0	0
rxn01835	0	0
rxn01842	0	0
rxn01843	0	0
rxn01851	0	0
rxn01855	0	0
rxn01857	0	0
rxn01858	0	999,9971425
rxn01859	-999,9971425	0,5
rxn01860	0	0
rxn01870	0	0
rxn01871	-510,2602619	0
rxn01872	0	0
rxn01873	0	0
rxn01879	0	0
rxn01885	0	0
rxn01892	0	0
rxn01901	0	0
rxn01902	0	0
rxn01906	0	0
rxn01914	0	0
rxn01917	0	1000
rxn01932	0	0
rxn01937	0	0
rxn01946	0	0
rxn01953	-1000	0
rxn01961	0	999,9971425
rxn01962	0	0
rxn01964	0	500,7015214
rxn01966	-1000	0
rxn01967	-1000	0,505
rxn01972	0,031494975	1000

rxn01973	-999,968505	0
rxn01974	0,031494975	1,167322992
rxn01975	-1000	1000
rxn01977	-1000	1000
rxn01982	0	0
rxn01985	0	999,9971425
rxn01986	-0,057583371	0,808245774
rxn01987	-0,5	0
rxn01991	0	0
rxn01996	-1000	0
rxn01997	0	0
rxn01998	0	0
rxn01999	0	0
rxn02000	0	0
rxn02003	0	0
rxn02004	0	0
rxn02007	0	0
rxn02008	0,031494975	0,031494975
rxn02011	0,031494975	0,031494975
rxn02012	0	0
rxn02015	0	0
rxn02020	0	0
rxn02021	0	0
rxn02023	0	0
rxn02033	0	0
rxn02040	0	0
rxn02046	0	0
rxn02056	0	999,9997453
rxn02085	0	0
rxn02089	0	0
rxn02090	0	0
rxn02093	0	0
rxn02100	0	0
rxn02102	-1000	0
rxn02103	0	1000
rxn02106	0	0
rxn02112	-8,282631927	0
rxn02113	0	8,282631927
rxn02118	0	0
rxn02122	0	0
rxn02128	0	0
rxn02138	0	0
rxn02139	0	0
rxn02140	0	0
rxn02144	0	0
rxn02154	0	999,9973972
rxn02155	0,002602787	1000
rxn02160	0	0
rxn02161	0	0
rxn02166	0	0

rxn02167	0	0
rxn02168	0	999,999309
rxn02170	0	0
rxn02171	0,000690955	510,2609529
rxn02173	0	0
rxn02175	0,000657835	1000
rxn02176	0	999,9993422
rxn02177	0	0
rxn02181	0	0
rxn02183	0	0
rxn02185	-8,282631927	510,2602619
rxn02186	0	2,271656034
rxn02187	0	0
rxn02190	0	0
rxn02195	0	0
rxn02196	0	0
rxn02199	0	0
rxn02200	0	1,092277686
rxn02201	0	1,092277686
rxn02209	0	0
rxn02212	0,000254683	2,271910716
rxn02213	0,000254683	2,271910716
rxn02219	0	0
rxn02222	0	0
rxn02228	0	0
rxn02229	0	0
rxn02230	0	0
rxn02235	0	0
rxn02262	0	0
rxn02263	0	0
rxn02264	0,000254683	0,000254683
rxn02272	0	0
rxn02275	0	0
rxn02277	0	0
rxn02283	0	0
rxn02284	-0,031494975	0
rxn02285	-0,031494975	0
rxn02286	0,031494975	0,031494975
rxn02287	-999,9997453	1000
rxn02288	0	0
rxn02297	0	0
rxn02304	0	0
rxn02305	0,000254683	1000
rxn02312	0	0
rxn02313	0	0
rxn02314	0	1000
rxn02315	0	1000
rxn02316	0	1000
rxn02317	-1000	0
rxn02319	0	0

rxn02320	0	0
rxn02321	0	0
rxn02322	0,000690955	0,000690955
rxn02331	0	0
rxn02337	0	0
rxn02339	0	0
rxn02341	0,000657835	0,000657835
rxn02342	0	510,2602619
rxn02350	0	0
rxn02351	0	0
rxn02352	0	0
rxn02356	-1000	1000
rxn02358	-1000	1000
rxn02373	-1000	1000
rxn02377	0	0
rxn02380	-1000	1000
rxn02386	0	0
rxn02400	0	999,9971425
rxn02402	-0,002602787	0
rxn02404	0	0
rxn02405	0	0
rxn02409	0	0
rxn02410	0	0
rxn02415	0	0
rxn02417	0	0
rxn02432	0	0
rxn02433	0	0
rxn02444	0	0
rxn02449	0	1000
rxn02454	0	0
rxn02465	-1000	0
rxn02473	0	0
rxn02474	-0,000509365	0
rxn02475	0	0,000509365
rxn02476	0,000254683	2,271910716
rxn02483	0	0
rxn02484	0,000254683	1000
rxn02495	0	0
rxn02503	0	1,092277686
rxn02504	0	1,092277686
rxn02507	0	1,135828017
rxn02508	0	1,135828017
rxn02517	0	0
rxn02518	0	0
rxn02521	0	0
rxn02522	0	0
rxn02523	0	0
rxn02525	0	0
rxn02527	0	1000
rxn02528	-1000	0

rxn02569	0	0
rxn02570	0	0
rxn02571	0	0
rxn02581	0	0
rxn02596	0	0
rxn02597	0	0
rxn02625	0	0
rxn02632	0	0
rxn02650	0	0
rxn02663	0	0
rxn02679	0	0
rxn02720	0	0
rxn02727	0	0
rxn02729	0	0
rxn02749	0	1000
rxn02751	0	1000
rxn02760	0	1000
rxn02761	0	0
rxn02762	0	0
rxn02774	-999,9997453	0
rxn02775	0	0
rxn02776	0	0
rxn02789	0	0
rxn02791	0	0
rxn02792	0	0
rxn02795	0	0
rxn02796	0	0
rxn02798	0	0
rxn02803	0	0
rxn02811	0	0
rxn02821	0	0
rxn02822	0	0
rxn02831	0	0
rxn02832	0	0
rxn02834	0	0
rxn02835	0	0
rxn02845	0	510,2602619
rxn02849	0	0
rxn02853	0	0
rxn02866	0	0
rxn02869	0	0
rxn02875	0	0
rxn02888	0	0
rxn02889	0	0
rxn02895	0,000254683	0,000254683
rxn02897	0	0
rxn02900	0	0
rxn02906	0	0
rxn02911	0	0
rxn02914	0	0

rxn02922	0	0
rxn02928	-1000	999,968505
rxn02929	-1000	999,968505
rxn02931	0	0
rxn02933	0	0
rxn02934	0	0
rxn02936	0	0
rxn02937	0,000254683	0,000254683
rxn02939	0	0
rxn02943	0	0
rxn02949	0	0
rxn02950	0	0
rxn02974	0	0
rxn02975	0	0
rxn02986	0	0
rxn02988	-0,002602787	0
rxn02990	0	0
rxn02996	0	0
rxn03004	0	0,000254683
rxn03005	-0,000254683	0
rxn03008	0	0
rxn03030	0,031494975	1000
rxn03031	-999,968505	0
rxn03036	0	0
rxn03039	0	0
rxn03042	0	0
rxn03043	0	0
rxn03047	0	0
rxn03060	0	0
rxn03062	0	0
rxn03063	0	0
rxn03064	0	0
rxn03066	0	0
rxn03068	0	0
rxn03071	0	0
rxn03072	0	0
rxn03075	0	999,9997453
rxn03077	0	0
rxn03080	0	0,00101873
rxn03084	0,000254683	0,000254683
rxn03086	-1000	-0,031494975
rxn03087	0	999,968505
rxn03089	0	0
rxn03093	-1000	1000
rxn03094	0	0
rxn03095	0	0
rxn03102	0	0
rxn03106	0	0
rxn03108	0,000254683	1000
rxn03122	0	0

rxn03123	0	0
rxn03130	0	0
rxn03132	0	0
rxn03135	0	0
rxn03136	0	0
rxn03137	0	0
rxn03140	0	0
rxn03141	0	0
rxn03146	0	0
rxn03147	0	0
rxn03150	0	0
rxn03158	0	0
rxn03159	0	0
rxn03164	0,031494975	0,031494975
rxn03167	0	1,092277686
rxn03173	0	0
rxn03174	-1,092277686	0
rxn03175	0	0
rxn03181	0	0
rxn03182	0	0
rxn03194	0	2,271656034
rxn03199	0	0
rxn03200	-1000	0
rxn03201	0	1000
rxn03240	0	0
rxn03241	0	0
rxn03245	0	0
rxn03247	0	0
rxn03250	0	0
rxn03251	0	0
rxn03253	0	0
rxn03264	0	0
rxn03269	0	0
rxn03273	0	0
rxn03275	0	0
rxn03282	0	0
rxn03292	0	0
rxn03293	0	0
rxn03295	0	0
rxn03296	0	0
rxn03301	0	0
rxn03304	0	0
rxn03313	0	0
rxn03316	0	0
rxn03319	0	0
rxn03333	0	0
rxn03354	0	0
rxn03362	0	0
rxn03372	0	0
rxn03373	0	0

rxn03378	0	0
rxn03379	0	0
rxn03384	0	0
rxn03387	0	0
rxn03393	0	0
rxn03395	0	0
rxn03397	0	0
rxn03402	0	0
rxn03405	0	0
rxn03406	0	0
rxn03407	0	0
rxn03408	0,031494975	0,031494975
rxn03409	0	0
rxn03419	0	1,092277686
rxn03421	0	1,092277686
rxn03423	0	0
rxn03424	0	0
rxn03435	-2,271656034	0
rxn03436	0	2,271656034
rxn03437	0	2,271656034
rxn03439	0	0
rxn03445	0	0
rxn03446	0	0
rxn03462	0	0
rxn03465	0	0
rxn03467	0	0
rxn03468	0	0
rxn03481	0	0
rxn03482	0	0
rxn03483	0	0
rxn03491	0	0
rxn03492	0	0
rxn03512	0	0
rxn03513	0	0
rxn03514	0	0
rxn03524	0	0
rxn03535	0	0
rxn03536	0	0
rxn03537	0	0
rxn03538	0	0
rxn03540	0	0
rxn03546	0	0
rxn03548	0	1000
rxn03549	0	0
rxn03552	0	0
rxn03553	0	0
rxn03558	0	0
rxn03587	0	0
rxn03594	0	0
rxn03598	0	0

rxn03599	0	0
rxn03604	0	0
rxn03605	0	0
rxn03620	0	0
rxn03625	0	0
rxn03634	0	0
rxn03638	0,062989949	0,06298995
rxn03641	0,000690955	510,2609529
rxn03642	0,000690955	510,2609529
rxn03643	0	0
rxn03644	0	0
rxn03649	0	0
rxn03650	0	0
rxn03674	0	0
rxn03798	-1000	1000
rxn03807	0	0
rxn03809	0	0
rxn03821	0	0
rxn03838	0	0
rxn03839	0	0
rxn03841	0	1,092277686
rxn03842	0	0
rxn03846	0	0
rxn03852	0	0
rxn03864	0	0
rxn03869	0	0
rxn03870	0	0
rxn03874	0	0
rxn03884	0	0
rxn03885	0	0
rxn03887	0	0
rxn03891	6,28141E-05	6,28141E-05
rxn03893	0	0
rxn03900	0	0
rxn03901	0,031494975	0,031494975
rxn03902	0	0
rxn03903	0	0
rxn03904	0,031494975	0,031494975
rxn03907	0	0
rxn03908	0	0
rxn03909	0	0
rxn03910	0	0
rxn03917	0	0
rxn03919	0	0
rxn03924	0	0
rxn03933	0	0
rxn03951	0	1000
rxn03958	0	0
rxn03962	0	0
rxn03963	-1000	0

rxn03964	0	1000
rxn03974	-0,028334856	0
rxn03975	-0,028334856	0
rxn03976	0	0
rxn03978	0	0,06
rxn04016	0	0
rxn04023	0	0
rxn04028	0	0
rxn04045	0	0
rxn04046	0	0
rxn04047	0	0
rxn04048	0	0
rxn04050	0	0
rxn04051	0	0
rxn04052	0	0
rxn04068	0	0
rxn04070	0	0
rxn04082	0	0,5
rxn04113	0	0
rxn04142	0	0
rxn04234	0	0
rxn04274	0	0
rxn04275	0	0
rxn04285	0	0
rxn04286	0	0
rxn04288	0	0
rxn04290	0	0
rxn04308	0	0
rxn04384	0	0
rxn04385	0	0
rxn04413	0	0
rxn04417	0	0
rxn04418	0	0
rxn04432	0	0
rxn04443	0	0
rxn04447	0	0
rxn04480	0	0
rxn04597	0	0
rxn04598	0	0
rxn04651	0	0
rxn04673	0	0
rxn04674	0	0
rxn04676	-0,268981651	1000
rxn04678	-1000	0,268981651
rxn04703	0	0
rxn04704	0	0
rxn04726	0	0
rxn04736	0	0
rxn04751	0	0
rxn04776	0	0

rxn04786	0,007702147	0,007702147
rxn04792	0	0
rxn04794	0	1000
rxn04809	0	0
rxn04810	0	0
rxn04811	0	0
rxn04822	0	0
rxn04830	0	0
rxn04831	0	0
rxn04832	0	0
rxn04833	0	0
rxn04840	0	0
rxn04841	0	0
rxn04865	0	0
rxn04866	0	0
rxn04872	0	0
rxn04873	0	0
rxn04886	0	0
rxn04887	0	0
rxn04894	0	0
rxn04895	0	0
rxn04896	0	0
rxn04903	0	0
rxn04908	0	0
rxn04916	0	0
rxn04919	0	0
rxn04934	-1000	0
rxn04935	0	1000
rxn04938	0	0
rxn04943	0	0
rxn04947	-1000	1000
rxn04952	-1000	0
rxn04953	-1000	0
rxn04954	-8,283395975	0
rxn04960	0	0
rxn04961	0	0
rxn05004	0	0
rxn05005	-1000	0
rxn05006	-1000	0
rxn05010	0	0
rxn05011	0	0
rxn05023	0	0
rxn05025	0	0
rxn05028	6,28141E-05	6,28141E-05
rxn05029	0	0
rxn05030	6,28141E-05	6,28141E-05
rxn05039	0	0,000509365
rxn05040	0	0,00101873
rxn05050	0	0
rxn05054	0	0

rxn05072	0	0
rxn05087	0	0
rxn05088	0	0
rxn05089	0	0
rxn05109	0	1000
rxn05115	0	0
rxn05116	0	1000
rxn05117	0	0,002602787
rxn05119	0	0,002602787
rxn05122	0	0
rxn05124	0	0
rxn05233	0	0
rxn05234	0	0
rxn05236	0	0
rxn05239	0	0
rxn05247	0	0
rxn05248	0	0
rxn05249	0	0
rxn05250	0	0
rxn05251	0	0
rxn05252	0	0
rxn05256	0	0
rxn05269	0	0
rxn05274	0	0
rxn05275	0	0
rxn05276	0	0
rxn05278	0	0
rxn05279	0	0
rxn05280	0	0
rxn05289	0	0
rxn05291	0	0
rxn05322	0	0
rxn05323	0	0
rxn05324	0	0
rxn05325	0	0
rxn05326	0	0
rxn05327	0	0
rxn05328	0	0
rxn05329	0	0
rxn05330	0	0
rxn05331	0	0
rxn05332	0	0
rxn05333	0	0
rxn05334	0	0
rxn05335	0	0
rxn05336	0	0
rxn05337	0	0
rxn05338	0	0
rxn05339	0	0
rxn05340	0	0

rxn05341	0	0
rxn05342	0	0
rxn05343	0	0
rxn05344	0	0
rxn05345	0	0
rxn05346	0	0
rxn05347	0	0
rxn05348	0	0
rxn05350	0	0
rxn05351	0	0
rxn05352	0	0
rxn05353	0	0
rxn05354	0	0
rxn05355	0	0
rxn05356	0	0
rxn05357	0	0
rxn05457	-1000	1000
rxn05465	0	0
rxn05733	0	0
rxn05736	0	1000
rxn05740	-1000	1000
rxn05744	0	0
rxn05759	-0,5	0
rxn05760	-1000	1000
rxn05763	0	0
rxn05771	0	0
rxn05778	0	0
rxn05779	0	0
rxn05794	-1000	0
rxn05833	0	0
rxn05853	0	0
rxn05856	0	0
rxn05871	0	0
rxn05872	0	0
rxn05873	0	0
rxn05874	0	0
rxn05878	0	0
rxn05889	0	0
rxn05890	0	0
rxn05899	0	0
rxn05901	0	0
rxn05918	0	0
rxn05927	0	0
rxn05934	0	0
rxn05937	-1000	1000
rxn05938	-510,2602619	0
rxn05939	-507,685881	1000
rxn05940	-1000	1000
rxn05952	0	0
rxn05962	0	0

rxn05964	0	0
rxn05970	0	0
rxn05981	0	0
rxn05990	0	0
rxn05994	0	0
rxn05995	0	0
rxn06005	0	0
rxn06023	0	0
rxn06025	0	0
rxn06031	0	0
rxn06038	0	0
rxn06043	0	1000
rxn06044	0	0
rxn06045	0	1000
rxn06071	0,000509365	1000
rxn06075	0	0
rxn06077	0	0
rxn06078	0	0
rxn06079	0	1000
rxn06080	0	1000
rxn06081	0	1000
rxn06087	0	0
rxn06090	0	0
rxn06091	0	0
rxn06094	0	1000
rxn06096	-1000	0
rxn06108	-1000	0
rxn06109	-7,585764059	507,6857691
rxn06139	0	1000
rxn06140	0	0
rxn06181	0	1000
rxn06182	0	1000
rxn06190	0	0
rxn06194	0	0
rxn06195	0	0
rxn06196	0	0
rxn06197	0	0
rxn06200	0	0
rxn06201	0	0
rxn06209	0	0
rxn06212	0	0
rxn06217	0	0
rxn06218	0	0
rxn06219	0	0
rxn06227	0	0
rxn06231	0	0
rxn06233	0	0
rxn06239	0	0
rxn06240	0	0
rxn06244	0	0

rxn06251	0	0
rxn06252	-1000	0
rxn06253	0	1000
rxn06280	0	0
rxn06285	0	0
rxn06293	0	0
rxn06298	0	0
rxn06299	0	0
rxn06300	0	0
rxn06316	0	0
rxn06328	0	0
rxn06347	0	0
rxn06348	0	0
rxn06362	0	0
rxn06365	0	0
rxn06373	0	0
rxn06376	0	0
rxn06377	0	1000
rxn06381	0	0
rxn06394	0	0
rxn06400	0	0
rxn06403	0	0
rxn06418	0	0
rxn06425	0	0
rxn06432	0	0
rxn06434	0	0
rxn06435	0	0
rxn06437	0	0
rxn06438	0	0
rxn06439	0	0
rxn06440	0	0
rxn06441	0	0
rxn06443	0	0
rxn06444	0	0
rxn06445	0	0
rxn06446	0	0
rxn06447	0	0
rxn06448	0	0
rxn06449	0	0
rxn06459	0	0
rxn06485	0	0
rxn06489	0	0
rxn06490	0	0
rxn06493	0	1000
rxn06500	0	0
rxn06522	0	0
rxn06525	0	1000
rxn06526	-1000	0
rxn06528	0	0
rxn06538	0	0

rxn06556	0	1000
rxn06565	0	0
rxn06581	0	0
rxn06584	0	0
rxn06591	0,00203746	0,00203746
rxn06592	0	0
rxn06595	0	0
rxn06600	0	1000
rxn06608	0	0
rxn06614	0	0
rxn06621	0	0
rxn06624	0	0
rxn06641	0	0
rxn06648	0	0
rxn06655	0	0
rxn06663	0	0
rxn06664	0	0
rxn06671	0	0
rxn06672	0	0
rxn06678	0	0
rxn06691	0	0
rxn06694	0	0
rxn06699	0	0
rxn06701	0	0
rxn06723	0	0
rxn06726	0	0
rxn06729	0	0
rxn06733	0	0
rxn06737	0	0
rxn06741	0	0
rxn06751	0	0
rxn06752	0	0
rxn06760	0	0
rxn06768	0	0
rxn06798	0	0
rxn06799	0	0
rxn06817	0	0
rxn06823	0	0
rxn06831	0	0
rxn06836	0	0
rxn06837	-1000	0
rxn06838	0	0
rxn06850	0	0
rxn06860	0	0
rxn06864	0	0
rxn06865	0	0
rxn06882	0	0
rxn06887	0	0
rxn06889	0	1000
rxn06890	0	0

rxn06936	0	0
rxn06937	0,00203746	0,00203746
rxn06947	0	0
rxn06958	-1000	-0,000509365
rxn06979	0	0
rxn07056	0	0
rxn07059	0	0
rxn07099	0	0
rxn07122	0	1000
rxn07172	0	0
rxn07181	0	0
rxn07189	0	0
rxn07193	0	0
rxn07199	0	0
rxn07204	0	0
rxn07241	0	0
rxn07251	0	0
rxn07258	0	0
rxn07267	-1000	0
rxn07271	0	0
rxn07272	0	0
rxn07292	0	0
rxn07312	0	0
rxn07325	0	0
rxn07332	0	0
rxn07335	0	0
rxn07430	0	0
rxn07431	0	0
rxn07432	0	0
rxn07433	0	0
rxn07434	0	0
rxn07435	0	0
rxn07437	0	0
rxn07438	0	0
rxn07439	0	0
rxn07441	0	999,968505
rxn07443	0	0
rxn07450	0	0
rxn07456	0	1000
rxn07465	0,007702147	0,007702147
rxn07466	-1000	1000
rxn07473	0	0
rxn07474	0	0
rxn07475	0	0
rxn07476	0	0
rxn07489	0	0
rxn07490	0	0
rxn07573	0	0
rxn07577	0	0
rxn07578	0	0

rxn07579	0	0
rxn07580	0	0
rxn07581	0	0
rxn07584	0	0
rxn07585	0	0
rxn07586	0	0
rxn07587	0	0
rxn07588	0	0
rxn07603	0	0
rxn07623	0	0
rxn07645	0	0
rxn07679	0	0
rxn07680	0	0
rxn07683	0	0
rxn07684	0	0
rxn07687	0	0
rxn07688	0	0
rxn07804	0	0
rxn07807	0	0
rxn07832	0	0
rxn07846	0	0
rxn07849	0	0
rxn07876	0	0
rxn07879	0	0
rxn07882	0	0
rxn07885	0	0
rxn07899	0	0
rxn07900	0	0
rxn07901	0	0
rxn07946	0	0
rxn07947	0	0
rxn07948	0	0
rxn07949	0	0
rxn07950	0	0
rxn07951	0	0
rxn07952	0	0
rxn07960	0	0
rxn07961	0	0
rxn07962	0	0
rxn07963	0	0
rxn07964	0	0
rxn07965	0	0
rxn07966	0	0
rxn07987	0	0
rxn07989	0	0
rxn07991	0	0
rxn07992	0	0
rxn07993	0	0
rxn07994	0	0
rxn07997	0	0

rxn07998	0	0
rxn08000	0	0
rxn08001	0	0
rxn08002	0	0
rxn08014	-1000	1000
rxn08015	0	1000
rxn08016	0	1000
rxn08017	0	1000
rxn08018	0	1000
rxn08019	0	1000
rxn08020	0	1000
rxn08021	0	1000
rxn08022	0	1000
rxn08025	0	0
rxn08035	0	0
rxn08038	0	1000
rxn08040	0	0
rxn08043	0	2,271656034
rxn08044	0	0
rxn08067	-1000	1000
rxn08083	0	0
rxn08084	0	0
rxn08085	0	0
rxn08086	0	0
rxn08087	0	0
rxn08088	0	0
rxn08089	0	0
rxn08094	0	1000
rxn08126	0	0
rxn08127	0	0
rxn08128	0	0
rxn08129	0	0
rxn08133	0	0
rxn08180	0	0
rxn08194	-1000	1000
rxn08206	0	0
rxn08207	0	0
rxn08208	0	0
rxn08209	0	0
rxn08294	0	0
rxn08295	0	0
rxn08296	0	0
rxn08297	0	0
rxn08298	0	0
rxn08299	0	0
rxn08300	0	0
rxn08306	0	0
rxn08307	0	0
rxn08308	0	0
rxn08309	0	0

rxn08310	0	0
rxn08311	0	0
rxn08312	0	0
rxn08352	0	0
rxn08386	0	0
rxn08387	0	0
rxn08390	0	0
rxn08391	0	0
rxn08392	0	0
rxn08393	0	0
rxn08394	0	0
rxn08395	0	0
rxn08396	0	0
rxn08397	0	0
rxn08398	0	0
rxn08399	0	0
rxn08413	0	0
rxn08433	0	1000
rxn08435	0	1000
rxn08436	0	1000
rxn08437	0	1000
rxn08438	0	1000
rxn08448	0	0
rxn08449	0	0
rxn08451	0	0
rxn08453	0	0
rxn08454	0	1000
rxn08455	0	0
rxn08456	0	1000
rxn08457	0	0
rxn08519	0,057583371	0,057583371
rxn08521	0	0
rxn08546	0	1000
rxn08547	0	1000
rxn08548	0	1000
rxn08549	0	1000
rxn08550	0	1000
rxn08551	0	1000
rxn08552	0	1000
rxn08571	0	1000
rxn08582	0	0
rxn08605	0	0
rxn08607	0	0
rxn08615	-1000	1000
rxn08647	0	500,7015214
rxn08668	0	0
rxn08669	0	0
rxn08700	0	0
rxn08706	0	0
rxn08713	0	0

rxn08733	-1000	0
rxn08764	0	2,271656034
rxn08796	0	1000
rxn08797	0	1000
rxn08798	0	1000
rxn08799	0	1000
rxn08800	0	1000
rxn08801	0	1000
rxn08802	0	1000
rxn08803	0	0
rxn08804	0	0
rxn08805	0	0
rxn08806	0	0
rxn08807	0	0
rxn08808	0	0
rxn08809	0	0
rxn08810	0	0
rxn08811	0	0
rxn08812	0	0
rxn08813	0	0
rxn08814	0	0
rxn08815	0	0
rxn08816	0	0
rxn08817	0	0
rxn08818	0	0
rxn08819	0	0
rxn08820	0	0
rxn08821	0	0
rxn08822	0	0
rxn08823	0	0
rxn08838	0	0
rxn08839	0	0
rxn08840	0	0
rxn08841	0	0
rxn08842	0	0
rxn08843	0	0
rxn08844	0	0
rxn08845	0	0
rxn08846	0	0
rxn08847	0	0
rxn08848	0	0
rxn08849	0	0
rxn08850	0	0
rxn08851	0	0
rxn08857	0	0
rxn08889	0,000768616	0,000768616
rxn08890	0,006222019	0,006222019
rxn08891	0,000768616	0,000768616
rxn08892	-999,9854054	1000
rxn08893	-999,9923184	999,993087

rxn08894	-999,9854054	1000
rxn08897	-0,006912974	-0,006912974
rxn08926	0,000690955	0,000690955
rxn08927	-999,9984639	999,9869415
rxn08928	-999,9854054	1000
rxn08929	0,00153609	0,00153609
rxn08930	0	0
rxn08958	0,000768616	0,000768616
rxn09010	0	0
rxn09016	0	999,7611923
rxn09038	0	0
rxn09069	0	0
rxn09101	0	0
rxn09102	0	0
rxn09103	0	0
rxn09104	0	0
rxn09105	0	0
rxn09106	0	0
rxn09107	0	0
rxn09108	0	0
rxn09109	0	0
rxn09110	0	0
rxn09111	0	0
rxn09112	0	0
rxn09113	0	0
rxn09114	0	0
rxn09176	-1000	1000
rxn09177	0	0,000657835
rxn09179	0	0
rxn09180	0	0
rxn09197	0	0
rxn09198	0	0
rxn09199	0	0
rxn09200	0	0
rxn09201	0	0
rxn09202	0	0
rxn09203	0	0
rxn09205	0	0
rxn09206	0	0
rxn09207	0	0
rxn09208	0	0
rxn09209	0	0
rxn09210	0	0
rxn09211	0	0
rxn09235	0,028334856	0,028334856
rxn09237	0,029248515	0,029248515
rxn09240	0	1000
rxn09244	0	0
rxn09264	0	0
rxn09265	0	0

rxn09288	0	0
rxn09340	0	0
rxn09341	0	999,6310107
rxn09348	0	0
rxn09355	0	0
rxn09395	0	0
rxn09398	-1000	1000
rxn09399	0	0
rxn09412	-1000	1000
rxn09445	0	0
rxn09446	0	0
rxn09447	0	0
rxn09454	0	0
rxn09455	0	1000
rxn09456	0	1000
rxn09461	0	0
rxn09473	0	0
rxn09486	-999,9370101	1000
rxn09498	-1000	1000
rxn09499	-1000	1000
rxn09502	0	1000
rxn09519	0	0
rxn09521	0	0
rxn09523	0	0,15
rxn09531	0	0
rxn09557	0,000254683	1000
rxn09616	0,000690955	0,000690955
rxn09625	0	0
rxn09626	0	0
rxn09631	0,000254683	0,000254683
rxn09632	0	1000
rxn09633	0,000254683	0,000254683
rxn09888	0	0
rxn09889	0	0
rxn09949	0	0
rxn09952	0	0
rxn09978	0	0
rxn09979	0	0
rxn09988	0	0
rxn09991	0	0
rxn09992	0	0
rxn09995	0	0
rxn10003	0	0,000657835
rxn10019	0	0
rxn10020	0	0
rxn10021	0	0
rxn10026	0	0
rxn10029	0	0
rxn10030	0	0
rxn10034	0	0

rxn10036	0	0
rxn10038	0	0
rxn10052	-1000	1000
rxn10054	0	999,6310107
rxn10056	0	0,000510507
rxn10058	0	0,000510507
rxn10060	0	0,000510507
rxn10091	-1000	1000
rxn10107	0	0
rxn10110	0	0
rxn10111	0	0
rxn10191	0	0
rxn10192	0	0
rxn10193	0	0
rxn10194	0	0
rxn10196	0	0
rxn10202	0	1000
rxn10203	0	1000
rxn10204	0	1000
rxn10205	0	0
rxn10206	0	0
rxn10207	0	0
rxn10208	0	0
rxn10209	0	0
rxn10210	0	0
rxn10211	0	0
rxn10212	0	0
rxn10213	0	0
rxn10214	0	0
rxn10215	0	0
rxn10216	0	0
rxn10217	0	0
rxn10218	0	0
rxn10219	0	0
rxn10220	0	0
rxn10221	0	0
rxn10222	0	0
rxn10223	0	0
rxn10224	0	0
rxn10225	0	0
rxn10226	0	0
rxn10227	0	0
rxn10228	0	0
rxn10229	0	0
rxn10230	0	0
rxn10231	0	0
rxn10232	0	0
rxn10233	0	0
rxn10234	0	0
rxn10235	0	0

rxn10236	0	0
rxn10237	0	0
rxn10253	0	0
rxn10254	0	0
rxn10255	0	0
rxn10256	0	0
rxn10257	0	0
rxn10258	0	0
rxn10259	0	0
rxn10260	0	0
rxn10261	0	0
rxn10262	0	0
rxn10263	0	0
rxn10264	0	0
rxn10265	0	0
rxn10266	0	0
rxn10267	0	0
rxn10268	0	0
rxn10269	0	0
rxn10270	0	0
rxn10289	0	0
rxn10290	0	0
rxn10291	0	0
rxn10292	0	0
rxn10293	0	0
rxn10294	0	0
rxn10295	0	0
rxn10296	0	0
rxn10297	0	0
rxn10298	0	0
rxn10299	0	0
rxn10300	0	0
rxn10301	0	0
rxn10302	0	0
rxn10303	0	0
rxn10304	0	0
rxn10305	0	0
rxn10306	0	0
rxn10363	0	0
rxn10404	0	0
rxn10405	0	0
rxn10406	0	0
rxn10407	0	0
rxn10408	0	0
rxn10409	0	0
rxn10410	0	0
rxn10476	0	0
rxn10951	0	0,028334856
rxn11007	0,028334856	0,028334856
rxn11510	0	0

rxn11513	0	0
rxn11547	0	0
rxn11548	0	0
rxn11550	0	0
rxn11551	-1000	1000
rxn11552	-1000	1000
rxn11564	0	0
rxn11567	0	0
rxn11571	0	0
rxn11577	0	0
rxn11587	0	0
rxn11599	0	0
rxn11609	0	0
rxn11612	0	0
rxn11641	0	0
rxn11642	0	0
rxn11663	-1000	0
rxn11702	0	0
rxn11703	0	0
rxn11728	0	0
rxn11731	0	0
rxn11732	0	0
rxn11735	0	1000
rxn11749	0	0
rxn11755	0	0
rxn11757	-999,9971425	0
rxn11759	0	999,9971425
rxn11760	-999,9971425	0
rxn11761	0	0
rxn11765	0	0
rxn11766	0	0
rxn11768	0	0
rxn11772	0	0
rxn11773	0	0
rxn11788	0	0
rxn11808	0	0
rxn11809	0	0
rxn11834	0	0
rxn11838	0	0
rxn11879	0	0
rxn11890	0	0
rxn11891	0	0
rxn11894	0	0
rxn11946	0	0
rxn11951	0	0
rxn11962	0	0
rxn11965	0	0
rxn11970	0	0
rxn11984	0	0
rxn12008	-6,28141E-05	-6,28141E-05

rxn12013	0	0
rxn12033	0	0
rxn12053	0	0
rxn12054	0	0
rxn12146	0	0
rxn12147	0	0
rxn12154	0	0
rxn12218	-1000	-0,000254683
rxn12221	0,000254683	1000
rxn12510	0,000657835	0,000657835
rxn12649	-1000	0
rxn12707	0	0
rxn12767	0	0
rxn12768	0	0
rxn12769	0	0
rxn12770	0	0
rxn12771	0	0
rxn12778	0	0
rxn12822	-1000	0
rxn12844	0	0
rxn12845	0	0
rxn12846	0	0
rxn12847	0	0
rxn13420	0,000690955	1000
rxn13421	0,000690955	1000
rxn13477	6,28141E-05	6,28141E-05
rxn13666	0	0
rxn13667	0	0
rxn13668	0	0
rxn13669	0	0
rxn13671	0	0
rxn13672	0	0
rxn13673	0	0
rxn13687	0	0
rxn13705	0	0
rxn13728	0	0
rxn13734	0	0
rxn13735	0	0
rxn13741	0	0,15
rxn13936	0,015363179	0,01536318
rxn13974	-510,2602619	0
rxn13990	0	1000
rxn13996	0	0
rxn14007	-1000	0
rxn14012	6,28141E-05	6,28141E-05
rxn14029	0	0
rxn14033	0	0
rxn14043	0	0
rxn14048	-1000	0
rxn14050	0	0

rxn14054	-1000	0
rxn14063	0	0
rxn14070	0	0
rxn14089	-1000	0
rxn14093	0	0
rxn14120	-1000	1,091258956
rxn14123	0	0
rxn14132	0	0
rxn14147	0	1000
rxn14160	0	0
rxn14173	-0,1	0
rxn14178	-1000	1000
rxn14180	0	0
rxn14191	0	0
rxn14198	0	1000
rxn14219	0	0
rxn14235	0	0
rxn14238	0	0
rxn14250	0	0
rxn14270	0	0
rxn14275	0	0
rxn14276	0	1000
rxn14279	0	0
rxn14297	0	1000
rxn14322	0	0
rxn14346	0	0
rxn14357	0	0
rxn14372	0	0
rxn14399	0	0
rxn90002	-8,966882519	1000
rxn90003	0	0
rxn90004	0	0
rxn90005	-0,028845363	-0,028334856
rxn08173	0	500,3
rxn13688	0	0
rxn13689	0	0,15
Biomass_Bacteria	1,142074	1,142074006
t_Cl	0,005153038	0,005153038
t_Sulfate	0,004294198	0,004294198
t_Cu2+	0,003435359	0,003435359
t_Mg	0,008587254	0,008587254
t_Ca2+	0,005153038	0,005153038
t_NH3	-2,271656034	0
t_H2O	-26,81579492	10
t_Biomass	-1,142074006	-1,142074
t_Butyrate	-8,282631927	0
t_D-Lactate	-11,04350924	0
t_Ethanol	-16,56526385	0
t_Formate	-23,48970097	0
t_H2	0	0,5

t_L-Lactate	-11,04350924	0
t_Nitrite	0	0
t_Phosphate	1,51196664	2,011966651
t_Propionate	-11,04350924	0
t_O2	0	0
t_D-Glucose	0	0,5
t_CO2	-23,48970097	0
t_Acetate	-16,56526385	0
t_Succinate	-8,282631927	0
t_(S,S)-2,3-Butanediol	-8,282631927	0
t_BDOH	-8,282631927	0
t_H2S	-0,397199635	0
Ex_Cl	-0,005153038	-0,005153038
Ex_Sulfate	-0,004294198	-0,004294198
Ex_Cu2+	-0,003435359	-0,003435359
Ex_Mg	-0,008587254	-0,008587254
Ex_Ca2+	-0,005153038	-0,005153038
Ex_NH3	0	2,271656034
Ex_H2O	-10	26,81579492
Ex_Biomass	1,142074	1,142074006
Ex_Butyrate	0	8,282631927
Ex_D-Lactate	0	11,04350924
Ex_Ethanol	0	16,56526385
Ex_Formate	0	23,48970097
Ex_H2	-0,5	0
Ex_L-Lactate	0	11,04350924
Ex_Nitrite	0	0
Ex_Phosphate	-2,011966651	-1,51196664
Ex_Propionate	0	11,04350924
Ex_O2	0	0
Ex_D-Glucose	-0,5	0
Ex_CO2	0	23,48970097
Ex_Acetate	0	16,56526385
Ex_Succinate	0	8,282631927
Ex_(S,S)-2,3-Butanediol	0	8,282631927
Ex_BDOH	0	8,282631927
Ex_H2S	0	0,397199635
t_Fe2	0,007983097	0,007983097
t_fe3	0,007728415	0,007728415
t_Acetaldehyde	-16,56526385	0
t_Adenosine	0	0,5
t_Allantoin	0	0
t_AMP	0	0,5
t_Amylotriose	0	0
t_BIOT	0	0
t_Choline	0	0
t_Cytidine	0	0
t_Cytosine	0	0
t_DAlanine	0	0
t_Deoxyadenosine	0	0,5

t_Deoxycytidine	0	0,365829146
t_Deoxyguanosine	0	0
t_Deoxyinosine	0	0
t_Deoxyuridine	0	0
t_DRibose	0	0,5
t_DSerine	0	0
t_GLUM	0	0
t_Glycerol	0	0
t_GSH	0	0
t_Guanine	0	0
t_H2S2O3	0	0
t_Heme	0,000254683	0,000254683
t_Homocysteine	0	0
t_HYXN	0	0,5
t_Inosine	0	0,5
t_LACT	0	0
t_LAlanine	-1,771656034	0,5
t_LArabinose	0	0,5
t_LArginine	-0,067914008	0,5
t_LAsparagine	-0,635828017	0,5
t_LAspartate	-1,771656034	0,5
t_LCysteine	0,102800365	0,5
t_LGlutamate	-1,771656034	0,5
t_LGlutamine	-0,635828017	0,5
t_LHistidine	0,105185015	0,105185016
t_LInositol	0	0
t_LIsoleucine	-1,949248544	0,322407492
t_LLeucine	0,499999997	0,5
t_LLysine	-0,755060545	0,380767474
t_LMethionine	-0,226433304	0,170766332
t_LPhenylalanine	-2,066082714	0,205573321
t_LThreonine	-1,990134793	0,281521243
t_LTryptophan	-1,07275127	0,063076747
t_LTyrosine	-2,118618118	0,153037917
t_LValine	-1,801235753	0,471078118
t_Maltose	0	0,5
t_Niacin	0	0,002602787
t_Ornithine	0	0
t_PPi	0	0
t_Pyridoxol	0	0
t_XAN	0	0
t_(R)3Hydroxybutanoate	0	0
t_1,3Propanediol	0	0
t_5Deoxyadenosine	0	0
t_Acetoacetate	-8,282631927	0
t_BET	0	0
t_Calomide	0	0
t_Carnosine	0	0
t_Cbl	0	0
t_Citrate	0	0

t_CysGly	0	0
t_Dulcose	0	0
t_Glycine	-1,771656034	0,5
t_Glycolaldehyde	0	0
t_LProline	-2,026338539	0,245317497
t_Maltohexaose	0	0
t_Methanol	0	0
t_NAcetylDglucosamine	0	0
t_PM	0	0
t_Putrescine	0	0
t_Pyridoxal	0,000254683	0,000254683
t_Riboflavin	0	0,000509365
t_Salicin	0	0
t_Sorbitol	0	0
t_Spermidine	0	0
t_Sucrose	0	0,5
t_Taurine	0	0
t_Thiamin	0	0
t_Thymidine	0	0
t_Thyminose	0	0,5
t_TRHL	0	0
t_Uracil	0	0,365829146
t_Uridine	0	0,365829146
t_Ursin	0	0
t_Mn2+	0,003435359	0,003435359
t_Formaldehyde	0	0
t_Fumarate	-8,282631927	0
t_Oxidized glutathione	0	0
t_Adenine	0	0
t_Nicotinamide	0	0
t_4-Hydroxybenzoate	0	0
t_Co2+	0,003435359	0,003435359
t_D-Arabinose	0	0,5
t_D-Glutamate	0	0
t_Nitrate	0	0,1
t_Chorismate	0	0
t_Folate	-1,091258956	0,00101873
t_N-Acetyl-D-mannosamine	0	0
t_Siroheme	0	0
t_Selenate	0	0
t_Menaquinone 7	0	0
t_2-Demethylmenaquinone 8	0	0
t_Menaquinone 8	0	0
t_Ubiquinone-8	0	0
t_2-Oxobutyrate	0	0
t_3MOP	0	0
t_ABEE	0	0
t_Neu5Ac	0	0
t_Glycerol-3-phosphate	0	0
t_H+	-1000	0,5

t_indol	0	0
t_Nicotinamide ribonucleotide	0	0
t_PAN	0	0,000657835
t_Pyridoxal phosphate	0	0
t_Zn2+	0,003435359	0,003435359
t_1,2-Diacyl-sn-glycerol dioctadecanoyl	0	0
t_meso-2,6-Diaminopimelate	0	0
t_L-Serine	-1,771656034	0,5
t_D-Fructose	0	0,5
t_D-Mannose	0	0
t_Cholesterol	0	0
t_D-Mannitol	0	0
t_L-Rhamnose	0	0
t_beta D-Galactose	0	0
t_L-Fucose	0	0
Ex_Fe2	-0,007983097	-0,007983097
Ex_fe3	-0,007728415	-0,007728415
Ex_Acetaldehyde	0	16,56526385
Ex_Adenosine	-0,5	0
Ex_Allantoin	0	0
Ex_AMP	-0,5	0
Ex_Amylotriose	0	0
Ex_BIOT	0	0
Ex_Choline	0	0
Ex_Cytidine	0	0
Ex_Cytosine	0	0
Ex_DAlanine	0	0
Ex_Deoxyadenosine	-0,5	0
Ex_Deoxycytidine	-0,365829146	0
Ex_Deoxyguanosine	0	0
Ex_Deoxyinosine	0	0
Ex_Deoxyuridine	0	0
Ex_DRibose	-0,5	0
Ex_DSerine	0	0
Ex_GLUM	0	0
Ex_Glycerol	0	0
Ex_GSH	0	0
Ex_Guanine	0	0
Ex_Heme	-0,000254683	-0,000254683
Ex_Homocysteine	0	0
Ex_HYXN	-0,5	0
Ex_Inosine	-0,5	0
Ex_LACT	0	0
Ex_LAlanine	-0,5	1,771656034
Ex_LArabinose	-0,5	0
Ex_LArginine	-0,5	0,067914008
Ex_LAsparagine	-0,5	0,635828017
Ex_LAspartate	-0,5	1,771656034
Ex_LCysteine	-0,5	-0,102800365
Ex_LGlutamate	-0,5	1,771656034

Ex_LGlutamine	-0,5	0,635828017
Ex_LHistidine	-0,105185016	-0,105185015
Ex_LInositol	0	0
Ex_LIsoleucine	-0,322407492	1,949248544
Ex_LLeucine	-0,5	-0,499999997
Ex_LLysine	-0,380767474	0,755060545
Ex_LMethionine	-0,170766332	0,226433304
Ex_LPhenylalanine	-0,205573321	2,066082714
Ex_LThreonine	-0,281521243	1,990134793
Ex_LTryptophan	-0,063076747	1,07275127
Ex_LTyrosine	-0,153037917	2,118618118
Ex_LValine	-0,471078118	1,801235753
Ex_Maltose	-0,5	0
Ex_Niacin	-0,002602787	0
Ex_Ornithine	0	0
Ex_PPi	0	0
Ex_XAN	0	0
Ex_(R)3Hydroxybutanoate	0	0
Ex_5Deoxyadenosine	0	0
Ex_Acetoacetate	0	8,282631927
Ex_BET	0	0
Ex_Calomide	0	0
Ex_Carnosine	0	0
Ex_Cbl	0	0
Ex_Citrate	0	0
Ex_CysGly	0	0
Ex_Dulcose	0	0
Ex_Glycine	-0,5	1,771656034
Ex_Glycolaldehyde	0	0
Ex_LProline	-0,245317497	2,026338539
Ex_Maltohexaose	0	0
Ex_Methanol	0	0
Ex_NAcetylDglucosamine	0	0
Ex_PM	0	0
Ex_Putrescine	0	0
Ex_Pyridoxal	-0,000254683	-0,000254683
Ex_Riboflavin	-0,000509365	0
Ex_Salicin	0	0
Ex_Sorbitol	0	0
Ex_Spermidine	0	0
Ex_Sucrose	-0,5	0
Ex_Taurine	0	0
Ex_Thiamin	0	0
Ex_Thymidine	0	0
Ex_Thyminose	-0,5	0
Ex_TRHL	0	0
Ex_Uracil	-0,365829146	0
Ex_Uridine	-0,365829146	0
Ex_Ursin	0	0
Ex_Mn2+	-0,003435359	-0,003435359

Ex_Formaldehyde	0	0
Ex_Fumarate	0	8,282631927
Ex_Oxidized glutathione	0	0
Ex_Adenine	0	0
Ex_Nicotinamide	0	0
Ex_4-Hydroxybenzoate	0	0
Ex_Co2+	-0,003435359	-0,003435359
Ex_D-Arabinose	-0,5	0
Ex_D-Glutamate	0	0
Ex_Nitrate	-0,1	0
Ex_Folate	-0,00101873	1,091258956
Ex_N-Acetyl-D-mannosamine	0	0
Ex_Siroheme	0	0
Ex_Selenate	0	0
Ex_Menaquinone 7	0	0
Ex_2-Demethylmenaquinone 8	0	0
Ex_Menaquinone 8	0	0
Ex_Ubiquinone-8	0	0
Ex_ABEE	0	0
Ex_Neu5Ac	0	0
Ex_H+	-0,5	1000
Ex_indol	0	0
Ex_Nicotinamide ribonucleotide	0	0
Ex_PAN	-0,000657835	0
Ex_Zn2+	-0,003435359	-0,003435359
Ex_1,2-Diacyl-sn-glycerol dioctadecanoyl	0	0
Ex_L-Serine	-0,5	1,771656034
Ex_D-Fructose	-0,5	0
Ex_D-Mannose	0	0
Ex_Cholesterol	0	0
Ex_D-Mannitol	0	0
Ex_L-Rhamnose	0	0
Ex_beta D-Galactose	0	0
Ex_L-Fucose	0	0
t_Arabinan	0	0
t_Starch	0	0,005
t_octanoate	0	0
t_Melibiose	0	0
t_Amylose	0	0
Ex_Arabinan	0	0
Ex_Starch	-0,005	0
Ex_Melibiose	0	0
Ex_Amylose	0	0
t_Raffinose_Melitose	0	0
t_Isovaleric_acid	0	0
t_H2O2	0	0
t_Nitric_oxide	-0,16	0
Ex_Raffinose_Melitose	0	0
Ex_Isovaleric_acid	0	0
Ex_H2O2	0	0

Ex_Nitric_oxide	0	0,16
rxn01207_1	0	0
rxn08972	0	0,15
rxn08973	0	0
rxn06111	0	1000
rxn13726	0	0,15
rxn13727	0	0,15
rxn13729	0	0
rxn10044	0	0,15
rxn08974	0	0
rxn10122	0	0,15
rxn10123	0	0
rxn10124	0	0
rxn12665	0	0,15
rxn06097	0	0,005
t_Sulfite	0	0
Ex_Sulfite	0	0
rxn09526	0	0,15

rxn ID	minFlux	max Flux
rxn00001	0	1000
rxn00003	-511,6966237	0
rxn00006	0	0,199745317
rxn00011	-511,6966237	0
rxn00014	0	0,199745317
rxn00016	0	0
rxn00020	0	0
rxn00022	0	0,505
rxn00028	0	0
rxn00029	0,00101873	0,00101873
rxn00031	0	0
rxn00048	0	0,000509365
rxn00059	0	0
rxn00060	0,000254683	0,000254683
rxn00062	0	1000
rxn00063	0	1000
rxn00065	0	1000
rxn00067	0	0
rxn00076	0	1000
rxn00077	0	0,000510507
rxn00085	-1000	0
rxn00097	-1000	1000
rxn00100	0,000657835	0,000657835
rxn00104	-1000	0
rxn00105	-999,9973972	1000
rxn00106	-1000	0
rxn00107	0	0
rxn00109	0	0
rxn00117	-1000	1000
rxn00119	0,368989262	1000
rxn00121	-0,000254683	0
rxn00122	0	0,000254683
rxn00124	0	0,000254683
rxn00126	0,000827718	36,84238928
rxn00131	-1000	1000
rxn00132	0	1000
rxn00134	0	1000
rxn00137	0	1000
rxn00138	0	1000
rxn00139	-999,9971425	0
rxn00140	0	1000
rxn00141	-999,999427	0
rxn00142	0	0
rxn00143	0,000573036	1000
rxn00144	0	0
rxn00148	-1000	0
rxn00151	-1000	0
rxn00154	0	511,6966237
rxn00157	-511,6966237	0

rxn00159	-1000	1000
rxn00161	-1000	1000
rxn00162	0	1000
rxn00165	0	500,1556373
rxn00170	0	18,25645047
rxn00173	0	0
rxn00179	0	1000
rxn00183	0	1000
rxn00184	-1000	0
rxn00187	0	1000
rxn00189	0	1000
rxn00190	0	1000
rxn00192	0	18,25645047
rxn00193	0,031494975	0,031494975
rxn00194	0	0
rxn00198	0	1000
rxn00199	0	1000
rxn00206	0	201,5474594
rxn00208	0	0
rxn00209	0	0,000254683
rxn00211	0	0
rxn00212	0	999,6310107
rxn00213	-1000	1000
rxn00214	-1	0
rxn00216	0	1000
rxn00222	0	0
rxn00231	0	0
rxn00237	-1000	1000
rxn00238	-1000	0
rxn00239	0,238807673	1000
rxn00242	0	1000
rxn00247	0	1000
rxn00248	-0,45925651	1000
rxn00250	-1000	999,9999646
rxn00256	-508,1327834	0
rxn00258	-1000	999,9999646
rxn00260	-1000	0,459291914
rxn00262	0	201,8644491
rxn00272	-1000	1000
rxn00273	0	0
rxn00274	0	0
rxn00275	-1000	1000
rxn00278	-1000	0
rxn00283	0,027731841	0,027731841
rxn00285	-512,5578034	1,147048895
rxn00289	0	0
rxn00293	0,062989949	1000
rxn00297	0	999,9370101
rxn00299	0	0
rxn00300	0	0,000509365

rxn00301	0	999,7611923
rxn00302	0	0
rxn00303	0	1000
rxn00304	-1000	0
rxn00305	0	1000
rxn00307	0	0
rxn00309	0	0
rxn00313	0	1,038057342
rxn00322	0	0
rxn00324	-201,8644491	0
rxn00333	0	201,8644491
rxn00337	0,039197122	18,29564759
rxn00338	0,002602787	0,002602787
rxn00340	0	1,038057342
rxn00346	0	0,000657835
rxn00347	0	1000
rxn00348	0	0
rxn00350	-0,000254683	-0,000254683
rxn00358	0	0
rxn00359	0	1000
rxn00360	0	1000
rxn00361	0	1000
rxn00362	0	0
rxn00363	0	1000
rxn00364	-999,6310107	1000
rxn00365	0	1000
rxn00367	0	1000
rxn00371	0	1000
rxn00379	0	1000
rxn00391	0	999,9997453
rxn00392	0,000254683	1000
rxn00394	0	0
rxn00395	0	1000
rxn00405	0,045698949	0,045698949
rxn00409	-1000	1000
rxn00410	-999,8225103	999,8085005
rxn00411	-1000	0
rxn00412	0	1000
rxn00414	0	0,519028671
rxn00422	-1000	1000
rxn00423	0	18,25645047
rxn00424	-1000	1000
rxn00426	0	0
rxn00427	0	0,199745317
rxn00433	0	0
rxn00436	0	999,9997453
rxn00438	0,000254683	1000
rxn00441	0	1000
rxn00453	0	18,25645047
rxn00456	0	18,25645047

rxn00459	-0,911185699	9,808141465
rxn00460	-1000	0
rxn00461	0,031494975	0,031494975
rxn00462	0	0
rxn00463	0	999,6310107
rxn00469	0	18,25645047
rxn00471	0	2,076114684
rxn00473	0	500,5925605
rxn00474	0	500,1556373
rxn00490	0	2,076114684
rxn00493	-2,076114684	0
rxn00498	0	0
rxn00500	-12,17096698	0
rxn00503	-2,076114684	0
rxn00504	-2,076114684	0
rxn00510	0	0
rxn00514	0	0
rxn00515	-1000	1000
rxn00517	-1000	0
rxn00519	0	1000
rxn00525	0	2,076114684
rxn00527	-2,076369366	-0,000254683
rxn00533	-999,9999646	1000
rxn00541	0	0
rxn00545	0	4,81785252
rxn00547	0	1000
rxn00551	0	4,81785252
rxn00552	0	1000
rxn00553	0	0
rxn00554	0	4,81785252
rxn00555	0	1000
rxn00556	0	4,81785252
rxn00557	0	4,81785252
rxn00558	-1000	1000
rxn00559	0	0
rxn00565	0	0
rxn00566	0	18,98288377
rxn00567	-0,299745317	-0,000127341
rxn00575	0	1000
rxn00579	-0,5	1000
rxn00602	0	18,80416289
rxn00605	0	0
rxn00608	0	0
rxn00611	-36,61656356	0
rxn00616	0	36,61656356
rxn00621	0	0
rxn00622	0	0
rxn00623	-0,095705802	0
rxn00634	0	1000
rxn00641	0	0

rxn00642	0	0
rxn00647	0	0
rxn00649	0	18,25645047
rxn00650	-0,000254683	-0,000254683
rxn00654	0	0
rxn00670	0	0
rxn00684	0	0
rxn00685	0	999,9989813
rxn00686	0	0
rxn00687	0	999,9989813
rxn00688	0	1000
rxn00689	0	0
rxn00690	0	1000
rxn00692	-0,260507082	499,8951302
rxn00693	0	36,51290093
rxn00695	-1000	1000
rxn00701	0	1000
rxn00704	-1000	1,5
rxn00708	0	1000
rxn00709	0	1000
rxn00710	0	0
rxn00711	-0,33749429	0
rxn00726	0	2,076114684
rxn00727	0	2,076114684
rxn00729	0	0
rxn00735	0	0
rxn00737	0	0,218478759
rxn00742	-0,492905437	18,58568413
rxn00743	0	1000
rxn00745	0	1000
rxn00747	-4,815249734	0,002602787
rxn00754	0	0
rxn00756	0	0
rxn00758	0	0
rxn00762	0	0
rxn00763	0	0
rxn00765	0	0
rxn00770	0,002857469	1000
rxn00772	0,000573036	1000
rxn00775	0	0
rxn00777	-2,283262163	0,351182047
rxn00778	-999,9707515	1000
rxn00781	-0,911185699	9,808141465
rxn00785	-0,175293894	2,18032513
rxn00786	0	4,81785252
rxn00789	-0,692038228	0
rxn00790	-0,415477619	-0,000254683
rxn00791	-2,076114684	0
rxn00792	0	0
rxn00796	0	0

rxn00799	-1000	6,396621186
rxn00800	-0,268565555	999,7290863
rxn00802	0	1000
rxn00806	0	0
rxn00808	0	1
rxn00809	-503,8686484	0
rxn00811	-503,8686484	0
rxn00816	0	0,5
rxn00817	0	0,5
rxn00818	0	0
rxn00819	0	0
rxn00829	0,000690955	0,000690955
rxn00830	6,28141E-05	6,28141E-05
rxn00831	0	999,9971425
rxn00832	-0,692038228	0
rxn00834	0,268056189	0,268565555
rxn00836	-999,9971425	0
rxn00838	-0,268565555	999,7290863
rxn00851	0	1000
rxn00853	0,045698949	0,045698949
rxn00855	0	0
rxn00856	0,007702147	0,007702147
rxn00859	0	1000
rxn00863	0	1000
rxn00865	0	0
rxn00871	0	0
rxn00879	0	0
rxn00881	0	0
rxn00882	0	0
rxn00883	0	0
rxn00889	0	0
rxn00890	0	0
rxn00897	-999,9370101	0
rxn00898	0	2,076114684
rxn00902	0	0
rxn00903	-1000	1000
rxn00904	-1000	1000
rxn00907	-999,9994906	0,311783926
rxn00908	-1000	1000
rxn00909	-0,103407949	36,51315561
rxn00910	-36,61656356	0
rxn00912	0	0,000657835
rxn00913	0	1000
rxn00915	-999,9707515	0
rxn00916	-999,7319438	0,268565555
rxn00917	0	1000
rxn00925	0	0
rxn00926	0	999,9971425
rxn00929	-1000	1000
rxn00931	-1000	1000

rxn00938	0	0
rxn00941	-999,9971425	0
rxn00945	0	0
rxn00947	0	1000
rxn00950	-18,58568413	0,492905437
rxn00952	0	18,25645047
rxn00955	0,000573036	1000
rxn00963	0,000254683	0,2
rxn00965	0	0
rxn00966	-0,199745317	0
rxn00973	-1000	1000
rxn00974	-1000	1000
rxn00977	0	0
rxn00980	0	0
rxn00987	0	0
rxn01000	0	2,076114684
rxn01007	0	0
rxn01008	0	0
rxn01016	0	0
rxn01018	0	0
rxn01019	0	0,519028671
rxn01029	0,045698949	0,045698949
rxn01068	0	0
rxn01073	0	0
rxn01078	0	0
rxn01080	0	0
rxn01094	0	0
rxn01100	-1000	0
rxn01101	0	0
rxn01106	-9,808141465	0,911185699
rxn01108	-1000	1000
rxn01109	-1000	1000
rxn01115	0	0
rxn01116	-2,284280893	0,35084247
rxn01117	0	0
rxn01119	0	0
rxn01122	0	0
rxn01124	0	0
rxn01133	0	0
rxn01138	-1000	999,9707515
rxn01139	0	0
rxn01153	0	0
rxn01169	0	1000
rxn01171	-1000	1000
rxn01188	0	0
rxn01200	0	1000
rxn01210	0	0
rxn01211	-999,9997453	0,311783926
rxn01213	6,28141E-05	6,28141E-05
rxn01228	0	0

rxn01231	0	0
rxn01237	0	0
rxn01241	-1000	1000
rxn01242	0	511,6966237
rxn01243	0	1000
rxn01244	0	0
rxn01245	0	0
rxn01249	-201,8644491	0
rxn01251	0	0
rxn01252	0	201,8644491
rxn01255	0,000254683	2,076369366
rxn01256	0	2,076114684
rxn01257	0	0
rxn01258	0	0
rxn01259	0	0
rxn01268	0	2,076114684
rxn01269	0	2,076114684
rxn01270	-2,076114684	0
rxn01274	0	0
rxn01276	0	0
rxn01278	0	0
rxn01280	0	0
rxn01281	0	0
rxn01290	0	0
rxn01291	0	0
rxn01301	-18,25645047	0
rxn01302	-18,25645047	0
rxn01303	0	18,25645047
rxn01304	0	0
rxn01305	0	0
rxn01310	-1000	0
rxn01313	0	0
rxn01314	0	0
rxn01321	0	0
rxn01322	0	0
rxn01329	0	0
rxn01332	0,000254683	2,076369366
rxn01333	-4,642303944	0,857667511
rxn01334	0	4,81785252
rxn01343	0	4,81785252
rxn01346	0	4,81785252
rxn01347	0	4,81785252
rxn01348	0	4,81785252
rxn01351	0	1000
rxn01352	-1000	-0,029248515
rxn01353	-1000	1000
rxn01354	-1000	0
rxn01355	0	18,80416289
rxn01358	0	999,9971425
rxn01361	0	0

rxn01362	0	0
rxn01367	0	0
rxn01368	0	999,6310107
rxn01379	0	0
rxn01380	0	0
rxn01387	-1000	0
rxn01388	-1000	1000
rxn01396	0	0,000254683
rxn01416	0	0
rxn01418	0	0
rxn01424	0	0
rxn01434	0	1000
rxn01437	0	0
rxn01441	0	0
rxn01445	0	999,9707515
rxn01446	-0,029248515	-0,029248515
rxn01454	-0,000690955	-0,000690955
rxn01457	0	0
rxn01464	0	0
rxn01465	0	0
rxn01466	6,28141E-05	6,28141E-05
rxn01476	0	0
rxn01480	0	0
rxn01484	0	0
rxn01486	0	0
rxn01489	0	0
rxn01492	0	0
rxn01501	-0,000690955	-0,000690955
rxn01506	0	0
rxn01509	-999,9707515	1000
rxn01513	0,028334856	0,028334856
rxn01517	0	1000
rxn01518	0,028334856	1000
rxn01519	0	1000
rxn01521	0	999,9716651
rxn01539	0	0
rxn01544	-999,9971425	0
rxn01548	0,029248515	1000
rxn01549	0	0
rxn01562	0	0
rxn01575	-2,076114684	0
rxn01593	0	0
rxn01594	0	0
rxn01601	0	0
rxn01602	0	0
rxn01603	0	0
rxn01607	0,000690955	0,000690955
rxn01610	0	0
rxn01615	0	0
rxn01620	0	0

rxn01626	0	0
rxn01629	-0,00203746	-0,00203746
rxn01636	-18,25645047	2,076114684
rxn01637	-2,076114684	0
rxn01641	0	0
rxn01643	-18,29564759	-0,039197122
rxn01644	0,031494975	1,069552317
rxn01646	0	999,9971425
rxn01647	0	0
rxn01649	0	999,9971425
rxn01652	0	1000
rxn01653	0	1000
rxn01664	0	0
rxn01667	-1000	0
rxn01669	0	999,9973972
rxn01670	0	999,9971425
rxn01675	0	0
rxn01678	0	1000
rxn01682	-500,1556373	0
rxn01684	0	0
rxn01686	0	0
rxn01704	0	0
rxn01731	0	0
rxn01735	0	0
rxn01738	0	0
rxn01739	0,000254683	2,076369366
rxn01740	-2,076369366	-0,000254683
rxn01741	0	0
rxn01750	0	0
rxn01757	0	0
rxn01784	0	0
rxn01790	-0,000657835	0
rxn01791	0	0,000657835
rxn01799	-0,028334856	0,029248515
rxn01800	0	0,057583371
rxn01807	0	0
rxn01819	0	0
rxn01832	0	0
rxn01834	0	0
rxn01835	0	0
rxn01842	0	0
rxn01843	0	0
rxn01857	0	0
rxn01859	0	0,057583371
rxn01860	0	0
rxn01870	0	0
rxn01871	-511,6966237	0
rxn01872	-1000	0
rxn01873	0	0
rxn01879	0	0

rxn01885	0	0
rxn01906	0	0
rxn01917	0	2,076114684
rxn01932	0	0
rxn01946	0,000254683	0,2
rxn01953	-999,9370101	0
rxn01961	0	999,9971425
rxn01962	0	0
rxn01964	0	500,1556373
rxn01967	0	0
rxn01972	0,031494975	18,28794544
rxn01973	0	0
rxn01974	0,031494975	1,069552317
rxn01977	-1000	1000
rxn01982	0	0
rxn01985	0	0
rxn01986	-0,057583371	0
rxn01987	-0,057583371	0
rxn01991	0	0
rxn01996	-18,80416289	0
rxn01997	0	0
rxn02000	0	0
rxn02003	0	0
rxn02007	0	0
rxn02008	0,031494975	0,031494975
rxn02011	0,031494975	0,031494975
rxn02023	0	0
rxn02046	0	0
rxn02056	0	999,9997453
rxn02062	0	0
rxn02093	0	0
rxn02112	0	0
rxn02128	0	0
rxn02138	0	0
rxn02139	0	0
rxn02154	0	999,9973972
rxn02155	0,002602787	1000
rxn02159	-1000	0
rxn02160	0	0,692038228
rxn02169	0,000690955	0,000690955
rxn02173	0	0
rxn02175	0,000657835	1000
rxn02176	0	999,9993422
rxn02185	-2,076114684	511,6966237
rxn02186	0	2,076114684
rxn02187	0	0
rxn02190	0	0
rxn02195	0	0
rxn02199	0	0
rxn02200	0	0

rxn02201	0	0
rxn02209	0	0
rxn02212	0,000254683	2,076369366
rxn02213	0,000254683	2,076369366
rxn02222	0	0
rxn02236	0	0
rxn02253	0	0
rxn02263	0	0
rxn02264	0,000254683	0,000254683
rxn02275	0	0
rxn02277	0	0
rxn02283	0	0
rxn02284	-0,031494975	0
rxn02285	-0,031494975	0
rxn02286	0,031494975	0,031494975
rxn02287	-999,9997453	1000
rxn02297	0	0
rxn02305	0,000254683	0,000254683
rxn02312	0	0
rxn02314	0	1000
rxn02315	0	1000
rxn02316	0	1000
rxn02317	-1000	0
rxn02319	0	0
rxn02320	-0,692038228	0
rxn02322	0,000690955	0,000690955
rxn02331	0	0
rxn02339	0	0
rxn02341	0,000657835	0,000657835
rxn02342	0	511,6966237
rxn02351	0	0
rxn02356	-1000	1000
rxn02358	-1000	1000
rxn02373	-1000	1000
rxn02376	0	1000
rxn02380	-1000	1000
rxn02398	0	0
rxn02400	0	0
rxn02402	-0,002602787	-0,002602787
rxn02405	0	0
rxn02409	0	0
rxn02444	0	0
rxn02449	0	1000
rxn02454	0	0
rxn02465	-2,076114684	0
rxn02473	0	0,692038228
rxn02474	-0,000509365	0
rxn02475	0	0,000509365
rxn02476	0,000254683	2,076369366
rxn02483	0	0

rxn02484	0	0
rxn02495	0	0
rxn02503	0	0
rxn02504	0	0
rxn02507	0	2,076114684
rxn02508	0	2,076114684
rxn02517	0	0
rxn02518	0	0
rxn02521	0	0
rxn02522	0	0
rxn02570	0	0
rxn02571	0	0
rxn02581	0	0
rxn02596	0	0
rxn02632	0	0
rxn02650	0	0
rxn02678	0	0
rxn02719	0	0
rxn02727	0	0
rxn02749	0	0
rxn02751	0	0
rxn02762	0	0
rxn02768	-0,000690955	-0,000690955
rxn02774	-999,9997453	0
rxn02776	0	0
rxn02789	0	0
rxn02791	0	0
rxn02792	0	0
rxn02798	0	0
rxn02802	0	0
rxn02810	0,000690955	0,000690955
rxn02811	0	0
rxn02821	0	0
rxn02822	0	0
rxn02831	0	0
rxn02832	0	0
rxn02834	0	0,692038228
rxn02835	0	0,692038228
rxn02849	0	0
rxn02875	0	0
rxn02889	0	0
rxn02895	0,000254683	0,415477619
rxn02897	0	0
rxn02898	0	0
rxn02900	0	0
rxn02914	0	0
rxn02922	0	0
rxn02928	-1000	999,968505
rxn02929	-1000	999,968505
rxn02931	0	0

rxn02936	0	0
rxn02937	0,000254683	0,415477619
rxn02938	-0,415222937	0
rxn02945	0	0
rxn02950	0	0
rxn02986	0	0
rxn02988	-0,002602787	-0,002602787
rxn02990	0	0
rxn03003	0	0
rxn03004	0	0,415477619
rxn03005	-0,415477619	0
rxn03008	0	0
rxn03030	0,031494975	18,28794544
rxn03043	0	0
rxn03047	0	0
rxn03052	0	0
rxn03062	0	0
rxn03066	0	0
rxn03068	0	0
rxn03071	0	0
rxn03072	0	0
rxn03075	0,000254683	0,000254683
rxn03077	0	0
rxn03080	0	0,00101873
rxn03084	0,000254683	0,415477619
rxn03086	-18,28794544	-0,031494975
rxn03087	0	0
rxn03102	0	0
rxn03108	0,000254683	0,000254683
rxn03117	0	0
rxn03132	0	0
rxn03135	-0,692038228	0
rxn03136	0	0,415222937
rxn03137	0	0,692038228
rxn03140	0	0
rxn03141	0	0
rxn03146	0	0
rxn03147	0	0,415222937
rxn03150	0	0
rxn03159	0	0
rxn03164	0,031494975	0,031494975
rxn03167	0	0
rxn03174	0	0
rxn03175	0	0,692038228
rxn03181	0	0
rxn03182	0	0
rxn03188	0	0
rxn03194	0	2,076114684
rxn03252	0	0
rxn03263	0	0

rxn03264	0	0
rxn03269	0	0
rxn03273	0	0
rxn03282	0	0
rxn03292	0	0
rxn03293	0	0
rxn03295	0	0
rxn03296	0	0
rxn03301	0	0
rxn03304	0	0
rxn03313	0	0
rxn03316	0	0
rxn03362	0	0
rxn03371	0	73,68312313
rxn03372	0	0
rxn03373	0	0
rxn03374	0	0
rxn03378	0	0
rxn03379	0	0
rxn03387	0	0
rxn03395	0	0
rxn03397	0	0
rxn03402	0	0
rxn03405	0	0
rxn03406	0	0
rxn03407	0	0
rxn03408	0,031494975	0,031494975
rxn03409	0	0
rxn03419	0	0
rxn03421	0	0
rxn03424	0	0
rxn03435	-2,076114684	0
rxn03436	0	2,076114684
rxn03437	0	2,076114684
rxn03439	0	0
rxn03445	0	0
rxn03462	0	0
rxn03465	0	0
rxn03467	0	0
rxn03481	0	0
rxn03482	0	0
rxn03483	0	0
rxn03491	0	0
rxn03492	0	0
rxn03511	0	0
rxn03535	0	0
rxn03536	0	0
rxn03537	0	0
rxn03538	0	0
rxn03540	0	0

rxn03548	0	0
rxn03549	0	0
rxn03558	0	0
rxn03587	0	0
rxn03638	0,062989949	0,06298995
rxn03838	0	0
rxn03839	0	0
rxn03841	0	0
rxn03852	0	0
rxn03869	0	0
rxn03885	0	0
rxn03891	0	0
rxn03893	0	0
rxn03901	0,031494975	0,031494975
rxn03902	0	0
rxn03903	0	0
rxn03904	0,031494975	0,031494975
rxn03907	0	0
rxn03908	0	0
rxn03909	0	0
rxn03910	0	0
rxn03916	0	0
rxn03917	0	0
rxn03918	0	0
rxn03919	0	0
rxn03933	0	0
rxn03951	0	1000
rxn03958	0	0
rxn03962	0	0
rxn03990	0	0,099872659
rxn03991	0	0,099872659
rxn04016	0	0
rxn04050	0	0
rxn04070	0	0
rxn04113	0	0
rxn04142	0	0
rxn04147	0	0
rxn04286	0	0
rxn04308	0	0
rxn04384	0	0
rxn04385	0	0
rxn04413	0	0
rxn04443	0	0
rxn04597	0	0
rxn04598	0	0
rxn04651	0	0
rxn04673	0	0
rxn04674	0	0
rxn04676	-0,444595834	1000
rxn04678	-1000	0,444595834

rxn04702	-0,2	-0,000254683
rxn04704	0	0
rxn04786	0,007702147	0,007702147
rxn04793	0	0
rxn04794	0	1000
rxn04865	0	0
rxn04866	0	0
rxn04943	0	0
rxn04954	-36,61656356	0
rxn04960	0	0
rxn05005	-1000	0
rxn05006	-1000	0
rxn05010	0	0
rxn05023	0	0
rxn05024	0	0
rxn05029	0	0
rxn05030	6,28141E-05	6,28141E-05
rxn05031	0	0
rxn05039	0	0,000509365
rxn05040	0	0,00101873
rxn05050	0	0
rxn05054	0	0
rxn05070	0	0
rxn05071	0	0
rxn05087	0	0
rxn05088	0	0
rxn05108	0	0
rxn05115	0	0
rxn05116	0	1000
rxn05233	0	0
rxn05234	0	0
rxn05236	0	0
rxn05239	0	0
rxn05247	0	0
rxn05248	0	0
rxn05249	0	0
rxn05250	0	0
rxn05251	0	0
rxn05252	0	0
rxn05256	0	0
rxn05269	0	0
rxn05289	0	0
rxn05322	0	0
rxn05323	0	0
rxn05324	0	0
rxn05325	0	0
rxn05326	0	0
rxn05327	0	0
rxn05328	0	0
rxn05329	0	0

rxn05330	0	0
rxn05331	0	0
rxn05332	0	0
rxn05333	0	0
rxn05334	0	0
rxn05335	0	0
rxn05336	0	0
rxn05337	0	0
rxn05338	0	0
rxn05339	0	0
rxn05340	0	0
rxn05341	0	0
rxn05342	0	0
rxn05343	0	0
rxn05344	0	0
rxn05345	0	0
rxn05346	0	0
rxn05347	0	0
rxn05348	0	0
rxn05350	0	0
rxn05351	0	0
rxn05352	0	0
rxn05353	0	0
rxn05354	0	0
rxn05355	0	0
rxn05356	0	0
rxn05357	0	0
rxn05457	-1000	1000
rxn05465	0	0
rxn05733	0	0
rxn05736	0	1000
rxn05740	-1000	1000
rxn05760	-1000	1000
rxn05763	0	0
rxn05771	0	0
rxn05778	0	0
rxn05779	0	0
rxn05794	-1000	0
rxn05872	0	0
rxn05874	0	0
rxn05887	0	0,5
rxn05889	0	0
rxn05890	0	0
rxn05894	-0,299745317	-0,000127341
rxn05901	0	0
rxn05918	0	0
rxn05927	0	0
rxn05934	-1000	0
rxn05938	-511,6966237	0
rxn05939	-508,0396397	1000

rxn05940	-1000	1000
rxn05952	0	0
rxn05962	0	0
rxn05963	0	0
rxn05970	0	0
rxn05979	0	0
rxn05988	0	0
rxn05990	0	0
rxn05994	0	0
rxn05995	0	0
rxn06005	0	0
rxn06023	0	0
rxn06025	0	0
rxn06031	0	0
rxn06033	0	0
rxn06041	0	1000
rxn06043	0	1000
rxn06044	0	0
rxn06071	0	403,0949187
rxn06075	0	0
rxn06077	0	0
rxn06078	0	0
rxn06080	0	0
rxn06086	0	0
rxn06090	0	0
rxn06091	0	0
rxn06096	0	0
rxn06108	-1000	0
rxn06109	-6,128140526	512,5578034
rxn06139	0	0
rxn06140	0	0
rxn06181	0	1000
rxn06182	0	1000
rxn06190	0	0
rxn06195	6,36706E-05	36,84162523
rxn06196	6,36706E-05	36,84162523
rxn06200	0	0
rxn06201	0	0
rxn06209	0	0
rxn06212	0	0
rxn06217	0	0
rxn06218	0	0
rxn06219	0	0
rxn06251	0	0
rxn06252	-1000	0
rxn06253	0	1000
rxn06280	0	0
rxn06285	0	0
rxn06293	0	0
rxn06298	0	0

rxn06299	0	0
rxn06300	0	0
rxn06328	0	0
rxn06348	0	0
rxn06376	0	0
rxn06377	0	1000
rxn06381	0	0
rxn06394	0	0
rxn06400	0	0
rxn06403	0	0
rxn06418	0	0
rxn06432	0	0
rxn06434	0	0
rxn06435	0	0
rxn06437	0	0
rxn06438	0	0
rxn06439	0	0
rxn06440	0	0
rxn06441	0	0
rxn06443	0	0
rxn06444	0	0
rxn06445	0	0
rxn06446	0	0
rxn06447	0	0
rxn06448	0	0
rxn06449	0	0
rxn06485	0	0
rxn06493	0	1000
rxn06515	0	0
rxn06522	0	0
rxn06525	0	1000
rxn06526	-1000	0
rxn06528	0	0
rxn06537	0	0
rxn06538	0	0
rxn06556	0	1000
rxn06557	0	0
rxn06565	0	0
rxn06584	0	0
rxn06591	0,00203746	0,00203746
rxn06595	0	0
rxn06600	0	1000
rxn06608	0	0
rxn06621	0	0
rxn06624	0	0
rxn06641	0	0
rxn06648	0	0
rxn06664	0	0
rxn06671	0	0
rxn06672	0	0

rxn06692	0	0
rxn06699	0	0
rxn06701	0	0
rxn06719	0	0
rxn06723	0	0
rxn06726	0	0
rxn06729	0	0
rxn06737	0	0
rxn06741	0	0
rxn06751	0	0
rxn06752	0	0
rxn06760	0	0
rxn06768	0	0
rxn06798	0	0
rxn06799	0	0
rxn06804	0	0
rxn06805	0	0
rxn06817	0	0
rxn06823	0	0
rxn06831	0	0
rxn06850	0	0
rxn06864	0	0
rxn06865	0	0
rxn06882	0	0
rxn06883	0	0
rxn06887	0	0
rxn06889	0	0
rxn06936	0	0
rxn06937	0,00203746	0,00203746
rxn06947	0	0
rxn06958	-403,0949187	0
rxn06968	0	0
rxn06979	0	0
rxn06983	0	0
rxn06990	-1000	0
rxn07056	0	0
rxn07059	0	0
rxn07099	0	0
rxn07122	0	1000
rxn07172	0	0
rxn07193	0	0
rxn07200	0	0,5
rxn07221	0	0
rxn07223	0	0
rxn07241	0	0
rxn07246	-1000	1000
rxn07247	-1000	1000
rxn07251	0	0
rxn07262	0	0
rxn07267	0	0

rxn07292	0	0
rxn07332	0	0
rxn07430	0	0
rxn07431	0	0
rxn07432	0	0
rxn07433	0	0
rxn07434	0	0
rxn07435	0	0
rxn07437	0	0
rxn07438	0	0
rxn07439	0	0
rxn07441	0	18,25645047
rxn07456	0	0,519028671
rxn07465	0,007702147	0,007702147
rxn07466	-0,029267931	999,9707321
rxn07474	0	0
rxn07486	0	0
rxn07489	0	0
rxn07573	0	0
rxn07577	0	0
rxn07579	0	0
rxn07580	0	0
rxn07581	0	0
rxn07584	0	0
rxn07585	0	0
rxn07586	0	0
rxn07804	0	0
rxn07807	0	0
rxn07832	0	0
rxn07846	0	0
rxn07849	0	0
rxn07946	0	0
rxn07947	0	0
rxn07948	0	0
rxn07949	0	0
rxn07950	0	0
rxn07951	0	0
rxn07952	0	0
rxn07960	0	0
rxn07961	0	0
rxn07962	0	0
rxn07963	0	0
rxn07964	0	0
rxn07965	0	0
rxn07966	0	0
rxn07987	0	0
rxn07989	0	0
rxn07991	0	0
rxn07992	0	0
rxn07993	0	0

rxn07994	0	0
rxn08014	-1000	1000
rxn08015	0	1000
rxn08016	0	1000
rxn08017	0	1000
rxn08018	0	1000
rxn08019	0	1000
rxn08020	0	1000
rxn08021	0	1000
rxn08022	0	1000
rxn08025	0	0
rxn08040	0	0
rxn08043	0	2,076114684
rxn08067	-1000	1000
rxn08083	0	0
rxn08084	0	0
rxn08085	0	0
rxn08086	0	0
rxn08087	0	0
rxn08088	0	0
rxn08089	0	0
rxn08094	0	1000
rxn08126	0	0
rxn08127	0	0
rxn08128	0	0
rxn08129	0	0
rxn08131	0,000254683	0,000254683
rxn08171	0	0
rxn08180	0	0
rxn08194	-1000	1000
rxn08199	0	1000
rxn08200	0	1000
rxn08201	0	1000
rxn08202	0	1000
rxn08203	0	1000
rxn08204	0	1000
rxn08205	0	1000
rxn08294	0	0
rxn08295	0	0
rxn08296	0	0
rxn08297	0	0
rxn08298	0	0
rxn08299	0	0
rxn08300	0	0
rxn08306	0	1000
rxn08307	0	1000
rxn08308	0	1000
rxn08309	0	1000
rxn08310	0	1000
rxn08311	0	1000

rxn08312	0	1000
rxn08352	0	0
rxn08386	0	0
rxn08387	0	0
rxn08390	0	0
rxn08391	0	0
rxn08392	0	0
rxn08393	0	0
rxn08394	0	0
rxn08395	0	0
rxn08396	0	0
rxn08397	0	0
rxn08398	0	0
rxn08399	0	0
rxn08433	0	1000
rxn08434	0	1000
rxn08435	0	1000
rxn08436	0	1000
rxn08437	0	1000
rxn08438	0	1000
rxn08448	0	0
rxn08449	0	0
rxn08451	0	0
rxn08453	0	0
rxn08454	0	1000
rxn08455	0	0
rxn08456	0	0
rxn08457	0	0
rxn08519	0,057583371	0,057583371
rxn08546	0	1000
rxn08547	0	1000
rxn08548	0	1000
rxn08549	0	1000
rxn08550	0	1000
rxn08551	0	1000
rxn08552	0	1000
rxn08582	0	0
rxn08605	0	0
rxn08607	0	0
rxn08615	-1000	1000
rxn08647	0	0
rxn08668	0	0
rxn08669	0	0
rxn08713	0	0
rxn08764	0	2,076114684
rxn08796	0	1000
rxn08797	0	1000
rxn08798	0	1000
rxn08799	0	1000
rxn08800	0	1000

rxn08801	0	1000
rxn08802	0	1000
rxn08803	0	0
rxn08804	0	0
rxn08805	0	0
rxn08806	0	0
rxn08807	0	0
rxn08808	0	0
rxn08809	0	0
rxn08810	0	0
rxn08811	0	0
rxn08812	0	0
rxn08813	0	0
rxn08814	0	0
rxn08815	0	0
rxn08816	0	0
rxn08817	0	0
rxn08818	0	0
rxn08819	0	0
rxn08820	0	0
rxn08821	0	0
rxn08822	0	0
rxn08823	0	0
rxn08838	0	0
rxn08839	0	0
rxn08840	0	0
rxn08841	0	0
rxn08842	0	0
rxn08843	0	0
rxn08844	0	0
rxn08845	0	0
rxn08846	0	0
rxn08847	0	0
rxn08848	0	0
rxn08849	0	0
rxn08850	0	0
rxn08851	0	0
rxn08857	0	0
rxn08889	0,000768616	0,000768616
rxn08890	0,006222019	0,006222019
rxn08891	0,000768616	0,000768616
rxn08892	0,013058474	0,013058474
rxn08893	0,0061455	0,0061455
rxn08894	0,00153609	0,00153609
rxn08897	-0,006912974	-0,006912974
rxn08926	0,000690955	0,000690955
rxn08928	0,00153609	0,00153609
rxn08929	0,00153609	0,00153609
rxn08958	0,000768616	0,000768616
rxn09010	0	0

rxn09016	0	999,7611923
rxn09069	0	0
rxn09101	0	0
rxn09102	0	0
rxn09103	0	0
rxn09104	0	0
rxn09105	0	0
rxn09106	0	0
rxn09107	0	0
rxn09174	0	7,521665157
rxn09176	-1000	1000
rxn09177	0	0,000657835
rxn09197	0	0
rxn09198	0	0
rxn09199	0	0
rxn09200	0	0
rxn09201	0	0
rxn09202	0	0
rxn09203	0	0
rxn09205	0	0
rxn09206	0	0
rxn09207	0	0
rxn09208	0	0
rxn09209	0	0
rxn09210	0	0
rxn09211	0	0
rxn09235	0,028334856	0,028334856
rxn09237	0,029248515	0,029248515
rxn09240	0	812,7124452
rxn09264	0	0
rxn09265	0	0
rxn09340	0	0
rxn09341	0	999,6310107
rxn09348	0	999,6310107
rxn09355	0	0
rxn09395	0	0
rxn09398	-1000	1000
rxn09399	0	0
rxn09402	0	0
rxn09412	-1000	1000
rxn09445	0	0
rxn09446	0	0
rxn09447	0	0
rxn09454	0	0
rxn09455	0	1000
rxn09456	0	1000
rxn09486	0	0
rxn09498	-1000	1000
rxn09499	-1000	1000
rxn09502	0	1000

rxn09507	0	0
rxn09521	0	0
rxn09531	0	0
rxn09557	0,000254683	1000
rxn09632	0	0
rxn09888	0	0
rxn09889	0	0
rxn09949	0	0
rxn09952	0	0
rxn09978	0	0
rxn09979	0	0
rxn09988	0	0
rxn09992	0	0
rxn09995	0	0
rxn10003	0	0,000657835
rxn10019	0	0
rxn10020	0	0
rxn10021	0	0
rxn10052	-1000	1000
rxn10054	0	999,6310107
rxn10056	0	0,000510507
rxn10058	0	0,000510507
rxn10060	0	0,000510507
rxn10091	-1000	1000
rxn10107	0	0
rxn10110	0	0
rxn10111	0	0
rxn10192	0	0
rxn10193	0	0
rxn10202	0	1000
rxn10203	0	1000
rxn10204	0	1000
rxn10205	0	0
rxn10206	0	0
rxn10207	0	0
rxn10208	0	0
rxn10209	0	0
rxn10210	0	0
rxn10211	0	0
rxn10212	0	0
rxn10213	0	0
rxn10214	0	0
rxn10215	0	0
rxn10216	0	0
rxn10217	0	0
rxn10218	0	0
rxn10219	0	0
rxn10220	0	0
rxn10221	0	0
rxn10222	0	0

rxn10223	0	0
rxn10224	0	0
rxn10225	0	0
rxn10226	0	0
rxn10227	0	0
rxn10228	0	0
rxn10229	0	0
rxn10230	0	0
rxn10231	0	0
rxn10232	0	0
rxn10233	0	0
rxn10234	0	0
rxn10235	0	0
rxn10236	0	0
rxn10237	0	0
rxn10253	0	0
rxn10254	0	0
rxn10255	0	0
rxn10256	0	0
rxn10257	0	0
rxn10258	0	0
rxn10265	0	0
rxn10266	0	0
rxn10267	0	0
rxn10268	0	0
rxn10269	0	0
rxn10270	0	0
rxn10289	0	0
rxn10290	0	0
rxn10291	0	0
rxn10292	0	0
rxn10293	0	0
rxn10294	0	0
rxn10295	0	0
rxn10296	0	0
rxn10297	0	0
rxn10298	0	0
rxn10299	0	0
rxn10300	0	0
rxn10301	0	0
rxn10302	0	0
rxn10303	0	0
rxn10304	0	0
rxn10305	0	0
rxn10306	0	0
rxn10363	0	0
rxn10404	0	0
rxn10405	0	0
rxn10406	0	0
rxn10407	0	0

rxn10408	0	0
rxn10409	0	0
rxn10410	0	0
rxn10563	-0,2	-0,000254683
rxn10785	6,28141E-05	6,28141E-05
rxn10790	0,000254683	1000
rxn10798	-1000	-0,000254683
rxn10816	0,000254683	0,000254683
rxn10951	0,028334856	0,028334856
rxn11007	0,028334856	0,028334856
rxn11513	0	0
rxn11547	0	0
rxn11548	0	0
rxn11550	0	0
rxn11551	-1000	1000
rxn11552	-1000	1000
rxn11564	0	0
rxn11567	0	0
rxn11571	0	0
rxn11575	0	0
rxn11577	0	0
rxn11587	0	0
rxn11599	0	0
rxn11609	0	0
rxn11612	0	0
rxn11641	0	0
rxn11663	-1000	0
rxn11702	0	0
rxn11703	0	0
rxn11732	-0,000690955	-0,000690955
rxn11749	0	0
rxn11755	0	0
rxn11759	0	0
rxn11761	0	0
rxn11765	0	0
rxn11766	0	0
rxn11768	0	0
rxn11772	0	0
rxn11773	0	0
rxn11788	0	0
rxn11794	0	0
rxn11878	0	0
rxn11879	0	0
rxn11890	0	0
rxn11894	0	0
rxn11934	-0,095705802	0
rxn11946	0	0
rxn11951	0	0
rxn11962	0	0
rxn12053	0	0

rxn12054	0	0
rxn12218	-1000	-0,000254683
rxn12221	0,000254683	1000
rxn12239	0,000254683	0,000254683
rxn12510	0,000657835	0,000657835
rxn12512	0	0,000657835
rxn12649	-999,9989813	0
rxn12676	-0,000254683	-0,000254683
rxn12822	-1000	0
rxn13141	-0,06298995	-0,062989949
rxn13147	0,000254683	0,000254683
rxn13207	0,000254683	0,000254683
rxn13208	0,000254683	0,000254683
rxn13420	0,000690955	0,000690955
rxn13421	0,000690955	0,000690955
rxn13687	0	0
rxn13741	0	0
rxn13768	-0,4	-0,000509365
rxn13906	-0,007702147	-0,007702147
rxn13936	0,015363179	0,01536318
rxn13974	-511,6966237	0
rxn13990	0	999,9370101
rxn13994	-73,68312313	0
rxn14000	0	0
rxn14028	0	0
rxn14029	0	0
rxn14033	0	0
rxn14043	0	0
rxn14048	-1000	0
rxn14050	0	0
rxn14058	-0,05	-6,36706E-05
rxn14063	0	0
rxn14070	0	0
rxn14093	0	0
rxn14120	-1000	-0,00101873
rxn14123	0	0
rxn14132	0	0
rxn14178	-1000	1000
rxn14180	0	0
rxn14207	6,36706E-05	36,84162523
rxn14238	0	0
rxn14250	0	0
rxn14279	0	0
rxn14299	0	0
rxn14346	0	0
rxn90002	-9,808141465	1000
rxn90003	0	0
rxn90004	0	0
rxn90005	-0,028845363	-0,028334856
rxn08173	0	500

Biomass_Bacteria	1,142074	1,142074006
t_Cl	0,005153038	0,005153038
t_Sulfate	0,004294198	0,1
t_Cu2+	0,003435359	0,003435359
t_Mg	0,008587254	0,008587254
t_Ca2+	0,005153038	0,005153038
t_NH3	-2,076114684	0
t_H2O	-25,05556792	10
t_Biomass	-1,142074006	-1,142074
t_Butyrate	0	0
t_D-Lactate	-12,17096698	0
t_Formate	-25,68359239	0
t_H2	0	0,5
t_Nitrite	0	0
t_Phosphate	1,517143664	2,011966651
t_Propionate	-7,521665157	0
t_O2	0	0
t_D-Glucose	0	0,5
t_CO2	-25,68359239	0
t_Acetate	-18,28838171	0
t_Succinate	-9,128225233	0
t_(S,S)-2,3-Butanediol	0	0
t_BDOH	0	0
t_H2S	-0,822139106	0
Ex_Cl	-0,005153038	-0,005153038
Ex_Sulfate	-0,1	-0,004294198
Ex_Cu2+	-0,003435359	-0,003435359
Ex_Mg	-0,008587254	-0,008587254
Ex_Ca2+	-0,005153038	-0,005153038
Ex_NH3	0	2,076114684
Ex_H2O	-10	25,05556792
Ex_Biomass	1,142074	1,142074006
Ex_Butyrate	0	0
Ex_D-Lactate	0	12,17096698
Ex_Formate	0	25,68359239
Ex_H2	-0,5	0
Ex_Nitrite	0	0
Ex_Phosphate	-2,011966651	-1,517143664
Ex_Propionate	0	7,521665157
Ex_O2	0	0
Ex_D-Glucose	-0,5	0
Ex_CO2	0	25,68359239
Ex_Acetate	0	18,28838171
Ex_Succinate	0	9,128225233
Ex_(S,S)-2,3-Butanediol	0	0
Ex_BDOH	0	0
Ex_H2S	0	0,822139106
t_Fe2	0,007983097	0,007983097
t_fe3	0,007728415	0,007728415
t_Acetaldehyde	0	0

t_Adenosine	0	0,494822979
t_AMP	0	0,494822979
t_Amylotriose	0	0
t_BIOT	0	0
t_Choline	0	0
t_Cytidine	0	0
t_Cytosine	0	0
t_DAlanine	0	0
t_Deoxyadenosine	0	0,057583371
t_Deoxycytidine	0	0,057583371
t_Deoxyguanosine	0	0
t_Deoxyinosine	0	0
t_Deoxyuridine	0	0
t_DRibose	0	0,5
t_DSerine	0	0
t_GLUM	0	0
t_Glycerol	0	0
t_GSH	0	0
t_Guanine	0	0
t_H2S2O3	0	0
t_Heme	0,000254683	0,000254683
t_Homocysteine	0	0
t_HYXN	0	0,494822979
t_Inosine	0	0,494822979
t_LACT	0	0,5
t_LAlanine	-1,576114684	0,5
t_LArabinose	0	0
t_LArginine	-0,145097654	0,373931019
t_LAsparagine	-0,770583611	0,267473732
t_LAspartate	-1,576114684	0,5
t_LCysteine	-0,322139106	0,5
t_LGlutamate	-1,576114684	0,5
t_LGlutamine	-0,538057342	0,5
t_LHistidine	-0,586853213	0,105185016
t_LInositol	0	0
t_LIsoleucine	-1,753707194	0,322407492
t_LLeucine	0,499999997	0,5
t_LLysine	-0,65728987	0,380767474
t_LMethionine	-0,322139106	0,5
t_LPhenylalanine	-1,870541364	0,205573321
t_LThreonine	0,281521241	0,5
t_LTryptophan	-0,756518968	0,5
t_LTyrosine	-1,923076768	0,153037917
t_LValine	-1,605694403	0,471078118
t_Maltose	0	0,5
t_Niacin	0	0
t_Ornithine	0	0
t_PPi	0	0
t_Pyridoxol	0	0
t_XAN	0	0

t_5Deoxyadenosine	0	0
t_Calomide	0	0
t_Carnosine	0	0
t_Cbl	0	0
t_Citrate	0	0
t_CysGly	0	0
t_Dulcose	0	0
t_Glycine	-1,576114684	0,5
t_Glycolaldehyde	0	0
t_LProline	-1,830797189	0,245317497
t_Maltohexaose	0	0
t_Methanol	0	0
t_NAcetylDglucosamine	0	0
t_PM	0	0
t_Putrescine	0	0
t_Pyridoxal	0,000254683	0,000254683
t_Riboflavin	0	0,000509365
t_Sorbitol	0	0
t_Spermidine	0	0
t_Sucrose	0	0,5
t_Taurine	0	0
t_Thiamin	0	0
t_Thyminose	0	0,057583371
t_Uracil	0	0,365829146
t_Uridine	0	0,33749429
t_Mn2+	0,003435359	0,003435359
t_Formaldehyde	-36,84162523	-6,36706E-05
t_Fumarate	-9,128225233	0
t_Oxidized glutathione	0	0
t_Adenine	0	0
t_Nicotinamide	0	0
t_4-Hydroxybenzoate	0	0
t_Co2+	0,003435359	0,003435359
t_D-Glutamate	0	0
t_Nitrate	0,000127341	0,1
t_Chorismate	0	0
t_Folate	0,00101873	0,00101873
t_N-Acetyl-D-mannosamine	0	0
t_Siroheme	0	0
t_Selenate	0	0
t_Menaquinone 7	0	0
t_2-Demethylmenaquinone 8	0	0
t_Menaquinone 8	0	0
t_Ubiquinone-8	0	0
t_2-Oxobutyrate	0	0
t_3MOP	0	0
t_ABEE	0	0
t_Neu5Ac	0	0
t_Glycerol-3-phosphate	0	0
t_H+	-1000	0,5

t_indol	-2,513037937	0
t_Nicotinamide ribonucleotide	0	0
t_PAN	0	0,000657835
t_Pyridoxal phosphate	0	0
t_Zn2+	0,003435359	0,003435359
t_1,2-Diacyl-sn-glycerol dioctadecanoyl	0	0
t_meso-2,6-Diaminopimelate	0	0
t_L-Serine	-1,576114684	0,5
t_D-Fructose	0	0
t_D-Mannose	0	0
t_Cholesterol	0	0
t_beta D-Galactose	0	0
t_L-Fucose	0	0
Ex_Fe2	-0,007983097	-0,007983097
Ex_fe3	-0,007728415	-0,007728415
Ex_Acetaldehyde	0	0
Ex_Adenosine	-0,494822979	0
Ex_AMP	-0,494822979	0
Ex_Amylotriose	0	0
Ex_BIOT	0	0
Ex_Choline	0	0
Ex_Cytidine	0	0
Ex_Cytosine	0	0
Ex_DAlanine	0	0
Ex_Deoxyadenosine	-0,057583371	0
Ex_Deoxycytidine	-0,057583371	0
Ex_Deoxyguanosine	0	0
Ex_Deoxyinosine	0	0
Ex_Deoxyuridine	0	0
Ex_DRibose	-0,5	0
Ex_DSerine	0	0
Ex_GLUM	0	0
Ex_Glycerol	0	0
Ex_GSH	0	0
Ex_Guanine	0	0
Ex_Heme	-0,000254683	-0,000254683
Ex_Homocysteine	0	0
Ex_HYXN	-0,494822979	0
Ex_Inosine	-0,494822979	0
Ex_LACT	-0,5	0
Ex_LAlanine	-0,5	1,576114684
Ex_LArabinose	0	0
Ex_LArginine	-0,373931019	0,145097654
Ex_LAsparagine	-0,267473732	0,770583611
Ex_LAspartate	-0,5	1,576114684
Ex_LCysteine	-0,5	0,322139106
Ex_LGlutamate	-0,5	1,576114684
Ex_LGlutamine	-0,5	0,538057342
Ex_LHistidine	-0,105185016	0,586853213
Ex_LInositol	0	0

Ex_LIsoleucine	-0,322407492	1,753707194
Ex_LLeucine	-0,5	-0,499999997
Ex_LLysine	-0,380767474	0,65728987
Ex_LMethionine	-0,5	0,322139106
Ex_LPhenylalanine	-0,205573321	1,870541364
Ex_LThreonine	-0,5	-0,281521241
Ex_LTryptophan	-0,5	0,756518968
Ex_LTyrosine	-0,153037917	1,923076768
Ex_LValine	-0,471078118	1,605694403
Ex_Maltose	-0,5	0
Ex_Niacin	0	0
Ex_Ornithine	0	0
Ex_PPi	0	0
Ex_XAN	0	0
Ex_5Deoxyadenosine	0	0
Ex_Calomide	0	0
Ex_Carnosine	0	0
Ex_Cbl	0	0
Ex_Citrate	0	0
Ex_CysGly	0	0
Ex_Dulcose	0	0
Ex_Glycine	-0,5	1,576114684
Ex_Glycolaldehyde	0	0
Ex_LProline	-0,245317497	1,830797189
Ex_Maltohexaose	0	0
Ex_Methanol	0	0
Ex_NAcetylDglucosamine	0	0
Ex_PM	0	0
Ex_Putrescine	0	0
Ex_Pyridoxal	-0,000254683	-0,000254683
Ex_Riboflavin	-0,000509365	0
Ex_Sorbitol	0	0
Ex_Spermidine	0	0
Ex_Sucrose	-0,5	0
Ex_Taurine	0	0
Ex_Thiamin	0	0
Ex_Thyminose	-0,057583371	0
Ex_Uracil	-0,365829146	0
Ex_Uridine	-0,33749429	0
Ex_Mn2+	-0,003435359	-0,003435359
Ex_Formaldehyde	6,36706E-05	36,84162523
Ex_Fumarate	0	9,128225233
Ex_Oxidized glutathione	0	0
Ex_Adenine	0	0
Ex_Nicotinamide	0	0
Ex_4-Hydroxybenzoate	0	0
Ex_Co2+	-0,003435359	-0,003435359
Ex_D-Glutamate	0	0
Ex_Nitrate	-0,1	-0,000127341
Ex_Folate	-0,00101873	-0,00101873

Ex_N-Acetyl-D-mannosamine	0	0
Ex_Siroheme	0	0
Ex_Selenate	0	0
Ex_Menaquinone 7	0	0
Ex_2-Demethylmenaquinone 8	0	0
Ex_Menaquinone 8	0	0
Ex_Ubiquinone-8	0	0
Ex_ABEE	0	0
Ex_Neu5Ac	0	0
Ex_H+	-0,5	1000
Ex_indol	0	2,513037937
Ex_Nicotinamide ribonucleotide	0	0
Ex_PAN	-0,000657835	0
Ex_Zn2+	-0,003435359	-0,003435359
Ex_1,2-Diacyl-sn-glycerol dioctadecanoyl	0	0
Ex_L-Serine	-0,5	1,576114684
Ex_D-Fructose	0	0
Ex_D-Mannose	0	0
Ex_Cholesterol	0	0
Ex_beta D-Galactose	0	0
Ex_L-Fucose	0	0
t_Arabinan	0	0
t_Starch	0	0,005
t_octanoate	0	0
t_Melibiose	0	0,5
t_Amylose	0	0
Ex_Arabinan	0	0
Ex_Starch	-0,005	0
Ex_Melibiose	-0,5	0
Ex_Amylose	0	0
t_Raffinose_Melitose	0	0
t_Isovaleric_acid	0	0
t_H2O2	0	0
t_Nitric_oxide	-0,099872659	0
Ex_Raffinose_Melitose	0	0
Ex_Isovaleric_acid	0	0
Ex_H2O2	0	0
Ex_Nitric_oxide	0	0,099872659
rxn01207_1	0	0
rxn08972	0	0
rxn08973	0	0
rxn06111	0	1000
rxn13726	0	0
rxn13727	0	0
rxn13729	0	0
rxn08974	0	0
rxn10122	0	0
rxn10123	0	0
rxn10124	0	0
rxn12665	0	0

rxn06097	0	0,005
t_Sulfite	0	0
Ex_Sulfite	0	0

rxn ID	minFlux	max Flux
rxn00001	0	1000
rxn00003	-3,719090622	0
rxn00004	0	0
rxn00011	-3,719090622	0
rxn00016	0	0
rxn00020	0	1000
rxn00022	0	0,505
rxn00029	0,000809497	0,000809498
rxn00031	0	0
rxn00047	0	0
rxn00060	0,000202374	0,000202374
rxn00062	0	1000
rxn00063	0	1000
rxn00065	0	1000
rxn00067	0	0
rxn00070	0	0
rxn00076	0	1000
rxn00077	0	0,000405656
rxn00085	-1000	0
rxn00086	0	0
rxn00097	-1000	1000
rxn00100	0,000522725	0,000522725
rxn00101	0	11,74046806
rxn00102	-1000	0,756749741
rxn00104	-1000	0
rxn00105	-999,9979318	1000
rxn00106	-1000	0
rxn00109	0	0
rxn00114	-1000	11,50128586
rxn00119	0,293204037	1000
rxn00121	-0,000202374	0
rxn00122	0	0,000202374
rxn00124	0,000202374	0,000202374
rxn00126	0,000607123	0,000607123
rxn00131	-1000	999,9973247
rxn00132	0	1000
rxn00133	0	0
rxn00137	0	0
rxn00139	-999,9977294	0
rxn00140	0	1000
rxn00142	0	0
rxn00143	0,000404749	0,000404749
rxn00144	0	0
rxn00148	-1000	0
rxn00151	-1000	0
rxn00154	0	21,16653134
rxn00157	-21,16653134	0
rxn00159	-1000	1000
rxn00161	-1000	1000

rxn00162	0	1000
rxn00165	0	7,935355476
rxn00171	0	1,137725312
rxn00173	0	1000
rxn00179	0	0
rxn00184	-1000	0
rxn00187	0	1000
rxn00189	0	1000
rxn00190	0,002068211	1000
rxn00192	0	1000
rxn00193	0,025026348	0,025026363
rxn00196	0	0
rxn00200	0	0
rxn00206	0,000202374	174,4158456
rxn00212	0	999,706796
rxn00214	-1,5	0
rxn00216	0	1000
rxn00221	0	1000
rxn00222	0	1000
rxn00224	0,000202374	1000
rxn00225	-1000	0
rxn00227	0	1000
rxn00231	0	0
rxn00239	0,189759923	1000
rxn00242	0	823,2397789
rxn00247	0	1000
rxn00250	-1000	1000
rxn00254	0	0
rxn00255	0	0
rxn00256	-10,78494586	0
rxn00258	-1000	1000
rxn00260	-1000	1000
rxn00262	0	174,4156432
rxn00275	-0,000202374	-0,000202374
rxn00283	0,022036109	0,022036122
rxn00293	-999,9499473	1000
rxn00295	-1000	999,9499473
rxn00297	0	0
rxn00299	0	0
rxn00300	0	0
rxn00301	0	999,8102401
rxn00302	0	0
rxn00303	0	823,2397789
rxn00304	-1000	0
rxn00307	0	0
rxn00313	0	1,859545311
rxn00322	0	0
rxn00324	-174,4156432	0
rxn00328	0	0
rxn00333	0,000202374	174,4158456

rxn00337	0,031146582	1,890691893
rxn00338	0	0,002068212
rxn00340	0	1000
rxn00342	0	1000
rxn00347	0	1000
rxn00350	-0,000202374	-0,000202374
rxn00358	0	0
rxn00359	0	0
rxn00360	0	0
rxn00362	-1000	1000
rxn00363	0	1000
rxn00364	-999,6817696	999,999451
rxn00365	0	1000
rxn00367	0	1000
rxn00368	0	999,6817696
rxn00369	0	1000
rxn00371	0	1000
rxn00388	0	0
rxn00391	0	999,9997976
rxn00392	0,000202374	1000
rxn00405	0	11,74046806
rxn00410	-999,8589642	999,8228054
rxn00411	-1000	0
rxn00412	0	1000
rxn00414	0	1000
rxn00416	0	1000
rxn00423	0	7,935355476
rxn00426	0	0
rxn00433	0	0
rxn00436	0	999,9997976
rxn00437	0	0
rxn00440	0,000202374	1000
rxn00453	0	999,9997976
rxn00456	0	999,9997976
rxn00459	-1,429995404	12,31934088
rxn00460	-1000	-0,318230385
rxn00461	0,025026348	0,025026363
rxn00463	0	999,6817696
rxn00469	0	1000
rxn00470	0	11,50128586
rxn00479	0	0
rxn00490	0	0
rxn00493	-3,719090622	0
rxn00499	-14,1110209	0
rxn00500	-14,1110209	0
rxn00505	0	10,78494586
rxn00506	0	1,137725312
rxn00512	-174,4156432	0
rxn00514	0	0
rxn00517	-1000	0

rxn00527	-3,719090622	0
rxn00533	-1000	1000
rxn00540	0	0
rxn00541	-1,137725312	0
rxn00543	-1,137725312	0
rxn00545	0	1000
rxn00547	0	1
rxn00549	0	1000
rxn00551	0	1000
rxn00552	-0,050052726	999,9499473
rxn00554	0	1000
rxn00555	0	1000
rxn00556	0	999,6817696
rxn00557	0	1000
rxn00558	-1000	1000
rxn00559	0	0
rxn00562	0	0
rxn00565	0	0
rxn00566	0	1000
rxn00575	0	0,5
rxn00585	0	0
rxn00601	0	0
rxn00602	0	1,41422683
rxn00606	0	0
rxn00607	0	0
rxn00608	0	0
rxn00611	-11,71013097	0
rxn00612	-11,71013097	0
rxn00615	0	11,71013097
rxn00616	0	11,71013097
rxn00622	0	0
rxn00641	0	0
rxn00642	0	0
rxn00647	0	0
rxn00649	0	7,935355476
rxn00650	-0,000202374	-0,000202374
rxn00653	0	0
rxn00654	0	0
rxn00670	0	1,41422683
rxn00684	0	0
rxn00685	0	999,9991905
rxn00686	0	0
rxn00687	0	999,9991905
rxn00689	0	0
rxn00690	0	15,31528884
rxn00692	-0,309695572	7,625659904
rxn00693	0	0,418718138
rxn00695	-1000	1000
rxn00698	-1000	0
rxn00701	0	1000

rxn00702	0	0
rxn00704	-1000	1,5
rxn00707	0	1000
rxn00708	0	1000
rxn00709	0	1000
rxn00710	0	0
rxn00711	-999,9977294	0
rxn00712	0	999,6817696
rxn00713	0	1000
rxn00714	0	0
rxn00715	0	1000
rxn00717	0	823,2323891
rxn00719	0	0
rxn00729	0	0
rxn00735	0	0
rxn00737	0	1,414024456
rxn00741	0	0
rxn00742	-1000	0,000202374
rxn00743	0	11,71013097
rxn00747	-5,842068908	0,674308964
rxn00748	0	0
rxn00751	0	0
rxn00756	0	0
rxn00758	0	0
rxn00762	-11,71013097	0
rxn00763	0	0
rxn00765	0	0
rxn00770	0,002270585	1000
rxn00772	0	1000
rxn00775	0	0
rxn00777	-1,66636055	0,362323996
rxn00778	-1000	1000
rxn00781	-1,429995404	12,31934088
rxn00784	0	1,137725312
rxn00785	-0,180959624	2,69292796
rxn00786	-1000	5,842068908
rxn00789	0	0
rxn00790	-0,000202374	-0,000202374
rxn00792	0	0
rxn00797	-1000	1000
rxn00799	-1000	7,870645622
rxn00800	-0,21300133	506,1530405
rxn00801	0	0
rxn00802	0	11,50128586
rxn00803	0	0
rxn00806	0	0
rxn00808	0	1000
rxn00816	0	0,5
rxn00817	0	0,5
rxn00818	0	0

rxn00819	0	0
rxn00827	0	0
rxn00829	0,000549042	0,000549043
rxn00830	4,99129E-05	4,9913E-05
rxn00831	0	999,9977294
rxn00832	0	0
rxn00834	-999,7847282	1000
rxn00836	-999,9977294	0
rxn00838	-0,21300133	506,1530405
rxn00851	0	1000
rxn00856	0,006466902	11,71027527
rxn00858	0	11,74046806
rxn00869	0	0
rxn00872	-11,70380837	0
rxn00875	0	1000
rxn00879	0	0
rxn00881	0	0
rxn00882	0	0
rxn00883	0	0
rxn00889	0	0
rxn00890	0	0
rxn00897	0	0
rxn00898	0	3,719090622
rxn00902	0	0
rxn00903	-3,719090622	0
rxn00907	-15,31488409	0,000404749
rxn00908	-8,007339469	0,129591081
rxn00909	-11,7099286	0,418920513
rxn00910	-11,71013097	0
rxn00913	0	1000
rxn00915	-999,9977294	0
rxn00916	-999,7869988	1000
rxn00917	0	1000
rxn00918	0	0
rxn00926	0	506,3660417
rxn00927	-1000	1000
rxn00929	-1000	1000
rxn00931	-1000	1000
rxn00938	0	999,9977294
rxn00942	0	1000
rxn00947	0	1000
rxn00950	-1000	0,41831339
rxn00952	0	999,9997976
rxn00955	0,000404749	0,000404749
rxn00973	-1000	1000
rxn00974	-1000	1000
rxn00977	0	0
rxn00979	0,000202374	0,000202374
rxn00980	0	0
rxn00983	0	0

rxn00985	-1,41422683	0
rxn00990	-999,999451	11,70435741
rxn00991	-0,000549043	-0,000549042
rxn00994	-1000	11,70380837
rxn01000	0	3,719090622
rxn01008	0	0
rxn01011	0	0
rxn01013	0	0
rxn01016	0	0
rxn01018	0	0
rxn01019	0	11,50128586
rxn01021	0	0
rxn01025	0	0
rxn01027	0	0
rxn01034	0	0
rxn01037	0	0
rxn01041	-1000	1000
rxn01042	-1000	1000
rxn01052	0	0
rxn01069	0	0
rxn01071	0	0
rxn01073	0	0
rxn01080	0	0
rxn01089	0	0
rxn01100	-1000	0
rxn01101	0	0
rxn01103	0	1000
rxn01106	-12,31934088	1,429995404
rxn01114	0	0
rxn01116	-1,66636055	0,362323996
rxn01119	0	0
rxn01122	0	0
rxn01123	0	0
rxn01124	0	0
rxn01133	0	0
rxn01138	-1000	1000
rxn01139	0	0
rxn01169	0	1000
rxn01171	-1000	1000
rxn01199	0	0
rxn01200	0	1000
rxn01201	-11,70435741	-0,000549042
rxn01204	0,000549042	11,70435741
rxn01210	0	0
rxn01211	-15,31508647	0,000404749
rxn01213	4,99129E-05	4,9913E-05
rxn01225	0	999,9977294
rxn01226	-999,9767587	1000
rxn01228	0	0
rxn01241	0	0

rxn01244	0	0
rxn01245	0	0
rxn01255	0,000202374	3,719292996
rxn01256	0	3,719090622
rxn01259	0	0
rxn01261	0	0
rxn01265	-999,9997976	0
rxn01268	0	3,719090622
rxn01274	0	0
rxn01275	0	0
rxn01276	0	0
rxn01278	0	0
rxn01280	0	0
rxn01281	0	0
rxn01286	0	0
rxn01292	0	0
rxn01297	-999,7869988	999,9977294
rxn01299	-1000	1000
rxn01303	0	0
rxn01304	0	0
rxn01305	0	0
rxn01308	0	0
rxn01321	0	0
rxn01322	0	0
rxn01329	0	0
rxn01332	0,000202374	3,719292996
rxn01333	-1000	1,323961209
rxn01334	0	1000
rxn01343	0	1000
rxn01344	0	0
rxn01346	0	1000
rxn01347	0	999,6817696
rxn01348	0	1000
rxn01351	0	1000
rxn01352	-1000	-0,02324128
rxn01354	-1000	0
rxn01355	0	1,41422683
rxn01358	-1000	1000
rxn01361	0	0
rxn01362	0	0
rxn01366	-1000	1000
rxn01367	0	0
rxn01368	0	823,2323891
rxn01370	0	1000
rxn01374	0	0
rxn01377	0	0
rxn01378	0	0
rxn01379	0	0
rxn01388	-1000	1000
rxn01390	0	0

rxn01396	0	0
rxn01416	0	0
rxn01423	0	0
rxn01426	0	0
rxn01434	0	11,50128586
rxn01437	0	0
rxn01445	0	999,9767587
rxn01446	-0,023241294	-0,02324128
rxn01457	0	0
rxn01459	0	11,70415503
rxn01465	0	0
rxn01466	4,99129E-05	4,9913E-05
rxn01476	0	0
rxn01480	0	0
rxn01484	0	0
rxn01485	-0,050052726	-0,050052696
rxn01486	0	0
rxn01492	0	0
rxn01500	-0,000549043	-0,000549042
rxn01506	0	0
rxn01509	-999,9767587	1000
rxn01510	0	1000
rxn01513	0,022515273	0,022515287
rxn01517	0	0
rxn01518	0,022515273	1000
rxn01519	0	0
rxn01521	0	999,9774847
rxn01522	0	0
rxn01539	-1000	-0,000202374
rxn01541	-1000	1000
rxn01544	-999,9977294	0
rxn01545	-1000	1000
rxn01548	-999,9767587	1000
rxn01549	0	0
rxn01562	0	0
rxn01563	0	0
rxn01575	-1,41422683	0
rxn01601	0	0
rxn01602	0	0
rxn01603	0	0
rxn01615	0	0
rxn01620	0	0
rxn01621	0	0
rxn01626	0	0
rxn01629	-0,001618995	-0,001618994
rxn01636	-1000	11,50128586
rxn01637	-11,50128586	0
rxn01641	0	0
rxn01642	0	0
rxn01643	-1,890691893	-0,031146582

rxn01644	0,025026348	1,884571659
rxn01646	-1000	999,9977294
rxn01647	0	999,9977294
rxn01649	-1000	1000
rxn01653	0	0
rxn01654	0	0
rxn01666	0	1000
rxn01667	-1000	0
rxn01669	0	999,9979318
rxn01670	0	999,9977294
rxn01675	0	0
rxn01679	0	0
rxn01683	-1000	1000
rxn01684	-1000	1000
rxn01686	0	0
rxn01704	0	0
rxn01706	0	0
rxn01710	0	0
rxn01735	0	0
rxn01739	0,000202374	3,719292996
rxn01740	-3,719292996	-0,000202374
rxn01741	0	0
rxn01747	0	0
rxn01750	0	0
rxn01757	0	0
rxn01761	0	0
rxn01763	0	0
rxn01775	0	0
rxn01790	0	0
rxn01799	-0,022515287	0,26817785
rxn01800	0	0,290693137
rxn01807	0	0
rxn01831	0	0
rxn01834	0	0
rxn01842	0	0
rxn01843	0	0
rxn01851	0	11,70415503
rxn01857	0	0
rxn01859	0	0,392788756
rxn01860	0	0
rxn01870	0	0
rxn01871	0	0
rxn01892	0	0
rxn01896	0	0
rxn01906	0	0
rxn01911	0	0
rxn01912	0	0
rxn01917	0	11,50128586
rxn01937	0	0
rxn01946	0	0

rxn01953	0	0
rxn01961	0	999,9977294
rxn01962	0	0
rxn01967	0	0
rxn01972	0,025026348	1000
rxn01973	0	0
rxn01974	0,025026348	1,884571659
rxn01977	-1000	1000
rxn01982	0	0
rxn01985	0	0
rxn01986	-0,045756581	0,637725312
rxn01987	-0,5	0
rxn01991	0	0
rxn01996	-1,41422683	0
rxn01997	0	0
rxn02000	0	0
rxn02003	0	0
rxn02008	0,025026348	0,025026363
rxn02011	0,025026348	0,025026363
rxn02020	0	0
rxn02021	0	0
rxn02023	0	0
rxn02033	0	0
rxn02056	0	999,9997976
rxn02090	0	0
rxn02093	0	0
rxn02106	0	0
rxn02122	0	0
rxn02128	0	0
rxn02138	0	0
rxn02139	0	0
rxn02154	0	999,9979318
rxn02155	0,002068211	1000
rxn02160	0	0
rxn02161	0	0
rxn02171	0,000549042	11,70435741
rxn02172	0	0
rxn02173	0	0
rxn02175	0,000522725	1000
rxn02176	0	999,9994773
rxn02185	-3,719090622	1,41422683
rxn02186	0	3,719090622
rxn02187	0	0
rxn02190	0	0
rxn02195	0	0
rxn02202	0	0
rxn02203	0	0
rxn02209	0	0
rxn02212	0,000202374	3,719292996
rxn02213	0,000202374	3,719292996

rxn02219	0	0
rxn02222	0	0
rxn02228	0	0
rxn02229	0	0
rxn02230	0	0
rxn02235	0	0
rxn02264	0,000202374	0,000202374
rxn02275	0	0
rxn02281	0	0
rxn02283	0	0
rxn02284	-0,025026363	0
rxn02285	-0,025026363	0
rxn02286	0,025026348	0,025026363
rxn02287	-999,9997976	1000
rxn02288	0	0
rxn02302	-1000	-0,000202374
rxn02305	0,000202374	0,000202374
rxn02314	0	1000
rxn02315	0	1000
rxn02316	0	999,6817696
rxn02317	-1000	0
rxn02318	0	0
rxn02320	0	0
rxn02321	0	0
rxn02322	0,000549042	0,000549043
rxn02339	0	0
rxn02341	0,000522725	0,000522725
rxn02350	0	0
rxn02351	0	0
rxn02356	-1000	1000
rxn02358	-1000	1000
rxn02369	0	0
rxn02373	-1000	1000
rxn02380	-1000	1000
rxn02400	0	999,9977294
rxn02402	-0,002068212	0
rxn02404	0	0
rxn02405	0	0
rxn02409	0	0
rxn02432	0	0
rxn02433	0	0
rxn02438	-1000	0
rxn02440	0	1000
rxn02449	0	823,2397789
rxn02454	0	0
rxn02465	-11,50128586	0
rxn02476	0,000202374	3,719292996
rxn02483	0	0
rxn02484	0,000202374	0,000202374
rxn02495	0	0

rxn02518	0	0
rxn02521	0	0
rxn02522	0	0
rxn02525	0	0
rxn02569	0	0
rxn02571	0	0
rxn02581	0	0
rxn02596	0	0
rxn02632	0	0
rxn02729	0	0
rxn02749	0	0
rxn02751	0	0
rxn02762	0	0
rxn02774	-999,9997976	0
rxn02775	0	0
rxn02776	0	0
rxn02789	0	0
rxn02811	0	0
rxn02821	0	0
rxn02822	0	0
rxn02832	0	0
rxn02834	0	0
rxn02835	0	0
rxn02853	0	0
rxn02875	0	0
rxn02889	0	0
rxn02895	0,000202374	0,000202374
rxn02897	0	0
rxn02900	0	0
rxn02914	0	0
rxn02922	0	0
rxn02928	-1000	999,9749737
rxn02929	-1000	999,9749737
rxn02931	0	0
rxn02936	0	0
rxn02937	0,000202374	0,000202374
rxn02943	0	0
rxn02950	0	0
rxn02986	0	0
rxn02988	-0,002068212	0
rxn02990	0	0
rxn03004	0	0,000202374
rxn03005	-0,000202374	0
rxn03023	0	0
rxn03030	0,025026348	1000
rxn03039	0	0
rxn03042	0	0
rxn03043	0	0
rxn03047	0	0
rxn03062	0	0

rxn03068	0	0
rxn03075	0	0
rxn03084	0,000202374	0,000202374
rxn03086	-1000	-0,025026348
rxn03087	0	0
rxn03102	0	0
rxn03106	0	0
rxn03108	0,000202374	0,000202374
rxn03123	0	0
rxn03130	0	0
rxn03136	0	0
rxn03137	0	0
rxn03140	0	0
rxn03141	0	0
rxn03146	0	0
rxn03147	0	0
rxn03150	0	0
rxn03158	0	0
rxn03164	0,025026348	0,025026363
rxn03167	0	0
rxn03174	0	0
rxn03182	0	0
rxn03188	0	0
rxn03194	0	1,41422683
rxn03251	0	0
rxn03263	0	0
rxn03264	0	0
rxn03269	0	0
rxn03273	0	0
rxn03282	0	0
rxn03333	0	0
rxn03354	0	0
rxn03362	0	0
rxn03374	0	0
rxn03379	0	0
rxn03384	0	0
rxn03387	0	0
rxn03397	0	0
rxn03402	0	0
rxn03405	0	0
rxn03406	0	0
rxn03407	0	0
rxn03408	0,025026348	0,025026363
rxn03409	0	0
rxn03419	0	0
rxn03421	0	0
rxn03423	0	0
rxn03435	-1,41422683	0
rxn03436	0	1,41422683
rxn03437	0	1,41422683

rxn03439	0	0
rxn03445	0	0
rxn03446	0	0
rxn03462	0	0
rxn03465	0	0
rxn03467	0	0
rxn03468	0	0
rxn03481	0	0
rxn03482	0	0
rxn03483	0	0
rxn03491	0	0
rxn03492	0	0
rxn03512	0	0
rxn03514	0	0
rxn03524	0	0
rxn03535	0	0
rxn03536	0	0
rxn03537	0	0
rxn03538	0	0
rxn03540	0	0
rxn03548	0	1000
rxn03549	0	0
rxn03552	0	0
rxn03553	0	0
rxn03558	0	0
rxn03596	0	0
rxn03598	0	0
rxn03599	0	0
rxn03608	0	0
rxn03634	0	0
rxn03638	0,050052696	0,050052726
rxn03641	0,000549042	11,70435741
rxn03642	0,000549042	11,70435741
rxn03643	0	0
rxn03644	0	0
rxn03663	0	0
rxn03668	0	0
rxn03669	0	0
rxn03670	0	0
rxn03671	0	0
rxn03838	0	0
rxn03839	0	0
rxn03845	0	0
rxn03852	0	0
rxn03869	0	0
rxn03870	0	0
rxn03884	0	0
rxn03885	0	0
rxn03887	0	0
rxn03891	0	0

rxn03901	0,025026348	0,025026363
rxn03902	0	0
rxn03903	0	0
rxn03904	0,025026348	0,025026363
rxn03907	0	0
rxn03908	0	0
rxn03909	0	0
rxn03910	0	0
rxn03917	0	0
rxn03919	0	0
rxn03932	0	0
rxn03933	0	0
rxn03951	0	1000
rxn03958	0	0
rxn03962	0	0
rxn03975	-0,022515287	0
rxn03978	0	0
rxn04045	0	0
rxn04046	0	0
rxn04048	0	0
rxn04050	0	0
rxn04052	0	0
rxn04096	0	0
rxn04113	0	0
rxn04142	0	0
rxn04234	0	0
rxn04308	0	0
rxn04384	0	0
rxn04385	0	0
rxn04413	0	0
rxn04432	0	0
rxn04443	0	0
rxn04476	0	0
rxn04674	0	0
rxn04676	-0,213331966	1000
rxn04678	-1000	0,213331966
rxn04704	0	0
rxn04726	0	0
rxn04736	0	0
rxn04786	0,006120234	0,006120238
rxn04794	0	1000
rxn04822	0	0
rxn04865	0	0
rxn04866	0	0
rxn04952	-1000	0
rxn04953	-1000	0
rxn04954	-11,71013097	0
rxn04960	0	0
rxn05005	-1000	0
rxn05006	-1000	0

rxn05012	0	0
rxn05029	0	0
rxn05030	4,99129E-05	4,9913E-05
rxn05039	0	0
rxn05040	0	0
rxn05050	0	0
rxn05054	0	0
rxn05115	0	0
rxn05116	0	1000
rxn05117	0	0,002068212
rxn05118	0	0
rxn05119	0	0,002068212
rxn05122	0	0
rxn05124	0	0
rxn05233	0	0
rxn05234	0	0
rxn05236	0	0
rxn05247	0	0
rxn05248	0	0
rxn05249	0	0
rxn05250	0	0
rxn05251	0	0
rxn05252	0	0
rxn05269	0	0
rxn05289	0	0
rxn05322	0	0
rxn05323	0	0
rxn05324	0	0
rxn05325	0	0
rxn05326	0	0
rxn05327	0	0
rxn05328	0	0
rxn05329	0	0
rxn05330	0	0
rxn05331	0	0
rxn05332	0	0
rxn05333	0	0
rxn05334	0	0
rxn05335	0	0
rxn05336	0	0
rxn05337	0	0
rxn05338	0	0
rxn05339	0	0
rxn05340	0	0
rxn05341	0	0
rxn05342	0	0
rxn05343	0	0
rxn05344	0	0
rxn05345	0	0
rxn05346	0	0

rxn05347	0	0
rxn05348	0	0
rxn05350	0	0
rxn05457	-1000	0
rxn05465	0	0
rxn05733	0	0
rxn05736	0	1000
rxn05740	-1000	1000
rxn05759	-0,6	0
rxn05760	-1000	1000
rxn05762	0	0
rxn05763	0	0
rxn05778	0	0
rxn05779	0	0
rxn05833	0	0
rxn05853	0	0
rxn05854	0	0
rxn05856	0	0
rxn05871	0	0
rxn05872	0	0
rxn05874	0	0
rxn05893	0	0
rxn05899	0	0
rxn05901	0	0
rxn05918	0	0
rxn05937	-1000	1000
rxn05938	-21,16653134	0
rxn05939	-8,89358E-05	1000
rxn05940	-1000	1,41422683
rxn05958	0	0
rxn05962	0	0
rxn05990	0	0
rxn05994	0	0
rxn06005	0	0
rxn06023	0	0
rxn06031	0	0
rxn06043	0	0
rxn06044	0	0
rxn06045	0	0
rxn06071	0,000404749	348,8316912
rxn06075	0	0
rxn06078	0	0
rxn06096	0	0
rxn06108	-1000	0
rxn06109	-7,65764442	0
rxn06139	0	0
rxn06140	0	0
rxn06155	0	0
rxn06181	0	1000
rxn06182	0	1000

rxn06190	0	0
rxn06195	0	0
rxn06196	0	0
rxn06200	0	0
rxn06201	0	0
rxn06209	0	0
rxn06217	0	0
rxn06218	0	0
rxn06219	0	0
rxn06231	0	0
rxn06244	0	0
rxn06280	0	0
rxn06285	0	0
rxn06293	0	0
rxn06298	0	0
rxn06299	0	0
rxn06300	0	0
rxn06316	0	0
rxn06348	0	0
rxn06394	0	0
rxn06403	0	0
rxn06432	0	0
rxn06434	0	0
rxn06435	0	0
rxn06437	0	0
rxn06438	0	0
rxn06439	0	0
rxn06440	0	0
rxn06441	0	0
rxn06443	0	0
rxn06444	0	0
rxn06445	0	0
rxn06446	0	0
rxn06447	0	0
rxn06448	0	0
rxn06449	0	0
rxn06485	0	0
rxn06489	0	0
rxn06493	0	0
rxn06500	0	0
rxn06522	0	0
rxn06538	0	0
rxn06556	0	0
rxn06565	0	0
rxn06581	0	0
rxn06584	0	0
rxn06591	0,001618994	0,001618995
rxn06592	0	0
rxn06595	0	0
rxn06621	0	0

rxn06624	0	0
rxn06641	0	0
rxn06648	0	0
rxn06660	0	0
rxn06664	0	0
rxn06671	0	0
rxn06672	0	1000
rxn06673	0	1000
rxn06677	0	0
rxn06678	0	0
rxn06701	0	0
rxn06709	0	0
rxn06726	0	0
rxn06731	0	0
rxn06737	0	0
rxn06751	0	0
rxn06752	0	0
rxn06760	0	0
rxn06768	0	0
rxn06799	0	0
rxn06823	0	0
rxn06831	0	0
rxn06860	0	0
rxn06864	0	0
rxn06874	0	0,1
rxn06882	0	0
rxn06883	0	0
rxn06887	0	0
rxn06889	0	1000
rxn06890	0	0
rxn06926	0	0
rxn06934	0	0
rxn06936	0	0
rxn06937	0,001618994	0,001618995
rxn06947	0	0
rxn06958	-348,8316912	-0,000404749
rxn06979	0	0
rxn07056	0	0
rxn07059	0	0
rxn07099	0	0
rxn07172	0	0
rxn07177	0	0
rxn07199	0	0
rxn07251	0	0
rxn07267	0	0
rxn07292	0	0
rxn07430	0	0
rxn07431	0	0
rxn07432	0	0
rxn07433	0	0

rxn07434	0	0
rxn07435	0	0
rxn07437	0	0
rxn07438	0	0
rxn07441	0	999,9749737
rxn07456	0	1000
rxn07465	0,006120234	0,006120238
rxn07466	-1000	1000
rxn07476	0	0
rxn07484	0	0
rxn07485	0	0
rxn07486	0	0
rxn07489	0	0
rxn07492	0	0
rxn07573	0	0
rxn07577	0	0
rxn07578	0	0
rxn07579	0	0
rxn07580	0	0
rxn07586	0	0
rxn07587	0	0
rxn07623	0	0
rxn07645	0	0
rxn07804	0	0
rxn07807	0	0
rxn07832	0	0
rxn07846	0	0
rxn07849	0	0
rxn07987	0	0
rxn07989	0	0
rxn07991	0	0
rxn07992	0	0
rxn07993	0	0
rxn07994	0	0
rxn08025	0	0
rxn08035	0	0
rxn08038	0	0
rxn08043	0	1,41422683
rxn08044	0	0
rxn08067	-1000	1000
rxn08083	0	0
rxn08084	0	0
rxn08085	0	0
rxn08086	0	0
rxn08087	0	0
rxn08088	0	0
rxn08089	0	0
rxn08094	0	1000
rxn08114	0	0
rxn08126	0	0

rxn08127	0	0
rxn08128	0	0
rxn08129	0	0
rxn08171	0	0
rxn08194	-1000	1000
rxn08294	0	0
rxn08295	0	0
rxn08296	0	0
rxn08297	0	0
rxn08298	0	0
rxn08299	0	0
rxn08300	0	0
rxn08306	0	0
rxn08307	0	0
rxn08308	0	0
rxn08309	0	0
rxn08310	0	0
rxn08311	0	0
rxn08312	0	0
rxn08352	0	0
rxn08386	0	0
rxn08390	0	0
rxn08392	0	0
rxn08394	0	0
rxn08396	0	0
rxn08398	0	0
rxn08433	0	0
rxn08438	0	0
rxn08448	0	0
rxn08449	0	0
rxn08451	0	0
rxn08453	0	0
rxn08454	0	1000
rxn08455	0	0
rxn08456	0	1000
rxn08457	0	0
rxn08519	0,045756553	0,045756581
rxn08546	0	0
rxn08547	0	1000
rxn08548	0	0
rxn08549	0	0
rxn08550	0	0
rxn08551	0	0
rxn08552	0	0
rxn08571	0	1000
rxn08582	0	0,5
rxn08605	0	0
rxn08607	0	0
rxn08615	-1000	1000
rxn08647	0	0

rxn08668	0	0
rxn08669	0	0
rxn08733	-1000	0
rxn08764	0	1,41422683
rxn08796	0	0
rxn08797	0	1000
rxn08798	0	0
rxn08799	0	1000
rxn08800	0	0
rxn08801	0	1000
rxn08802	0	0
rxn08803	0	0
rxn08804	0	0
rxn08805	0	0
rxn08806	0	0
rxn08807	0	0
rxn08808	0	0
rxn08809	0	0
rxn08810	0	0
rxn08811	0	0
rxn08812	0	0
rxn08813	0	0
rxn08814	0	0
rxn08815	0	0
rxn08816	0	0
rxn08817	0	0
rxn08818	0	0
rxn08819	0	0
rxn08820	0	0
rxn08821	0	0
rxn08822	0	0
rxn08823	0	0
rxn08838	0	0
rxn08839	0	0
rxn08840	0	0
rxn08841	0	0
rxn08842	0	0
rxn08843	0	0
rxn08844	0	0
rxn08845	0	0
rxn08846	0	0
rxn08847	0	0
rxn08848	0	0
rxn08849	0	0
rxn08850	0	0
rxn08851	0	0
rxn08857	0	0
rxn08889	0,000610753	0,000610753
rxn08890	0,004944104	0,004944107
rxn08891	0,000610753	0,000610753

rxn08892	-999,988403	1000
rxn08893	-999,9938961	999,9945069
rxn08894	-999,988403	1000
rxn08897	-0,005493149	-0,005493146
rxn08926	0,000549042	0,000549043
rxn08927	-999,9987794	999,9896236
rxn08928	-999,988403	1000
rxn08929	0,001220598	0,001220599
rxn08930	0	0
rxn08958	0,000610753	0,000610753
rxn09010	0	0
rxn09011	0	0
rxn09012	0	0
rxn09016	0	999,8102401
rxn09108	0	0
rxn09109	0	0
rxn09110	0	0
rxn09111	0	0
rxn09112	0	0
rxn09113	0	0
rxn09114	0	0
rxn09176	-1000	1000
rxn09177	0	0,000522725
rxn09205	0	0
rxn09206	0	0
rxn09207	0	0
rxn09208	0	0
rxn09209	0	0
rxn09210	0	0
rxn09211	0	0
rxn09235	0,022515273	0,022515287
rxn09237	0,02324128	0,023241294
rxn09340	0	0
rxn09341	0	999,6817696
rxn09348	0	999,706796
rxn09355	0	0
rxn09398	-999,706796	0
rxn09399	0	0
rxn09412	-1000	1000
rxn09445	0	0
rxn09446	0	0
rxn09447	0	0
rxn09486	-999,9499473	1000
rxn09502	0	1000
rxn09516	0	1000
rxn09519	0	0
rxn09557	0,000202374	1000
rxn09616	0,000549042	0,000549043
rxn09631	0,000202374	0,000202374
rxn09632	0	999,9997976

rxn09633	0,000202374	0,000202374
rxn09888	0	0
rxn09889	0	0
rxn09952	0	0
rxn09978	0	0
rxn09979	0	0
rxn09988	0	0
rxn09992	0	0
rxn09995	0	0
rxn10003	0	0,000522725
rxn10019	0	0
rxn10020	0	0
rxn10021	0	0
rxn10052	-1000	1000
rxn10054	0	999,6817696
rxn10056	0	0,000405656
rxn10058	0	0,000405656
rxn10060	0	0,000405656
rxn10091	-1000	1000
rxn10111	0	0
rxn10192	0	0
rxn10193	0	0
rxn10194	0	0
rxn10196	0	0
rxn10202	0	1000
rxn10203	0	1000
rxn10204	0	1000
rxn10205	0	0
rxn10206	0	0
rxn10207	0	0
rxn10208	0	0
rxn10209	0	0
rxn10210	0	0
rxn10211	0	0
rxn10212	0	0
rxn10213	0	0
rxn10214	0	0
rxn10215	0	0
rxn10216	0	0
rxn10217	0	0
rxn10218	0	0
rxn10219	0	0
rxn10220	0	0
rxn10221	0	0
rxn10222	0	0
rxn10223	0	0
rxn10224	0	0
rxn10225	0	0
rxn10226	0	0
rxn10227	0	0

rxn10228	0	0
rxn10229	0	0
rxn10230	0	0
rxn10231	0	0
rxn10253	0	0
rxn10254	0	0
rxn10255	0	0
rxn10256	0	0
rxn10257	0	0
rxn10258	0	0
rxn10259	0	0
rxn10260	0	0
rxn10261	0	0
rxn10262	0	0
rxn10263	0	0
rxn10264	0	0
rxn10289	0	0
rxn10290	0	0
rxn10291	0	0
rxn10292	0	0
rxn10293	0	0
rxn10294	0	0
rxn10295	0	0
rxn10296	0	0
rxn10297	0	0
rxn10298	0	0
rxn10299	0	0
rxn10300	0	0
rxn10301	0	0
rxn10302	0	0
rxn10303	0	0
rxn10304	0	0
rxn10305	0	0
rxn10306	0	0
rxn10363	0	0
rxn10404	0	0
rxn10405	0	0
rxn10406	0	0
rxn10407	0	0
rxn10408	0	0
rxn10409	0	0
rxn10410	0	0
rxn10785	4,99129E-05	4,9913E-05
rxn10951	0	0,022515287
rxn11007	0,022515273	0,022515287
rxn11511	0	0
rxn11513	0	0
rxn11547	0	0
rxn11548	0	0
rxn11550	0	0

rxn11567	0	0
rxn11571	0	0
rxn11587	0	0
rxn11599	0	0
rxn11609	0	0
rxn11641	0	0
rxn11663	-1000	0
rxn11676	0	0
rxn11677	0	0
rxn11678	0	0
rxn11702	0	0
rxn11729	0	0
rxn11730	0	0
rxn11732	0	0
rxn11749	0	0
rxn11755	0	0
rxn11756	0	0
rxn11757	-999,9977294	0
rxn11759	0	999,9977294
rxn11760	-999,9977294	0
rxn11765	0	0
rxn11766	0	0
rxn11768	0	0
rxn11772	0	0
rxn11773	0	0
rxn11788	0	0
rxn11878	0	0
rxn11879	0	0
rxn11951	0	0
rxn11965	0	0
rxn12013	0	0
rxn12053	0	0
rxn12054	0	0
rxn12218	-1000	-0,000202374
rxn12221	0,000202374	1000
rxn12510	0,000522725	0,000522725
rxn12649	-999,9991905	0
rxn12707	0	0
rxn12778	0	0
rxn12822	-1000	0
rxn13420	0,000549042	11,70435741
rxn13421	0,000549042	11,70435741
rxn13705	0	0
rxn13741	0	0
rxn13906	-0,006120238	-0,006120234
rxn13936	0,012207798	0,012207805
rxn13974	-21,16653134	0
rxn14043	0	0
rxn14048	-1000	0
rxn14050	0	0

rxn14054	-1000	0
rxn14070	0	0
rxn14089	-1000	0
rxn14093	0	0
rxn14120	-1000	-0,000809497
rxn14132	0	0
rxn14191	0	0
rxn14250	0	0
rxn14270	0	0
rxn14279	0	0
rxn14346	0	0
rxn90002	-12,31934088	1000
rxn90003	0	0
rxn90004	0	0
rxn90005	-0,022920943	-0,022515273
rxn08173	0	500
Biomass_Bacteria	0,907508	0,907508544
t_Cl	0,004094676	0,004094679
t_Sulfate	0,00341223	0,003412232
t_Cu2+	0,002729784	0,002729786
t_Mg	0,006823553	0,006823557
t_Ca2+	0,004094676	0,004094679
t_NH3	-3,719090622	0
t_H2O	-33,56475559	10
t_Biomass	-0,907508544	-0,907508
t_Butyrate	-11,70380837	0
t_D-Lactate	-14,1110209	0
t_Ethanol	-1,137725312	0
t_Formate	-15,31528884	0
t_H2	-0,1	0,5
t_L-Lactate	-14,1110209	0
t_Nitrite	0	0
t_Phosphate	1,205948358	1,598737837
t_Propionate	-1,41422683	0
t_O2	0	0
t_D-Glucose	0	0,5
t_CO2	-15,31528884	0
t_Acetate	-21,56989173	0
t_Succinate	-7,65764442	0
t_(S,S)-2,3-Butanediol	0	0
t_H2S	-0,418718138	0
Ex_Cl	-0,004094679	-0,004094676
Ex_Sulfate	-0,003412232	-0,00341223
Ex_Cu2+	-0,002729786	-0,002729784
Ex_Mg	-0,006823557	-0,006823553
Ex_Ca2+	-0,004094679	-0,004094676
Ex_NH3	0	3,719090622
Ex_H2O	-10	33,56475559
Ex_Biomass	0,907508	0,907508544
Ex_Butyrate	0	11,70380837

Ex_D-Lactate	0	14,1110209
Ex_Ethanol	0	1,137725312
Ex_Formate	0	15,31528884
Ex_H2	-0,5	0,1
Ex_L-Lactate	0	14,1110209
Ex_Nitrite	0	0
Ex_Phosphate	-1,598737837	-1,205948358
Ex_Propionate	0	1,41422683
Ex_O2	0	0
Ex_D-Glucose	-0,5	0
Ex_CO2	0	15,31528884
Ex_Acetate	0	21,56989173
Ex_Succinate	0	7,65764442
Ex_(S,S)-2,3-Butanediol	0	0
Ex_H2S	0	0,418718138
t_Fe2	0,006343481	0,006343485
t_fe3	0,006141107	0,00614111
t_Acetaldehyde	-1,137725312	0
t_Adenosine	0	0,392788756
t_AMP	0	0,392788756
t_Amylotriose	0	0
t_BIOT	0	0
t_Choline	0	0
t_Cytidine	0	0
t_Cytosine	0	0
t_DAlanine	0	0
t_Deoxyadenosine	0	0,392788756
t_Deoxycytidine	0	0,290693137
t_Deoxyguanosine	0	0
t_Deoxyinosine	0	0
t_Deoxyuridine	0	0
t_DRibose	0	0,5
t_Glycerol	0	0
t_GSH	0	0
t_Guanine	0	0
t_H2S2O3	0	0
t_Heme	0,000202374	0,000202374
t_Homocysteine	0	0
t_HYXN	0	0,392788756
t_Inosine	0	0,392788756
t_LACT	0	0,5
t_LAlanine	0,4999997	0,5
t_LArabinose	0	0
t_LArginine	-0,429772656	0,5
t_LAsparagine	-1,359545311	0,5
t_LAspartate	-3,219090622	0,5
t_LCysteine	0,081281862	0,5
t_LGlutamate	-3,219090622	0,5
t_LGlutamine	-1,359545311	0,5
t_LHistidine	0,083581487	0,083581537

t_LInositol	0	0
t_LIsoleucine	-1,158037168	0,256189662
t_LLeucine	0,397307002	0,397307241
t_LLysine	-1,556982144	0,302563349
t_LMethionine	-0,282620071	0,136098149
t_LPhenylalanine	-3,555739182	0,163351538
t_LThreonine	-0,914024456	0,5
t_LTryptophan	0,050121667	0,050121697
t_LTyrosine	-3,59748455	0,121606145
t_LValine	-3,345288077	0,373802769
t_Maltose	0	0,5
t_Niacin	0	0,002068212
t_Ornithine	0	0
t_PPi	0	0
t_Pyridoxol	0	0
t_XAN	0	0
t_1,3Propanediol	0	0
t_5Deoxyadenosine	0	0
t_Acetoacetate	-11,70380837	0
t_BET	0	0
t_Calomide	0	0
t_Cbl	0	0
t_Citrate	0	0
t_CysGly	0	0
t_Dulcose	0	0
t_Glycine	-3,219090622	0,5
t_Glycolaldehyde	0	0
t_LProline	0,194932718	0,194932835
t_Maltohexaose	0	0
t_Methanol	0	0
t_NAcetylDglucosamine	0	0
t_PM	0	0
t_Putrescine	0	0
t_Pyridoxal	0,000202374	0,000202374
t_Riboflavin	0,000404749	0,000404749
t_Salicin	0	0
t_Sorbitol	0	0
t_Spermidine	0	0
t_Sucrose	0	0,5
t_Thiamin	0	0
t_Thyminose	0	0,5
t_TRHL	0	0
t_Uracil	0	0,290693137
t_Uridine	0	0,290693137
t_Ursin	0	0
t_Mn2+	0,002729784	0,002729786
t_Formaldehyde	0	0
t_Fumarate	-7,65764442	0
t_Oxidized glutathione	0	0
t_Adenine	0	0

t_Nicotinamide	0	0
t_Co2+	0,002729784	0,002729786
t_D-Glutamate	0	0
t_Chorismate	0	0
t_Folate	0,000809497	0,000809498
t_N-Acetyl-D-mannosamine	0	0
t_Siroheme	0	0
t_Menaquinone 7	0	0
t_2-Demethylmenaquinone 8	0	0
t_Menaquinone 8	0	0
t_Ubiquinone-8	0	0
t_2-Oxobutyrate	0	0
t_3MOP	0	0
t_Neu5Ac	0	0
t_Glycerol-3-phosphate	0	0
t_H+	-1000	0,5
t_Nicotinamide ribonucleotide	0	0
t_PAN	0,000522725	0,000522725
t_Pyridoxal phosphate	0	0
t_Zn2+	0,002729784	0,002729786
t_1,2-Diacyl-sn-glycerol dioctadecanoyl	0	0
t_meso-2,6-Diaminopimelate	0	0
t_L-Serine	-3,219090622	0,5
t_D-Fructose	0	0,5
t_D-Mannose	0	0
t_L-Rhamnose	0	0
t_beta D-Galactose	0	0,5
Ex_Fe2	-0,006343485	-0,006343481
Ex_fe3	-0,00614111	-0,006141107
Ex_Acetaldehyde	0	1,137725312
Ex_Adenosine	-0,392788756	0
Ex_AMP	-0,392788756	0
Ex_Amylotriose	0	0
Ex_BIOT	0	0
Ex_Choline	0	0
Ex_Cytidine	0	0
Ex_Cytosine	0	0
Ex_DAlanine	0	0
Ex_Deoxyadenosine	-0,392788756	0
Ex_Deoxycytidine	-0,290693137	0
Ex_Deoxyguanosine	0	0
Ex_Deoxyinosine	0	0
Ex_Deoxyuridine	0	0
Ex_DRibose	-0,5	0
Ex_Glycerol	0	0
Ex_GSH	0	0
Ex_Guanine	0	0
Ex_Heme	-0,000202374	-0,000202374
Ex_Homocysteine	0	0
Ex_HYXN	-0,392788756	0

Ex_Inosine	-0,392788756	0
Ex_LACT	-0,5	0
Ex_LAlanine	-0,5	-0,4999997
Ex_LArabinose	0	0
Ex_LArginine	-0,5	0,429772656
Ex_LAsparagine	-0,5	1,359545311
Ex_LAspartate	-0,5	3,219090622
Ex_LCysteine	-0,5	-0,081281862
Ex_LGlutamate	-0,5	3,219090622
Ex_LGlutamine	-0,5	1,359545311
Ex_LHistidine	-0,083581537	-0,083581487
Ex_LInositol	0	0
Ex_LIsoleucine	-0,256189662	1,158037168
Ex_LLeucine	-0,397307241	-0,397307002
Ex_LLysine	-0,302563349	1,556982144
Ex_LMethionine	-0,136098149	0,282620071
Ex_LPhenylalanine	-0,163351538	3,555739182
Ex_LThreonine	-0,5	0,914024456
Ex_LTryptophan	-0,050121697	-0,050121667
Ex_LTyrosine	-0,121606145	3,59748455
Ex_LValine	-0,373802769	3,345288077
Ex_Maltose	-0,5	0
Ex_Niacin	-0,002068212	0
Ex_Ornithine	0	0
Ex_PPi	0	0
Ex_XAN	0	0
Ex_5Deoxyadenosine	0	0
Ex_Acetoacetate	0	11,70380837
Ex_BET	0	0
Ex_Calomide	0	0
Ex_Cbl	0	0
Ex_Citrate	0	0
Ex_CysGly	0	0
Ex_Dulcose	0	0
Ex_Glycine	-0,5	3,219090622
Ex_Glycolaldehyde	0	0
Ex_LProline	-0,194932835	-0,194932718
Ex_Maltohexaose	0	0
Ex_Methanol	0	0
Ex_NAcetylDglucosamine	0	0
Ex_PM	0	0
Ex_Putrescine	0	0
Ex_Pyridoxal	-0,000202374	-0,000202374
Ex_Riboflavin	-0,000404749	-0,000404749
Ex_Salicin	0	0
Ex_Sorbitol	0	0
Ex_Spermidine	0	0
Ex_Sucrose	-0,5	0
Ex_Thiamin	0	0
Ex_Thyminose	-0,5	0

Ex_TRHL	0	0
Ex_Uracil	-0,290693137	0
Ex_Uridine	-0,290693137	0
Ex_Ursin	0	0
Ex_Mn2+	-0,002729786	-0,002729784
Ex_Formaldehyde	0	0
Ex_Fumarate	0	7,65764442
Ex_Oxidized glutathione	0	0
Ex_Adenine	0	0
Ex_Nicotinamide	0	0
Ex_Co2+	-0,002729786	-0,002729784
Ex_D-Glutamate	0	0
Ex_Folate	-0,000809498	-0,000809497
Ex_N-Acetyl-D-mannosamine	0	0
Ex_Siroheme	0	0
Ex_Menaquinone 7	0	0
Ex_2-Demethylmenaquinone 8	0	0
Ex_Menaquinone 8	0	0
Ex_Ubiquinone-8	0	0
Ex_Neu5Ac	0	0
Ex_H+	-0,5	1000
Ex_Nicotinamide ribonucleotide	0	0
Ex_PAN	-0,000522725	-0,000522725
Ex_Zn2+	-0,002729786	-0,002729784
Ex_1,2-Diacyl-sn-glycerol dioctadecanoyl	0	0
Ex_L-Serine	-0,5	3,219090622
Ex_D-Fructose	-0,5	0
Ex_D-Mannose	0	0
Ex_L-Rhamnose	0	0
Ex_beta D-Galactose	-0,5	0
t_Arabinan	0	0
t_Starch	0	0,005
t_octanoate	0	0
t_Melibiose	0	0,5
t_Amylose	0	0
Ex_Arabinan	0	0
Ex_Starch	-0,005	0
Ex_Melibiose	-0,5	0
Ex_Amylose	0	0
t_Raffinose_Melitose	0	0
t_Isovaleric_acid	0	0
t_H2O2	0	0
Ex_Raffinose_Melitose	0	0
Ex_Isovaleric_acid	0	0
Ex_H2O2	0	0
rxn01207_1	0	0
rxn08972	0	0
rxn08973	0	0
rxn06111	0	1000
rxn13726	0	0

rxn13727	0	0
rxn13729	0	0
rxn08974	0	0
rxn10122	0	0
rxn10123	0	0
rxn10124	0	0
rxn12665	0	0
rxn06097	0	0,005
t_Sulfite	0	0
Ex_Sulfite	0	0
t_Nitrogen	0	0,1
Ex_Nitrogen	-0,1	0

rxn ID	minFlux	max Flux
rxn00001	0	1000
rxn00003	-21,12416088	0
rxn00011	-21,12416088	0
rxn00016	0	0
rxn00020	0	1000
rxn00022	0	0,505
rxn00029	0,00101873	0,00101873
rxn00031	0	0
rxn00044	0	0
rxn00060	0,000254683	0,000254683
rxn00062	0	1000
rxn00063	0	1000
rxn00065	0	1000
rxn00070	0	0
rxn00076	0	1000
rxn00077	0	0,000510507
rxn00085	-1000	0
rxn00086	0	0
rxn00097	-1000	1000
rxn00100	0,000657835	0,000657835
rxn00103	0	1000
rxn00104	-1000	0
rxn00105	-999,9971425	1000
rxn00106	-1000	0
rxn00107	0	0
rxn00109	0	0
rxn00114	-13,63785024	9,388704261
rxn00117	-1000	1000
rxn00119	0,368989262	1000
rxn00121	-0,000254683	0
rxn00122	0	0,000254683
rxn00124	0,000254683	0,000254683
rxn00126	0,000764048	1000
rxn00127	0	0
rxn00131	-1000	1000
rxn00132	0	1000
rxn00133	0	0
rxn00134	0	1000
rxn00137	0	0
rxn00138	0	999,9997453
rxn00139	-999,9971425	0
rxn00140	0	1000
rxn00141	-999,9994906	0
rxn00142	0	0
rxn00143	0,000509365	1000
rxn00148	-13,63785024	0
rxn00151	-13,63785024	0
rxn00154	0	21,12416088
rxn00157	-21,12416088	0

rxn00159	-1000	1000
rxn00161	-1000	1000
rxn00162	0	13,63785024
rxn00165	0	7,36003722
rxn00173	0	1000
rxn00175	0	1000
rxn00176	0	0
rxn00178	-14,14552727	0
rxn00179	0	0
rxn00184	-1000	0
rxn00187	0	1000
rxn00189	0	1000
rxn00190	0	999,9997453
rxn00192	0	1000
rxn00193	0,031494975	0,031494975
rxn00196	0	0
rxn00198	0	1000
rxn00199	0	1000
rxn00202	0	0
rxn00205	0	0
rxn00206	0,000254683	204,5323262
rxn00208	0	0
rxn00209	0	0
rxn00211	0	0
rxn00212	0	999,6310107
rxn00213	-1000	1000
rxn00214	-1,5	0
rxn00216	0	1000
rxn00221	0	1000
rxn00222	0	1000
rxn00224	0,000254683	1000
rxn00225	-1000	0
rxn00226	0	0
rxn00227	0	1000
rxn00231	0	0
rxn00237	-13,66709876	1000
rxn00238	-13,63785024	0
rxn00239	0,238807673	1000
rxn00245	0	0
rxn00247	0	1000
rxn00250	-1000	999,9999646
rxn00251	-1000	0
rxn00256	-13,63785024	0
rxn00258	-1000	999,9999646
rxn00259	0	0
rxn00260	-13,07317459	0,564675656
rxn00273	0	0
rxn00274	0	0
rxn00275	-0,000254683	-0,000254683
rxn00279	0,129235949	2,619370742

rxn00283	0,027731841	0,027731841
rxn00289	0	0
rxn00290	-10,8794511	-0,000690955
rxn00293	-999,9370101	1000
rxn00295	-1000	999,9370101
rxn00297	0	999,9370101
rxn00299	0	0
rxn00301	0	999,7611923
rxn00302	0	0
rxn00303	0	0
rxn00304	-13,63785024	0
rxn00307	0	0
rxn00313	0	1,245067396
rxn00322	0	0
rxn00324	-204,5320715	0
rxn00328	0	0
rxn00333	0,000254683	204,5323262
rxn00337	0,039197122	13,67704736
rxn00340	0	1000
rxn00342	0	1000
rxn00346	0	0
rxn00347	0	13,63785024
rxn00350	-0,000254683	-0,000254683
rxn00358	0	0
rxn00359	0	0
rxn00360	0	1000
rxn00361	0	1000
rxn00362	-1000	1000
rxn00363	0	1000
rxn00364	-999,6310107	1000
rxn00365	0	1000
rxn00367	0	1000
rxn00368	0	1000
rxn00369	0	1000
rxn00371	0	1000
rxn00388	0	0
rxn00391	0	999,9997453
rxn00392	0,000254683	1000
rxn00405	0	9,560472193
rxn00409	-1000	1000
rxn00410	-999,8225103	999,8085005
rxn00411	-13,63785024	0
rxn00412	0	1000
rxn00414	0	13,63785024
rxn00416	0	1000
rxn00420	0	0
rxn00423	0	7,36003722
rxn00426	0	0
rxn00433	0	0
rxn00436	0	1000

rxn00437	0	0
rxn00438	-999,9997453	1000
rxn00440	0	1000
rxn00452	0	999,999236
rxn00453	0	1000
rxn00456	0	1000
rxn00459	-0,353278212	13,13354919
rxn00460	-13,63785024	0
rxn00461	0,031494975	0,031494975
rxn00463	0	999,6310107
rxn00469	0	1000
rxn00470	0	24,56163366
rxn00474	0	0
rxn00479	0	0
rxn00490	0	0
rxn00493	-2,490134793	0
rxn00499	-14,08277392	0
rxn00500	-14,08277392	0
rxn00506	0	1,302559388
rxn00508	0	10,8794511
rxn00509	0	10,8794511
rxn00510	0	0
rxn00512	-204,5320715	0
rxn00514	0	0
rxn00515	-1000	1000
rxn00517	-13,63785024	0
rxn00527	-2,490134793	0
rxn00533	-999,9999646	1000
rxn00540	0	0
rxn00541	-1,302559388	0
rxn00545	0	1000
rxn00547	0	1000
rxn00549	0	1000
rxn00551	0	1000
rxn00552	-0,06298995	999,9370101
rxn00553	0	1000
rxn00554	0	1000
rxn00555	0	1000
rxn00556	0	1000
rxn00557	0	1000
rxn00558	-1000	1000
rxn00559	0	0
rxn00565	0	0
rxn00566	0	1000
rxn00575	0	0,5
rxn00585	0	0
rxn00606	0	0
rxn00611	-213,8523004	0
rxn00612	-213,8523004	0
rxn00615	0	213,8523004

rxn00616	0	213,8523004
rxn00621	0	0
rxn00622	0	0
rxn00624	0	0
rxn00634	0	1000
rxn00641	0	0
rxn00642	0	0
rxn00643	-1000	1000
rxn00647	0	0
rxn00649	0	7,36003722
rxn00650	-0,000254683	-0,000254683
rxn00653	0	0
rxn00654	0	0
rxn00670	0	1000
rxn00674	0	1000
rxn00675	0	0
rxn00684	0	0
rxn00685	0	999,9989813
rxn00686	0	0
rxn00687	0	999,9989813
rxn00689	0	0
rxn00690	0	14,40879988
rxn00692	-0,260507082	7,099530138
rxn00693	0	0,397709
rxn00695	-1000	1000
rxn00698	-1000	0
rxn00701	0	1000
rxn00702	0	0
rxn00704	-1000	1,5
rxn00707	0	1000
rxn00708	0	1000
rxn00709	0	1000
rxn00710	0	0,365829146
rxn00711	-999,9971425	0
rxn00712	0	1000
rxn00713	0	1000
rxn00714	0	0
rxn00715	0	1000
rxn00726	0	1,245067396
rxn00727	0	1,245067396
rxn00729	0	0
rxn00735	0	0
rxn00737	0	14,22392912
rxn00740	0	1000
rxn00741	0	0
rxn00742	-1000	13,63810492
rxn00743	0	1000
rxn00744	0	1000
rxn00747	-6,169905969	0,483273755
rxn00748	0	0

rxn00758	0	0
rxn00762	-213,8523004	0
rxn00763	0	0
rxn00765	0	0
rxn00770	0,002857469	1000
rxn00772	0	1000
rxn00775	0	0
rxn00777	-1,897775823	0,369817268
rxn00778	-1000	1000
rxn00780	0	0
rxn00781	-1000	1000
rxn00782	-1000	1000
rxn00784	0	1,302559388
rxn00785	-0,351320618	1,928816829
rxn00786	-0,499489018	5,818330669
rxn00789	0	0
rxn00790	-0,000254683	-0,000254683
rxn00791	-1,245067396	0
rxn00792	0	0
rxn00796	0	0
rxn00797	-1000	1000
rxn00799	-13,36979405	7,348877726
rxn00800	-0,26805619	13,36979405
rxn00801	0	0
rxn00802	0	9,388704261
rxn00806	0	0
rxn00808	0	1000
rxn00809	-511,3301787	0
rxn00811	-511,3301787	0
rxn00816	0	0,5
rxn00817	0	0,5
rxn00818	0	0
rxn00819	0	0
rxn00827	0	0
rxn00829	0,000690955	0,000690955
rxn00830	6,28141E-05	6,28141E-05
rxn00831	0	999,9971425
rxn00832	0	0
rxn00834	0,268056189	1000
rxn00836	-999,9971425	0
rxn00837	-999,7319438	0
rxn00838	-0,26805619	13,36979405
rxn00851	0	1000
rxn00853	0	9,560472193
rxn00855	0	0
rxn00856	0,008138419	24,60952082
rxn00858	0	0
rxn00868	-10,87876014	0
rxn00871	0	10,87876014
rxn00872	-10,87876014	0

rxn00874	0	0
rxn00879	0	0
rxn00881	0	0
rxn00882	0	0
rxn00883	0	0
rxn00889	0	0
rxn00890	0	0
rxn00892	0	999,9370101
rxn00897	-999,9370101	0
rxn00898	0	2,490134793
rxn00902	0	0
rxn00903	-2,490134793	0
rxn00907	-14,40829051	0,000509365
rxn00908	-8,051979605	0,080121065
rxn00909	-213,5925442	0,397963683
rxn00910	-213,8523004	0
rxn00913	0	1000
rxn00915	-999,9971425	0
rxn00916	-999,7319438	1000
rxn00917	0	1000
rxn00918	0	0
rxn00926	0	13,63785024
rxn00927	-1000	1000
rxn00929	-1000	1000
rxn00931	-1000	1000
rxn00938	0	999,9971425
rxn00942	0	1000
rxn00947	0	1000
rxn00950	-1000	0,397199635
rxn00952	0	1000
rxn00955	0,000509365	1000
rxn00973	-1000	1000
rxn00974	-1000	1000
rxn00977	0	0
rxn00979	0,000254683	0,000254683
rxn00980	0	0
rxn00983	0	0
rxn00985	-1000	0
rxn00986	0	0
rxn00987	0	0
rxn00991	-0,000690955	-0,000690955
rxn01000	0	2,490134793
rxn01007	0	0
rxn01008	0	0
rxn01011	0	0
rxn01013	0	0
rxn01016	0	0
rxn01018	0	0,365829146
rxn01019	0	9,388704261
rxn01021	0	0

rxn01022	0	0
rxn01029	0	9,560472193
rxn01034	0	0
rxn01042	0	0
rxn01043	0	0
rxn01052	0	0
rxn01069	0	13,63785024
rxn01073	0	0
rxn01080	0	0
rxn01089	0	0
rxn01100	-1000	0
rxn01101	0	0
rxn01103	0	1000
rxn01106	-13,13354919	0,353278212
rxn01114	0	0
rxn01115	0	0
rxn01116	-1,897775823	0,369817268
rxn01119	0	0
rxn01122	0	0
rxn01123	0	0
rxn01133	0	0
rxn01134	0	0
rxn01137	0	13,63785024
rxn01138	-1000	1000
rxn01139	0	0
rxn01146	0	0
rxn01153	0	0
rxn01169	0	1000
rxn01171	-1000	1000
rxn01199	0	0
rxn01200	0	1000
rxn01201	-14,14621822	-0,000690955
rxn01204	0,000690955	24,60207336
rxn01210	0	0
rxn01211	-14,4085452	0,000509365
rxn01213	6,28141E-05	6,28141E-05
rxn01226	-999,9707515	1000
rxn01228	0	0
rxn01236	-10,87876014	0
rxn01237	0	0
rxn01241	0	21,12416088
rxn01242	0	21,12416088
rxn01251	0	0
rxn01252	0	0
rxn01255	0,000254683	2,490389475
rxn01256	0	2,490134793
rxn01259	0	0
rxn01265	-999,9997453	-0,002602787
rxn01268	0	2,490134793
rxn01274	0	0

rxn01276	0	0
rxn01277	0	0
rxn01278	0	0
rxn01286	0	0
rxn01290	0	0
rxn01291	0	0
rxn01292	0	0,5
rxn01299	-1000	1000
rxn01300	0	13,63785024
rxn01301	-13,63785024	0
rxn01302	-13,63785024	0
rxn01303	0	13,63785024
rxn01304	0	6,216829198
rxn01305	0	0
rxn01308	0	0
rxn01310	-1000	0
rxn01316	0	0
rxn01321	0	0
rxn01329	0	0
rxn01332	0,000254683	2,490389475
rxn01334	0	1,059677184
rxn01343	0	1,059677184
rxn01346	0	1,059677184
rxn01347	0	1,059677184
rxn01348	0	1,059677184
rxn01351	0	1000
rxn01352	-1000	0
rxn01353	-1000	1000
rxn01354	-13,63785024	0
rxn01355	0	0
rxn01358	-1000	1000
rxn01360	0	204,5320715
rxn01361	-204,5320715	0
rxn01362	-0,365829146	0
rxn01366	-1000	1000
rxn01367	0	0
rxn01368	0	999,6310107
rxn01370	0	1000
rxn01374	0	0
rxn01377	0	0
rxn01379	0	0
rxn01380	0	0
rxn01387	-1000	0
rxn01388	-1000	1000
rxn01390	0	0
rxn01396	0	0
rxn01401	0	204,5320715
rxn01406	0	0
rxn01423	0	0
rxn01426	0	0

rxn01434	0	9,388704261
rxn01437	0	0
rxn01439	0	0
rxn01444	0	1000
rxn01445	0	1000
rxn01446	-0,029248515	-0,029248515
rxn01451	-999,999309	0
rxn01452	-999,999309	0
rxn01457	0	0
rxn01459	0	24,60181867
rxn01465	-0,365829146	0
rxn01466	6,28141E-05	6,28141E-05
rxn01476	0	0
rxn01480	0	0
rxn01484	0	999,9370101
rxn01485	-1000	-0,062989949
rxn01486	0	0
rxn01492	0	0
rxn01500	-0,000690955	-0,000690955
rxn01509	-999,9707515	1000
rxn01510	0	1000
rxn01513	0,028334856	0,028334856
rxn01517	0	1000
rxn01518	0,028334856	1000
rxn01519	0	1000
rxn01521	0	999,9716651
rxn01539	-1000	0
rxn01544	-999,9971425	0
rxn01545	-1000	1000
rxn01548	-999,9707515	1000
rxn01549	0	0
rxn01562	0	0
rxn01575	-2,490134793	0
rxn01594	0	0
rxn01601	0	0
rxn01602	0	0
rxn01603	0	0
rxn01610	0	0
rxn01615	0	0
rxn01621	0	0
rxn01626	0	0
rxn01629	-0,00203746	-0,00203746
rxn01636	-1000	24,56163366
rxn01637	-24,56163366	0
rxn01639	0	0
rxn01642	0	0
rxn01643	-13,67704736	-0,039197122
rxn01644	0,031494975	1,276562371
rxn01646	-1000	999,9971425
rxn01647	0	999,9971425

rxn01648	-1000	1000
rxn01649	-1000	1000
rxn01650	0	0
rxn01652	0	1000
rxn01653	0	1000
rxn01667	-13,63785024	0
rxn01670	0	999,9971425
rxn01675	0	0
rxn01678	0	1000
rxn01679	0	1000
rxn01682	0	0
rxn01683	-1000	1000
rxn01684	-1000	1000
rxn01686	0	0
rxn01704	0	0
rxn01706	0	1000
rxn01729	0	0
rxn01734	0	0
rxn01735	0	0
rxn01737	0	0
rxn01739	0,000254683	2,490389475
rxn01740	-2,490389475	-0,000254683
rxn01741	0	0
rxn01750	0	0
rxn01757	0	0
rxn01761	0	0
rxn01763	0	0,5
rxn01775	0	0
rxn01790	0	0
rxn01791	0	0
rxn01799	-0,028334856	0,33749429
rxn01800	0	0,365829146
rxn01807	0	0
rxn01816	-6,216829198	1000
rxn01832	0	0
rxn01834	0	0
rxn01835	0	0
rxn01842	0	0
rxn01843	0	0
rxn01851	0	24,60181867
rxn01857	0	0
rxn01858	0	13,63785024
rxn01859	-13,63785024	0,494313614
rxn01860	0	0
rxn01870	0	0
rxn01871	-21,12416088	0
rxn01879	0	0
rxn01885	0	0
rxn01892	0	0
rxn01906	0	0

rxn01917	0	24,56163366
rxn01937	0	0
rxn01946	0	0
rxn01953	-999,9370101	0
rxn01961	0	999,9971425
rxn01962	0	0
rxn01964	0	1,245067396
rxn01967	0	0
rxn01972	0,031494975	1000
rxn01973	0	0
rxn01974	0,031494975	1,276562371
rxn01977	-1000	1000
rxn01982	0	0
rxn01985	0	13,63785024
rxn01986	-0,057583371	0,802559388
rxn01987	-0,5	0
rxn01991	0	0
rxn01997	0	0
rxn01998	0	0
rxn01999	0	0
rxn02000	0	0
rxn02003	0	0
rxn02007	0	0
rxn02008	0,031494975	0,031494975
rxn02011	0,031494975	0,031494975
rxn02015	0	0
rxn02020	0	0
rxn02021	0	0
rxn02023	0	0
rxn02033	0	0
rxn02046	0	0
rxn02056	0	999,9997453
rxn02061	0	0
rxn02084	0	0
rxn02089	0	0
rxn02093	0	0
rxn02106	0	0
rxn02122	0	0
rxn02128	0	0
rxn02138	0	0
rxn02139	0	0
rxn02144	0	0
rxn02154	0	999,9973972
rxn02155	0,002602787	1000
rxn02160	0	0
rxn02161	0	0
rxn02166	0	0
rxn02167	0	999,999309
rxn02169	0	0
rxn02171	0,000690955	14,14621822

rxn02175	0,000657835	1000
rxn02176	0	999,9993422
rxn02185	-2,490134793	21,12416088
rxn02186	0	2,490134793
rxn02187	0	0
rxn02190	0	0
rxn02195	0	0
rxn02202	0	0
rxn02203	0	0
rxn02209	0	0
rxn02212	0,000254683	2,490389475
rxn02213	0,000254683	2,490389475
rxn02219	0	0
rxn02222	0	0
rxn02228	0	0
rxn02264	0,000254683	0,000254683
rxn02275	0	0
rxn02284	-0,031494975	0
rxn02285	-0,031494975	0
rxn02286	0,031494975	0,031494975
rxn02287	-999,9997453	1000
rxn02288	0	0
rxn02302	-1000	13,63759556
rxn02305	0,000254683	0,000254683
rxn02312	0	0
rxn02314	0	1000
rxn02315	0	1000
rxn02316	0	1000
rxn02317	-1000	0
rxn02318	0	0
rxn02320	0	0
rxn02321	0	0
rxn02322	0,000690955	0,000690955
rxn02339	0	0
rxn02341	0,000657835	0,000657835
rxn02342	0	21,12416088
rxn02350	0	0
rxn02351	0	0
rxn02356	-1000	1000
rxn02358	-1000	1000
rxn02373	-1000	1000
rxn02375	0	0
rxn02380	-1000	1000
rxn02400	0	999,9971425
rxn02404	0	0
rxn02405	0	0
rxn02409	0	0
rxn02432	0	0
rxn02433	0	0
rxn02438	-1000	0

rxn02440	0	1000
rxn02449	0	0
rxn02454	0	0
rxn02465	-24,56163366	0
rxn02473	0	0
rxn02476	0,000254683	2,490389475
rxn02483	0	0
rxn02484	0,000254683	0,000254683
rxn02495	0	0
rxn02504	0	0
rxn02507	0	1,245067396
rxn02508	0	1,245067396
rxn02517	0	0
rxn02518	0	0
rxn02521	0	0
rxn02522	0	0
rxn02525	0	0
rxn02571	0	0
rxn02581	0	0
rxn02596	0	0
rxn02597	0	0
rxn02632	0	0
rxn02663	0	0
rxn02716	0	0
rxn02729	0	0
rxn02749	0	0
rxn02751	0	0
rxn02760	0	1000
rxn02762	0	0
rxn02774	-999,9997453	0
rxn02775	0	0
rxn02776	0	0
rxn02789	0	0
rxn02795	0	0
rxn02796	0	0
rxn02811	0	0
rxn02821	0	0
rxn02822	0	0
rxn02832	0	0
rxn02834	0	0
rxn02835	0	0
rxn02853	0	0
rxn02875	0	0
rxn02895	0,000254683	0,000254683
rxn02897	0	0
rxn02900	0	0
rxn02914	0	0
rxn02922	0	0
rxn02928	-1000	999,968505
rxn02929	-1000	999,968505

rxn02931	0	0
rxn02936	0	0
rxn02937	0,000254683	0,000254683
rxn02943	0	0
rxn02974	0	0
rxn02986	0	0
rxn02990	0	0
rxn03004	0	0,000254683
rxn03005	-0,000254683	0
rxn03030	0,031494975	1000
rxn03039	0	0
rxn03047	0	0
rxn03052	0	0
rxn03057	0	0
rxn03062	0	0
rxn03064	0	0
rxn03066	0	0
rxn03068	0	0
rxn03084	0,000254683	0,000254683
rxn03086	-1000	-0,031494975
rxn03087	0	0
rxn03089	0	0
rxn03094	0	0
rxn03095	0	0
rxn03102	0	0
rxn03106	0	0
rxn03108	0,000254683	0,000254683
rxn03123	0	0
rxn03135	0	0
rxn03136	0	0
rxn03137	0	0
rxn03140	0	0
rxn03141	0	0
rxn03147	0	0
rxn03150	0	0
rxn03158	0	0
rxn03164	0,031494975	0,031494975
rxn03167	0	0
rxn03174	0	0
rxn03175	0	0
rxn03181	0	0
rxn03188	0	0
rxn03194	0	2,490134793
rxn03251	0	0
rxn03263	0	0
rxn03264	0	0
rxn03269	0	0
rxn03273	0	0
rxn03354	0	0
rxn03362	0	0

rxn03371	0	0
rxn03372	0	0
rxn03374	0	0
rxn03378	0	0
rxn03379	0	0
rxn03382	0	0
rxn03383	0	0
rxn03384	0	0
rxn03387	0	0
rxn03393	0	0
rxn03395	0	0
rxn03397	0	0
rxn03402	0	0
rxn03405	0	0
rxn03406	0	0
rxn03407	0	0
rxn03408	0,031494975	0,031494975
rxn03409	0	0
rxn03419	0	0
rxn03421	0	0
rxn03423	0	0
rxn03435	-2,490134793	0
rxn03436	0	2,490134793
rxn03437	0	2,490134793
rxn03445	0	0
rxn03446	0	0
rxn03462	0	0
rxn03465	0	0
rxn03467	0	0
rxn03468	0	0
rxn03483	0	0
rxn03491	0	0
rxn03492	0	0
rxn03511	0	0
rxn03512	0	0
rxn03513	0	0
rxn03514	0	0
rxn03535	0	0
rxn03536	0	0
rxn03537	0	0
rxn03538	0	0
rxn03540	0	0
rxn03552	0	0
rxn03553	0	0
rxn03558	0	0
rxn03598	0	0
rxn03599	0	0
rxn03634	0	0
rxn03638	0,062989949	1000
rxn03641	0,000690955	14,14621822

rxn03642	0,000690955	14,14621822
rxn03807	0	0
rxn03838	0	0
rxn03839	0	0
rxn03852	0	0
rxn03861	0	0
rxn03869	0	0
rxn03870	0	0
rxn03884	0	0
rxn03885	0	0
rxn03887	0	0
rxn03891	0	0
rxn03901	0,031494975	0,031494975
rxn03902	0	0
rxn03903	0	0
rxn03904	0,031494975	0,031494975
rxn03907	0	0
rxn03908	0	0
rxn03909	0	0
rxn03910	0	0
rxn03916	0	0
rxn03917	0	0
rxn03918	0	0
rxn03919	0	0
rxn03933	0	0
rxn03951	0	1000
rxn03958	0	0
rxn03962	0	0
rxn03964	0	0
rxn03974	-0,028334856	0
rxn03975	-0,028334856	0
rxn04045	0	0
rxn04046	0	0
rxn04047	0	0
rxn04048	0	0
rxn04050	0	0
rxn04052	0	0
rxn04068	0	0
rxn04082	0	0,5
rxn04113	0	0
rxn04142	0	0
rxn04162	0	0
rxn04234	0	0
rxn04285	0	0
rxn04286	0	0
rxn04288	0	0
rxn04290	0	0
rxn04308	0	0
rxn04384	0	0
rxn04385	0	0

rxn04413	0	0
rxn04417	0	0
rxn04418	0	0
rxn04432	0	0
rxn04443	0	0
rxn04453	0	0
rxn04476	0	0
rxn04480	0	0
rxn04482	0	0
rxn04673	0	0
rxn04674	0	0
rxn04676	0	1000
rxn04678	-1000	0
rxn04703	0	0
rxn04704	0	0
rxn04750	0	0
rxn04786	0,007702147	0,007702147
rxn04794	0	1000
rxn04822	0	0
rxn04840	0	0
rxn04841	0	0
rxn04865	0	0
rxn04866	0	0
rxn04943	0	0
rxn04954	-213,8523004	0
rxn05005	-1000	0
rxn05006	-1000	0
rxn05023	0	0
rxn05029	0	0
rxn05030	6,28141E-05	6,28141E-05
rxn05039	0	0
rxn05050	0	0
rxn05054	0	0
rxn05115	0	0
rxn05116	0	1000
rxn05122	0	0
rxn05234	0	0
rxn05236	0	0
rxn05239	0	0
rxn05247	0	0
rxn05248	0	0
rxn05249	0	0
rxn05250	0	0
rxn05251	0	0
rxn05252	0	0
rxn05256	0	0
rxn05269	0	0
rxn05289	0	0
rxn05322	0	0
rxn05323	0	0

rxn05324	0	0
rxn05325	0	0
rxn05326	0	0
rxn05327	0	0
rxn05328	0	0
rxn05329	0	0
rxn05330	0	0
rxn05331	0	0
rxn05332	0	0
rxn05333	0	0
rxn05334	0	0
rxn05335	0	0
rxn05336	0	0
rxn05337	0	0
rxn05338	0	0
rxn05339	0	0
rxn05340	0	0
rxn05341	0	0
rxn05342	0	0
rxn05343	0	0
rxn05344	0	0
rxn05345	0	0
rxn05346	0	0
rxn05347	0	0
rxn05348	0	0
rxn05350	0	0
rxn05457	-1000	0
rxn05465	0	0
rxn05733	0	0
rxn05736	0	1000
rxn05740	-1000	1000
rxn05759	-0,5	0
rxn05760	-14,71662405	1000
rxn05763	0	0
rxn05778	0	0
rxn05779	0	0
rxn05794	-1000	0
rxn05853	0	0
rxn05854	0	0
rxn05856	0	0
rxn05874	0	0
rxn05899	0	0
rxn05901	0	0
rxn05918	0	0
rxn05919	0	0
rxn05927	0	0
rxn05934	0	0
rxn05937	-1000	1000
rxn05938	-21,12416088	0
rxn05939	-6,216250166	1000

rxn05940	-1000	1000
rxn05957	0	1000
rxn05958	0	0
rxn05962	0	0
rxn05979	0	0
rxn05988	0	0
rxn05990	0	0
rxn05994	0	0
rxn06005	0	0
rxn06023	0	0
rxn06033	0	0
rxn06043	0	0
rxn06044	0	0
rxn06045	0	0
rxn06071	0,000509365	409,0646523
rxn06078	0	0
rxn06094	0	1000
rxn06096	-1000	0
rxn06108	-1000	0
rxn06139	0	0
rxn06140	0	0
rxn06181	0	1000
rxn06182	0	1000
rxn06190	0	0
rxn06194	0	0
rxn06195	0	0
rxn06196	0	0
rxn06197	0	0
rxn06200	0	0
rxn06201	0	0
rxn06209	0	0
rxn06217	0	0
rxn06218	0	0
rxn06219	0	0
rxn06227	0	0
rxn06231	0	0
rxn06243	0	0
rxn06244	0	0
rxn06251	0	0
rxn06252	-1000	0
rxn06253	0	1000
rxn06280	0	0
rxn06285	0	0
rxn06293	0	0
rxn06298	0	0
rxn06299	0	0
rxn06300	0	0
rxn06316	0	0
rxn06328	0	0
rxn06347	0	0

rxn06348	0	0
rxn06362	0	0
rxn06368	0	0
rxn06381	0	0
rxn06394	0	0
rxn06403	0	0
rxn06432	0	0
rxn06434	0	0
rxn06435	0	0
rxn06437	0	0
rxn06438	0	0
rxn06439	0	0
rxn06440	0	0
rxn06441	0	0
rxn06443	0	0
rxn06444	0	0
rxn06445	0	0
rxn06446	0	0
rxn06447	0	0
rxn06448	0	0
rxn06449	0	0
rxn06476	0	0
rxn06485	0	0
rxn06493	0	0
rxn06500	0	0
rxn06522	0	0
rxn06525	0	1000
rxn06526	-1000	0
rxn06538	0	0
rxn06556	0	0
rxn06565	0	0
rxn06581	0	0
rxn06584	0	0
rxn06591	0,00203746	0,00203746
rxn06592	0	0
rxn06595	0	0
rxn06600	0	0
rxn06614	0	0
rxn06624	0	0
rxn06648	0	0
rxn06664	0	0
rxn06671	0	0
rxn06673	0	0
rxn06678	0	0
rxn06694	0	0
rxn06699	0	0
rxn06701	0	0
rxn06709	0	0
rxn06726	0	0
rxn06733	0	0

rxn06737	0	0
rxn06741	0	0
rxn06751	0	0
rxn06752	0	0
rxn06760	0	0
rxn06768	0	0
rxn06799	0	0
rxn06820	0	0
rxn06823	0	0
rxn06831	0	0
rxn06850	0	0
rxn06860	0	0
rxn06864	0	0
rxn06865	0	0
rxn06882	0	0
rxn06883	0	0
rxn06887	0	0
rxn06890	0	0
rxn06936	0	0
rxn06937	0,00203746	0,00203746
rxn06947	0	0
rxn06958	-409,0646523	-0,000509365
rxn06979	0	0
rxn07056	0	0
rxn07059	0	0
rxn07099	0	0
rxn07189	0	0
rxn07193	0	0
rxn07223	0	0
rxn07241	0	0
rxn07251	0	0
rxn07257	0	0
rxn07267	0	0
rxn07292	0	0
rxn07437	0	0
rxn07441	0	999,968505
rxn07452	0	0
rxn07456	0	13,63785024
rxn07465	0,007702147	0,007702147
rxn07466	-1000	1000
rxn07476	0	0
rxn07486	0	0
rxn07487	-1000	1000
rxn07488	-1000	1000
rxn07489	0	0
rxn07573	0	0
rxn07577	0	0
rxn07578	0	0
rxn07579	0	0
rxn07580	0	0

rxn07584	0	0
rxn07585	0	0
rxn07586	0	0
rxn07587	0	0
rxn07645	0	0
rxn07679	0	0
rxn07680	0	0
rxn07683	0	0
rxn07684	0	0
rxn07687	0	0
rxn07688	0	0
rxn07846	0	0
rxn07849	0	0
rxn07987	0	0
rxn07989	0	0
rxn07991	0	0
rxn07992	0	0
rxn07993	0	0
rxn07994	0	0
rxn08035	0	0
rxn08038	0	999,9370101
rxn08040	0	0
rxn08043	0	2,490134793
rxn08044	0	0
rxn08067	-1000	1000
rxn08083	0	0
rxn08084	0	0
rxn08085	0	0
rxn08086	0	0
rxn08087	0	0
rxn08088	0	0
rxn08089	0	0
rxn08094	0	1000
rxn08126	0	0
rxn08127	0	0
rxn08128	0	0
rxn08129	0	0
rxn08133	0	0
rxn08180	0	0
rxn08194	-1000	1000
rxn08294	0	0
rxn08295	0	0
rxn08296	0	0
rxn08297	0	0
rxn08298	0	0
rxn08299	0	0
rxn08300	0	0
rxn08306	0	0
rxn08307	0	0
rxn08308	0	0

rxn08309	0	0
rxn08310	0	0
rxn08311	0	0
rxn08312	0	0
rxn08352	0	0
rxn08386	0	0
rxn08390	0	0
rxn08392	0	0
rxn08394	0	0
rxn08396	0	0
rxn08398	0	0
rxn08413	0	0
rxn08433	0	0
rxn08434	0	1000
rxn08435	0	0
rxn08436	0	0
rxn08437	0	0
rxn08438	0	0
rxn08448	0	0
rxn08449	0	0
rxn08451	0	0
rxn08453	0	0
rxn08454	0	1000
rxn08455	0	0
rxn08456	0	0
rxn08457	0	0
rxn08519	0,057583371	0,057583371
rxn08546	0	0
rxn08547	0	1000
rxn08548	0	0
rxn08549	0	0
rxn08550	0	0
rxn08551	0	0
rxn08552	0	0
rxn08571	0	1000
rxn08582	0	0,5
rxn08605	0	0
rxn08607	0	0
rxn08615	-1000	1000
rxn08647	0	0
rxn08668	0	0
rxn08669	0	0
rxn08700	0	0
rxn08713	0	0
rxn08764	0	2,490134793
rxn08796	0	0
rxn08797	0	1000
rxn08798	0	0
rxn08799	0	1000
rxn08800	0	0

rxn08801	0	1000
rxn08802	0	0
rxn08803	0	0
rxn08804	0	0
rxn08805	0	0
rxn08806	0	0
rxn08807	0	0
rxn08808	0	0
rxn08809	0	0
rxn08810	0	0
rxn08811	0	0
rxn08812	0	0
rxn08813	0	0
rxn08814	0	0
rxn08815	0	0
rxn08816	0	0
rxn08817	0	0
rxn08818	0	0
rxn08819	0	0
rxn08820	0	0
rxn08821	0	0
rxn08822	0	0
rxn08823	0	0
rxn08838	0	0
rxn08839	0	0
rxn08840	0	0
rxn08841	0	0
rxn08842	0	0
rxn08843	0	0
rxn08844	0	0
rxn08845	0	0
rxn08846	0	0
rxn08847	0	0
rxn08848	0	0
rxn08849	0	0
rxn08850	0	0
rxn08851	0	0
rxn08857	0	0
rxn08889	0,000768616	0,000768616
rxn08890	0,006222019	0,006222019
rxn08891	0,000768616	0,000768616
rxn08892	-999,9854054	1000
rxn08893	-999,9923184	999,993087
rxn08894	-999,9854054	1000
rxn08897	-0,006912974	-0,006912974
rxn08926	0,000690955	0,000690955
rxn08927	-999,9984639	999,9869415
rxn08928	-999,9854054	1000
rxn08929	0,00153609	0,00153609
rxn08930	0	0

rxn08958	0,000768616	0,000768616
rxn09010	0	0
rxn09016	0	999,7611923
rxn09069	0	0
rxn09108	0	0
rxn09109	0	0
rxn09110	0	0
rxn09111	0	0
rxn09112	0	0
rxn09113	0	0
rxn09114	0	0
rxn09176	-1000	1000
rxn09205	0	0
rxn09206	0	0
rxn09207	0	0
rxn09208	0	0
rxn09209	0	0
rxn09210	0	0
rxn09211	0	0
rxn09235	0,028334856	0,028334856
rxn09237	0,029248515	0,029248515
rxn09264	0	0
rxn09265	0	0
rxn09340	0	0
rxn09341	0	999,6310107
rxn09348	0	999,6310107
rxn09355	0	0
rxn09395	0	0
rxn09398	-1000	1000
rxn09399	0	0
rxn09402	0	0
rxn09412	-1000	1000
rxn09445	0	0
rxn09446	0	0
rxn09447	0	0
rxn09454	0	0
rxn09455	0	0
rxn09456	0	0
rxn09461	0	0
rxn09473	0	0
rxn09486	-999,9370101	1000
rxn09498	0	0
rxn09502	0	1000
rxn09519	0	0
rxn09531	0	0
rxn09557	0,000254683	1000
rxn09616	0,000690955	0,000690955
rxn09631	0,000254683	0,000254683
rxn09632	0	1000
rxn09633	0,000254683	0,000254683

rxn09888	0	0
rxn09889	0	0
rxn09949	0	0
rxn09952	0	0
rxn09978	0	0
rxn09979	0	0
rxn09988	0	0
rxn09995	0	0
rxn10003	0,000657835	0,000657835
rxn10019	0	0
rxn10020	0	0
rxn10021	0	0
rxn10052	-1000	1000
rxn10054	0	999,6310107
rxn10056	0	0,000510507
rxn10058	0	0,000510507
rxn10060	0	0,000510507
rxn10091	-1000	1000
rxn10111	0	0
rxn10191	0	0
rxn10192	0	0
rxn10193	0	0
rxn10194	0	0
rxn10196	0	0
rxn10202	0	1000
rxn10203	0	1000
rxn10204	0	1000
rxn10205	0	0
rxn10206	0	0
rxn10207	0	0
rxn10208	0	0
rxn10209	0	0
rxn10210	0	0
rxn10211	0	0
rxn10212	0	0
rxn10213	0	0
rxn10214	0	0
rxn10215	0	0
rxn10216	0	0
rxn10217	0	0
rxn10218	0	0
rxn10219	0	0
rxn10220	0	0
rxn10221	0	0
rxn10222	0	0
rxn10223	0	0
rxn10224	0	0
rxn10225	0	0
rxn10226	0	0
rxn10227	0	0

rxn10228	0	0
rxn10229	0	0
rxn10230	0	0
rxn10231	0	0
rxn10253	0	0
rxn10254	0	0
rxn10255	0	0
rxn10256	0	0
rxn10257	0	0
rxn10258	0	0
rxn10259	0	0
rxn10260	0	0
rxn10261	0	0
rxn10262	0	0
rxn10263	0	0
rxn10264	0	0
rxn10289	0	0
rxn10290	0	0
rxn10291	0	0
rxn10292	0	0
rxn10293	0	0
rxn10294	0	0
rxn10295	0	0
rxn10296	0	0
rxn10297	0	0
rxn10298	0	0
rxn10299	0	0
rxn10300	0	0
rxn10301	0	0
rxn10302	0	0
rxn10303	0	0
rxn10304	0	0
rxn10305	0	0
rxn10306	0	0
rxn10363	0	0
rxn10404	0	0
rxn10405	0	0
rxn10406	0	0
rxn10407	0	0
rxn10408	0	0
rxn10409	0	0
rxn10410	0	0
rxn10785	6,28141E-05	6,28141E-05
rxn10951	0	0,028334856
rxn11007	0,028334856	0,028334856
rxn11510	0	0
rxn11511	0	0
rxn11513	0	0
rxn11547	0	0
rxn11548	0	0

rxn11550	0	0
rxn11551	-1000	1000
rxn11552	-1000	1000
rxn11567	0	0
rxn11571	0	0
rxn11587	0	0
rxn11599	0	0
rxn11609	0	0
rxn11612	0	0
rxn11641	0	0
rxn11642	0	0
rxn11663	-1000	0
rxn11676	0	0
rxn11702	0	0
rxn11732	0	0
rxn11749	0	0
rxn11755	0	0
rxn11756	0	0
rxn11757	-999,9971425	0
rxn11759	0	999,9971425
rxn11760	-999,9971425	0
rxn11761	0	0
rxn11765	0	0
rxn11766	0	0
rxn11768	0	0
rxn11772	0	0
rxn11773	0	0
rxn11788	0	0
rxn11890	0	0
rxn11894	0	0
rxn11934	0	0
rxn11946	0	0
rxn11951	0	0
rxn11965	0	0
rxn12049	0	0
rxn12218	-1000	-0,000254683
rxn12221	0,000254683	1000
rxn12510	0,000657835	0,000657835
rxn12649	-999,9989813	0
rxn12778	0	0
rxn12822	-1000	0
rxn12844	0	0
rxn12845	0	0
rxn12846	0	0
rxn12847	0	0
rxn13420	0,000690955	1000
rxn13421	0,000690955	1000
rxn13705	0	0
rxn13741	0	0
rxn13906	-204,5397736	-0,007702147

rxn13936	0,015363179	0,01536318
rxn13974	-21,12416088	0
rxn13994	0	0
rxn14029	0	0
rxn14043	0	0
rxn14048	-1000	0
rxn14050	0	0
rxn14063	0	0
rxn14070	0	0
rxn14120	-1000	-0,00101873
rxn14132	0	0
rxn14136	0	0
rxn14146	0	0
rxn14160	0	0
rxn14178	-1000	1000
rxn14191	0	0
rxn14250	0	0
rxn14270	0	0
rxn14275	0	0
rxn14279	0	0
rxn14297	0	0
rxn14328	0	0
rxn14346	0	0
rxn90002	-13,13354919	1000
rxn90003	0	0
rxn90004	0	0
rxn90005	-0,028845363	-0,028334856
rxn08173	0	500
rxn13688	0	0
Biomass_Bacteria	1,142074	1,142074006
t_Cl	0,005153038	0,005153038
t_Sulfate	0,004294198	0,004294198
t_Cu2+	0,003435359	0,003435359
t_Mg	0,008587254	0,008587254
t_Ca2+	0,005153038	0,005153038
t_NH3	0	0
t_H2O	-32,52008513	8,695515883
t_Biomass	-1,142074006	-1,142074
t_Butyrate	-10,87876014	0
t_D-Lactate	-14,08277392	0
t_Formate	-14,40879988	0
t_H2	0	0,5
t_L-Lactate	-14,08277392	0
t_Nitrite	0	0
t_Phosphate	1,517653029	2,011966651
t_Propionate	-14,22418381	0
t_O2	0	0
t_D-Glucose	0	0,5
t_CO2	-14,40879988	0
t_Acetate	-21,75752028	0

t_Succinate	-10,87876014	0
t_(S,S)-2,3-Butanediol	0	0
t_H2S	-0,397709	0
Ex_Cl	-0,005153038	-0,005153038
Ex_Sulfate	-0,004294198	-0,004294198
Ex_Cu2+	-0,003435359	-0,003435359
Ex_Mg	-0,008587254	-0,008587254
Ex_Ca2+	-0,005153038	-0,005153038
Ex_NH3	0	0
Ex_H2O	-8,695515883	32,52008513
Ex_Biomass	1,142074	1,142074006
Ex_Butyrate	0	10,87876014
Ex_D-Lactate	0	14,08277392
Ex_Formate	0	14,40879988
Ex_H2	-0,5	0
Ex_L-Lactate	0	14,08277392
Ex_Nitrite	0	0
Ex_Phosphate	-2,011966651	-1,517653029
Ex_Propionate	0	14,22418381
Ex_O2	0	0
Ex_D-Glucose	-0,5	0
Ex_CO2	0	14,40879988
Ex_Acetate	0	21,75752028
Ex_Succinate	0	10,87876014
Ex_(S,S)-2,3-Butanediol	0	0
Ex_H2S	0	0,397709
t_Fe2	0,007983097	0,007983097
t_fe3	0,007728415	0,007728415
t_Acetaldehyde	-1,302559388	0
t_Adenosine	0	0,494313614
t_AMP	0	0,494313614
t_Amylotriose	0	0
t_BIOT	0	0
t_Choline	0	0
t_Cytidine	0	0
t_Cytosine	0	0
t_DAlanine	0	0
t_Deoxyadenosine	0	0,494313614
t_Deoxycytidine	0	0,365829146
t_Deoxyguanosine	0	0
t_Deoxyinosine	0	0
t_Deoxyuridine	0	0
t_DRibose	0	0,5
t_DSerine	0	0
t_GLUM	0	0
t_Glycerol	0	0
t_GSH	0	0
t_Guanine	0	0
t_H2S2O3	0	0
t_Heme	0,000254683	0,000254683

t_Homocysteine	0	0
t_HYXN	0	0,494313614
t_Inosine	0	0,494313614
t_LACT	0	0,5
t_LAlanine	-1,990134793	0,5
t_LArabinose	0	0,5
t_LArginine	-0,122533698	0,5
t_LAsparagine	-0,745067396	0,5
t_LAspartate	-1,990134793	0,5
t_LCysteine	0,102291	0,5
t_LGlutamate	-1,990134793	0,5
t_LGlutamine	-0,745067396	0,5
t_LHistidine	0,105185015	0,105185016
t_LInositol	0	0
t_LIsoleucine	-2,167727303	0,322407492
t_LLeucine	0,499999997	0,5
t_LLysine	-0,864299925	0,380767474
t_LMethionine	-0,226433304	0,171275697
t_LPhenylalanine	-2,284561473	0,205573321
t_LThreonine	-1,990134793	0,5
t_LTryptophan	-1,181990649	0,063076747
t_LTyrosine	-2,337096877	0,153037917
t_LValine	-2,019714512	0,470420283
t_Maltose	0	0,5
t_Niacin	0,002602787	0,002602787
t_Ornithine	0	0
t_PPi	0	0
t_Pyridoxol	0	0
t_XAN	0	0
t_5Deoxyadenosine	0	0
t_Acetoacetate	-10,87876014	0
t_Calomide	0	0
t_Cbl	0	0
t_Citrate	0	0
t_CysGly	0	0
t_Dulcose	0	0
t_Glycine	-1,990134793	0,5
t_Glycolaldehyde	0	0
t_LProline	0,245317495	0,245317497
t_Maltohexaose	0	0
t_Methanol	0	0
t_NAcetylDglucosamine	0	0
t_PM	0	0
t_Putrescine	0	0
t_Pyridoxal	0,000254683	0,000254683
t_Riboflavin	0,000509365	0,000509365
t_Salicin	0	0
t_Sorbitol	0	0
t_Spermidine	0	0
t_Sucrose	0	0,5

t_Taurine	0	0
t_Thiamin	0	0
t_Thymidine	0	0
t_Thyminose	0	0,5
t_TRHL	0	0
t_Uracil	0	0,365829146
t_Uridine	0	0,365829146
t_Ursin	0	0
t_Mn2+	0,003435359	0,003435359
t_Formaldehyde	0	0
t_Fumarate	-7,20439994	0
t_Oxidized glutathione	0	0
t_Adenine	0	0
t_Nicotinamide	0	0
t_Co2+	0,003435359	0,003435359
t_D-Glutamate	0	0
t_Chorismate	0	0
t_Folate	0,00101873	0,00101873
t_N-Acetyl-D-mannosamine	0	0
t_Siroheme	0	0
t_Menaquinone 7	0	0
t_2-Demethylmenaquinone 8	0	0
t_Menaquinone 8	0	0
t_Ubiquinone-8	0	0
t_2-Oxobutyrate	0	0
t_3MOP	0	0
t_Neu5Ac	0	0
t_Glycerol-3-phosphate	0	0
t_H+	-1000	0,5
t_indol	0	0
t_Nicotinamide ribonucleotide	0	0
t_PAN	0,000657835	0,000657835
t_Pyridoxal phosphate	0	0
t_Zn2+	0,003435359	0,003435359
t_1,2-Diacyl-sn-glycerol dioctadecanoyl	0	0
t_meso-2,6-Diaminopimelate	0	0
t_L-Serine	-1,990134793	0,5
t_D-Fructose	0	0,5
t_D-Mannose	0	0
t_L-Rhamnose	0	0
t_beta D-Galactose	0	0,5
t_L-Fucose	0	0
Ex_Fe2	-0,007983097	-0,007983097
Ex_fe3	-0,007728415	-0,007728415
Ex_Acetaldehyde	0	1,302559388
Ex_Adenosine	-0,494313614	0
Ex_AMP	-0,494313614	0
Ex_Amylotriose	0	0
Ex_BIOT	0	0
Ex_Choline	0	0

Ex_Cytidine	0	0
Ex_Cytosine	0	0
Ex_DAlanine	0	0
Ex_Deoxyadenosine	-0,494313614	0
Ex_Deoxycytidine	-0,365829146	0
Ex_Deoxyguanosine	0	0
Ex_Deoxyinosine	0	0
Ex_Deoxyuridine	0	0
Ex_DRibose	-0,5	0
Ex_DSerine	0	0
Ex_GLUM	0	0
Ex_Glycerol	0	0
Ex_GSH	0	0
Ex_Guanine	0	0
Ex_Heme	-0,000254683	-0,000254683
Ex_Homocysteine	0	0
Ex_HYXN	-0,494313614	0
Ex_Inosine	-0,494313614	0
Ex_LACT	-0,5	0
Ex_LAlanine	-0,5	1,990134793
Ex_LArabinose	-0,5	0
Ex_LArginine	-0,5	0,122533698
Ex_LAsparagine	-0,5	0,745067396
Ex_LAspartate	-0,5	1,990134793
Ex_LCysteine	-0,5	-0,102291
Ex_LGlutamate	-0,5	1,990134793
Ex_LGlutamine	-0,5	0,745067396
Ex_LHistidine	-0,105185016	-0,105185015
Ex_LInositol	0	0
Ex_LIsoleucine	-0,322407492	2,167727303
Ex_LLeucine	-0,5	-0,499999997
Ex_LLysine	-0,380767474	0,864299925
Ex_LMethionine	-0,171275697	0,226433304
Ex_LPhenylalanine	-0,205573321	2,284561473
Ex_LThreonine	-0,5	1,990134793
Ex_LTryptophan	-0,063076747	1,181990649
Ex_LTyrosine	-0,153037917	2,337096877
Ex_LValine	-0,470420283	2,019714512
Ex_Maltose	-0,5	0
Ex_Niacin	-0,002602787	-0,002602787
Ex_Ornithine	0	0
Ex_PPi	0	0
Ex_XAN	0	0
Ex_5Deoxyadenosine	0	0
Ex_Acetoacetate	0	10,87876014
Ex_Calomide	0	0
Ex_Cbl	0	0
Ex_Citrate	0	0
Ex_CysGly	0	0
Ex_Dulcose	0	0

Ex_Glycine	-0,5	1,990134793
Ex_Glycolaldehyde	0	0
Ex_LProline	-0,245317497	-0,245317495
Ex_Maltohexaose	0	0
Ex_Methanol	0	0
Ex_NAcetylDglucosamine	0	0
Ex_PM	0	0
Ex_Putrescine	0	0
Ex_Pyridoxal	-0,000254683	-0,000254683
Ex_Riboflavin	-0,000509365	-0,000509365
Ex_Salicin	0	0
Ex_Sorbitol	0	0
Ex_Spermidine	0	0
Ex_Sucrose	-0,5	0
Ex_Taurine	0	0
Ex_Thiamin	0	0
Ex_Thymidine	0	0
Ex_Thyminose	-0,5	0
Ex_TRHL	0	0
Ex_Uracil	-0,365829146	0
Ex_Uridine	-0,365829146	0
Ex_Ursin	0	0
Ex_Mn2+	-0,003435359	-0,003435359
Ex_Formaldehyde	0	0
Ex_Fumarate	0	7,20439994
Ex_Oxidized glutathione	0	0
Ex_Adenine	0	0
Ex_Nicotinamide	0	0
Ex_Co2+	-0,003435359	-0,003435359
Ex_D-Glutamate	0	0
Ex_Folate	-0,00101873	-0,00101873
Ex_N-Acetyl-D-mannosamine	0	0
Ex_Siroheme	0	0
Ex_Menaquinone 7	0	0
Ex_2-Demethylmenaquinone 8	0	0
Ex_Menaquinone 8	0	0
Ex_Ubiquinone-8	0	0
Ex_Neu5Ac	0	0
Ex_H+	-0,5	1000
Ex_indol	0	0
Ex_Nicotinamide ribonucleotide	0	0
Ex_PAN	-0,000657835	-0,000657835
Ex_Zn2+	-0,003435359	-0,003435359
Ex_1,2-Diacyl-sn-glycerol dioctadecanoyl	0	0
Ex_L-Serine	-0,5	1,990134793
Ex_D-Fructose	-0,5	0
Ex_D-Mannose	0	0
Ex_L-Rhamnose	0	0
Ex_beta D-Galactose	-0,5	0
Ex_L-Fucose	0	0

t_Arabinan	0	0
t_Starch	0	0,005
t_octanoate	0	0
t_Melibiose	0	0,5
t_Amylose	0	0
Ex_Arabinan	0	0
Ex_Starch	-0,005	0
Ex_Melibiose	-0,5	0
Ex_Amylose	0	0
t_Raffinose_Melitose	0	0
t_Isovaleric_acid	0	0
t_H2O2	0	0
Ex_Raffinose_Melitose	0	0
Ex_Isovaleric_acid	0	0
Ex_H2O2	0	0
rxn01207_1	0	0
rxn08972	0	0
rxn08973	0	0
rxn06111	0	1000
rxn13726	0	0
rxn13727	0	0
rxn13729	0	0
rxn08974	0	0
rxn10122	0	0
rxn10123	0	0
rxn10124	0	0
rxn12665	0	0
rxn06097	0	0,005
t_Sulfite	0	0
Ex_Sulfite	0	0

rxn ID	minFlux	max Flux
rxn00001	0	1000
rxn00003	-2,031365954	0
rxn00011	-2,031365954	0
rxn00016	0	0
rxn00020	0	1000
rxn00022	0	0,505
rxn00029	0,00101873	0,00101873
rxn00048	0	0,000509365
rxn00060	0,000254683	0,000254683
rxn00062	0	1000
rxn00065	0	0
rxn00067	0	0
rxn00076	0	1000
rxn00077	0	0,000510507
rxn00085	-1000	0
rxn00097	-1000	1000
rxn00100	0,000657835	0,000657835
rxn00101	0	14,43615903
rxn00103	0	1000
rxn00104	-1000	0
rxn00105	-999,9973972	1000
rxn00106	-1000	0
rxn00109	0	0
rxn00114	-1000	14,61720574
rxn00119	0,368989262	999,968505
rxn00122	0,000254683	0,000254683
rxn00124	0,000254683	0,000254683
rxn00126	0,000764048	0,000764048
rxn00131	-1000	999,9966332
rxn00132	0	1000
rxn00133	0	0
rxn00137	0	0
rxn00139	-999,9971425	0
rxn00142	0	0
rxn00143	0,000509365	0,000509365
rxn00144	0	0
rxn00148	-1000	0
rxn00151	-1000	0
rxn00154	0	14,23516143
rxn00157	-14,23516143	0
rxn00159	-1000	1000
rxn00161	-1000	1000
rxn00162	0	1000
rxn00165	0	0,975809746
rxn00171	0	0
rxn00172	-1000	1000
rxn00173	-21,35274214	1000
rxn00178	-14,3900238	0
rxn00179	0	0

rxn00184	-1000	0
rxn00187	0	1000
rxn00189	0	1000
rxn00190	0,002602787	1000
rxn00191	-1000	999,8707641
rxn00192	0	1000
rxn00193	0,031494975	0,031494975
rxn00196	0	0
rxn00198	0	1000
rxn00199	0	1000
rxn00200	0	0
rxn00206	0,000254683	335,0104805
rxn00213	0	999,5995158
rxn00214	-1,5	0
rxn00216	0	1000
rxn00221	0	1000
rxn00222	0	1000
rxn00224	0,000254683	1000
rxn00225	-1000	0
rxn00227	0	1000
rxn00239	0,238807673	1000
rxn00245	0	0
rxn00247	0	1000
rxn00248	-1,076429882	1000
rxn00250	-1000	999,9999646
rxn00254	0	0
rxn00256	-14,23516143	0
rxn00258	-1000	999,9999646
rxn00260	-1000	1,257181366
rxn00262	0	335,0102259
rxn00270	0	0
rxn00275	-0,895943364	-0,000254683
rxn00283	0,027731841	0,027731841
rxn00293	0,062989949	999,6625057
rxn00299	0	0,895688681
rxn00300	0	0,000509365
rxn00301	0	999,7611923
rxn00302	0	0,895688681
rxn00303	0	0
rxn00304	-1000	0
rxn00307	0	0
rxn00313	0	1,015682977
rxn00322	0	0
rxn00333	0,000254683	0,895943364
rxn00337	0,039197122	1,054880099
rxn00338	0	0,002602787
rxn00340	0	1000
rxn00342	0	1000
rxn00346	0	0
rxn00347	0	1000

rxn00350	-0,000254683	-0,000254683
rxn00358	0	0
rxn00360	0	1000
rxn00361	0	1000
rxn00362	0	0
rxn00363	0	1000
rxn00364	-999,5995158	0,000657835
rxn00365	0	1000
rxn00368	0	999,5995158
rxn00369	0	1000
rxn00371	0	1000
rxn00379	0	0,095705802
rxn00391	0	999,9997453
rxn00392	0,000254683	1000
rxn00405	0	14,43615903
rxn00407	0	1000
rxn00410	-999,8225103	1000
rxn00411	-1000	0
rxn00412	0	1000
rxn00414	0	1000
rxn00416	0	1000
rxn00420	0	0
rxn00423	0	0,975809746
rxn00426	0	0
rxn00436	0	999,9997453
rxn00437	0	0
rxn00440	0,000254683	1000
rxn00453	0	999,9997453
rxn00456	0	999,9997453
rxn00459	-0,710695536	13,52446589
rxn00460	-1000	-0,400484237
rxn00461	0,031494975	0,031494975
rxn00469	0	1000
rxn00470	0	14,43615903
rxn00471	0	2,031365954
rxn00474	0	0
rxn00490	0	2,031365954
rxn00493	-2,031365954	0
rxn00499	-14,23516143	0
rxn00500	-14,23516143	0
rxn00506	0	0
rxn00510	0	0
rxn00514	0	0
rxn00517	-1000	0
rxn00527	-2,031365954	0
rxn00533	-1000	1000
rxn00541	0	0
rxn00543	0	0
rxn00545	0	1000
rxn00547	0	1

rxn00548	0	21,35274214
rxn00549	0	1000
rxn00551	0	1000
rxn00552	-0,06298995	999,9370101
rxn00554	0	1000
rxn00555	0	1000
rxn00556	0	999,5995158
rxn00557	0	1000
rxn00558	-1000	1000
rxn00560	0	0
rxn00562	0	0
rxn00565	0	0
rxn00566	0	1,373009382
rxn00575	0	0,5
rxn00585	0	0
rxn00598	0	0
rxn00606	0	0
rxn00611	0	0
rxn00612	0	0
rxn00615	0	0
rxn00621	0	0
rxn00622	0	0
rxn00623	-0,195705802	0
rxn00634	0	0
rxn00646	0	0
rxn00647	0	0
rxn00649	0	0,975809746
rxn00650	-0,000254683	-0,000254683
rxn00653	0	0
rxn00659	0	0
rxn00661	0	0
rxn00669	-0,218224076	1000
rxn00670	0	1000
rxn00676	0	0
rxn00684	-0,895688681	0
rxn00685	0	1000
rxn00686	-0,895688681	0
rxn00687	0	1000
rxn00689	0	0
rxn00690	0	0,890648333
rxn00692	-0,260507082	0,715302664
rxn00693	0,000509365	0,218733442
rxn00695	-1000	1000
rxn00698	-1000	0
rxn00701	0	1000
rxn00704	-1000	1,5
rxn00707	0	1000
rxn00708	0	1000
rxn00709	0	1000
rxn00710	0	0

rxn00711	-999,9971425	0
rxn00712	0	999,5995158
rxn00713	0	1000
rxn00714	0	0
rxn00715	0	1000
rxn00726	0	0,585485848
rxn00727	0	0,585485848
rxn00735	0	0
rxn00737	0,000254683	0,218478759
rxn00741	0	0
rxn00742	-1000	-0,000254683
rxn00743	0	0
rxn00747	-6,503239303	7,731922123
rxn00748	0	0
rxn00758	0	0
rxn00763	0	0
rxn00765	0	0
rxn00768	0	0
rxn00769	0	0
rxn00770	0,002857469	1000
rxn00772	0	1000
rxn00775	0	0
rxn00777	-1,480430192	7,154064648
rxn00778	-1000	1000
rxn00781	-0,710695536	13,52446589
rxn00785	-14,75314871	6,599593428
rxn00786	-1000	6,503239303
rxn00789	-0,72729634	0
rxn00790	-0,000254683	-0,000254683
rxn00791	-0,585485848	0
rxn00792	0	0
rxn00796	0	0
rxn00799	-1000	7,87682323
rxn00800	-0,831325949	506,624653
rxn00802	0	14,61720574
rxn00806	0	0
rxn00808	0	1000
rxn00816	0	0,5
rxn00817	0	0,5
rxn00818	0	0
rxn00819	0	0
rxn00829	0,000690955	0,000690955
rxn00830	6,28141E-05	6,28141E-05
rxn00831	0	999,9971425
rxn00832	-0,72729634	0
rxn00834	-999,7290863	1000
rxn00836	-999,9971425	0
rxn00838	-0,831325949	506,624653
rxn00851	0	1000
rxn00853	0	14,43615903

rxn00855	0	0
rxn00856	0,008138419	14,39816222
rxn00858	0	14,43615903
rxn00859	0	1000
rxn00863	0	1000
rxn00864	0	0
rxn00867	0	0
rxn00868	-8,670345255	0
rxn00869	0	0
rxn00874	0	0
rxn00875	0	8,670345255
rxn00879	0	0
rxn00881	0	0
rxn00882	0	0
rxn00883	0	0
rxn00889	0	0
rxn00890	0	0
rxn00898	0	2,031365954
rxn00902	0	0
rxn00903	-1000	999,8707641
rxn00904	-999,8707641	1000
rxn00907	-0,890138968	0,231153496
rxn00909	-0,103407949	-0,007702147
rxn00910	-0,322396073	0
rxn00913	0	1000
rxn00915	-999,9971425	0
rxn00916	-999,7319438	1000
rxn00917	0	1000
rxn00918	0	0
rxn00921	0	0
rxn00926	0	506,8927092
rxn00927	-1000	1000
rxn00929	-1000	1000
rxn00931	-1000	1000
rxn00938	0	999,9971425
rxn00942	0	1000
rxn00943	0	1000
rxn00947	0	1000
rxn00952	0	0,218224076
rxn00955	0,000509365	0,000509365
rxn00973	-1000	1000
rxn00974	-1000	1000
rxn00977	0	0
rxn00979	0,000254683	0,895943364
rxn00980	0	0
rxn00983	0	0
rxn00985	-1000	0
rxn00990	0,000690955	8,671036209
rxn00991	-0,000690955	-0,000690955
rxn00999	0	0

rxn01000	0	2,031365954
rxn01016	0	0
rxn01018	0	0
rxn01019	0	14,61720574
rxn01021	0	0
rxn01029	0	14,43615903
rxn01034	0	0
rxn01042	0	0
rxn01068	0	0
rxn01069	0	0
rxn01073	0	0
rxn01089	0	0
rxn01094	0	0
rxn01100	-1000	0
rxn01101	0	0
rxn01103	0	1000
rxn01106	-13,52446589	0,710695536
rxn01108	-1000	1000
rxn01109	-1000	1000
rxn01114	0	0,5
rxn01116	-1,314612467	7,654064648
rxn01119	0	0
rxn01123	0	0
rxn01133	0	0
rxn01138	-1000	1000
rxn01139	0	0
rxn01152	0	0,5
rxn01169	0	1000
rxn01171	-1000	1000
rxn01187	0	21,35274214
rxn01199	0	0
rxn01200	0	1000
rxn01201	-14,39071476	-0,000690955
rxn01204	0,000690955	14,39071476
rxn01210	0	0
rxn01211	-0,89039365	0,231153496
rxn01213	6,28141E-05	6,28141E-05
rxn01225	0	999,9971425
rxn01226	-999,9707515	1000
rxn01228	0	0
rxn01237	0	0
rxn01255	0,000254683	2,031620636
rxn01256	0	2,031365954
rxn01257	0	0,895688681
rxn01259	0	0
rxn01261	0	0
rxn01265	-999,9997453	0
rxn01268	0	2,031365954
rxn01270	-2,031365954	0
rxn01274	0	0

rxn01276	0	0
rxn01278	0	0
rxn01286	0	0
rxn01292	0	0,5
rxn01297	-999,7319438	999,9971425
rxn01299	-1000	1000
rxn01300	0	0
rxn01303	0	0
rxn01304	0	0
rxn01305	0	0
rxn01308	0	0
rxn01321	0	0
rxn01322	0	0
rxn01329	0	0
rxn01332	0,000254683	2,031620636
rxn01333	-1000	1,175999707
rxn01334	0	1000
rxn01343	0	1000
rxn01344	0	0
rxn01346	0	1000
rxn01347	0	999,5995158
rxn01348	0	1000
rxn01351	0	1000
rxn01352	-1000	-0,029248515
rxn01354	-1000	0
rxn01355	0	0
rxn01358	-1000	1000
rxn01361	0	0
rxn01362	0	0
rxn01366	-0,33749429	1000
rxn01367	0	0
rxn01368	0	999,5995158
rxn01370	0	1000
rxn01374	0	0
rxn01380	0	0
rxn01387	-1000	0
rxn01388	-1000	1000
rxn01390	0	0
rxn01392	0	0
rxn01396	0	0
rxn01406	0	0
rxn01416	0	0
rxn01423	0	0
rxn01426	0	0
rxn01434	0	14,61720574
rxn01445	0	999,9707515
rxn01446	-0,029248515	-0,029248515
rxn01452	-999,999309	0
rxn01453	0	0
rxn01459	0	14,39046008

rxn01463	0	0
rxn01465	0	0
rxn01466	6,28141E-05	6,28141E-05
rxn01476	0	0
rxn01484	0	0
rxn01485	-0,06298995	-0,062989949
rxn01486	0	0
rxn01492	0	0
rxn01500	-0,000690955	-0,000690955
rxn01506	0	0
rxn01509	-999,9707515	1000
rxn01510	0	1000
rxn01513	0,028334856	0,028334856
rxn01518	0,028334856	1000
rxn01519	0	0
rxn01521	0	999,9716651
rxn01522	0	0
rxn01539	-1000	-0,000254683
rxn01544	-999,9971425	0
rxn01545	-1000	1000
rxn01548	-999,9707515	1000
rxn01549	0	0
rxn01562	0	0
rxn01575	-0,218224076	0
rxn01597	0	0
rxn01601	0	0,895688681
rxn01602	0	0,895688681
rxn01603	0	0,895688681
rxn01610	0	0
rxn01615	0	0
rxn01620	0	0
rxn01621	0	0
rxn01626	0	0
rxn01629	-0,00203746	-0,00203746
rxn01636	-1000	14,61720574
rxn01637	-14,61720574	0
rxn01643	-1,054880099	-0,039197122
rxn01644	0,031494975	1,047177952
rxn01646	-1000	999,9971425
rxn01647	0	999,9971425
rxn01649	-1000	1000
rxn01653	0	0
rxn01654	0	0
rxn01667	0	0
rxn01669	0	999,9973972
rxn01670	0	999,9971425
rxn01675	0	0
rxn01679	0	0
rxn01682	0	0
rxn01683	-1000	1000

rxn01684	-1000	1000
rxn01686	0	0
rxn01704	0	0
rxn01706	0	0
rxn01709	0	0
rxn01710	0	0
rxn01735	0	0
rxn01737	0	0
rxn01739	0,000254683	2,031620636
rxn01740	-2,031620636	-0,000254683
rxn01741	0	0
rxn01748	0	0
rxn01757	0	0
rxn01761	0	0
rxn01763	0	0,5
rxn01775	0	0
rxn01790	0	0
rxn01791	0	0
rxn01799	-0,028334856	0,029248515
rxn01800	0	0,057583371
rxn01807	0	0
rxn01827	0	0
rxn01834	0	0
rxn01842	0	0
rxn01851	0	14,39046008
rxn01855	0	0
rxn01857	0	0
rxn01859	0	0,057583371
rxn01860	0	0
rxn01870	0	0
rxn01871	0	0
rxn01885	0	0
rxn01896	0	0
rxn01906	0	0
rxn01917	0	14,61720574
rxn01937	0	0
rxn01946	0	0
rxn01961	0	999,9971425
rxn01962	0	0
rxn01964	0	0,585485848
rxn01966	0	0
rxn01967	0	0
rxn01972	0,031494975	1000
rxn01973	0	0
rxn01974	0,031494975	1,047177952
rxn01977	-1000	1000
rxn01982	0	0
rxn01983	0	0
rxn01985	0	0
rxn01986	-0,057583371	0

rxn01987	-0,057583371	0
rxn01991	0	0
rxn01997	0	0
rxn02000	0	0
rxn02003	0	0
rxn02008	0,031494975	0,031494975
rxn02011	0,031494975	0,031494975
rxn02012	0	0
rxn02015	0	0
rxn02020	0	0
rxn02023	0	0
rxn02046	0	0
rxn02056	0	999,9997453
rxn02061	0	0
rxn02084	0	0
rxn02085	0	0
rxn02093	0	0
rxn02102	-1000	0
rxn02103	0	1000
rxn02106	0	0
rxn02118	0	0
rxn02122	0	0
rxn02123	0	0
rxn02128	0	0
rxn02138	0	0
rxn02139	0	0
rxn02154	0	999,9973972
rxn02155	0,002602787	1000
rxn02159	-1000	0
rxn02160	0	0,72729634
rxn02161	0	0
rxn02167	0	999,999309
rxn02171	0,000690955	14,39071476
rxn02175	0,000657835	0,000657835
rxn02185	-2,031365954	0,218224076
rxn02186	0	2,031365954
rxn02187	0	0
rxn02190	0	0
rxn02195	0	0
rxn02200	0	0,895688681
rxn02201	0	0,895688681
rxn02209	0	0
rxn02212	0,000254683	2,031620636
rxn02213	0,000254683	2,031620636
rxn02222	0	0
rxn02228	0	0
rxn02236	0	0
rxn02262	0	0
rxn02263	0	0
rxn02264	0,000254683	0,000254683

rxn02277	0	0
rxn02284	-0,031494975	0
rxn02285	-0,031494975	0
rxn02286	0,031494975	0,031494975
rxn02287	-999,9997453	1000
rxn02288	0	0
rxn02302	-1000	-0,000254683
rxn02305	0,000254683	0,000254683
rxn02312	0	0
rxn02314	0	1000
rxn02315	0	1000
rxn02316	0	999,5995158
rxn02317	-1000	0
rxn02320	-0,72729634	0
rxn02322	0,000690955	0,000690955
rxn02339	0	0
rxn02341	0,000657835	0,000657835
rxn02350	0	0
rxn02351	0	0
rxn02352	0	0
rxn02356	-1000	1000
rxn02358	-1000	1000
rxn02373	-1000	1000
rxn02380	-1000	1000
rxn02400	0	999,9971425
rxn02402	-0,002602787	0
rxn02405	0	0
rxn02409	0	0
rxn02415	0	0
rxn02449	0	0
rxn02450	0	0
rxn02452	0	0
rxn02454	0	0
rxn02465	-14,61720574	0
rxn02466	0	0
rxn02473	0	0,72729634
rxn02474	-0,000509365	0
rxn02475	0	0,000509365
rxn02476	0,000254683	2,031620636
rxn02483	0	0
rxn02484	0,000254683	0,000254683
rxn02495	0	0
rxn02503	0	0,895688681
rxn02504	0	0,895688681
rxn02507	0	0,585485848
rxn02508	0	0,585485848
rxn02518	0	0
rxn02521	0	0
rxn02522	0	0
rxn02569	0	0

rxn02571	0	0
rxn02581	0	0
rxn02596	0	0
rxn02597	0	0
rxn02632	0	0
rxn02680	0	0
rxn02729	0	0
rxn02740	0	0
rxn02749	0	0
rxn02751	0	0
rxn02760	0	0
rxn02762	0	0
rxn02774	-999,9997453	0
rxn02775	0	0
rxn02776	0	0
rxn02789	0	0
rxn02795	0	0
rxn02796	0	0
rxn02804	0	0
rxn02810	0	0
rxn02811	0	0
rxn02821	0	0
rxn02822	0	0
rxn02834	0	0,72729634
rxn02835	0	0,72729634
rxn02853	0	0
rxn02875	0	0
rxn02895	0,000254683	0,000254683
rxn02897	0	0
rxn02898	0	0
rxn02914	0	0
rxn02922	0	0
rxn02928	-1000	999,968505
rxn02929	-1000	999,968505
rxn02931	0	0
rxn02936	0	0
rxn02937	0,000254683	0,000254683
rxn02988	-0,002602787	0
rxn02990	0	0
rxn03004	0	0,000254683
rxn03005	-0,000254683	0
rxn03008	0	0
rxn03030	0,031494975	1000
rxn03034	0	0
rxn03036	0	0
rxn03039	0	0
rxn03047	0	0
rxn03062	0	0
rxn03063	0	0
rxn03066	0	0

rxn03068	0	0
rxn03075	0	0
rxn03080	0	0,00101873
rxn03084	0,000254683	0,000254683
rxn03086	-1000	-0,031494975
rxn03087	0	0
rxn03094	0	0
rxn03095	0	0
rxn03102	0	0
rxn03106	0	0
rxn03108	0,000254683	0,000254683
rxn03135	-0,72729634	0
rxn03136	0	0
rxn03137	0	0,72729634
rxn03140	0	0
rxn03141	0	0
rxn03147	0	0
rxn03150	0	0
rxn03159	0	0
rxn03164	0,031494975	0,031494975
rxn03167	0	0,895688681
rxn03174	-0,895688681	0
rxn03175	0	0,72729634
rxn03194	0	0,218224076
rxn03243	0	0
rxn03248	0	0
rxn03263	0	0
rxn03264	0	0
rxn03269	0	0
rxn03273	0	0
rxn03282	0	0
rxn03288	0	0
rxn03292	0	0
rxn03293	0	0
rxn03295	0	0
rxn03296	0	0
rxn03301	0	0
rxn03304	0	0
rxn03313	0	0
rxn03316	0	0
rxn03333	0	0
rxn03354	0	0
rxn03371	0	0
rxn03372	0	0
rxn03373	0	0
rxn03374	0	0
rxn03384	0	0
rxn03393	0	0
rxn03395	0	0
rxn03397	0	0

rxn03402	0	0
rxn03405	0	0
rxn03406	0	0
rxn03407	0	0
rxn03408	0,031494975	0,031494975
rxn03409	0	0
rxn03419	0	0,895688681
rxn03421	0	0,895688681
rxn03423	0	0
rxn03435	-0,218224076	0
rxn03436	0	0,218224076
rxn03437	0	0,218224076
rxn03445	0	0
rxn03446	0	0
rxn03462	0	0
rxn03465	0	0
rxn03467	0	0
rxn03468	0	0
rxn03483	0	0
rxn03491	0	0
rxn03492	0	0
rxn03512	0	0
rxn03513	0	0
rxn03514	0	0
rxn03535	0	0
rxn03536	0	0
rxn03537	0	0
rxn03538	0	0
rxn03540	0	0
rxn03548	0	1000
rxn03549	0	0
rxn03552	0	0
rxn03553	0	0
rxn03596	0	0
rxn03598	0	0
rxn03599	0	0
rxn03608	0	0
rxn03634	0	0
rxn03638	0,062989949	0,06298995
rxn03641	0,000690955	14,39071476
rxn03642	0,000690955	14,39071476
rxn03663	0	0
rxn03668	0	0
rxn03669	0	0
rxn03670	0	0
rxn03671	0	0
rxn03796	0	0
rxn03799	0	0
rxn03838	0	0
rxn03841	0	0,895688681

rxn03861	0	0
rxn03866	0	0
rxn03891	0	0
rxn03900	0	0
rxn03901	0,031494975	0,031494975
rxn03902	0	0
rxn03903	0	0
rxn03904	0,031494975	0,031494975
rxn03907	0	0
rxn03908	0	0
rxn03909	0	0
rxn03910	0	0
rxn03919	0	0
rxn03933	0	0
rxn03958	0	0
rxn03962	0	0
rxn03974	-0,028334856	0
rxn03975	-0,028334856	0
rxn04045	0	0
rxn04046	0	0
rxn04047	0	0
rxn04048	0	0
rxn04050	0	0
rxn04051	0	0
rxn04052	0	0
rxn04068	0	0
rxn04082	0	0,5
rxn04096	0	0
rxn04113	0	0
rxn04142	0	0
rxn04234	0	0
rxn04308	0	0
rxn04384	0	0
rxn04385	0	0
rxn04413	0	0
rxn04432	0	0
rxn04443	0	0
rxn04674	0	0
rxn04676	-6,599338746	1000
rxn04678	-1000	6,599338746
rxn04704	0	0
rxn04726	0	0
rxn04736	0	0
rxn04750	0	0
rxn04786	0,007702147	0,007702147
rxn04794	0	1000
rxn04822	0	0
rxn04865	0	0
rxn04866	0	0
rxn04934	0	0

rxn04943	0	0
rxn04954	-0,322396073	0
rxn04960	0	0
rxn05004	0	0
rxn05005	-1000	0
rxn05006	-1000	0
rxn05010	0	0
rxn05011	0	0
rxn05012	0	0
rxn05024	0	0
rxn05029	0	0
rxn05030	6,28141E-05	6,28141E-05
rxn05039	0	0,000509365
rxn05040	0	0,00101873
rxn05050	0	0
rxn05054	0	0
rxn05108	0	0
rxn05114	0	0
rxn05115	0	0
rxn05234	0	0
rxn05236	0	0
rxn05247	0	0
rxn05248	0	0
rxn05249	0	0
rxn05250	0	0
rxn05251	0	0
rxn05252	0	0
rxn05269	0	0
rxn05289	0	0
rxn05322	0	0
rxn05323	0	0
rxn05324	0	0
rxn05325	0	0
rxn05326	0	0
rxn05327	0	0
rxn05328	0	0
rxn05329	0	0
rxn05330	0	0
rxn05331	0	0
rxn05332	0	0
rxn05333	0	0
rxn05334	0	0
rxn05335	0	0
rxn05336	0	0
rxn05337	0	0
rxn05338	0	0
rxn05339	0	0
rxn05340	0	0
rxn05341	0	0
rxn05342	0	0

rxn05343	0	0
rxn05344	0	0
rxn05345	0	0
rxn05346	0	0
rxn05347	0	0
rxn05348	0	0
rxn05350	0	0
rxn05351	0	0
rxn05352	0	0
rxn05353	0	0
rxn05354	0	0
rxn05355	0	0
rxn05356	0	0
rxn05357	0	0
rxn05457	-1000	0
rxn05465	0	0
rxn05733	0	0
rxn05736	0	1000
rxn05740	-1000	1000
rxn05759	-0,5	0
rxn05760	-1000	1000
rxn05762	0	0
rxn05778	0	0
rxn05779	0	0
rxn05794	-1000	0
rxn05824	0	0
rxn05833	0	0
rxn05853	0	0
rxn05854	0	0
rxn05871	0	0
rxn05872	0	0
rxn05874	0	0
rxn05899	0	0
rxn05901	0	0
rxn05902	-0,195705802	0
rxn05918	0	0
rxn05927	0	0
rxn05934	-1000	0
rxn05937	-1000	1000
rxn05938	-14,23516143	0
rxn05939	-0,000111923	999,9998881
rxn05940	-1000	1000
rxn05958	0	0
rxn05962	0	0
rxn05994	0	0
rxn05995	0	0
rxn06023	0	0
rxn06033	0	0
rxn06038	0	0
rxn06043	0	0

rxn06044	0	0
rxn06045	0	0
rxn06071	0,000509365	670,0209611
rxn06078	0	0
rxn06081	0	0
rxn06090	0	0
rxn06096	0	0
rxn06108	-1000	0
rxn06109	-7,388135093	0
rxn06139	0	0
rxn06140	0	0
rxn06154	0	0
rxn06155	0	0
rxn06181	0	1000
rxn06182	0	1000
rxn06190	0	0
rxn06195	0	0
rxn06196	0	0
rxn06200	0	0
rxn06201	0	0
rxn06231	0	0
rxn06251	0	0
rxn06280	0	0
rxn06285	0	0
rxn06298	0	0
rxn06299	0	0
rxn06300	0	0
rxn06316	0	0
rxn06328	0	0
rxn06347	0	0
rxn06348	0	0
rxn06373	0	0
rxn06394	0	0
rxn06403	0	0
rxn06432	0	0
rxn06434	0	0
rxn06435	0	0
rxn06437	0	0
rxn06438	0	0
rxn06439	0	0
rxn06440	0	0
rxn06441	0	0
rxn06443	0	0
rxn06444	0	0
rxn06445	0	0
rxn06446	0	0
rxn06447	0	0
rxn06448	0	0
rxn06449	0	0
rxn06476	0	0

rxn06485	0	0
rxn06489	0	0
rxn06500	0	0
rxn06510	0	0
rxn06525	0	1000
rxn06526	-1000	0
rxn06538	0	0
rxn06556	0	0
rxn06565	0	0
rxn06584	0	0
rxn06591	0,00203746	0,00203746
rxn06595	0	0
rxn06624	0	0
rxn06648	0	0
rxn06664	0	0
rxn06672	0	1000
rxn06673	0	1000
rxn06701	0	0
rxn06726	0	0
rxn06729	0	0
rxn06737	0	0
rxn06751	0	0
rxn06752	0	0
rxn06760	0	0
rxn06768	0	0
rxn06799	0	0
rxn06823	0	0
rxn06831	0	0
rxn06850	0	0
rxn06864	0	0
rxn06865	0	0
rxn06882	0	0
rxn06887	0	0
rxn06889	0	1000
rxn06890	0	0
rxn06936	0	0
rxn06937	0,00203746	0,00203746
rxn06947	0	0
rxn06958	-670,0209611	-0,000509365
rxn06979	0	0
rxn07056	0	0
rxn07059	0	0
rxn07099	0	0
rxn07189	0	0
rxn07199	0	0
rxn07267	0	0
rxn07292	0	0
rxn07312	0	0
rxn07332	0	0
rxn07335	0	0

rxn07437	0	0
rxn07441	0	999,968505
rxn07456	0	1000
rxn07465	0,007702147	0,007702147
rxn07466	-0,029267931	999,9707321
rxn07476	0	0
rxn07484	0	0
rxn07485	0	0
rxn07489	0	0
rxn07573	0	0
rxn07577	0	0
rxn07578	0	0
rxn07579	0	0
rxn07581	0	0
rxn07584	0	0
rxn07585	0	0
rxn07586	0	0
rxn07587	0	0
rxn07588	0	0
rxn07623	0	0
rxn07679	0	0
rxn07681	0	0
rxn07683	0	0
rxn07685	0	0
rxn07687	0	0
rxn07689	0	0
rxn07804	0	0
rxn07807	0	0
rxn07832	0	0
rxn07846	0	0
rxn07849	0	0
rxn07880	0	0
rxn07884	0	0
rxn07987	0	0
rxn07989	0	0
rxn07991	0	0
rxn07992	0	0
rxn07993	0	0
rxn07994	0	0
rxn08035	0	0
rxn08038	0	0
rxn08043	0	0,218224076
rxn08067	-1000	1000
rxn08083	0	0
rxn08084	0	0
rxn08085	0	0
rxn08086	0	0
rxn08087	0	0
rxn08088	0	0
rxn08089	0	0

rxn08126	0	0
rxn08127	0	0
rxn08128	0	0
rxn08129	0	0
rxn08171	0	0
rxn08180	0	0
rxn08194	-1000	1000
rxn08206	0	0
rxn08207	0	0
rxn08208	0	0
rxn08209	0	0
rxn08294	0	0
rxn08295	0	0
rxn08296	0	0
rxn08297	0	0
rxn08298	0	0
rxn08299	0	0
rxn08300	0	0
rxn08306	0	0
rxn08307	0	0
rxn08308	0	0
rxn08309	0	0
rxn08310	0	0
rxn08311	0	0
rxn08312	0	0
rxn08352	0	0
rxn08386	0	0
rxn08387	0	0
rxn08390	0	0
rxn08391	0	0
rxn08392	0	0
rxn08393	0	0
rxn08394	0	0
rxn08395	0	0
rxn08396	0	0
rxn08397	0	0
rxn08398	0	0
rxn08399	0	0
rxn08413	0	0
rxn08433	0	0
rxn08434	0	1000
rxn08435	0	0
rxn08436	0	0
rxn08437	0	0
rxn08438	0	0
rxn08444	0	1000
rxn08448	0	0
rxn08449	0	0
rxn08451	0	0
rxn08453	0	0

rxn08454	0	1000
rxn08455	0	0
rxn08456	0	1000
rxn08457	0	0
rxn08519	0,057583371	0,057583371
rxn08546	0	0
rxn08547	0	1000
rxn08548	0	0
rxn08549	0	0
rxn08550	0	0
rxn08551	0	0
rxn08552	0	0
rxn08571	0	1000
rxn08582	0	0,5
rxn08605	0	0
rxn08607	0	0
rxn08615	-1000	1000
rxn08647	0	0
rxn08668	0	0
rxn08669	0	0
rxn08733	-1000	0
rxn08764	0	0,218224076
rxn08767	0	0
rxn08796	0	0
rxn08797	0	1000
rxn08798	0	0
rxn08799	0	1000
rxn08800	0	0
rxn08801	0	1000
rxn08802	0	0
rxn08803	0	0
rxn08804	0	0
rxn08805	0	0
rxn08806	0	0
rxn08807	0	0
rxn08808	0	0
rxn08809	0	0
rxn08810	0	0
rxn08811	0	0
rxn08812	0	0
rxn08813	0	0
rxn08814	0	0
rxn08815	0	0
rxn08816	0	0
rxn08817	0	0
rxn08818	0	0
rxn08819	0	0
rxn08820	0	0
rxn08821	0	0
rxn08822	0	0

rxn08823	0	0
rxn08838	0	0
rxn08839	0	0
rxn08840	0	0
rxn08841	0	0
rxn08842	0	0
rxn08843	0	0
rxn08844	0	0
rxn08845	0	0
rxn08846	0	0
rxn08847	0	0
rxn08848	0	0
rxn08849	0	0
rxn08850	0	0
rxn08851	0	0
rxn08857	0	0
rxn08889	0,000768616	0,000768616
rxn08890	0,006222019	0,006222019
rxn08891	0,000768616	0,000768616
rxn08892	0,013058474	0,013058474
rxn08893	0,0061455	0,0061455
rxn08894	0,00153609	0,00153609
rxn08897	-0,006912974	-0,006912974
rxn08926	0,000690955	0,000690955
rxn08928	0,00153609	0,00153609
rxn08929	0,00153609	0,00153609
rxn08958	0,000768616	0,000768616
rxn09010	0	0
rxn09016	0	999,7611923
rxn09108	0	0
rxn09109	0	0
rxn09110	0	0
rxn09111	0	0
rxn09112	0	0
rxn09113	0	0
rxn09114	0	0
rxn09176	-1000	1000
rxn09177	0	0,000657835
rxn09197	0	0
rxn09198	0	0
rxn09199	0	0
rxn09200	0	0
rxn09201	0	0
rxn09202	0	0
rxn09203	0	0
rxn09205	0	0
rxn09206	0	0
rxn09207	0	0
rxn09208	0	0
rxn09209	0	0

rxn09210	0	0
rxn09211	0	0
rxn09235	0,028334856	0,028334856
rxn09237	0,029248515	0,029248515
rxn09240	0	0,095705802
rxn09244	0	0
rxn09264	0	0
rxn09265	0	0
rxn09340	0	0
rxn09341	0	999,5995158
rxn09348	0	999,5995158
rxn09355	0	0
rxn09395	0	0
rxn09399	0	0
rxn09412	-1000	1000
rxn09445	0	0
rxn09446	0	0
rxn09447	0	0
rxn09454	0	0
rxn09455	0	0
rxn09456	0	0
rxn09473	0	0
rxn09480	0	0
rxn09486	0	0
rxn09502	0	1000
rxn09531	0	0
rxn09557	0,000254683	1000
rxn09616	0,000690955	0,000690955
rxn09631	0,000254683	0,000254683
rxn09633	0,000254683	0,000254683
rxn09888	0	0
rxn09889	0	0
rxn09949	0	0
rxn09952	0	0
rxn09978	0	0
rxn09979	0	0
rxn09988	0	0
rxn09995	0	0
rxn10003	0	0,000657835
rxn10019	0	0
rxn10020	0	0
rxn10021	0	0
rxn10052	-1000	1000
rxn10054	0	999,5995158
rxn10056	0	0,000510507
rxn10058	0	0,000510507
rxn10060	0	0,000510507
rxn10091	-1000	1000
rxn10110	0	0
rxn10111	0	0

rxn10192	0	0
rxn10193	0	0
rxn10194	0	0
rxn10196	0	0
rxn10202	0	1000
rxn10203	0	1000
rxn10204	0	1000
rxn10205	0	0
rxn10206	0	0
rxn10207	0	0
rxn10208	0	0
rxn10209	0	0
rxn10210	0	0
rxn10211	0	0
rxn10212	0	0
rxn10213	0	0
rxn10214	0	0
rxn10215	0	0
rxn10216	0	0
rxn10217	0	0
rxn10218	0	0
rxn10219	0	0
rxn10220	0	0
rxn10221	0	0
rxn10222	0	0
rxn10223	0	0
rxn10224	0	0
rxn10225	0	0
rxn10226	0	0
rxn10227	0	0
rxn10228	0	0
rxn10229	0	0
rxn10230	0	0
rxn10231	0	0
rxn10232	0	0
rxn10233	0	0
rxn10234	0	0
rxn10235	0	0
rxn10236	0	0
rxn10237	0	0
rxn10253	0	0
rxn10254	0	0
rxn10255	0	0
rxn10256	0	0
rxn10257	0	0
rxn10258	0	0
rxn10259	0	0
rxn10260	0	0
rxn10261	0	0
rxn10262	0	0

rxn10263	0	0
rxn10264	0	0
rxn10289	0	0
rxn10290	0	0
rxn10291	0	0
rxn10292	0	0
rxn10293	0	0
rxn10294	0	0
rxn10295	0	0
rxn10296	0	0
rxn10297	0	0
rxn10298	0	0
rxn10299	0	0
rxn10300	0	0
rxn10301	0	0
rxn10302	0	0
rxn10303	0	0
rxn10304	0	0
rxn10305	0	0
rxn10306	0	0
rxn10363	0	0
rxn10404	0	0
rxn10405	0	0
rxn10406	0	0
rxn10407	0	0
rxn10408	0	0
rxn10409	0	0
rxn10410	0	0
rxn10785	6,28141E-05	6,28141E-05
rxn10951	0	0,028334856
rxn11007	0,028334856	0,028334856
rxn11547	0	0
rxn11548	0	0
rxn11550	0	0
rxn11564	0	0
rxn11567	0	0
rxn11571	-833,5631637	0
rxn11587	0	0
rxn11599	0	0
rxn11609	0	0
rxn11642	0	0
rxn11676	0	0
rxn11702	0	0
rxn11703	0	0
rxn11711	0	0
rxn11712	0	0
rxn11713	0	0
rxn11716	0	0
rxn11728	0	0
rxn11732	0	0

rxn11749	0	0
rxn11755	0	0
rxn11757	-999,9971425	0
rxn11759	0	999,9971425
rxn11760	-999,9971425	0
rxn11761	0	0
rxn11765	0	0
rxn11766	0	0
rxn11768	0	0
rxn11772	0	0
rxn11773	0	0
rxn11934	-0,095705802	0
rxn11946	0	0
rxn11951	0	0
rxn11987	0	833,5631637
rxn12013	0	0
rxn12049	0	0
rxn12218	-1000	-0,000254683
rxn12221	0,000254683	1000
rxn12510	0,000657835	0,000657835
rxn12649	-1000	0
rxn12767	0	0
rxn12768	0	0
rxn12769	0	0
rxn12770	0	0
rxn12771	0	0
rxn12778	0	0
rxn12822	-1000	0
rxn13420	0,000690955	1000
rxn13421	0,000690955	1000
rxn13705	0	0
rxn13741	0	0
rxn13906	-0,007702147	-0,007702147
rxn13936	0,015363179	0,01536318
rxn13974	-14,23516143	0
rxn13994	0	0
rxn14029	0	0
rxn14033	0	0
rxn14043	0	0
rxn14048	-1000	0
rxn14050	0	0
rxn14054	-1000	0
rxn14057	-0,195705802	0
rxn14063	0	0
rxn14070	0	0
rxn14089	-1000	0
rxn14093	0	0
rxn14120	-1000	0,894669951
rxn14132	0	0
rxn14178	-1000	1000

rxn14250	0	0
rxn14270	0	0
rxn14279	0	0
rxn14328	0	0
rxn14346	0	0
rxn90002	-13,52446589	1000
rxn90003	0	0
rxn90004	0	0
rxn90005	-0,028845363	-0,028334856
rxn08173	0	500
Biomass_Bacteria	1,142074	1,142074006
t_Cl	0,005153038	0,005153038
t_Sulfate	0,004294198	0,1
t_Cu2+	0,003435359	0,003435359
t_Mg	0,008587254	0,008587254
t_Ca2+	0,005153038	0,005153038
t_NH3	0	0
t_H2O	-23,75482219	8,351937578
t_Biomass	-1,142074006	-1,142074
t_Butyrate	-8,670345255	0
t_D-Lactate	-14,23516143	0
t_Ethanol	0	0
t_Formate	-14,77627019	0
t_H2	0	0,5
t_L-Lactate	-14,23516143	0
t_Nitrite	0	0
t_Phosphate	1,51196664	2,011966651
t_Propionate	-0,218224076	0
t_O2	0	0
t_D-Glucose	0	0,5
t_CO2	-14,77627019	0
t_Acetate	-21,59843195	0
t_Succinate	-7,388135093	0
t_(S,S)-2,3-Butanediol	0	0
t_H2S	-0,592905437	0
Ex_Cl	-0,005153038	-0,005153038
Ex_Sulfate	-0,1	-0,004294198
Ex_Cu2+	-0,003435359	-0,003435359
Ex_Mg	-0,008587254	-0,008587254
Ex_Ca2+	-0,005153038	-0,005153038
Ex_NH3	0	0
Ex_H2O	-8,351937578	23,75482219
Ex_Biomass	1,142074	1,142074006
Ex_Butyrate	0	8,670345255
Ex_D-Lactate	0	14,23516143
Ex_Ethanol	0	0
Ex_Formate	0	14,77627019
Ex_H2	-0,5	0
Ex_L-Lactate	0	14,23516143
Ex_Nitrite	0	0

Ex_Phosphate	-2,011966651	-1,51196664
Ex_Propionate	0	0,218224076
Ex_O2	0	0
Ex_D-Glucose	-0,5	0
Ex_CO2	0	14,77627019
Ex_Acetate	0	21,59843195
Ex_Succinate	0	7,388135093
Ex_(S,S)-2,3-Butanediol	0	0
Ex_H2S	0	0,592905437
t_Fe2	0,007983097	0,007983097
t_fe3	0,007728415	0,007728415
t_Acetaldehyde	0	0
t_Adenosine	0	0,5
t_Allantoin	0	0
t_AMP	0	0,5
t_Amylotriose	0	0
t_BIOT	0	0
t_Choline	0	0
t_Cytidine	0	0
t_Cytosine	0	0
t_DAlanine	0	0
t_Deoxyadenosine	0	0,057583371
t_Deoxycytidine	0	0,057583371
t_Deoxyguanosine	0	0
t_Deoxyinosine	0	0
t_Deoxyuridine	0	0
t_DRibose	0	0,5
t_DSerine	0	0
t_Glycerol	0	0
t_GSH	0	0
t_Guanine	0	0
t_H2S2O3	0	0
t_Heme	0,000254683	0,000254683
t_Homocysteine	0	0
t_HYXN	0	0,5
t_Inosine	0	0,5
t_LACT	0	0,5
t_LAlanine	-1,531365954	0,5
t_LArabinose	0	0,5
t_LArginine	-0,007841488	0,5
t_LAsparagine	-0,515682977	0,5
t_LAspartate	-1,531365954	0,5
t_LCysteine	-0,092905437	0,5
t_LGlutamate	-1,531365954	0,5
t_LGlutamine	-0,515682977	0,5
t_LHistidine	-0,622111325	0,105185016
t_LInositol	0	0
t_LIsoleucine	0,104183414	0,322407492
t_LLeucine	0,499999997	0,5
t_LLysine	-0,634915505	0,380767474

t_LMethionine	-0,047457746	0,170766332
t_LPhenylalanine	-1,825792634	0,205573321
t_LThreonine	0,281775924	0,5
t_LTryptophan	-0,522409101	0,063076747
t_LTyrosine	-1,878328038	0,153037917
t_LValine	-1,560945673	0,470420283
t_Maltose	0	0,5
t_Niacin	0	0,002602787
t_Ornithine	0	0
t_PPi	0	0
t_Pyridoxol	0	0
t_XAN	0	0
t_5Deoxyadenosine	0	0
t_Acetoacetate	-8,670345255	0
t_BET	0	0
t_Calomide	0	0
t_Carnosine	0	0
t_Cbl	0	0
t_Citrate	0	0
t_CysGly	0	0
t_Glycine	-0,16798625	0,5
t_Glycolaldehyde	0	0
t_LProline	-1,786048459	0,245317497
t_Maltohexaose	0	0
t_Methanol	0	0
t_NAcetylDglucosamine	0	0
t_PM	0	0
t_Putrescine	0	0
t_Pyridoxal	0,000254683	0,000254683
t_Riboflavin	0	0,000509365
t_Sorbitol	0	0
t_Spermidine	0	0
t_Sucrose	0	0,5
t_Taurine	0	0
t_Thiamin	0	0
t_Thyminose	0	0,057583371
t_TRHL	0	0
t_Uracil	0	0,365829146
t_Uridine	0	0,365829146
t_Mn2+	0,003435359	0,003435359
t_Fumarate	-7,388135093	0
t_Oxidized glutathione	0	0
t_Adenine	0	0
t_Nicotinamide	0	0
t_4-Hydroxybenzoate	0	0
t_Co2+	0,003435359	0,003435359
t_D-Arabinose	0	0,5
t_D-Glutamate	0	0
t_Chorismate	0	0
t_Folate	-0,894669951	0,00101873

t_N-Acetyl-D-mannosamine	0	0
t_Siroheme	0	0
t_Selenate	0	0
t_Menaquinone 7	0	0
t_2-Demethylmenaquinone 8	0	0
t_Menaquinone 8	0	0
t_Ubiquinone-8	0	0
t_2-Oxobutyrate	0	0
t_3MOP	0	0
t_ABEE	0	0
t_Neu5Ac	0	0
t_Glycerol-3-phosphate	0	0
t_H+	-1000	0,5
t_indol	0	0
t_Nicotinamide ribonucleotide	0	0
t_PAN	0,000657835	0,000657835
t_Pyridoxal phosphate	0	0
t_Zn2+	0,003435359	0,003435359
t_1,2-Diacyl-sn-glycerol dioctadecanoyl	0	0
t_meso-2,6-Diaminopimelate	0	0
t_L-Serine	-0,23185731	0,5
t_D-Fructose	0	0,5
t_D-Mannose	0	0
t_L-Rhamnose	0	0
t_beta D-Galactose	0	0,5
t_L-Fucose	0	0
Ex_Fe2	-0,007983097	-0,007983097
Ex_fe3	-0,007728415	-0,007728415
Ex_Acetaldehyde	0	0
Ex_Adenosine	-0,5	0
Ex_Allantoin	0	0
Ex_AMP	-0,5	0
Ex_Amylotriose	0	0
Ex_BIOT	0	0
Ex_Choline	0	0
Ex_Cytidine	0	0
Ex_Cytosine	0	0
Ex_DAlanine	0	0
Ex_Deoxyadenosine	-0,057583371	0
Ex_Deoxycytidine	-0,057583371	0
Ex_Deoxyguanosine	0	0
Ex_Deoxyinosine	0	0
Ex_Deoxyuridine	0	0
Ex_DRibose	-0,5	0
Ex_DSerine	0	0
Ex_Glycerol	0	0
Ex_GSH	0	0
Ex_Guanine	0	0
Ex_Heme	-0,000254683	-0,000254683
Ex_Homocysteine	0	0

Ex_HYXN	-0,5	0
Ex_Inosine	-0,5	0
Ex_LACT	-0,5	0
Ex_LAlanine	-0,5	1,531365954
Ex_LArabinose	-0,5	0
Ex_LArginine	-0,5	0,007841488
Ex_LAsparagine	-0,5	0,515682977
Ex_LAspartate	-0,5	1,531365954
Ex_LCysteine	-0,5	0,092905437
Ex_LGlutamate	-0,5	1,531365954
Ex_LGlutamine	-0,5	0,515682977
Ex_LHistidine	-0,105185016	0,622111325
Ex_LInositol	0	0
Ex_LIsoleucine	-0,322407492	-0,104183414
Ex_LLeucine	-0,5	-0,499999997
Ex_LLysine	-0,380767474	0,634915505
Ex_LMethionine	-0,170766332	0,047457746
Ex_LPhenylalanine	-0,205573321	1,825792634
Ex_LThreonine	-0,5	-0,281775924
Ex_LTryptophan	-0,063076747	0,522409101
Ex_LTyrosine	-0,153037917	1,878328038
Ex_LValine	-0,470420283	1,560945673
Ex_Maltose	-0,5	0
Ex_Niacin	-0,002602787	0
Ex_Ornithine	0	0
Ex_PPi	0	0
Ex_XAN	0	0
Ex_5Deoxyadenosine	0	0
Ex_Acetoacetate	0	8,670345255
Ex_BET	0	0
Ex_Calomide	0	0
Ex_Carnosine	0	0
Ex_Cbl	0	0
Ex_Citrate	0	0
Ex_CysGly	0	0
Ex_Glycine	-0,5	0,16798625
Ex_Glycolaldehyde	0	0
Ex_LProline	-0,245317497	1,786048459
Ex_Maltohexaose	0	0
Ex_Methanol	0	0
Ex_NAcetylDglucosamine	0	0
Ex_PM	0	0
Ex_Putrescine	0	0
Ex_Pyridoxal	-0,000254683	-0,000254683
Ex_Riboflavin	-0,000509365	0
Ex_Sorbitol	0	0
Ex_Spermidine	0	0
Ex_Sucrose	-0,5	0
Ex_Taurine	0	0
Ex_Thiamin	0	0

Ex_Thyminose	-0,057583371	0
Ex_TRHL	0	0
Ex_Uracil	-0,365829146	0
Ex_Uridine	-0,365829146	0
Ex_Mn2+	-0,003435359	-0,003435359
Ex_Fumarate	0	7,388135093
Ex_Oxidized glutathione	0	0
Ex_Adenine	0	0
Ex_Nicotinamide	0	0
Ex_4-Hydroxybenzoate	0	0
Ex_Co2+	-0,003435359	-0,003435359
Ex_D-Arabinose	-0,5	0
Ex_D-Glutamate	0	0
Ex_Folate	-0,00101873	0,894669951
Ex_N-Acetyl-D-mannosamine	0	0
Ex_Siroheme	0	0
Ex_Selenate	0	0
Ex_Menaquinone 7	0	0
Ex_2-Demethylmenaquinone 8	0	0
Ex_Menaquinone 8	0	0
Ex_Ubiquinone-8	0	0
Ex_ABEE	0	0
Ex_Neu5Ac	0	0
Ex_H+	-0,5	1000
Ex_indol	0	0
Ex_Nicotinamide ribonucleotide	0	0
Ex_PAN	-0,000657835	-0,000657835
Ex_Zn2+	-0,003435359	-0,003435359
Ex_1,2-Diacyl-sn-glycerol dioctadecanoyl	0	0
Ex_L-Serine	-0,5	0,23185731
Ex_D-Fructose	-0,5	0
Ex_D-Mannose	0	0
Ex_L-Rhamnose	0	0
Ex_beta D-Galactose	-0,5	0
Ex_L-Fucose	0	0
t_Arabinan	0	0
t_Starch	0	0,005
t_octanoate	0	0
t_Melibiose	0	0,5
t_Linolenate	0	0
t_Amylose	0	0
t_Linoleate	0	0
Ex_Arabinan	0	0
Ex_Starch	-0,005	0
Ex_Melibiose	-0,5	0
Ex_Linolenate	0	0
Ex_Amylose	0	0
Ex_Linoleate	0	0
t_Raffinose_Melitose	0	0
t_Isovaleric_acid	0	0

t_H2O2	0	0
Ex_Raffinose_Melitose	0	0
Ex_Isovaleric_acid	0	0
Ex_H2O2	0	0
rxn01207_1	0	0
rxn08972	0	0
rxn08973	0	0
rxn06111	0	1000
rxn13726	0	0
rxn13727	0	0
rxn13729	0	0
rxn08974	0	0
rxn10122	0	0
rxn10123	0	0
rxn10124	0	0
rxn12665	0	0
rxn06097	0	0,005
t_Sulfite	0	0,1
Ex_Sulfite	-0,1	0

rxn ID	minFlux	max Flux
rxn00001	0	1000
rxn00003	-2,590644158	0
rxn00004	0	0
rxn00011	-2,590644158	0
rxn00016	0	0
rxn00020	0	0
rxn00022	0	0,505
rxn00028	0	0
rxn00029	0,00101873	0,00101873
rxn00031	0	0
rxn00048	0	0,000509365
rxn00059	0	0
rxn00060	0,000254683	0,000254683
rxn00062	0	1000
rxn00065	0	1000
rxn00067	0	0
rxn00076	0	1000
rxn00077	0	0,000510507
rxn00083	-1000	1000
rxn00085	-1000	0
rxn00097	-1000	1000
rxn00100	0,000657835	0,000657835
rxn00102	-1000	0,694385566
rxn00103	0	1000
rxn00104	-1000	0
rxn00105	-999,9973972	1000
rxn00106	-1000	0
rxn00109	0	0
rxn00114	-1000	23,16301225
rxn00119	0,368989262	1000
rxn00121	-0,000254683	0
rxn00122	0	0,000254683
rxn00124	0,000254683	0,000254683
rxn00126	0,000764048	1000
rxn00131	-1000	999,9966332
rxn00132	0	1000
rxn00133	0	0
rxn00137	0	0
rxn00139	-999,9971425	0
rxn00140	0	1000
rxn00141	-999,9994906	0
rxn00142	0	0
rxn00143	0,000509365	1000
rxn00144	0	0
rxn00148	-1000	0
rxn00151	-1000	0
rxn00154	0	22,78027698
rxn00157	-22,78027698	0
rxn00159	-1000	1000

rxn00161	-1000	1000
rxn00162	0	1000
rxn00165	0	8,183510942
rxn00171	0	1,303068753
rxn00173	-22,78027698	1000
rxn00175	0	1000
rxn00176	0	0
rxn00178	-23,28864496	0
rxn00179	0	0
rxn00184	-1000	0
rxn00187	0	1000
rxn00189	0	1000
rxn00190	0,002602787	1000
rxn00191	-1000	1000
rxn00192	0	1000
rxn00193	0,031494975	0,031494975
rxn00196	0	0
rxn00200	0	0
rxn00206	0,000254683	174,3070303
rxn00211	0	0
rxn00214	-1,5	0
rxn00216	0	1000
rxn00221	0	1000
rxn00222	0	0
rxn00223	-1	1000
rxn00224	0,000254683	1000
rxn00225	-1000	0
rxn00226	0	0
rxn00239	0,238807673	1000
rxn00242	0	821,8829791
rxn00245	0	0
rxn00247	0	1000
rxn00250	-0,69442097	999,9999646
rxn00254	0	0
rxn00255	0	0
rxn00256	-22,78027698	0
rxn00259	-22,78027698	0
rxn00260	-1000	1000
rxn00262	0	174,3067757
rxn00272	-1000	1000
rxn00275	-1000	1000
rxn00278	-1000	0
rxn00283	0,027731841	0,027731841
rxn00290	-8,028564616	-0,000690955
rxn00293	-999,9370101	1000
rxn00295	-1000	999,9370101
rxn00299	0	0
rxn00300	0	0,000509365
rxn00301	0	999,7611923
rxn00302	0	0

rxn00303	0	821,8829791
rxn00304	-1000	0
rxn00307	0	0
rxn00313	0	1,295322079
rxn00322	0	0
rxn00324	-174,3067757	0
rxn00328	0	0
rxn00333	0,000254683	174,3070303
rxn00337	0,039197122	1,334519201
rxn00338	0	0
rxn00340	0	1000
rxn00342	0	1000
rxn00346	0	0
rxn00347	0	1000
rxn00350	-0,000254683	-0,000254683
rxn00358	0	0
rxn00360	0	1000
rxn00361	0	1000
rxn00362	-1000	1000
rxn00363	0	1000
rxn00364	-999,5995158	0,000657835
rxn00365	0	1000
rxn00368	0	999,5995158
rxn00369	0	1000
rxn00371	0	1000
rxn00379	0	0
rxn00383	0	0
rxn00391	0	999,9997453
rxn00392	0,000254683	1000
rxn00405	0	23,33478018
rxn00410	-999,8225103	999,7770055
rxn00411	-1000	0
rxn00412	0	1000
rxn00414	0	1000
rxn00416	0	1000
rxn00422	-1000	1000
rxn00423	0	8,183510942
rxn00424	-1000	1000
rxn00426	0	0
rxn00433	0	0
rxn00436	0	999,9997453
rxn00437	0	0
rxn00440	0,000254683	1000
rxn00452	0	999,999236
rxn00453	0	999,9997453
rxn00456	0	999,9997453
rxn00459	-1,220478164	22,15039346
rxn00460	-1000	-0,400484237
rxn00461	0,031494975	0,031494975
rxn00462	0	0

rxn00469	0	1000
rxn00470	0	23,16301225
rxn00474	0	0
rxn00490	0	0
rxn00493	-2,590644158	0
rxn00499	-15,18685132	0
rxn00500	-15,18685132	0
rxn00505	0	22,78027698
rxn00506	0	1,303068753
rxn00510	0	0
rxn00512	-174,3067757	0
rxn00514	0	0
rxn00517	-1000	0
rxn00521	0	0
rxn00527	-2,590644158	0
rxn00533	-999,9999646	3,54043E-05
rxn00536	-1000	1000
rxn00541	-1,303068753	0
rxn00543	-1000	1000
rxn00545	0	1000
rxn00547	0	1000
rxn00548	0	22,78027698
rxn00549	0	1000
rxn00551	0	1000
rxn00552	-0,06298995	999,9370101
rxn00554	0	1000
rxn00555	0	1000
rxn00556	0	999,5995158
rxn00557	0	1000
rxn00558	-1000	1000
rxn00559	0	0
rxn00565	0	0
rxn00566	0	1000
rxn00575	0	1000
rxn00577	-1000	0,5
rxn00585	0	0
rxn00586	0	0
rxn00602	0	0
rxn00606	0	0
rxn00607	0	0
rxn00608	0	0
rxn00611	-12,20502086	0
rxn00615	0	12,20502086
rxn00616	0	12,20502086
rxn00622	0	0
rxn00641	0	0
rxn00647	0	0
rxn00649	0	8,183510942
rxn00650	-0,000254683	-0,000254683
rxn00653	0	0

rxn00654	0	0
rxn00659	0	0
rxn00661	0	0
rxn00670	0	1000
rxn00673	0	0
rxn00674	0	1000
rxn00675	0	0
rxn00684	0	0
rxn00685	0	999,9989813
rxn00686	0	0
rxn00687	0	999,9989813
rxn00689	0	0
rxn00690	0	16,05574732
rxn00692	-0,260507082	7,92300386
rxn00693	0	0,397709
rxn00695	-1000	1000
rxn00701	0	1000
rxn00702	0	0
rxn00704	-1000	2
rxn00707	0	1000
rxn00708	0	1000
rxn00709	0	1000
rxn00710	0	0
rxn00711	-999,9971425	0
rxn00712	0	999,5995158
rxn00713	0	1000
rxn00714	0	0
rxn00715	0	1000
rxn00726	0	1,295322079
rxn00727	0	1,295322079
rxn00729	0	0
rxn00735	0	0
rxn00737	0	1,52154751
rxn00740	0	1000
rxn00741	0	0
rxn00742	-1000	0,000254683
rxn00743	0	12,20502086
rxn00747	-6,503239303	8,683612015
rxn00748	0	0
rxn00756	0	0
rxn00758	0	0
rxn00762	-12,20502086	0
rxn00763	0	0
rxn00765	0	0
rxn00770	0,002857469	1000
rxn00772	0	1000
rxn00775	0	0
rxn00777	-2,165599981	7,629909594
rxn00778	-1000	1000
rxn00781	-1,220478164	13,96688252

rxn00784	0	1,303068753
rxn00785	-15,7048386	7,075438374
rxn00786	-1000	6,503239303
rxn00789	0	0
rxn00790	-0,000254683	-0,000254683
rxn00791	-1,295322079	0
rxn00792	0	0
rxn00796	0	0
rxn00797	-1000	1000
rxn00799	-1000	8,297045275
rxn00800	-0,268565555	510,9376168
rxn00801	0	0
rxn00802	0	23,16301225
rxn00806	0	0
rxn00808	0	1000
rxn00809	-500,9953161	0
rxn00811	-500,9953161	0
rxn00816	0	0,5
rxn00817	0	0,5
rxn00818	0	0
rxn00819	0	0
rxn00827	0	0
rxn00829	0,000690955	0,000690955
rxn00830	6,28141E-05	6,28141E-05
rxn00831	0	999,9971425
rxn00832	0	0
rxn00834	-999,7290863	1000
rxn00836	-999,9971425	0
rxn00838	-0,268565555	510,9376168
rxn00851	0	1000
rxn00853	0	23,33478018
rxn00856	0,008138419	23,29678338
rxn00858	0	0
rxn00864	0	0
rxn00869	0	0
rxn00871	0	12,08769731
rxn00872	-12,08769731	0
rxn00874	0	0
rxn00879	0	0
rxn00881	0	0
rxn00882	0	0
rxn00883	0	0
rxn00889	0	0
rxn00890	0	0
rxn00897	0	0
rxn00898	0	2,590644158
rxn00902	0	0
rxn00903	-1000	1000
rxn00904	-1000	1000
rxn00907	-16,05523796	0,000509365

rxn00908	-8,467955317	0,033850505
rxn00909	-12,09539946	0,397963683
rxn00910	-12,20502086	0
rxn00913	0	1000
rxn00915	-999,9971425	0
rxn00916	-999,7319438	1000
rxn00917	0	1000
rxn00918	0	0
rxn00926	0	511,205673
rxn00927	-1000	1000
rxn00929	-1000	1000
rxn00931	-1000	1000
rxn00938	0	999,9971425
rxn00942	0	1000
rxn00946	0	0
rxn00947	0	0
rxn00950	-1000	0,397199635
rxn00952	0	999,9997453
rxn00955	0,000509365	1000
rxn00972	-22,78027698	0
rxn00973	-1000	1000
rxn00974	-1000	1000
rxn00977	0	0
rxn00979	0,000254683	0,000254683
rxn00980	0	0
rxn00985	-1000	0
rxn00986	0	0
rxn00991	-0,000690955	-0,000690955
rxn01000	0	2,590644158
rxn01008	0	0
rxn01011	-8,183510942	0
rxn01013	-8,183510942	0
rxn01016	0	0
rxn01018	0	0
rxn01019	0	23,16301225
rxn01021	0	0
rxn01029	0	23,33478018
rxn01037	0	0
rxn01042	0	0
rxn01044	0	0
rxn01052	0	0
rxn01069	0	0
rxn01073	0	0
rxn01080	0	0
rxn01100	-1000	0
rxn01101	0	0
rxn01106	-13,96688252	1,220478164
rxn01108	-1000	1000
rxn01109	-1000	1000
rxn01114	0	0,5

rxn01116	-1,999952044	8,129909594
rxn01119	0	0
rxn01122	0	0
rxn01123	0	0
rxn01124	0	0
rxn01133	0	0
rxn01134	0	0
rxn01137	0	511,205673
rxn01138	-1000	1000
rxn01139	0	0
rxn01152	0	0,5
rxn01155	0	0
rxn01169	0	1000
rxn01171	0	1000
rxn01187	0	22,78027698
rxn01189	0	0
rxn01196	0	0
rxn01199	0	0
rxn01200	0	1000
rxn01201	-23,28933591	-0,000690955
rxn01202	0	0
rxn01203	0	0
rxn01204	0,000690955	23,28933591
rxn01210	0	0
rxn01211	-16,05549264	0,000509365
rxn01213	6,28141E-05	6,28141E-05
rxn01225	0	999,9971425
rxn01226	-999,9707515	1000
rxn01228	0	0
rxn01236	-12,08769731	0
rxn01237	0	0
rxn01241	0	0
rxn01249	0	0
rxn01255	0,000254683	2,59089884
rxn01256	0	2,590644158
rxn01259	0	0
rxn01261	0	0
rxn01265	-999,9997453	-0,002602787
rxn01268	0	2,590644158
rxn01274	0	0
rxn01276	0	0
rxn01277	0	0
rxn01278	0	0
rxn01280	0	0
rxn01281	0	0
rxn01286	0	0
rxn01290	0	0
rxn01291	0	0
rxn01292	0	0,5
rxn01297	-999,7319438	999,9971425

rxn01299	-1000	1000
rxn01300	0	0
rxn01303	0	0
rxn01304	0	0
rxn01305	0	0
rxn01308	0	0
rxn01310	0	0
rxn01329	0	0
rxn01332	0,000254683	2,59089884
rxn01333	-1000	1,259677184
rxn01334	0	1000
rxn01343	0	1000
rxn01344	0	0
rxn01346	0	1000
rxn01347	0	999,5995158
rxn01348	0	1000
rxn01351	0	1000
rxn01352	-1000	-0,029248515
rxn01354	-1000	0
rxn01355	0	0
rxn01358	-1000	1000
rxn01361	0	0
rxn01362	0	0
rxn01365	0	0
rxn01366	-1000	1000
rxn01367	0	0
rxn01368	0	821,8736792
rxn01370	0	1000
rxn01374	0	0
rxn01388	-1000	1000
rxn01390	0	0
rxn01396	0	0
rxn01406	0	0
rxn01416	0	0
rxn01423	0	0
rxn01426	0	0
rxn01434	0	23,16301225
rxn01437	0	0
rxn01445	0	999,9707515
rxn01446	-0,029248515	-0,029248515
rxn01457	0	0
rxn01459	0	23,28908123
rxn01463	0	0
rxn01465	0	0
rxn01466	6,28141E-05	6,28141E-05
rxn01476	0	0
rxn01478	0	0
rxn01480	0	0
rxn01484	0	0
rxn01485	-0,06298995	-0,062989949

rxn01486	0	0
rxn01492	0	0
rxn01500	-0,000690955	-0,000690955
rxn01503	0	0
rxn01509	-999,9707515	1000
rxn01510	0	1000
rxn01513	0,028334856	0,028334856
rxn01517	0	0
rxn01518	0,028334856	1000
rxn01519	0	0
rxn01521	0	999,9716651
rxn01522	0	0
rxn01530	0	0
rxn01539	-1000	-0,000254683
rxn01541	-1000	1000
rxn01544	-999,9971425	0
rxn01545	-1000	1000
rxn01548	-999,9707515	1000
rxn01549	0	0
rxn01562	0	0
rxn01563	0	0
rxn01575	-1,521802193	0
rxn01594	0	0
rxn01601	0	0
rxn01602	0	0
rxn01603	0	0
rxn01605	0	0
rxn01610	0	0
rxn01615	0	0
rxn01619	0	0
rxn01620	0	0
rxn01621	0	0
rxn01626	0	0
rxn01629	-0,00203746	-0,00203746
rxn01636	-1000	23,16301225
rxn01637	-23,16301225	0
rxn01641	0	0
rxn01642	0	0
rxn01643	-1,334519201	-0,039197122
rxn01644	0,031494975	1,326817054
rxn01646	-1000	999,9971425
rxn01647	0	999,9971425
rxn01649	-1000	1000
rxn01653	0	0
rxn01654	0	0
rxn01667	0	0
rxn01669	0	999,9973972
rxn01670	0	999,9971425
rxn01675	0	0
rxn01679	0	0

rxn01682	0	0
rxn01683	-1000	1000
rxn01684	-1000	1000
rxn01686	0	0
rxn01704	0	0
rxn01706	0	0
rxn01710	0	0
rxn01735	0	0
rxn01737	0	0
rxn01739	0,000254683	2,59089884
rxn01740	-2,59089884	-0,000254683
rxn01741	0	0
rxn01750	0	0
rxn01757	0	0
rxn01761	0	0
rxn01763	0	0,5
rxn01775	0	0
rxn01790	0	0
rxn01799	-0,028334856	0,33749429
rxn01800	0	0,365829146
rxn01807	0	0
rxn01816	0	1000
rxn01831	0	0
rxn01834	0	0
rxn01835	0	0
rxn01851	0	23,28908123
rxn01857	0	0
rxn01858	0	511,205673
rxn01859	-511,205673	0,494822979
rxn01860	0	0
rxn01870	0	0
rxn01871	0	0
rxn01879	0	0
rxn01892	0	0
rxn01896	0	0
rxn01906	0	0
rxn01911	0	0
rxn01912	0	0
rxn01917	0	23,16301225
rxn01937	0	0
rxn01961	0	999,9971425
rxn01962	0	0
rxn01964	0	1,295322079
rxn01967	0	0
rxn01972	0,031494975	1000
rxn01973	0	0
rxn01974	0,031494975	1,326817054
rxn01977	-1000	1000
rxn01985	0	511,205673
rxn01986	-0,057583371	0,803068753

rxn01987	-0,5	0
rxn01990	0	0
rxn01991	0	0
rxn01997	0	0
rxn02000	0	0
rxn02003	0	0
rxn02007	0	0
rxn02008	0,031494975	0,031494975
rxn02011	0,031494975	0,031494975
rxn02015	0	0
rxn02020	0	0
rxn02023	0	0
rxn02033	0	0
rxn02056	0	999,9997453
rxn02061	0	0
rxn02093	0	0
rxn02102	-1000	0
rxn02103	0	1000
rxn02106	0	0
rxn02112	0	0
rxn02122	0	0
rxn02128	0	0
rxn02138	0	0
rxn02139	0	0
rxn02144	0	0
rxn02154	0	999,9973972
rxn02155	0,002602787	1000
rxn02160	0	0
rxn02161	0	0
rxn02166	0	0
rxn02167	0	0
rxn02171	0,000690955	23,28933591
rxn02172	0	0
rxn02173	0	0
rxn02175	0,000657835	0,000657835
rxn02181	0	0
rxn02185	-2,590644158	1,521802193
rxn02186	0	2,590644158
rxn02187	0	0
rxn02190	0	0
rxn02195	0	0
rxn02199	0	0
rxn02209	0	0
rxn02212	0,000254683	2,59089884
rxn02213	0,000254683	2,59089884
rxn02222	0	0
rxn02228	0	0
rxn02235	0	0
rxn02263	0	0
rxn02264	0,000254683	0,000254683

rxn02283	0	0
rxn02284	-0,031494975	0
rxn02285	-0,031494975	0
rxn02286	0,031494975	0,031494975
rxn02287	-999,9997453	1000
rxn02288	0	0
rxn02302	-1000	-0,000254683
rxn02304	0	0
rxn02305	0,000254683	0,000254683
rxn02312	0	0
rxn02314	0	1000
rxn02315	0	1000
rxn02316	0	999,5995158
rxn02317	-1000	0
rxn02318	0	0
rxn02320	0	0
rxn02322	0,000690955	0,000690955
rxn02339	0	0
rxn02341	0,000657835	0,000657835
rxn02346	0	0
rxn02350	0	0
rxn02351	0	0
rxn02352	0	0
rxn02356	-1000	1000
rxn02358	-1000	1000
rxn02369	0	0
rxn02373	-1000	1000
rxn02380	-1000	1000
rxn02400	0	999,9971425
rxn02409	0	0
rxn02425	0	0
rxn02432	0	0
rxn02433	0	0
rxn02449	0	821,8829791
rxn02454	0	0
rxn02465	-23,16301225	0
rxn02466	0	0
rxn02473	0	0
rxn02474	-0,000509365	0
rxn02475	0	0,000509365
rxn02476	0,000254683	2,59089884
rxn02483	0	0
rxn02484	0,000254683	0,000254683
rxn02495	0	0
rxn02503	0	0
rxn02504	0	0
rxn02507	0	1,295322079
rxn02508	0	1,295322079
rxn02518	0	0
rxn02521	0	0

rxn02522	0	0
rxn02545	0	0
rxn02547	0	0
rxn02569	0	0
rxn02571	0	0
rxn02581	0	0
rxn02596	0	0
rxn02597	0	0
rxn02632	0	0
rxn02679	0	0
rxn02720	0	0
rxn02729	0	0
rxn02749	0	0
rxn02751	0	0
rxn02760	0	0
rxn02762	0	0
rxn02774	-999,9997453	0
rxn02775	0	0
rxn02776	0	0
rxn02789	0	0
rxn02795	0	0
rxn02796	0	0
rxn02803	0	0
rxn02811	0	0
rxn02822	0	0
rxn02831	0	0
rxn02834	0	0
rxn02835	0	0
rxn02845	0	0
rxn02853	0	0
rxn02866	0	0
rxn02875	0	0
rxn02888	0	0
rxn02889	0	0
rxn02895	0,000254683	0,000254683
rxn02897	0	0
rxn02900	0	0
rxn02911	0	0
rxn02914	0	0
rxn02922	0	0
rxn02928	-1000	999,968505
rxn02929	-1000	999,968505
rxn02931	0	0
rxn02934	0	0
rxn02936	0	0
rxn02937	0,000254683	0,000254683
rxn02943	0	0
rxn02949	0	0
rxn02986	0	0
rxn02988	0	0

rxn02990	0	0
rxn03003	0	0
rxn03004	0	0,000254683
rxn03005	-0,000254683	0
rxn03008	0	0
rxn03030	0,031494975	1000
rxn03034	0	0
rxn03039	0	0
rxn03043	0	0
rxn03047	0	0
rxn03052	0	0
rxn03060	0	0
rxn03062	0	0
rxn03068	0	0
rxn03075	0	0
rxn03080	0	0,00101873
rxn03084	0,000254683	0,000254683
rxn03086	-1000	-0,031494975
rxn03087	0	0
rxn03089	0	0
rxn03092	0	0
rxn03094	0	0
rxn03095	0	0
rxn03106	0	0
rxn03108	0,000254683	0,000254683
rxn03123	0	0
rxn03129	0	0
rxn03135	0	0
rxn03136	0	0
rxn03137	0	0
rxn03140	0	0
rxn03141	0	0
rxn03147	0	0
rxn03148	0	0
rxn03150	0	0
rxn03158	0	0
rxn03164	0,031494975	0,031494975
rxn03167	0	0
rxn03174	0	0
rxn03175	0	0
rxn03188	0	0
rxn03194	0	1,521802193
rxn03240	0	0
rxn03241	0	0
rxn03245	0	0
rxn03247	0	0
rxn03250	0	0
rxn03251	0	0
rxn03253	0	0
rxn03263	0	0

rxn03264	0	0
rxn03269	0	0
rxn03273	0	0
rxn03282	0	0
rxn03286	0	0
rxn03287	0	0
rxn03289	0	0
rxn03290	0	0
rxn03292	0	0
rxn03293	0	0
rxn03295	0	0
rxn03296	0	0
rxn03301	0	0
rxn03304	0	0
rxn03313	0	0
rxn03316	0	0
rxn03333	0	0
rxn03354	0	0
rxn03362	0	0
rxn03372	0	0
rxn03373	0	0
rxn03374	0	0
rxn03378	0	0
rxn03379	0	0
rxn03382	0	0
rxn03383	0	0
rxn03384	0	0
rxn03395	0	0
rxn03397	0	0
rxn03402	0	0
rxn03405	0	0
rxn03406	0	0
rxn03407	0	0
rxn03408	0,031494975	0,031494975
rxn03409	0	0
rxn03419	0	0
rxn03421	0	0
rxn03423	0	0
rxn03435	-1,521802193	0
rxn03436	0	1,521802193
rxn03437	0	1,521802193
rxn03445	0	0
rxn03446	0	0
rxn03462	0	0
rxn03468	0	0
rxn03481	0	0
rxn03482	0	0
rxn03483	0	0
rxn03491	0	0
rxn03492	0	0

rxn03504	0	0
rxn03514	0	0
rxn03535	0	0
rxn03536	0	0
rxn03537	0	0
rxn03538	0	0
rxn03540	0	0
rxn03546	0	0
rxn03548	0	1000
rxn03549	0	0
rxn03552	0	0
rxn03553	0	0
rxn03558	0	0
rxn03563	0	0
rxn03596	0	0
rxn03598	0	0
rxn03608	0	0
rxn03638	0,062989949	0,06298995
rxn03641	0,000690955	23,28933591
rxn03642	0,000690955	23,28933591
rxn03643	0	0
rxn03644	0	0
rxn03663	0	0
rxn03666	0	0
rxn03668	0	0
rxn03669	0	0
rxn03670	0	0
rxn03671	0	0
rxn03676	0	0
rxn03838	0	0
rxn03852	0	0
rxn03874	0	0
rxn03884	0	0
rxn03885	0	0
rxn03887	0	0
rxn03891	0	0
rxn03901	0,031494975	0,031494975
rxn03902	0	0
rxn03903	0	0
rxn03904	0,031494975	0,031494975
rxn03907	0	0
rxn03908	0	0
rxn03909	0	0
rxn03910	0	0
rxn03917	0	0
rxn03919	0	0
rxn03933	0	0
rxn03951	0	1000
rxn03958	0	0
rxn03964	0	0

rxn03974	-0,028334856	0
rxn03975	-0,028334856	0
rxn03978	0	0
rxn03990	0	0
rxn03991	0	0
rxn04045	0	0
rxn04046	0	0
rxn04048	0	0
rxn04050	0	0
rxn04068	0	0
rxn04082	0	0,5
rxn04096	0	0
rxn04113	0	0
rxn04142	0	0
rxn04234	0	0
rxn04274	0	0
rxn04275	0	0
rxn04308	0	0
rxn04384	0	0
rxn04385	0	0
rxn04413	0	0
rxn04432	0	0
rxn04443	0	0
rxn04476	0	0
rxn04674	0	0
rxn04676	-7,075183692	1000
rxn04678	-1000	7,075183692
rxn04681	0	0
rxn04682	0	0
rxn04704	0	0
rxn04726	0	0
rxn04736	0	0
rxn04751	0	0
rxn04752	0	0
rxn04786	0,007702147	0,007702147
rxn04794	0	1000
rxn04822	0	0
rxn04908	0	0
rxn04928	0	0
rxn04934	0	0
rxn04952	-1000	0
rxn04953	-1000	0
rxn04954	-12,20502086	0
rxn04960	0	0
rxn05004	0	0
rxn05005	-1000	0
rxn05006	-1000	0
rxn05012	0	0
rxn05029	0	0
rxn05030	6,28141E-05	6,28141E-05

rxn05039	0	0,000509365
rxn05040	0	0,00101873
rxn05044	0	0
rxn05050	0	0
rxn05054	0	0
rxn05115	0	0
rxn05116	0	1000
rxn05117	0	0
rxn05119	0	0
rxn05122	0	0
rxn05124	0	0
rxn05233	0	0
rxn05234	0	0
rxn05236	0	0
rxn05239	0	0
rxn05247	0	0
rxn05248	0	0
rxn05249	0	0
rxn05250	0	0
rxn05251	0	0
rxn05252	0	0
rxn05256	0	0
rxn05269	0	0
rxn05289	0	0
rxn05322	0	0
rxn05323	0	0
rxn05324	0	0
rxn05325	0	0
rxn05326	0	0
rxn05327	0	0
rxn05328	0	0
rxn05329	0	0
rxn05330	0	0
rxn05331	0	0
rxn05332	0	0
rxn05333	0	0
rxn05334	0	0
rxn05335	0	0
rxn05336	0	0
rxn05337	0	0
rxn05338	0	0
rxn05339	0	0
rxn05340	0	0
rxn05341	0	0
rxn05342	0	0
rxn05343	0	0
rxn05344	0	0
rxn05345	0	0
rxn05346	0	0
rxn05347	0	0

rxn05348	0	0
rxn05350	0	0
rxn05457	0	0
rxn05465	0	0
rxn05733	0	0
rxn05736	0	0
rxn05740	-1000	1000
rxn05759	-0,5	0
rxn05760	-1000	1000
rxn05762	0	0
rxn05778	0	0
rxn05779	0	0
rxn05824	0	0
rxn05853	0	0
rxn05856	0	0
rxn05871	0	0
rxn05872	0	0
rxn05873	0	0
rxn05874	0	0
rxn05878	0	0
rxn05893	-0,1	0
rxn05899	0	0
rxn05901	0	0
rxn05918	0	0
rxn05927	0	0
rxn05934	0	0
rxn05937	-1000	1000
rxn05938	-22,78027698	0
rxn05939	0,000579032	1000
rxn05940	-1000	1,521802193
rxn05957	0	1000
rxn05958	0	0
rxn05979	0	0
rxn05990	0	0
rxn05994	0	0
rxn06023	0	0
rxn06043	0	0
rxn06045	0	0
rxn06071	0,000509365	348,6140607
rxn06075	0	0
rxn06078	0	0
rxn06096	0	0
rxn06108	-1000	-0,000690955
rxn06109	-8,028564616	-0,000690955
rxn06139	0	0
rxn06140	0	0
rxn06155	0	0
rxn06181	0	1000
rxn06182	0	1000
rxn06190	0	0

rxn06195	0	0
rxn06196	0	0
rxn06197	0	0
rxn06200	0	0
rxn06201	0	0
rxn06217	0	0
rxn06218	0	0
rxn06219	0	0
rxn06231	0	0
rxn06244	0	0
rxn06252	-1000	0
rxn06253	0	1000
rxn06280	0	0
rxn06285	0	0
rxn06293	0	0
rxn06298	0	0
rxn06299	0	0
rxn06300	0	0
rxn06316	0	0
rxn06328	0	0
rxn06347	0	0
rxn06348	0	0
rxn06400	0	0
rxn06403	0	0
rxn06432	0	0
rxn06434	0	0
rxn06435	0	0
rxn06437	0	0
rxn06438	0	0
rxn06439	0	0
rxn06440	0	0
rxn06441	0	0
rxn06443	0	0
rxn06444	0	0
rxn06445	0	0
rxn06446	0	0
rxn06447	0	0
rxn06448	0	0
rxn06449	0	0
rxn06485	0	0
rxn06489	0	0
rxn06493	0	0
rxn06538	0	0
rxn06556	0	0
rxn06565	0	0
rxn06584	0	0
rxn06591	0,00203746	0,00203746
rxn06592	0	0
rxn06595	0	0
rxn06624	0	0

rxn06641	0	0
rxn06648	0	0
rxn06664	0	0
rxn06671	0	0
rxn06672	0	1000
rxn06673	0	1000
rxn06678	0	0
rxn06691	0	0
rxn06694	0	0
rxn06701	0	0
rxn06726	0	0
rxn06737	0	0
rxn06751	0	0
rxn06768	0	0
rxn06799	0	0
rxn06820	0	0
rxn06823	0	0
rxn06831	0	0
rxn06836	0	0
rxn06837	0	0
rxn06838	0	0
rxn06850	0	0
rxn06860	0	0
rxn06864	0	0
rxn06882	0	0
rxn06883	0	0
rxn06887	0	0
rxn06889	0	1000
rxn06890	0	0
rxn06934	0	0
rxn06936	0	0
rxn06937	0,00203746	0,00203746
rxn06947	0	0
rxn06958	-348,6140607	-0,000509365
rxn06968	0	0
rxn06979	0	0
rxn07056	0	0
rxn07059	0	0
rxn07099	0	0
rxn07189	0	0
rxn07193	0	0
rxn07199	0	0
rxn07251	0	0
rxn07267	0	0
rxn07292	0	0
rxn07332	0	0
rxn07335	0	0
rxn07437	0	0
rxn07438	0	0
rxn07441	0	999,968505

rxn07450	0	0
rxn07456	0	1000
rxn07465	0,007702147	0,007702147
rxn07466	-0,029267931	999,9707321
rxn07484	0	0
rxn07485	0	0
rxn07489	0	0
rxn07492	0	0
rxn07573	0	0
rxn07577	0	0
rxn07578	0	0
rxn07579	0	0
rxn07586	0	0
rxn07587	0	0
rxn07603	0	0
rxn07630	0	0
rxn07679	0	0
rxn07680	0	0
rxn07683	0	0
rxn07684	0	0
rxn07687	0	0
rxn07688	0	0
rxn07804	0	0
rxn07807	0	0
rxn07832	0	0
rxn07846	0	0
rxn07849	0	0
rxn07882	0	0
rxn07987	0	0
rxn07989	0	0
rxn07991	0	0
rxn07992	0	0
rxn07993	0	0
rxn07994	0	0
rxn08025	0	0
rxn08035	0	0
rxn08038	0	0
rxn08040	0	0
rxn08043	0	1,521802193
rxn08044	0	0
rxn08067	-1000	1000
rxn08083	0	0
rxn08084	0	0
rxn08085	0	0
rxn08086	0	0
rxn08087	0	0
rxn08088	0	0
rxn08089	0	0
rxn08094	0	999,999421
rxn08126	0	0

rxn08127	0	0
rxn08128	0	0
rxn08129	0	0
rxn08133	0	0
rxn08171	0	0
rxn08180	0	0
rxn08194	-1000	1000
rxn08294	0	0
rxn08295	0	0
rxn08296	0	0
rxn08297	0	0
rxn08298	0	0
rxn08299	0	0
rxn08300	0	0
rxn08306	0	0
rxn08307	0	0
rxn08308	0	0
rxn08309	0	0
rxn08310	0	0
rxn08311	0	0
rxn08312	0	0
rxn08352	0	0
rxn08386	0	0
rxn08390	0	0
rxn08392	0	0
rxn08394	0	0
rxn08396	0	0
rxn08398	0	0
rxn08413	0	0
rxn08433	0	0
rxn08438	0	0
rxn08448	0	0
rxn08449	0	0
rxn08451	0	0
rxn08453	0	0
rxn08454	0	0
rxn08455	0	0
rxn08456	0	0
rxn08457	0	0
rxn08519	0,057583371	0,057583371
rxn08546	0	0
rxn08547	0	0
rxn08548	0	0
rxn08549	0	0
rxn08550	0	0
rxn08551	0	0
rxn08552	0	0
rxn08571	0	1000
rxn08582	0	0,5
rxn08605	0	0

rxn08607	0	0
rxn08615	-1000	1000
rxn08647	0	8,183510942
rxn08668	0	0
rxn08669	0	0
rxn08700	0	0
rxn08764	0	1,521802193
rxn08857	0	0
rxn08889	0,000768616	0,000768616
rxn08890	0,006222019	0,006222019
rxn08891	0,000768616	0,000768616
rxn08892	0,013058474	0,013058474
rxn08893	0,0061455	0,0061455
rxn08894	0,00153609	0,00153609
rxn08897	-0,006912974	-0,006912974
rxn08926	0,000690955	0,000690955
rxn08928	0,00153609	0,00153609
rxn08929	0,00153609	0,00153609
rxn08958	0,000768616	0,000768616
rxn09010	0	0
rxn09016	0	999,7611923
rxn09069	0	0
rxn09108	0	0
rxn09109	0	0
rxn09110	0	0
rxn09111	0	0
rxn09112	0	0
rxn09113	0	0
rxn09114	0	0
rxn09176	-1000	1000
rxn09177	0	0,000657835
rxn09179	0	0
rxn09180	0	0
rxn09235	0,028334856	0,028334856
rxn09237	0,029248515	0,029248515
rxn09240	0	0
rxn09340	0	0
rxn09341	0	999,5995158
rxn09348	0	999,6310107
rxn09355	0	0
rxn09398	-999,6310107	0
rxn09399	0	0
rxn09412	-1000	1000
rxn09445	0	0
rxn09446	0	0
rxn09447	0	0
rxn09461	0	0
rxn09473	0	0
rxn09486	-999,9370101	1000
rxn09502	0	1000

rxn09507	0	0
rxn09531	0	0
rxn09557	0,000254683	1000
rxn09616	0,000690955	0,000690955
rxn09631	0,000254683	0,000254683
rxn09633	0,000254683	0,000254683
rxn09888	0	0
rxn09889	0	0
rxn09952	0	0
rxn09978	0	0
rxn09979	0	0
rxn09988	0	0
rxn09992	0	0
rxn09995	0	0
rxn10003	0	0,000657835
rxn10019	0	0
rxn10020	0	0
rxn10021	0	0
rxn10052	-1000	1000
rxn10054	0	999,5995158
rxn10056	0	0,000510507
rxn10058	0	0,000510507
rxn10060	0	0,000510507
rxn10091	-1000	1000
rxn10192	0	0
rxn10193	0	0
rxn10194	0	0
rxn10196	0	0
rxn10202	0	0
rxn10203	0	0
rxn10204	0	0
rxn10205	0	0
rxn10206	0	0
rxn10207	0	0
rxn10208	0	0
rxn10209	0	0
rxn10210	0	0
rxn10211	0	0
rxn10212	0	0
rxn10213	0	0
rxn10214	0	0
rxn10215	0	0
rxn10216	0	0
rxn10217	0	0
rxn10218	0	0
rxn10219	0	0
rxn10220	0	0
rxn10221	0	0
rxn10222	0	0
rxn10223	0	0

rxn10224	0	0
rxn10225	0	0
rxn10253	0	0
rxn10254	0	0
rxn10255	0	0
rxn10256	0	0
rxn10257	0	0
rxn10258	0	0
rxn10259	0	0
rxn10260	0	0
rxn10261	0	0
rxn10262	0	0
rxn10263	0	0
rxn10264	0	0
rxn10289	0	0
rxn10290	0	0
rxn10291	0	0
rxn10292	0	0
rxn10293	0	0
rxn10294	0	0
rxn10295	0	0
rxn10296	0	0
rxn10297	0	0
rxn10298	0	0
rxn10299	0	0
rxn10300	0	0
rxn10301	0	0
rxn10302	0	0
rxn10303	0	0
rxn10304	0	0
rxn10305	0	0
rxn10306	0	0
rxn10363	0	0
rxn10404	0	0
rxn10405	0	0
rxn10406	0	0
rxn10407	0	0
rxn10408	0	0
rxn10409	0	0
rxn10410	0	0
rxn10785	6,28141E-05	6,28141E-05
rxn10951	0	0,028334856
rxn11007	0,028334856	0,028334856
rxn11510	0	0
rxn11511	0	0
rxn11547	0	0
rxn11548	0	0
rxn11550	0	0
rxn11551	-1000	1000
rxn11552	-1000	1000

rxn11567	0	0
rxn11571	0	0
rxn11587	0	0
rxn11599	0	0
rxn11609	0	0
rxn11663	-1000	0
rxn11702	0	0
rxn11703	0	0
rxn11732	0	0
rxn11735	0	0
rxn11749	0	0
rxn11755	0	0
rxn11757	-999,9971425	0
rxn11759	0	999,9971425
rxn11760	-999,9971425	0
rxn11765	0	0
rxn11766	0	0
rxn11768	0	0
rxn11772	0	0
rxn11773	0	0
rxn11809	0	0
rxn11834	0	0
rxn11838	0	0
rxn11946	0	0
rxn11951	0	0
rxn11965	0	0
rxn11985	0	0
rxn12013	0	0
rxn12033	0	0
rxn12049	0	0
rxn12053	0	0
rxn12054	0	0
rxn12218	-1000	-0,000254683
rxn12221	0,000254683	1000
rxn12510	0,000657835	0,000657835
rxn12649	-999,9989813	0
rxn12707	0	0
rxn12709	0	0
rxn12778	0	0
rxn12822	-1000	0
rxn12844	0	0
rxn12845	0	0
rxn12846	0	0
rxn12847	0	0
rxn13420	0,000690955	23,28933591
rxn13421	0,000690955	23,28933591
rxn13705	0	0
rxn13734	0	0
rxn13735	0	0
rxn13741	0	0

rxn13906	-0,007702147	-0,007702147
rxn13936	0,015363179	0,01536318
rxn13974	-22,78027698	0
rxn13996	0	0
rxn14028	0	0
rxn14029	0	0
rxn14043	0	0
rxn14048	-999,999421	0
rxn14050	0	0
rxn14054	-1000	0
rxn14063	0	0
rxn14070	0	0
rxn14089	-1000	0
rxn14093	0	0
rxn14120	-1000	-0,00101873
rxn14132	0	0
rxn14147	0	0
rxn14160	0	0
rxn14191	0	0
rxn14250	0	0
rxn14270	0	0
rxn14279	0	0
rxn14297	0	0
rxn14346	0	0
rxn14399	0	0
rxn90002	-13,96688252	1000
rxn90003	0	0
rxn90004	0	0
rxn90005	-0,028845363	-0,028334856
rxn08173	0	499,9996545
Biomass_Bacteria	1,142074	1,142074006
t_Cl	0,005153038	0,005153038
t_Sulfate	0,004294198	0,004294198
t_Cu2+	0,003435359	0,003435359
t_Mg	0,008587254	0,008587254
t_Ca2+	0,005153038	0,005153038
t_NH3	-2,590644158	0
t_H2O	-34,22212259	10
t_Biomass	-1,142074006	-1,142074
t_Butyrate	-12,08769731	0
t_D-Lactate	-15,18685132	0
t_Ethanol	-1,303068753	0
t_Formate	-16,05574732	0
t_H2	0	0,5
t_L-Lactate	-15,18685132	0
t_Nitrite	0	0,1
t_Phosphate	1,517143664	2,011966651
t_Propionate	-1,521802193	0
t_O2	0	0
t_D-Glucose	0	0,5

t_CO2	-16,05574732	0
t_Acetate	-24,17539462	0
t_Succinate	-8,027873661	0
t_(S,S)-2,3-Butanediol	0	0
t_BDOH	0	0
t_H2S	-0,397709	0
Ex_Cl	-0,005153038	-0,005153038
Ex_Sulfate	-0,004294198	-0,004294198
Ex_Cu2+	-0,003435359	-0,003435359
Ex_Mg	-0,008587254	-0,008587254
Ex_Ca2+	-0,005153038	-0,005153038
Ex_NH3	0	2,590644158
Ex_H2O	-10	34,22212259
Ex_Biomass	1,142074	1,142074006
Ex_Butyrate	0	12,08769731
Ex_D-Lactate	0	15,18685132
Ex_Ethanol	0	1,303068753
Ex_Formate	0	16,05574732
Ex_H2	-0,5	0
Ex_L-Lactate	0	15,18685132
Ex_Nitrite	-0,1	0
Ex_Phosphate	-2,011966651	-1,517143664
Ex_Propionate	0	1,521802193
Ex_O2	0	0
Ex_D-Glucose	-0,5	0
Ex_CO2	0	16,05574732
Ex_Acetate	0	24,17539462
Ex_Succinate	0	8,027873661
Ex_(S,S)-2,3-Butanediol	0	0
Ex_BDOH	0	0
Ex_H2S	0	0,397709
t_Fe2	0,007983097	0,007983097
t_fe3	0,007728415	0,007728415
t_Acetaldehyde	-1,303068753	0
t_Adenosine	0	0,494822979
t_AMP	0	0,494822979
t_Amylotriose	0	0
t_BIOT	0	0
t_Choline	0	0
t_Cytidine	0	0
t_Cytosine	0	0
t_DAlanine	0	0
t_Deoxyadenosine	0	0,494822979
t_Deoxycytidine	0	0,365829146
t_Deoxyguanosine	0	0
t_Deoxyinosine	0	0
t_Deoxyuridine	0	0
t_DRibose	0	0,5
t_Glycerol	0	0
t_GSH	0	0

t_Guanine	0	0
t_H2S2O3	0	0
t_Heme	0,000254683	0,000254683
t_Homocysteine	0	0
t_HYXN	0	0,494822979
t_Inosine	0	0,494822979
t_LACT	0	0,5
t_LAlanine	-2,090644158	0,5
t_LArabinose	0	0,5
t_LArginine	-0,147661039	0,5
t_LAsparagine	-0,795322079	0,5
t_LAspartate	-2,090644158	0,5
t_LCysteine	0,102291	0,5
t_LGlutamate	-2,090644158	0,5
t_LGlutamine	-0,795322079	0,5
t_LHistidine	0,105185015	0,105185016
t_LInositol	0	0
t_LIsoleucine	-1,199394701	0,322407492
t_LLeucine	0,499999997	0,5
t_LLysine	-0,914554607	0,380767474
t_LMethionine	-0,226433304	0,171275697
t_LPhenylalanine	-2,385070838	0,205573321
t_LThreonine	-1,02154751	0,5
t_LTryptophan	-1,232245332	0,063076747
t_LTyrosine	-2,437606242	0,153037917
t_LValine	-2,120223877	0,470420283
t_Maltose	0	0,5
t_Niacin	0,002602787	0,002602787
t_Ornithine	0	0
t_PPi	0	0
t_Pyridoxol	0	0
t_XAN	0	0
t_1,3Propanediol	0	0
t_5Deoxyadenosine	0	0
t_Acetoacetate	-8,027873661	0
t_Calomide	0	0
t_Carnosine	0	0
t_Cbl	0	0
t_Citrate	0	0
t_CysGly	0	0
t_Dulcose	0	0
t_Glycine	-2,090644158	0,5
t_Glycolaldehyde	0	0
t_LProline	0,245317495	0,245317497
t_Maltohexaose	0	0
t_Methanol	0	0
t_NAcetylDglucosamine	0	0
t_PM	0	0
t_Putrescine	0	0
t_Pyridoxal	0,000254683	0,000254683

t_Riboflavin	0	0,000509365
t_Salicin	0	0
t_Sorbitol	0	0
t_Spermidine	0	0
t_Sucrose	0	0,5
t_Taurine	0	0
t_Thiamin	0	0
t_Thyminose	0	0,5
t_TRHL	0	0
t_Uracil	0	0,365829146
t_Uridine	0	0,365829146
t_Ursin	0	0
t_Mn2+	0,003435359	0,003435359
t_Formaldehyde	0	0
t_Fumarate	-8,027873661	0
t_Oxidized glutathione	0	0
t_Adenine	0	0
t_Nicotinamide	0	0
t_Co2+	0,003435359	0,003435359
t_D-Arabinose	0	0,5
t_D-Glutamate	0	0
t_Nitrate	0	0
t_Chorismate	0	0
t_Folate	0,00101873	0,00101873
t_N-Acetyl-D-mannosamine	0	0
t_Siroheme	0	0
t_Selenate	0	0
t_Menaquinone 7	0	0
t_2-Demethylmenaquinone 8	0	0
t_Menaquinone 8	0	0
t_Ubiquinone-8	0	0
t_2-Oxobutyrate	0	0
t_3MOP	0	0
t_Neu5Ac	0	0
t_Glycerol-3-phosphate	0	0
t_H+	-1000	0,5
t_indol	0	0
t_Nicotinamide ribonucleotide	0	0
t_PAN	0,000657835	0,000657835
t_Pyridoxal phosphate	0	0
t_Zn2+	0,003435359	0,003435359
t_1,2-Diacyl-sn-glycerol dioctadecanoyl	0	0
t_meso-2,6-Diaminopimelate	0	0
t_L-Serine	-2,090644158	0,5
t_D-Fructose	0	0,5
t_D-Mannose	0	0
t_Oxalate	0	0
t_L-Rhamnose	0	0
t_beta D-Galactose	0	0,5
t_L-Fucose	0	0

Ex_Fe2	-0,007983097	-0,007983097
Ex_fe3	-0,007728415	-0,007728415
Ex_Acetaldehyde	0	1,303068753
Ex_Adenosine	-0,494822979	0
Ex_AMP	-0,494822979	0
Ex_Amylotriose	0	0
Ex_BIOT	0	0
Ex_Choline	0	0
Ex_Cytidine	0	0
Ex_Cytosine	0	0
Ex_DAlanine	0	0
Ex_Deoxyadenosine	-0,494822979	0
Ex_Deoxycytidine	-0,365829146	0
Ex_Deoxyguanosine	0	0
Ex_Deoxyinosine	0	0
Ex_Deoxyuridine	0	0
Ex_DRibose	-0,5	0
Ex_Glycerol	0	0
Ex_GSH	0	0
Ex_Guanine	0	0
Ex_Heme	-0,000254683	-0,000254683
Ex_Homocysteine	0	0
Ex_HYXN	-0,494822979	0
Ex_Inosine	-0,494822979	0
Ex_LACT	-0,5	0
Ex_LAlanine	-0,5	2,090644158
Ex_LArabinose	-0,5	0
Ex_LArginine	-0,5	0,147661039
Ex_LAsparagine	-0,5	0,795322079
Ex_LAspartate	-0,5	2,090644158
Ex_LCysteine	-0,5	-0,102291
Ex_LGlutamate	-0,5	2,090644158
Ex_LGlutamine	-0,5	0,795322079
Ex_LHistidine	-0,105185016	-0,105185015
Ex_LInositol	0	0
Ex_LIsoleucine	-0,322407492	1,199394701
Ex_LLeucine	-0,5	-0,499999997
Ex_LLysine	-0,380767474	0,914554607
Ex_LMethionine	-0,171275697	0,226433304
Ex_LPhenylalanine	-0,205573321	2,385070838
Ex_LThreonine	-0,5	1,02154751
Ex_LTryptophan	-0,063076747	1,232245332
Ex_LTyrosine	-0,153037917	2,437606242
Ex_LValine	-0,470420283	2,120223877
Ex_Maltose	-0,5	0
Ex_Niacin	-0,002602787	-0,002602787
Ex_Ornithine	0	0
Ex_PPi	0	0
Ex_XAN	0	0
Ex_5Deoxyadenosine	0	0

Ex_Acetoacetate	0	8,027873661
Ex_Calomide	0	0
Ex_Carnosine	0	0
Ex_Cbl	0	0
Ex_Citrate	0	0
Ex_CysGly	0	0
Ex_Dulcose	0	0
Ex_Glycine	-0,5	2,090644158
Ex_Glycolaldehyde	0	0
Ex_LProline	-0,245317497	-0,245317495
Ex_Maltohexaose	0	0
Ex_Methanol	0	0
Ex_NAcetylDglucosamine	0	0
Ex_PM	0	0
Ex_Putrescine	0	0
Ex_Pyridoxal	-0,000254683	-0,000254683
Ex_Riboflavin	-0,000509365	0
Ex_Salicin	0	0
Ex_Sorbitol	0	0
Ex_Spermidine	0	0
Ex_Sucrose	-0,5	0
Ex_Taurine	0	0
Ex_Thiamin	0	0
Ex_Thyminose	-0,5	0
Ex_TRHL	0	0
Ex_Uracil	-0,365829146	0
Ex_Uridine	-0,365829146	0
Ex_Ursin	0	0
Ex_Mn2+	-0,003435359	-0,003435359
Ex_Formaldehyde	0	0
Ex_Fumarate	0	8,027873661
Ex_Oxidized glutathione	0	0
Ex_Adenine	0	0
Ex_Nicotinamide	0	0
Ex_Co2+	-0,003435359	-0,003435359
Ex_D-Arabinose	-0,5	0
Ex_D-Glutamate	0	0
Ex_Nitrate	0	0
Ex_Folate	-0,00101873	-0,00101873
Ex_N-Acetyl-D-mannosamine	0	0
Ex_Siroheme	0	0
Ex_Selenate	0	0
Ex_Menaquinone 7	0	0
Ex_2-Demethylmenaquinone 8	0	0
Ex_Menaquinone 8	0	0
Ex_Ubiquinone-8	0	0
Ex_Neu5Ac	0	0
Ex_H+	-0,5	1000
Ex_indol	0	0
Ex_Nicotinamide ribonucleotide	0	0

Ex_PAN	-0,000657835	-0,000657835
Ex_Zn2+	-0,003435359	-0,003435359
Ex_1,2-Diacyl-sn-glycerol dioctadecanoyl	0	0
Ex_L-Serine	-0,5	2,090644158
Ex_D-Fructose	-0,5	0
Ex_D-Mannose	0	0
Ex_Oxalate	0	0
Ex_L-Rhamnose	0	0
Ex_beta D-Galactose	-0,5	0
Ex_L-Fucose	0	0
t_Arabinan	0	0
t_Starch	0	0,005
t_octanoate	0	0
t_Melibiose	0	0,5
t_Amylose	0	0
Ex_Arabinan	0	0
Ex_Starch	-0,005	0
Ex_Melibiose	-0,5	0
Ex_Amylose	0	0
t_Raffinose_Melitose	0	0
t_Isovaleric_acid	0	0
t_H2O2	0	0
t_Nitric_oxide	0	0
Ex_Raffinose_Melitose	0	0
Ex_Isovaleric_acid	0	0
Ex_H2O2	0	0
Ex_Nitric_oxide	0	0
rxn01207_1	0	0
rxn08972	0	0
rxn08973	0	0
rxn06111	0	999,999309
rxn13726	0	0
rxn13727	0	0
rxn13729	0	0
rxn08974	0	0
rxn10122	0	0
rxn10123	0	0
rxn10124	0	0
rxn12665	0	0
rxn06097	0	0,005
t_Sulfite	0	0
Ex_Sulfite	0	0

rxn ID	minFlux	max Flux
rxn00003	-1,478394266	0
rxn00006	0	0,739324474
rxn00011	-1,478394266	0
rxn00016	0	0
rxn00020	0	1000
rxn00022	0	0,505
rxn00029	0,00101873	0,00101873
rxn00031	0	0
rxn00060	0,000254683	0,000254683
rxn00062	0	1000
rxn00063	0	1000
rxn00065	0	0
rxn00067	0	0
rxn00076	0	1000
rxn00077	0	0,000510507
rxn00085	-1000	0
rxn00097	-1000	1000
rxn00100	0,000657835	0,000657835
rxn00104	-1000	0
rxn00105	-999,9973972	1000
rxn00109	0	0
rxn00114	-0,697408921	0,455949069
rxn00119	0,368989262	1000
rxn00121	-0,000254683	0
rxn00122	0	0,000254683
rxn00124	0,000254683	0,000254683
rxn00126	0,008466195	0,912152821
rxn00127	0,007702147	0,007702147
rxn00131	-1000	1000
rxn00132	0	1000
rxn00133	0	0
rxn00134	0	1000
rxn00135	0	0
rxn00136	0	0
rxn00137	0	721,0195684
rxn00138	0	1000
rxn00139	-999,9971425	0
rxn00143	0,000509365	0,904195992
rxn00148	-18,92319896	0
rxn00151	-18,92319896	0
rxn00154	0	12,82021739
rxn00157	-12,90129322	0
rxn00159	-1000	1000
rxn00161	-1000	1000
rxn00162	0	0,701578348
rxn00165	0	6,747190411
rxn00171	0	0
rxn00173	0	19,0419444
rxn00178	-7,074762644	0

rxn00179	0	0
rxn00184	-1000	0
rxn00187	0	1000
rxn00189	0	1000
rxn00190	0	1000
rxn00191	-1000	1000
rxn00192	0	19,0419444
rxn00193	0,031494975	0,031494975
rxn00194	0,000690955	7,075453599
rxn00196	0	0
rxn00198	0	1000
rxn00199	0	1000
rxn00202	0	0
rxn00211	0	0
rxn00212	0	999,6310107
rxn00213	-1000	18,52136593
rxn00214	-1,5	0
rxn00216	0	1000
rxn00221	0	1000
rxn00222	0	1000
rxn00224	0,000254683	0,000254683
rxn00225	-19,0419444	0
rxn00227	0	19,0419444
rxn00239	0,238807673	19,19125515
rxn00247	0	0,701578348
rxn00250	-0,701613752	-3,54043E-05
rxn00256	-0,701578348	0
rxn00259	0	0
rxn00260	3,54043E-05	0,701613752
rxn00269	0	1,478394266
rxn00272	-1000	1000
rxn00275	-1000	1000
rxn00283	0,027731841	1,506126107
rxn00290	-6,348005753	-0,000690955
rxn00292	0	19,0419444
rxn00293	-999,9370101	1000
rxn00295	-1000	999,9370101
rxn00301	0	18,95244747
rxn00303	0	0
rxn00304	-18,92319896	0
rxn00307	0	0
rxn00313	0	0,701578348
rxn00322	0	0
rxn00324	-1,478394266	0
rxn00328	0	0
rxn00333	0,000254683	1,478648949
rxn00337	0,031494975	0,733073323
rxn00340	0	1000
rxn00342	0	1000
rxn00347	0	0,701578348

rxn00350	-0,000254683	-0,000254683
rxn00358	0	0
rxn00359	0	721,0195684
rxn00360	0	1000
rxn00361	0	1000
rxn00362	0	0
rxn00363	0	1000
rxn00364	-999,6310107	18,92185017
rxn00365	0	1000
rxn00367	0	729,9077087
rxn00368	0	1000
rxn00369	0	18,95244747
rxn00379	0	721,0195684
rxn00391	0	999,9997453
rxn00392	0,000254683	1000
rxn00405	0	0
rxn00410	-999,8225103	999,8085005
rxn00411	-18,92319896	0
rxn00412	0	1000
rxn00414	0	0,701578348
rxn00416	0	1000
rxn00420	0	0
rxn00422	-1000	1000
rxn00423	0	6,747190411
rxn00424	-1000	1000
rxn00426	0	0
rxn00427	0	0,903686627
rxn00433	0	0
rxn00436	0	721,0195684
rxn00437	0	0
rxn00440	0,000254683	721,0198231
rxn00453	0	1000
rxn00456	0	1000
rxn00459	-0,435542943	18,49635023
rxn00460	-18,92319896	0
rxn00461	0,031494975	0,031494975
rxn00463	0	999,6310107
rxn00469	0	19,0419444
rxn00470	0,045698949	0,045698949
rxn00474	0	0
rxn00490	0	0
rxn00493	-1,478394266	0
rxn00499	-12,6946296	0
rxn00500	-12,6946296	0
rxn00506	0	0
rxn00512	-1,478394266	0
rxn00514	0	0
rxn00517	-18,92319896	0
rxn00527	-1,478394266	0
rxn00533	-999,9999646	3,54043E-05

rxn00541	0	0
rxn00543	0	0
rxn00545	0	1000
rxn00547	0	1
rxn00549	0	1000
rxn00551	0	1000
rxn00552	-0,06298995	999,9370101
rxn00554	0	732,7227023
rxn00555	0	1000
rxn00556	0	1000
rxn00557	0	1000
rxn00558	-1000	1000
rxn00559	0	0
rxn00565	0	0
rxn00566	0	1000
rxn00567	-0,455949069	-0,004169427
rxn00575	0	0,5
rxn00585	0	0
rxn00598	-0,000254683	-0,000254683
rxn00606	0	0
rxn00608	0	1000
rxn00611	-5,625724589	0
rxn00612	-5,625724589	0
rxn00615	0	0
rxn00616	0	5,625724589
rxn00622	0	0
rxn00633	0	0
rxn00641	0	0
rxn00642	0	0
rxn00647	0	0
rxn00649	0	6,747190411
rxn00650	-0,000254683	-0,000254683
rxn00653	0	0
rxn00670	0	0,547712428
rxn00673	0	0
rxn00684	0	0
rxn00685	0	999,9989813
rxn00686	0	0
rxn00687	0	999,9989813
rxn00689	0	0
rxn00690	0	13,18285158
rxn00692	-0,260507082	6,486683329
rxn00693	0	0,848818536
rxn00695	-1000	1000
rxn00698	-1000	0
rxn00701	0	1000
rxn00704	-1000	1,5
rxn00707	0	1000
rxn00708	0	1000
rxn00709	0	1000

rxn00710	0	0
rxn00711	-999,9971425	0
rxn00712	0	1000
rxn00713	0	18,95244747
rxn00714	0	0
rxn00715	0	1000
rxn00726	0	0,739197133
rxn00727	0	0,739197133
rxn00735	0	0
rxn00737	0	0,218478759
rxn00740	0	1000
rxn00741	0	0
rxn00742	-1000	0,329233669
rxn00747	-5,793218746	0,264873958
rxn00748	0	0
rxn00756	0	0
rxn00758	0	0
rxn00762	0	0
rxn00763	0	0
rxn00765	0	0
rxn00770	0,002857469	1000
rxn00772	0	1000
rxn00775	0	0
rxn00777	-0,898727169	0,326208695
rxn00778	-1000	1000
rxn00781	-0,606844766	11,74915982
rxn00785	-0,162977006	1,092034429
rxn00786	-0,291578306	5,629987057
rxn00789	0	0
rxn00790	-0,000254683	-0,000254683
rxn00791	-0,739197133	0
rxn00792	0	0
rxn00799	-0,433085887	6,609956509
rxn00800	-0,26805619	0,433522159
rxn00802	0	0,369598567
rxn00806	0	0
rxn00808	0	1000
rxn00816	0	0,5
rxn00817	0	0,5
rxn00818	0	0
rxn00819	0	0
rxn00827	0	0
rxn00829	0,000690955	0,000690955
rxn00830	6,28141E-05	6,28141E-05
rxn00831	0	999,9971425
rxn00832	0	0
rxn00834	0,268056189	0,26805619
rxn00836	-999,9971425	0
rxn00838	-0,26805619	0,433522159
rxn00851	0	1000

rxn00855	0	0
rxn00867	0	0
rxn00869	0	0
rxn00871	0	4,897085859
rxn00872	-4,897085859	0
rxn00874	0	0
rxn00879	0	0
rxn00881	0	0
rxn00882	0	0
rxn00883	0	0
rxn00889	0	0
rxn00890	0	0
rxn00898	0	1,478394266
rxn00902	0	0
rxn00903	-1000	1000
rxn00904	-1000	1000
rxn00907	-13,18234221	0,000509365
rxn00908	-6,830717422	0,080375747
rxn00909	-4,897085859	0,849073219
rxn00910	-5,625724589	0
rxn00913	0	1000
rxn00915	-999,9971425	0
rxn00916	-999,7319438	721,2876246
rxn00917	0	1000
rxn00918	0	0
rxn00925	0	0
rxn00926	0	0,701578348
rxn00927	-1000	1000
rxn00929	-1000	1000
rxn00931	-1000	1000
rxn00938	0	721,0195684
rxn00942	0	1000
rxn00943	0	0
rxn00946	0	0
rxn00950	-1000	0,218478759
rxn00952	0	1000
rxn00955	0,000509365	0,904195992
rxn00957	0,000254683	0,000254683
rxn00962	0,000254683	0,000254683
rxn00973	-1000	1000
rxn00974	-1000	1000
rxn00977	0	0
rxn00979	0,000254683	0,000254683
rxn00980	0	0
rxn00983	0	0
rxn00985	-0,547712428	0
rxn00991	-0,000690955	-0,000690955
rxn01000	0	1,478394266
rxn01008	0	0
rxn01011	-6,747190411	0

rxn01013	-6,747190411	0
rxn01016	0	0
rxn01018	0	0
rxn01019	0,004169427	0,455949069
rxn01021	0	0
rxn01022	0,007702147	0,007702147
rxn01029	0	0
rxn01034	0	0
rxn01041	-1000	1000
rxn01042	-1000	1000
rxn01049	0	1000
rxn01069	0	0
rxn01073	0	0
rxn01100	-1000	0
rxn01101	0	0
rxn01103	0	1000
rxn01106	-11,74915982	0,606844766
rxn01108	-1000	1000
rxn01109	-1000	1000
rxn01114	0	0
rxn01116	-0,898727169	0,326208695
rxn01119	0	0
rxn01124	0	0
rxn01133	0	0
rxn01137	0	0,701578348
rxn01138	-1000	1000
rxn01139	0	0
rxn01169	0	1000
rxn01171	-1000	1000
rxn01192	0,000254683	0,000254683
rxn01199	0	0
rxn01200	0	1000
rxn01201	-7,075453599	-0,000690955
rxn01204	0,000690955	7,075453599
rxn01210	0	0
rxn01211	-13,1825969	0,000509365
rxn01213	6,28141E-05	6,28141E-05
rxn01225	0	721,0195684
rxn01226	-999,9707515	1000
rxn01228	0	0
rxn01236	-4,897085859	0
rxn01241	0	0
rxn01255	0,000254683	1,478648949
rxn01256	0	1,478394266
rxn01257	0	0
rxn01259	0	0
rxn01265	-999,9997453	-0,002602787
rxn01268	0	1,478394266
rxn01274	0	0
rxn01276	0	0

rxn01278	0	0
rxn01286	0	0
rxn01299	-1000	1000
rxn01300	0	0
rxn01303	0	0
rxn01304	0	0
rxn01305	0	0
rxn01316	0	0
rxn01332	0,000254683	1,478648949
rxn01334	0	0,656029778
rxn01343	0	0,656029778
rxn01346	0	0,656029778
rxn01347	0	0,656029778
rxn01348	0	0,656029778
rxn01351	0	1000
rxn01352	-1000	-0,029248515
rxn01354	-18,92319896	0
rxn01355	0	0
rxn01358	-1000	1000
rxn01361	0	0
rxn01362	0	0
rxn01366	-0,33749429	1000
rxn01367	0	0
rxn01368	0	721,0195684
rxn01370	0	1000
rxn01374	0	0
rxn01379	0	0
rxn01387	-1000	0
rxn01388	-1000	1000
rxn01396	0	0
rxn01406	0,007702147	0,007702147
rxn01423	0	0
rxn01426	0	0
rxn01434	0	0,369598567
rxn01445	0	999,9707515
rxn01446	-0,029248515	-0,029248515
rxn01452	-999,999309	0
rxn01459	0	0
rxn01465	0	0
rxn01466	6,28141E-05	6,28141E-05
rxn01476	0	0
rxn01484	0	19,0419444
rxn01485	-19,10493434	-0,062989949
rxn01486	0	0
rxn01492	0	0
rxn01500	-0,000690955	-0,000690955
rxn01506	-19,0419444	0
rxn01509	-999,9707515	30,67235877
rxn01510	0	1000
rxn01513	0,028334856	0,028334856

rxn01518	0,028334856	1000
rxn01519	0	0
rxn01521	0	999,9716651
rxn01539	-721,0198231	-0,000254683
rxn01544	-999,9971425	0
rxn01545	-1000	1000
rxn01548	-999,9707515	1000
rxn01549	0	0
rxn01562	0	0
rxn01575	-0,547712428	0
rxn01594	0	0
rxn01601	0	0
rxn01602	0	0
rxn01603	0	0
rxn01615	0	0
rxn01629	-0,00203746	-0,00203746
rxn01636	-18,80067555	0,501648018
rxn01637	-0,501648018	-0,049868376
rxn01643	-0,733073323	-0,031494975
rxn01644	0,031494975	0,733073323
rxn01646	-1000	721,0195684
rxn01647	0	999,9971425
rxn01649	-1000	1000
rxn01653	0	0
rxn01667	0	0
rxn01669	0	999,9973972
rxn01670	0	721,0195684
rxn01675	0	0
rxn01679	0	0
rxn01682	0	0
rxn01683	-1000	1000
rxn01684	-1000	1000
rxn01686	0	0
rxn01704	0	0
rxn01706	0	0
rxn01735	0	0
rxn01737	0	0
rxn01738	0	0
rxn01739	0,000254683	1,478648949
rxn01740	-1,478648949	-0,000254683
rxn01741	0	0
rxn01757	0	0
rxn01763	0	0
rxn01778	0	0
rxn01791	0	0
rxn01799	-0,028334856	0,029248515
rxn01800	0	0,057583371
rxn01807	0	0
rxn01816	0	1000
rxn01831	0	0

rxn01842	0	0
rxn01851	0	0
rxn01858	0	0,701578348
rxn01859	-0,701578348	0,057583371
rxn01860	0	0
rxn01870	0	0
rxn01892	0	0
rxn01895	0	0
rxn01906	0	0
rxn01917	0,049868376	0,501648018
rxn01932	0	0
rxn01937	0	0
rxn01945	0	0
rxn01946	0	0
rxn01951	0	19,0419444
rxn01961	0	999,9971425
rxn01962	0	0
rxn01964	0	0,739197133
rxn01967	0	0
rxn01972	0,031494975	19,07343937
rxn01973	-6,450646608	0
rxn01974	0,031494975	0,733073323
rxn01977	-1000	1000
rxn01981	0	0
rxn01982	0	0
rxn01985	0	0,701578348
rxn01987	0	0
rxn01991	0	0
rxn01996	0	0
rxn01997	0	0
rxn01998	0	0
rxn01999	0	0
rxn02000	0	0
rxn02003	0	0
rxn02007	0	0
rxn02008	0,031494975	0,031494975
rxn02011	0,031494975	0,031494975
rxn02015	0	0
rxn02020	0	0
rxn02023	0	0
rxn02033	0	0
rxn02035	0	0
rxn02046	0	0
rxn02071	0	0
rxn02091	0	0
rxn02093	0	0
rxn02106	0	0
rxn02122	0	0
rxn02123	0	0
rxn02128	0	0

rxn02138	0	0
rxn02139	0	0
rxn02143	0,000254683	0,000254683
rxn02144	0,000254683	0,000254683
rxn02154	0	999,9973972
rxn02155	0,002602787	1000
rxn02160	0	0
rxn02166	0	0
rxn02167	0	999,999309
rxn02171	0,000690955	7,075453599
rxn02175	0,000657835	1000
rxn02176	0	999,9993422
rxn02185	-1,478394266	0,547712428
rxn02186	0	1,478394266
rxn02187	0	0
rxn02195	0	0
rxn02202	0	0
rxn02203	0	0
rxn02209	0	0
rxn02212	0,000254683	1,478648949
rxn02213	0,000254683	1,478648949
rxn02228	0	0
rxn02262	0	0
rxn02264	0,000254683	0,000254683
rxn02277	0	0
rxn02284	-0,031494975	0
rxn02285	-0,031494975	0
rxn02286	0,031494975	0,031494975
rxn02288	0	0
rxn02293	0	0
rxn02302	-1000	0
rxn02305	0,000254683	0,000254683
rxn02314	0	1000
rxn02315	0	1000
rxn02316	0	1000
rxn02317	-1000	0
rxn02320	0	0
rxn02322	0,000690955	0,000690955
rxn02339	0	0
rxn02341	0,000657835	0,000657835
rxn02350	0	0
rxn02351	0	0
rxn02356	-1000	1000
rxn02358	-1000	1000
rxn02363	0	0
rxn02369	-0,000254683	-0,000254683
rxn02373	-1000	1000
rxn02380	-1000	1000
rxn02400	0	999,9971425
rxn02405	0	0

rxn02409	0	0
rxn02438	-1000	0
rxn02440	0	1000
rxn02449	0	0
rxn02454	0	0
rxn02465	-0,501648018	-0,049868376
rxn02473	0	0
rxn02476	0,000254683	1,478648949
rxn02483	0,000254683	0,000254683
rxn02484	0,000254683	0,000254683
rxn02495	0	0
rxn02503	0	0
rxn02504	0	0
rxn02507	0	0,739197133
rxn02508	0	0,739197133
rxn02518	0	0
rxn02521	0	0
rxn02522	0	0
rxn02571	0	0
rxn02581	0	0
rxn02596	0	0
rxn02597	0	0
rxn02632	0	0
rxn02663	0	0
rxn02679	0	0
rxn02718	0	0
rxn02720	0	0
rxn02729	0	0
rxn02749	0	0
rxn02751	0	0
rxn02760	0	0
rxn02762	0	0
rxn02775	0	0
rxn02789	0	0
rxn02795	0	0
rxn02796	0	0
rxn02798	0	0
rxn02803	0	0
rxn02811	0	0
rxn02821	0	0
rxn02822	0	0
rxn02832	0	0
rxn02834	0	0
rxn02835	0	0
rxn02853	0	0
rxn02866	0	0
rxn02875	0	0
rxn02895	0,000254683	0,000254683
rxn02897	0	0
rxn02914	0	0

rxn02922	0	0
rxn02928	-1000	999,968505
rxn02929	-1000	999,968505
rxn02931	0	0
rxn02936	0	0
rxn02937	0,000254683	0,000254683
rxn02943	0	0
rxn02946	0	0
rxn02990	0	0
rxn03004	0	0,000254683
rxn03005	-0,000254683	0
rxn03030	0,031494975	19,07343937
rxn03031	-6,450646608	0
rxn03039	0	0
rxn03047	0	0
rxn03057	0,007702147	0,007702147
rxn03062	0	0
rxn03064	0	0
rxn03066	0	0
rxn03068	0	0
rxn03075	0,000254683	0,000254683
rxn03084	0,000254683	0,000254683
rxn03086	-19,07343937	-0,031494975
rxn03087	0	6,450646608
rxn03094	0	0
rxn03095	0	0
rxn03102	0	0
rxn03106	0	0
rxn03108	0,000254683	0,000254683
rxn03132	0	0
rxn03135	0	0
rxn03136	0	0
rxn03137	0	0
rxn03140	0	0
rxn03141	0	0
rxn03146	0	0
rxn03147	0	0
rxn03150	0	0
rxn03164	0,031494975	0,031494975
rxn03175	0	0
rxn03182	0	0
rxn03194	0	0,547712428
rxn03251	0	0
rxn03253	0	0
rxn03255	-1000	0
rxn03256	0	1000
rxn03263	0	0
rxn03264	0	0
rxn03269	0	0
rxn03273	0	0

rxn03282	0	0
rxn03309	-1000	0
rxn03310	0	1000
rxn03333	0	0
rxn03354	0	0
rxn03371	0,020847133	2,279745347
rxn03372	0	0
rxn03373	0	0
rxn03374	0	0
rxn03379	0	0
rxn03382	0	0
rxn03383	0	0
rxn03384	0	0
rxn03387	0	0
rxn03393	0	0
rxn03395	0	0
rxn03397	0	0
rxn03402	0	0
rxn03405	0	0
rxn03406	0	0
rxn03407	0	0
rxn03408	0,031494975	0,031494975
rxn03409	0	0
rxn03423	0	0
rxn03435	-0,547712428	0
rxn03436	0	0,547712428
rxn03437	0	0,547712428
rxn03439	0	0
rxn03445	0	0
rxn03446	0	0
rxn03462	0	0
rxn03465	0	0
rxn03467	0	0
rxn03468	0	0
rxn03481	0	0
rxn03482	0	0
rxn03483	0	0
rxn03491	0	0
rxn03512	0	0
rxn03513	0	0
rxn03514	0	0
rxn03535	0	0
rxn03536	0	0
rxn03537	0	0
rxn03538	0	0
rxn03540	0	0
rxn03548	0	1000
rxn03549	0	0
rxn03552	0	0
rxn03553	0	0

rxn03558	0	0
rxn03598	0	0
rxn03599	0	0
rxn03634	0	0
rxn03638	0,062989949	19,10493434
rxn03641	0,000690955	7,075453599
rxn03642	0,000690955	7,075453599
rxn03838	0	0
rxn03852	0	0
rxn03861	0	0
rxn03891	6,28141E-05	6,28141E-05
rxn03901	0,031494975	0,031494975
rxn03902	0	0
rxn03903	0	0
rxn03904	0,031494975	0,031494975
rxn03907	0	0
rxn03908	0	0
rxn03909	0	0
rxn03910	0	0
rxn03933	0	0
rxn03951	0	1000
rxn03958	0	0
rxn03962	0	0
rxn03964	0	0
rxn03974	-0,028334856	0
rxn03975	-0,028334856	0
rxn03978	0,004169427	0,455949069
rxn04045	0	0
rxn04046	0	0
rxn04047	0	0
rxn04048	0	0
rxn04052	0	0
rxn04068	0	0
rxn04082	0	0
rxn04092	0	1,478394266
rxn04113	0	0
rxn04142	0	0
rxn04234	0	0
rxn04288	0	0
rxn04308	0	0
rxn04384	0	0
rxn04385	0	0
rxn04413	0	0
rxn04432	0	0
rxn04443	0	0
rxn04447	0	0
rxn04604	0	0
rxn04674	0	0
rxn04676	0	1000
rxn04678	-1000	0

rxn04703	0	0
rxn04704	0	0
rxn04726	0	0
rxn04736	0	0
rxn04750	0	0
rxn04794	0	0,547712428
rxn04822	0	0
rxn04865	0	0
rxn04866	0	0
rxn04954	-5,625724589	0
rxn04960	0	0
rxn05010	0	0
rxn05028	6,28141E-05	6,28141E-05
rxn05029	0	0
rxn05030	6,28141E-05	6,28141E-05
rxn05039	0	0
rxn05050	0	0
rxn05054	0	0
rxn05092	0,007702147	0,007702147
rxn05104	0,007702147	0,007702147
rxn05105	0,007702147	0,007702147
rxn05106	0,007702147	0,007702147
rxn05108	0,007702147	0,007702147
rxn05110	0	0
rxn05115	0	0
rxn05116	0	1000
rxn05234	0	0
rxn05236	0	0
rxn05269	0	0
rxn05289	0	0
rxn05322	0	0
rxn05323	0	0
rxn05324	0	0
rxn05325	0	0
rxn05326	0	0
rxn05327	0	0
rxn05328	0	0
rxn05329	0	0
rxn05330	0	0
rxn05331	0	0
rxn05332	0	0
rxn05333	0	0
rxn05334	0	0
rxn05335	0	0
rxn05336	0	0
rxn05337	0	0
rxn05338	0	0
rxn05339	0	0
rxn05340	0	0
rxn05341	0	0

rxn05342	0	0
rxn05343	0	0
rxn05344	0	0
rxn05345	0	0
rxn05346	0	0
rxn05347	0	0
rxn05348	0	0
rxn05350	0	0
rxn05457	0	1000
rxn05465	0	0
rxn05733	0	0
rxn05740	-1000	1000
rxn05759	-0,5	0
rxn05760	-2,268186413	1000
rxn05762	0	0
rxn05763	0	0
rxn05778	-1,478394266	0
rxn05779	-1,478394266	0
rxn05794	-1000	0
rxn05824	0	0
rxn05853	0	0
rxn05854	0	0
rxn05871	0	0
rxn05872	0	0
rxn05874	0	0
rxn05899	0	0
rxn05901	0	0
rxn05918	0	0
rxn05934	0	0
rxn05937	-1000	1000
rxn05939	0,000579032	1000
rxn05953	0	0
rxn05957	0	1000
rxn05958	0	0
rxn05962	0	0
rxn05970	0	0
rxn05978	0	0
rxn05994	0	0
rxn06005	0	0
rxn06023	0	0
rxn06043	0	0
rxn06044	0	0
rxn06045	0	0
rxn06077	0	0
rxn06078	0	0
rxn06080	0	0
rxn06090	0	0
rxn06091	0	0
rxn06096	0	0
rxn06108	-1000	-0,000436272

rxn06109	-6,451082881	-0,000436272
rxn06139	0	0
rxn06140	0	0
rxn06181	0	1000
rxn06182	0	1000
rxn06195	0	0,903686627
rxn06196	0	0,903686627
rxn06200	0	0
rxn06201	0	0
rxn06206	0	0
rxn06209	0	0
rxn06212	0	0
rxn06240	0	0
rxn06280	0	0
rxn06285	0	0
rxn06293	0	0
rxn06298	0	0
rxn06299	0	0
rxn06300	0	0
rxn06316	0	0
rxn06328	0	0
rxn06341	0	0
rxn06347	0	0
rxn06373	0	0
rxn06376	0	0
rxn06381	0	0
rxn06394	0	0
rxn06400	0	0
rxn06403	0	0
rxn06432	0	0
rxn06434	0	0
rxn06435	0	0
rxn06437	0	0
rxn06438	0	0
rxn06439	0	0
rxn06440	0	0
rxn06441	0	0
rxn06443	0	0
rxn06444	0	0
rxn06445	0	0
rxn06446	0	0
rxn06447	0	0
rxn06448	0	0
rxn06449	0	0
rxn06459	0	0
rxn06472	0	0
rxn06485	0	0
rxn06493	0	0
rxn06500	0	0
rxn06538	0	0

rxn06556	0	1000
rxn06565	0	0
rxn06581	0	0
rxn06584	0	0
rxn06591	0,00203746	0,00203746
rxn06595	0	0
rxn06624	0	0
rxn06648	0	0
rxn06660	0	0
rxn06664	0	0
rxn06672	0	1000
rxn06673	0	1000
rxn06691	0	0
rxn06701	0	0
rxn06723	0	0
rxn06726	0	0
rxn06737	0	0
rxn06741	0	0
rxn06751	0	0
rxn06752	0	0
rxn06760	0	0
rxn06768	0	0
rxn06798	0	0
rxn06799	0	0
rxn06820	0	0
rxn06823	0	0
rxn06831	0	0
rxn06850	0	0
rxn06864	0	0
rxn06882	0	0
rxn06883	0	0
rxn06887	0	0
rxn06889	0	1000
rxn06890	0	0
rxn06934	0	0
rxn06936	0	0
rxn06937	0,00203746	0,00203746
rxn06947	0	0
rxn06979	0	0
rxn07053	0	1,478394266
rxn07056	0	0
rxn07059	0	0
rxn07172	0	0
rxn07193	0	0
rxn07223	0	0
rxn07241	0	0
rxn07251	0	0
rxn07267	0	0
rxn07292	0	0
rxn07332	0	0

rxn07437	0	0
rxn07438	0	0
rxn07439	0	0
rxn07441	0	19,0419444
rxn07456	0	0,701578348
rxn07466	-1000	1000
rxn07473	0	0
rxn07474	0	0
rxn07475	0	0
rxn07476	0	0
rxn07489	0	0
rxn07573	0	0
rxn07577	0	0
rxn07578	0	0
rxn07579	0	0
rxn07580	0	0
rxn07586	0	0
rxn07587	0	0
rxn07679	0	0
rxn07683	0	0
rxn07687	0	0
rxn07804	0	0
rxn07807	0	0
rxn07832	0	0
rxn07846	0	0
rxn07946	0	0
rxn07947	0	0
rxn07948	0	0
rxn07949	0	0
rxn07950	0	0
rxn07951	0	0
rxn07952	0	0
rxn07960	0	0
rxn07961	0	0
rxn07962	0	0
rxn07963	0	0
rxn07964	0	0
rxn07965	0	0
rxn07966	0	0
rxn07987	0	0
rxn07989	0	0
rxn07991	0	0
rxn07992	0	0
rxn07993	0	0
rxn07994	0	0
rxn08014	0	1000
rxn08015	0	1000
rxn08016	0	1000
rxn08017	0	1000
rxn08018	0	1000

rxn08019	0	1000
rxn08020	0	1000
rxn08021	0	1000
rxn08022	0	1000
rxn08025	0	0
rxn08035	0	0
rxn08038	0	0
rxn08040	0	0
rxn08043	0	0,547712428
rxn08044	0	0
rxn08067	-1000	1000
rxn08083	0	0
rxn08084	0	0
rxn08085	0	0
rxn08086	0	0
rxn08087	0	0
rxn08088	0	0
rxn08089	0	0
rxn08094	0	999,999421
rxn08126	0	0
rxn08127	0	0
rxn08128	0	0
rxn08129	0	0
rxn08171	0	0
rxn08194	-442,0391368	1000
rxn08206	0	0
rxn08207	0	0
rxn08208	0	0
rxn08209	0	0
rxn08294	0	1000
rxn08295	0	1000
rxn08296	0	1000
rxn08297	0	1000
rxn08298	0	1000
rxn08299	0	1000
rxn08300	0	1000
rxn08306	0	0
rxn08307	0	0
rxn08308	0	0
rxn08309	0	0
rxn08310	0	0
rxn08311	0	0
rxn08312	0	0
rxn08352	0	0
rxn08386	0	0
rxn08390	0	0
rxn08392	0	0
rxn08394	0	0
rxn08396	0	0
rxn08398	0	0

rxn08413	0	0
rxn08433	0	1000
rxn08438	0	1000
rxn08444	0	0
rxn08519	0,057583371	0,057583371
rxn08546	0	1000
rxn08547	0	1000
rxn08548	0	1000
rxn08549	0	1000
rxn08550	0	1000
rxn08551	0	1000
rxn08552	0	1000
rxn08571	0	1000
rxn08582	0	0,5
rxn08605	0	0
rxn08607	0	0
rxn08615	-1000	1000
rxn08647	0	6,747190411
rxn08668	0	0
rxn08669	0	0
rxn08764	0	0,547712428
rxn08796	0	1000
rxn08797	0	1000
rxn08798	0	1000
rxn08799	0	1000
rxn08800	0	1000
rxn08801	0	1000
rxn08802	0	1000
rxn08803	0	0
rxn08804	0	0
rxn08805	0	0
rxn08806	0	0
rxn08807	0	0
rxn08808	0	0
rxn08809	0	0
rxn08810	0	0
rxn08811	0	0
rxn08812	0	0
rxn08813	0	0
rxn08814	0	0
rxn08815	0	0
rxn08816	0	0
rxn08817	0	0
rxn08818	0	0
rxn08819	0	0
rxn08820	0	0
rxn08821	0	0
rxn08822	0	0
rxn08823	0	0
rxn08838	0	0

rxn08839	0	0
rxn08840	0	0
rxn08841	0	0
rxn08842	0	0
rxn08843	0	0
rxn08844	0	0
rxn08845	0	0
rxn08846	0	0
rxn08847	0	0
rxn08848	0	0
rxn08849	0	0
rxn08850	0	0
rxn08851	0	0
rxn08857	0	0
rxn08889	0,000768616	0,000768616
rxn08890	0,006222019	0,006222019
rxn08891	0,000768616	0,000768616
rxn08892	-999,9854054	1000
rxn08893	-999,9923184	999,993087
rxn08894	-999,9854054	1000
rxn08897	-0,006912974	-0,006912974
rxn08926	0,000690955	0,000690955
rxn08927	-999,9984639	999,9869415
rxn08928	-999,9854054	1000
rxn08929	0,00153609	0,00153609
rxn08930	0	0
rxn08958	0,000768616	0,000768616
rxn09010	0	0
rxn09016	0	18,95244747
rxn09062	0	1000
rxn09063	0	1000
rxn09064	0	1000
rxn09065	0	1000
rxn09066	0	1000
rxn09067	0	1000
rxn09068	0	1000
rxn09101	0	0
rxn09102	0	0
rxn09103	0	0
rxn09104	0	0
rxn09105	0	0
rxn09106	0	0
rxn09107	0	0
rxn09108	0	0
rxn09109	0	0
rxn09110	0	0
rxn09111	0	0
rxn09112	0	0
rxn09113	0	0
rxn09114	0	0

rxn09176	-1000	1000
rxn09197	0	0
rxn09198	0	0
rxn09199	0	0
rxn09200	0	0
rxn09201	0	0
rxn09202	0	0
rxn09203	0	0
rxn09205	0	0
rxn09206	0	0
rxn09207	0	0
rxn09208	0	0
rxn09209	0	0
rxn09210	0	0
rxn09211	0	0
rxn09235	0,028334856	0,028334856
rxn09237	0,029248515	0,029248515
rxn09240	0	18,95244747
rxn09264	0	0
rxn09265	0	0
rxn09340	0	0
rxn09341	0	999,6310107
rxn09348	0	999,6310107
rxn09355	0	0
rxn09398	-1000	18,52136593
rxn09399	0	0
rxn09412	-1000	1000
rxn09473	0	0
rxn09486	-999,9370101	1000
rxn09502	0	1000
rxn09507	0	0
rxn09557	0,000254683	0,000254683
rxn09616	0,000690955	0,000690955
rxn09632	0	721,0195684
rxn09633	0,000254683	0,000254683
rxn09888	0	0
rxn09889	0	0
rxn09949	0	0
rxn09952	0	0
rxn09978	0	0
rxn09979	0	0
rxn09988	0	0
rxn09992	0	0
rxn09995	0	0
rxn10003	0,000657835	0,000657835
rxn10052	-1000	1000
rxn10054	0	999,6310107
rxn10056	0	0,000510507
rxn10058	0	0,000510507
rxn10060	0	0,000510507

rxn10091	-1000	442,0391368
rxn10107	0	0
rxn10110	0	0
rxn10111	0	0
rxn10192	0	0
rxn10193	0	0
rxn10194	0	0
rxn10196	0	0
rxn10202	0	0
rxn10203	0	1000
rxn10204	0	0
rxn10205	0	0
rxn10206	0	0
rxn10207	0	0
rxn10208	0	0
rxn10209	0	0
rxn10210	0	0
rxn10211	0	0
rxn10212	0	0
rxn10213	0	0
rxn10214	0	0
rxn10215	0	0
rxn10216	0	0
rxn10217	0	0
rxn10218	0	0
rxn10219	0	0
rxn10220	0	0
rxn10221	0	0
rxn10222	0	0
rxn10223	0	0
rxn10224	0	0
rxn10225	0	0
rxn10226	0	0
rxn10227	0	0
rxn10228	0	0
rxn10229	0	0
rxn10230	0	0
rxn10231	0	0
rxn10232	0	0
rxn10233	0	0
rxn10234	0	0
rxn10235	0	0
rxn10236	0	0
rxn10237	0	0
rxn10238	0	1000
rxn10239	0	1000
rxn10240	0	1000
rxn10241	0	1000
rxn10242	0	1000
rxn10243	0	1000

rxn10253	0	1000
rxn10254	0	1000
rxn10255	0	1000
rxn10256	0	1000
rxn10257	0	1000
rxn10258	0	1000
rxn10259	0	0
rxn10260	0	0
rxn10261	0	0
rxn10262	0	0
rxn10263	0	0
rxn10264	0	0
rxn10265	0	0
rxn10266	0	0
rxn10267	0	0
rxn10268	0	0
rxn10269	0	0
rxn10270	0	0
rxn10289	0	0
rxn10290	0	0
rxn10291	0	0
rxn10292	0	0
rxn10293	0	0
rxn10294	0	0
rxn10295	0	0
rxn10296	0	0
rxn10297	0	0
rxn10298	0	0
rxn10299	0	0
rxn10300	0	0
rxn10301	0	0
rxn10302	0	0
rxn10303	0	0
rxn10304	0	0
rxn10305	0	0
rxn10306	0	0
rxn10363	0	0
rxn10410	0	0
rxn10476	0	0
rxn10816	0,000254683	0,000254683
rxn11007	0,028334856	0,028334856
rxn11513	0	0
rxn11547	0	0
rxn11548	0	0
rxn11550	0	0
rxn11564	0	0
rxn11567	0	0
rxn11571	0	0
rxn11587	0	0
rxn11609	0	0

rxn11612	0	0
rxn11641	0	0
rxn11642	0	0
rxn11663	-1000	0
rxn11676	0	0
rxn11677	0	0
rxn11678	0	0
rxn11703	0	0
rxn11711	0	0
rxn11712	0	0
rxn11713	0	0
rxn11716	0	0
rxn11728	0	0
rxn11731	0	0
rxn11732	0	0
rxn11749	0	0
rxn11757	-999,9971425	0
rxn11759	0	999,9971425
rxn11760	-999,9971425	0
rxn11761	0	0
rxn11765	0	0
rxn11766	0	0
rxn11768	0	0
rxn11772	0	0
rxn11773	0	0
rxn11788	0	0
rxn11890	0	0
rxn11894	0	0
rxn11946	0	0
rxn11951	0	0
rxn11962	0	0
rxn12008	-6,28141E-05	-6,28141E-05
rxn12218	-0,000254683	-0,000254683
rxn12221	0,000254683	0,000254683
rxn12239	0,000254683	0,000254683
rxn12510	0,000657835	0,000657835
rxn12649	-999,9989813	0
rxn12778	0	0
rxn12822	-1000	0
rxn13147	0,000254683	0,000254683
rxn13420	0,000690955	1000
rxn13421	0,000690955	1000
rxn13477	6,28141E-05	6,28141E-05
rxn13705	0	0
rxn13741	0	0
rxn13936	0,015363179	0,01536318
rxn13974	-12,82021739	0
rxn13994	-2,279745347	-0,020847133
rxn14012	6,28141E-05	6,28141E-05
rxn14029	0	0

rxn14043	0	0
rxn14048	-999,999421	0
rxn14050	0	0
rxn14054	-1000	0
rxn14058	-0,227974535	-0,002084713
rxn14063	0	0
rxn14070	0	0
rxn14089	-1000	0
rxn14093	0	0
rxn14120	-1000	-0,00101873
rxn14132	0	0
rxn14136	0	0
rxn14250	0	0
rxn14270	0	0
rxn14279	0	0
rxn14299	0	0
rxn14346	0	0
rxn90002	-11,74915982	1000
rxn90003	0	0
rxn90004	0	0
rxn90005	-0,028845363	-0,028334856
rxn08173	0	499,9997819
Biomass_Bacteria	1,142074	1,142074006
t_Cl	0,005153038	0,005153038
t_Sulfate	0,004294198	0,004294198
t_Cu2+	0,003435359	0,003435359
t_Mg	0,008587254	0,008587254
t_Ca2+	0,005153038	0,005153038
t_NH3	0	0
t_H2O	-22,66065134	7,357021328
t_Biomass	-1,142074006	-1,142074
t_Butyrate	-4,897085859	0
t_D-Lactate	-12,6946296	0
t_Ethanol	0	0
t_Formate	-13,09792468	0
t_H2	0	0,5
t_L-Lactate	-12,6946296	0
t_Nitrite	0	0
t_Phosphate	1,517653029	2,011966651
t_Propionate	-0,547712428	0
t_O2	0	0
t_D-Glucose	0	0,5
t_CO2	-12,90129322	0
t_Acetate	-19,21490579	-0,032185929
t_Succinate	-6,450646608	0
t_(S,S)-2,3-Butanediol	0	0
t_H2S	-0,726433304	0
Ex_Cl	-0,005153038	-0,005153038
Ex_Sulfate	-0,004294198	-0,004294198
Ex_Cu2+	-0,003435359	-0,003435359

Ex_Mg	-0,008587254	-0,008587254
Ex_Ca2+	-0,005153038	-0,005153038
Ex_NH3	0	0
Ex_H2O	-7,357021328	22,66065134
Ex_Biomass	1,142074	1,142074006
Ex_Butyrate	0	4,897085859
Ex_D-Lactate	0	12,6946296
Ex_Ethanol	0	0
Ex_Formate	0	13,09792468
Ex_H2	-0,5	0
Ex_L-Lactate	0	12,6946296
Ex_Nitrite	0	0
Ex_Phosphate	-2,011966651	-1,517653029
Ex_Propionate	0	0,547712428
Ex_O2	0	0
Ex_D-Glucose	-0,5	0
Ex_CO2	0	12,90129322
Ex_Acetate	0,032185929	19,21490579
Ex_Succinate	0	6,450646608
Ex_(S,S)-2,3-Butanediol	0	0
Ex_H2S	0	0,726433304
t_Fe2	0,007983097	0,007983097
t_fe3	0,007728415	0,007728415
t_Acetaldehyde	0	0
t_Adenosine	0	0,494313614
t_AMP	0	0,494313614
t_Amylotriose	0	0
t_BIOT	0	0
t_Choline	0	0
t_Cytidine	0	0
t_Cytosine	0	0
t_DAlanine	0	0
t_Deoxyadenosine	0	0,057583371
t_Deoxycytidine	0	0,057583371
t_Deoxyguanosine	0	0
t_Deoxyinosine	0	0
t_Deoxyuridine	0	0
t_DRibose	0	0,5
t_DSerine	0	0
t_Glycerol	0	0
t_GSH	0	0
t_Guanine	0	0
t_H2S2O3	0	0
t_Heme	0,000254683	0,000254683
t_Homocysteine	0	0
t_HYXN	0	0,494313614
t_Inosine	0	0,494313614
t_LACT	0	0,5
t_LAlanine	-0,978394266	0,5
t_LArabinose	0	0

t_LArginine	-0,127717002	0,324062643
t_LAsparagine	-0,201578348	0,5
t_LAspartate	-0,201578348	0,5
t_LCysteine	-0,226433304	0,5
t_LGlutamate	-0,978394266	0,5
t_LGlutamine	-0,239197133	0,5
t_LHistidine	0,105185015	0,105185016
t_LInositol	0	0
t_LIsoleucine	-0,225304938	0,322407492
t_LLeucine	0,499999997	0,5
t_LLysine	-0,320810876	0,380767474
t_LMethionine	-0,047712428	0,5
t_LPhenylalanine	-1,272820946	0,205573321
t_LThreonine	0,281521241	0,5
t_LTryptophan	-0,676120386	0,063076747
t_LTyrosine	-1,325101668	0,153292599
t_LValine	-1,007973986	0,470420283
t_Maltose	0	0,5
t_Niacin	0,002602787	0,002602787
t_Ornithine	0	0
t_PPi	0	0
t_Pyridoxol	0	0
t_XAN	0	0
t_5Deoxyadenosine	0	0
t_Acetoacetate	-6,347314798	0
t_BET	0	0
t_Calomide	0	0
t_Cbl	0	0
t_Citrate	0	0
t_CysGly	0	0
t_Dulcose	0	0
t_Glycine	-0,978394266	0,5
t_Glycolaldehyde	0	0
t_LProline	0,245317495	0,245317497
t_Maltohexaose	0	0
t_Methanol	0	0
t_NAcetylDglucosamine	0	0
t_PM	0	0
t_Putrescine	0	0
t_Pyridoxal	0,000254683	0,000254683
t_Riboflavin	0,000509365	0,000509365
t_Sorbitol	0	0
t_Spermidine	0	0
t_Sucrose	0	0,5
t_Taurine	0	0
t_Thiamin	0	0
t_Thyminose	0	0
t_Uracil	0	0,365829146
t_Uridine	0	0,365829146
t_Mn2+	0,003435359	0,003435359

t_Formaldehyde	-0,903686627	0
t_Fumarate	-6,450646608	0
t_Oxidized glutathione	0	0
t_Adenine	0	0
t_Nicotinamide	0	0
t_4-Hydroxybenzoate	0	0
t_Co2+	0,003435359	0,003435359
t_D-Glutamate	0	0
t_Chorismate	0	0
t_Folate	0,00101873	0,00101873
t_N-Acetyl-D-mannosamine	0	0
t_Siroheme	0	0
t_Selenate	0	0
t_Menaquinone 7	0	0
t_2-Demethylmenaquinone 8	0	0
t_Menaquinone 8	0	0
t_Ubiquinone-8	0	0
t_2-Oxobutyrate	0	0
t_3MOP	0	0
t_Neu5Ac	0	0
t_Glycerol-3-phosphate	0	0
t_H+	-1000	0,5
t_indol	0	0
t_Nicotinamide ribonucleotide	0	0
t_PAN	0,000657835	0,000657835
t_Pyridoxal phosphate	0	0
t_Zn2+	0,003435359	0,003435359
t_1,2-Diacyl-sn-glycerol dioctadecanoyl	0	0
t_meso-2,6-Diaminopimelate	0	0
t_L-Serine	-0,978394266	0,5
t_D-Fructose	0	0,5
t_D-Mannose	0	0
t_beta D-Galactose	0	0,5
t_L-Fucose	0	0
Ex_Fe2	-0,007983097	-0,007983097
Ex_fe3	-0,007728415	-0,007728415
Ex_Acetaldehyde	0	0
Ex_Adenosine	-0,494313614	0
Ex_AMP	-0,494313614	0
Ex_Amylotriose	0	0
Ex_BIOT	0	0
Ex_Choline	0	0
Ex_Cytidine	0	0
Ex_Cytosine	0	0
Ex_DAlanine	0	0
Ex_Deoxyadenosine	-0,057583371	0
Ex_Deoxycytidine	-0,057583371	0
Ex_Deoxyguanosine	0	0
Ex_Deoxyinosine	0	0
Ex_Deoxyuridine	0	0

Ex_DRibose	-0,5	0
Ex_DSerine	0	0
Ex_Glycerol	0	0
Ex_GSH	0	0
Ex_Guanine	0	0
Ex_Heme	-0,000254683	-0,000254683
Ex_Homocysteine	0	0
Ex_HYXN	-0,494313614	0
Ex_Inosine	-0,494313614	0
Ex_LACT	-0,5	0
Ex_LAlanine	-0,5	0,978394266
Ex_LArabinose	0	0
Ex_LArginine	-0,324062643	0,127717002
Ex_LAsparagine	-0,5	0,201578348
Ex_LAspartate	-0,5	0,201578348
Ex_LCysteine	-0,5	0,226433304
Ex_LGlutamate	-0,5	0,978394266
Ex_LGlutamine	-0,5	0,239197133
Ex_LHistidine	-0,105185016	-0,105185015
Ex_LInositol	0	0
Ex_LIsoleucine	-0,322407492	0,225304938
Ex_LLeucine	-0,5	-0,499999997
Ex_LLysine	-0,380767474	0,320810876
Ex_LMethionine	-0,5	0,047712428
Ex_LPhenylalanine	-0,205573321	1,272820946
Ex_LThreonine	-0,5	-0,281521241
Ex_LTryptophan	-0,063076747	0,676120386
Ex_LTyrosine	-0,153292599	1,325101668
Ex_LValine	-0,470420283	1,007973986
Ex_Maltose	-0,5	0
Ex_Niacin	-0,002602787	-0,002602787
Ex_Ornithine	0	0
Ex_PPi	0	0
Ex_XAN	0	0
Ex_5Deoxyadenosine	0	0
Ex_Acetoacetate	0	6,347314798
Ex_BET	0	0
Ex_Calomide	0	0
Ex_Cbl	0	0
Ex_Citrate	0	0
Ex_CysGly	0	0
Ex_Dulcose	0	0
Ex_Glycine	-0,5	0,978394266
Ex_Glycolaldehyde	0	0
Ex_LProline	-0,245317497	-0,245317495
Ex_Maltohexaose	0	0
Ex_Methanol	0	0
Ex_NAcetylDglucosamine	0	0
Ex_PM	0	0
Ex_Putrescine	0	0

Ex_Pyridoxal	-0,000254683	-0,000254683
Ex_Riboflavin	-0,000509365	-0,000509365
Ex_Sorbitol	0	0
Ex_Spermidine	0	0
Ex_Sucrose	-0,5	0
Ex_Taurine	0	0
Ex_Thiamin	0	0
Ex_Thyminose	0	0
Ex_Uracil	-0,365829146	0
Ex_Uridine	-0,365829146	0
Ex_Mn2+	-0,003435359	-0,003435359
Ex_Formaldehyde	0	0,903686627
Ex_Fumarate	0	6,450646608
Ex_Oxidized glutathione	0	0
Ex_Adenine	0	0
Ex_Nicotinamide	0	0
Ex_4-Hydroxybenzoate	0	0
Ex_Co2+	-0,003435359	-0,003435359
Ex_D-Glutamate	0	0
Ex_Folate	-0,00101873	-0,00101873
Ex_N-Acetyl-D-mannosamine	0	0
Ex_Siroheme	0	0
Ex_Selenate	0	0
Ex_Menaquinone 7	0	0
Ex_2-Demethylmenaquinone 8	0	0
Ex_Menaquinone 8	0	0
Ex_Ubiquinone-8	0	0
Ex_Neu5Ac	0	0
Ex_H+	-0,5	1000
Ex_indol	0	0
Ex_Nicotinamide ribonucleotide	0	0
Ex_PAN	-0,000657835	-0,000657835
Ex_Zn2+	-0,003435359	-0,003435359
Ex_1,2-Diacyl-sn-glycerol dioctadecanoyl	0	0
Ex_L-Serine	-0,5	0,978394266
Ex_D-Fructose	-0,5	0
Ex_D-Mannose	0	0
Ex_beta D-Galactose	-0,5	0
Ex_L-Fucose	0	0
t_Arabinan	0	0
t_Starch	0	0,005
t_octanoate	0	0
t_Melibiose	0	0,5
t_Linolenate	0	0
t_Amylose	0	0
t_Linoleate	0	0
Ex_Arabinan	0	0
Ex_Starch	-0,005	0
Ex_Melibiose	-0,5	0
Ex_Linolenate	0	0

Ex_Amylose	0	0
Ex_Linoleate	0	0
t_Raffinose_Melitose	0	0
t_Isovaleric_acid	0	0
t_H2O2	0	0
t_Nitric_oxide	0	0
Ex_Raffinose_Melitose	0	0
Ex_Isovaleric_acid	0	0
Ex_H2O2	0	0
Ex_Nitric_oxide	0	0
rxn01207_1	0	0
rxn08972	0	0
rxn08973	0	0
rxn06111	0	999,9995637
rxn13726	0	0
rxn13727	0	0
rxn13729	0	0
rxn08974	0	0
rxn10122	0	0
rxn10123	0	0
rxn10124	0	0
rxn12665	0	0
rxn06097	0	0,005
t_Sulfite	0	0
Ex_Sulfite	0	0

rxn ID	minFlux	max Flux
rxn00001	0	1000
rxn00003	-1,101402489	0
rxn00011	-1,101402489	0
rxn00016	0	0
rxn00020	0	1000
rxn00022	0	1000
rxn00029	0,00101873	0,00101873
rxn00031	0	0
rxn00044	0	0
rxn00060	0,000254683	0,000254683
rxn00062	0	1000
rxn00065	0	1000
rxn00067	0	0
rxn00076	0	1000
rxn00077	0	0,000510507
rxn00083	-1000	1000
rxn00085	-1000	0
rxn00097	-1000	1000
rxn00100	0,000657835	0,000657835
rxn00102	-1000	999,9999646
rxn00104	-1000	0
rxn00105	-999,9973972	1000
rxn00106	-1000	0
rxn00109	0	0
rxn00114	-1000	0,443869351
rxn00119	0,368989262	1000
rxn00122	0,000254683	0,000254683
rxn00124	0	0,000254683
rxn00126	0,000764048	0,000764048
rxn00132	0	1000
rxn00133	0	0
rxn00134	0	1000
rxn00137	0	0
rxn00138	0	1000
rxn00139	-999,9971425	0
rxn00140	0	1000
rxn00141	-999,9994906	0
rxn00142	0	0
rxn00143	0,000509365	1000
rxn00148	-1000	0
rxn00152	0	170,44825
rxn00154	0	512,3327877
rxn00157	-512,3327877	0
rxn00159	-1000	1000
rxn00161	-1000	1000
rxn00162	0	1000
rxn00165	0	500,1555099
rxn00171	0	0
rxn00173	-748,3285603	999,9957058

rxn00174	0	1000
rxn00175	0	1000
rxn00176	0	0
rxn00178	-495,4229739	0
rxn00179	0	0
rxn00184	-1000	0
rxn00187	0	1000
rxn00189	0	1000
rxn00190	0	1000
rxn00191	-1000	1000
rxn00192	0	0
rxn00193	0,031494975	0,031494975
rxn00196	0	0
rxn00198	0	1000
rxn00199	0	1000
rxn00200	0	0
rxn00206	0,008588396	203,0028857
rxn00208	0	0
rxn00209	0	0,000254683
rxn00211	0	0
rxn00213	-1000	999,5995158
rxn00214	-1,5	0
rxn00216	0	1000
rxn00222	0	1000
rxn00223	-1000	1000
rxn00224	0,000254683	1000
rxn00225	-1000	0
rxn00226	0	0
rxn00231	0	0
rxn00239	0,238807673	1000
rxn00248	-1000	1000
rxn00250	-1000	999,9999646
rxn00251	-1000	0
rxn00254	0	0
rxn00256	-981,7377305	0
rxn00260	-1000	0,693911605
rxn00278	-1000	0
rxn00283	0,027731841	0,027731841
rxn00284	-1000	0
rxn00285	-1000	1000
rxn00286	0	0
rxn00290	-1000	1000
rxn00293	-999,9370101	1000
rxn00295	-1000	999,9370101
rxn00299	0	0
rxn00300	0	0
rxn00301	0	999,7611923
rxn00302	0	0
rxn00303	0	0
rxn00304	-1000	0

rxn00305	0	1000
rxn00307	0	0
rxn00313	0	0,550701244
rxn00324	0	0
rxn00328	0	0
rxn00337	0,039197122	12,79645159
rxn00340	0	1000
rxn00342	0	1000
rxn00347	0	1000
rxn00350	-0,000254683	-0,000254683
rxn00358	0	0
rxn00360	0	1000
rxn00361	0	1000
rxn00363	0	1000
rxn00364	-999,6310107	999,999309
rxn00365	0	1000
rxn00368	0	1000
rxn00369	0	1000
rxn00371	0	1000
rxn00374	0	0
rxn00383	0,004294198	0,004294198
rxn00388	0	0
rxn00391	0	999,9997453
rxn00392	0,000254683	1000
rxn00395	0	1000
rxn00407	0	1000
rxn00410	-999,8225103	1000
rxn00411	-1000	0
rxn00412	0	1000
rxn00414	0	1000
rxn00416	0	1000
rxn00420	0	0
rxn00423	0	500,1555099
rxn00426	0	0
rxn00430	0	0
rxn00436	0	1000
rxn00437	0	0
rxn00438	-999,9997453	1000
rxn00440	0	1000
rxn00453	0	0
rxn00459	-487,1917115	11,69113256
rxn00460	-1000	0
rxn00461	0,031494975	0,031494975
rxn00470	0,046135221	496,0138607
rxn00471	0	1,101402489
rxn00474	0	0
rxn00490	0	0
rxn00493	-1,101402489	0
rxn00499	0	0
rxn00500	-12,57102997	0

rxn00506	0	0
rxn00512	0	0
rxn00514	0	0
rxn00517	-1000	0
rxn00519	0	1000
rxn00527	-1,101402489	0
rxn00533	-999,9999646	1000
rxn00536	-1000	1000
rxn00538	0	0
rxn00541	0	0
rxn00543	-1000	1000
rxn00545	0	1000
rxn00547	0	1000
rxn00548	0	748,3242661
rxn00549	0	1000
rxn00550	0	1000
rxn00552	-0,06298995	999,9370101
rxn00554	0	1000
rxn00555	0	1000
rxn00556	0	1000
rxn00557	0	1000
rxn00558	-1000	1000
rxn00559	0	0
rxn00565	0	0
rxn00566	0	1000
rxn00575	0	1000
rxn00577	-1000	0,5
rxn00585	0	0
rxn00602	0	0
rxn00604	-1000	1000
rxn00605	0	1000
rxn00608	0	1000
rxn00609	0	0
rxn00611	-9,633064047	0
rxn00615	0	0
rxn00616	0	9,633064047
rxn00620	0	1000
rxn00621	0	1000
rxn00622	0	0
rxn00634	0	1000
rxn00641	0	0
rxn00645	0,004294198	0,004294198
rxn00646	0	0
rxn00647	0	0
rxn00649	0	500,1555099
rxn00650	-0,000254683	-0,000254683
rxn00653	0	0
rxn00668	0	0
rxn00670	0	1000
rxn00673	0	0

rxn00674	0	1000
rxn00675	0	0
rxn00684	0	0
rxn00685	0	999,9989813
rxn00686	0	0
rxn00687	0	999,9989813
rxn00689	0	0
rxn00690	0	1000
rxn00692	-0,260507082	499,8950029
rxn00693	0	0,393414802
rxn00695	-1000	1000
rxn00698	-1000	0
rxn00701	0	1000
rxn00704	-1000	2
rxn00707	0	1000
rxn00708	0	1000
rxn00709	0	1000
rxn00710	0	0,365829146
rxn00711	-999,9971425	0
rxn00712	0	1000
rxn00713	0	1000
rxn00714	0	0
rxn00715	0	1000
rxn00717	0	999,6310107
rxn00726	0	0
rxn00727	0	0
rxn00729	0	0
rxn00735	0	0
rxn00737	0	12,7530956
rxn00740	0	12,57119976
rxn00741	0	0
rxn00743	0	1000
rxn00744	0	1000
rxn00745	0	1000
rxn00747	-5,669905969	493,2129381
rxn00756	0	0
rxn00758	0	0
rxn00763	0	0
rxn00765	0	0
rxn00770	0,002857469	1000
rxn00772	0	1000
rxn00775	0	0
rxn00777	-334,2393075	249,6445726
rxn00778	-1000	1000
rxn00780	0	0
rxn00781	-487,1917115	11,69113256
rxn00785	-499,2341647	249,0901014
rxn00786	-1000	5,669905969
rxn00789	0	0
rxn00790	-0,000254683	-0,000254683

rxn00791	0	0
rxn00792	0	0
rxn00796	0	0
rxn00799	-1000	9,413019079
rxn00800	-0,26805619	613,1051769
rxn00801	0	0
rxn00802	0	1000
rxn00806	0	0
rxn00808	0	1,5
rxn00809	-507,4857432	0
rxn00811	-507,4857432	0
rxn00816	0	0,5
rxn00817	0	0,5
rxn00818	0	0
rxn00819	0	0
rxn00829	0,000690955	0,000690955
rxn00830	6,28141E-05	6,28141E-05
rxn00831	0	999,9971425
rxn00832	0	0
rxn00834	0,268056189	0,26805619
rxn00836	-999,9971425	0
rxn00838	-0,26805619	613,1051769
rxn00851	0	1000
rxn00853	0	0
rxn00855	0	0
rxn00856	0,008138419	495,9758639
rxn00858	0	0
rxn00865	0	0
rxn00869	0	0
rxn00872	-9,428272476	0
rxn00874	0	0
rxn00875	0	1000
rxn00879	0	0
rxn00881	0	0
rxn00882	0	0
rxn00883	0	0
rxn00889	0	0
rxn00890	0	0
rxn00892	0	0
rxn00898	0	1,101402489
rxn00902	0	0
rxn00903	-1000	1000
rxn00904	-1000	1000
rxn00907	-999,9994906	0,000509365
rxn00908	-500,6263448	0,079866382
rxn00909	-9,435974623	0,393669484
rxn00910	-9,633064047	0
rxn00913	0	1000
rxn00915	-999,9707515	0
rxn00916	-999,7319438	0,26805619

rxn00917	0	1000
rxn00918	0	0
rxn00925	0	0
rxn00926	0	613,3732331
rxn00927	-1000	999,9966332
rxn00929	-1000	1000
rxn00931	-1000	1000
rxn00938	0	999,9971425
rxn00942	0	999,9971425
rxn00943	0	1000
rxn00946	0	0
rxn00947	0	1000
rxn00950	-1000	500,351708
rxn00952	0	1000
rxn00953	0	500,1555099
rxn00955	0,000509365	1000
rxn00972	0	0
rxn00973	-1000	1000
rxn00974	-1000	1000
rxn00977	0	0
rxn00980	0	0
rxn00983	0	0
rxn00985	-1000	0
rxn00986	0	0
rxn00990	-1000	1000
rxn00991	-0,000690955	-0,000690955
rxn00994	-1000	9,428272476
rxn01000	0	1,101402489
rxn01004	0	0
rxn01007	0	0
rxn01008	0	0
rxn01011	0	0
rxn01013	0	0
rxn01016	0	0
rxn01018	0	0,365829146
rxn01019	0	0,275350622
rxn01021	0	0
rxn01025	0	0
rxn01029	0	0
rxn01041	-1000	1000
rxn01042	-1000	1000
rxn01044	0	0
rxn01049	0	1000
rxn01069	0	12,62620109
rxn01073	0	0
rxn01078	0	0
rxn01089	0	0
rxn01097	0	0
rxn01098	0	0
rxn01100	-1000	0

rxn01101	0	0
rxn01106	-11,69113256	487,1917115
rxn01114	0	0,5
rxn01115	0	1000
rxn01116	-0,739307528	750,1445726
rxn01117	0	0
rxn01119	0	0
rxn01123	0	0
rxn01124	0	0
rxn01133	0	0
rxn01134	-1000	0
rxn01139	0	0
rxn01146	0	0
rxn01152	0	0,5
rxn01153	0	0
rxn01169	0	1000
rxn01171	-1000	1000
rxn01182	0	0
rxn01187	0	748,3242661
rxn01199	0	0
rxn01200	0	1000
rxn01201	-495,9684165	-0,000690955
rxn01202	0	0
rxn01203	0	0
rxn01204	0,000690955	495,9684165
rxn01210	0	0
rxn01211	-999,9997453	0,000509365
rxn01213	6,28141E-05	6,28141E-05
rxn01226	0,029248515	1000
rxn01228	0	0
rxn01231	0,004294198	0,004294198
rxn01233	0,004294198	0,004294198
rxn01237	0	0
rxn01241	0	0
rxn01242	0	0
rxn01249	-202,9942973	0
rxn01251	0	0
rxn01252	0	202,9942973
rxn01255	0,000254683	1,101657171
rxn01256	0	1,101402489
rxn01257	0	0
rxn01259	0	0
rxn01261	0	0
rxn01265	-999,9997453	-0,002602787
rxn01268	0	1,101402489
rxn01274	0	0
rxn01275	0	0
rxn01276	0	0
rxn01278	0	0
rxn01286	0	0

rxn01290	0	0
rxn01291	0	0
rxn01292	0	0
rxn01299	0	999,9971425
rxn01300	0	12,62620109
rxn01301	-12,75725447	0
rxn01302	-12,75725447	0
rxn01303	0	12,70208334
rxn01304	0	12,70208334
rxn01305	0	0
rxn01308	0	0
rxn01310	-1000	0
rxn01321	0	0
rxn01322	0	0
rxn01329	0	0
rxn01332	0,000254683	1,101657171
rxn01333	-1000	334,0328483
rxn01334	0	1000
rxn01343	0	1000
rxn01346	0	1000
rxn01347	0	1000
rxn01348	0	1000
rxn01351	0	1000
rxn01352	-1000	-0,029248515
rxn01354	-1000	0
rxn01355	0	0
rxn01360	0	202,9942973
rxn01361	-202,9942973	0
rxn01362	-0,365829146	0
rxn01365	0	0
rxn01366	-999,968505	1000
rxn01367	0	0
rxn01368	0	999,6310107
rxn01370	0	1000
rxn01374	0	0
rxn01377	0	0
rxn01380	0	0
rxn01385	0	0
rxn01387	-1000	0
rxn01388	-1000	1000
rxn01390	0	0
rxn01395	0	0
rxn01396	0	0,000254683
rxn01406	0	0
rxn01423	0	0
rxn01427	0	0
rxn01434	0	1000
rxn01437	0	0
rxn01439	0	0
rxn01441	0	0

rxn01445	0	999,9707515
rxn01446	-0,029248515	-0,029248515
rxn01452	-999,999309	0
rxn01459	0	495,9681618
rxn01464	0	0
rxn01465	-0,365829146	0
rxn01466	6,28141E-05	6,28141E-05
rxn01476	0	1000
rxn01484	0	0
rxn01485	-0,06298995	-0,062989949
rxn01486	0	0
rxn01492	0	0
rxn01500	-0,000690955	-0,000690955
rxn01509	-999,9707515	1000
rxn01510	0	1000
rxn01513	0,028334856	0,028334856
rxn01517	0	0
rxn01518	0,028334856	1000
rxn01519	0	0
rxn01521	0	999,9716651
rxn01537	0	999,9997453
rxn01539	-1000	0
rxn01541	-1000	999,9707515
rxn01544	-999,9971425	0
rxn01545	0	999,9971425
rxn01549	0	0
rxn01563	0	1000
rxn01571	0	0
rxn01575	-1,101402489	0
rxn01590	0	0
rxn01601	0	0
rxn01602	0	0
rxn01603	0	0
rxn01610	0	0
rxn01613	0	0
rxn01615	0	0
rxn01621	0	0
rxn01626	0	0
rxn01629	-0,00203746	-0,00203746
rxn01636	0,046135221	496,3462456
rxn01637	-496,3462456	-0,046135221
rxn01643	-12,79645159	-0,039197122
rxn01644	0,031494975	0,582196219
rxn01648	-999,9707515	1000
rxn01650	-1000	0
rxn01653	0	0
rxn01667	0	0
rxn01669	0	999,9973972
rxn01670	0	1000
rxn01671	0	1000

rxn01675	0	0
rxn01679	0	0
rxn01682	0	0
rxn01683	0	0
rxn01685	0	0
rxn01686	0	0
rxn01704	0	0
rxn01706	0	0
rxn01731	0	0
rxn01735	0	0
rxn01737	0	0
rxn01739	0,000254683	1,101657171
rxn01740	-1,101657171	-0,000254683
rxn01741	0	0
rxn01748	0	0
rxn01757	0	0
rxn01758	-0,004294198	-0,004294198
rxn01763	0	0
rxn01775	0	0
rxn01790	0	0
rxn01799	-0,028334856	0,029248515
rxn01800	0	0,057583371
rxn01802	0	0
rxn01807	0	0
rxn01816	-0,393160119	1000
rxn01831	0	0
rxn01834	0	0
rxn01835	0	0
rxn01842	0	0
rxn01843	0	0
rxn01851	0	495,9681618
rxn01857	0	0
rxn01860	0	0
rxn01870	0	0
rxn01879	0	0
rxn01885	0	0
rxn01896	0	0
rxn01906	0	0
rxn01917	0,046135221	496,3462456
rxn01937	0	0
rxn01946	0	0
rxn01961	0	999,9971425
rxn01962	0	0
rxn01964	0	0
rxn01967	0	0
rxn01971	0	1000
rxn01972	0,031494975	1000
rxn01973	-999,968505	0
rxn01974	0,031494975	0,582196219
rxn01975	-1000	1000

rxn01977	-1000	1000
rxn01982	0	0
rxn01986	-0,057583371	0
rxn01987	-0,057583371	0
rxn01991	0	0
rxn01997	0	0
rxn01998	0	0
rxn01999	0	0
rxn02000	0	0
rxn02003	0	0
rxn02004	0	1000
rxn02008	0,031494975	0,031494975
rxn02011	0,031494975	0,031494975
rxn02012	0	0
rxn02015	0	0
rxn02020	0	0
rxn02023	0	0
rxn02033	0	0
rxn02046	0	0
rxn02056	0	999,9997453
rxn02061	0	0
rxn02085	0	0
rxn02090	0	0
rxn02093	0	0
rxn02106	0	0
rxn02112	0	0
rxn02122	0	0
rxn02128	0	0
rxn02138	0	0
rxn02139	0	0
rxn02144	0	0
rxn02154	0	999,9973972
rxn02155	0,002602787	1000
rxn02160	0	0
rxn02161	0	0
rxn02166	0	0
rxn02167	0	999,999309
rxn02171	0,000690955	495,9684165
rxn02175	0,000657835	0,000657835
rxn02185	-1,101402489	1,101402489
rxn02186	0	1,101402489
rxn02187	0	0
rxn02190	0	0
rxn02195	0	0
rxn02199	0	0
rxn02200	0	0
rxn02201	0	0
rxn02209	0	0
rxn02212	0,000254683	1,101657171
rxn02213	0,000254683	1,101657171

rxn02219	0	0
rxn02222	0	0
rxn02228	0	0
rxn02258	0	0
rxn02263	0	0
rxn02264	0,000254683	0,000254683
rxn02275	0	0
rxn02284	-0,031494975	0
rxn02285	-0,031494975	0
rxn02286	0,031494975	0,031494975
rxn02287	-999,9997453	1000
rxn02288	0	0
rxn02302	-1000	12,70182866
rxn02305	0,000254683	1000
rxn02314	0	1000
rxn02315	0	1000
rxn02316	0	1000
rxn02317	-1000	0
rxn02318	0	0
rxn02319	0	0
rxn02320	0	0
rxn02321	0	0
rxn02322	0,000690955	0,000690955
rxn02339	0	0
rxn02341	0,000657835	0,000657835
rxn02342	0	0
rxn02350	0	0
rxn02351	0	0
rxn02353	0	0
rxn02356	-1000	1000
rxn02358	-1000	1000
rxn02373	-1000	1000
rxn02375	0	0
rxn02380	-1000	1000
rxn02397	0	0
rxn02400	0	0
rxn02405	0	0
rxn02409	0	0
rxn02418	0	0
rxn02429	0	0
rxn02449	0	0
rxn02454	0	0
rxn02461	0	0
rxn02465	-496,3462456	-0,046135221
rxn02473	0	0
rxn02474	0	0
rxn02475	0	0
rxn02476	0,000254683	1,101657171
rxn02480	0	0
rxn02483	0	0

rxn02484	0	999,9997453
rxn02495	0	0
rxn02503	0	0
rxn02504	0	0
rxn02508	0	0
rxn02518	0	0
rxn02521	0	0
rxn02522	0	0
rxn02525	0	0
rxn02569	0	0
rxn02571	0	0
rxn02581	0	0
rxn02596	0	0
rxn02597	0	0
rxn02632	0	0
rxn02638	0	0
rxn02640	0	0
rxn02650	0	0
rxn02663	0	0
rxn02679	0	0
rxn02720	0	0
rxn02729	0	0
rxn02749	0	0
rxn02751	0	0
rxn02760	0	0
rxn02762	0	0
rxn02774	-999,9997453	0
rxn02776	0	0
rxn02789	0	0
rxn02795	0	1000
rxn02796	-1000	0
rxn02798	0	0
rxn02803	0	0
rxn02811	0	0
rxn02821	0	0
rxn02822	0	0
rxn02831	0	0
rxn02834	0	0
rxn02835	0	0
rxn02849	0	0
rxn02853	0	0
rxn02866	0	0
rxn02875	0	0
rxn02884	0	0
rxn02885	0	0
rxn02889	0	0
rxn02895	0,000254683	0,000254683
rxn02897	0	0
rxn02898	0	0
rxn02900	0	0

rxn02914	0	0
rxn02922	0	0
rxn02928	-1000	999,968505
rxn02929	-1000	999,968505
rxn02931	0	0
rxn02936	0	0
rxn02937	0,000254683	0,000254683
rxn02939	0	0
rxn02988	0	0
rxn02990	0	0
rxn03004	0	0,000254683
rxn03005	-0,000254683	0
rxn03008	0	0
rxn03030	0,031494975	1000
rxn03031	-999,968505	0
rxn03036	0	0
rxn03039	0	0
rxn03041	0	0
rxn03044	0	0
rxn03047	0	0
rxn03052	0	0
rxn03062	0	0
rxn03066	0	0
rxn03068	0	0
rxn03075	0	999,9997453
rxn03084	0,000254683	0,000254683
rxn03086	-1000	-0,031494975
rxn03087	0	999,968505
rxn03094	0	0
rxn03095	0	0
rxn03102	0	0
rxn03106	0	0
rxn03108	0,000254683	1000
rxn03114	0	0
rxn03132	0	0
rxn03135	0	0
rxn03136	0	0
rxn03137	0	0
rxn03140	0	0
rxn03141	0	0
rxn03146	0	0
rxn03147	0	0
rxn03164	0,031494975	0,031494975
rxn03167	0	0
rxn03173	0	0
rxn03174	0	0
rxn03175	0	0
rxn03181	0	0
rxn03182	0	0
rxn03194	0	1,101402489

rxn03251	0	0
rxn03253	0	0
rxn03264	0	0
rxn03269	0	0
rxn03273	0	0
rxn03282	0	0
rxn03292	0	0
rxn03293	0	0
rxn03295	0	0
rxn03296	0	0
rxn03298	0	0
rxn03301	0	0
rxn03304	0	0
rxn03313	0	0
rxn03316	0	0
rxn03333	0	0
rxn03354	0	0
rxn03362	0	0
rxn03371	0	0
rxn03372	0	0
rxn03378	0	0
rxn03379	0	0
rxn03380	0	0
rxn03382	0	0
rxn03383	0	0
rxn03384	0	0
rxn03387	0	0
rxn03396	0	0
rxn03402	0	0
rxn03405	0	0
rxn03406	0	0
rxn03407	0	0
rxn03408	0,031494975	0,031494975
rxn03409	0	0
rxn03419	0	0
rxn03421	0	0
rxn03423	0	0
rxn03435	-1,101402489	0
rxn03436	0	1,101402489
rxn03437	0	1,101402489
rxn03439	0	0
rxn03445	0	0
rxn03446	0	0
rxn03462	0	0
rxn03465	0	0
rxn03467	0	0
rxn03468	0	0
rxn03481	0	0
rxn03482	0	0
rxn03483	0	0

rxn03491	0	0
rxn03492	0	0
rxn03511	0	0
rxn03536	0	0
rxn03537	0	0
rxn03538	0	0
rxn03546	0	0
rxn03548	0	1000
rxn03549	0	0
rxn03552	0	0
rxn03553	0	0
rxn03596	0	0
rxn03598	0	0
rxn03599	0	0
rxn03608	0	0
rxn03638	0,062989949	0,06298995
rxn03641	0,000690955	495,9684165
rxn03642	0,000690955	495,9684165
rxn03663	0	0
rxn03668	0	0
rxn03669	0	0
rxn03670	0	0
rxn03671	0	0
rxn03674	0	0
rxn03838	0	0
rxn03839	0	0
rxn03861	0	0
rxn03869	0	0
rxn03870	0	0
rxn03884	0	0
rxn03887	0	0
rxn03891	0	0
rxn03901	0,031494975	0,031494975
rxn03902	0	0
rxn03903	0	0
rxn03904	0,031494975	0,031494975
rxn03907	0	0
rxn03908	0	0
rxn03909	0	0
rxn03910	0	0
rxn03916	0	0
rxn03917	0	0
rxn03918	0	0
rxn03923	0,004294198	0,004294198
rxn03933	0	0
rxn03951	0	1000
rxn03958	0	0
rxn03963	-1000	0
rxn03964	0	1000
rxn03974	-0,028334856	0

rxn03975	-0,028334856	0
rxn04016	0	0
rxn04045	0	0
rxn04050	0	0
rxn04068	0	0
rxn04096	0	0
rxn04113	0	0
rxn04142	0	0
rxn04234	0	0
rxn04308	0	0
rxn04413	0	0
rxn04417	0	0
rxn04418	0	0
rxn04437	0	0
rxn04443	0	0
rxn04673	0	0
rxn04676	-249,0898467	1000
rxn04678	-1000	249,0898467
rxn04703	0	0
rxn04704	0	0
rxn04726	0	0
rxn04736	0	0
rxn04750	0	0
rxn04786	0,007702147	0,007702147
rxn04794	0	1000
rxn04822	0	0
rxn04941	0	0
rxn04942	0	0
rxn04943	0	0
rxn04954	-9,633064047	0
rxn04960	0	0
rxn05005	-1000	0
rxn05006	-1000	0
rxn05010	0	0
rxn05011	0	0
rxn05024	0	0
rxn05030	6,28141E-05	6,28141E-05
rxn05039	0	0
rxn05040	0	0
rxn05044	0	0
rxn05050	0	0
rxn05072	0	0
rxn05085	0	0
rxn05108	0	0
rxn05114	0	0
rxn05115	0	0
rxn05116	0	1000
rxn05122	0	0
rxn05233	0	0
rxn05234	0	0

rxn05236	0	0
rxn05247	0	0
rxn05248	0	0
rxn05249	0	0
rxn05250	0	0
rxn05251	0	0
rxn05252	0	0
rxn05269	0	0
rxn05289	0	0
rxn05322	0	0
rxn05323	0	0
rxn05324	0	0
rxn05325	0	0
rxn05326	0	0
rxn05327	0	0
rxn05328	0	0
rxn05329	0	0
rxn05330	0	0
rxn05331	0	0
rxn05332	0	0
rxn05333	0	0
rxn05334	0	0
rxn05335	0	0
rxn05336	0	0
rxn05337	0	0
rxn05338	0	0
rxn05339	0	0
rxn05340	0	0
rxn05341	0	0
rxn05342	0	0
rxn05343	0	0
rxn05344	0	0
rxn05345	0	0
rxn05346	0	0
rxn05347	0	0
rxn05348	0	0
rxn05349	0	0
rxn05350	0	0
rxn05457	-1000	0
rxn05465	0	0
rxn05733	0	0
rxn05736	0	1000
rxn05740	-1000	1000
rxn05762	0,004294198	0,004294198
rxn05778	0	0
rxn05779	0	0
rxn05794	-1000	0
rxn05833	0	0
rxn05853	0	0
rxn05856	0	0

rxn05871	0	0
rxn05872	0	0
rxn05873	0	0
rxn05874	0	0
rxn05899	0	0
rxn05901	0	0
rxn05909	0	500,1555099
rxn05918	0	0
rxn05927	0	0
rxn05934	0	0
rxn05937	-1000	1000
rxn05938	-512,3327877	0
rxn05939	-550,0166899	1000
rxn05940	-1000	1000
rxn05957	0	1000
rxn05958	0	0
rxn05962	0	0
rxn05990	0	0
rxn06005	0	0
rxn06023	0	0
rxn06033	0	0
rxn06043	0	0
rxn06045	0	0
rxn06071	0,017176793	406,0057714
rxn06075	0	0
rxn06078	0	0
rxn06081	0	0
rxn06096	0	0
rxn06108	-1000	0
rxn06109	-9,232300287	1000
rxn06139	0	0
rxn06140	0	0
rxn06155	0	0
rxn06181	0	1000
rxn06182	0	1000
rxn06190	0	0
rxn06195	0	0
rxn06196	0	0
rxn06200	0	0
rxn06201	0	0
rxn06217	0	0
rxn06218	0	0
rxn06219	0	0
rxn06224	0	0
rxn06251	0	0
rxn06252	-1000	0
rxn06253	0	1000
rxn06280	0	0
rxn06285	0	0
rxn06298	0	0

rxn06300	0	0
rxn06316	0	0
rxn06328	0	0
rxn06347	0	0
rxn06348	0	0
rxn06362	0	0
rxn06365	0	0
rxn06368	0	0
rxn06377	0	0
rxn06381	0	0
rxn06390	0	0
rxn06394	0	0
rxn06400	0	0
rxn06403	0	0
rxn06432	0	0
rxn06434	0	0
rxn06435	0	0
rxn06438	0	0
rxn06439	0	0
rxn06440	0	0
rxn06441	0	0
rxn06443	0	0
rxn06444	0	0
rxn06445	0	0
rxn06446	0	0
rxn06447	0	0
rxn06448	0	0
rxn06449	0	0
rxn06453	0	0
rxn06485	0	0
rxn06489	0	0
rxn06493	0	0
rxn06500	0	0
rxn06522	0	0
rxn06538	0	0
rxn06556	0	0
rxn06565	0	0
rxn06584	0	0
rxn06591	0,00203746	0,00203746
rxn06595	0	0
rxn06624	0	0
rxn06641	0	0
rxn06648	0	0
rxn06664	0	0
rxn06671	0	0
rxn06672	0	1000
rxn06673	0	1000
rxn06680	0	0
rxn06691	0	0
rxn06694	0	0

rxn06701	0	0
rxn06723	0	0
rxn06726	0	0
rxn06729	0	0
rxn06733	0	0
rxn06737	0	0
rxn06741	0	0
rxn06751	0	0
rxn06752	0	0
rxn06760	0	0
rxn06768	0	0
rxn06798	0	0
rxn06799	0	0
rxn06820	0	0
rxn06823	0	0
rxn06836	0	0
rxn06837	0	0
rxn06838	0	0
rxn06850	0	0
rxn06864	0	0
rxn06882	0	0
rxn06889	0	1000
rxn06890	0	0
rxn06936	0	0
rxn06937	0,00203746	0,00203746
rxn06947	0	0
rxn06958	-406,0057714	-0,017176793
rxn07056	0	0
rxn07059	0	0
rxn07099	0	0
rxn07172	0	0
rxn07199	0	0
rxn07241	0	0
rxn07292	0	0
rxn07414	0	0
rxn07415	0	0
rxn07437	0	0
rxn07438	0	0
rxn07441	0	999,968505
rxn07452	0	0
rxn07456	0	1000
rxn07465	0,007702147	0,007702147
rxn07466	-1000	1000
rxn07476	0	0
rxn07484	0	0
rxn07485	0	0
rxn07486	0	0
rxn07573	0	0
rxn07577	0	0
rxn07578	0	0

rxn07579	0	0
rxn07584	0	0
rxn07585	0	0
rxn07603	0	0
rxn07623	0	0
rxn07645	0	0
rxn07679	0	0
rxn07683	0	0
rxn07687	0	0
rxn07804	0	0
rxn07807	0	0
rxn07832	0	0
rxn07846	0	0
rxn07849	0	0
rxn07987	0	0
rxn07989	0	0
rxn07991	0	0
rxn07992	0	0
rxn07993	0	0
rxn07994	0	0
rxn08025	0	0
rxn08040	0	0
rxn08043	0	1,101402489
rxn08044	0	0
rxn08067	-1000	1000
rxn08083	0	0
rxn08084	0	0
rxn08085	0	0
rxn08086	0	0
rxn08087	0	0
rxn08088	0	0
rxn08089	0	0
rxn08094	0	1000
rxn08126	0	0
rxn08127	0	0
rxn08128	0	0
rxn08129	0	0
rxn08131	0,000254683	0,000254683
rxn08133	0	0
rxn08171	0	0
rxn08180	0	0
rxn08294	0	0
rxn08295	0	0
rxn08296	0	0
rxn08297	0	0
rxn08298	0	0
rxn08299	0	0
rxn08300	0	0
rxn08306	0	0
rxn08307	0	0

rxn08308	0	0
rxn08309	0	0
rxn08310	0	0
rxn08311	0	0
rxn08312	0	0
rxn08352	0	0
rxn08413	0	0
rxn08433	0	0
rxn08434	0	1000
rxn08435	0	0
rxn08436	0	0
rxn08437	0	0
rxn08438	0	0
rxn08444	0	1000
rxn08448	0	0
rxn08449	0	0
rxn08451	0	0
rxn08453	0	0
rxn08454	0	1000
rxn08455	0	0
rxn08456	0	1000
rxn08457	0	0
rxn08519	0,057583371	0,057583371
rxn08546	0	0
rxn08547	0	1000
rxn08548	0	0
rxn08549	0	0
rxn08550	0	0
rxn08551	0	0
rxn08552	0	0
rxn08582	0	0,5
rxn08605	0	0
rxn08607	0	0
rxn08615	-1000	1000
rxn08647	0	0
rxn08668	0	0
rxn08669	0	0
rxn08713	0	0
rxn08733	-1000	0
rxn08764	0	1,101402489
rxn08796	0	0
rxn08797	0	1000
rxn08798	0	0
rxn08799	0	1000
rxn08800	0	0
rxn08801	0	1000
rxn08802	0	0
rxn08803	0	0
rxn08804	0	0
rxn08805	0	0

rxn08806	0	0
rxn08807	0	0
rxn08808	0	0
rxn08809	0	0
rxn08810	0	0
rxn08811	0	0
rxn08812	0	0
rxn08813	0	0
rxn08814	0	0
rxn08815	0	0
rxn08816	0	0
rxn08817	0	0
rxn08818	0	0
rxn08819	0	0
rxn08820	0	0
rxn08821	0	0
rxn08822	0	0
rxn08823	0	0
rxn08838	0	0
rxn08839	0	0
rxn08840	0	0
rxn08841	0	0
rxn08842	0	0
rxn08843	0	0
rxn08844	0	0
rxn08845	0	0
rxn08846	0	0
rxn08847	0	0
rxn08848	0	0
rxn08849	0	0
rxn08850	0	0
rxn08851	0	0
rxn08857	0	0
rxn08889	0,000768616	0,000768616
rxn08890	0,006222019	0,006222019
rxn08891	0,000768616	0,000768616
rxn08892	-999,9854054	1000
rxn08893	-999,9923184	999,993087
rxn08894	-999,9854054	1000
rxn08897	-0,006912974	-0,006912974
rxn08926	0,000690955	0,000690955
rxn08927	-999,9984639	999,9869415
rxn08928	-999,9854054	1000
rxn08929	0,00153609	0,00153609
rxn08930	0	0
rxn08958	0,000768616	0,000768616
rxn09010	0	0
rxn09016	0	999,7611923
rxn09108	0	0
rxn09109	0	0

rxn09110	0	0
rxn09111	0	0
rxn09112	0	0
rxn09113	0	0
rxn09114	0	0
rxn09176	-1000	1000
rxn09177	0	0,000657835
rxn09235	0,028334856	0,028334856
rxn09237	0,029248515	0,029248515
rxn09264	0	0
rxn09265	0	0
rxn09340	0	0
rxn09341	0	999,6310107
rxn09348	0	999,6310107
rxn09355	0	0
rxn09395	0	0
rxn09398	-1000	999,5995158
rxn09399	0	0
rxn09412	-1000	1000
rxn09445	0	0
rxn09446	0	0
rxn09447	0	0
rxn09454	0	0
rxn09455	0	0
rxn09456	0	0
rxn09473	0	0
rxn09486	-999,9370101	1000
rxn09499	0	0
rxn09502	0	1000
rxn09519	0	0
rxn09531	0	0
rxn09557	0,000254683	1000
rxn09616	0,000690955	0,000690955
rxn09631	0,000254683	0,000254683
rxn09632	0	1000
rxn09888	0	0
rxn09889	0	0
rxn09949	0	0
rxn09952	0	0
rxn09978	0	0
rxn09979	0	0
rxn09988	0	0
rxn09992	0	0
rxn09995	0	0
rxn10003	0	0,000657835
rxn10019	0	0
rxn10020	0	0
rxn10021	0	0
rxn10052	-1000	1000
rxn10056	0	0,000510507

rxn10058	0	0,000510507
rxn10060	0	0,000510507
rxn10192	0	0
rxn10193	0	0
rxn10202	0	1000
rxn10203	0	1000
rxn10204	0	1000
rxn10205	0	0
rxn10206	0	0
rxn10207	0	0
rxn10208	0	0
rxn10209	0	0
rxn10210	0	0
rxn10211	0	0
rxn10212	0	0
rxn10213	0	0
rxn10214	0	0
rxn10215	0	0
rxn10216	0	0
rxn10217	0	0
rxn10218	0	0
rxn10219	0	0
rxn10220	0	0
rxn10221	0	0
rxn10222	0	0
rxn10223	0	0
rxn10224	0	0
rxn10225	0	0
rxn10253	0	0
rxn10254	0	0
rxn10255	0	0
rxn10256	0	0
rxn10257	0	0
rxn10258	0	0
rxn10259	0	0
rxn10260	0	0
rxn10261	0	0
rxn10262	0	0
rxn10263	0	0
rxn10264	0	0
rxn10289	0	0
rxn10290	0	0
rxn10291	0	0
rxn10292	0	0
rxn10293	0	0
rxn10294	0	0
rxn10295	0	0
rxn10296	0	0
rxn10297	0	0
rxn10298	0	0

rxn10299	0	0
rxn10300	0	0
rxn10301	0	0
rxn10302	0	0
rxn10303	0	0
rxn10304	0	0
rxn10305	0	0
rxn10306	0	0
rxn10363	0	0
rxn10404	0	0
rxn10405	0	0
rxn10406	0	0
rxn10407	0	0
rxn10408	0	0
rxn10409	0	0
rxn10410	0	0
rxn10785	6,28141E-05	6,28141E-05
rxn10951	0	0,028334856
rxn11007	0,028334856	0,028334856
rxn11513	0	0
rxn11548	0	0
rxn11550	0	0
rxn11551	-1000	1000
rxn11552	-1000	1000
rxn11567	0	0
rxn11571	0	0
rxn11587	0	0
rxn11599	0	0
rxn11601	0	0
rxn11609	0	0
rxn11641	0	0
rxn11663	-1000	0
rxn11676	0	0
rxn11677	0	0
rxn11678	0	0
rxn11702	0	0
rxn11710	0	0
rxn11711	0	0
rxn11712	0	0
rxn11713	0	0
rxn11714	0	0
rxn11715	0	0
rxn11716	0	0
rxn11717	0	0
rxn11718	0	0
rxn11728	0	0
rxn11732	0	0
rxn11749	0	0
rxn11755	0	0
rxn11757	-999,9971425	0

rxn11759	0	999,9971425
rxn11760	-999,9971425	0
rxn11765	0	0
rxn11766	0	0
rxn11768	0	0
rxn11772	0	0
rxn11773	0	0
rxn11788	0	0
rxn11879	0	0
rxn11934	0	0
rxn11965	0	0
rxn11984	0	0
rxn12013	0	0
rxn12049	0	0
rxn12053	0	0
rxn12054	0	0
rxn12147	0	0
rxn12218	-1000	-0,000254683
rxn12221	0,000254683	1000
rxn12510	0,000657835	0,000657835
rxn12649	-999,9989813	0
rxn12778	0	0
rxn12844	0	0
rxn12845	0	0
rxn12846	0	0
rxn12847	0	0
rxn13420	0,000690955	1000
rxn13421	0,000690955	1000
rxn13705	0	0
rxn13741	0	0
rxn13906	-0,007702147	-0,007702147
rxn13936	0,015363179	0,01536318
rxn13963	-0,008588397	-0,008588396
rxn13974	-512,3327877	0
rxn13994	0	0
rxn13996	0	0
rxn14043	0	0
rxn14048	-1000	0
rxn14050	0	0
rxn14054	-1000	0
rxn14063	0	0
rxn14070	0	0
rxn14089	-1000	0
rxn14120	-1000	-0,00101873
rxn14132	0	0
rxn14136	0	0
rxn14178	-1000	1000
rxn14193	0	1000
rxn14219	0	0
rxn14228	-1000	0

rxn14235	0	0
rxn14250	0	0
rxn14275	0	0
rxn14279	0	0
rxn14328	0	0
rxn14346	0	0
rxn90002	-11,69113256	1000
rxn90003	0	0
rxn90004	0	0
rxn90005	-0,028845363	-0,028334856
rxn08173	0	500
Biomass_Bacteria	1,142074	1,142074006
t_Cl	0,005153038	0,005153038
t_Sulfate	0	0
t_Cu2+	0,003435359	0,003435359
t_Mg	0,008587254	0,008587254
t_Ca2+	0,005153038	0,005153038
t_NH3	0	0
t_H2O	-27,82375517	10
t_Biomass	-1,142074006	-1,142074
t_Butyrate	-9,428272476	0
t_D-Lactate	-12,57102997	0
t_Ethanol	0	0
t_Formate	-25,21583678	0
t_L-Lactate	0	0
t_Nitrite	0	0
t_Phosphate	1,517653029	2,011966651
t_Propionate	-12,75326539	0
t_O2	0	0
t_D-Glucose	0	0,5
t_CO2	-25,21583678	0
t_Acetate	-18,85654495	0
t_Succinate	-9,428272476	0
t_(S,S)-2,3-Butanediol	0	0
t_BDOH	0	0
t_H2S	0	0
Ex_Cl	-0,005153038	-0,005153038
Ex_Sulfate	0	0
Ex_Cu2+	-0,003435359	-0,003435359
Ex_Mg	-0,008587254	-0,008587254
Ex_Ca2+	-0,005153038	-0,005153038
Ex_NH3	0	0
Ex_H2O	-10	27,82375517
Ex_Biomass	1,142074	1,142074006
Ex_Butyrate	0	9,428272476
Ex_D-Lactate	0	12,57102997
Ex_Ethanol	0	0
Ex_Formate	0	25,21583678
Ex_L-Lactate	0	0
Ex_Nitrite	0	0

Ex_Phosphate	-2,011966651	-1,517653029
Ex_Propionate	0	12,75326539
Ex_O2	0	0
Ex_D-Glucose	-0,5	0
Ex_CO2	0	25,21583678
Ex_Acetate	0	18,85654495
Ex_Succinate	0	9,428272476
Ex_(S,S)-2,3-Butanediol	0	0
Ex_BDOH	0	0
Ex_H2S	0	0
t_Fe2	0,007983097	0,007983097
t_fe3	0,007728415	0,007728415
t_Acetaldehyde	0	0
t_Adenosine	0	0,494313614
t_Allantoin	0	0
t_AMP	0	0,494313614
t_Amylotriose	0	0
t_BIOT	0	0
t_Choline	0	0
t_Cytidine	0	0
t_Cytosine	0	0
t_DAlanine	0	0
t_Deoxycytidine	0	0,057583371
t_Deoxyguanosine	0	0
t_Deoxyinosine	0	0
t_Deoxyuridine	0	0
t_DRibose	0	0,5
t_DSerine	0	0
t_GLUM	0	0
t_Glycerol	0	0
t_GSH	0	0
t_Guanine	0	0
t_H2S2O3	0	0
t_Heme	0,000254683	0,000254683
t_Homocysteine	0	0
t_HYXN	0	0,494313614
t_Inosine	0	0,494313614
t_LACT	0	0,5
t_LAlanine	-0,601402489	0,5
t_LArabinose	0	0
t_LArginine	0,052881445	0,328232069
t_LAsparagine	-0,050701244	0,5
t_LAspartate	-0,601402489	0,5
t_LCysteine	0,106585198	0,5
t_LGlutamate	-0,601402489	0,5
t_LGlutamine	-0,050701244	0,5
t_LHistidine	0,105185015	0,105185016
t_LInositol	0	0
t_LIsoleucine	-0,778994999	0,322407492
t_LLeucine	0,499999997	0,5

t_LLysine	-0,169933773	0,380767474
t_LMethionine	-0,222139106	0,171275697
t_LPhenylalanine	-0,895829169	0,205573321
t_LThreonine	-0,601402489	0,5
t_LTryptophan	0,063076747	0,063076747
t_LTyrosine	-0,948364573	0,153037917
t_LValine	-0,630982208	0,470420283
t_Maltose	0	0,5
t_Niacin	0,002602787	0,002602787
t_Ornithine	0	0
t_PPi	0	0
t_Pyridoxol	0	0
t_XAN	0	0
t_5Deoxyadenosine	0	0
t_Acetoacetate	-9,428272476	0
t_BET	0	0
t_Calomide	0	0
t_Carnosine	0	0
t_Citrate	0	0
t_CysGly	0	0
t_Dulcose	0	0
t_Glycine	-0,601402489	0,5
t_Glycolaldehyde	0	0
t_LProline	-0,856084994	0,245317497
t_Maltohexaose	0	0
t_Methanol	0	0
t_NAcetylDglucosamine	0	0
t_PM	0	0
t_Putrescine	0	0
t_Pyridoxal	0,000254683	0,000254683
t_Riboflavin	0,000509365	0,000509365
t_Sorbitol	0	0
t_Spermidine	0	0
t_Sucrose	0	0,5
t_Taurine	0	0
t_Thiamin	0	0
t_Thymidine	0	0
t_Thyminose	0	0,057583371
t_TRHL	0	0
t_Uracil	0	0,365829146
t_Uridine	0	0,365829146
t_Mn2+	0,003435359	0,003435359
t_Formaldehyde	0	0
t_Fumarate	-9,428272476	0
t_Oxidized glutathione	0	0
t_Adenine	0	0
t_Nicotinamide	0	0
t_Co2+	0,003435359	0,003435359
t_D-Arabinose	0	0,5
t_D-Glutamate	0	0

t_Chorismate	0	0
t_Folate	0,00101873	0,00101873
t_N-Acetyl-D-mannosamine	0	0
t_Siroheme	0	0
t_Menaquinone 7	0	0
t_2-Demethylmenaquinone 8	0	0
t_Menaquinone 8	0	0
t_Ubiquinone-8	0	0
t_2-Oxobutyrate	0	0
t_3MOP	0	0
t_ABEE	0	0
t_Neu5Ac	0	0
t_Glycerol-3-phosphate	0	0
t_H+	-1000	0,5
t_indol	0	0
t_Nicotinamide ribonucleotide	0	0
t_PAN	0,000657835	0,000657835
t_Pyridoxal phosphate	0	0
t_Zn2+	0,003435359	0,003435359
t_1,2-Diacyl-sn-glycerol dioctadecanoyl	0	0
t_meso-2,6-Diaminopimelate	0	0
t_L-Serine	-0,601402489	0,5
t_D-Fructose	0	0
t_D-Mannose	0	0
t_Oxalate	0	0
t_Arachidonate	0	0
t_D-Mannitol	0	0
t_beta D-Galactose	0	0,5
t_L-Fucose	0	0
Ex_Fe2	-0,007983097	-0,007983097
Ex_fe3	-0,007728415	-0,007728415
Ex_Acetaldehyde	0	0
Ex_Adenosine	-0,494313614	0
Ex_Allantoin	0	0
Ex_AMP	-0,494313614	0
Ex_Amylotriose	0	0
Ex_BIOT	0	0
Ex_Choline	0	0
Ex_Cytidine	0	0
Ex_Cytosine	0	0
Ex_DAlanine	0	0
Ex_Deoxycytidine	-0,057583371	0
Ex_Deoxyguanosine	0	0
Ex_Deoxyinosine	0	0
Ex_Deoxyuridine	0	0
Ex_DRibose	-0,5	0
Ex_DSerine	0	0
Ex_GLUM	0	0
Ex_Glycerol	0	0
Ex_GSH	0	0

Ex_Guanine	0	0
Ex_Heme	-0,000254683	-0,000254683
Ex_Homocysteine	0	0
Ex_HYXN	-0,494313614	0
Ex_Inosine	-0,494313614	0
Ex_LACT	-0,5	0
Ex_LAlanine	-0,5	0,601402489
Ex_LArabinose	0	0
Ex_LArginine	-0,328232069	-0,052881445
Ex_LAsparagine	-0,5	0,050701244
Ex_LAspartate	-0,5	0,601402489
Ex_LCysteine	-0,5	-0,106585198
Ex_LGlutamate	-0,5	0,601402489
Ex_LGlutamine	-0,5	0,050701244
Ex_LHistidine	-0,105185016	-0,105185015
Ex_LInositol	0	0
Ex_LIsoleucine	-0,322407492	0,778994999
Ex_LLeucine	-0,5	-0,499999997
Ex_LLysine	-0,380767474	0,169933773
Ex_LMethionine	-0,171275697	0,222139106
Ex_LPhenylalanine	-0,205573321	0,895829169
Ex_LThreonine	-0,5	0,601402489
Ex_LTryptophan	-0,063076747	-0,063076747
Ex_LTyrosine	-0,153037917	0,948364573
Ex_LValine	-0,470420283	0,630982208
Ex_Maltose	-0,5	0
Ex_Niacin	-0,002602787	-0,002602787
Ex_Ornithine	0	0
Ex_PPi	0	0
Ex_XAN	0	0
Ex_5Deoxyadenosine	0	0
Ex_Acetoacetate	0	9,428272476
Ex_BET	0	0
Ex_Calomide	0	0
Ex_Carnosine	0	0
Ex_Citrate	0	0
Ex_CysGly	0	0
Ex_Dulcose	0	0
Ex_Glycine	-0,5	0,601402489
Ex_Glycolaldehyde	0	0
Ex_LProline	-0,245317497	0,856084994
Ex_Maltohexaose	0	0
Ex_Methanol	0	0
Ex_NAcetylDglucosamine	0	0
Ex_PM	0	0
Ex_Putrescine	0	0
Ex_Pyridoxal	-0,000254683	-0,000254683
Ex_Riboflavin	-0,000509365	-0,000509365
Ex_Sorbitol	0	0
Ex_Spermidine	0	0

Ex_Sucrose	-0,5	0
Ex_Taurine	0	0
Ex_Thiamin	0	0
Ex_Thymidine	0	0
Ex_Thyminose	-0,057583371	0
Ex_TRHL	0	0
Ex_Uracil	-0,365829146	0
Ex_Uridine	-0,365829146	0
Ex_Mn2+	-0,003435359	-0,003435359
Ex_Formaldehyde	0	0
Ex_Fumarate	0	9,428272476
Ex_Oxidized glutathione	0	0
Ex_Adenine	0	0
Ex_Nicotinamide	0	0
Ex_Co2+	-0,003435359	-0,003435359
Ex_D-Arabinose	-0,5	0
Ex_D-Glutamate	0	0
Ex_Folate	-0,00101873	-0,00101873
Ex_N-Acetyl-D-mannosamine	0	0
Ex_Siroheme	0	0
Ex_Menaquinone 7	0	0
Ex_2-Demethylmenaquinone 8	0	0
Ex_Menaquinone 8	0	0
Ex_Ubiquinone-8	0	0
Ex_ABEE	0	0
Ex_Neu5Ac	0	0
Ex_H+	-0,5	1000
Ex_indol	0	0
Ex_Nicotinamide ribonucleotide	0	0
Ex_PAN	-0,000657835	-0,000657835
Ex_Zn2+	-0,003435359	-0,003435359
Ex_1,2-Diacyl-sn-glycerol dioctadecanoyl	0	0
Ex_L-Serine	-0,5	0,601402489
Ex_D-Fructose	0	0
Ex_D-Mannose	0	0
Ex_Oxalate	0	0
Ex_Arachidonate	0	0
Ex_D-Mannitol	0	0
Ex_beta D-Galactose	-0,5	0
Ex_L-Fucose	0	0
t_Arabinan	0	0
t_Starch	0	0,005
t_octanoate	0	0
t_Melibiose	0	0,5
t_Linolenate	0	0
t_Amylose	0	0
t_Linoleate	0	0
Ex_Arabinan	0	0
Ex_Starch	-0,005	0
Ex_Melibiose	-0,5	0

Ex_Linolenate	0	0
Ex_Amylose	0	0
Ex_Linoleate	0	0
t_Raffinose_Melitose	0	0
t_Isovaleric_acid	0	0
t_H2O2	0	0
Ex_Raffinose_Melitose	0	0
Ex_Isovaleric_acid	0	0
Ex_H2O2	0	0
rxn01207_1	0	0
rxn08972	0	0
rxn08973	0	0
rxn06111	0	1000
rxn13726	0	0
rxn13727	0	0
rxn13729	0	0
rxn08974	0	0
rxn10122	0	0
rxn10123	0	0
rxn10124	0	0
rxn12665	0	0
rxn06097	0	0,005
t_Sulfite	0	0
Ex_Sulfite	0	0

rxn ID	minFlux	max Flux
rxn00001	0	1000
rxn00003	-1,835607611	0
rxn00004	0	0
rxn00011	-1,835607611	0
rxn00016	0	0
rxn00020	0	0
rxn00022	0	0,505
rxn00029	0,00101873	0,00101873
rxn00031	0	0
rxn00060	0,000254683	0,000254683
rxn00062	0	1000
rxn00065	0	0
rxn00066	0,000254683	0,918058488
rxn00076	0	1000
rxn00077	0	0,000510507
rxn00085	-1000	0
rxn00096	-1000	0
rxn00097	-1000	1000
rxn00100	0,000657835	0,000657835
rxn00102	-1000	0,701578348
rxn00103	0	1000
rxn00104	-1000	0
rxn00105	-999,9973972	1000
rxn00106	-1000	0
rxn00109	0	0
rxn00114	-1000	0,463135
rxn00119	0,368989262	1000
rxn00122	0,000254683	0,000254683
rxn00124	0,000254683	0,000254683
rxn00126	0,008466195	0,008466195
rxn00127	0,007702147	0,007702147
rxn00132	0	999,988931
rxn00133	0	1000
rxn00137	0	0
rxn00138	0	1000
rxn00139	-999,9971425	0
rxn00142	0	0
rxn00143	0,000509365	0,000509365
rxn00144	0	0
rxn00148	-1000	0
rxn00151	-1000	0
rxn00157	-11,83953443	0
rxn00159	-1000	1000
rxn00161	-1000	1000
rxn00162	0	1000
rxn00165	0	0,080375747
rxn00171	0	1,302559388
rxn00172	-1000	1000
rxn00173	0	1000

rxn00175	0	1000
rxn00176	0	0
rxn00178	-12,17095574	0
rxn00179	0	0
rxn00182	-1000	0
rxn00184	-1000	0
rxn00187	0	1000
rxn00189	0	1000
rxn00190	0	1000
rxn00191	-1000	1000
rxn00192	0	1000
rxn00193	0,031494975	0,031494975
rxn00194	0,000690955	12,1716467
rxn00198	0	1000
rxn00199	0	1000
rxn00211	0	0
rxn00213	-1000	999,5995158
rxn00214	-1,5	0
rxn00216	0	1000
rxn00222	0	0
rxn00224	0,000254683	1000
rxn00225	-1000	0
rxn00226	0	0
rxn00227	0	1000
rxn00231	0	0
rxn00239	0,238807673	1000
rxn00242	0	0
rxn00245	0	0
rxn00247	0	1000
rxn00250	-1000	1000
rxn00255	0	0
rxn00256	-11,83953443	0
rxn00258	-1000	1000
rxn00259	-11,83953443	0
rxn00260	-505,9899319	0,701613752
rxn00262	0	0,917803805
rxn00272	-1000	1000
rxn00273	0	0
rxn00274	0	0
rxn00275	-1000	1000
rxn00283	0,027731841	0,027731841
rxn00293	0,062989949	999,6940007
rxn00297	0	0
rxn00298	0	0
rxn00299	0	0
rxn00300	0	0
rxn00301	0	999,7611923
rxn00302	0	0
rxn00303	0	0
rxn00304	-1000	0

rxn00307	0	0
rxn00313	0	0,917803805
rxn00322	0	0
rxn00328	0	0
rxn00333	0,000254683	0,918058488
rxn00337	0,031494975	0,94929878
rxn00338	0	0,002602787
rxn00340	0	1000
rxn00342	0	1000
rxn00346	0	0
rxn00350	-0,000254683	-0,000254683
rxn00358	0	0
rxn00360	0	1000
rxn00361	0	1000
rxn00363	0	1000
rxn00364	-999,6310107	0,000657835
rxn00365	0	1000
rxn00368	0	1000
rxn00369	0	1000
rxn00371	0	1000
rxn00374	0	0
rxn00379	0	1000
rxn00391	0	999,9997453
rxn00392	0,000254683	1000
rxn00405	0	0
rxn00407	0	1000
rxn00410	-999,8225103	1000
rxn00411	-1000	0
rxn00412	0	1000
rxn00414	0	1000
rxn00416	0	1000
rxn00420	0	0
rxn00422	-1000	1000
rxn00423	0	0,080375747
rxn00424	-1000	1000
rxn00426	0	0
rxn00433	0	0
rxn00436	0	999,9997453
rxn00437	0	0
rxn00440	0,000254683	1000
rxn00453	0	999,8497316
rxn00456	0	999,8497316
rxn00459	-0,718652365	11,12088206
rxn00460	-1000	0
rxn00461	0,031494975	0,031494975
rxn00462	0	0
rxn00469	0	1000
rxn00470	0,045698949	0,045698949
rxn00473	0	0,080375747
rxn00474	0	0,080375747

rxn00490	0	0
rxn00493	-1,835607611	0
rxn00499	-11,83953443	0
rxn00505	0	1000
rxn00510	0	0
rxn00512	-0,917803805	0
rxn00514	0	0
rxn00517	-1000	0
rxn00527	-1,835607611	0
rxn00533	-1000	1000
rxn00536	-1000	1000
rxn00541	-0,080375747	0
rxn00543	-1000	1000
rxn00545	0	1000
rxn00547	0	1000
rxn00549	0	1000
rxn00551	0	1000
rxn00552	-0,06298995	999,9370101
rxn00554	0	1000
rxn00555	0	1000
rxn00556	0	1000
rxn00557	0	1000
rxn00558	-1000	1000
rxn00559	0	0
rxn00560	0	0
rxn00562	0	0
rxn00565	0	0
rxn00566	0	1000
rxn00567	-0,463135	-0,004233097
rxn00575	0	0
rxn00585	0	0
rxn00598	-0,000254683	-0,000254683
rxn00603	0	0
rxn00606	0	0
rxn00607	0	0
rxn00608	0	0
rxn00611	-12,23749811	0
rxn00615	0	0
rxn00616	0	12,23749811
rxn00621	0	0
rxn00622	0	0
rxn00634	0	1000
rxn00647	0	0
rxn00649	0	0,080375747
rxn00650	-0,000254683	-0,000254683
rxn00669	-1000	1000
rxn00670	0	1000
rxn00673	-11,83953443	0
rxn00674	0	1000
rxn00675	0	0

rxn00677	-1000	1000
rxn00679	0	0
rxn00684	0	0
rxn00685	0	999,9989813
rxn00686	0	0
rxn00687	0	999,9989813
rxn00689	0	0
rxn00690	0	0,246931251
rxn00692	-0,260507082	-0,180131335
rxn00693	0,150777749	0,397709
rxn00695	-1000	1000
rxn00701	0	1000
rxn00704	-1000	1,5
rxn00707	0	1000
rxn00708	0	1000
rxn00709	0	1000
rxn00710	0	0
rxn00711	-999,988931	0
rxn00712	0	1000
rxn00713	0	1000
rxn00714	0	0
rxn00715	0	1000
rxn00717	0	0
rxn00726	0	0,080375747
rxn00727	0	0,080375747
rxn00735	0	0
rxn00737	0	0,298854506
rxn00740	0	999,8497316
rxn00741	0	0
rxn00742	-1000	-0,150268384
rxn00744	0	0
rxn00747	-5,164940993	0,70940578
rxn00748	0	0
rxn00758	0	0
rxn00762	0	0
rxn00763	0	0
rxn00764	0	0
rxn00765	0	0
rxn00770	0,011068981	1000
rxn00772	0	1000
rxn00775	0	0
rxn00777	-1,347492013	0,364597609
rxn00778	-1000	1000
rxn00780	0	0
rxn00781	-0,718652365	11,12088206
rxn00784	0	1,302559388
rxn00785	-0,34883813	1,494947862
rxn00786	-1000	5,164940993
rxn00787	0	0
rxn00789	0	0

rxn00790	-0,000254683	-0,000254683
rxn00791	-0,080375747	0
rxn00792	0	0
rxn00796	0	0
rxn00797	-1000	1000
rxn00799	-505,793594	6,634165918
rxn00800	-0,26805619	505,6276287
rxn00802	0	0,458901903
rxn00806	0	2,79999E-09
rxn00808	0	1,5
rxn00809	-504,0530318	0
rxn00811	-504,0530318	0
rxn00816	0	0,5
rxn00817	0	0,5
rxn00818	0	0
rxn00819	0	0
rxn00827	0	0
rxn00829	0,000690955	0,000690955
rxn00830	6,28141E-05	6,28141E-05
rxn00831	0	999,988931
rxn00832	0	0
rxn00834	-999,7290863	1000
rxn00836	-999,9971425	0
rxn00838	-0,26805619	505,6276287
rxn00851	0	1000
rxn00858	0	0
rxn00869	0	0
rxn00872	0	0
rxn00874	0	0
rxn00879	0	0
rxn00881	0	0
rxn00882	0	0
rxn00883	0	0
rxn00889	0	0
rxn00890	0	0
rxn00898	0	1,835607611
rxn00902	0	0
rxn00903	-1000	1000
rxn00904	-1000	1000
rxn00907	-0,246421886	0,000509365
rxn00909	-11,83953443	0,397963683
rxn00910	-12,23749811	0
rxn00913	0	1000
rxn00915	-999,988931	0
rxn00916	-999,7319438	1000
rxn00917	0	1000
rxn00918	0	999,7611923
rxn00926	0	505,8956849
rxn00927	-1000	1000
rxn00929	-1000	1000

rxn00931	-1000	1000
rxn00938	0	999,988931
rxn00942	0	1000
rxn00946	0	0
rxn00947	0	1000
rxn00950	-999,8497316	0,397199635
rxn00952	0	1000
rxn00955	0,000509365	0,000509365
rxn00957	0,000254683	0,000254683
rxn00962	0,000254683	0,000254683
rxn00965	0	0
rxn00972	-11,83953443	0
rxn00973	-1000	1000
rxn00974	-1000	1000
rxn00977	0	0
rxn00979	0,000254683	0,000254683
rxn00980	0	0
rxn00983	0	0
rxn00985	-1000	0
rxn00986	0	0
rxn01000	0	1,835607611
rxn01016	0	0
rxn01018	0	0
rxn01019	0,004233097	0,463135
rxn01020	0	0
rxn01021	0	0
rxn01022	0,007702147	0,007702147
rxn01025	0	0
rxn01034	0	0
rxn01037	0	0
rxn01042	0	0
rxn01043	0	0
rxn01056	-1000	1000
rxn01068	0	0
rxn01069	0	0
rxn01073	0	0
rxn01078	0	0
rxn01080	0	0
rxn01089	0	0
rxn01097	0	0
rxn01098	0	0
rxn01100	-1000	0
rxn01101	0	0
rxn01103	0	1000
rxn01106	-11,12088206	0,718652365
rxn01113	0	0
rxn01114	0	0,5
rxn01116	-1,180825347	0,697930943
rxn01119	0	0
rxn01122	0	0

rxn01123	0	0
rxn01124	0	0
rxn01133	0	0
rxn01137	0	505,8956849
rxn01138	-1000	1000
rxn01139	0	0
rxn01152	0	0,5
rxn01169	0	1000
rxn01171	0	1000
rxn01184	0	1000
rxn01192	0,000254683	0,000254683
rxn01199	0	0
rxn01200	0	1000
rxn01201	-12,1716467	-0,000690955
rxn01202	0	0
rxn01203	0	0
rxn01204	0,000690955	12,1716467
rxn01210	0	0
rxn01211	-0,246676569	0,000509365
rxn01213	6,28141E-05	6,28141E-05
rxn01225	0	999,988931
rxn01226	-999,9707515	1000
rxn01228	0	0
rxn01237	0	0
rxn01248	0	0
rxn01249	0	0
rxn01255	0,000254683	1,835862293
rxn01256	0	1,835607611
rxn01259	0	0
rxn01261	0	0
rxn01265	-999,9915338	0
rxn01268	0	1,835607611
rxn01274	0	0
rxn01275	0	0
rxn01276	0	0
rxn01278	0	0
rxn01280	0	0
rxn01281	0	0
rxn01290	0	0
rxn01291	0	0
rxn01292	0	0
rxn01297	-999,7319438	999,9971425
rxn01299	-1000	1000
rxn01300	0	0
rxn01303	0	0
rxn01304	0	0
rxn01305	0	0
rxn01308	0	0
rxn01310	-1000	0
rxn01321	0	0

rxn01332	0,000254683	1,835862293
rxn01333	-1000	0,839018968
rxn01334	0	1000
rxn01339	0	0
rxn01343	0	1000
rxn01346	0	1000
rxn01347	0	1000
rxn01348	0	1000
rxn01351	0	1000
rxn01352	-1000	-0,029248515
rxn01354	-1000	0
rxn01355	0	0
rxn01358	-1000	1000
rxn01361	0	0
rxn01362	0	0
rxn01366	-1000	1000
rxn01367	0	0
rxn01368	0	999,6310107
rxn01370	0	1000
rxn01374	0	0
rxn01380	0	0
rxn01385	0	0
rxn01387	-1000	0
rxn01388	-1000	1000
rxn01389	0	0
rxn01390	0	0
rxn01396	0	0
rxn01406	0,007702147	0,007702147
rxn01423	0	0
rxn01426	0	0
rxn01434	0	0,458901903
rxn01445	0	999,9707515
rxn01446	-0,029248515	-0,029248515
rxn01452	-999,999309	0
rxn01453	0	0
rxn01454	-0,000690955	-0,000690955
rxn01457	0	0
rxn01465	0	0
rxn01466	6,28141E-05	6,28141E-05
rxn01478	0	0
rxn01480	0	0
rxn01484	0	0
rxn01485	-0,06298995	-0,062989949
rxn01486	0	0
rxn01492	0	0
rxn01500	-0,000690955	-0,000690955
rxn01506	0	0
rxn01509	-999,9707515	1000
rxn01510	0	1000
rxn01513	0,028334856	0,028334856

rxn01518	0,028334856	1000
rxn01519	0	0
rxn01521	0	999,9716651
rxn01522	0	0
rxn01539	-1000	-0,000254683
rxn01544	-999,9971425	0
rxn01545	-1000	1000
rxn01548	-999,9707515	1000
rxn01549	0	0
rxn01575	-1,835607611	0
rxn01587	0	0
rxn01594	0	0
rxn01601	0	0
rxn01602	0	0
rxn01603	0	0
rxn01610	0	0
rxn01615	0	0
rxn01619	0	0
rxn01620	0	0
rxn01621	0	0
rxn01626	0	0
rxn01629	-0,00203746	-0,00203746
rxn01636	-999,950068	0,508833949
rxn01637	-0,508833949	-0,049932046
rxn01641	0	0
rxn01643	-0,94929878	-0,031494975
rxn01644	0,031494975	0,94929878
rxn01646	-1000	999,988931
rxn01647	0	999,988931
rxn01649	-1000	1000
rxn01653	0	0
rxn01654	0	0
rxn01663	0	0
rxn01667	0	0
rxn01669	0	999,9973972
rxn01670	0	999,988931
rxn01675	0	0
rxn01679	0	0
rxn01682	-0,080375747	0
rxn01683	-1000	1000
rxn01684	-1000	1000
rxn01685	0	0
rxn01692	0	0
rxn01693	0	0
rxn01704	0	0
rxn01706	0	0
rxn01710	0	0
rxn01735	0	0
rxn01737	0	0
rxn01739	0,000254683	1,835862293

rxn01740	-1,835862293	-0,000254683
rxn01741	0	0
rxn01757	0	0
rxn01761	0	0
rxn01763	0	0
rxn01772	0	0
rxn01790	0	0
rxn01791	0	0
rxn01799	-0,028334856	0,33749429
rxn01800	0	0,365829146
rxn01807	0	0
rxn01810	0	0
rxn01816	0	1000
rxn01834	0	0
rxn01835	0	0
rxn01842	0	0
rxn01843	0	0
rxn01857	0	0
rxn01858	0	505,8956849
rxn01859	-505,8956849	0,494313614
rxn01860	0	0
rxn01870	0	0
rxn01879	0	0
rxn01885	0	0
rxn01892	0	0
rxn01906	0	0
rxn01914	0	0
rxn01917	0,049932046	0,508833949
rxn01946	0	0
rxn01953	0	0
rxn01961	0	999,988931
rxn01962	0	0
rxn01964	0	0,080375747
rxn01966	0	1000
rxn01967	0	1000
rxn01972	0,031494975	1000
rxn01973	-6,366364412	0
rxn01974	0,031494975	0,94929878
rxn01977	-1000	1000
rxn01982	0	0
rxn01985	0	505,8956849
rxn01986	-0,057583371	0,802559388
rxn01987	-0,5	0
rxn01989	0	0
rxn01990	0	0
rxn01991	0	0
rxn01997	0	0
rxn01998	0	0
rxn01999	0	0
rxn02000	0	0

rxn02003	0	0
rxn02008	0,031494975	0,031494975
rxn02011	0,031494975	0,031494975
rxn02012	0	0
rxn02015	0	0
rxn02020	0	0
rxn02023	0	0
rxn02040	0	0
rxn02046	0	0
rxn02056	0	999,9997453
rxn02061	0	0
rxn02085	0	0
rxn02089	0	0
rxn02090	0	0
rxn02093	0	0
rxn02123	0	11,83953443
rxn02128	0	0
rxn02138	0	0
rxn02139	0	0
rxn02143	0,000254683	0,000254683
rxn02144	0,000254683	0,000254683
rxn02154	0	999,9973972
rxn02155	0,002602787	1000
rxn02160	0	0
rxn02161	0	0
rxn02167	0	999,999309
rxn02171	0,000690955	12,1716467
rxn02173	0	0
rxn02175	0,000657835	0,000657835
rxn02185	-1,835607611	1,835607611
rxn02186	0	1,835607611
rxn02187	0	0
rxn02190	0	0
rxn02195	0	0
rxn02199	0	0
rxn02200	0	0
rxn02201	0	0
rxn02209	0	0
rxn02212	0,000254683	1,835862293
rxn02213	0,000254683	1,835862293
rxn02222	0	0
rxn02228	0	0
rxn02262	0	0
rxn02263	0	0
rxn02264	0,000254683	0,000254683
rxn02272	0	0
rxn02275	0	0
rxn02277	0	0
rxn02283	0	0
rxn02284	-0,031494975	0

rxn02285	-0,031494975	0
rxn02286	0,031494975	0,031494975
rxn02287	-999,9997453	1000
rxn02288	0	0
rxn02297	0	0
rxn02302	-1000	0
rxn02305	0,000254683	0,000254683
rxn02312	0	0
rxn02314	0	1000
rxn02315	0	1000
rxn02316	0	1000
rxn02317	-1000	0
rxn02318	0	0
rxn02319	0	0
rxn02320	0	0
rxn02321	0	0
rxn02322	0,000690955	0,000690955
rxn02339	0	0
rxn02341	0,000657835	0,000657835
rxn02346	0	0
rxn02351	0	0
rxn02356	-1000	1000
rxn02358	-1000	1000
rxn02369	-0,000254683	-0,000254683
rxn02373	-1000	1000
rxn02375	0	0
rxn02380	-1000	1000
rxn02386	0	0
rxn02400	0	999,988931
rxn02402	-0,002602787	0
rxn02409	0	0
rxn02454	0	0
rxn02465	-0,508833949	-0,049932046
rxn02466	0	0
rxn02473	0	0
rxn02474	0	0
rxn02476	0,000254683	1,835862293
rxn02483	0,000254683	0,000254683
rxn02484	0,000254683	0,000254683
rxn02495	0	0
rxn02503	0	0
rxn02504	0	0
rxn02507	0	0,080375747
rxn02508	0	0,080375747
rxn02518	0	0
rxn02521	0	0
rxn02522	0	0
rxn02569	0	0
rxn02571	0	0
rxn02581	0	0

rxn02596	0	0
rxn02597	0	0
rxn02650	0	0
rxn02663	0	0
rxn02679	0	0
rxn02720	0	0
rxn02749	0	0
rxn02751	0	0
rxn02760	0	0
rxn02762	0	0
rxn02774	-999,9997453	0
rxn02775	0	0
rxn02776	0	0
rxn02789	0	0
rxn02795	0	0
rxn02796	0	0
rxn02803	0	0
rxn02811	0	0
rxn02821	0	0
rxn02822	0	0
rxn02831	0	0
rxn02834	0	0
rxn02835	0	0
rxn02866	0	0
rxn02875	0	0
rxn02895	0,000254683	0,000254683
rxn02897	0	0
rxn02900	0	0
rxn02906	0	0
rxn02914	0	0
rxn02922	0	0
rxn02928	-1000	999,968505
rxn02929	-1000	999,968505
rxn02931	0	0
rxn02936	0	0
rxn02937	0,000254683	0,000254683
rxn02943	0	0
rxn02986	0	0
rxn02988	-0,002602787	0
rxn02990	0	0
rxn03004	0	0,000254683
rxn03005	-0,000254683	0
rxn03030	0,031494975	1000
rxn03031	-6,366364412	0
rxn03034	0	0
rxn03047	0	0
rxn03052	0	0
rxn03057	0,007702147	0,007702147
rxn03062	0	0
rxn03066	0	0

rxn03068	0	0
rxn03075	0,000254683	0,000254683
rxn03084	0,000254683	0,000254683
rxn03086	-1000	-0,031494975
rxn03087	0	6,366364412
rxn03094	0	0
rxn03095	0	0
rxn03102	0	0
rxn03108	0,000254683	0,000254683
rxn03123	0	0
rxn03132	0	0
rxn03135	0	0
rxn03136	0	0
rxn03137	0	0
rxn03140	0	0
rxn03141	0	0
rxn03147	0	0
rxn03150	0	0
rxn03158	0	0
rxn03164	0,031494975	0,031494975
rxn03174	0	0
rxn03175	0	0
rxn03194	0	1,835607611
rxn03199	0	0
rxn03200	-1000	0
rxn03201	0	1000
rxn03251	0	0
rxn03253	0	0
rxn03263	0	0
rxn03264	0	0
rxn03269	0	0
rxn03273	0	0
rxn03282	0	0
rxn03319	0	0
rxn03333	0	0
rxn03371	0,021165486	2,315675
rxn03372	0	0
rxn03373	0	0
rxn03374	0	0
rxn03379	0	0
rxn03382	0	0
rxn03383	0	0
rxn03384	0	0
rxn03387	0	0
rxn03395	0	0
rxn03397	0	0
rxn03402	0	0
rxn03405	0	0
rxn03406	0	0
rxn03407	0	0

rxn03408	0,031494975	0,031494975
rxn03409	0	0
rxn03419	0	0
rxn03421	0	0
rxn03435	-1,835607611	0
rxn03436	0	1,835607611
rxn03437	0	1,835607611
rxn03445	0	0
rxn03446	0	0
rxn03462	0	0
rxn03465	0	0
rxn03467	0	0
rxn03481	0	0
rxn03482	0	0
rxn03483	0	0
rxn03489	0	0
rxn03491	0	0
rxn03492	0	0
rxn03512	0	0
rxn03513	0	0
rxn03514	0	0
rxn03535	0	0
rxn03536	0	0
rxn03537	0	0
rxn03538	0	0
rxn03540	0	0
rxn03546	0	0
rxn03547	0	0
rxn03548	0	1000
rxn03549	0	0
rxn03596	0	0
rxn03599	0	0
rxn03634	0	0
rxn03638	0,062989949	0,06298995
rxn03641	0,000690955	12,1716467
rxn03642	0,000690955	12,1716467
rxn03643	0	0
rxn03644	0	0
rxn03668	0	0
rxn03669	0	0
rxn03670	0	0
rxn03671	0	0
rxn03798	-1000	1000
rxn03838	0	0
rxn03839	0	0
rxn03845	0	0
rxn03852	0	0
rxn03856	0	0
rxn03861	0	0
rxn03864	0	0

rxn03869	0	0
rxn03884	0	0
rxn03885	0	0
rxn03887	0	0
rxn03891	6,28141E-05	6,28141E-05
rxn03900	0	0
rxn03901	0,031494975	0,031494975
rxn03902	0	0
rxn03903	0	0
rxn03904	0,031494975	0,031494975
rxn03907	0	0
rxn03908	0	0
rxn03909	0	0
rxn03910	0	0
rxn03933	0	0
rxn03953	0	0
rxn03954	0	0
rxn03958	0	0
rxn03974	-0,028334856	0
rxn03975	-0,028334856	0
rxn03978	0,004233097	0,463135
rxn04045	0	0
rxn04046	0	0
rxn04047	0	0
rxn04048	0	0
rxn04050	0	0
rxn04052	0	0
rxn04068	0	0
rxn04082	0	0
rxn04113	0	0
rxn04142	0	0
rxn04308	0	0
rxn04384	0	0
rxn04385	0	0
rxn04413	0	0
rxn04432	0	0
rxn04443	0	0
rxn04674	0	0
rxn04676	-0,270954774	1000
rxn04678	-1000	0,270954774
rxn04681	0	0
rxn04682	0	0
rxn04704	0	0
rxn04726	0	0
rxn04736	0	0
rxn04750	0	0
rxn04794	0	1000
rxn04809	0	0
rxn04810	0	0
rxn04811	0	0

rxn04822	0	0
rxn04830	0	0
rxn04831	0	0
rxn04832	0	0
rxn04833	0	0
rxn04872	0	0
rxn04873	0	0
rxn04886	0	0
rxn04887	0	0
rxn04894	0	0
rxn04895	0	0
rxn04896	0	0
rxn04903	0	0
rxn04916	0	0
rxn04919	0	0
rxn04930	0	0
rxn04943	0	0
rxn04954	-12,23749811	0
rxn05005	-1000	0
rxn05006	-1000	0
rxn05010	0	0
rxn05011	0	0
rxn05012	0	0
rxn05028	6,28141E-05	6,28141E-05
rxn05029	0	0
rxn05030	6,28141E-05	6,28141E-05
rxn05039	0	0
rxn05040	0	0
rxn05050	0	0
rxn05054	0	0
rxn05092	0,007702147	0,007702147
rxn05104	0,007702147	0,007702147
rxn05105	0,007702147	0,007702147
rxn05106	0,007702147	0,007702147
rxn05108	0,007702147	0,007702147
rxn05114	0	0
rxn05115	0	0
rxn05234	0	0
rxn05236	0	0
rxn05239	0	0
rxn05247	0	0
rxn05248	0	0
rxn05249	0	0
rxn05250	0	0
rxn05251	0	0
rxn05252	0	0
rxn05256	0	0
rxn05269	0	0
rxn05274	0	0
rxn05275	0	0

rxn05276	0	0
rxn05278	0	0
rxn05279	0	0
rxn05280	0	0
rxn05289	0	0
rxn05291	0	0
rxn05322	0	0
rxn05323	0	0
rxn05324	0	0
rxn05325	0	0
rxn05326	0	0
rxn05327	0	0
rxn05328	0	0
rxn05329	0	0
rxn05330	0	0
rxn05331	0	0
rxn05332	0	0
rxn05333	0	0
rxn05334	0	0
rxn05335	0	0
rxn05336	0	0
rxn05337	0	0
rxn05338	0	0
rxn05339	0	0
rxn05340	0	0
rxn05341	0	0
rxn05342	0	0
rxn05343	0	0
rxn05344	0	0
rxn05345	0	0
rxn05346	0	0
rxn05347	0	0
rxn05348	0	0
rxn05350	0	0
rxn05457	-1000	0
rxn05465	0	0
rxn05733	0	0
rxn05736	0	1000
rxn05740	-1000	1000
rxn05744	0	0
rxn05759	-0,6	0
rxn05760	-491,4341003	1000
rxn05762	0	0
rxn05778	0	0
rxn05779	0	0
rxn05794	-1000	0
rxn05854	0	0
rxn05871	0	0
rxn05872	0	0
rxn05873	0	0

rxn05874	0	0
rxn05887	0	0,6
rxn05899	0	0
rxn05901	0	0
rxn05918	0	0
rxn05927	0	0
rxn05934	0	0
rxn05937	-1000	1000
rxn05938	-11,83953443	0
rxn05939	-0,000111923	1000
rxn05940	-1000	1000
rxn05957	0	1000
rxn05958	0	0
rxn05962	0	0
rxn05965	0	0
rxn05966	0	0
rxn05970	0	0
rxn05990	0	0
rxn05994	0	0
rxn06005	0	0
rxn06023	0	0
rxn06038	0	0
rxn06043	0	0
rxn06044	0	0
rxn06045	0	0
rxn06078	0	0
rxn06081	0	0
rxn06089	0	0
rxn06090	0	0
rxn06091	0	0
rxn06094	0	1000
rxn06096	-1000	0
rxn06108	-1000	0
rxn06109	-6,366109729	0,000254683
rxn06139	0	0
rxn06140	0	0
rxn06155	0	0
rxn06181	0	1000
rxn06182	0	1000
rxn06190	0	0
rxn06195	0	0
rxn06196	0	0
rxn06200	0	0
rxn06201	0	0
rxn06217	0	0
rxn06218	0	0
rxn06219	0	0
rxn06227	0	0
rxn06231	0	0
rxn06244	0	0

rxn06252	-1000	0
rxn06253	0	1000
rxn06280	0	0
rxn06285	0	0
rxn06298	0	0
rxn06299	0	0
rxn06300	0	0
rxn06312	0	0
rxn06316	0	0
rxn06328	0	0
rxn06347	0	0
rxn06348	0	0
rxn06373	0	0
rxn06376	0	0
rxn06394	0	0
rxn06400	0	0
rxn06403	0	0
rxn06432	0	0
rxn06434	0	0
rxn06435	0	0
rxn06437	0	0
rxn06438	0	0
rxn06439	0	0
rxn06440	0	0
rxn06441	0	0
rxn06443	0	0
rxn06444	0	0
rxn06445	0	0
rxn06446	0	0
rxn06447	0	0
rxn06448	0	0
rxn06449	0	0
rxn06485	0	0
rxn06500	0	0
rxn06522	0	0
rxn06538	0	0
rxn06556	0	0
rxn06565	0	0
rxn06581	0	0
rxn06584	0	0
rxn06591	0,00203746	0,00203746
rxn06592	0	0
rxn06595	0	0
rxn06624	0	0
rxn06648	0	0
rxn06664	0	0
rxn06672	0	1000
rxn06673	0	1000
rxn06678	0	0
rxn06691	0	0

rxn06694	0	0
rxn06701	0	0
rxn06726	0	0
rxn06733	0	0
rxn06737	0	0
rxn06751	0	0
rxn06752	0	0
rxn06760	0	0
rxn06768	0	0
rxn06799	0	0
rxn06820	0	0
rxn06823	0	0
rxn06831	0	0
rxn06850	0	0
rxn06860	0	0
rxn06864	0	0
rxn06865	0	0
rxn06874	0	0,1
rxn06882	0	0
rxn06883	0	0
rxn06887	0	0
rxn06889	0	1000
rxn06926	0	0
rxn06936	0	0
rxn06937	0,00203746	0,00203746
rxn06947	0	0
rxn06979	0	0
rxn07056	0	0
rxn07172	0	0
rxn07177	0	0
rxn07181	0	0
rxn07189	0	0
rxn07199	0	0
rxn07200	0	0,6
rxn07256	0	0
rxn07264	0	0
rxn07267	0	0
rxn07270	0	0
rxn07271	0	0
rxn07272	0	0
rxn07292	0	0
rxn07437	0	0
rxn07438	0	0
rxn07441	0	999,968505
rxn07452	0	0
rxn07456	0	1000
rxn07466	-0,029267931	999,9707321
rxn07474	0	0
rxn07476	0	0
rxn07484	0	0

rxn07485	0	0
rxn07486	0	0
rxn07489	0	0
rxn07573	0	0
rxn07577	0	0
rxn07578	0	0
rxn07579	0	0
rxn07580	0	0
rxn07584	0	0
rxn07585	0	0
rxn07586	0	0
rxn07587	0	0
rxn07603	0	0
rxn07679	0	0
rxn07683	0	0
rxn07687	0	0
rxn07845	0	0
rxn07846	0	0
rxn07849	0	0
rxn07987	0	0
rxn07989	0	0
rxn07991	0	0
rxn07992	0	0
rxn07993	0	0
rxn07994	0	0
rxn07997	0	0
rxn07998	0	0
rxn08000	0	0
rxn08001	0	0
rxn08002	0	0
rxn08025	0	0
rxn08040	0	0
rxn08043	0	1,835607611
rxn08067	-1000	1000
rxn08083	0	0
rxn08084	0	1000
rxn08085	0	0
rxn08086	0	0
rxn08087	0	0
rxn08088	0	0
rxn08089	0	0
rxn08126	0	0
rxn08127	0	0
rxn08128	0	0
rxn08129	0	0
rxn08171	0	0
rxn08180	0	0
rxn08194	-1000	1000
rxn08206	0	0
rxn08207	0	0

rxn08208	0	0
rxn08209	0	0
rxn08294	0	0
rxn08295	0	0
rxn08296	0	0
rxn08297	0	0
rxn08298	0	0
rxn08299	0	0
rxn08300	0	0
rxn08306	0	0
rxn08307	0	0
rxn08308	0	0
rxn08309	0	0
rxn08310	0	0
rxn08311	0	0
rxn08312	0	0
rxn08352	0	0
rxn08386	0	0
rxn08390	0	0
rxn08392	0	0
rxn08394	0	0
rxn08396	0	0
rxn08398	0	0
rxn08413	0	0
rxn08433	0	0
rxn08438	0	0
rxn08448	0	0
rxn08449	0	0
rxn08451	0	0
rxn08453	0	0
rxn08454	0	1000
rxn08455	0	0
rxn08456	0	0
rxn08457	0	0
rxn08519	0,057583371	0,057583371
rxn08546	0	0
rxn08547	0	1000
rxn08548	0	0
rxn08549	0	0
rxn08550	0	0
rxn08551	0	0
rxn08552	0	0
rxn08582	0	0,5
rxn08605	0	0
rxn08607	0	0
rxn08615	-1000	1000
rxn08647	0	0
rxn08668	0	0
rxn08669	0	0
rxn08764	0	1,835607611

rxn08796	0	0
rxn08797	0	1000
rxn08798	0	0
rxn08799	0	1000
rxn08800	0	0
rxn08801	0	1000
rxn08802	0	0
rxn08803	0	0
rxn08804	0	0
rxn08805	0	0
rxn08806	0	0
rxn08807	0	0
rxn08808	0	0
rxn08809	0	0
rxn08810	0	0
rxn08811	0	0
rxn08812	0	0
rxn08813	0	0
rxn08814	0	0
rxn08815	0	0
rxn08816	0	0
rxn08817	0	0
rxn08818	0	500
rxn08819	0	0
rxn08820	0	500
rxn08821	0	0
rxn08822	0	500
rxn08823	0	0
rxn08838	0	0
rxn08839	0	0
rxn08840	0	0
rxn08841	0	0
rxn08842	0	0
rxn08843	0	0
rxn08844	0	0
rxn08845	0	0
rxn08846	0	0
rxn08847	0	0
rxn08848	0	0
rxn08849	0	0
rxn08850	0	0
rxn08851	0	0
rxn08857	0	0
rxn08889	0,000768616	0,000768616
rxn08890	0,006222019	0,006222019
rxn08891	0,000768616	0,000768616
rxn08892	0,013058474	0,013058474
rxn08893	0,0061455	0,0061455
rxn08894	0,00153609	0,00153609
rxn08897	-0,006912974	-0,006912974

rxn08926	0,000690955	0,000690955
rxn08928	0,00153609	0,00153609
rxn08929	0,00153609	0,00153609
rxn08958	0,000768616	0,000768616
rxn09010	0	0
rxn09016	0	999,7611923
rxn09069	0	0
rxn09108	0	0
rxn09109	0	0
rxn09110	0	0
rxn09111	0	0
rxn09112	0	0
rxn09113	0	0
rxn09114	0	0
rxn09123	0	0
rxn09124	0	500
rxn09125	0	0
rxn09126	0	500
rxn09127	0	0
rxn09128	0	500
rxn09129	0	0
rxn09130	0	0
rxn09131	0	0
rxn09132	0	0
rxn09133	0	0
rxn09134	0	0
rxn09135	0	0
rxn09136	0	0
rxn09137	0	0
rxn09138	0	0
rxn09139	0	0
rxn09140	0	0
rxn09141	0	0
rxn09142	0	0
rxn09143	0	0
rxn09144	0	0
rxn09145	0	1000
rxn09146	0	0
rxn09147	0	1000
rxn09148	0	0
rxn09149	0	1000
rxn09150	0	0
rxn09151	0	0
rxn09152	0	0
rxn09153	0	0
rxn09154	0	0
rxn09155	0	0
rxn09156	0	0
rxn09157	0	0
rxn09158	0	0

rxn09159	0	0
rxn09160	0	0
rxn09161	0	0
rxn09162	0	0
rxn09163	0	0
rxn09164	0	0
rxn09176	-1000	1000
rxn09177	0	0,000657835
rxn09197	0	0
rxn09198	0	0
rxn09199	0	0
rxn09200	0	0
rxn09201	0	0
rxn09202	0	0
rxn09203	0	0
rxn09205	0	0
rxn09206	0	0
rxn09207	0	0
rxn09208	0	0
rxn09209	0	0
rxn09210	0	0
rxn09211	0	0
rxn09235	0,028334856	0,028334856
rxn09237	0,029248515	0,029248515
rxn09240	0	1000
rxn09244	0	0
rxn09340	0	0
rxn09341	0	999,6310107
rxn09348	0	999,6310107
rxn09355	0	0
rxn09395	0	0
rxn09398	-1000	999,5995158
rxn09399	0	0
rxn09412	-1000	1000
rxn09445	0	0
rxn09446	0	0
rxn09447	0	0
rxn09473	0	0
rxn09486	0	0
rxn09502	0	1000
rxn09531	0	0
rxn09557	0,000254683	1000
rxn09564	0	999,7611923
rxn09565	0	0
rxn09616	0,000690955	0,000690955
rxn09625	0	0
rxn09626	0	0
rxn09632	0	999,9997453
rxn09633	0,000254683	0,000254683
rxn09888	0	0

rxn09889	0	0
rxn09952	0	0
rxn09978	0	0
rxn09979	0	0
rxn09988	0	0
rxn09992	0	0
rxn09995	0	0
rxn10003	0	0,000657835
rxn10019	0	0
rxn10020	0	0
rxn10021	0	0
rxn10026	0	0
rxn10029	0	0
rxn10030	0	0
rxn10034	0	0
rxn10036	0	0
rxn10038	0	0
rxn10052	-1000	1000
rxn10054	0	999,6310107
rxn10056	0	0,000510507
rxn10058	0	0,000510507
rxn10060	0	0,000510507
rxn10091	-1000	1000
rxn10110	0	0
rxn10111	0	0
rxn10191	0	0
rxn10192	0	0
rxn10193	0	0
rxn10194	0	0
rxn10196	0	0
rxn10202	0	1000
rxn10203	0	1000
rxn10204	0	1000
rxn10205	0	0
rxn10206	0	0
rxn10207	0	0
rxn10208	0	0
rxn10209	0	0
rxn10210	0	0
rxn10211	0	1000
rxn10212	0	1000
rxn10213	0	1000
rxn10214	0	0
rxn10215	0	0
rxn10216	0	0
rxn10217	0	0
rxn10218	0	0
rxn10219	0	0
rxn10220	0	0
rxn10221	0	0

rxn10222	0	0
rxn10223	0	0
rxn10224	0	0
rxn10225	0	0
rxn10226	0	0
rxn10227	0	0
rxn10228	0	0
rxn10229	0	0
rxn10230	0	0
rxn10231	0	0
rxn10232	0	0
rxn10233	0	0
rxn10234	0	0
rxn10235	0	0
rxn10236	0	0
rxn10237	0	0
rxn10253	0	0
rxn10254	0	0
rxn10255	0	0
rxn10256	0	0
rxn10257	0	0
rxn10258	0	0
rxn10259	0	0
rxn10260	0	0
rxn10261	0	0
rxn10262	0	0
rxn10263	0	0
rxn10264	0	0
rxn10289	0	0
rxn10290	0	0
rxn10291	0	0
rxn10292	0	0
rxn10293	0	0
rxn10294	0	0
rxn10295	0	0
rxn10296	0	0
rxn10297	0	0
rxn10298	0	0
rxn10299	0	0
rxn10300	0	0
rxn10301	0	0
rxn10302	0	0
rxn10303	0	0
rxn10304	0	0
rxn10305	0	0
rxn10306	0	0
rxn10363	0	0
rxn10404	0	0
rxn10405	0	0
rxn10406	0	0

rxn10407	0	0
rxn10408	0	0
rxn10409	0	0
rxn10410	0	0
rxn10816	0,000254683	0,000254683
rxn10951	0	0,028334856
rxn11007	0,028334856	0,028334856
rxn11510	0	0
rxn11513	0	0
rxn11547	0	0
rxn11548	0	0
rxn11550	0	0
rxn11551	-1000	1000
rxn11552	-1000	1000
rxn11567	0	0
rxn11571	0	0
rxn11587	0	0
rxn11609	0	0
rxn11641	0	0
rxn11702	0	0
rxn11728	0	0
rxn11732	0	0
rxn11749	0	0
rxn11755	0	0
rxn11757	-999,988931	0
rxn11759	0	999,988931
rxn11760	-999,988931	0
rxn11765	0	0
rxn11766	0	0
rxn11768	0	0
rxn11772	0	0
rxn11773	0	0
rxn11788	0	0
rxn11808	0	0
rxn11946	0	0
rxn11951	0	0
rxn11965	0	0
rxn11977	0	0
rxn12008	-6,28141E-05	-6,28141E-05
rxn12013	0	0
rxn12049	0	0
rxn12147	0	0
rxn12154	0	0
rxn12218	-1000	-0,000254683
rxn12221	0,000254683	1000
rxn12239	0,000254683	0,000254683
rxn12510	0,000657835	0,000657835
rxn12649	-999,9989813	0
rxn12707	0	0
rxn12767	0	0

rxn12768	0	0
rxn12769	0	0
rxn12770	0	0
rxn12771	0	0
rxn12822	-1000	0
rxn13147	0,000254683	0,000254683
rxn13420	0,000690955	1000
rxn13421	0,000690955	1000
rxn13477	6,28141E-05	6,28141E-05
rxn13666	0	0
rxn13667	0	0
rxn13668	0	0
rxn13669	0	0
rxn13671	0	0
rxn13672	0	0
rxn13673	0	0
rxn13705	0	0
rxn13741	0	0
rxn13936	0,015363179	0,01536318
rxn13954	0	1000
rxn13962	0	0
rxn13974	-11,83953443	0
rxn13994	-2,315675	-0,021165486
rxn14007	-1000	0
rxn14012	6,28141E-05	6,28141E-05
rxn14028	0	0
rxn14029	0	0
rxn14043	0	0
rxn14048	-1000	0
rxn14050	0	0
rxn14054	-1000	0
rxn14058	-0,2315675	-0,002116549
rxn14063	0	0
rxn14070	0	0
rxn14089	-1000	0
rxn14093	0	0
rxn14109	0	0
rxn14120	-1000	-0,00101873
rxn14132	0	0
rxn14136	0	0
rxn14152	0	2,79999E-09
rxn14178	-1000	1000
rxn14191	0	0
rxn14250	0	0
rxn14270	0	0
rxn14276	0	1000
rxn14279	0	0
rxn14346	0	0
rxn90002	-11,12088206	1000
rxn90003	0	0

rxn90004	0	0
rxn90005	-0,028845363	-0,028334856
rxn08173	0	500
Biomass_Bacteria	1,142074	1,142074006
t_Cl	0,005153038	0,005153038
t_Sulfate	0,004294198	0,004294198
t_Cu2+	0,003435359	0,003435359
t_Mg	0,008587254	0,008587254
t_Ca2+	0,005153038	0,005153038
t_NH3	-1,835607611	0
t_H2O	-21,60853473	8,109516612
t_Biomass	-1,142074006	-1,142074
t_Butyrate	0	0
t_D-Lactate	0	0
t_Ethanol	-1,302559388	0
t_Formate	-12,73272882	0
t_H2	-0,1	0,5
t_L-Lactate	-11,83953443	0
t_Nitrite	0	0
t_Phosphate	1,517653029	2,011966651
t_Propionate	-11,9077448	0
t_O2	0	0
t_D-Glucose	0	0,5
t_CO2	-12,73272882	0
t_Acetate	-19,24737783	0
t_Succinate	-6,366364412	0
t_H2S	-0,246931251	0
Ex_Cl	-0,005153038	-0,005153038
Ex_Sulfate	-0,004294198	-0,004294198
Ex_Cu2+	-0,003435359	-0,003435359
Ex_Mg	-0,008587254	-0,008587254
Ex_Ca2+	-0,005153038	-0,005153038
Ex_NH3	0	1,835607611
Ex_H2O	-8,109516612	21,60853473
Ex_Biomass	1,142074	1,142074006
Ex_Butyrate	0	0
Ex_D-Lactate	0	0
Ex_Ethanol	0	1,302559388
Ex_Formate	0	12,73272882
Ex_H2	-0,5	0,1
Ex_L-Lactate	0	11,83953443
Ex_Nitrite	0	0
Ex_Phosphate	-2,011966651	-1,517653029
Ex_Propionate	0	11,9077448
Ex_O2	0	0
Ex_D-Glucose	-0,5	0
Ex_CO2	0	12,73272882
Ex_Acetate	0	19,24737783
Ex_Succinate	0	6,366364412
Ex_H2S	0	0,246931251

t_Fe2	0,007983097	0,007983097
t_fe3	0,007728415	0,007728415
t_Acetaldehyde	-1,302559388	0
t_Adenosine	0	0,494313614
t_AMP	0	0,494313614
t_Amylotriose	0	0
t_BIOT	0	0
t_Choline	0	0
t_Cytidine	0	0
t_Cytosine	0	0
t_DAlanine	0	0
t_Deoxyadenosine	0	0,494313614
t_Deoxycytidine	0	0,365829146
t_Deoxyguanosine	0	0
t_Deoxyinosine	0	0
t_Deoxyuridine	0	0
t_DRibose	0	0,5
t_DSerine	0	0
t_Glycerol	0	0
t_GSH	0	0
t_Guanine	0	0
t_H2S2O3	0	0
t_Heme	0,000254683	0,000254683
t_Homocysteine	0	0
t_HYXN	0	0,494313614
t_Inosine	0	0,494313614
t_LACT	0	0,5
t_LAlanine	-1,335607611	0,5
t_LArabinose	0	0
t_LArginine	-0,134902932	0,323998972
t_LAsparagine	-0,417803805	0,5
t_LAspartate	-1,335607611	0,5
t_LCysteine	0,253068749	0,5
t_LGlutamate	-1,335607611	0,5
t_LGlutamine	-0,417803805	0,5
t_LHistidine	0,105185015	0,105185016
t_LInositol	0	0
t_LIsoleucine	-1,51320012	0,322407492
t_LLeucine	0,499999997	0,5
t_LLysine	-0,537036334	0,380767474
t_LMethionine	-0,226433304	0,020497947
t_LPhenylalanine	-1,630034291	0,205573321
t_LThreonine	0,201145494	0,5
t_LTryptophan	-0,017299	0,063076747
t_LTyrosine	-1,682315012	0,153292599
t_LValine	-1,36518733	0,470420283
t_Maltose	0	0,5
t_Niacin	0	0,002602787
t_Ornithine	0	0
t_PPi	0	0

t_Pyridoxol	0	0
t_XAN	0	0
t_5Deoxyadenosine	0	0
t_BET	0	0
t_Calomide	0	0
t_Cbl	0	0
t_Citrate	0	0
t_CysGly	0	0
t_Dulcose	0	0
t_Glycine	0,419624253	0,5
t_Glycolaldehyde	0	0
t_LProline	0,245317495	0,245317497
t_Maltohexaose	0	0
t_Methanol	0	0
t_NAcetylDglucosamine	0	0
t_PM	0	0
t_Putrescine	0	0
t_Pyridoxal	0,000254683	0,000254683
t_Riboflavin	0,000509365	0,000509365
t_Salicin	0	0
t_Sorbitol	0	0
t_Spermidine	0	0
t_Sucrose	0	0
t_Taurine	0	0
t_Thiamin	0	0
t_Thyminose	0	0,5
t_TRHL	0	0
t_Uracil	0	0,365829146
t_Uridine	0	0,365829146
t_Ursin	0	0
t_Mn2+	0,003435359	0,003435359
t_Formaldehyde	0	0
t_Fumarate	-6,366364412	0
t_Oxidized glutathione	0	0
t_Adenine	0	0
t_Nicotinamide	0	0
t_4-Hydroxybenzoate	0	0
t_Co2+	0,003435359	0,003435359
t_D-Arabinose	0	0,5
t_D-Glutamate	0	0
t_Chorismate	0	0
t_Folate	0,00101873	0,00101873
t_N-Acetyl-D-mannosamine	0	0
t_Siroheme	0	0
t_Selenate	0	0
t_Menaquinone 7	0	0
t_2-Demethylmenaquinone 8	0	0
t_Menaquinone 8	0	0
t_Ubiquinone-8	0	0
t_2-Oxobutyrate	0	0

t_3MOP	0	0
t_ABEE	0	0
t_Neu5Ac	0	0
t_Glycerol-3-phosphate	0	0
t_H+	-1000	0,5
t_indol	0	0
t_Nicotinamide ribonucleotide	0	0
t_PAN	0,000657835	0,000657835
t_Pyridoxal phosphate	0	0
t_Zn2+	0,003435359	0,003435359
t_1,2-Diacyl-sn-glycerol dioctadecanoyl	0	0
t_meso-2,6-Diaminopimelate	0	0
t_L-Serine	0,419624253	0,5
t_D-Fructose	0	0,5
t_D-Mannose	0	0
t_L-Rhamnose	0	0
t_beta D-Galactose	0	0,5
t_L-Fucose	0	0
Ex_Fe2	-0,007983097	-0,007983097
Ex_fe3	-0,007728415	-0,007728415
Ex_Acetaldehyde	0	1,302559388
Ex_Adenosine	-0,494313614	0
Ex_AMP	-0,494313614	0
Ex_Amylotriose	0	0
Ex_BIOT	0	0
Ex_Choline	0	0
Ex_Cytidine	0	0
Ex_Cytosine	0	0
Ex_DAlanine	0	0
Ex_Deoxyadenosine	-0,494313614	0
Ex_Deoxycytidine	-0,365829146	0
Ex_Deoxyguanosine	0	0
Ex_Deoxyinosine	0	0
Ex_Deoxyuridine	0	0
Ex_DRibose	-0,5	0
Ex_DSerine	0	0
Ex_Glycerol	0	0
Ex_GSH	0	0
Ex_Guanine	0	0
Ex_Heme	-0,000254683	-0,000254683
Ex_Homocysteine	0	0
Ex_HYXN	-0,494313614	0
Ex_Inosine	-0,494313614	0
Ex_LACT	-0,5	0
Ex_LAlanine	-0,5	1,335607611
Ex_LArabinose	0	0
Ex_LArginine	-0,323998972	0,134902932
Ex_LAsparagine	-0,5	0,417803805
Ex_LAspartate	-0,5	1,335607611
Ex_LCysteine	-0,5	-0,253068749

Ex_LGlutamate	-0,5	1,335607611
Ex_LGlutamine	-0,5	0,417803805
Ex_LHistidine	-0,105185016	-0,105185015
Ex_LInositol	0	0
Ex_LIsoleucine	-0,322407492	1,51320012
Ex_LLeucine	-0,5	-0,499999997
Ex_LLysine	-0,380767474	0,537036334
Ex_LMethionine	-0,020497947	0,226433304
Ex_LPhenylalanine	-0,205573321	1,630034291
Ex_LThreonine	-0,5	-0,201145494
Ex_LTryptophan	-0,063076747	0,017299
Ex_LTyrosine	-0,153292599	1,682315012
Ex_LValine	-0,470420283	1,36518733
Ex_Maltose	-0,5	0
Ex_Niacin	-0,002602787	0
Ex_Ornithine	0	0
Ex_PPi	0	0
Ex_XAN	0	0
Ex_5Deoxyadenosine	0	0
Ex_BET	0	0
Ex_Calomide	0	0
Ex_Cbl	0	0
Ex_Citrate	0	0
Ex_CysGly	0	0
Ex_Dulcose	0	0
Ex_Glycine	-0,5	-0,419624253
Ex_Glycolaldehyde	0	0
Ex_LProline	-0,245317497	-0,245317495
Ex_Maltohexaose	0	0
Ex_Methanol	0	0
Ex_NAcetylDglucosamine	0	0
Ex_PM	0	0
Ex_Putrescine	0	0
Ex_Pyridoxal	-0,000254683	-0,000254683
Ex_Riboflavin	-0,000509365	-0,000509365
Ex_Salicin	0	0
Ex_Sorbitol	0	0
Ex_Spermidine	0	0
Ex_Sucrose	0	0
Ex_Taurine	0	0
Ex_Thiamin	0	0
Ex_Thyminose	-0,5	0
Ex_TRHL	0	0
Ex_Uracil	-0,365829146	0
Ex_Uridine	-0,365829146	0
Ex_Ursin	0	0
Ex_Mn2+	-0,003435359	-0,003435359
Ex_Formaldehyde	0	0
Ex_Fumarate	0	6,366364412
Ex_Oxidized glutathione	0	0

Ex_Adenine	0	0
Ex_Nicotinamide	0	0
Ex_4-Hydroxybenzoate	0	0
Ex_Co2+	-0,003435359	-0,003435359
Ex_D-Arabinose	-0,5	0
Ex_D-Glutamate	0	0
Ex_Folate	-0,00101873	-0,00101873
Ex_N-Acetyl-D-mannosamine	0	0
Ex_Siroheme	0	0
Ex_Selenate	0	0
Ex_Menaquinone 7	0	0
Ex_2-Demethylmenaquinone 8	0	0
Ex_Menaquinone 8	0	0
Ex_Ubiquinone-8	0	0
Ex_ABEE	0	0
Ex_Neu5Ac	0	0
Ex_H+	-0,5	1000
Ex_indol	0	0
Ex_Nicotinamide ribonucleotide	0	0
Ex_PAN	-0,000657835	-0,000657835
Ex_Zn2+	-0,003435359	-0,003435359
Ex_1,2-Diacyl-sn-glycerol dioctadecanoyl	0	0
Ex_L-Serine	-0,5	-0,419624253
Ex_D-Fructose	-0,5	0
Ex_D-Mannose	0	0
Ex_L-Rhamnose	0	0
Ex_beta D-Galactose	-0,5	0
Ex_L-Fucose	0	0
t_Arabinan	0	0
t_Starch	0	0,005
t_octanoate	0	0
t_Melibiose	0	0,5
t_Amylose	0	0
Ex_Arabinan	0	0
Ex_Starch	-0,005	0
Ex_Melibiose	-0,5	0
Ex_Amylose	0	0
t_Raffinose_Melitose	0	0
t_Isovaleric_acid	-2,79999E-09	0
t_H2O2	0	0
t_Nitric_oxide	0	0
Ex_Raffinose_Melitose	0	0
Ex_Isovaleric_acid	0	2,8E-09
Ex_H2O2	0	0
Ex_Nitric_oxide	0	0
rxn01207_1	0	2,8E-09
rxn08972	0	0
rxn08973	0	0
rxn06111	0	1000
rxn13726	0	0

rxn13727	0	0
rxn13729	0	0
rxn08974	0	0
rxn10122	0	0
rxn10123	0	0
rxn10124	0	0
rxn12665	0	0
rxn06097	0	0,005
t_Sulfite	0	0
Ex_Sulfite	0	0
t_Nitrogen	0	0,1
Ex_Nitrogen	-0,1	0

rxn ID	minFlux	max Flux
rxn00001	0	1000
rxn00003	-22,37277897	0
rxn00007	0	1000
rxn00011	-22,37277897	0
rxn00016	0	0
rxn00020	0	1000
rxn00022	0	0,505
rxn00029	0,00101873	0,00101873
rxn00060	0,000254683	0,000254683
rxn00062	0	1000
rxn00063	0	1000
rxn00065	0	0
rxn00067	0	0
rxn00070	0	0
rxn00076	0	1000
rxn00077	0	0,000510507
rxn00085	-1000	0
rxn00086	0	0
rxn00097	-1000	1000
rxn00100	0,000657835	0,000657835
rxn00101	0	0,865644133
rxn00103	0	1000
rxn00104	-1000	0
rxn00105	-999,9973972	1000
rxn00106	-1000	0
rxn00109	0	0
rxn00113	0	1000
rxn00114	-1000	0,693876201
rxn00119	0,368989262	1000
rxn00121	-0,000254683	0
rxn00122	0	0,000254683
rxn00124	0,000254683	0,000254683
rxn00126	0,000764048	0,000764048
rxn00131	-1000	999,9966332
rxn00132	0	1000
rxn00133	0	0
rxn00137	0	0
rxn00139	-999,9971425	0
rxn00141	-999,9994906	0
rxn00142	0	0
rxn00143	0,000509365	1000
rxn00144	0	0
rxn00147	0	1000
rxn00148	-1000	0
rxn00151	-1000	0
rxn00154	0	22,37277897
rxn00157	-22,37277897	0
rxn00159	-1000	1000
rxn00161	-1000	1000

rxn00162	0	0,693876201
rxn00165	0	7,776012932
rxn00171	0	1,302559388
rxn00173	0	1000
rxn00175	0	1000
rxn00176	0	0
rxn00179	0	0
rxn00184	-1000	0
rxn00187	0	1000
rxn00189	0	1000
rxn00190	0,002602787	1000
rxn00191	-1000	1000
rxn00192	0	1000
rxn00193	-999,9407732	1000
rxn00196	0	0
rxn00198	0	197,1758569
rxn00199	0	197,0130681
rxn00202	0	0
rxn00206	0,000254683	169,0589881
rxn00211	0	0
rxn00212	0	999,6310107
rxn00214	-1,5	0
rxn00216	0	1000
rxn00221	0	1000
rxn00222	0	1000
rxn00224	0,000254683	1000
rxn00225	-1000	0
rxn00226	0	0
rxn00227	0	1000
rxn00239	0,238807673	1000
rxn00242	0	690,3619171
rxn00245	0	0
rxn00247	0	0,693876201
rxn00250	-0,693911605	-3,54043E-05
rxn00256	-0,693876201	0
rxn00260	-169,0586981	0,693911605
rxn00262	0	169,0587335
rxn00275	-0,000254683	-0,000254683
rxn00283	-999,9407732	1000
rxn00290	-7,621066606	-0,000690955
rxn00293	0,062989949	999,6625057
rxn00297	0	0
rxn00301	0	999,7611923
rxn00303	0	690,3619171
rxn00304	-1000	0
rxn00307	0	0
rxn00313	0	0,693876201
rxn00322	0	0
rxn00333	0,000254683	169,0589881
rxn00337	0,039197122	0,733073323

rxn00338	0	0,002602787
rxn00340	0	1000
rxn00342	0	1000
rxn00347	0	0,693876201
rxn00350	-0,000254683	-0,000254683
rxn00358	0	0
rxn00359	0	0
rxn00360	0	0
rxn00362	0	0
rxn00363	0	1000
rxn00364	-999,5995158	999,999309
rxn00365	0	1000
rxn00367	0	1000
rxn00368	0	999,5995158
rxn00369	0	1000
rxn00371	0	1000
rxn00391	0	999,9997453
rxn00392	0,000254683	1000
rxn00405	0	0,865644133
rxn00407	0	1000
rxn00410	-999,8225103	1000
rxn00411	-1000	0
rxn00412	0	1000
rxn00414	0	0,693876201
rxn00416	0	1000
rxn00420	0	0
rxn00423	0	7,776012932
rxn00426	0	0
rxn00436	0	999,9997453
rxn00437	0	0
rxn00440	0,000254683	1000
rxn00453	0	999,9997453
rxn00456	0	999,9997453
rxn00459	-0,948303459	13,96688252
rxn00460	-1000	-0,400484237
rxn00461	0,031494975	0,031494975
rxn00463	0	999,5995158
rxn00469	0	1000
rxn00470	0	7,666510873
rxn00474	0	0
rxn00490	0	0
rxn00493	-1,981766811	0
rxn00498	0	0
rxn00499	-14,91518598	0
rxn00500	-14,91518598	0
rxn00506	0	1,302559388
rxn00510	0	0
rxn00512	-169,0587335	0
rxn00514	0	0
rxn00517	-1000	0

rxn00527	-1,981766811	0
rxn00533	-999,9999646	3,54043E-05
rxn00536	-1000	1000
rxn00539	0	0
rxn00541	-1,302559388	0
rxn00543	-1000	1000
rxn00545	0	1000
rxn00546	0	0
rxn00547	0	1000
rxn00549	0	1000
rxn00551	0	1000
rxn00552	-0,06298995	999,9370101
rxn00554	0	1000
rxn00555	0	1000
rxn00556	0	999,5995158
rxn00557	0	1000
rxn00558	-1000	1000
rxn00559	0	0
rxn00560	0	0
rxn00562	0	0
rxn00565	0	0
rxn00566	0	1000
rxn00575	0	0,5
rxn00585	0	0
rxn00592	0	0
rxn00606	0	0
rxn00607	0	0
rxn00608	0	0
rxn00609	0	0
rxn00611	0	0
rxn00615	0	0
rxn00621	0	0
rxn00622	0	0
rxn00623	0	0
rxn00633	0	0
rxn00634	0	1000
rxn00641	0	0
rxn00642	0	0
rxn00647	0	0
rxn00649	0	7,776012932
rxn00650	-0,000254683	-0,000254683
rxn00653	0	0
rxn00659	0	0
rxn00661	0	0
rxn00670	0	1000
rxn00674	0	1000
rxn00675	0	0
rxn00677	-1000	1000
rxn00684	0	0
rxn00685	0	197,0130681

rxn00686	0	0
rxn00687	0	197,0130681
rxn00689	0	0
rxn00690	0	15,2407513
rxn00692	-0,260507082	7,51550585
rxn00693	0	0,397709
rxn00695	-1000	1000
rxn00698	-1000	0
rxn00701	0	1000
rxn00704	-1000	1,5
rxn00707	0	1000
rxn00708	0	1000
rxn00709	0	1000
rxn00710	0	0
rxn00711	-999,9971425	0
rxn00712	0	999,5995158
rxn00713	0	1000
rxn00714	0	0
rxn00715	0	1000
rxn00726	0	0,990883405
rxn00727	0	0,990883405
rxn00735	0	0
rxn00737	0	1,521038145
rxn00740	0	1000
rxn00741	0	0
rxn00742	-1000	0,000254683
rxn00743	0	0
rxn00747	-6,503239303	0,788775952
rxn00748	0	0
rxn00758	0	0
rxn00762	0	0
rxn00763	0	0
rxn00765	0	0
rxn00770	0,002857469	1000
rxn00772	0,000509365	1000
rxn00775	0	0
rxn00777	-1,861161307	0,369817268
rxn00778	-1000	1000
rxn00781	-0,948303459	13,96688252
rxn00784	0	1,302559388
rxn00785	-0,517987285	1,589904841
rxn00786	-1000	6,503239303
rxn00787	0	0
rxn00789	0	0
rxn00790	-0,000254683	-0,000254683
rxn00791	-0,990883405	0
rxn00792	0	0
rxn00796	0	0
rxn00799	-0,425129058	7,765544392
rxn00800	-0,26805619	0,425820012

rxn00802	0	0,693876201
rxn00806	0	0
rxn00808	0	1000
rxn00809	-98,50653405	0
rxn00811	-98,50653405	0
rxn00816	0	0,5
rxn00817	0	0,5
rxn00818	0	0
rxn00819	0	0
rxn00827	0	0
rxn00829	0,000690955	0,000690955
rxn00830	6,28141E-05	6,28141E-05
rxn00831	0	999,9971425
rxn00832	0	0
rxn00834	-999,7290863	1000
rxn00836	-999,9971425	0
rxn00838	-0,26805619	0,425820012
rxn00849	-999,968505	999,9722682
rxn00851	0	1000
rxn00856	0,008138419	7,628514071
rxn00858	0	0,865644133
rxn00864	0	0
rxn00869	0	0
rxn00879	0	0
rxn00881	0	0
rxn00882	0	0
rxn00883	0	0
rxn00889	0	0
rxn00890	0	0
rxn00898	0	1,981766811
rxn00902	0	0
rxn00903	-1000	1000
rxn00904	-1000	1000
rxn00907	-15,24024194	0,000509365
rxn00908	-8,150954971	0,033850505
rxn00909	-0,007702147	-0,007702147
rxn00910	-0,40566583	0
rxn00913	0	1000
rxn00915	-999,9707515	0
rxn00916	-999,7319438	1000
rxn00917	0	1000
rxn00918	0	0
rxn00921	0	0
rxn00925	0	0
rxn00926	0	0,693876201
rxn00929	-1000	1000
rxn00931	-1000	1000
rxn00938	0	999,9971425
rxn00943	0	1000
rxn00947	0	1000

rxn00950	-1000	0,397199635
rxn00952	0	999,9997453
rxn00955	0,000509365	1000
rxn00973	-1000	1000
rxn00974	-1000	1000
rxn00977	0	0
rxn00979	0,000254683	0,000254683
rxn00980	0	0
rxn00983	0	0
rxn00985	-1000	0
rxn00986	0	0
rxn00991	-0,000690955	-0,000690955
rxn01000	0	1,981766811
rxn01008	0	0
rxn01011	0	0
rxn01016	0	0
rxn01018	0	0
rxn01019	0	0,693876201
rxn01021	0	0
rxn01034	0	0
rxn01035	0	0
rxn01041	-1000	1000
rxn01042	-1000	1000
rxn01043	0	0
rxn01056	-1000	1000
rxn01069	0	0
rxn01073	0	0
rxn01089	0	0
rxn01100	-1000	0
rxn01101	0	0
rxn01103	0	1000
rxn01106	-13,96688252	0,948303459
rxn01114	0	0,5
rxn01116	-1,69449464	0,703150601
rxn01117	0	0
rxn01119	0	0
rxn01122	0	0
rxn01123	0	0
rxn01124	0	0
rxn01133	0	0
rxn01137	0	0,693876201
rxn01138	-1000	1000
rxn01152	0	0,5
rxn01153	0	0
rxn01156	0	0
rxn01169	0	1000
rxn01171	-1000	1000
rxn01199	0	0
rxn01200	0	1000
rxn01201	-7,621066606	-0,000690955

rxn01204	0,000690955	7,621066606
rxn01210	0	0
rxn01211	-15,24049662	0,000509365
rxn01213	6,28141E-05	6,28141E-05
rxn01225	0	999,9707515
rxn01228	0	0
rxn01237	0	0
rxn01241	0	22,37277897
rxn01242	0	22,37277897
rxn01249	0	0
rxn01255	0,000254683	1,982021493
rxn01256	0	1,981766811
rxn01259	0	0
rxn01261	0	0
rxn01265	-999,9997453	0
rxn01268	0	1,981766811
rxn01274	0	0
rxn01275	0	0
rxn01276	0	0
rxn01278	0	0
rxn01286	0	0
rxn01290	0	0
rxn01291	0	0
rxn01292	0	0,5
rxn01297	-999,7319438	999,9971425
rxn01300	0	0
rxn01303	0	0
rxn01304	0	0
rxn01305	0	0
rxn01308	0	0
rxn01310	-1000	0
rxn01316	-1000	0
rxn01321	0	0
rxn01322	0	1000
rxn01329	0	0
rxn01332	0,000254683	1,982021493
rxn01333	-1000	1,056887856
rxn01334	0	1000
rxn01343	0	1000
rxn01346	0	1000
rxn01347	0	999,5995158
rxn01348	0	1000
rxn01351	0	1000
rxn01352	-1000	-0,029248515
rxn01354	-1000	0
rxn01355	0	0
rxn01358	0	999,9971425
rxn01361	0	0
rxn01362	0	0
rxn01366	-0,33749429	1000

rxn01368	0	690,3526172
rxn01370	0	1000
rxn01380	0	0
rxn01387	-197,0130681	0
rxn01388	-1000	1000
rxn01390	0	0
rxn01396	0	0
rxn01406	0	0
rxn01416	0	0
rxn01423	0	0
rxn01426	0	0
rxn01431	0	0
rxn01434	0	0,693876201
rxn01441	0	0
rxn01445	0	999,9707515
rxn01446	-0,029248515	-0,029248515
rxn01453	0	0
rxn01455	0	0
rxn01459	0	7,620811924
rxn01463	0	0
rxn01465	0	0
rxn01466	6,28141E-05	6,28141E-05
rxn01476	0	0
rxn01484	0	0
rxn01485	-0,06298995	-0,062989949
rxn01486	0	0
rxn01492	0	0
rxn01500	-0,000690955	-0,000690955
rxn01506	0	0
rxn01509	-999,9707515	1000
rxn01510	0	1000
rxn01513	0,028334856	0,028334856
rxn01518	0,028334856	1000
rxn01519	0	0
rxn01521	0	999,9716651
rxn01522	0	0
rxn01539	-1000	-0,000254683
rxn01544	-999,9971425	0
rxn01548	0,029248515	1000
rxn01562	0	0
rxn01575	-1,521292828	0
rxn01601	0	0
rxn01602	0	0
rxn01603	0	0
rxn01605	0	0
rxn01610	0	0
rxn01615	0	0
rxn01620	0	0
rxn01621	0	0
rxn01626	0	0

rxn01629	-0,00203746	-0,00203746
rxn01636	-1000	7,90224095
rxn01637	-7,90224095	0
rxn01643	-0,733073323	-0,039197122
rxn01644	0,031494975	0,725371176
rxn01646	0	999,9971425
rxn01647	0	999,9971425
rxn01649	0	999,9971425
rxn01653	0	0
rxn01654	0	0
rxn01667	0	0
rxn01669	0	999,9973972
rxn01670	0	999,9971425
rxn01675	0	0
rxn01679	0	0
rxn01682	0	0
rxn01684	0	0
rxn01706	0	0
rxn01710	0	0
rxn01735	0	0
rxn01737	0	0
rxn01739	0,000254683	1,982021493
rxn01740	-1,982021493	-0,000254683
rxn01741	0	0
rxn01747	0	0
rxn01757	0	0
rxn01763	0	0,5
rxn01775	0	0
rxn01790	0	0
rxn01799	-0,028334856	0,33749429
rxn01800	0	0,365829146
rxn01807	0	0
rxn01816	0	1000
rxn01819	0	0
rxn01834	0	0
rxn01835	0	0
rxn01851	0	7,620811924
rxn01858	0	0,693876201
rxn01859	-0,693876201	0,494313614
rxn01860	0	0
rxn01870	0	0
rxn01871	-22,37277897	0
rxn01879	0	0
rxn01885	0	0
rxn01892	0	0
rxn01906	0	0
rxn01917	0	7,90224095
rxn01937	0	0
rxn01953	0	0
rxn01961	0	999,9971425

rxn01962	0	0
rxn01964	0	0,990883405
rxn01966	-1000	0
rxn01967	-1000	0
rxn01972	0,031494975	1000
rxn01973	0	0
rxn01974	0,031494975	0,725371176
rxn01977	-1000	1000
rxn01982	0	0
rxn01985	0	0,693876201
rxn01986	-0,057583371	0,802559388
rxn01987	-0,5	0
rxn01991	0	0
rxn01997	0	0
rxn01998	0	0
rxn01999	0	0
rxn02000	0	0
rxn02003	0	0
rxn02007	0	0
rxn02008	0,031494975	0,031494975
rxn02011	0,031494975	0,031494975
rxn02015	0	0
rxn02020	0	0
rxn02021	0	0
rxn02023	0	0
rxn02029	0	0
rxn02046	0	0
rxn02056	0	999,9997453
rxn02061	0	0
rxn02062	0	0
rxn02093	0	0
rxn02106	0	0
rxn02118	0	0
rxn02122	0	0
rxn02128	0	0
rxn02138	0	0
rxn02139	0	0
rxn02144	0	0
rxn02154	0	999,9973972
rxn02155	0,002602787	1000
rxn02160	0	0
rxn02161	0	0
rxn02171	0,000690955	7,621066606
rxn02175	0,000657835	1000
rxn02176	0	999,9993422
rxn02185	-1,981766811	22,37277897
rxn02186	0	1,981766811
rxn02187	0	0
rxn02190	0	0
rxn02199	0	0

rxn02200	0	0
rxn02201	0	0
rxn02202	0	0
rxn02203	0	0
rxn02209	0	0
rxn02212	0,000254683	1,982021493
rxn02213	0,000254683	1,982021493
rxn02228	0	0
rxn02262	0	0
rxn02263	0	0
rxn02264	0,000254683	0,000254683
rxn02277	0	0
rxn02284	-0,031494975	0
rxn02285	-0,031494975	0
rxn02286	0,031494975	0,031494975
rxn02287	-197,0130681	1000
rxn02288	0	0
rxn02297	0	0
rxn02302	-1000	-0,000254683
rxn02305	0,000254683	0,000254683
rxn02314	0	1000
rxn02315	0	1000
rxn02316	0	999,5995158
rxn02317	-1000	0
rxn02320	0	0
rxn02322	0,000690955	0,000690955
rxn02339	0	0
rxn02341	0,000657835	0,000657835
rxn02342	0	22,37277897
rxn02350	0	0
rxn02351	0	0
rxn02352	0	0
rxn02356	-1000	1000
rxn02358	-1000	1000
rxn02373	-1000	1000
rxn02375	0	0
rxn02380	-1000	1000
rxn02400	0	999,9971425
rxn02402	-0,002602787	0
rxn02405	0	0
rxn02409	0	0
rxn02410	0	0
rxn02415	0	0
rxn02449	0	690,3619171
rxn02465	-7,90224095	0
rxn02473	0	0
rxn02476	0,000254683	1,982021493
rxn02483	0	0
rxn02484	0,000254683	0,000254683
rxn02495	0	0

rxn02504	0	0
rxn02507	0	0,990883405
rxn02508	0	0,990883405
rxn02518	0	0
rxn02569	0	0
rxn02571	0	0
rxn02581	0	0
rxn02596	0	0
rxn02597	0	0
rxn02663	0	0
rxn02729	0	0
rxn02749	0	0
rxn02751	0	0
rxn02760	0	0
rxn02774	-197,0130681	0
rxn02775	0	0
rxn02776	0	0
rxn02789	0	0
rxn02795	0	0
rxn02796	0	0
rxn02798	0	0
rxn02811	0	0
rxn02822	0	0
rxn02834	0	0
rxn02835	0	0
rxn02853	0	0
rxn02875	0	0
rxn02892	0	0
rxn02895	0,000254683	0,000254683
rxn02897	0	0
rxn02914	0	0
rxn02922	0	0
rxn02928	-1000	999,968505
rxn02929	-1000	999,968505
rxn02931	0	0
rxn02936	0	0
rxn02937	0,000254683	0,000254683
rxn02943	0	0
rxn02981	0	1000
rxn02982	-1000	0
rxn02986	0	0
rxn02988	-0,002602787	0
rxn02990	0	0
rxn03003	0	0
rxn03004	0	0,000254683
rxn03005	-0,000254683	0
rxn03008	0	0
rxn03030	0,031494975	1000
rxn03036	0	0
rxn03039	0	0

rxn03047	0	0
rxn03052	0	0
rxn03055	0	0
rxn03056	0	0
rxn03060	0	0
rxn03062	0	0
rxn03063	0	0
rxn03064	0	0
rxn03066	0	0
rxn03068	0	0
rxn03075	0	0
rxn03084	0,000254683	0,000254683
rxn03086	-1000	-0,031494975
rxn03087	0	0
rxn03094	0	0
rxn03095	0	0
rxn03102	0	0
rxn03106	0	0
rxn03108	0,000254683	0,000254683
rxn03123	0	0
rxn03130	0	0
rxn03135	0	0
rxn03136	0	0
rxn03137	0	0
rxn03140	0	0
rxn03141	0	0
rxn03147	0	0
rxn03150	0	0
rxn03158	0	0
rxn03164	0,031494975	0,031494975
rxn03167	0	0
rxn03175	0	0
rxn03194	0	1,521292828
rxn03263	0	0
rxn03264	0	0
rxn03269	0	0
rxn03273	0	0
rxn03282	0	0
rxn03292	0	0
rxn03293	0	0
rxn03295	0	0
rxn03296	0	0
rxn03301	0	0
rxn03304	0	0
rxn03313	0	0
rxn03316	0	0
rxn03333	0	0
rxn03354	0	0
rxn03374	0	0
rxn03378	0	0

rxn03379	0	0
rxn03382	0	0
rxn03383	0	0
rxn03384	0	0
rxn03387	0	0
rxn03393	0	0
rxn03395	0	0
rxn03397	0	0
rxn03402	0	0
rxn03405	0	0
rxn03406	0	0
rxn03407	0	0
rxn03408	0,031494975	0,031494975
rxn03409	0	0
rxn03423	0	0
rxn03426	0	0
rxn03435	-1,521292828	0
rxn03436	0	1,521292828
rxn03437	0	1,521292828
rxn03445	0	0
rxn03446	0	0
rxn03462	0	0
rxn03468	0	0
rxn03481	0	0
rxn03482	0	0
rxn03489	0	0
rxn03491	0	0
rxn03492	0	0
rxn03512	0	0
rxn03513	0	0
rxn03514	0	0
rxn03535	0	0
rxn03536	0	0
rxn03537	0	0
rxn03538	0	0
rxn03540	0	0
rxn03541	0	0
rxn03546	0	0
rxn03548	0	197,0130681
rxn03549	0	0
rxn03552	0	0
rxn03553	0	0
rxn03558	0	0
rxn03596	0	0
rxn03598	0	0
rxn03634	0	0
rxn03638	0,062989949	0,06298995
rxn03641	0,000690955	7,621066606
rxn03642	0,000690955	7,621066606
rxn03668	0	0

rxn03669	0	0
rxn03670	0	0
rxn03671	0	0
rxn03798	-1000	1000
rxn03838	0	0
rxn03845	0	0
rxn03856	0	0
rxn03885	0	0
rxn03891	0	0
rxn03900	0	0
rxn03901	0,031494975	0,031494975
rxn03902	0	0
rxn03903	0	0
rxn03904	0,031494975	0,031494975
rxn03907	0	0
rxn03908	0	0
rxn03909	0	0
rxn03910	0	0
rxn03919	0	0
rxn03933	0	0
rxn03952	0	0
rxn03958	0	0
rxn03962	0	0
rxn03963	-1000	0
rxn03964	0	1000
rxn03974	-0,028334856	0
rxn03975	-0,028334856	0
rxn04009	0	0
rxn04016	0	0
rxn04045	0	0
rxn04046	0	0
rxn04047	0	0
rxn04048	0	0
rxn04050	0	0
rxn04052	0	0
rxn04068	0	0
rxn04070	0	0
rxn04082	0	0,5
rxn04113	0	0
rxn04142	0	0
rxn04147	0	0
rxn04234	0	0
rxn04308	0	0
rxn04384	0	0
rxn04385	0	0
rxn04413	0	0
rxn04432	0	0
rxn04443	0	0
rxn04674	0	0
rxn04676	-0,268472286	1000

rxn04678	-1000	0,268472286
rxn04703	0	0
rxn04704	0	0
rxn04726	0	0
rxn04736	0	0
rxn04786	0,007702147	0,007702147
rxn04794	0	1000
rxn04822	0	0
rxn04865	0	0
rxn04866	0	0
rxn04943	0	0
rxn04954	-0,40566583	0
rxn04960	0	0
rxn05004	0	0
rxn05005	-1000	0
rxn05006	-1000	0
rxn05010	0	0
rxn05011	0	0
rxn05012	0	0
rxn05025	0	0
rxn05029	0	0
rxn05030	6,28141E-05	6,28141E-05
rxn05039	0	0
rxn05050	0	0
rxn05054	0	0
rxn05072	0	0
rxn05115	0	0
rxn05234	0	0
rxn05236	0	0
rxn05247	0	0
rxn05248	0	0
rxn05249	0	0
rxn05250	0	0
rxn05251	0	0
rxn05252	0	0
rxn05269	0	0
rxn05289	0	0
rxn05322	0	0
rxn05323	0	0
rxn05324	0	0
rxn05325	0	0
rxn05326	0	0
rxn05327	0	0
rxn05328	0	0
rxn05329	0	0
rxn05330	0	0
rxn05331	0	0
rxn05332	0	0
rxn05333	0	0
rxn05334	0	0

rxn05335	0	0
rxn05336	0	0
rxn05337	0	0
rxn05338	0	0
rxn05339	0	0
rxn05340	0	0
rxn05341	0	0
rxn05342	0	0
rxn05343	0	0
rxn05344	0	0
rxn05345	0	0
rxn05346	0	0
rxn05347	0	0
rxn05348	0	0
rxn05350	0	0
rxn05457	-1000	0
rxn05465	0	0
rxn05733	0	0
rxn05736	0	1000
rxn05740	-1000	1000
rxn05759	-0,5	0
rxn05760	-171,0752696	1000
rxn05762	0	0
rxn05763	0	0
rxn05778	0	0
rxn05779	0	0
rxn05794	-197,0130681	0
rxn05853	0	0
rxn05854	0	0
rxn05871	0	0
rxn05872	0	0
rxn05873	0	0
rxn05874	0	0
rxn05878	0	0
rxn05899	0	0
rxn05901	0	0
rxn05918	0	0
rxn05927	0	0
rxn05934	0	0
rxn05937	-1000	1000
rxn05938	-22,37277897	0
rxn05939	0,000579032	1000
rxn05940	-1000	1,521292828
rxn05957	0	1000
rxn05958	0	0
rxn05962	0	0
rxn05970	0	0
rxn05990	0	0
rxn05994	0	0
rxn06005	0	0

rxn06023	0	0
rxn06038	0	0
rxn06043	0	0
rxn06044	0	0
rxn06045	0	0
rxn06071	0,000509365	338,1179763
rxn06077	0	0
rxn06078	0	0
rxn06081	0	0
rxn06090	0	0
rxn06091	0	0
rxn06096	0	0
rxn06108	-197,0137591	-0,000690955
rxn06109	-7,621066606	-0,000690955
rxn06139	0	0
rxn06140	0	0
rxn06155	0	0
rxn06168	0	0
rxn06181	0	1000
rxn06182	0	1000
rxn06190	0	0
rxn06195	0	0
rxn06196	0	0
rxn06200	0	0
rxn06217	0	0
rxn06218	0	0
rxn06219	0	0
rxn06233	0	0
rxn06244	0	0
rxn06252	-1000	0
rxn06253	0	1000
rxn06280	0	0
rxn06281	0	0
rxn06285	0	0
rxn06293	0	0
rxn06298	0	0
rxn06299	0	0
rxn06300	0	0
rxn06316	0	0
rxn06328	0	0
rxn06348	0	0
rxn06373	0	0
rxn06376	0	0
rxn06403	0	0
rxn06432	0	0
rxn06434	0	0
rxn06435	0	0
rxn06437	0	0
rxn06438	0	0
rxn06439	0	0

rxn06440	0	0
rxn06441	0	0
rxn06443	0	0
rxn06444	0	0
rxn06445	0	0
rxn06446	0	0
rxn06447	0	0
rxn06448	0	0
rxn06449	0	0
rxn06485	0	0
rxn06493	0	0
rxn06538	0	0
rxn06556	0	0
rxn06581	0	0
rxn06584	0	0
rxn06591	0,00203746	0,00203746
rxn06614	0	0
rxn06624	0	0
rxn06648	0	0
rxn06663	0	0
rxn06664	0	0
rxn06671	0	0
rxn06672	0	1000
rxn06673	0	1000
rxn06694	0	0
rxn06701	0	0
rxn06726	0	0
rxn06729	0	0
rxn06737	0	0
rxn06751	0	0
rxn06768	0	0
rxn06798	0	0
rxn06799	0	0
rxn06820	0	0
rxn06823	0	0
rxn06831	0	0
rxn06850	0	0
rxn06864	0	0
rxn06865	0	0
rxn06874	0	0
rxn06882	0	0
rxn06883	0	0
rxn06887	0	0
rxn06889	0	197,0130681
rxn06890	0	0
rxn06926	0	0
rxn06936	0	0
rxn06937	0,00203746	0,00203746
rxn06947	0	0
rxn06958	-338,1179763	-0,000509365

rxn06966	0	0
rxn06979	0	0
rxn07056	0	0
rxn07059	0	0
rxn07099	0	0
rxn07177	0	0
rxn07199	0	0
rxn07251	0	0
rxn07267	0	0
rxn07292	0	0
rxn07312	0	0
rxn07332	0	0
rxn07335	0	0
rxn07439	0	0
rxn07441	0	999,968505
rxn07456	0	0,693876201
rxn07465	0,007702147	0,007702147
rxn07466	-1000	1000
rxn07484	0	0
rxn07485	0	0
rxn07489	0	0
rxn07492	0	0
rxn07573	0	0
rxn07577	0	0
rxn07578	0	0
rxn07579	0	0
rxn07584	0	0
rxn07585	0	0
rxn07586	0	0
rxn07587	0	0
rxn07804	0	0
rxn07807	0	0
rxn07832	0	0
rxn07846	0	0
rxn07849	0	0
rxn07987	0	0
rxn07989	0	0
rxn07991	0	0
rxn07992	0	0
rxn07993	0	0
rxn07994	0	0
rxn08025	0	0
rxn08035	0	0
rxn08038	0	0
rxn08043	0	1,521292828
rxn08067	-1000	1000
rxn08083	0	0
rxn08084	0	0
rxn08085	0	0
rxn08086	0	0

rxn08087	0	0
rxn08088	0	0
rxn08089	0	0
rxn08094	0	999,999421
rxn08114	0	0
rxn08126	0	0
rxn08127	0	0
rxn08128	0	0
rxn08129	0	0
rxn08171	0	0
rxn08180	0	0
rxn08194	-1000	1000
rxn08206	0	0
rxn08207	0	0
rxn08208	0	0
rxn08209	0	0
rxn08294	0	0
rxn08295	0	0
rxn08296	0	0
rxn08297	0	0
rxn08298	0	0
rxn08299	0	0
rxn08300	0	0
rxn08306	0	0
rxn08307	0	0
rxn08308	0	0
rxn08309	0	0
rxn08310	0	0
rxn08311	0	0
rxn08312	0	0
rxn08352	0	0
rxn08386	0	0
rxn08390	0	0
rxn08392	0	0
rxn08394	0	0
rxn08396	0	0
rxn08398	0	0
rxn08433	0	0
rxn08438	0	0
rxn08444	0	1000
rxn08448	0	0
rxn08449	0	0
rxn08451	0	0
rxn08453	0	0
rxn08454	0	1000
rxn08455	0	0
rxn08456	0	0
rxn08457	0	0
rxn08519	0,057583371	0,057583371
rxn08546	0	0

rxn08547	0	1000
rxn08548	0	0
rxn08549	0	0
rxn08550	0	0
rxn08551	0	0
rxn08552	0	0
rxn08571	0	1000
rxn08582	0	0,5
rxn08605	0	0
rxn08607	0	0
rxn08615	-1000	1000
rxn08647	0	0
rxn08668	0	0
rxn08669	0	0
rxn08764	0	1,521292828
rxn08796	0	0
rxn08797	0	1000
rxn08798	0	0
rxn08799	0	1000
rxn08800	0	0
rxn08801	0	1000
rxn08802	0	0
rxn08803	0	0
rxn08804	0	0
rxn08805	0	0
rxn08806	0	0
rxn08807	0	0
rxn08808	0	0
rxn08809	0	0
rxn08810	0	0
rxn08811	0	0
rxn08812	0	0
rxn08813	0	0
rxn08814	0	0
rxn08815	0	0
rxn08816	0	0
rxn08817	0	0
rxn08818	0	0
rxn08819	0	0
rxn08820	0	0
rxn08821	0	0
rxn08822	0	0
rxn08823	0	0
rxn08838	0	0
rxn08839	0	0
rxn08840	0	0
rxn08841	0	0
rxn08842	0	0
rxn08843	0	0
rxn08844	0	0

rxn08845	0	0
rxn08846	0	0
rxn08847	0	0
rxn08848	0	0
rxn08849	0	0
rxn08850	0	0
rxn08851	0	0
rxn08857	0	0
rxn08889	0,000768616	0,000768616
rxn08890	0,006222019	0,006222019
rxn08891	0,000768616	0,000768616
rxn08892	-999,9854054	1000
rxn08893	-999,9923184	999,993087
rxn08894	-999,9854054	1000
rxn08897	-0,006912974	-0,006912974
rxn08926	0,000690955	0,000690955
rxn08927	-999,9984639	999,9869415
rxn08928	-999,9854054	1000
rxn08929	0,00153609	0,00153609
rxn08930	0	0
rxn08958	0,000768616	0,000768616
rxn09010	0	0
rxn09016	0	999,7611923
rxn09108	0	0
rxn09109	0	0
rxn09110	0	0
rxn09111	0	0
rxn09112	0	0
rxn09113	0	0
rxn09114	0	0
rxn09176	-1000	1000
rxn09177	0	0,000657835
rxn09197	0	0
rxn09198	0	0
rxn09199	0	0
rxn09200	0	0
rxn09201	0	0
rxn09202	0	0
rxn09203	0	0
rxn09205	0	0
rxn09206	0	0
rxn09207	0	0
rxn09208	0	0
rxn09209	0	0
rxn09210	0	0
rxn09211	0	0
rxn09235	0,028334856	0,028334856
rxn09237	0,029248515	0,029248515
rxn09244	0	0
rxn09340	0	0

rxn09341	0	999,5995158
rxn09348	0	999,6310107
rxn09355	0	0
rxn09395	0	0
rxn09398	-999,6310107	0
rxn09399	0	0
rxn09412	-1000	1000
rxn09445	0	0
rxn09446	0	0
rxn09447	0	0
rxn09486	0	0
rxn09502	0	1000
rxn09531	0	0
rxn09557	0,000254683	197,0133228
rxn09616	0,000690955	0,000690955
rxn09631	0,000254683	0,000254683
rxn09632	0	999,9997453
rxn09633	0,000254683	0,000254683
rxn09888	0	0
rxn09889	0	0
rxn09952	0	0
rxn09978	0	0
rxn09979	0	0
rxn09988	0	0
rxn09992	0	0
rxn09995	0	0
rxn10003	0	0,000657835
rxn10019	0	0
rxn10020	0	0
rxn10021	0	0
rxn10052	-1000	1000
rxn10054	0	999,5995158
rxn10056	0	0,000510507
rxn10058	0	0,000510507
rxn10060	0	0,000510507
rxn10091	-1000	1000
rxn10110	0	0
rxn10111	0	0
rxn10192	0	0
rxn10194	0	0
rxn10196	0	0
rxn10202	0	1000
rxn10203	0	1000
rxn10204	0	1000
rxn10205	0	0
rxn10206	0	0
rxn10207	0	0
rxn10208	0	0
rxn10209	0	0
rxn10210	0	0

rxn10211	0	0
rxn10212	0	0
rxn10213	0	0
rxn10214	0	0
rxn10215	0	0
rxn10216	0	0
rxn10217	0	0
rxn10218	0	0
rxn10219	0	0
rxn10220	0	0
rxn10221	0	0
rxn10222	0	0
rxn10223	0	0
rxn10224	0	0
rxn10225	0	0
rxn10226	0	0
rxn10227	0	0
rxn10228	0	0
rxn10229	0	0
rxn10230	0	0
rxn10231	0	0
rxn10232	0	0
rxn10233	0	0
rxn10234	0	0
rxn10235	0	0
rxn10236	0	0
rxn10237	0	0
rxn10253	0	0
rxn10254	0	0
rxn10255	0	0
rxn10256	0	0
rxn10257	0	0
rxn10258	0	0
rxn10259	0	0
rxn10260	0	0
rxn10261	0	0
rxn10262	0	0
rxn10263	0	0
rxn10264	0	0
rxn10289	0	0
rxn10290	0	0
rxn10291	0	0
rxn10292	0	0
rxn10293	0	0
rxn10294	0	0
rxn10295	0	0
rxn10296	0	0
rxn10297	0	0
rxn10363	0	0
rxn10404	0	0

rxn10405	0	0
rxn10406	0	0
rxn10407	0	0
rxn10408	0	0
rxn10409	0	0
rxn10410	0	0
rxn10785	6,28141E-05	6,28141E-05
rxn10951	0	0,028334856
rxn11007	0,028334856	0,028334856
rxn11513	0	0
rxn11547	0	0
rxn11548	0	0
rxn11550	0	0
rxn11551	-1000	1000
rxn11552	-1000	1000
rxn11567	0	0
rxn11571	0	0
rxn11587	0	0
rxn11599	0	0
rxn11609	0	0
rxn11641	0	0
rxn11642	0	0
rxn11703	0	0
rxn11711	0	0
rxn11712	0	0
rxn11713	0	0
rxn11716	0	0
rxn11732	0	0
rxn11749	0	0
rxn11755	0	0
rxn11757	-999,9971425	0
rxn11759	0	999,9971425
rxn11760	-999,9971425	0
rxn11761	0	0
rxn11765	0	0
rxn11766	0	0
rxn11768	0	0
rxn11772	0	0
rxn11773	0	0
rxn11788	0	0
rxn11890	0	0
rxn11943	0	0
rxn11946	0	0
rxn11951	0	0
rxn11968	0	0
rxn12013	0	0
rxn12023	0	0
rxn12028	0	0
rxn12030	0	0
rxn12033	0	0

rxn12049	0	0
rxn12218	-1000	-0,000254683
rxn12221	0,000254683	1000
rxn12510	0,000657835	0,000657835
rxn12649	-999,9989813	0
rxn12767	0	0
rxn12768	0	0
rxn12769	0	0
rxn12770	0	0
rxn12771	0	0
rxn12778	0	0
rxn12822	-197,0130681	0
rxn13420	0,000690955	7,621066606
rxn13421	0,000690955	7,621066606
rxn13705	0	0
rxn13741	0	0
rxn13906	-0,007702147	-0,007702147
rxn13936	0,015363179	0,01536318
rxn13974	-22,37277897	0
rxn14029	0	0
rxn14043	0	0
rxn14050	0	0
rxn14054	-197,0130681	0
rxn14063	0	0
rxn14070	0	0
rxn14089	-197,0130681	0
rxn14093	0	0
rxn14120	-1000	-0,00101873
rxn14132	0	0
rxn14136	0	0
rxn14219	0	0
rxn14250	0	0
rxn14270	0	0
rxn14279	0	0
rxn14346	0	0
rxn90002	-13,96688252	1000
rxn90003	0	0
rxn90004	0	0
rxn90005	-0,028845363	-0,028334856
rxn08173	0	98,50653405
Biomass_Bacteria	1,142074	1,142074006
t_Cl	0,005153038	0,005153038
t_Sulfate	0,004294198	0,004294198
t_Cu2+	0,003435359	0,003435359
t_Mg	0,008587254	0,008587254
t_Ca2+	0,005153038	0,005153038
t_NH3	0	0
t_H2O	-23,8980203	9,527467307
t_Biomass	-1,142074006	-1,142074
t_Butyrate	0	0

t_D-Lactate	-14,91518598	0
t_Ethanol	-1,302559388	0
t_Formate	-15,2407513	0
t_H2	0	0,5
t_L-Lactate	-14,91518598	0
t_Nitrite	0	0
t_Phosphate	1,517653029	2,011966651
t_Propionate	-1,521292828	0
t_O2	0	0
t_D-Glucose	0	0,5
t_CO2	-15,2407513	0
t_Acetate	-22,75116065	0
t_Succinate	-7,620375652	0
t_H2S	-0,397709	0
Ex_Cl	-0,005153038	-0,005153038
Ex_Sulfate	-0,004294198	-0,004294198
Ex_Cu2+	-0,003435359	-0,003435359
Ex_Mg	-0,008587254	-0,008587254
Ex_Ca2+	-0,005153038	-0,005153038
Ex_NH3	0	0
Ex_H2O	-9,527467307	23,8980203
Ex_Biomass	1,142074	1,142074006
Ex_Butyrate	0	0
Ex_D-Lactate	0	14,91518598
Ex_Ethanol	0	1,302559388
Ex_Formate	0	15,2407513
Ex_H2	-0,5	0
Ex_L-Lactate	0	14,91518598
Ex_Nitrite	0	0
Ex_Phosphate	-2,011966651	-1,517653029
Ex_Propionate	0	1,521292828
Ex_O2	0	0
Ex_D-Glucose	-0,5	0
Ex_CO2	0	15,2407513
Ex_Acetate	0	22,75116065
Ex_Succinate	0	7,620375652
Ex_H2S	0	0,397709
t_Fe2	0,007983097	0,007983097
t_fe3	0,007728415	0,007728415
t_Acetaldehyde	-1,302559388	0
t_Adenosine	0	0,494313614
t_AMP	0	0,494313614
t_Amylotriose	0	0
t_BIOT	0	0
t_Choline	0	0
t_Cytidine	0	0
t_Cytosine	0	0
t_DAlanine	0	0
t_Deoxyadenosine	0	0,494313614
t_Deoxycytidine	0	0,365829146

t_Deoxyguanosine	0	0
t_Deoxyinosine	0	0
t_Deoxyuridine	0	0
t_DRibose	0	0,5
t_DSerine	0	0
t_GLUM	0	0
t_Glycerol	0	0
t_GSH	0	0
t_Guanine	0	0
t_H2S2O3	0	0
t_Heme	0,000254683	0,000254683
t_Homocysteine	0	0
t_HYXN	0	0,494313614
t_Inosine	0	0,494313614
t_LACT	0	0,5
t_LAlanine	-1,481766811	0,5
t_LArabinose	0	0,5
t_LArginine	-0,122533698	0,5
t_LAsparagine	-0,193876201	0,5
t_LAspartate	-0,193876201	0,5
t_LCysteine	0,102291	0,5
t_LGlutamate	-0,702244183	0,5
t_LGlutamine	-0,702244183	0,5
t_LHistidine	0,105185015	0,105185016
t_LInositol	0	0
t_LIsoleucine	-1,198885336	0,322407492
t_LLeucine	0,499999997	0,5
t_LLysine	-0,313108729	0,380767474
t_LMethionine	-0,226433304	0,171275697
t_LPhenylalanine	-1,776193491	0,205573321
t_LThreonine	-1,021038145	0,5
t_LTryptophan	-0,927806658	0,063076747
t_LTyrosine	-1,828728895	0,153037917
t_LValine	-1,51134653	0,470420283
t_Maltose	0	0,5
t_Niacin	0	0,002602787
t_Ornithine	0	0
t_PPi	0	0
t_Pyridoxol	0	0
t_XAN	0	0
t_5Deoxyadenosine	0	0
t_Acetoacetate	-7,620375652	0
t_BET	0	0
t_Calomide	0	0
t_Carnosine	0	0
t_Cbl	0	0
t_Citrate	0	0
t_CysGly	0	0
t_Dulcose	0	0
t_Glycine	-1,481766811	0,5

t_Glycolaldehyde	0	0
t_LProline	0,245317495	0,245317497
t_Maltohexaose	0	0
t_Methanol	0	0
t_NAcetylDglucosamine	0	0
t_PM	0	0
t_Putrescine	0	0
t_Pyridoxal	0,000254683	0,000254683
t_Riboflavin	0,000509365	0,000509365
t_Sorbitol	0	0
t_Spermidine	0	0
t_Sucrose	0	0,5
t_Taurine	0	0
t_Thiamin	0	0
t_Thyminose	0	0,5
t_TRHL	0	0
t_Uracil	0	0,365829146
t_Uridine	0	0,365829146
t_Mn2+	0,003435359	0,003435359
t_Fumarate	-7,620375652	0
t_Oxidized glutathione	0	0
t_Adenine	0	0
t_Nicotinamide	0	0
t_4-Hydroxybenzoate	0	0
t_Co2+	0,003435359	0,003435359
t_D-Arabinose	0	0,5
t_D-Glutamate	0	0
t_Chorismate	0	0
t_Folate	0,00101873	0,00101873
t_N-Acetyl-D-mannosamine	0	0
t_Siroheme	0	0
t_Menaquinone 7	0	0
t_2-Demethylmenaquinone 8	0	0
t_Menaquinone 8	0	0
t_Ubiquinone-8	0	0
t_2-Oxobutyrate	0	0
t_3MOP	0	0
t_ABEE	0	0
t_Neu5Ac	0	0
t_Glycerol-3-phosphate	0	0
t_H+	-1000	0,5
t_indol	0	0
t_Nicotinamide ribonucleotide	0	0
t_PAN	0,000657835	0,000657835
t_Pyridoxal phosphate	0	0
t_Zn2+	0,003435359	0,003435359
t_1,2-Diacyl-sn-glycerol dioctadecanoyl	0	0
t_meso-2,6-Diaminopimelate	0	0
t_L-Serine	-1,481766811	0,5
t_D-Fructose	0	0,5

t_D-Mannose	0	0
t_beta D-Galactose	0	0,5
t_L-Fucose	0	0
Ex_Fe2	-0,007983097	-0,007983097
Ex_fe3	-0,007728415	-0,007728415
Ex_Acetaldehyde	0	1,302559388
Ex_Adenosine	-0,494313614	0
Ex_AMP	-0,494313614	0
Ex_Amylotriose	0	0
Ex_BIOT	0	0
Ex_Choline	0	0
Ex_Cytidine	0	0
Ex_Cytosine	0	0
Ex_DAlanine	0	0
Ex_Deoxyadenosine	-0,494313614	0
Ex_Deoxycytidine	-0,365829146	0
Ex_Deoxyguanosine	0	0
Ex_Deoxyinosine	0	0
Ex_Deoxyuridine	0	0
Ex_DRibose	-0,5	0
Ex_DSerine	0	0
Ex_GLUM	0	0
Ex_Glycerol	0	0
Ex_GSH	0	0
Ex_Guanine	0	0
Ex_Heme	-0,000254683	-0,000254683
Ex_Homocysteine	0	0
Ex_HYXN	-0,494313614	0
Ex_Inosine	-0,494313614	0
Ex_LACT	-0,5	0
Ex_LAlanine	-0,5	1,481766811
Ex_LArabinose	-0,5	0
Ex_LArginine	-0,5	0,122533698
Ex_LAsparagine	-0,5	0,193876201
Ex_LAspartate	-0,5	0,193876201
Ex_LCysteine	-0,5	-0,102291
Ex_LGlutamate	-0,5	0,702244183
Ex_LGlutamine	-0,5	0,702244183
Ex_LHistidine	-0,105185016	-0,105185015
Ex_LInositol	0	0
Ex_LIsoleucine	-0,322407492	1,198885336
Ex_LLeucine	-0,5	-0,499999997
Ex_LLysine	-0,380767474	0,313108729
Ex_LMethionine	-0,171275697	0,226433304
Ex_LPhenylalanine	-0,205573321	1,776193491
Ex_LThreonine	-0,5	1,021038145
Ex_LTryptophan	-0,063076747	0,927806658
Ex_LTyrosine	-0,153037917	1,828728895
Ex_LValine	-0,470420283	1,51134653
Ex_Maltose	-0,5	0

Ex_Niacin	-0,002602787	0
Ex_Ornithine	0	0
Ex_PPi	0	0
Ex_XAN	0	0
Ex_5Deoxyadenosine	0	0
Ex_Acetoacetate	0	7,620375652
Ex_BET	0	0
Ex_Calomide	0	0
Ex_Carnosine	0	0
Ex_Cbl	0	0
Ex_Citrate	0	0
Ex_CysGly	0	0
Ex_Dulcose	0	0
Ex_Glycine	-0,5	1,481766811
Ex_Glycolaldehyde	0	0
Ex_LProline	-0,245317497	-0,245317495
Ex_Maltohexaose	0	0
Ex_Methanol	0	0
Ex_NAcetylDglucosamine	0	0
Ex_PM	0	0
Ex_Putrescine	0	0
Ex_Pyridoxal	-0,000254683	-0,000254683
Ex_Riboflavin	-0,000509365	-0,000509365
Ex_Sorbitol	0	0
Ex_Spermidine	0	0
Ex_Sucrose	-0,5	0
Ex_Taurine	0	0
Ex_Thiamin	0	0
Ex_Thyminose	-0,5	0
Ex_TRHL	0	0
Ex_Uracil	-0,365829146	0
Ex_Uridine	-0,365829146	0
Ex_Mn2+	-0,003435359	-0,003435359
Ex_Fumarate	0	7,620375652
Ex_Oxidized glutathione	0	0
Ex_Adenine	0	0
Ex_Nicotinamide	0	0
Ex_4-Hydroxybenzoate	0	0
Ex_Co2+	-0,003435359	-0,003435359
Ex_D-Arabinose	-0,5	0
Ex_D-Glutamate	0	0
Ex_Folate	-0,00101873	-0,00101873
Ex_N-Acetyl-D-mannosamine	0	0
Ex_Siroheme	0	0
Ex_Menaquinone 7	0	0
Ex_2-Demethylmenaquinone 8	0	0
Ex_Menaquinone 8	0	0
Ex_Ubiquinone-8	0	0
Ex_ABEE	0	0
Ex_Neu5Ac	0	0

Ex_H+	-0,5	1000
Ex_indol	0	0
Ex_Nicotinamide ribonucleotide	0	0
Ex_PAN	-0,000657835	-0,000657835
Ex_Zn2+	-0,003435359	-0,003435359
Ex_1,2-Diacyl-sn-glycerol dioctadecanoyl	0	0
Ex_L-Serine	-0,5	1,481766811
Ex_D-Fructose	-0,5	0
Ex_D-Mannose	0	0
Ex_beta D-Galactose	-0,5	0
Ex_L-Fucose	0	0
t_Arabinan	0	0
t_Starch	0	0,005
t_octanoate	0	0
t_Melibiose	0	0,5
t_Linolenate	0	0
t_Amylose	0	0
t_Linoleate	0	0
Ex_Arabinan	0	0
Ex_Starch	-0,005	0
Ex_Melibiose	-0,5	0
Ex_Linolenate	0	0
Ex_Amylose	0	0
Ex_Linoleate	0	0
t_Raffinose_Melitose	0	0
t_Isovaleric_acid	0	0
t_H2O2	0	0
Ex_Raffinose_Melitose	0	0
Ex_Isovaleric_acid	0	0
Ex_H2O2	0	0
rxn01207_1	0	0
rxn08972	0	0
rxn08973	0	0
rxn06111	0	197,0130681
rxn13726	0	0
rxn13727	0	0
rxn13729	0	0
rxn08974	0	0
rxn10122	0	0
rxn10123	0	0
rxn10124	0	0
rxn12665	0	0
rxn06097	0	0,005
t_Sulfite	0	0
Ex_Sulfite	0	0
t_Nitrogen	0	0
Ex_Nitrogen	0	0

rxn ID	minFlux	max Flux
rxn00001	0	1000
rxn00003	-2,382143703	0
rxn00011	-2,382143703	0
rxn00016	0	0
rxn00020	0	1000
rxn00022	0	0,005
rxn00029	0,00101873	0,00101873
rxn00031	0	0
rxn00056	0	2,128348661
rxn00060	0,000254683	0,000254683
rxn00062	0	1000
rxn00065	0	0
rxn00067	0	0
rxn00070	0	0
rxn00076	0	1000
rxn00077	0	0,000510507
rxn00085	-1000	0
rxn00086	0	0
rxn00097	-1000	1000
rxn00100	0,000657835	0,000657835
rxn00102	-1000	1000
rxn00103	0	1000
rxn00104	-1000	0
rxn00105	-999,9973972	1000
rxn00106	-1000	0
rxn00109	0	0
rxn00114	-1000	6,40968052
rxn00119	0,368989262	1000
rxn00121	-0,000254683	0
rxn00122	0	0,000254683
rxn00124	0,000254683	0,000254683
rxn00126	0,008466195	0,008466195
rxn00127	0,007702147	0,007702147
rxn00131	-1000	999,988931
rxn00132	0	1000
rxn00137	0	0
rxn00138	0	1000
rxn00139	-999,9971425	0
rxn00141	-999,9994906	0
rxn00142	0	0
rxn00143	0,000509365	1000
rxn00147	0	1000
rxn00148	-1000	0
rxn00151	-1000	0
rxn00154	0	507,9682483
rxn00157	-507,9682483	0
rxn00159	-1000	1000
rxn00161	-1000	1000
rxn00162	0	1000

rxn00165	0	500,6370696
rxn00171	0	0
rxn00173	0	1000
rxn00179	0	0
rxn00184	-1000	0
rxn00187	0	1000
rxn00189	0	1000
rxn00190	0	1000
rxn00191	-1000	999,8707641
rxn00192	0	1000
rxn00193	0,031494975	0,031494975
rxn00196	0	0
rxn00198	0	1000
rxn00199	0	1000
rxn00206	0,000254683	171,6824771
rxn00211	0	0
rxn00213	-1000	999,5995158
rxn00214	-1,5	0
rxn00216	0	1000
rxn00221	0	1000
rxn00222	0	1000
rxn00224	0,000254683	1000
rxn00225	-1000	0
rxn00227	0	1000
rxn00231	0	0
rxn00239	0,238807673	1000
rxn00242	0	1000
rxn00245	0	0
rxn00247	0	1000
rxn00248	-1000	1000
rxn00250	-1000	1000
rxn00256	-506,7538956	0
rxn00258	-1000	1000
rxn00260	-1000	1,264883513
rxn00262	0	171,6822225
rxn00273	0	0
rxn00274	0	0
rxn00275	-0,962864713	-0,000254683
rxn00283	0,027731841	0,027731841
rxn00285	-1000	1000
rxn00293	0,062989949	0,06298995
rxn00297	0	0
rxn00299	0	0,962610031
rxn00301	0	999,7611923
rxn00302	0	0,962610031
rxn00303	0	1000
rxn00304	-1000	0
rxn00307	0	0
rxn00313	0	1,191071851
rxn00322	0	0

rxn00324	-171,6822225	0
rxn00328	0	0
rxn00333	0,000254683	171,6824771
rxn00337	0,031494975	1,222566826
rxn00338	0	0,002602787
rxn00340	0	1000
rxn00342	0	1000
rxn00346	0	0
rxn00350	-0,000254683	-0,000254683
rxn00358	0	0
rxn00360	0	1000
rxn00361	0	1000
rxn00362	-1000	1000
rxn00363	0	1000
rxn00364	-999,6310107	999,999309
rxn00365	0	1000
rxn00368	0	1000
rxn00369	0	1000
rxn00371	0	1000
rxn00379	0	0
rxn00391	0	999,9997453
rxn00392	0,000254683	1000
rxn00405	0	6,581448453
rxn00410	-999,8225103	999,8085005
rxn00411	-1000	0
rxn00412	0	1000
rxn00414	0	1000
rxn00416	0	1000
rxn00420	0	0
rxn00423	0	500,6370696
rxn00426	0	0
rxn00436	0	999,9997453
rxn00437	0	0
rxn00440	0,000254683	1000
rxn00453	0	1000
rxn00456	0	1000
rxn00459	-1,601658954	6,8451321
rxn00460	-1000	0
rxn00461	0,031494975	0,031494975
rxn00469	0	1000
rxn00470	0	6,385045984
rxn00474	0	0
rxn00490	0	2,382143703
rxn00493	-2,382143703	0
rxn00499	-8,513394645	0
rxn00500	-8,513394645	0
rxn00506	0	0
rxn00510	0	0
rxn00512	-171,6822225	0
rxn00514	0	0

rxn00517	-1000	0
rxn00527	-2,382143703	0
rxn00533	-1000	1000
rxn00536	-1000	1000
rxn00541	0	0
rxn00543	-1000	1000
rxn00545	0	1000
rxn00547	0	1000
rxn00549	0	1000
rxn00551	0	1000
rxn00552	-0,06298995	999,9370101
rxn00554	0	1000
rxn00555	0	1000
rxn00556	0	1000
rxn00557	0	1000
rxn00558	-1000	1000
rxn00559	0	0
rxn00560	0	0
rxn00565	0	0
rxn00566	0	1000
rxn00567	-17,09248139	-0,065692096
rxn00575	0	1000
rxn00577	-1000	0
rxn00585	0	0
rxn00598	-0,000254683	-0,000254683
rxn00609	0	0
rxn00611	-8,829564893	0
rxn00615	0	8,829564893
rxn00616	0	8,829564893
rxn00621	0	0
rxn00622	0	0
rxn00634	0	1000
rxn00642	0	0
rxn00647	0	0
rxn00649	0	500,6370696
rxn00650	-0,000254683	-0,000254683
rxn00653	0	0
rxn00654	0	0
rxn00670	0	1000
rxn00673	-8,513394645	0
rxn00677	-1000	1000
rxn00684	-0,962610031	0
rxn00685	0	1000
rxn00686	-0,962610031	0
rxn00687	0	1000
rxn00689	0	0
rxn00690	0	1000
rxn00692	-0,260507082	500,3765626
rxn00693	0	0,497709
rxn00695	-1000	1000

rxn00698	-1000	0
rxn00701	0	1,5
rxn00702	0	0
rxn00704	-1000	1,5
rxn00707	0	1000
rxn00708	0	1000
rxn00709	0	1000
rxn00710	0	0,365829146
rxn00711	-999,9971425	0
rxn00712	0	1000
rxn00713	0	1000
rxn00715	0	1000
rxn00726	0	1,191071851
rxn00727	0	1,191071851
rxn00729	0	0
rxn00735	0	0
rxn00737	0	0,218478759
rxn00740	0	1000
rxn00741	0	0
rxn00742	-1000	0,000509365
rxn00743	0	1000
rxn00745	0	1000
rxn00747	-3,33160766	0,657198185
rxn00748	0	0
rxn00758	0	0
rxn00762	-8,829564893	0
rxn00763	0	0
rxn00764	-8,829564893	0
rxn00765	0	0
rxn00770	0,002857469	1000
rxn00772	0	1000
rxn00775	0	0
rxn00777	-1,486079273	0,364597609
rxn00778	-1000	1000
rxn00781	-1000	1000
rxn00782	-1000	1000
rxn00785	-0,182171463	1,683664762
rxn00786	-1000	3,33160766
rxn00787	0	0
rxn00789	0	0
rxn00790	-0,000254683	-0,000254683
rxn00791	-1,191071851	0
rxn00792	0	0
rxn00796	0	0
rxn00799	-1000	4,916242969
rxn00800	-0,831325949	863,0469246
rxn00802	0	6,385045984
rxn00806	0	0
rxn00808	0	1000
rxn00809	-1000	0

rxn00811	-1000	0
rxn00814	0	0
rxn00815	0	0
rxn00816	0	0,5
rxn00817	0	0,5
rxn00818	0	0
rxn00819	0	0
rxn00827	0	0
rxn00829	0,000690955	0,000690955
rxn00830	6,28141E-05	6,28141E-05
rxn00831	0	999,9971425
rxn00832	0	0
rxn00834	-999,7290863	1000
rxn00836	-999,9971425	0
rxn00838	-0,831325949	863,0469246
rxn00851	0	1000
rxn00853	0	6,581448453
rxn00855	0	0
rxn00856	0,000690955	6,535749504
rxn00858	0	0
rxn00869	0	0
rxn00872	-6,535058549	0
rxn00875	0	6,535058549
rxn00879	0	0
rxn00881	0	0
rxn00882	0	0
rxn00883	0	0
rxn00889	0	0
rxn00890	0	0
rxn00898	0	2,382143703
rxn00902	0	0
rxn00903	-1000	999,8707641
rxn00904	-999,8707641	1000
rxn00907	-999,9994906	0,000509365
rxn00908	-1000	1000
rxn00909	-8,66340721	0,497963683
rxn00910	-8,829564893	0
rxn00913	0	1000
rxn00915	-999,9971425	0
rxn00916	-999,7319438	1000
rxn00917	0	1000
rxn00921	0	0
rxn00926	0	863,3149808
rxn00927	-1000	1000
rxn00929	-1000	1000
rxn00931	-1000	1000
rxn00942	0	1000
rxn00943	0	1000
rxn00946	0	0
rxn00947	0	1000

rxn00950	-1000	0,497199635
rxn00952	0	1000
rxn00955	0,000509365	1000
rxn00957	0,000254683	0,000254683
rxn00962	0,000254683	0,000254683
rxn00973	-1000	1000
rxn00974	-1000	1000
rxn00977	0	0
rxn00979	0,000254683	0,962864713
rxn00980	0	0
rxn00983	0	0
rxn00985	-1000	0
rxn00990	0,000690955	6,535749504
rxn00991	-0,000690955	-0,000690955
rxn01000	0	2,382143703
rxn01008	0	0
rxn01011	0	0
rxn01013	0	0
rxn01016	0	0
rxn01018	0	0,365829146
rxn01019	0,024634536	6,40968052
rxn01021	0	0
rxn01022	0,007702147	0,007702147
rxn01029	0	6,581448453
rxn01034	0	0
rxn01037	0	0
rxn01042	0	0
rxn01056	-1000	1000
rxn01069	0	0
rxn01073	0	0
rxn01080	0	0
rxn01089	0	0
rxn01100	-1000	0
rxn01101	0	0
rxn01103	0	1000
rxn01106	-6,8451321	1,601658954
rxn01115	0	0
rxn01116	-1,486079273	0,364597609
rxn01119	0	0
rxn01123	0	0
rxn01124	0	0
rxn01133	0	0
rxn01134	0	0
rxn01138	-1000	1000
rxn01139	0	0
rxn01146	0	0
rxn01169	0	1000
rxn01171	-1000	1000
rxn01192	0,000254683	0,000254683
rxn01199	0	0

rxn01200	0	1000
rxn01201	-6,535749504	-0,000690955
rxn01204	0,000690955	6,535749504
rxn01210	0	0
rxn01211	-999,9997453	0,000509365
rxn01213	6,28141E-05	6,28141E-05
rxn01225	0	999,9971425
rxn01226	-999,9707515	1000
rxn01228	0	0
rxn01237	0	0
rxn01241	-1000	1000
rxn01249	0	0
rxn01255	0,000254683	2,382398385
rxn01256	0	2,382143703
rxn01257	0	0,962610031
rxn01258	0	0
rxn01259	0	0
rxn01265	-999,9997453	0
rxn01268	0	2,382143703
rxn01270	-2,382143703	0
rxn01274	0	0
rxn01275	0	0
rxn01276	0	0
rxn01278	0	0
rxn01280	0	0
rxn01281	0	0
rxn01286	0	0
rxn01290	0	0
rxn01291	0	0
rxn01292	0	0
rxn01297	-999,7319438	999,9971425
rxn01299	-1000	1000
rxn01300	0	0
rxn01303	0	0
rxn01304	0	0
rxn01305	0	0
rxn01310	-1000	0
rxn01321	0	0
rxn01332	0,000254683	2,382398385
rxn01333	-1000	0,95727959
rxn01334	0	1000
rxn01343	0	1000
rxn01344	0	0
rxn01346	0	1000
rxn01347	0	1000
rxn01348	0	1000
rxn01351	0	1000
rxn01352	-1000	-0,029248515
rxn01354	-1000	0
rxn01355	0	0

rxn01358	-1000	1000
rxn01360	0	171,6822225
rxn01361	-171,6822225	0
rxn01362	-0,365829146	0
rxn01366	-0,33749429	1000
rxn01367	0	0
rxn01368	0	999,6310107
rxn01370	0	1000
rxn01380	0	0
rxn01387	-1000	0
rxn01388	-1000	1000
rxn01396	0	0
rxn01406	0,007702147	0,007702147
rxn01423	0	0
rxn01426	0	0
rxn01434	0	6,385045984
rxn01435	0	0
rxn01437	0	0
rxn01445	0	999,9707515
rxn01446	-0,029248515	-0,029248515
rxn01457	0	0
rxn01459	0	6,535749504
rxn01465	-0,365829146	0
rxn01466	6,28141E-05	6,28141E-05
rxn01478	0	0
rxn01480	0	0
rxn01484	0	0
rxn01485	-0,06298995	-0,062989949
rxn01486	0	0
rxn01492	0	0
rxn01500	-0,000690955	-0,000690955
rxn01506	0	0
rxn01509	-999,9707515	1000
rxn01510	0	1000
rxn01513	0,028334856	0,028334856
rxn01518	0,028334856	1000
rxn01519	0	0
rxn01521	0	999,9716651
rxn01522	0	0
rxn01530	0	0
rxn01539	-1000	-0,000254683
rxn01541	-1000	1000
rxn01544	-999,9971425	0
rxn01545	-1000	1000
rxn01548	-999,9707515	1000
rxn01549	0	0
rxn01562	0	0
rxn01563	0	1000
rxn01575	-2,382143703	0
rxn01594	0	0

rxn01601	0	0,962610031
rxn01602	0	0,962610031
rxn01603	0	0,962610031
rxn01610	0	0
rxn01615	0	0
rxn01629	-0,00203746	-0,00203746
rxn01636	-999,9753655	6,40968052
rxn01637	-6,40968052	-0,024634536
rxn01642	0	0
rxn01643	-1,222566826	-0,031494975
rxn01644	0,031494975	1,222566826
rxn01646	-1000	0
rxn01647	0	999,9971425
rxn01648	-1000	1000
rxn01649	-1000	1000
rxn01650	-1000	0
rxn01652	0	1000
rxn01653	0	1000
rxn01667	0	0
rxn01669	0	999,9973972
rxn01670	0	0
rxn01675	0	0
rxn01679	0	0
rxn01682	0	0
rxn01683	-1000	1000
rxn01684	-1000	1000
rxn01686	0	0
rxn01704	0	0
rxn01706	0	0
rxn01710	0	0
rxn01731	-1000	1000
rxn01734	-1000	1000
rxn01735	0	0
rxn01737	0	0
rxn01739	0,000254683	2,382398385
rxn01740	-2,382398385	-0,000254683
rxn01741	0	0
rxn01748	0	0
rxn01757	0	0
rxn01775	0	0
rxn01790	0	0
rxn01791	0	0
rxn01799	-0,028334856	0,029248515
rxn01800	0	0,057583371
rxn01807	0	0
rxn01816	0	1000
rxn01831	0	0
rxn01834	0	0
rxn01835	0	0
rxn01842	0	0

rxn01843	0	0
rxn01851	0	6,535749504
rxn01857	0	0
rxn01859	0	0,057583371
rxn01860	0	0
rxn01870	0	0
rxn01871	0	0
rxn01879	0	0
rxn01885	0	0
rxn01892	0	0
rxn01906	0	0
rxn01917	0,024634536	6,40968052
rxn01937	0	0
rxn01946	0	0
rxn01953	0	0
rxn01961	0	999,9971425
rxn01962	0	0
rxn01964	0	1,191071851
rxn01967	0	0
rxn01972	0,031494975	1000
rxn01973	0	0
rxn01974	0,031494975	1,222566826
rxn01977	-1000	1000
rxn01981	0	0
rxn01982	0	0
rxn01985	0	0
rxn01986	-0,057583371	0
rxn01987	-0,057583371	0
rxn01991	0	0
rxn01996	0	0
rxn01997	0	0
rxn01998	0	0
rxn01999	0	0
rxn02000	0	0
rxn02003	0	0
rxn02008	0,031494975	0,031494975
rxn02009	0	0
rxn02010	0	0
rxn02011	0,031494975	0,031494975
rxn02015	0	0
rxn02021	0	0
rxn02023	0	0
rxn02033	0	0
rxn02046	0	0
rxn02056	0	999,9997453
rxn02061	0	0
rxn02093	0	0
rxn02102	-1000	0
rxn02103	0	1000
rxn02106	0	0

rxn02112	0	0
rxn02118	0	0
rxn02122	0	0
rxn02123	0	8,513394645
rxn02128	0	0
rxn02138	0	0
rxn02139	0	0
rxn02143	0,000254683	0,000254683
rxn02144	0,000254683	0,000254683
rxn02154	0	999,9973972
rxn02155	0,002602787	1000
rxn02160	0	0
rxn02167	0	0
rxn02171	0,000690955	6,535749504
rxn02175	0,000657835	0,000657835
rxn02185	-2,382143703	2,382143703
rxn02186	0	2,382143703
rxn02187	0	0
rxn02195	0	0
rxn02199	0	0
rxn02200	0	0,962610031
rxn02201	0	0,962610031
rxn02202	0	0
rxn02203	0	0
rxn02209	0	0
rxn02212	0,000254683	2,382398385
rxn02213	0,000254683	2,382398385
rxn02222	0	0
rxn02228	0	0
rxn02235	0	0
rxn02236	0	0
rxn02246	0	0
rxn02264	0,000254683	0,000254683
rxn02275	0	0
rxn02281	0	0
rxn02284	-0,031494975	0
rxn02285	-0,031494975	0
rxn02286	0,031494975	0,031494975
rxn02287	-999,9997453	1000
rxn02288	0	0
rxn02302	-1000	0
rxn02304	0	0
rxn02305	0,000254683	0,000254683
rxn02314	0	1000
rxn02315	0	1000
rxn02316	0	1000
rxn02317	-1000	0
rxn02320	0	0
rxn02322	0,000690955	0,000690955
rxn02339	0	0

rxn02341	0,000657835	0,000657835
rxn02350	0	0
rxn02351	0	0
rxn02356	-1000	1000
rxn02358	-1000	1000
rxn02369	-0,000254683	-0,000254683
rxn02373	-1000	1000
rxn02375	0	0
rxn02380	-1000	1000
rxn02400	0	999,9971425
rxn02402	-0,002602787	0
rxn02409	0	0
rxn02415	0	0
rxn02432	0	0
rxn02433	0	0
rxn02449	0	1000
rxn02452	0	0
rxn02454	0	0
rxn02465	-6,40968052	-0,024634536
rxn02473	0	0
rxn02474	0	0
rxn02475	0	0
rxn02476	0,000254683	2,382398385
rxn02483	0,000254683	0,000254683
rxn02484	0,000254683	0,000254683
rxn02495	0	0
rxn02503	0	0,962610031
rxn02504	0	0,962610031
rxn02507	0	1,191071851
rxn02508	0	1,191071851
rxn02518	0	0
rxn02521	0	0
rxn02522	0	0
rxn02571	0	0
rxn02581	0	0
rxn02596	0	0
rxn02597	0	0
rxn02632	0	0
rxn02679	0	0
rxn02720	0	0
rxn02727	0	0
rxn02729	0	0
rxn02749	0	0
rxn02751	0	0
rxn02760	0	0
rxn02762	0	0
rxn02774	-999,9997453	0
rxn02775	0	0
rxn02776	0	0
rxn02789	0	0

rxn02791	0	0
rxn02792	0	0
rxn02795	0	0
rxn02796	0	0
rxn02803	0	0
rxn02811	0	0
rxn02821	0	0
rxn02822	0	0
rxn02830	0	0
rxn02834	0	0
rxn02835	0	0
rxn02853	0	0
rxn02866	0	0
rxn02875	0	0
rxn02895	0,000254683	0,000254683
rxn02897	0	0
rxn02900	0	0
rxn02912	0	0
rxn02914	0	0
rxn02922	0	0
rxn02928	-1000	999,968505
rxn02929	-1000	999,968505
rxn02931	0	0
rxn02936	0	0
rxn02937	0,000254683	0,000254683
rxn02943	0	0
rxn02986	0	0
rxn02988	-0,002602787	0
rxn02990	0	0
rxn03004	0	0,000254683
rxn03005	-0,000254683	0
rxn03030	0,031494975	1000
rxn03047	0	0
rxn03052	0	0
rxn03057	0,007702147	0,007702147
rxn03062	0	0
rxn03066	0	0
rxn03068	0	0
rxn03075	0,000254683	0,000254683
rxn03084	0,000254683	0,000254683
rxn03086	-1000	-0,031494975
rxn03087	0	0
rxn03094	0	0
rxn03095	0	0
rxn03102	0	0
rxn03106	0	0
rxn03108	0,000254683	0,000254683
rxn03135	0	0
rxn03136	0	0
rxn03137	0	0

rxn03140	0	0
rxn03141	0	0
rxn03146	0	0
rxn03147	0	0
rxn03150	0	0
rxn03164	0,031494975	0,031494975
rxn03167	0	0,962610031
rxn03174	-0,962610031	0
rxn03175	0	0
rxn03181	0	0
rxn03188	0	0
rxn03194	0	2,382143703
rxn03224	0	0
rxn03251	0	0
rxn03253	0	0
rxn03263	0	0
rxn03264	0	0
rxn03269	0	0
rxn03273	0	0
rxn03282	0	0
rxn03333	0	0
rxn03354	0	0
rxn03362	0	0
rxn03372	0	0
rxn03373	0	0
rxn03374	0	0
rxn03378	0	0
rxn03379	0	0
rxn03382	0	0
rxn03383	0	0
rxn03384	0	0
rxn03387	0	0
rxn03393	0	0
rxn03395	0	0
rxn03397	0	0
rxn03402	0	0
rxn03405	0	0
rxn03406	0	0
rxn03407	0	0
rxn03408	0,031494975	0,031494975
rxn03409	0	0
rxn03419	0	0,962610031
rxn03421	0	0,962610031
rxn03423	0	0
rxn03435	-2,382143703	0
rxn03436	0	2,382143703
rxn03437	0	2,382143703
rxn03445	0	0
rxn03446	0	0
rxn03462	0	0

rxn03465	0	0
rxn03467	0	0
rxn03468	0	0
rxn03483	0	0
rxn03491	0	0
rxn03492	0	0
rxn03512	0	0
rxn03513	0	0
rxn03514	0	0
rxn03535	0	0
rxn03536	0	0
rxn03537	0	0
rxn03538	0	0
rxn03540	0	0
rxn03546	0	0
rxn03548	0	1000
rxn03549	0	0
rxn03552	0	0
rxn03553	0	0
rxn03558	0	0
rxn03598	0	0
rxn03599	0	0
rxn03602	0	0
rxn03603	0	0
rxn03634	0	0
rxn03638	0,062989949	0,06298995
rxn03641	0,000690955	6,535749504
rxn03642	0,000690955	6,535749504
rxn03644	0	0
rxn03728	0	0
rxn03798	-1000	1000
rxn03838	0	0
rxn03839	0	0
rxn03841	0	0,962610031
rxn03852	0	0
rxn03869	0	0
rxn03884	0	0
rxn03885	0	0
rxn03891	6,28141E-05	6,28141E-05
rxn03897	0	0
rxn03898	0	0
rxn03900	0	0
rxn03901	0,031494975	0,031494975
rxn03902	0	0
rxn03903	0	0
rxn03904	0,031494975	0,031494975
rxn03907	0	0
rxn03908	0	0
rxn03909	0	0
rxn03910	0	0

rxn03933	0	0
rxn03936	0	0
rxn03951	0	1000
rxn03952	0	0
rxn03958	0	0
rxn03962	0	0
rxn03964	0	0
rxn03978	0,024634536	6,40968052
rxn03990	0	5,341400434
rxn03991	0	5,341400434
rxn04016	0	0
rxn04023	0	0
rxn04045	0	0
rxn04046	0	0
rxn04047	0	0
rxn04048	0	0
rxn04050	0	0
rxn04052	0	0
rxn04068	0	0
rxn04074	0	0
rxn04113	0	0
rxn04137	0	0
rxn04142	0	0
rxn04234	0	0
rxn04286	0	0
rxn04308	0	0
rxn04384	0	0
rxn04385	0	0
rxn04413	0	0
rxn04432	0	0
rxn04443	0	0
rxn04565	0	0
rxn04651	0	0
rxn04674	0	0
rxn04676	-0,270954774	1000
rxn04678	-1000	0,270954774
rxn04681	0	0
rxn04682	0	0
rxn04703	0	0
rxn04704	0	0
rxn04726	0	0
rxn04736	0	0
rxn04794	0	1000
rxn04822	0	0
rxn04865	0	0
rxn04866	0	0
rxn04911	0	0
rxn04912	0	0
rxn04913	0	0
rxn04914	0	0

rxn04943	0	0
rxn04954	-8,829564893	0
rxn04960	0	0
rxn05003	0	0
rxn05005	-1000	0
rxn05006	-1000	0
rxn05010	0	0
rxn05011	0	0
rxn05028	6,28141E-05	6,28141E-05
rxn05029	0	0
rxn05030	6,28141E-05	6,28141E-05
rxn05039	0	0
rxn05050	0	0
rxn05054	0	0
rxn05072	0	0
rxn05089	0	0
rxn05092	0,007702147	0,007702147
rxn05104	0,007702147	0,007702147
rxn05105	0,007702147	0,007702147
rxn05106	0,007702147	0,007702147
rxn05108	0,007702147	0,007702147
rxn05115	0	0
rxn05116	0	1000
rxn05118	0	0
rxn05122	0	0
rxn05234	0	0
rxn05236	0	0
rxn05239	0	0
rxn05247	0	0
rxn05248	0	0
rxn05249	0	0
rxn05250	0	0
rxn05251	0	0
rxn05252	0	0
rxn05256	0	0
rxn05269	0	0
rxn05274	0	0
rxn05275	0	0
rxn05276	0	0
rxn05278	0	0
rxn05279	0	0
rxn05280	0	0
rxn05289	0	0
rxn05291	0	0
rxn05322	0	0
rxn05323	0	0
rxn05324	0	0
rxn05325	0	0
rxn05326	0	0
rxn05327	0	0

rxn05328	0	0
rxn05329	0	0
rxn05330	0	0
rxn05331	0	0
rxn05332	0	0
rxn05333	0	0
rxn05334	0	0
rxn05335	0	0
rxn05336	0	0
rxn05337	0	0
rxn05338	0	0
rxn05339	0	0
rxn05340	0	0
rxn05341	0	0
rxn05342	0	0
rxn05343	0	0
rxn05344	0	0
rxn05345	0	0
rxn05346	0	0
rxn05347	0	0
rxn05348	0	0
rxn05350	0	0
rxn05457	-1000	0
rxn05465	0	0
rxn05733	0	0
rxn05736	0	1000
rxn05740	-1000	1000
rxn05744	0	0
rxn05759	-0,6	0
rxn05760	-825,8274818	1000
rxn05778	0	0
rxn05779	0	0
rxn05794	-1000	0
rxn05824	0	0
rxn05833	0	0
rxn05853	0	0
rxn05854	0	0
rxn05871	0	0
rxn05872	0	0
rxn05873	0	0
rxn05874	0	0
rxn05899	0	0
rxn05901	0	0
rxn05902	-0,1	0
rxn05918	0	0
rxn05927	0	0
rxn05934	0	0
rxn05937	-1000	1000
rxn05938	-507,9682483	0
rxn05939	-506,7231651	1000

rxn05940	-1000	0,218988124
rxn05952	0	0
rxn05957	0	1000
rxn05958	0	0
rxn05962	0	0
rxn05964	0	0
rxn05970	0	0
rxn05990	0	0
rxn05994	0	0
rxn06005	0	0
rxn06023	0	0
rxn06025	0	0
rxn06043	0	1000
rxn06044	0	0
rxn06045	0	1000
rxn06071	0,000509365	343,3649543
rxn06078	0	0
rxn06079	0	1000
rxn06080	0	1000
rxn06087	0	0
rxn06090	0	0
rxn06091	0	0
rxn06096	0	0
rxn06108	-1000	0
rxn06109	-4,479624651	506,7233079
rxn06139	0	1000
rxn06140	0	0
rxn06181	0	1000
rxn06182	0	1000
rxn06190	0	0
rxn06194	0	0
rxn06195	0	0
rxn06196	0	0
rxn06197	0	0
rxn06200	0	0
rxn06201	0	0
rxn06209	0	0
rxn06217	0	0
rxn06218	0	0
rxn06219	0	0
rxn06231	0	0
rxn06252	-1000	0
rxn06253	0	1000
rxn06280	0	0
rxn06285	0	0
rxn06298	0	0
rxn06299	0	0
rxn06300	0	0
rxn06316	0	0
rxn06325	0	0

rxn06328	0	0
rxn06347	0	0
rxn06348	0	0
rxn06362	0	0
rxn06368	0	0
rxn06373	0	0
rxn06376	0	0
rxn06377	0	1000
rxn06381	0	0
rxn06390	0	0
rxn06394	0	0
rxn06403	0	0
rxn06432	0	0
rxn06434	0	0
rxn06435	0	0
rxn06437	0	0
rxn06438	0	0
rxn06439	0	0
rxn06440	0	0
rxn06441	0	0
rxn06443	0	0
rxn06444	0	0
rxn06445	0	0
rxn06446	0	0
rxn06447	0	0
rxn06448	0	0
rxn06449	0	0
rxn06459	0	0
rxn06472	0	0
rxn06485	0	0
rxn06493	0	1000
rxn06500	0	0
rxn06522	0	0
rxn06528	0	0
rxn06538	0	0
rxn06556	0	0
rxn06575	0	0
rxn06581	0	0
rxn06584	0	0
rxn06591	0,00203746	0,00203746
rxn06592	0	0
rxn06595	0	0
rxn06600	0	1000
rxn06608	0	0
rxn06624	0	0
rxn06648	0	0
rxn06664	0	0
rxn06671	0	0
rxn06672	0	1000
rxn06673	0	1000

rxn06678	0	0
rxn06691	0	0
rxn06699	0	0
rxn06701	0	0
rxn06709	0	0
rxn06723	0	0
rxn06726	0	0
rxn06737	0	0
rxn06741	0	0
rxn06751	0	0
rxn06752	0	0
rxn06760	0	0
rxn06768	0	0
rxn06799	0	0
rxn06817	0	0
rxn06820	0	0
rxn06823	0	0
rxn06831	0	0
rxn06850	0	0
rxn06860	0	0
rxn06864	0	0
rxn06865	0	0
rxn06874	0	0,1
rxn06882	0	0
rxn06883	0	0
rxn06887	0	0
rxn06889	0	1000
rxn06890	0	0
rxn06926	0	0
rxn06934	0	0
rxn06936	0	0
rxn06937	0,00203746	0,00203746
rxn06947	0	0
rxn06958	-343,3649543	-0,000509365
rxn06979	0	0
rxn06983	0	0
rxn07056	0	0
rxn07059	0	0
rxn07099	0	0
rxn07172	0	0
rxn07189	0	0
rxn07193	0	0
rxn07199	0	0
rxn07241	0	0
rxn07251	0	0
rxn07258	0	0
rxn07267	0	0
rxn07292	0	0
rxn07332	0	0
rxn07413	0	0

rxn07437	0	0
rxn07438	0	0
rxn07441	0	999,968505
rxn07456	0	1000
rxn07466	-1000	1000
rxn07476	0	0
rxn07486	0	0
rxn07489	0	0
rxn07573	0	0
rxn07577	0	0
rxn07578	0	0
rxn07579	0	0
rxn07580	0	0
rxn07581	0	0
rxn07584	0	0
rxn07585	0	0
rxn07586	0	0
rxn07587	0	0
rxn07623	0	0
rxn07679	0	0
rxn07683	0	0
rxn07687	0	0
rxn07804	0	0
rxn07807	0	0
rxn07832	0	0
rxn07846	0	0
rxn07889	0	0
rxn07890	0	0
rxn07896	0	0
rxn07897	0	0
rxn07898	0	0
rxn07987	0	0
rxn07989	0	0
rxn07991	0	0
rxn07992	0	0
rxn07993	0	0
rxn07994	0	0
rxn07997	0	0
rxn07998	0	0
rxn08000	0	0
rxn08001	0	0
rxn08002	0	0
rxn08035	0	0
rxn08038	0	0
rxn08040	0	0
rxn08043	0	2,382143703
rxn08067	-1000	1000
rxn08083	0	0
rxn08084	0	0
rxn08085	0	0

rxn08086	0	0
rxn08087	0	0
rxn08088	0	0
rxn08089	0	0
rxn08094	0	1000
rxn08126	0	0
rxn08127	0	0
rxn08128	0	0
rxn08129	0	0
rxn08171	0	0
rxn08180	0	0
rxn08194	-1000	1000
rxn08206	0	0
rxn08207	0	0
rxn08208	0	0
rxn08209	0	0
rxn08294	0	0
rxn08295	0	0
rxn08296	0	0
rxn08297	0	0
rxn08298	0	0
rxn08299	0	0
rxn08300	0	0
rxn08306	0	0
rxn08307	0	0
rxn08308	0	0
rxn08309	0	0
rxn08310	0	0
rxn08311	0	0
rxn08312	0	0
rxn08352	0	0
rxn08386	0	0
rxn08390	0	0
rxn08392	0	0
rxn08394	0	0
rxn08396	0	0
rxn08398	0	0
rxn08413	0	0
rxn08433	0	0
rxn08438	0	0
rxn08444	0	1000
rxn08448	0	0
rxn08449	0	0
rxn08451	0	0
rxn08453	0	0
rxn08454	0	1000
rxn08455	0	0
rxn08456	0	1000
rxn08457	0	0
rxn08519	0,057583371	0,057583371

rxn08546	0	0
rxn08547	0	1000
rxn08548	0	0
rxn08549	0	0
rxn08550	0	0
rxn08551	0	0
rxn08552	0	0
rxn08571	0	1000
rxn08582	0	0,5
rxn08605	0	0
rxn08607	0	0
rxn08615	-1000	1000
rxn08647	0	0
rxn08668	0	0
rxn08669	0	0
rxn08733	-1000	0
rxn08764	0	2,382143703
rxn08796	0	0
rxn08797	0	1000
rxn08798	0	0
rxn08799	0	1000
rxn08800	0	0
rxn08801	0	1000
rxn08802	0	0
rxn08803	0	0
rxn08804	0	0
rxn08805	0	0
rxn08806	0	0
rxn08807	0	0
rxn08808	0	0
rxn08809	0	0
rxn08810	0	0
rxn08811	0	0
rxn08812	0	0
rxn08813	0	0
rxn08814	0	0
rxn08815	0	0
rxn08816	0	0
rxn08817	0	0
rxn08818	0	0
rxn08819	0	0
rxn08820	0	0
rxn08821	0	0
rxn08822	0	0
rxn08823	0	0
rxn08838	0	0
rxn08839	0	0
rxn08840	0	0
rxn08841	0	0
rxn08842	0	0

rxn08843	0	0
rxn08844	0	0
rxn08845	0	0
rxn08846	0	0
rxn08847	0	0
rxn08848	0	0
rxn08849	0	0
rxn08850	0	0
rxn08851	0	0
rxn08857	0	0
rxn08889	0,000768616	0,000768616
rxn08890	0,006222019	0,006222019
rxn08891	0,000768616	0,000768616
rxn08892	-999,9854054	1000
rxn08893	-999,9923184	999,993087
rxn08894	-999,9854054	1000
rxn08897	-0,006912974	-0,006912974
rxn08926	0,000690955	0,000690955
rxn08927	-999,9984639	999,9869415
rxn08928	-999,9854054	1000
rxn08929	0,00153609	0,00153609
rxn08930	0	0
rxn08958	0,000768616	0,000768616
rxn09010	0	0
rxn09016	0	999,7611923
rxn09069	0	0
rxn09101	0	0
rxn09102	0	0
rxn09103	0	0
rxn09104	0	0
rxn09105	0	0
rxn09106	0	0
rxn09107	0	0
rxn09108	0	0
rxn09109	0	0
rxn09110	0	0
rxn09111	0	0
rxn09112	0	0
rxn09113	0	0
rxn09114	0	0
rxn09176	-1000	1000
rxn09177	0	0,000657835
rxn09179	0	0
rxn09180	0	0
rxn09197	0	0
rxn09198	0	0
rxn09199	0	0
rxn09200	0	0
rxn09201	0	0
rxn09202	0	0

rxn09203	0	0
rxn09205	0	0
rxn09206	0	0
rxn09207	0	0
rxn09208	0	0
rxn09209	0	0
rxn09210	0	0
rxn09211	0	0
rxn09235	0,028334856	0,028334856
rxn09237	0,029248515	0,029248515
rxn09240	0	0
rxn09264	0	0
rxn09265	0	0
rxn09395	0	0
rxn09398	-1000	999,5995158
rxn09399	0	0
rxn09412	-1000	1000
rxn09445	0	0
rxn09446	0	0
rxn09447	0	0
rxn09473	0	0
rxn09486	0	0
rxn09498	-1000	1000
rxn09499	-1000	1000
rxn09502	0	1000
rxn09521	0	0
rxn09531	0	0
rxn09557	0,000254683	1000
rxn09616	0,000690955	0,000690955
rxn09633	0,000254683	0,000254683
rxn09888	0	0
rxn09889	0	0
rxn09949	0	0
rxn09952	0	0
rxn09978	0	0
rxn09979	0	0
rxn09988	0	0
rxn09995	0	0
rxn10003	0	0,000657835
rxn10019	0	0
rxn10020	0	0
rxn10021	0	0
rxn10026	0	0
rxn10029	0	0
rxn10030	0	0
rxn10034	0	0
rxn10036	0	0
rxn10038	0	0
rxn10052	-1000	1000
rxn10054	0	999,6310107

rxn10056	0	0,000510507
rxn10058	0	0,000510507
rxn10060	0	0,000510507
rxn10091	-1000	1000
rxn10107	0	0
rxn10110	0	0
rxn10111	0	0
rxn10192	0	0
rxn10194	0	0
rxn10196	0	0
rxn10202	0	1000
rxn10203	0	1000
rxn10204	0	1000
rxn10205	0	0
rxn10206	0	0
rxn10207	0	0
rxn10208	0	0
rxn10209	0	0
rxn10210	0	0
rxn10211	0	0
rxn10212	0	0
rxn10213	0	0
rxn10214	0	0
rxn10215	0	0
rxn10216	0	0
rxn10217	0	0
rxn10218	0	0
rxn10219	0	0
rxn10220	0	0
rxn10221	0	0
rxn10222	0	0
rxn10223	0	0
rxn10224	0	0
rxn10225	0	0
rxn10226	0	0
rxn10227	0	0
rxn10228	0	0
rxn10229	0	0
rxn10230	0	0
rxn10231	0	0
rxn10232	0	0
rxn10233	0	0
rxn10234	0	0
rxn10235	0	0
rxn10236	0	0
rxn10237	0	0
rxn10253	0	0
rxn10254	0	0
rxn10255	0	0
rxn10256	0	0

rxn10257	0	0
rxn10258	0	0
rxn10259	0	0
rxn10260	0	0
rxn10261	0	0
rxn10262	0	0
rxn10263	0	0
rxn10264	0	0
rxn10265	0	0
rxn10266	0	0
rxn10267	0	0
rxn10268	0	0
rxn10269	0	0
rxn10270	0	0
rxn10289	0	0
rxn10290	0	0
rxn10291	0	0
rxn10292	0	0
rxn10293	0	0
rxn10294	0	0
rxn10295	0	0
rxn10296	0	0
rxn10297	0	0
rxn10363	0	0
rxn10404	0	0
rxn10405	0	0
rxn10406	0	0
rxn10407	0	0
rxn10408	0	0
rxn10409	0	0
rxn10410	0	0
rxn10816	0,000254683	0,000254683
rxn10951	0,028334856	0,028334856
rxn11007	0,028334856	0,028334856
rxn11510	0	0
rxn11511	0	0
rxn11513	0	0
rxn11547	0	0
rxn11548	0	0
rxn11550	0	0
rxn11551	-1000	1000
rxn11552	-1000	1000
rxn11564	0	0
rxn11567	0	0
rxn11571	-804,5356914	0
rxn11577	0	0
rxn11587	0	0
rxn11590	0	0
rxn11599	0	0
rxn11609	0	0

rxn11641	0	0
rxn11642	0	0
rxn11663	-1000	0
rxn11702	0	0
rxn11703	0	0
rxn11711	0	0
rxn11712	0	0
rxn11713	0	0
rxn11716	0	0
rxn11732	0	0
rxn11749	0	0
rxn11756	0	0
rxn11759	0	0
rxn11760	0	0
rxn11761	0	0
rxn11765	0	0
rxn11766	0	0
rxn11768	0	0
rxn11772	0	0
rxn11773	0	0
rxn11788	0	0
rxn11946	0	0
rxn11951	0	0
rxn11965	0	0
rxn11984	0	0
rxn11987	0	804,5356914
rxn12008	-6,28141E-05	-6,28141E-05
rxn12013	0	0
rxn12049	0	0
rxn12147	0	0
rxn12218	-1000	-0,000254683
rxn12221	0,000254683	1000
rxn12239	0,000254683	0,000254683
rxn12510	0,000657835	0,000657835
rxn12649	-1000	0
rxn12822	-1000	0
rxn13147	0,000254683	0,000254683
rxn13186	0	0
rxn13420	0,000690955	6,535749504
rxn13421	0,000690955	6,535749504
rxn13477	6,28141E-05	6,28141E-05
rxn13666	0	0
rxn13667	0	0
rxn13668	0	0
rxn13669	0	0
rxn13671	0	0
rxn13672	0	0
rxn13673	0	0
rxn13705	0	0
rxn13741	0	0

rxn13936	0,015363179	0,01536318
rxn13974	-507,9682483	0
rxn14012	6,28141E-05	6,28141E-05
rxn14028	0	0
rxn14029	0	0
rxn14033	0	0
rxn14042	0	0
rxn14043	0	0
rxn14048	-1000	0
rxn14050	0	0
rxn14054	-1000	0
rxn14057	-0,1	0
rxn14058	-3,20484026	-0,012317268
rxn14063	0	0
rxn14070	0	0
rxn14089	-1000	0
rxn14093	0	0
rxn14120	-1000	0,961591301
rxn14123	0	0
rxn14132	0	0
rxn14146	0	0
rxn14173	-10,68280087	-0,04105756
rxn14191	0	0
rxn14250	0	0
rxn14270	0	0
rxn14279	0	0
rxn14299	0	0
rxn14328	0	0
rxn14346	0	0
rxn90002	-6,8451321	1000
rxn90003	0	0
rxn90004	0	0
rxn90005	-0,028845363	-0,028334856
rxn08173	0	500
Biomass_Bacteria	1,142074	1,142074006
t_Cl	0,005153038	0,005153038
t_Sulfate	0,004294198	0,004294198
t_Cu2+	0,003435359	0,003435359
t_Mg	0,008587254	0,008587254
t_Ca2+	0,005153038	0,005153038
t_NH3	-2,382143703	0
t_H2O	-24,72367296	10
t_Biomass	-1,142074006	-1,142074
t_Butyrate	-6,535058549	0
t_D-Lactate	-8,513394645	0
t_Ethanol	0	0
t_Formate	-20,74084354	0
t_H2	-0,1	0,5
t_L-Lactate	-8,513394645	0
t_Nitrite	0	0

t_Phosphate	1,51196664	2,011966651
t_Propionate	-8,732382769	0
t_O2	0	0
t_D-Glucose	0	0
t_CO2	-20,74084354	0
t_Acetate	-12,77009197	0
t_Succinate	-6,385045984	0
t_(S,S)-2,3-Butanediol	0	0
t_BDOH	0	0
t_H2S	-0,497709	0
Ex_Cl	-0,005153038	-0,005153038
Ex_Sulfate	-0,004294198	-0,004294198
Ex_Cu2+	-0,003435359	-0,003435359
Ex_Mg	-0,008587254	-0,008587254
Ex_Ca2+	-0,005153038	-0,005153038
Ex_NH3	0	2,382143703
Ex_H2O	-10	24,72367296
Ex_Biomass	1,142074	1,142074006
Ex_Butyrate	0	6,535058549
Ex_D-Lactate	0	8,513394645
Ex_Ethanol	0	0
Ex_Formate	0	20,74084354
Ex_H2	-0,5	0,1
Ex_L-Lactate	0	8,513394645
Ex_Nitrite	0	0
Ex_Phosphate	-2,011966651	-1,51196664
Ex_Propionate	0	8,732382769
Ex_O2	0	0
Ex_D-Glucose	0	0
Ex_CO2	0	20,74084354
Ex_Acetate	0	12,77009197
Ex_Succinate	0	6,385045984
Ex_(S,S)-2,3-Butanediol	0	0
Ex_BDOH	0	0
Ex_H2S	0	0,497709
t_Fe2	0,007983097	8,521377743
t_fe3	-8,505666231	0,007728415
t_Acetaldehyde	0	0
t_Adenosine	0	0,5
t_Allantoin	0	0
t_AMP	0	0,5
t_Amylotriose	0	0
t_BIOT	0	0
t_Choline	0	0
t_Cytidine	0	0
t_Cytosine	0	0
t_DAlanine	0	0
t_Deoxyadenosine	0	0,057583371
t_Deoxycytidine	0	0,057583371
t_Deoxyguanosine	0	0

t_Deoxyinosine	0	0
t_Deoxyuridine	0	0
t_DRibose	0	0,5
t_DSerine	0	0
t_Glycerol	0	0
t_GSH	0	0
t_Guanine	0	0
t_H2S2O3	0	0
t_Heme	0,000254683	0,000254683
t_Homocysteine	0	0
t_HYXN	0	0,5
t_Inosine	0	0,5
t_LACT	0	0,5
t_LAlanine	-1,882143703	0,5
t_LArabinose	0	0
t_LArginine	-0,095535926	0,5
t_LAsparagine	-0,691071851	0,5
t_LAspartate	-1,882143703	0,5
t_LCysteine	0,002291	0,5
t_LGlutamate	-1,882143703	0,5
t_LGlutamine	-0,691071851	0,5
t_LHistidine	0,105185015	0,105185016
t_LInositol	0	0
t_LIsoleucine	-2,059736212	0,322407492
t_LLeucine	0,499999997	0,5
t_LLysine	-0,81030438	0,380767474
t_LMethionine	-0,326433304	0,171275697
t_LPhenylalanine	-2,176570383	0,205573321
t_LThreonine	0,281521241	0,5
t_LTryptophan	-1,127995104	0,063076747
t_LTyrosine	-2,228851104	0,153292599
t_LValine	-1,911723422	0,470420283
t_Maltose	0	0
t_Niacin	0	0,002602787
t_Ornithine	0	0
t_PPi	0	0
t_Pyridoxol	0	0
t_XAN	0	0
t_1,3Propanediol	0	0
t_5Deoxyadenosine	0	0
t_Acetoacetate	-6,535058549	0
t_BET	0	0
t_Calomide	0	0
t_Cbl	0	0
t_Citrate	0	0
t_CysGly	0	0
t_Dulcose	0	0
t_Glycine	-1,882143703	0,5
t_Glycolaldehyde	0	0
t_LProline	0,245317495	0,245317497

t_Maltohexaose	0	0
t_Methanol	0	0
t_NAcetylDglucosamine	0	0
t_PM	0	0
t_Putrescine	0	0
t_Pyridoxal	0,000254683	0,000254683
t_Riboflavin	0,000509365	0,000509365
t_Salicin	0	0
t_Sorbitol	0	0
t_Spermidine	0	0
t_Sucrose	0	0
t_Taurine	0	0
t_Thiamin	0	0
t_Thymidine	0	0
t_Thyminose	0	0,057583371
t_TRHL	0	0
t_Uracil	0	0,365829146
t_Uridine	0	0,365829146
t_Ursin	0	0
t_Mn2+	0,003435359	0,003435359
t_Fumarate	-6,385045984	0
t_Oxidized glutathione	0	0
t_Adenine	0	0
t_Nicotinamide	0	0
t_4-Hydroxybenzoate	0	0
t_Co2+	0,003435359	0,003435359
t_D-Glutamate	0	0
t_Nitrate	0	0
t_Chorismate	0	0
t_Folate	-0,961591301	0,00101873
t_N-Acetyl-D-mannosamine	0	0
t_Siroheme	0	0
t_Selenate	0	0
t_Menaquinone 7	0	0
t_2-Demethylmenaquinone 8	0	0
t_Menaquinone 8	0	0
t_Ubiquinone-8	0	0
t_2-Oxobutyrate	0	0
t_3MOP	0	0
t_ABEE	0	0
t_Neu5Ac	0	0
t_Glycerol-3-phosphate	0	0
t_H+	-1000	0,5
t_indol	0	0
t_Nicotinamide ribonucleotide	0	0
t_PAN	0,000657835	0,000657835
t_Pyridoxal phosphate	0	0
t_Zn2+	0,003435359	0,003435359
t_1,2-Diacyl-sn-glycerol dioctadecanoyl	0	0
t_meso-2,6-Diaminopimelate	0	0

t_L-Serine	-1,882143703	0,5
t_D-Fructose	0	0,5
t_D-Mannose	0	0
t_beta D-Galactose	0	0,5
t_L-Fucose	0	0
Ex_Fe2	-8,521377743	-0,007983097
Ex_fe3	-0,007728415	8,505666231
Ex_Acetaldehyde	0	0
Ex_Adenosine	-0,5	0
Ex_Allantoin	0	0
Ex_AMP	-0,5	0
Ex_Amylotriose	0	0
Ex_BIOT	0	0
Ex_Choline	0	0
Ex_Cytidine	0	0
Ex_Cytosine	0	0
Ex_DAlanine	0	0
Ex_Deoxyadenosine	-0,057583371	0
Ex_Deoxycytidine	-0,057583371	0
Ex_Deoxyguanosine	0	0
Ex_Deoxyinosine	0	0
Ex_Deoxyuridine	0	0
Ex_DRibose	-0,5	0
Ex_DSerine	0	0
Ex_Glycerol	0	0
Ex_GSH	0	0
Ex_Guanine	0	0
Ex_Heme	-0,000254683	-0,000254683
Ex_Homocysteine	0	0
Ex_HYXN	-0,5	0
Ex_Inosine	-0,5	0
Ex_LACT	-0,5	0
Ex_LAlanine	-0,5	1,882143703
Ex_LArabinose	0	0
Ex_LArginine	-0,5	0,095535926
Ex_LAsparagine	-0,5	0,691071851
Ex_LAspartate	-0,5	1,882143703
Ex_LCysteine	-0,5	-0,002291
Ex_LGlutamate	-0,5	1,882143703
Ex_LGlutamine	-0,5	0,691071851
Ex_LHistidine	-0,105185016	-0,105185015
Ex_LInositol	0	0
Ex_LIsoleucine	-0,322407492	2,059736212
Ex_LLeucine	-0,5	-0,499999997
Ex_LLysine	-0,380767474	0,81030438
Ex_LMethionine	-0,171275697	0,326433304
Ex_LPhenylalanine	-0,205573321	2,176570383
Ex_LThreonine	-0,5	-0,281521241
Ex_LTryptophan	-0,063076747	1,127995104
Ex_LTyrosine	-0,153292599	2,228851104

Ex_LValine	-0,470420283	1,911723422
Ex_Maltose	0	0
Ex_Niacin	-0,002602787	0
Ex_Ornithine	0	0
Ex_PPi	0	0
Ex_XAN	0	0
Ex_5Deoxyadenosine	0	0
Ex_Acetoacetate	0	6,535058549
Ex_BET	0	0
Ex_Calomide	0	0
Ex_Cbl	0	0
Ex_Citrate	0	0
Ex_CysGly	0	0
Ex_Dulcose	0	0
Ex_Glycine	-0,5	1,882143703
Ex_Glycolaldehyde	0	0
Ex_LProline	-0,245317497	-0,245317495
Ex_Maltohexaose	0	0
Ex_Methanol	0	0
Ex_NAcetylDglucosamine	0	0
Ex_PM	0	0
Ex_Putrescine	0	0
Ex_Pyridoxal	-0,000254683	-0,000254683
Ex_Riboflavin	-0,000509365	-0,000509365
Ex_Salicin	0	0
Ex_Sorbitol	0	0
Ex_Spermidine	0	0
Ex_Sucrose	0	0
Ex_Taurine	0	0
Ex_Thiamin	0	0
Ex_Thymidine	0	0
Ex_Thyminose	-0,057583371	0
Ex_TRHL	0	0
Ex_Uracil	-0,365829146	0
Ex_Uridine	-0,365829146	0
Ex_Ursin	0	0
Ex_Mn2+	-0,003435359	-0,003435359
Ex_Fumarate	0	6,385045984
Ex_Oxidized glutathione	0	0
Ex_Adenine	0	0
Ex_Nicotinamide	0	0
Ex_4-Hydroxybenzoate	0	0
Ex_Co2+	-0,003435359	-0,003435359
Ex_D-Glutamate	0	0
Ex_Nitrate	0	0
Ex_Folate	-0,00101873	0,961591301
Ex_N-Acetyl-D-mannosamine	0	0
Ex_Siroheme	0	0
Ex_Selenate	0	0
Ex_Menaquinone 7	0	0

Ex_2-Demethylmenaquinone 8	0	0
Ex_Menaquinone 8	0	0
Ex_Ubiquinone-8	0	0
Ex_ABEE	0	0
Ex_Neu5Ac	0	0
Ex_H+	-0,5	1000
Ex_indol	0	0
Ex_Nicotinamide ribonucleotide	0	0
Ex_PAN	-0,000657835	-0,000657835
Ex_Zn2+	-0,003435359	-0,003435359
Ex_1,2-Diacyl-sn-glycerol dioctadecanoyl	0	0
Ex_L-Serine	-0,5	1,882143703
Ex_D-Fructose	-0,5	0
Ex_D-Mannose	0	0
Ex_beta D-Galactose	-0,5	0
Ex_L-Fucose	0	0
t_Arabinan	0	0
t_Starch	0	0,005
t_octanoate	0	0
t_Melibiose	0	0,5
t_Linolenate	0	0
t_Amylose	0	0
t_Linoleate	0	0
Ex_Arabinan	0	0
Ex_Starch	-0,005	0
Ex_Melibiose	-0,5	0
Ex_Linolenate	0	0
Ex_Amylose	0	0
Ex_Linoleate	0	0
t_Raffinose_Melitose	0	0
t_Isovaleric_acid	0	0
t_H2O2	0	0
t_Nitric_oxide	0	0
Ex_Raffinose_Melitose	0	0
Ex_Isovaleric_acid	0	0
Ex_H2O2	0	0
Ex_Nitric_oxide	0	0
rxn01207_1	0	0
rxn08972	0	0
rxn08973	0	0
rxn06111	0	1000
rxn13726	0	0
rxn13727	0	0
rxn13729	0	0
rxn08974	0	0
rxn10122	0	0
rxn10123	0	0
rxn10124	0	0
rxn12665	0	0
rxn06097	0	0,005

t_Sulfite	0	0,1
Ex_Sulfite	-0,1	0
t_Nitrogen	0	0,1
Ex_Nitrogen	-0,1	0

rxn ID	minFlux	max Flux
rxn00001	0	1000
rxn00003	-1,509648261	0
rxn00011	-1,509648261	0
rxn00016	0	0
rxn00020	0	0
rxn00022	0	0,505
rxn00029	0,00101873	0,00101873
rxn00048	0	0
rxn00060	0,000254683	0,000254683
rxn00062	0	1000
rxn00065	0	1000
rxn00070	0	0
rxn00076	0	1000
rxn00077	0	0,000510507
rxn00085	-1000	0
rxn00086	0	0
rxn00097	-1000	1000
rxn00100	0,000657835	0,000657835
rxn00105	-999,9973972	1000
rxn00106	-1000	0
rxn00107	0	0
rxn00109	0	0
rxn00117	-9,366601954	1000
rxn00119	0,368989262	1000
rxn00122	0,000254683	0,000254683
rxn00124	0,000254683	0,000254683
rxn00126	0,000764048	999,9973972
rxn00132	0	999,9966332
rxn00133	0	0
rxn00137	0	0
rxn00139	-999,9971425	0
rxn00140	0	1000
rxn00142	0	0
rxn00143	0,000509365	999,9971425
rxn00144	0	0
rxn00148	-9,767086191	0
rxn00151	-9,767086191	0
rxn00154	0	14,87775356
rxn00157	-14,30353931	0
rxn00159	-1000	1000
rxn00161	-1000	1000
rxn00162	0	0,693876201
rxn00165	0	7,307534276
rxn00173	0	15,44870777
rxn00178	-1,03047625	0
rxn00179	0	0
rxn00184	-1000	0
rxn00187	0	1000
rxn00189	0	1000

rxn00190	0,002602787	1000
rxn00192	0	15,44870777
rxn00193	0,031494975	0,031494975
rxn00196	0	0
rxn00200	0	0
rxn00202	0	0
rxn00206	0,008588396	201,2085334
rxn00211	0	0
rxn00214	0	0
rxn00216	0	1000
rxn00221	0	1000
rxn00222	0	0
rxn00224	0,000254683	1000
rxn00225	-15,44870777	0
rxn00237	-9,796334706	1000
rxn00238	-9,767086191	1000
rxn00239	0,238807673	1000
rxn00242	0	1000
rxn00247	0	0,693876201
rxn00250	-0,343527488	-3,54043E-05
rxn00254	0	0
rxn00256	-0,693876201	0
rxn00259	-0,693876201	0
rxn00260	-1,509612857	0,693911605
rxn00273	0	0
rxn00274	0	0
rxn00278	-1,63888421	-0,129235949
rxn00283	0,027731841	0,027731841
rxn00290	-5,150260212	-0,000690955
rxn00293	0,062989949	999,6940007
rxn00297	0	0
rxn00301	0	999,7611923
rxn00303	0	1000
rxn00304	-9,767086191	0
rxn00307	0	0
rxn00313	0	0,693876201
rxn00324	-168,5573759	0
rxn00333	0	168,5573759
rxn00337	0,039197122	0,733073323
rxn00340	0	1000
rxn00342	0	1000
rxn00348	0	0
rxn00350	-0,000254683	-0,000254683
rxn00358	0	0
rxn00360	0	0
rxn00363	0	1000
rxn00364	-999,6310107	0,000657835
rxn00365	0	1000
rxn00368	0	1000
rxn00369	0	1000

rxn00383	0,004294198	0,004294198
rxn00388	0	0
rxn00391	0	999,9997453
rxn00392	0,000254683	1000
rxn00409	-1000	1000
rxn00410	-999,8225103	999,8085005
rxn00411	-9,767086191	0
rxn00412	0	1000
rxn00414	0	0,343492083
rxn00416	0	1000
rxn00423	0	7,307534276
rxn00426	0	0
rxn00433	0	0
rxn00436	0	999,9997453
rxn00437	0	0
rxn00440	0,000254683	1000
rxn00452	0	999,9966332
rxn00453	0	999,9997453
rxn00456	0	999,9997453
rxn00459	-0,21134879	9,133549186
rxn00460	-9,767086191	0
rxn00461	0,031494975	0,031494975
rxn00462	0	0
rxn00466	0	0
rxn00469	0	15,44870777
rxn00470	0,046135221	6,226180728
rxn00490	0	1,509648261
rxn00493	-1,509648261	0
rxn00499	-9,918502375	0
rxn00500	-9,918502375	0
rxn00505	0	0,693876201
rxn00506	0	1,302559388
rxn00514	0	0
rxn00515	-1000	1000
rxn00517	-9,767086191	0
rxn00518	0	1000
rxn00527	-1,509648261	0
rxn00533	-999,9999646	3,54043E-05
rxn00541	-1,302559388	0
rxn00545	0	3,818330669
rxn00546	0	0
rxn00547	0	1
rxn00551	0	3,818330669
rxn00552	-0,06298995	-0,062989949
rxn00554	0	3,818330669
rxn00556	0	3,818330669
rxn00557	0	3,818330669
rxn00558	-1000	1000
rxn00559	0	0,5
rxn00560	0	0

rxn00566	0	1000
rxn00575	0	0,5
rxn00585	0	0
rxn00604	-1000	1000
rxn00606	0	0
rxn00607	0	0
rxn00608	0	0
rxn00611	-0,401371631	0
rxn00615	0	0
rxn00616	0	0,401371631
rxn00622	0	0
rxn00623	-200,9342154	0
rxn00634	-1	0
rxn00635	0	1
rxn00645	0,004294198	200,9385096
rxn00646	0	0
rxn00647	0	0
rxn00649	0	7,307534276
rxn00650	-0,000254683	-0,000254683
rxn00653	0	0
rxn00670	0	15,44870777
rxn00673	0	0
rxn00677	-1000	1000
rxn00684	0	0
rxn00685	0	191,0722117
rxn00686	0	0
rxn00687	0	191,0722117
rxn00689	0	0
rxn00690	0	14,30404867
rxn00692	-0,260507082	7,047027194
rxn00693	0	0,393414802
rxn00695	-1000	1000
rxn00704	-1000	0
rxn00707	0	1000
rxn00708	0	1000
rxn00709	0	1000
rxn00710	0	0
rxn00711	-999,9966332	0
rxn00712	0	1000
rxn00713	0	1000
rxn00714	0	0
rxn00715	0	1000
rxn00729	0	0
rxn00735	0	0
rxn00737	0	1,521038145
rxn00740	0	1000
rxn00741	0	0
rxn00742	-1000	0,000254683
rxn00744	0	0
rxn00745	0	0

rxn00747	-4,169905969	0
rxn00756	0	0
rxn00758	0	0
rxn00762	0	0
rxn00770	0,003366834	1000
rxn00772	0	1000
rxn00775	0	0
rxn00776	0	0
rxn00777	-2,386086112	0,369817268
rxn00778	-1000	1000
rxn00780	0	0
rxn00781	-0,21134879	9,133549186
rxn00784	0	1,302559388
rxn00785	-2,260485953	1,275159141
rxn00786	0	3,818330669
rxn00787	0	0
rxn00789	0	0
rxn00790	-0,000254683	-0,000254683
rxn00792	0	0
rxn00796	0	0
rxn00799	-0,425129058	7,420516798
rxn00800	-0,26805619	0,425820012
rxn00802	0	0,343492083
rxn00806	0	0
rxn00816	0	0
rxn00817	0	0
rxn00818	0	0
rxn00819	0	0
rxn00829	0,000690955	0,000690955
rxn00830	6,28141E-05	6,28141E-05
rxn00831	0	999,9966332
rxn00832	0	0
rxn00834	-999,7290863	1000
rxn00836	-999,9971425	0
rxn00838	-0,26805619	0,425820012
rxn00851	0	1000
rxn00855	0	0
rxn00856	0,008138419	6,188183926
rxn00858	0	0
rxn00872	0	0
rxn00874	0	0
rxn00879	0	0
rxn00889	0	0
rxn00890	0	0
rxn00898	0	1,509648261
rxn00902	0	0
rxn00903	-1,509648261	0
rxn00907	-14,30353931	0,000509365
rxn00908	-7,448876208	0,033723164
rxn00909	-0,007702147	0,393669484

rxn00910	-0,401371631	0
rxn00913	0	1000
rxn00915	-999,9966332	0
rxn00916	-999,7319438	0,26805619
rxn00917	0	1000
rxn00918	0	0
rxn00925	0	0
rxn00927	-1000	1000
rxn00929	-1000	1000
rxn00931	-1000	1000
rxn00942	0	1000
rxn00946	0	0
rxn00947	0	1000
rxn00950	-1000	0,392905437
rxn00952	0	999,9997453
rxn00955	0,000509365	999,9971425
rxn00972	-0,693876201	0
rxn00973	-1000	1000
rxn00974	-1000	1000
rxn00975	0	0,5
rxn00976	0	0,5
rxn00977	0	0
rxn00980	0	0
rxn00983	0	0
rxn00985	-15,44870777	0
rxn00991	-0,000690955	-0,000690955
rxn00992	0	0
rxn01000	0	1,509648261
rxn01007	0	0
rxn01011	0	0
rxn01013	0	0
rxn01016	0	0
rxn01018	0	0
rxn01019	0	0,343492083
rxn01021	0	0
rxn01042	0	0
rxn01056	-1000	1000
rxn01069	0	0
rxn01071	0	0
rxn01073	0	0
rxn01089	0	0
rxn01100	-1000	0
rxn01101	0	0
rxn01106	-9,133549186	0,21134879
rxn01107	0	0
rxn01114	0	0
rxn01115	0	5,727496003
rxn01116	-1,206827322	4,02148127
rxn01124	0	0
rxn01133	0	0

rxn01137	0	0,693876201
rxn01138	-1000	1000
rxn01139	0	0
rxn01146	0	0
rxn01169	0	1000
rxn01171	0	1000
rxn01188	0	0
rxn01199	0	0
rxn01200	0	1000
rxn01201	-6,180736462	-0,000690955
rxn01204	0,000690955	6,180736462
rxn01210	0	0
rxn01211	-14,30379399	0,000509365
rxn01213	6,28141E-05	6,28141E-05
rxn01226	-999,9707515	1000
rxn01228	0	0
rxn01237	0	0
rxn01241	0	0
rxn01255	0,000254683	1,509902944
rxn01256	0	1,509648261
rxn01265	-999,999236	-0,002602787
rxn01268	0	1,509648261
rxn01270	-1,509648261	0
rxn01274	0	0
rxn01276	0	0
rxn01278	0	0
rxn01280	0	0
rxn01281	0	0
rxn01286	0	0
rxn01292	0	0,5
rxn01297	-999,7319438	999,9971425
rxn01299	-1000	1000
rxn01300	0	0
rxn01303	0	0
rxn01304	0	0
rxn01321	0	0
rxn01329	0	0
rxn01332	0,000254683	1,509902944
rxn01334	0	2,260740635
rxn01343	0	2,260740635
rxn01346	0	2,260740635
rxn01347	0	2,260740635
rxn01348	0	2,260740635
rxn01351	0	1000
rxn01352	-1000	-0,029248515
rxn01353	-1000	1000
rxn01354	-9,767086191	0
rxn01355	0	0
rxn01358	-1000	1000
rxn01361	0	0

rxn01362	0	0
rxn01366	-0,33749429	1000
rxn01367	0	0
rxn01368	0	999,6310107
rxn01370	0	1000
rxn01377	0	0
rxn01379	0	0
rxn01388	-1000	1000
rxn01423	0	0
rxn01426	0	0
rxn01434	0	0,343492083
rxn01437	0	0
rxn01439	0	0
rxn01442	0	0
rxn01445	0	999,9707515
rxn01446	-0,029248515	-0,029248515
rxn01452	-999,999309	0
rxn01459	0	6,180481779
rxn01465	0	0
rxn01466	6,28141E-05	6,28141E-05
rxn01476	0	5,727496003
rxn01480	0	0
rxn01484	0	0
rxn01485	-0,06298995	-0,062989949
rxn01486	0	0
rxn01492	0	0
rxn01500	-0,000690955	-0,000690955
rxn01506	0	0
rxn01509	-999,9707515	1000
rxn01510	0	1000
rxn01513	0,028334856	0,028334856
rxn01517	0	1000
rxn01518	0,028334856	1000
rxn01519	0	1000
rxn01521	0	999,9716651
rxn01522	0	0
rxn01538	0	999,9997453
rxn01539	-1000	-0,000254683
rxn01544	-999,9971425	0
rxn01545	-1000	1000
rxn01548	-999,9707515	1000
rxn01549	0	0
rxn01575	-1,509648261	0
rxn01594	0	0
rxn01601	0	0
rxn01602	0	0
rxn01603	0	0
rxn01610	0	0
rxn01629	-0,00203746	-0,00203746
rxn01636	-15,0659725	6,226180728

rxn01637	-6,226180728	-0,046135221
rxn01642	0	0
rxn01643	-0,733073323	-0,039197122
rxn01644	0,031494975	0,725371176
rxn01646	-1000	0
rxn01647	0	999,9966332
rxn01648	0	0
rxn01649	-1000	1000
rxn01650	0	0
rxn01653	0	0
rxn01666	0	1000
rxn01667	-9,767086191	0
rxn01669	0	999,9973972
rxn01670	0	0
rxn01675	0	0
rxn01678	0	1000
rxn01679	0	1000
rxn01683	-1000	1000
rxn01684	-1000	1000
rxn01686	0	0
rxn01704	0	0
rxn01706	0	1000
rxn01735	0	0
rxn01739	0,000254683	1,509902944
rxn01740	-1,509902944	-0,000254683
rxn01741	0	0
rxn01757	0	0
rxn01758	-200,9385096	-0,004294198
rxn01763	0	0,5
rxn01790	0	0
rxn01799	-0,028334856	0,33749429
rxn01800	0	0,365829146
rxn01816	0	1000
rxn01829	0	0
rxn01831	0	0
rxn01834	0	0
rxn01851	0	6,180481779
rxn01857	0	0
rxn01858	0	0,693876201
rxn01859	-0,693876201	0,494313614
rxn01860	0	0
rxn01870	0	0
rxn01895	0	0
rxn01898	0	0
rxn01906	0	0
rxn01917	0,046135221	6,226180728
rxn01919	0	0
rxn01937	0	0
rxn01953	0	0
rxn01961	0	999,9966332

rxn01962	0	0
rxn01967	0	0
rxn01972	0,031494975	15,48020275
rxn01973	0	0
rxn01974	0,031494975	0,725371176
rxn01975	-1000	1000
rxn01977	-1000	1000
rxn01982	0	0
rxn01985	0	0,693876201
rxn01986	-0,057583371	0,802559388
rxn01987	-0,5	0
rxn01991	0	0
rxn01997	0	0
rxn01998	0	0
rxn01999	0	0
rxn02003	0	0
rxn02008	0,031494975	0,031494975
rxn02011	0,031494975	0,031494975
rxn02012	0	0
rxn02020	0	0
rxn02021	0	0
rxn02023	0	0
rxn02042	0	1000
rxn02043	-1000	0
rxn02046	0	0
rxn02056	0	999,9997453
rxn02057	0	1
rxn02059	0	1
rxn02093	0	0
rxn02100	0	0
rxn02106	0	0
rxn02122	0	0
rxn02128	0	0
rxn02134	0	0
rxn02138	0	0
rxn02139	0	0
rxn02144	0	0
rxn02154	0	999,9973972
rxn02155	0,002602787	1000
rxn02160	0	0
rxn02166	0	0
rxn02167	0	999,999309
rxn02171	0,000690955	6,180736462
rxn02175	0,000657835	0,000657835
rxn02185	-1,509648261	1,509648261
rxn02186	0	1,509648261
rxn02187	0	0
rxn02195	0	0
rxn02200	0	0
rxn02201	0	0

rxn02212	0,000254683	1,509902944
rxn02213	0,000254683	1,509902944
rxn02219	0	0
rxn02222	0	0
rxn02228	0	0
rxn02236	0	0
rxn02264	0,000254683	0,000254683
rxn02284	-0,031494975	0
rxn02285	-0,031494975	0
rxn02286	0,031494975	0,031494975
rxn02287	-191,0722117	1000
rxn02288	0	0
rxn02302	-1000	-0,000254683
rxn02305	0,000254683	0,000254683
rxn02314	0	1000
rxn02315	0	1000
rxn02316	0	1000
rxn02317	-1000	0
rxn02320	0	0
rxn02322	0,000690955	0,000690955
rxn02339	0	0
rxn02341	0,000657835	0,000657835
rxn02350	0	0
rxn02351	0	0
rxn02356	-1000	1000
rxn02358	-1000	1000
rxn02373	-1000	1000
rxn02377	0	0
rxn02380	-1000	1000
rxn02400	0	999,9966332
rxn02409	0	0
rxn02444	0	0
rxn02449	0	1000
rxn02452	0	0
rxn02454	0	0
rxn02465	-6,226180728	-0,046135221
rxn02473	0	0
rxn02474	0	0
rxn02475	0	0
rxn02476	0,000254683	1,509902944
rxn02495	0	0
rxn02503	0	0
rxn02517	0	1000
rxn02518	0	0
rxn02521	0	0
rxn02522	0	0
rxn02525	0	0
rxn02527	0	1000
rxn02528	-1000	0
rxn02571	0	0

rxn02581	0	0
rxn02596	0	0
rxn02629	0	0
rxn02632	0	0
rxn02663	0	0
rxn02679	0	0
rxn02720	0	0
rxn02727	0	0
rxn02729	0	0
rxn02740	0	0
rxn02749	0	0
rxn02751	0	0
rxn02762	0	0
rxn02774	-191,0722117	0
rxn02776	0	0
rxn02789	0	0
rxn02791	0	0
rxn02792	0	0
rxn02798	0	0
rxn02803	0	0
rxn02808	0	0
rxn02809	0	0
rxn02810	0	0
rxn02811	0	0
rxn02821	0	0
rxn02822	0	0
rxn02834	0	0
rxn02835	0	0
rxn02845	0	0
rxn02853	0	0
rxn02866	0	0
rxn02875	0	0
rxn02895	0,000254683	0,000254683
rxn02897	0	0
rxn02900	0	0
rxn02914	0	0
rxn02922	0	0
rxn02928	-1000	999,968505
rxn02929	-1000	999,968505
rxn02931	0	0
rxn02936	0	0
rxn02937	0,000254683	0,000254683
rxn02990	0	0
rxn03004	0	0,000254683
rxn03005	-0,000254683	0
rxn03030	0,031494975	15,48020275
rxn03039	0	0
rxn03047	0	0
rxn03061	0	0
rxn03062	0	0

rxn03066	0	0
rxn03068	0	0
rxn03084	0,000254683	0,000254683
rxn03086	-15,48020275	-0,031494975
rxn03087	0	0
rxn03102	0	0
rxn03106	0	0
rxn03108	0,000254683	0,000254683
rxn03123	0	0
rxn03135	0	0
rxn03136	0	0
rxn03137	0	0
rxn03140	0	0
rxn03141	0	0
rxn03147	0	0
rxn03150	0	0
rxn03158	0	0
rxn03164	0,031494975	0,031494975
rxn03175	0	0
rxn03194	0	1,509648261
rxn03251	0	0
rxn03253	0	0
rxn03263	0	0
rxn03264	0	0
rxn03269	0	0
rxn03275	0	0
rxn03354	0	0
rxn03362	0	0
rxn03371	0	0
rxn03374	0	0
rxn03379	0	0
rxn03382	0	0
rxn03383	0	0
rxn03384	0	0
rxn03397	0	0
rxn03402	0	0
rxn03405	0	0
rxn03406	0	0
rxn03407	0	0
rxn03408	0,031494975	0,031494975
rxn03409	0	0
rxn03423	0	0
rxn03435	-1,509648261	0
rxn03436	0	1,509648261
rxn03437	0	1,509648261
rxn03445	0	0
rxn03446	0	0
rxn03462	0	0
rxn03465	0	0
rxn03467	0	0

rxn03468	0	0
rxn03481	0	0
rxn03482	0	0
rxn03483	0	0
rxn03535	0	0
rxn03552	0	0
rxn03553	0	0
rxn03596	0	0
rxn03598	0	0
rxn03599	0	0
rxn03634	0	0
rxn03638	0,062989949	0,06298995
rxn03641	0,000690955	6,180736462
rxn03642	0,000690955	6,180736462
rxn03643	0	0
rxn03644	0	0
rxn03660	0	0
rxn03661	0	0
rxn03662	0	0
rxn03668	0	0
rxn03669	0	0
rxn03670	0	0
rxn03671	0	0
rxn03798	-1000	1000
rxn03799	0	0
rxn03807	0	0
rxn03838	0	0
rxn03861	0	0
rxn03870	0	0
rxn03886	0	1
rxn03891	0	0
rxn03895	0	0
rxn03900	0	0
rxn03901	0,031494975	0,031494975
rxn03902	0	0
rxn03903	0	0
rxn03904	0,031494975	0,031494975
rxn03907	0	0
rxn03908	0	0
rxn03909	0	0
rxn03910	0	0
rxn03917	0	0
rxn03933	0	0
rxn03958	0	0
rxn03962	0	0
rxn03964	0	0
rxn03974	-0,028334856	0
rxn03975	-0,028334856	0
rxn04082	0	0,5
rxn04113	0	0

rxn04142	0	0
rxn04234	0	0
rxn04290	0	0
rxn04308	0	0
rxn04417	0	0
rxn04418	0	0
rxn04432	0	0
rxn04443	0	0
rxn04604	0	0
rxn04630	0	0
rxn04631	0	0
rxn04674	0	0
rxn04676	0	1000
rxn04678	-1000	0
rxn04704	0	0
rxn04750	0	0
rxn04786	0,007702147	0,007702147
rxn04794	0	14,30353931
rxn04809	0	0
rxn04810	0	0
rxn04811	0	0
rxn04822	0	0
rxn04830	0	0
rxn04831	0	0
rxn04832	0	0
rxn04833	0	0
rxn04865	0	0
rxn04866	0	0
rxn04872	0	0
rxn04873	0	0
rxn04886	0	0
rxn04887	0	0
rxn04894	0	0
rxn04895	0	0
rxn04896	0	0
rxn04903	0	0
rxn04916	0	0
rxn04919	0	0
rxn04943	0	0
rxn04954	-0,401371631	0
rxn05005	-1000	0
rxn05006	-1000	0
rxn05010	0	0
rxn05029	0	0
rxn05030	6,28141E-05	6,28141E-05
rxn05039	0	0
rxn05050	0	0
rxn05108	0	0
rxn05115	0	0
rxn05124	0	0

rxn05233	0	0
rxn05234	0	0
rxn05236	0	0
rxn05247	0	0
rxn05248	0	0
rxn05249	0	0
rxn05250	0	0
rxn05251	0	0
rxn05252	0	0
rxn05269	0	0
rxn05289	0	0
rxn05322	0	0
rxn05323	0	0
rxn05324	0	0
rxn05325	0	0
rxn05326	0	0
rxn05327	0	0
rxn05328	0	0
rxn05329	0	0
rxn05330	0	0
rxn05331	0	0
rxn05332	0	0
rxn05333	0	0
rxn05334	0	0
rxn05335	0	0
rxn05336	0	0
rxn05337	0	0
rxn05338	0	0
rxn05339	0	0
rxn05340	0	0
rxn05341	0	0
rxn05342	0	0
rxn05343	0	0
rxn05344	0	0
rxn05345	0	0
rxn05346	0	0
rxn05347	0	0
rxn05348	0	0
rxn05350	0	0
rxn05457	-1000	0
rxn05465	0	0
rxn05733	0	0
rxn05736	0	1000
rxn05740	-1000	1000
rxn05759	-0,5	0
rxn05760	-1,250094808	1000
rxn05762	0,004294198	200,9385096
rxn05778	0	0
rxn05779	0	0
rxn05853	0	0

rxn05878	0	0
rxn05893	-0,1	0
rxn05899	0	0
rxn05901	0	0
rxn05918	0	0
rxn05927	0	0
rxn05934	0	0
rxn05937	-1000	1000
rxn05938	-14,87775356	0
rxn05939	0,000579032	1000
rxn05940	-14,30353931	1,521292828
rxn05952	0	0
rxn05957	0	1000
rxn05958	0	0
rxn05962	0	0
rxn05970	0	0
rxn05979	0	0
rxn05990	0	0
rxn05994	0	0
rxn06023	0	0
rxn06025	0	0
rxn06043	0	0
rxn06044	0	0
rxn06045	0	0
rxn06071	0,017176793	402,4170667
rxn06075	0	0
rxn06077	0	0
rxn06078	0	0
rxn06081	0	0
rxn06091	0	0
rxn06096	0	0
rxn06108	-191,0729026	-0,000690955
rxn06109	-7,152460609	-0,000690955
rxn06139	0	0
rxn06140	0	0
rxn06154	0	0
rxn06155	0	0
rxn06181	0	1000
rxn06182	0	1000
rxn06190	0	0
rxn06194	0	0
rxn06195	0	0
rxn06196	0	0
rxn06200	0	0
rxn06201	0	0
rxn06217	0	0
rxn06218	0	0
rxn06227	0	0
rxn06231	0	0
rxn06244	0	0

rxn06280	0	0
rxn06285	0	0
rxn06298	0	0
rxn06300	0	0
rxn06316	0	0
rxn06328	0	0
rxn06347	0	0
rxn06348	0	0
rxn06376	0	0
rxn06377	0	0
rxn06381	0	0
rxn06394	0	0
rxn06403	0	0
rxn06432	0	0
rxn06434	0	0
rxn06435	0	0
rxn06438	0	0
rxn06439	0	0
rxn06440	0	0
rxn06441	0	0
rxn06443	0	0
rxn06444	0	0
rxn06445	0	0
rxn06446	0	0
rxn06447	0	0
rxn06448	0	0
rxn06449	0	0
rxn06459	0	0
rxn06485	0	0
rxn06489	0	0
rxn06493	0	0
rxn06500	0	0
rxn06525	0	1000
rxn06526	-1000	0
rxn06528	0	0
rxn06538	0	0
rxn06556	0	0
rxn06565	0	0
rxn06578	0	0
rxn06584	0	0
rxn06591	0,00203746	0,00203746
rxn06592	0	0
rxn06608	0	0
rxn06624	0	0
rxn06641	0	0
rxn06648	0	0
rxn06660	0	0
rxn06664	0	0
rxn06672	0	1000
rxn06673	0	1000

rxn06677	0	0
rxn06678	0	0
rxn06691	0	0
rxn06701	0	0
rxn06726	0	0
rxn06737	0	0
rxn06751	0	0
rxn06752	0	0
rxn06760	0	0
rxn06768	0	0
rxn06798	0	0
rxn06817	0	0
rxn06820	0	0
rxn06823	0	0
rxn06831	0	0
rxn06860	0	0
rxn06864	0	0
rxn06882	0	0
rxn06883	0	0
rxn06890	0	0
rxn06934	0	0
rxn06936	0	0
rxn06937	0,00203746	0,00203746
rxn06947	0	0
rxn06958	-402,4170667	-0,017176793
rxn07056	0	0
rxn07099	0	0
rxn07181	0	0
rxn07193	0	0
rxn07199	0	0
rxn07267	0	0
rxn07292	0	0
rxn07439	0	0
rxn07441	0	15,44870777
rxn07450	0,004294198	200,9385096
rxn07452	0	0
rxn07456	0	0,343492083
rxn07465	0,007702147	0,007702147
rxn07466	-0,029267931	999,9707321
rxn07474	0	0
rxn07476	0	0
rxn07484	0	0
rxn07485	0	0
rxn07486	0	0
rxn07573	0	0
rxn07577	0	0
rxn07578	0	0
rxn07579	0	0
rxn07580	0	0
rxn07645	0	0

rxn07679	0	0
rxn07683	0	0
rxn07687	0	0
rxn07804	0	0
rxn07807	0	0
rxn07832	0	0
rxn07846	0	0
rxn07849	0	0
rxn07987	0	0
rxn07989	0	0
rxn07991	0	0
rxn07992	0	0
rxn07993	0	0
rxn07994	0	0
rxn08025	0	0
rxn08035	0	0
rxn08038	0	0
rxn08040	0	0
rxn08043	0	1,509648261
rxn08044	0	0
rxn08067	-1000	1000
rxn08083	0	0
rxn08084	0	0
rxn08085	0	0
rxn08086	0	0
rxn08087	0	0
rxn08088	0	0
rxn08089	0	0
rxn08094	0	999,999421
rxn08126	0	0
rxn08127	0	0
rxn08128	0	0
rxn08129	0	0
rxn08131	0,000254683	0,000254683
rxn08171	0	0
rxn08180	0	0
rxn08194	-1000	1000
rxn08294	0	0
rxn08295	0	0
rxn08296	0	0
rxn08297	0	0
rxn08298	0	0
rxn08299	0	0
rxn08300	0	0
rxn08306	0	0
rxn08307	0	0
rxn08308	0	0
rxn08309	0	0
rxn08310	0	0
rxn08311	0	0

rxn08312	0	0
rxn08352	0	0
rxn08386	0	0
rxn08390	0	0
rxn08392	0	0
rxn08394	0	0
rxn08396	0	0
rxn08398	0	0
rxn08413	0	0
rxn08433	0	0
rxn08438	0	0
rxn08448	0	0
rxn08449	0	0
rxn08451	0	0
rxn08453	0	0
rxn08454	0	1000
rxn08455	0	0
rxn08456	0	0
rxn08457	0	0
rxn08519	0,057583371	0,057583371
rxn08546	0	0
rxn08547	0	1000
rxn08548	0	0
rxn08549	0	0
rxn08550	0	0
rxn08551	0	0
rxn08552	0	0
rxn08571	0	0
rxn08582	0	0
rxn08605	0	0
rxn08607	0	0
rxn08615	-1000	1000
rxn08647	0	0
rxn08668	0	0
rxn08669	0	0
rxn08764	0	1,509648261
rxn08796	0	0
rxn08797	0	1000
rxn08798	0	0
rxn08799	0	1000
rxn08800	0	0
rxn08801	0	1000
rxn08802	0	0
rxn08803	0	0
rxn08804	0	0
rxn08805	0	0
rxn08806	0	0
rxn08807	0	0
rxn08808	0	0
rxn08809	0	0

rxn08810	0	0
rxn08811	0	0
rxn08812	0	0
rxn08813	0	0
rxn08814	0	0
rxn08815	0	0
rxn08816	0	0
rxn08817	0	0
rxn08818	0	0
rxn08819	0	0
rxn08820	0	0
rxn08821	0	0
rxn08822	0	0
rxn08823	0	0
rxn08838	0	0
rxn08839	0	0
rxn08840	0	0
rxn08841	0	0
rxn08842	0	0
rxn08843	0	0
rxn08844	0	0
rxn08845	0	0
rxn08846	0	0
rxn08847	0	0
rxn08848	0	0
rxn08849	0	0
rxn08850	0	0
rxn08851	0	0
rxn08857	0	0
rxn08889	0,000768616	0,000768616
rxn08890	0,006222019	0,006222019
rxn08891	0,000768616	0,000768616
rxn08892	0,013058474	0,013058474
rxn08893	0,0061455	0,0061455
rxn08894	0,00153609	0,00153609
rxn08897	-0,006912974	-0,006912974
rxn08926	0,000690955	0,000690955
rxn08928	0,00153609	0,00153609
rxn08929	0,00153609	0,00153609
rxn08958	0,000768616	0,000768616
rxn09010	0	0
rxn09011	0	1000
rxn09012	0	0
rxn09016	0	999,7611923
rxn09108	0	0
rxn09109	0	0
rxn09110	0	0
rxn09111	0	0
rxn09112	0	0
rxn09113	0	0

rxn09114	0	0
rxn09177	0	0,000657835
rxn09205	0	0
rxn09206	0	0
rxn09207	0	0
rxn09208	0	0
rxn09209	0	0
rxn09210	0	0
rxn09211	0	0
rxn09235	0,028334856	0,028334856
rxn09237	0,029248515	0,029248515
rxn09264	0	0
rxn09265	0	0
rxn09340	0	0
rxn09341	0	999,6310107
rxn09348	0	0
rxn09355	0	0
rxn09395	0	0
rxn09398	0	0
rxn09399	0	0
rxn09412	-1000	1000
rxn09445	0	0
rxn09446	0	0
rxn09447	0	0
rxn09473	0	0
rxn09486	0	0
rxn09499	0	0
rxn09502	0	1000
rxn09516	0	1000
rxn09519	0	0
rxn09521	0	0
rxn09531	0	0
rxn09557	0,000254683	191,0724663
rxn09616	0,000690955	0,000690955
rxn09631	0,000254683	0,000254683
rxn09889	0	0
rxn09949	0	0
rxn09952	0	0
rxn09978	0	0
rxn09979	0	0
rxn09992	0	0
rxn09995	0	0
rxn10003	0	0,000657835
rxn10019	0	0
rxn10020	0	0
rxn10021	0	0
rxn10052	-1000	1000
rxn10054	0	999,6310107
rxn10056	0	0,000510507
rxn10058	0	0,000510507

rxn10060	0	0,000510507
rxn10091	-1000	1000
rxn10111	0	0
rxn10191	0	0
rxn10192	0	0
rxn10193	0	0
rxn10194	0	0
rxn10196	0	0
rxn10202	0	1000
rxn10203	0	1000
rxn10204	0	1000
rxn10205	0	0
rxn10206	0	0
rxn10207	0	0
rxn10208	0	0
rxn10209	0	0
rxn10210	0	0
rxn10211	0	0
rxn10212	0	0
rxn10213	0	0
rxn10214	0	0
rxn10215	0	0
rxn10216	0	0
rxn10217	0	0
rxn10218	0	0
rxn10219	0	0
rxn10220	0	0
rxn10221	0	0
rxn10222	0	0
rxn10223	0	0
rxn10224	0	0
rxn10225	0	0
rxn10226	0	0
rxn10227	0	0
rxn10228	0	0
rxn10229	0	0
rxn10230	0	0
rxn10231	0	0
rxn10253	0	0
rxn10254	0	0
rxn10255	0	0
rxn10256	0	0
rxn10257	0	0
rxn10258	0	0
rxn10259	0	0
rxn10260	0	0
rxn10261	0	0
rxn10262	0	0
rxn10263	0	0
rxn10264	0	0

rxn10289	0	0
rxn10290	0	0
rxn10291	0	0
rxn10292	0	0
rxn10293	0	0
rxn10294	0	0
rxn10295	0	0
rxn10296	0	0
rxn10297	0	0
rxn10298	0	0
rxn10299	0	0
rxn10300	0	0
rxn10301	0	0
rxn10302	0	0
rxn10303	0	0
rxn10304	0	0
rxn10305	0	0
rxn10306	0	0
rxn10363	0	0
rxn10404	0	0
rxn10405	0	0
rxn10406	0	0
rxn10407	0	0
rxn10408	0	0
rxn10409	0	0
rxn10410	0	0
rxn10785	6,28141E-05	6,28141E-05
rxn10951	0	0,028334856
rxn11007	0,028334856	0,028334856
rxn11547	0	0
rxn11548	0	0
rxn11550	0	0
rxn11571	0	0
rxn11577	0	0
rxn11587	0	0
rxn11599	0	0
rxn11609	0	0
rxn11703	0	0
rxn11732	0	0
rxn11749	0	0
rxn11757	-999,9966332	0
rxn11759	0	999,9966332
rxn11760	-999,9966332	0
rxn11761	0	0
rxn11764	0	0
rxn11765	0	0
rxn11766	0	0
rxn11767	0	0
rxn11768	0	0
rxn11772	0	0

rxn11773	0	0
rxn11774	0	0
rxn11808	0	0
rxn11878	0	0
rxn11879	0	0
rxn11890	0	0
rxn11934	0	0
rxn11951	0	0
rxn11965	0	0
rxn12013	0	0
rxn12033	0	0
rxn12053	0	0
rxn12054	0	0
rxn12154	0	0
rxn12218	-1000	-0,000254683
rxn12221	0,000254683	1000
rxn12510	0,000657835	0,000657835
rxn12649	-999,9989813	0
rxn12778	0	0
rxn12822	-191,0722117	0
rxn13420	0,000690955	1000
rxn13421	0,000690955	1000
rxn13705	0	0
rxn13741	0	0
rxn13906	-0,007702147	-0,007702147
rxn13936	0,015363179	0,01536318
rxn13963	-401,8770192	-0,008588396
rxn13974	-14,87775356	0
rxn13994	0	0
rxn14043	0	0
rxn14070	0	0
rxn14120	-1000	-0,00101873
rxn14123	0	0
rxn14132	0	0
rxn14180	0	0
rxn14191	0	0
rxn14238	0	0
rxn14250	0	0
rxn14275	0	0
rxn14279	0	0
rxn14328	0	0
rxn14346	0	0
rxn14372	0	0
rxn90002	-9,133549186	1000
rxn90003	0	0
rxn90004	0	0
rxn90005	-0,028845363	-0,028334856
rxn08173	0	95,53610583
Biomass_Bacteria	1,142074	1,142074006
t_Cl	0,005153038	0,005153038

t_Sulfate	0	0
t_Cu2+	0,003435359	0,003435359
t_Mg	0,008587254	0,008587254
t_Ca2+	0,005153038	0,005153038
t_NH3	-1,509648261	0
t_H2O	-20,16041787	8,999869877
t_Biomass	-1,142074006	-1,142074
t_Butyrate	0	0
t_D-Lactate	-9,918502375	0
t_Formate	-14,30404867	0
t_H2	0	0,5
t_L-Lactate	-9,918502375	0
t_Nitrite	0	0,1
t_Phosphate	1,517653029	2,011966651
t_Propionate	-1,521292828	0
t_O2	0	0
t_D-Glucose	0	0,5
t_CO2	-14,30353931	0
t_Acetate	-15,92933534	0
t_Succinate	-7,151769654	0
t_(S,S)-2,3-Butanediol	0	0
t_H2S	0	0
Ex_Cl	-0,005153038	-0,005153038
Ex_Sulfate	0	0
Ex_Cu2+	-0,003435359	-0,003435359
Ex_Mg	-0,008587254	-0,008587254
Ex_Ca2+	-0,005153038	-0,005153038
Ex_NH3	0	1,509648261
Ex_H2O	-8,999869877	20,16041787
Ex_Biomass	1,142074	1,142074006
Ex_Butyrate	0	0
Ex_D-Lactate	0	9,918502375
Ex_Formate	0	14,30404867
Ex_H2	-0,5	0
Ex_L-Lactate	0	9,918502375
Ex_Nitrite	-0,1	0
Ex_Phosphate	-2,011966651	-1,517653029
Ex_Propionate	0	1,521292828
Ex_O2	0	0
Ex_D-Glucose	-0,5	0
Ex_CO2	0	14,30353931
Ex_Acetate	0	15,92933534
Ex_Succinate	0	7,151769654
Ex_(S,S)-2,3-Butanediol	0	0
Ex_H2S	0	0
t_Fe2	0,007983097	0,007983097
t_fe3	0,007728415	0,007728415
t_Acetaldehyde	-1,302559388	0
t_Adenosine	0	0,494313614
t_AMP	0	0,494313614

t_Amylotriose	0	0
t_BIOT	0	0
t_Choline	0	0
t_Cytidine	0	0
t_Cytosine	0	0
t_DAlanine	0	0
t_Deoxyadenosine	0	0,494313614
t_Deoxycytidine	0	0,365829146
t_Deoxyguanosine	0	0
t_Deoxyinosine	0	0
t_Deoxyuridine	0	0
t_DRibose	0	0,5
t_DSerine	0	0
t_GLUM	0	0
t_Glycerol	0	0
t_GSH	0	0
t_Guanine	0	0
t_H2S2O3	0	0
t_Heme	0,000254683	0,000254683
t_Homocysteine	0	0
t_HYXN	0	0,494313614
t_Inosine	0	0,494313614
t_LACT	0	0
t_LAlanine	-1,009648261	0,5
t_LArabinose	0	0,5
t_LArginine	-0,015260016	0,328232069
t_LAsparagine	-0,193876201	0,5
t_LAspartate	-0,193876201	0,5
t_LCysteine	0,106585198	0,5
t_LGlutamate	-0,53047625	0,5
t_LGlutamine	-0,254824131	0,5
t_LHistidine	0,105185015	0,105185016
t_LInositol	0	0
t_LIsoleucine	-1,187240771	0,322407492
t_LLeucine	0,499999997	0,5
t_LLysine	-0,313108729	0,380767474
t_LMethionine	-0,222139106	0,171275697
t_LPhenylalanine	-1,304074941	0,205573321
t_LThreonine	-1,009648261	0,5
t_LTryptophan	0,063076747	0,063076747
t_LTyrosine	-1,356610345	0,153037917
t_LValine	-1,039227981	0,470420283
t_Maltose	0	0,5
t_Niacin	0,002602787	0,002602787
t_Ornithine	0	0
t_PPi	0	0
t_XAN	0	0
t_(R)3Hydroxybutanoate	0	0
t_5Deoxyadenosine	0	0
t_Acetoacetate	-5,149569257	0

t_Calomide	0	0
t_Cbl	0	0
t_Citrate	0	0
t_CysGly	0	0
t_Dulcose	0	0
t_Glycine	-1,009648261	0,5
t_LProline	0,245317495	0,245317497
t_Maltohexaose	0	0
t_Methanol	0	0
t_NAcetylDglucosamine	0	0
t_Putrescine	0	0
t_Pyridoxal	0,000254683	0,000254683
t_Riboflavin	0,000509365	0,000509365
t_Salicin	0	0
t_Sorbitol	0	0
t_Spermidine	0	0
t_Sucrose	0	0,5
t_Taurine	0	0
t_Thiamin	0	0
t_Thymidine	0	0
t_Thyminose	0	0,5
t_TRHL	0	0
t_Uracil	0	0,365829146
t_Uridine	0	0,365829146
t_Ursin	0	0
t_Mn2+	0,003435359	0,003435359
t_Formaldehyde	0	0
t_Fumarate	-7,151769654	0
t_Oxidized glutathione	0	0
t_Adenine	0	0
t_Nicotinamide	0	0
t_Co2+	0,003435359	0,003435359
t_D-Glutamate	0	0
t_Chorismate	0	0
t_Folate	0,00101873	0,00101873
t_N-Acetyl-D-mannosamine	0	0
t_Siroheme	0	0
t_Menaquinone 7	0	0
t_2-Demethylmenaquinone 8	0	0
t_Menaquinone 8	0	0
t_Ubiquinone-8	0	0
t_2-Oxobutyrate	0	0
t_3MOP	0	0
t_ABEE	0	0
t_Neu5Ac	0	0
t_Glycerol-3-phosphate	0	0
t_H+	-1000	0,5
t_Nicotinamide ribonucleotide	0	0
t_PAN	0,000657835	0,000657835
t_Pyridoxal phosphate	0	0

t_Zn2+	0,003435359	0,003435359
t_1,2-Diacyl-sn-glycerol dioctadecanoyl	0	0
t_meso-2,6-Diaminopimelate	0	0
t_L-Serine	-1,009648261	0,5
t_D-Fructose	0	0,5
t_D-Mannose	0	0,5
t_Cholesterol	0	0
t_beta D-Galactose	0	0
t_L-Fucose	0	0
Ex_Fe2	-0,007983097	-0,007983097
Ex_fe3	-0,007728415	-0,007728415
Ex_Acetaldehyde	0	1,302559388
Ex_Adenosine	-0,494313614	0
Ex_AMP	-0,494313614	0
Ex_Amylotriose	0	0
Ex_BIOT	0	0
Ex_Choline	0	0
Ex_Cytidine	0	0
Ex_Cytosine	0	0
Ex_DAlanine	0	0
Ex_Deoxyadenosine	-0,494313614	0
Ex_Deoxycytidine	-0,365829146	0
Ex_Deoxyguanosine	0	0
Ex_Deoxyinosine	0	0
Ex_Deoxyuridine	0	0
Ex_DRibose	-0,5	0
Ex_DSerine	0	0
Ex_GLUM	0	0
Ex_Glycerol	0	0
Ex_GSH	0	0
Ex_Guanine	0	0
Ex_Heme	-0,000254683	-0,000254683
Ex_Homocysteine	0	0
Ex_HYXN	-0,494313614	0
Ex_Inosine	-0,494313614	0
Ex_LACT	0	0
Ex_LAlanine	-0,5	1,009648261
Ex_LArabinose	-0,5	0
Ex_LArginine	-0,328232069	0,015260016
Ex_LAsparagine	-0,5	0,193876201
Ex_LAspartate	-0,5	0,193876201
Ex_LCysteine	-0,5	-0,106585198
Ex_LGlutamate	-0,5	0,53047625
Ex_LGlutamine	-0,5	0,254824131
Ex_LHistidine	-0,105185016	-0,105185015
Ex_LInositol	0	0
Ex_LIsoleucine	-0,322407492	1,187240771
Ex_LLeucine	-0,5	-0,499999997
Ex_LLysine	-0,380767474	0,313108729
Ex_LMethionine	-0,171275697	0,222139106

Ex_LPhenylalanine	-0,205573321	1,304074941
Ex_LThreonine	-0,5	1,009648261
Ex_LTryptophan	-0,063076747	-0,063076747
Ex_LTyrosine	-0,153037917	1,356610345
Ex_LValine	-0,470420283	1,039227981
Ex_Maltose	-0,5	0
Ex_Niacin	-0,002602787	-0,002602787
Ex_Ornithine	0	0
Ex_PPi	0	0
Ex_XAN	0	0
Ex_(R)3Hydroxybutanoate	0	0
Ex_5Deoxyadenosine	0	0
Ex_Acetoacetate	0	5,149569257
Ex_Calomide	0	0
Ex_Cbl	0	0
Ex_Citrate	0	0
Ex_CysGly	0	0
Ex_Dulcose	0	0
Ex_Glycine	-0,5	1,009648261
Ex_LProline	-0,245317497	-0,245317495
Ex_Maltohexaose	0	0
Ex_Methanol	0	0
Ex_NAcetylDglucosamine	0	0
Ex_Putrescine	0	0
Ex_Pyridoxal	-0,000254683	-0,000254683
Ex_Riboflavin	-0,000509365	-0,000509365
Ex_Salicin	0	0
Ex_Sorbitol	0	0
Ex_Spermidine	0	0
Ex_Sucrose	-0,5	0
Ex_Taurine	0	0
Ex_Thiamin	0	0
Ex_Thymidine	0	0
Ex_Thyminose	-0,5	0
Ex_TRHL	0	0
Ex_Uracil	-0,365829146	0
Ex_Uridine	-0,365829146	0
Ex_Ursin	0	0
Ex_Mn2+	-0,003435359	-0,003435359
Ex_Formaldehyde	0	0
Ex_Fumarate	0	7,151769654
Ex_Oxidized glutathione	0	0
Ex_Adenine	0	0
Ex_Nicotinamide	0	0
Ex_Co2+	-0,003435359	-0,003435359
Ex_D-Glutamate	0	0
Ex_Folate	-0,00101873	-0,00101873
Ex_N-Acetyl-D-mannosamine	0	0
Ex_Siroheme	0	0
Ex_Menaquinone 7	0	0

Ex_2-Demethylmenaquinone 8	0	0
Ex_Menaquinone 8	0	0
Ex_Ubiquinone-8	0	0
Ex_ABEE	0	0
Ex_Neu5Ac	0	0
Ex_H+	-0,5	1000
Ex_Nicotinamide ribonucleotide	0	0
Ex_PAN	-0,000657835	-0,000657835
Ex_Zn2+	-0,003435359	-0,003435359
Ex_1,2-Diacyl-sn-glycerol dioctadecanoyl	0	0
Ex_L-Serine	-0,5	1,009648261
Ex_D-Fructose	-0,5	0
Ex_D-Mannose	-0,5	0
Ex_Cholesterol	0	0
Ex_beta D-Galactose	0	0
Ex_L-Fucose	0	0
t_Starch	0	0,005
t_octanoate	0	0
t_Melibiose	0	0
t_Amylose	0	0
Ex_Starch	-0,005	0
Ex_Melibiose	0	0
Ex_Amylose	0	0
t_Raffinose_Melitose	0	0
t_Isovaleric_acid	0	0
t_H2O2	0	0
Ex_Raffinose_Melitose	0	0
Ex_Isovaleric_acid	0	0
Ex_H2O2	0	0
rxn01207_1	0	0
rxn08972	0	0
rxn08973	0	0
rxn06111	0	191,0722117
rxn13726	0	0
rxn13727	0	0
rxn13729	0	0
rxn08974	0	0
rxn10122	0	0
rxn10123	0	0
rxn10124	0	0
rxn12665	0	0
rxn06097	0	0,005
t_Sulfite	0	0
Ex_Sulfite	0	0

rxn ID	minFlux	max Flux
rxn00001	0	1000
rxn00003	-1,04549995	0
rxn00006	0	0
rxn00011	-1,04549995	0
rxn00016	0	0
rxn00018	0	0
rxn00020	0	0
rxn00022	0	0,400770971
rxn00029	0,00101873	0,00101873
rxn00048	0	0,000509365
rxn00056	0	0
rxn00060	0,000254683	0,000254683
rxn00062	0	1000
rxn00063	0	1,465433417
rxn00065	0	1,465433417
rxn00066	0	0
rxn00067	0	0
rxn00076	0	1,465433417
rxn00077	0	0,000510507
rxn00085	-1000	0
rxn00100	0,000657835	0,000657835
rxn00101	0	0,904484641
rxn00102	-1000	-3,54043E-05
rxn00104	-1000	0
rxn00105	-1,465433417	1,465433417
rxn00106	-1000	0
rxn00109	0	0
rxn00114	-999,9999646	0,732716709
rxn00119	0,368989262	1000
rxn00121	-0,000254683	0
rxn00122	0	0,000254683
rxn00124	0,000254683	0,000254683
rxn00126	0,008466195	0,008466195
rxn00127	0,007702147	0,007702147
rxn00131	-1000	0,962398929
rxn00132	0	1000
rxn00137	0	1,465433417
rxn00139	-1,465433417	0
rxn00140	0	1,465433417
rxn00141	-999,9994906	0
rxn00142	0	0
rxn00143	0,000509365	1000
rxn00144	0	0
rxn00148	-1,465433417	0
rxn00151	-1,975965624	-0,510022839
rxn00154	0	2,023298889
rxn00157	-2,023298889	0
rxn00159	-1000	1000
rxn00161	-1000	1000

rxn00162	0	1,465433417
rxn00165	0	0,079866382
rxn00171	0	1,303068753
rxn00173	0	3,766594916
rxn00178	-2,156692285	0
rxn00179	0	0
rxn00184	-1000	0
rxn00187	0	1000
rxn00189	0	1000
rxn00190	0,002602787	1,468036204
rxn00192	0	3,766594916
rxn00193	-1,304702494	2,184029813
rxn00194	0,000436272	2,157128558
rxn00196	0	0
rxn00198	0	8,743625546
rxn00199	0	8,743625546
rxn00200	0	0
rxn00202	0	0
rxn00211	0	0
rxn00212	0	999,6310107
rxn00213	-1000	1,064291346
rxn00214	-0,650770971	0
rxn00216	0	1000
rxn00221	0	1000
rxn00222	0	0
rxn00224	0,000254683	1000
rxn00225	-3,766594916	0
rxn00231	0	0
rxn00239	0,238807673	1,733308017
rxn00242	0	0,746995489
rxn00251	-1000	0
rxn00254	0	0
rxn00260	-0,763564984	0,701868435
rxn00262	0	0
rxn00279	0	1,465433417
rxn00283	-2,124802997	1,363929309
rxn00290	-1,649015258	-0,000690955
rxn00293	0,062989949	999,6940007
rxn00297	0	0
rxn00299	0	0
rxn00300	0	0,000509365
rxn00301	0	1,493990978
rxn00302	0	0
rxn00303	0	0,746995489
rxn00304	-1,465433417	0
rxn00305	0	1,493990978
rxn00307	0	0
rxn00313	0	1,011649445
rxn00322	0	0
rxn00333	0	0

rxn00337	0,031749657	1,497183074
rxn00338	0	0
rxn00340	0	1,465433417
rxn00342	0	1,697959686
rxn00346	0	0,000657835
rxn00350	-0,000254683	-0,000254683
rxn00358	0	0
rxn00359	0	1,465433417
rxn00360	0	1000
rxn00361	0	1000
rxn00362	-1000	1000
rxn00363	0	1000
rxn00364	-969,6533572	1,465433417
rxn00365	0	1000
rxn00367	0	969,8797582
rxn00368	0	1000
rxn00369	0	1,493990978
rxn00371	0	1000
rxn00379	0	1,465433417
rxn00388	0	0
rxn00391	0	999,9997453
rxn00392	0,000254683	1000
rxn00394	0	0,904484641
rxn00405	0	0
rxn00410	-999,8225103	969,8308469
rxn00411	-1,465433417	0
rxn00412	0	1000
rxn00414	0	999,9999646
rxn00416	0	1,465433417
rxn00423	0	0,079866382
rxn00426	0	0
rxn00427	0	0
rxn00433	0	0
rxn00436	0	1,465433417
rxn00437	0	0
rxn00440	0,000254683	1,4656881
rxn00449	0	0
rxn00453	0	0
rxn00459	0	1,364598371
rxn00460	-1,465433417	0
rxn00461	0,031494975	0,031494975
rxn00463	0	999,6310107
rxn00469	0	3,766594916
rxn00470	0,045698949	0,045698949
rxn00474	0	0
rxn00490	0	0
rxn00493	-0,560848276	0
rxn00499	-2,023298889	0
rxn00500	-2,023298889	0
rxn00506	0	1,303068753

rxn00512	0	0
rxn00514	0	0
rxn00517	-1,465433417	0
rxn00519	0	1000
rxn00527	-0,560848276	0
rxn00533	-999,9999646	3,54043E-05
rxn00540	0	0
rxn00543	-1,303068753	0
rxn00545	0	0
rxn00546	0	0
rxn00547	0	0,650770971
rxn00551	0	0
rxn00552	-0,06298995	999,9370101
rxn00554	0	0
rxn00555	0	1000
rxn00556	0	0
rxn00557	0	0
rxn00558	-1000	1000
rxn00559	0	0
rxn00565	0	0
rxn00566	0	1,791721686
rxn00575	0	0,400770971
rxn00585	0	0
rxn00606	0	0
rxn00608	0	0
rxn00611	-1,698978884	0
rxn00615	0	0
rxn00616	0	1,698978884
rxn00621	0	0
rxn00622	0	0
rxn00623	0	0
rxn00624	0	0
rxn00641	0	0
rxn00642	0	0
rxn00646	0	0
rxn00647	0	0
rxn00649	0	0,079866382
rxn00650	-0,000254683	-0,000254683
rxn00653	0	0
rxn00654	0	0
rxn00657	0	0
rxn00670	0	1,533134428
rxn00684	0	0
rxn00685	0	8,743625546
rxn00686	0	0
rxn00687	0	8,743625546
rxn00689	0	0
rxn00690	0	0,247079721
rxn00692	-0,260507082	-0,1806407
rxn00693	0,150629279	0,397709

rxn00695	-1000	1000
rxn00701	0	1000
rxn00704	-1000	0,650770971
rxn00707	0	1000
rxn00708	0	1000
rxn00709	0	1000
rxn00710	0	0
rxn00711	-1,465433417	0
rxn00712	0	1000
rxn00713	0	1,493990978
rxn00715	0	1000
rxn00717	0	969,1473704
rxn00726	0	0,079866382
rxn00727	0	0,079866382
rxn00735	0	0
rxn00737	0	1,683912176
rxn00741	0	0
rxn00742	-0,397199635	1,314655669
rxn00743	0	1000
rxn00744	0	1000
rxn00745	0	1,465433417
rxn00747	0	0
rxn00748	0	0
rxn00751	0	0
rxn00758	0	0
rxn00763	0	0
rxn00765	0	0
rxn00770	0,002857469	1,468290886
rxn00772	0,000509365	1000
rxn00775	0	0
rxn00777	-0,924778581	0,064263362
rxn00778	-1000	1000
rxn00779	0	1,364598371
rxn00780	0	0
rxn00784	0	1,303068753
rxn00785	-0,031367634	0,582335827
rxn00789	0	0
rxn00790	-0,000254683	-0,000254683
rxn00791	-0,079866382	0
rxn00792	0	0
rxn00796	0	0
rxn00799	-0,463714883	1,917580812
rxn00800	-0,268565555	0,229771016
rxn00802	0	0,732716709
rxn00806	0	0
rxn00808	0	1000
rxn00816	0	0,400770971
rxn00817	0	0,400770971
rxn00818	0	0
rxn00819	0	0

rxn00827	0	0
rxn00829	0,000690955	0,000690955
rxn00830	6,28141E-05	6,28141E-05
rxn00831	0	1,465433417
rxn00832	0	0
rxn00834	-1,196867864	1,733998972
rxn00836	-1,465433417	0
rxn00838	-0,268565555	0,229771016
rxn00849	-2,152534838	1,336197468
rxn00851	0	1000
rxn00855	0	0
rxn00869	0	0
rxn00871	0	1,424987633
rxn00872	-1,424987633	0
rxn00874	0	0
rxn00881	0	0
rxn00882	0	0
rxn00883	0	0
rxn00889	0	0
rxn00890	0	0
rxn00898	0	1,011649445
rxn00902	0	0
rxn00903	-1,011649445	0,000657835
rxn00907	-0,246570356	0,000509365
rxn00909	-1,424987633	0,397963683
rxn00910	-1,698978884	0
rxn00912	0	0,000657835
rxn00913	0	1,494681932
rxn00915	-1,465433417	0
rxn00916	-999,7319438	1,001282264
rxn00917	0	1000
rxn00926	0	0,497996993
rxn00929	-1000	1000
rxn00931	-1000	1000
rxn00938	0	0,732716709
rxn00947	0	1,465433417
rxn00950	-1,314655669	0,397199635
rxn00952	0	1,465433417
rxn00955	0,000509365	1000
rxn00973	-1000	1000
rxn00974	-1000	1000
rxn00977	0	0
rxn00980	0	0
rxn00983	0	0
rxn00985	-1,533134428	0
rxn00991	-0,000690955	-0,000690955
rxn00995	0	0
rxn01000	0	0,560848276
rxn01016	0	0
rxn01018	0	0

rxn01019	0	0,732716709
rxn01020	0	0
rxn01021	0,007702147	0,007702147
rxn01025	0	0
rxn01037	0	0
rxn01042	0	0
rxn01049	0	0
rxn01052	0	0
rxn01069	0	1,465433417
rxn01071	0	0
rxn01073	0	0
rxn01080	0	0
rxn01089	0	0
rxn01100	-1000	0
rxn01101	0	0
rxn01106	-1,364598371	0
rxn01108	-1000	1000
rxn01109	-1000	1000
rxn01113	0	0
rxn01114	0	0
rxn01116	-0,925797311	0,063244632
rxn01117	0	0
rxn01119	0	0
rxn01138	-999,9994906	1000
rxn01139	0	0
rxn01146	0	0
rxn01153	0	0
rxn01156	0	0
rxn01169	0	1000
rxn01171	0	1000
rxn01199	0	0
rxn01200	0	1000
rxn01201	-2,15738324	-0,000690955
rxn01204	0,000690955	2,15738324
rxn01210	0	0
rxn01211	-0,246825038	0,000509365
rxn01213	6,28141E-05	6,28141E-05
rxn01225	0	0,732716709
rxn01228	0	0
rxn01236	-1,424987633	0
rxn01237	0	0
rxn01255	0,000254683	0,561102959
rxn01256	0	0,560848276
rxn01257	0	0
rxn01259	0	0
rxn01261	0	0
rxn01265	-1,468036204	-0,002602787
rxn01268	0	0,560848276
rxn01274	0	0
rxn01280	0	0

rxn01281	0	0
rxn01286	0	0
rxn01292	0	0,5
rxn01297	-1,465433417	1,465433417
rxn01300	0	1,465433417
rxn01301	-1,4656881	0
rxn01302	-1,4656881	0
rxn01303	0,000254683	1,4656881
rxn01304	0	0
rxn01321	0	0
rxn01322	0	0
rxn01329	0	0
rxn01332	0,000254683	0,561102959
rxn01333	-0,271549033	0,291112
rxn01343	0	0
rxn01346	0	0
rxn01347	0	0
rxn01348	0	0
rxn01351	0	1000
rxn01352	-1000	-0,029248515
rxn01354	-1,465433417	0
rxn01358	0	1,465433417
rxn01361	0	0
rxn01362	0	0
rxn01366	-969,4848647	1,12793913
rxn01367	0	0
rxn01368	0	969,1473704
rxn01370	0	1000
rxn01377	0	0
rxn01379	0	0
rxn01380	0	0
rxn01387	-8,743625546	0
rxn01388	-1000	1000
rxn01389	0	0
rxn01390	0	0
rxn01396	0	0
rxn01406	0,007702147	0,007702147
rxn01423	0	0
rxn01426	0	0
rxn01434	0	0,732716709
rxn01445	0	999,9707515
rxn01446	-0,029248515	-0,029248515
rxn01452	-999,999309	0
rxn01457	0	0
rxn01459	0	0
rxn01465	0	0
rxn01466	6,28141E-05	6,28141E-05
rxn01480	0	0
rxn01484	0	0
rxn01485	-0,06298995	-0,062989949

rxn01486	0	0
rxn01492	0	0
rxn01500	-0,000690955	-0,000690955
rxn01506	0	0
rxn01509	-999,9707515	1,465433417
rxn01510	0	1000
rxn01513	0,028334856	0,028334856
rxn01517	0	0
rxn01518	0,028334856	1000
rxn01519	0	0
rxn01521	0	999,9716651
rxn01522	0	0
rxn01537	0	969,1473704
rxn01538	0	999,9997453
rxn01539	-1000	-0,000254683
rxn01544	-1,465433417	0
rxn01548	0,029248515	1,494681932
rxn01549	0	0
rxn01562	0	0
rxn01575	-0,961960745	0
rxn01594	0	0
rxn01601	0	0
rxn01602	0	0
rxn01603	0	0
rxn01610	0	0
rxn01626	0	0
rxn01629	-0,00203746	-0,00203746
rxn01636	-3,892663899	0,458934736
rxn01637	-0,458934736	0,126068983
rxn01641	0	0
rxn01643	-1,497183074	-0,031749657
rxn01644	0,031494975	1,043144419
rxn01646	0	0,732716709
rxn01647	0	1,465433417
rxn01648	-1000	1000
rxn01649	0	1,465433417
rxn01650	0	0
rxn01653	0	0
rxn01654	0	0
rxn01667	0	0
rxn01669	0	1,465433417
rxn01670	0	0,732716709
rxn01675	0	0
rxn01679	0	0
rxn01682	0	0
rxn01684	0	0
rxn01697	0	0
rxn01704	0	0
rxn01706	0	0
rxn01710	0	0

rxn01721	0	0
rxn01735	0	0
rxn01737	0	0
rxn01739	0,000254683	0,561102959
rxn01740	-0,561102959	-0,000254683
rxn01741	0	0
rxn01757	0	0
rxn01761	0	0
rxn01763	0	0,5
rxn01772	0	0
rxn01790	-0,000657835	0
rxn01791	0	0,000657835
rxn01795	0	0
rxn01799	-0,028334856	0,33749429
rxn01800	0	0,365829146
rxn01807	0	0
rxn01812	0	0
rxn01834	0	0
rxn01840	0	0
rxn01843	0	0
rxn01851	0	0
rxn01857	0	0
rxn01859	0	0,494822979
rxn01860	0	0
rxn01867	0	0
rxn01868	0	0
rxn01871	0	0
rxn01885	0	0
rxn01892	0	0
rxn01893	0	0
rxn01894	0	0
rxn01906	0	0
rxn01917	-0,126068983	0,458934736
rxn01932	0	0
rxn01937	0	0
rxn01953	0	0
rxn01961	0	1,465433417
rxn01962	0	0
rxn01964	0	0,079866382
rxn01967	0	0
rxn01972	0,031494975	3,798089891
rxn01973	-1,648324304	0
rxn01974	0,031494975	1,043144419
rxn01977	-1000	1000
rxn01982	0	0
rxn01985	0	0
rxn01986	-0,057583371	0,803068753
rxn01987	-0,5	0
rxn01997	0	0
rxn02000	0	0

rxn02003	0	0
rxn02008	0,031494975	0,031494975
rxn02011	0,031494975	0,031494975
rxn02015	0	0
rxn02023	0	0
rxn02035	0	0
rxn02046	0	0
rxn02056	0	999,9997453
rxn02078	0	0
rxn02090	0	0
rxn02093	0	0
rxn02106	0	0
rxn02122	0	0
rxn02128	0	0
rxn02138	0	0
rxn02139	0	0
rxn02144	0	0
rxn02154	0	1,465433417
rxn02155	0,002602787	1,468036204
rxn02160	0	0
rxn02161	0	0
rxn02167	0	999,999309
rxn02169	0	0
rxn02171	0,000690955	2,15738324
rxn02175	0,000657835	1,466091252
rxn02176	0	1,465433417
rxn02185	-1,011649445	0,961960745
rxn02186	0	1,011649445
rxn02187	0	0
rxn02190	0	0
rxn02195	0	0
rxn02199	0	0
rxn02200	0	0
rxn02201	0	0
rxn02212	0,000254683	0,561102959
rxn02213	0,000254683	0,561102959
rxn02217	0	0
rxn02219	0	0
rxn02222	0	0
rxn02228	0	0
rxn02235	0	0
rxn02251	0	0
rxn02264	0,000254683	0,000254683
rxn02275	0	0
rxn02279	0	0
rxn02281	0	0
rxn02283	0	0
rxn02284	-0,031494975	0
rxn02285	-0,031494975	0
rxn02286	0,031494975	0,031494975

rxn02287	-8,743625546	1000
rxn02303	0	0
rxn02305	0,000254683	969,1476251
rxn02314	0	1000
rxn02315	0	970,3854161
rxn02316	0	1000
rxn02317	-1000	0
rxn02320	0	0
rxn02322	0,000690955	0,000690955
rxn02339	0	0
rxn02341	0,000657835	0,000657835
rxn02350	0	0
rxn02351	0	0
rxn02356	-1000	1000
rxn02358	-1000	1000
rxn02366	0	0
rxn02373	-1000	1000
rxn02375	0	0
rxn02380	-1000	1000
rxn02400	0	1,465433417
rxn02402	0	0
rxn02409	0	0
rxn02449	0	0,746995489
rxn02454	0	0
rxn02465	-0,458934736	0,126068983
rxn02473	0	0
rxn02474	-0,000509365	0
rxn02475	0	0,000509365
rxn02476	0,000254683	0,561102959
rxn02483	0	0
rxn02484	0	969,1473704
rxn02495	0	0
rxn02503	0	0
rxn02504	0	0
rxn02507	0	0,079866382
rxn02508	0	0,079866382
rxn02518	0	0
rxn02521	0	0
rxn02522	0	0
rxn02525	0	0
rxn02527	0	1000
rxn02528	-1000	0
rxn02569	0	0
rxn02571	0	0
rxn02581	0	0
rxn02596	0	0
rxn02597	0	0
rxn02650	0	0
rxn02718	0	0
rxn02729	0	0

rxn02749	0	0
rxn02751	0	0
rxn02760	0	0
rxn02762	0	0
rxn02774	-8,743625546	0
rxn02775	0	0
rxn02776	0	0
rxn02788	0	0
rxn02789	0	0
rxn02795	0	0
rxn02796	0	0
rxn02811	0	0
rxn02821	0	0
rxn02822	0	0
rxn02834	0	0
rxn02835	0	0
rxn02853	0	0
rxn02875	0	0
rxn02894	0,007702147	0,007702147
rxn02895	0,000254683	0,000254683
rxn02897	0	0
rxn02914	0	0
rxn02922	0	0
rxn02926	0	0
rxn02928	-1000	999,968505
rxn02929	-1000	999,968505
rxn02936	0	0
rxn02937	0,000254683	0,000254683
rxn02943	0	0
rxn02986	0	0
rxn02988	0	0
rxn02990	0	0
rxn03004	0	0,000254683
rxn03005	-0,000254683	0
rxn03024	0	0
rxn03025	0	0
rxn03026	0	0
rxn03030	0,031494975	3,798089891
rxn03031	-1,648324304	0
rxn03036	0	0
rxn03038	0	0
rxn03039	0	0
rxn03047	0	0
rxn03057	0,007702147	0,007702147
rxn03062	0	0
rxn03068	0	0
rxn03075	0	969,1473704
rxn03080	0	0,00101873
rxn03084	0,000254683	0,000254683
rxn03086	-3,798089891	-0,031494975

rxn03087	0	1,648324304
rxn03094	0	0
rxn03095	0	0
rxn03102	0	0
rxn03106	0	0
rxn03108	0,000254683	969,1476251
rxn03135	0	0
rxn03136	0	0
rxn03137	0	0
rxn03140	0	0
rxn03141	0	0
rxn03147	0	0
rxn03150	0	0
rxn03164	0,031494975	0,031494975
rxn03167	0	0
rxn03173	0	0
rxn03174	0	0
rxn03175	0	0
rxn03188	0	0
rxn03194	0	0,961960745
rxn03202	0	0
rxn03251	0	0
rxn03255	-1000	0
rxn03256	0	1000
rxn03263	0	0
rxn03264	0	0
rxn03269	0	0
rxn03272	0	0
rxn03273	0	0
rxn03282	0	0
rxn03309	-1000	0
rxn03310	0	1000
rxn03333	0	0
rxn03354	0	0
rxn03372	0	0
rxn03373	0	0
rxn03374	0	0
rxn03378	0	0
rxn03379	0	0
rxn03387	0	0
rxn03393	0	0
rxn03397	0	0
rxn03402	0	0
rxn03405	0	0
rxn03406	0	0
rxn03407	0	0
rxn03408	0,031494975	0,031494975
rxn03409	0	0
rxn03419	0	0
rxn03421	0	0

rxn03423	0	0
rxn03426	0	0
rxn03435	-0,961960745	0
rxn03436	0	0,961960745
rxn03437	0	0,961960745
rxn03445	0	0
rxn03446	0	0
rxn03462	0	0
rxn03465	0	0
rxn03467	0	0
rxn03468	0	0
rxn03481	0	0
rxn03482	0	0
rxn03483	0	0
rxn03491	0	0
rxn03492	0	0
rxn03512	0	0
rxn03513	0	0
rxn03514	0	0
rxn03535	0	0
rxn03536	0	0
rxn03537	0	0
rxn03538	0	0
rxn03540	0	0
rxn03548	0	8,743625546
rxn03549	0	0
rxn03552	0	0
rxn03553	0	0
rxn03598	0	0
rxn03599	0	0
rxn03609	0	0
rxn03634	0	0
rxn03638	0,062989949	0,06298995
rxn03641	0,000690955	2,15738324
rxn03642	0,000690955	2,15738324
rxn03675	0	0
rxn03690	0	0
rxn03838	0	0
rxn03839	0	0
rxn03841	0	0
rxn03852	0	0
rxn03861	0	0
rxn03869	0	0
rxn03870	0	0
rxn03901	0,031494975	0,031494975
rxn03902	0	0
rxn03903	0	0
rxn03904	0,031494975	0,031494975
rxn03907	0	0
rxn03908	0	0

rxn03909	0	0
rxn03910	0	0
rxn03933	0	0
rxn03958	0	0
rxn03990	0	0
rxn03991	0	0
rxn04045	0	0
rxn04046	0	0
rxn04047	0	0
rxn04048	0	0
rxn04050	0	0
rxn04051	0	1000
rxn04052	0	0
rxn04068	0	0
rxn04070	0	0
rxn04082	0	0,5
rxn04095	0	0
rxn04113	0	0
rxn04234	0	0
rxn04264	0	0
rxn04308	0	0
rxn04345	0	0
rxn04346	0	0
rxn04347	0	0
rxn04348	0	0
rxn04349	0	0
rxn04350	0	0
rxn04351	0	0
rxn04352	0	0
rxn04353	0	0
rxn04354	0	0
rxn04355	0	0
rxn04356	0	0
rxn04357	0	0
rxn04358	0	0
rxn04359	0	0
rxn04360	0	0
rxn04384	0	0
rxn04385	0	0
rxn04413	0	0
rxn04432	0	0
rxn04443	0	0
rxn04603	0	0
rxn04674	0	0
rxn04676	-0,271549033	1000
rxn04678	-1000	0,271549033
rxn04704	0	0
rxn04726	0	0
rxn04736	0	0
rxn04750	0	0

rxn04794	0	1000
rxn04822	0	0
rxn04909	0	0
rxn04930	0	0
rxn04954	-1,698978884	0
rxn04960	0	0
rxn05005	-1000	0
rxn05006	-1000	0
rxn05012	0	0
rxn05029	0	0
rxn05030	6,28141E-05	6,28141E-05
rxn05039	0	0,000509365
rxn05040	0	0,00101873
rxn05050	0	0
rxn05054	0	0
rxn05089	0	0
rxn05104	0,007702147	0,007702147
rxn05108	0,007702147	0,007702147
rxn05115	0	0
rxn05124	0	0
rxn05126	0	0
rxn05127	0	0
rxn05233	0	0
rxn05234	0	0
rxn05236	0	0
rxn05247	0	0
rxn05248	0	0
rxn05249	0	0
rxn05250	0	0
rxn05251	0	0
rxn05252	0	0
rxn05269	0	0
rxn05274	0	0
rxn05275	0	0
rxn05276	0	0
rxn05278	0	0
rxn05279	0	0
rxn05280	0	0
rxn05289	0	0
rxn05291	0	0
rxn05322	0	0
rxn05323	0	0
rxn05324	0	0
rxn05325	0	0
rxn05326	0	0
rxn05327	0	0
rxn05328	0	0
rxn05329	0	0
rxn05330	0	0
rxn05331	0	0

rxn05332	0	0
rxn05333	0	0
rxn05334	0	0
rxn05335	0	0
rxn05336	0	0
rxn05337	0	0
rxn05338	0	0
rxn05339	0	0
rxn05340	0	0
rxn05341	0	0
rxn05342	0	0
rxn05343	0	0
rxn05344	0	0
rxn05345	0	0
rxn05346	0	0
rxn05347	0	0
rxn05348	0	0
rxn05350	0	0
rxn05457	-1,465433417	0
rxn05465	0	0
rxn05733	0	0
rxn05736	0	1,465433417
rxn05740	-1000	1000
rxn05744	0	0
rxn05759	-0,5	0
rxn05760	-2,000244414	1000
rxn05763	0	0
rxn05778	0	0
rxn05779	0	0
rxn05794	-8,743625546	0
rxn05853	0	0
rxn05871	0	0
rxn05872	0	0
rxn05874	0	0
rxn05893	0	0
rxn05899	0	0
rxn05901	0	0
rxn05918	0	0
rxn05927	0	0
rxn05934	0	0
rxn05937	-1000	1000
rxn05938	-2,023298889	0
rxn05939	0,000579032	1,648903335
rxn05940	-1000	1,533134428
rxn05958	0	0
rxn05962	0	0
rxn05979	0	0
rxn05990	0	0
rxn05994	0	0
rxn06005	0	0

rxn06023	0	0
rxn06033	0	0
rxn06043	0	0
rxn06044	0	0
rxn06045	0	0
rxn06075	0	0
rxn06078	0	0
rxn06080	0	0
rxn06090	0	0
rxn06094	0	1000
rxn06096	-1000	0
rxn06108	-8,744316501	-0,000690955
rxn06109	-1,649015258	-0,000690955
rxn06139	0	0
rxn06140	0	0
rxn06168	0	0
rxn06181	0	1000
rxn06182	0	1000
rxn06194	0	0
rxn06195	0	0
rxn06196	0	0
rxn06200	0	0
rxn06201	0	0
rxn06212	0	0
rxn06217	0	0
rxn06218	0	0
rxn06219	0	0
rxn06224	0	0
rxn06231	0	0
rxn06251	0	0
rxn06280	0	0
rxn06281	0	0
rxn06285	0	0
rxn06298	0	0
rxn06300	0	0
rxn06316	0	0
rxn06328	0	0
rxn06341	0	0
rxn06347	0	0
rxn06348	0	0
rxn06373	0	0
rxn06381	0	0
rxn06394	0	0
rxn06403	0	0
rxn06425	0	0
rxn06432	0	0
rxn06434	0	0
rxn06435	0	0
rxn06437	0	0
rxn06438	0	0

rxn06439	0	0
rxn06440	0	0
rxn06441	0	0
rxn06443	0	0
rxn06444	0	0
rxn06445	0	0
rxn06446	0	0
rxn06447	0	0
rxn06448	0	0
rxn06449	0	0
rxn06485	0	0
rxn06489	0	0
rxn06500	0	0
rxn06522	0	0
rxn06538	0	0
rxn06556	0	0
rxn06581	0	0
rxn06584	0	0
rxn06591	0,00203746	0,00203746
rxn06592	0	0
rxn06595	0	0
rxn06624	0	0
rxn06641	0	0
rxn06648	0	0
rxn06664	0	0
rxn06671	0	0
rxn06672	0	1000
rxn06673	0	1000
rxn06677	0	0
rxn06678	0	0
rxn06701	0	0
rxn06726	0	0
rxn06729	0	0
rxn06731	0	0
rxn06737	0	0
rxn06751	0	0
rxn06752	0	0
rxn06760	0	0
rxn06768	0	0
rxn06823	0	0
rxn06831	0	0
rxn06860	0	0
rxn06864	0	0
rxn06882	0	0
rxn06883	0	0
rxn06887	0	0
rxn06889	0	8,743625546
rxn06890	0	0
rxn06936	0	0
rxn06937	0,00203746	0,00203746

rxn06947	0	0
rxn06979	0	0
rxn07056	0	0
rxn07099	0	0
rxn07189	0	0
rxn07193	0	0
rxn07199	0	0
rxn07267	0	0
rxn07292	0	0
rxn07405	0	0
rxn07437	0	0
rxn07438	0	0
rxn07441	0	3,766594916
rxn07452	0	0
rxn07456	0	999,9999646
rxn07466	-1000	1000
rxn07474	0	0
rxn07489	0	0
rxn07573	0	0
rxn07577	0	0
rxn07578	0	0
rxn07579	0	0
rxn07580	0	0
rxn07586	0	0
rxn07587	0	0
rxn07588	-1000	0
rxn07589	-1000	0
rxn07645	0	0
rxn07679	0	0
rxn07683	0	0
rxn07687	0	0
rxn07846	0	0
rxn07849	0	0
rxn07987	0	0
rxn07989	0	0
rxn07991	0	0
rxn07992	0	0
rxn07993	0	0
rxn07994	0	0
rxn07997	0	0
rxn07998	0	0
rxn08000	0	0
rxn08001	0	0
rxn08002	0	0
rxn08025	0	0
rxn08035	0	0
rxn08038	0	0
rxn08040	0	0
rxn08043	0	0,961960745
rxn08044	0	0

rxn08083	0	0
rxn08084	0	0
rxn08085	0	0
rxn08086	0	0
rxn08087	0	0
rxn08088	0	0
rxn08089	0	0
rxn08126	0	0
rxn08127	0	0
rxn08128	0	0
rxn08129	0	0
rxn08131	0,000254683	0,000254683
rxn08171	0	0
rxn08180	0	0
rxn08194	-938,2947409	1000
rxn08206	0	0
rxn08207	0	0
rxn08208	0	0
rxn08209	0	0
rxn08294	0	1000
rxn08295	0	1000
rxn08296	0	1000
rxn08297	0	1000
rxn08298	0	1000
rxn08299	0	1000
rxn08300	0	1000
rxn08306	0	0
rxn08307	0	0
rxn08308	0	0
rxn08309	0	0
rxn08310	0	0
rxn08311	0	0
rxn08312	0	0
rxn08352	0	0
rxn08386	0	0
rxn08390	0	0
rxn08392	0	0
rxn08394	0	0
rxn08396	0	0
rxn08398	0	0
rxn08413	0	0
rxn08433	0	0
rxn08434	0	1,465433417
rxn08435	0	0
rxn08436	0	0
rxn08437	0	0
rxn08438	0	0
rxn08448	0	0
rxn08449	0	0
rxn08451	0	0

rxn08453	0	0
rxn08454	0	1,465433417
rxn08455	0	0
rxn08456	0	0
rxn08457	0	0
rxn08519	0,057583371	0,057583371
rxn08546	0	0
rxn08547	0	1,465433417
rxn08548	0	0
rxn08549	0	0
rxn08550	0	0
rxn08551	0	0
rxn08552	0	0
rxn08571	0	1000
rxn08582	0	0,5
rxn08605	0	0
rxn08615	-1000	1000
rxn08647	0	0
rxn08668	0	0
rxn08669	0	0
rxn08764	0	0,961960745
rxn08796	0	0
rxn08797	0	1,465433417
rxn08798	0	0
rxn08799	0	1,465433417
rxn08800	0	0
rxn08801	0	1,465433417
rxn08802	0	0
rxn08803	0	0
rxn08804	0	0
rxn08805	0	0
rxn08806	0	0
rxn08807	0	0
rxn08808	0	0
rxn08809	0	0
rxn08810	0	0
rxn08811	0	0
rxn08812	0	0
rxn08813	0	0
rxn08814	0	0
rxn08815	0	0
rxn08816	0	0
rxn08817	0	0
rxn08818	0	0
rxn08819	0	0
rxn08820	0	0
rxn08821	0	0
rxn08822	0	0
rxn08823	0	0
rxn08838	0	0

rxn08839	0	0
rxn08840	0	0
rxn08841	0	0
rxn08842	0	0
rxn08843	0	0
rxn08844	0	0
rxn08845	0	0
rxn08846	0	0
rxn08847	0	0
rxn08848	0	0
rxn08849	0	0
rxn08850	0	0
rxn08851	0	0
rxn08857	0	0
rxn08889	0,000768616	0,000768616
rxn08890	0,006222019	0,006222019
rxn08891	0,000768616	0,000768616
rxn08892	-999,9854054	1000
rxn08893	-999,9923184	999,993087
rxn08894	-999,9854054	1000
rxn08897	-0,006912974	-0,006912974
rxn08926	0,000690955	0,000690955
rxn08927	-999,9984639	999,9869415
rxn08928	-999,9854054	1000
rxn08929	0,00153609	0,00153609
rxn08930	0	0
rxn08958	0,000768616	0,000768616
rxn09010	0	0
rxn09016	0	0,761619746
rxn09062	0	1000
rxn09063	0	1000
rxn09064	0	1000
rxn09065	0	1000
rxn09066	0	1000
rxn09067	0	1000
rxn09068	0	1000
rxn09101	0	0
rxn09102	0	0
rxn09103	0	0
rxn09104	0	0
rxn09105	0	0
rxn09106	0	0
rxn09107	0	0
rxn09108	0	0
rxn09109	0	0
rxn09110	0	0
rxn09111	0	0
rxn09112	0	0
rxn09113	0	0
rxn09114	0	0

rxn09176	-1000	1000
rxn09177	0	0,000657835
rxn09197	0	0
rxn09198	0	0
rxn09199	0	0
rxn09200	0	0
rxn09201	0	0
rxn09202	0	0
rxn09203	0	0
rxn09205	0	0
rxn09206	0	0
rxn09207	0	0
rxn09208	0	0
rxn09209	0	0
rxn09210	0	0
rxn09211	0	0
rxn09235	0,028334856	0,028334856
rxn09237	0,029248515	0,029248515
rxn09240	0	0,746995489
rxn09264	0	0
rxn09265	0	0
rxn09340	0	0
rxn09341	0	999,6310107
rxn09348	0	999,6310107
rxn09355	0	0
rxn09398	-1000	1,064291346
rxn09399	0	0
rxn09412	-1000	1000
rxn09445	0	0
rxn09446	0	0
rxn09447	0	0
rxn09454	0	0
rxn09455	0	0
rxn09456	0	0
rxn09473	0	0
rxn09486	0	0
rxn09502	0	1000
rxn09519	0	0
rxn09523	0	0
rxn09531	0	0
rxn09557	0,000254683	8,743880229
rxn09615	0,000690955	0,000690955
rxn09631	0,000254683	0,000254683
rxn09889	0	0
rxn09949	0	0
rxn09952	0	0
rxn09978	0	0
rxn09979	0	0
rxn09988	0	0
rxn09992	0	0

rxn09995	0	0
rxn10003	0	0,000657835
rxn10019	0	0
rxn10020	0	0
rxn10021	0	0
rxn10026	0	0
rxn10029	0	0
rxn10030	0	0
rxn10034	0	0
rxn10036	0	0
rxn10038	0	0
rxn10054	0	969,6533572
rxn10056	0	0,000510507
rxn10058	0	0,000510507
rxn10060	0	0,000510507
rxn10075	0,007702147	0,007702147
rxn10091	-1000	938,2947409
rxn10107	0	0
rxn10110	0	0
rxn10111	0	0
rxn10192	0	0
rxn10202	0	1,465433417
rxn10203	0	1,465433417
rxn10204	0	1,465433417
rxn10205	0	0
rxn10206	0	0
rxn10207	0	0
rxn10208	0	0
rxn10209	0	0
rxn10210	0	0
rxn10211	0	0
rxn10212	0	0
rxn10213	0	0
rxn10214	0	0
rxn10215	0	0
rxn10216	0	0
rxn10217	0	0
rxn10218	0	0
rxn10219	0	0
rxn10220	0	0
rxn10221	0	0
rxn10222	0	0
rxn10223	0	0
rxn10224	0	0
rxn10225	0	0
rxn10226	0	0
rxn10227	0	0
rxn10228	0	0
rxn10229	0	0
rxn10230	0	0

rxn10231	0	0
rxn10232	0	0
rxn10233	0	0
rxn10234	0	0
rxn10235	0	0
rxn10236	0	0
rxn10237	0	0
rxn10238	0	1000
rxn10239	0	1000
rxn10240	0	1000
rxn10241	0	1000
rxn10242	0	1000
rxn10243	0	1000
rxn10253	0	1000
rxn10254	0	1000
rxn10255	0	1000
rxn10256	0	1000
rxn10257	0	1000
rxn10258	0	1000
rxn10259	0	0
rxn10260	0	0
rxn10261	0	0
rxn10262	0	0
rxn10263	0	0
rxn10264	0	0
rxn10265	0	0
rxn10266	0	0
rxn10267	0	0
rxn10268	0	0
rxn10269	0	0
rxn10270	0	0
rxn10289	0	0
rxn10290	0	0
rxn10291	0	0
rxn10292	0	0
rxn10293	0	0
rxn10294	0	0
rxn10295	0	0
rxn10296	0	0
rxn10297	0	0
rxn10363	0	0
rxn10404	0	0
rxn10405	0	0
rxn10406	0	0
rxn10407	0	0
rxn10408	0	0
rxn10409	0	0
rxn10410	0	0
rxn10785	6,28141E-05	6,28141E-05
rxn10951	0,028334856	0,028334856

rxn11007	0,028334856	0,028334856
rxn11513	0	0
rxn11547	0	0
rxn11548	0	0
rxn11550	0	0
rxn11564	0	0
rxn11567	0	0
rxn11571	0	0
rxn11587	0	0
rxn11599	0	0
rxn11609	0	0
rxn11641	0	0
rxn11642	0	0
rxn11732	0	0
rxn11749	0	0
rxn11755	0	0
rxn11756	0	0
rxn11759	0	0
rxn11760	0	0
rxn11761	0	0
rxn11765	0	0
rxn11766	0	0
rxn11768	0	0
rxn11772	0	0
rxn11773	0	0
rxn11787	0	0
rxn11788	0	0
rxn11789	0	0
rxn11790	0	0
rxn11791	0	0
rxn11943	0	0
rxn11951	0	0
rxn11962	0	0
rxn11965	0	0
rxn11968	0	0
rxn11984	0	0
rxn12013	0	0
rxn12053	0	0
rxn12054	0	0
rxn12218	-1000	-0,000254683
rxn12221	0,000254683	1000
rxn12510	0,000657835	0,000657835
rxn12649	-999,9989813	0
rxn12708	0	0
rxn12778	0	0
rxn12822	-8,743625546	0
rxn13420	0,000690955	1000
rxn13421	0,000690955	1000
rxn13666	0	0
rxn13667	0	0

rxn13668	0	0
rxn13669	0	0
rxn13671	0	0
rxn13672	0	0
rxn13673	0	0
rxn13705	0	0
rxn13728	0	0
rxn13741	0	0
rxn13936	0,015363179	0,01536318
rxn13974	-2,023298889	0
rxn14014	0	0
rxn14028	0	0
rxn14043	0	0
rxn14050	0	0
rxn14054	-8,743625546	0
rxn14063	0	0
rxn14070	0	0
rxn14089	-8,743625546	0
rxn14093	0	0
rxn14120	-1000	-0,00101873
rxn14132	0	0
rxn14144	0	0
rxn14173	0	0
rxn14191	0	0
rxn14250	0	0
rxn14270	0	0
rxn14279	0	0
rxn14299	0	0
rxn14346	0	0
rxn90002	0	1000
rxn90003	0	0
rxn90004	0	0
rxn90005	-0,028845363	-0,028334856
rxn08173	0	4,371812773
rxn13688	0	0
Biomass_Bacteria	1,142074	1,142074006
t_Cl	0,005153038	0,005153038
t_Sulfate	0,004294198	0,004294198
t_Cu2+	0,003435359	0,003435359
t_Mg	0,008587254	0,008587254
t_Ca2+	0,005153038	0,005153038
t_NH3	-2,340014879	0
t_H2O	-11,70840436	-1,887599843
t_Biomass	-1,142074006	-1,142074
t_Butyrate	-1,424987633	0
t_D-Lactate	-2,023298889	0
t_Ethanol	-1,303068753	0
t_Formate	-3,296648607	0
t_H2	0	0,5
t_L-Lactate	-2,023298889	0

t_Nitrite	0	0
t_Phosphate	1,517143664	2,011966651
t_Propionate	-1,533134428	0
t_O2	0	0
t_D-Glucose	0	0,5
t_CO2	-3,296648607	0
t_Acetate	-5,955727814	-0,032440612
t_Succinate	-1,648324304	0
t_H2S	-0,247079721	0
Ex_Cl	-0,005153038	-0,005153038
Ex_Sulfate	-0,004294198	-0,004294198
Ex_Cu2+	-0,003435359	-0,003435359
Ex_Mg	-0,008587254	-0,008587254
Ex_Ca2+	-0,005153038	-0,005153038
Ex_NH3	0	2,340014879
Ex_H2O	1,887599843	11,70840436
Ex_Biomass	1,142074	1,142074006
Ex_Butyrate	0	1,424987633
Ex_D-Lactate	0	2,023298889
Ex_Ethanol	0	1,303068753
Ex_Formate	0	3,296648607
Ex_H2	-0,5	0
Ex_L-Lactate	0	2,023298889
Ex_Nitrite	0	0
Ex_Phosphate	-2,011966651	-1,517143664
Ex_Propionate	0	1,533134428
Ex_O2	0	0
Ex_D-Glucose	-0,5	0
Ex_CO2	0	3,296648607
Ex_Acetate	0,032440612	5,955727814
Ex_Succinate	0	1,648324304
Ex_H2S	0	0,247079721
t_Fe2	0,007983097	0,007983097
t_fe3	0,007728415	0,007728415
t_Acetaldehyde	-1,303068753	0
t_Adenosine	0	0,494822979
t_AMP	0	0,494822979
t_Amylotriose	0	0
t_BIOT	0	0
t_Choline	0	0
t_Cytidine	0	0
t_Cytosine	0	0
t_DAlanine	0	0
t_Deoxyadenosine	0	0,494822979
t_Deoxycytidine	0	0,365829146
t_Deoxyguanosine	0	0
t_Deoxyinosine	0	0
t_Deoxyuridine	0	0
t_DRibose	0	0,5
t_DSerine	0	0

t_Glycerol	0	0
t_GSH	0	0
t_Guanine	0	0
t_H2S2O3	0	0
t_Heme	0,000254683	0,000254683
t_Homocysteine	0	0
t_HYXN	0	0,373497744
t_Inosine	0	0,373497744
t_LACT	0	0,400770971
t_LAlanine	-1,523298889	0,5
t_LArabinose	0	0,5
t_LArginine	-0,08500372	0,5
t_LAsparagine	-0,348979843	0,5
t_LAspartate	-0,965433417	0,5
t_LCysteine	0,252920279	0,5
t_LGlutamate	-0,937794857	0,5
t_LGlutamine	-0,670007439	0,5
t_LHistidine	0,105185015	0,105185016
t_LInositol	0	0
t_LIsoleucine	-0,639553254	0,322407492
t_LLeucine	0,499999997	0,5
t_LLysine	-0,630881973	0,380767474
t_LMethionine	-0,226433304	0,020646417
t_LPhenylalanine	-0,355274956	0,205573321
t_LThreonine	-1,033134428	0,5
t_LTryptophan	-0,016789635	0,063076747
t_LTyrosine	-0,40781036	0,153037917
t_LValine	-0,541229164	0,471078118
t_Maltose	0	0,400770971
t_Niacin	0,002602787	0,002602787
t_Ornithine	0	0
t_PPi	0	0
t_Pyridoxol	0	0
t_XAN	0	0
t_1,3Propanediol	0	0
t_5Deoxyadenosine	0	0
t_Acetoacetate	-1,648324304	0
t_BET	0	0
t_Calomide	0	0
t_Cbl	0	0
t_Citrate	0	0
t_CysGly	0	0
t_Glycine	0,420133618	0,5
t_Glycolaldehyde	0	0
t_LProline	0,245317495	0,245317497
t_Maltohexaose	0	0
t_Methanol	0	0
t_NAcetylDglucosamine	0	0
t_PM	0	0
t_Putrescine	0	0

t_Pyridoxal	0,000254683	0,000254683
t_Riboflavin	0	0,000509365
t_Salicin	0	0
t_Sorbitol	0	0
t_Spermidine	0	0
t_Sucrose	0	0,400770971
t_Taurine	0	0
t_Thiamin	0	0
t_Thymidine	0	0
t_Thyminose	0	0,5
t_TRHL	0	0
t_Uracil	0	0,365829146
t_Uridine	0	0,365829146
t_Ursin	0	0
t_Mn2+	0,003435359	0,003435359
t_Formaldehyde	0	0
t_Fumarate	-1,648324304	0
t_Oxidized glutathione	0	0
t_Adenine	0	0
t_Nicotinamide	0	0
t_Co2+	0,003435359	0,003435359
t_D-Glutamate	0	0
t_Nitrate	0	0
t_Chorismate	0	0
t_Folate	0,00101873	0,00101873
t_N-Acetyl-D-mannosamine	0	0
t_Siroheme	0	0
t_Selenate	0	0
t_Menaquinone 7	0	0
t_2-Demethylmenaquinone 8	0	0
t_Menaquinone 8	0	0
t_Ubiquinone-8	0	0
t_2-Oxobutyrate	0	0
t_3MOP	0	0
t_ABEE	0	0
t_Neu5Ac	0	0
t_Glycerol-3-phosphate	0	0
t_H+	-15,37547858	0,5
t_indol	0	0
t_Nicotinamide ribonucleotide	0	0
t_PAN	0	0,000657835
t_Pyridoxal phosphate	0	0
t_Zn2+	0,003435359	0,003435359
t_1,2-Diacyl-sn-glycerol dioctadecanoyl	0	0
t_meso-2,6-Diaminopimelate	0	0
t_L-Serine	0,420133618	0,5
t_D-Fructose	0	0,5
t_D-Mannose	0	0
t_D-Mannitol	0	0
t_L-Rhamnose	0	0

t_beta D-Galactose	0	0,5
t_L-Fucose	0	0
Ex_Fe2	-0,007983097	-0,007983097
Ex_fe3	-0,007728415	-0,007728415
Ex_Acetaldehyde	0	1,303068753
Ex_Adenosine	-0,494822979	0
Ex_AMP	-0,494822979	0
Ex_Amylotriose	0	0
Ex_BIOT	0	0
Ex_Choline	0	0
Ex_Cytidine	0	0
Ex_Cytosine	0	0
Ex_DAlanine	0	0
Ex_Deoxyadenosine	-0,494822979	0
Ex_Deoxycytidine	-0,365829146	0
Ex_Deoxyguanosine	0	0
Ex_Deoxyinosine	0	0
Ex_Deoxyuridine	0	0
Ex_DRibose	-0,5	0
Ex_DSerine	0	0
Ex_Glycerol	0	0
Ex_GSH	0	0
Ex_Guanine	0	0
Ex_Heme	-0,000254683	-0,000254683
Ex_Homocysteine	0	0
Ex_HYXN	-0,373497744	0
Ex_Inosine	-0,373497744	0
Ex_LACT	-0,400770971	0
Ex_LAlanine	-0,5	1,523298889
Ex_LArabinose	-0,5	0
Ex_LArginine	-0,5	0,08500372
Ex_LAsparagine	-0,5	0,348979843
Ex_LAspartate	-0,5	0,965433417
Ex_LCysteine	-0,5	-0,252920279
Ex_LGlutamate	-0,5	0,937794857
Ex_LGlutamine	-0,5	0,670007439
Ex_LHistidine	-0,105185016	-0,105185015
Ex_LInositol	0	0
Ex_LIsoleucine	-0,322407492	0,639553254
Ex_LLeucine	-0,5	-0,499999997
Ex_LLysine	-0,380767474	0,630881973
Ex_LMethionine	-0,020646417	0,226433304
Ex_LPhenylalanine	-0,205573321	0,355274956
Ex_LThreonine	-0,5	1,033134428
Ex_LTryptophan	-0,063076747	0,016789635
Ex_LTyrosine	-0,153037917	0,40781036
Ex_LValine	-0,471078118	0,541229164
Ex_Maltose	-0,400770971	0
Ex_Niacin	-0,002602787	-0,002602787
Ex_Ornithine	0	0

Ex_PPi	0	0
Ex_XAN	0	0
Ex_5Deoxyadenosine	0	0
Ex_Acetoacetate	0	1,648324304
Ex_BET	0	0
Ex_Calomide	0	0
Ex_Cbl	0	0
Ex_Citrate	0	0
Ex_CysGly	0	0
Ex_Glycine	-0,5	-0,420133618
Ex_Glycolaldehyde	0	0
Ex_LProline	-0,245317497	-0,245317495
Ex_Maltohexaose	0	0
Ex_Methanol	0	0
Ex_NAcetylDglucosamine	0	0
Ex_PM	0	0
Ex_Putrescine	0	0
Ex_Pyridoxal	-0,000254683	-0,000254683
Ex_Riboflavin	-0,000509365	0
Ex_Salicin	0	0
Ex_Sorbitol	0	0
Ex_Spermidine	0	0
Ex_Sucrose	-0,400770971	0
Ex_Taurine	0	0
Ex_Thiamin	0	0
Ex_Thymidine	0	0
Ex_Thyminose	-0,5	0
Ex_TRHL	0	0
Ex_Uracil	-0,365829146	0
Ex_Uridine	-0,365829146	0
Ex_Ursin	0	0
Ex_Mn2+	-0,003435359	-0,003435359
Ex_Formaldehyde	0	0
Ex_Fumarate	0	1,648324304
Ex_Oxidized glutathione	0	0
Ex_Adenine	0	0
Ex_Nicotinamide	0	0
Ex_Co2+	-0,003435359	-0,003435359
Ex_D-Glutamate	0	0
Ex_Nitrate	0	0
Ex_Folate	-0,00101873	-0,00101873
Ex_N-Acetyl-D-mannosamine	0	0
Ex_Siroheme	0	0
Ex_Selenate	0	0
Ex_Menaquinone 7	0	0
Ex_2-Demethylmenaquinone 8	0	0
Ex_Menaquinone 8	0	0
Ex_Ubiquinone-8	0	0
Ex_ABEE	0	0
Ex_Neu5Ac	0	0

Ex_H+	-0,5	15,37547858
Ex_indol	0	0
Ex_Nicotinamide ribonucleotide	0	0
Ex_PAN	-0,000657835	0
Ex_Zn2+	-0,003435359	-0,003435359
Ex_1,2-Diacyl-sn-glycerol dioctadecanoyl	0	0
Ex_L-Serine	-0,5	-0,420133618
Ex_D-Fructose	-0,5	0
Ex_D-Mannose	0	0
Ex_D-Mannitol	0	0
Ex_L-Rhamnose	0	0
Ex_beta D-Galactose	-0,5	0
Ex_L-Fucose	0	0
t_Arabinan	0	0
t_Starch	0	0,005
t_octanoate	0	0
t_Melibiose	0	0,400770971
t_Amylose	0	0
Ex_Arabinan	0	0
Ex_Starch	-0,005	0
Ex_Melibiose	-0,400770971	0
Ex_Amylose	0	0
t_Raffinose_Melitose	0	0
t_Isovaleric_acid	0	0
t_H2O2	0	0
t_Nitric_oxide	0	0
Ex_Raffinose_Melitose	0	0
Ex_Isovaleric_acid	0	0
Ex_H2O2	0	0
Ex_Nitric_oxide	0	0
rxn01207_1	0	0
rxn08972	0	0
rxn08973	0	0
rxn06111	0	8,743625546
rxn13726	0	0
rxn13727	0	0
rxn13729	0	0
rxn08974	0	0
rxn10122	0	0
rxn10123	0	0
rxn10124	0	0
rxn12665	0	0
rxn06097	0	0,005
t_Sulfite	0	0
Ex_Sulfite	0	0

rxn ID	minFlux	max Flux
rxn00001	0	1000
rxn00003	-1,987438566	0
rxn00011	-1,987438566	0
rxn00016	0	0
rxn00020	0	1000
rxn00022	0	0,505
rxn00029	0,000809497	0,000809498
rxn00031	0	0
rxn00060	0,000202374	0,000202374
rxn00062	0	1000
rxn00063	0	1000
rxn00066	0	0
rxn00076	0	1000
rxn00077	0	0,000405656
rxn00085	-1000	0
rxn00097	-1000	1000
rxn00100	0,000522725	0,000522725
rxn00104	-1000	0
rxn00105	-999,9979318	1000
rxn00106	-1000	0
rxn00109	0	0
rxn00119	0,293204037	10,18515191
rxn00121	-0,000202374	0
rxn00122	0	0,000202374
rxn00124	0,000202374	0,000202374
rxn00126	0,000607123	1000
rxn00131	-1000	999,9973247
rxn00132	0	1000
rxn00137	0	0
rxn00138	0	1000
rxn00139	-999,9977294	0
rxn00143	0,000404749	999,9997976
rxn00148	-9,891425144	0
rxn00151	-9,891425144	0
rxn00157	-10,97559197	0
rxn00159	-1000	1000
rxn00161	-1000	1000
rxn00162	0	0,469288114
rxn00165	0	5,76560823
rxn00170	0	16,06852057
rxn00173	0	16,06852057
rxn00178	-0,94214122	0
rxn00179	0	0
rxn00184	-1000	0
rxn00187	0	1000
rxn00189	0	1000
rxn00190	0	1000
rxn00192	0	16,06852057
rxn00193	0,025026348	0,025026363

rxn00196	0	0
rxn00198	0	1000
rxn00199	0	1000
rxn00200	0	0
rxn00206	0	0
rxn00212	0	9,891425144
rxn00213	0	9,891425144
rxn00214	-1	0
rxn00216	0	1000
rxn00221	0	1000
rxn00222	0	1000
rxn00225	-16,06852057	0
rxn00227	0	16,06852057
rxn00239	0,189759923	10,10442635
rxn00247	0	0,469288114
rxn00254	0	0
rxn00256	-0,469288114	0
rxn00258	-0,23467219	-2,81327E-05
rxn00260	2,81327E-05	1000
rxn00283	0,022036109	0,022036122
rxn00290	-5,356722565	-0,000549042
rxn00293	0,050052696	9,94147784
rxn00301	0	9,914666424
rxn00303	0	0
rxn00304	-9,891425144	0
rxn00305	0	0,469288114
rxn00313	0	0,469288114
rxn00322	0	0
rxn00328	0	0
rxn00333	0	0
rxn00337	0,031146582	0,500434697
rxn00340	0	0,469288114
rxn00350	-0,000202374	-0,000202374
rxn00358	0	0
rxn00359	0	0
rxn00360	0	0
rxn00362	-1000	1000
rxn00363	0	1000
rxn00364	-9,891947868	9,891947868
rxn00365	0	1000
rxn00367	0	9,891425144
rxn00368	0	9,891425144
rxn00369	0	9,914666424
rxn00388	0	0
rxn00391	0	999,9997976
rxn00392	0,000202374	1000
rxn00405	0	0
rxn00410	-999,8589642	10,03298369
rxn00411	-9,891947868	0
rxn00412	0	1000

rxn00414	0	0,234644057
rxn00416	0	0,469288114
rxn00423	0	5,76560823
rxn00426	0	0
rxn00433	0	0
rxn00436	0	999,9997976
rxn00437	0	0
rxn00440	0,000202374	1000
rxn00452	0	999,9993929
rxn00453	0	999,9997976
rxn00456	0	999,9997976
rxn00459	-0,101838187	9,78883651
rxn00460	-10,21017825	-0,318230385
rxn00461	0,025026348	0,025026363
rxn00463	0	9,891425144
rxn00469	0	16,06852057
rxn00470	0,036659693	5,828125198
rxn00479	0	0
rxn00490	0	0
rxn00493	-1,987438566	0
rxn00500	-10,71234705	0
rxn00506	0	0,637725312
rxn00510	0	0
rxn00512	0	0
rxn00514	0	0
rxn00517	-9,891425144	0
rxn00519	0	0,469288114
rxn00527	-1,987438566	0
rxn00533	-0,234644057	0
rxn00536	-1000	1000
rxn00541	-0,637725312	0
rxn00543	-1000	1000
rxn00545	0	1000
rxn00547	0	1
rxn00549	0	1000
rxn00551	0	1000
rxn00552	-0,050052726	999,9499473
rxn00554	0	9,891425144
rxn00555	0	1000
rxn00556	0	9,891425144
rxn00557	0	1000
rxn00558	-1000	1000
rxn00562	0	0
rxn00565	0	0
rxn00566	0	1000
rxn00575	0	0,5
rxn00585	0	0
rxn00608	0	1000
rxn00611	-1,367181966	0
rxn00615	0	0

rxn00616	0	1,367181966
rxn00622	0	0
rxn00634	0	0
rxn00647	0	0
rxn00649	0	5,76560823
rxn00650	-0,000202374	-0,000202374
rxn00653	0	0
rxn00670	0	0,91422683
rxn00684	0	0
rxn00685	0	999,9991905
rxn00686	0	0
rxn00687	0	999,9991905
rxn00689	0	0
rxn00690	0	10,97599672
rxn00692	-0,309695572	5,455912658
rxn00693	0	0,418718138
rxn00695	-1000	1000
rxn00698	-1000	0
rxn00701	0	10,89142514
rxn00702	0	0
rxn00704	-1000	1
rxn00707	0	1000
rxn00708	0	1000
rxn00709	0	1000
rxn00710	0	0
rxn00711	-999,9977294	0
rxn00712	0	9,891425144
rxn00713	0	9,914666424
rxn00715	0	1000
rxn00735	0	0
rxn00737	0	0,914024456
rxn00742	-1000	0,000202374
rxn00743	0	0
rxn00747	-4,811564539	0,175156454
rxn00748	0	0
rxn00758	0	0
rxn00763	0	0
rxn00765	0	0
rxn00770	0,002270585	1000
rxn00772	0,000404749	1000
rxn00775	0	0
rxn00777	-1,089143198	0,33181961
rxn00778	-1000	1000
rxn00781	-0,101838187	9,78883651
rxn00784	0	0,637725312
rxn00785	-0,165707431	1,538493256
rxn00786	-0,175156454	4,645654734
rxn00789	0	0
rxn00790	-0,000202374	-0,000202374
rxn00792	0	0

rxn00796	0	0
rxn00799	-0,255737869	5,654462023
rxn00800	-0,21300133	0,256286912
rxn00801	0	0
rxn00802	0	0,234644057
rxn00806	0	0
rxn00808	0	1000
rxn00816	0	0,5
rxn00829	0,000549042	0,000549043
rxn00830	4,99129E-05	4,9913E-05
rxn00831	0	999,9977294
rxn00832	0	0
rxn00834	-999,7847282	1000
rxn00836	-999,9977294	0
rxn00838	-0,21300133	0,256286912
rxn00851	0	1000
rxn00853	0	0
rxn00856	0,006466902	5,797932407
rxn00858	0	0
rxn00871	0	0,94214122
rxn00872	-0,94214122	0
rxn00874	0	0
rxn00881	0	0
rxn00882	0	0
rxn00883	0	0
rxn00889	0	0
rxn00898	0	1,987438566
rxn00902	0	0
rxn00903	-1,987438566	0
rxn00907	-10,97559197	0,000404749
rxn00908	-5,957016388	0,129489894
rxn00909	-0,948261454	0,418920513
rxn00910	-1,367181966	0
rxn00913	0	1000
rxn00915	-999,9767587	0
rxn00916	-999,7869988	0,21300133
rxn00917	0	1000
rxn00926	0	0,469288114
rxn00929	-1000	1000
rxn00931	-1000	1000
rxn00947	0	1000
rxn00950	-1000	0,41831339
rxn00952	0	999,9997976
rxn00955	0,000404749	999,9997976
rxn00973	-1000	1000
rxn00974	-1000	1000
rxn00980	0	0
rxn00983	0	0
rxn00985	-0,91422683	0
rxn00991	-0,000549043	-0,000549042

rxn01000	0	1,987438566
rxn01016	0	0
rxn01018	0	0
rxn01019	0	0,234644057
rxn01021	0	0
rxn01034	0	0
rxn01037	0	0
rxn01042	0	0
rxn01048	0	0
rxn01049	0	1000
rxn01052	0	0
rxn01069	0	0
rxn01073	0	0
rxn01080	0	0
rxn01089	0	0
rxn01100	-1000	0
rxn01101	0	0
rxn01103	0	1000
rxn01106	-9,78883651	0,101838187
rxn01108	-1000	1000
rxn01109	-1000	1000
rxn01116	-1,089143198	0,33181961
rxn01122	0	0
rxn01123	0	0
rxn01138	0	1000
rxn01139	0	0
rxn01146	0	0
rxn01169	0	1000
rxn01171	-1000	1000
rxn01199	0	0
rxn01200	0	1000
rxn01201	-5,792014547	-0,000549042
rxn01204	0,000549042	5,792014547
rxn01210	0	0
rxn01211	-10,97579435	0,000404749
rxn01213	4,99129E-05	4,9913E-05
rxn01228	0	0
rxn01236	-0,94214122	0
rxn01255	0,000202374	1,98764094
rxn01256	0	1,987438566
rxn01257	0	0
rxn01261	0	0
rxn01265	-999,9997976	-0,002068211
rxn01268	0	1,987438566
rxn01274	0	0
rxn01276	0	0
rxn01278	0	0
rxn01286	0	0
rxn01297	-999,7869988	999,9977294
rxn01300	0	0

rxn01303	0	0
rxn01304	0	0
rxn01305	0	0
rxn01322	0	0
rxn01332	0,000202374	1,98764094
rxn01334	0	0,746743857
rxn01343	0	0,746743857
rxn01346	0	0,746743857
rxn01347	0	0,746743857
rxn01348	0	0,746743857
rxn01351	0	1000
rxn01352	-1000	-0,02324128
rxn01354	-9,891425144	0
rxn01355	0	0
rxn01358	0	999,9977294
rxn01361	0	0
rxn01362	0	0
rxn01366	-0,26817785	1000
rxn01367	0	0
rxn01368	0	9,891947868
rxn01370	0	1000
rxn01377	0	0
rxn01380	0	0
rxn01387	-1000	0
rxn01388	-1000	1000
rxn01396	0	0
rxn01406	0	0
rxn01423	0	0
rxn01434	0	0,234644057
rxn01445	0	999,9767587
rxn01446	-0,023241294	-0,02324128
rxn01452	-999,999451	0
rxn01455	0	0
rxn01457	0	0
rxn01459	0	5,791812173
rxn01465	0	0
rxn01466	4,99129E-05	4,9913E-05
rxn01484	0	0
rxn01485	-0,050052726	-0,050052696
rxn01486	0	0
rxn01492	0	0
rxn01500	-0,000549043	-0,000549042
rxn01506	0	0
rxn01509	-999,9767587	19,68026165
rxn01510	0	1000
rxn01513	0,022515273	0,022515287
rxn01517	0	0
rxn01518	0,022515273	1000
rxn01519	0	0
rxn01521	0	999,9774847

rxn01522	0	0
rxn01537	0	999,9997976
rxn01539	-1000	-0,000202374
rxn01544	-999,9977294	0
rxn01548	0,02324128	1000
rxn01549	0	0
rxn01562	0	0
rxn01575	-0,91422683	0
rxn01601	0	0
rxn01602	0	0
rxn01603	0	0
rxn01607	0,000549042	0,000549043
rxn01615	0	0
rxn01620	0	0
rxn01629	-0,001618995	-0,001618994
rxn01636	-15,9707328	5,839235112
rxn01637	-5,839235112	-0,036659693
rxn01639	0	0
rxn01641	0	0
rxn01642	0	0
rxn01643	-0,500434697	-0,031146582
rxn01644	0,025026348	0,494314462
rxn01646	0	0
rxn01647	0	999,9977294
rxn01648	-1000	1000
rxn01649	0	999,9977294
rxn01650	0	0
rxn01653	0	0
rxn01654	0	0
rxn01667	0	0
rxn01669	0	999,9979318
rxn01670	0	0
rxn01675	0	0
rxn01679	0	0
rxn01684	0	0
rxn01685	0	0
rxn01686	0	0
rxn01704	0	0
rxn01706	0	0
rxn01734	0	0
rxn01735	0	0
rxn01737	0	0
rxn01739	0,000202374	1,98764094
rxn01740	-1,98764094	-0,000202374
rxn01741	0	0
rxn01757	0	0
rxn01775	0	0
rxn01790	0	0
rxn01799	-0,022515287	0,26817785
rxn01800	0	0,290693137

rxn01807	0	0
rxn01834	0	0
rxn01850	0	0
rxn01851	0	5,791812173
rxn01857	0	0
rxn01859	0	0,392788756
rxn01860	0	0
rxn01870	0	0
rxn01881	0	0
rxn01885	0	0
rxn01906	0	0
rxn01917	0,036659693	5,839235112
rxn01937	0	0
rxn01961	0	999,9977294
rxn01962	0	0
rxn01966	0	0
rxn01967	0	0
rxn01972	0,025026348	16,09354692
rxn01973	0	0
rxn01974	0,025026348	0,494314462
rxn01977	-1000	1000
rxn01982	0	0
rxn01985	0	0
rxn01986	0	0,637725312
rxn01997	0	0
rxn02000	0	0
rxn02003	0	0
rxn02008	0,025026348	0,025026363
rxn02011	0,025026348	0,025026363
rxn02023	0	0
rxn02056	0	999,9997976
rxn02061	0	0
rxn02106	0	0
rxn02122	0	0
rxn02123	0	0
rxn02128	0	0
rxn02132	0	0
rxn02138	0	0
rxn02139	0	0
rxn02154	0	999,9979318
rxn02155	0,002068211	1000
rxn02160	0	0
rxn02167	0	999,999451
rxn02171	0,000549042	5,792014547
rxn02175	0,000522725	1000
rxn02176	0	999,9994773
rxn02185	-1,987438566	0,91422683
rxn02186	0	1,987438566
rxn02187	0	0
rxn02195	0	0

rxn02202	0	0
rxn02203	0	0
rxn02209	0	0
rxn02212	0,000202374	1,98764094
rxn02213	0,000202374	1,98764094
rxn02219	0	0
rxn02264	0,000202374	0,000202374
rxn02283	0	0
rxn02284	-0,025026363	0
rxn02285	-0,025026363	0
rxn02286	0,025026348	0,025026363
rxn02297	0	0
rxn02302	-1000	-0,000202374
rxn02305	0,000202374	1000
rxn02314	0	1000
rxn02315	0	9,891425144
rxn02316	0	9,891425144
rxn02317	-1000	0
rxn02318	0	0
rxn02320	0	0
rxn02322	0,000549042	0,000549043
rxn02339	0	0
rxn02341	0,000522725	0,000522725
rxn02350	0	0
rxn02351	0	0
rxn02356	-1000	1000
rxn02358	-1000	1000
rxn02373	-1000	1000
rxn02375	0	0
rxn02380	-1000	1000
rxn02400	0	999,9977294
rxn02409	0	0
rxn02449	0	0
rxn02454	0	0
rxn02465	-5,839235112	-0,036659693
rxn02473	0	0
rxn02476	0,000202374	1,98764094
rxn02484	0	999,9997976
rxn02495	0	0
rxn02518	0	0
rxn02521	0	0
rxn02522	0	0
rxn02525	0	0
rxn02569	0	0
rxn02571	0	0
rxn02581	0	0
rxn02597	0	0
rxn02632	0	0
rxn02729	0	0
rxn02749	0	0

rxn02751	0	0
rxn02760	0	0
rxn02762	0	0
rxn02774	0	0
rxn02775	0	0
rxn02789	0	0
rxn02811	0	0
rxn02834	0	0
rxn02835	0	0
rxn02853	0	0
rxn02875	0	0
rxn02895	0,000202374	0,000202374
rxn02897	0	0
rxn02898	0	0
rxn02914	0	0
rxn02922	0	0
rxn02928	-1000	999,9749737
rxn02929	-1000	999,9749737
rxn02931	0	0
rxn02936	0	0
rxn02937	0,000202374	0,000202374
rxn02985	0	0
rxn02990	0	0
rxn03004	0	0,000202374
rxn03005	-0,000202374	0
rxn03008	0	0
rxn03030	0,025026348	16,09354692
rxn03039	0	0
rxn03047	0	0
rxn03062	0	0
rxn03068	0	0
rxn03075	0	999,9997976
rxn03084	0,000202374	0,000202374
rxn03086	-16,09354692	-0,025026348
rxn03087	0	0
rxn03102	0	0
rxn03106	0	0
rxn03108	0,000202374	1000
rxn03131	0	0
rxn03135	0	0
rxn03136	0	0
rxn03137	0	0
rxn03140	0	0
rxn03141	0	0
rxn03147	0	0
rxn03150	0	0
rxn03159	0	0
rxn03164	0,025026348	0,025026363
rxn03167	0	0
rxn03175	0	0

rxn03181	0	0
rxn03194	0	0,91422683
rxn03251	0	0
rxn03263	0	0
rxn03264	0	0
rxn03269	0	0
rxn03273	0	0
rxn03282	0	0
rxn03292	0	0
rxn03293	0	0
rxn03295	0	0
rxn03296	0	0
rxn03301	0	0
rxn03304	0	0
rxn03313	0	0
rxn03316	0	0
rxn03333	0	0
rxn03354	0	0
rxn03374	0	0
rxn03379	0	0
rxn03387	0	0
rxn03402	0	0
rxn03405	0	0
rxn03406	0	0
rxn03407	0	0
rxn03408	0,025026348	0,025026363
rxn03409	0	0
rxn03423	0	0
rxn03435	-0,91422683	0
rxn03436	0	0,91422683
rxn03437	0	0,91422683
rxn03445	0	0
rxn03446	0	0
rxn03462	0	0
rxn03468	0	0
rxn03481	0	0
rxn03482	0	0
rxn03483	0	0
rxn03491	0	0
rxn03492	0	0
rxn03512	0	0
rxn03513	0	0
rxn03514	0	0
rxn03535	0	0
rxn03536	0	0
rxn03537	0	0
rxn03538	0	0
rxn03540	0	0
rxn03546	0	0
rxn03548	0	1000

rxn03549	0	0
rxn03552	0	0
rxn03553	0	0
rxn03598	0	0
rxn03599	0	0
rxn03634	0	0
rxn03638	0,050052696	0,050052726
rxn03641	0,000549042	5,792014547
rxn03642	0,000549042	5,792014547
rxn03807	0	0
rxn03861	0	0
rxn03870	0	0
rxn03884	0	0
rxn03885	0	0
rxn03891	0	0
rxn03901	0,025026348	0,025026363
rxn03902	0	0
rxn03903	0	0
rxn03904	0,025026348	0,025026363
rxn03907	0	0
rxn03908	0	0
rxn03909	0	0
rxn03910	0	0
rxn03933	0	0
rxn03958	0	0
rxn03963	-1000	0
rxn03964	0	1000
rxn04045	0	0
rxn04046	0	0
rxn04047	0	0
rxn04048	0	0
rxn04050	0	0
rxn04051	0	0
rxn04052	0	0
rxn04113	0	0
rxn04142	0	0
rxn04234	0	0
rxn04290	0	0
rxn04308	0	0
rxn04384	0	0
rxn04385	0	0
rxn04413	0	0
rxn04443	0	0
rxn04482	0	0
rxn04676	0	1000
rxn04677	0	0
rxn04678	-1000	0
rxn04704	0	0
rxn04726	0	0
rxn04736	0	0

rxn04750	0	0
rxn04786	0,006120234	0,006120238
rxn04794	0	10,97559197
rxn04809	0	0
rxn04810	0	0
rxn04811	0	0
rxn04822	0	0
rxn04830	0	0
rxn04831	0	0
rxn04832	0	0
rxn04833	0	0
rxn04872	0	0
rxn04873	0	0
rxn04886	0	0
rxn04887	0	0
rxn04894	0	0
rxn04895	0	0
rxn04896	0	0
rxn04903	0	0
rxn04916	0	0
rxn04919	0	0
rxn04943	0	0
rxn04952	-999,9999719	0
rxn04953	-999,9999719	0
rxn04954	-1,367181966	0
rxn05024	0	0
rxn05029	0	0
rxn05030	4,99129E-05	4,9913E-05
rxn05039	0	0
rxn05050	0	0
rxn05054	0	0
rxn05115	0	0
rxn05117	0	0
rxn05119	0	0
rxn05122	0	0
rxn05234	0	0
rxn05236	0	0
rxn05247	0	0
rxn05248	0	0
rxn05249	0	0
rxn05250	0	0
rxn05251	0	0
rxn05252	0	0
rxn05269	0	0
rxn05289	0	0
rxn05322	0	0
rxn05323	0	0
rxn05324	0	0
rxn05325	0	0
rxn05326	0	0

rxn05327	0	0
rxn05328	0	0
rxn05329	0	0
rxn05330	0	0
rxn05331	0	0
rxn05332	0	0
rxn05333	0	0
rxn05334	0	0
rxn05335	0	0
rxn05336	0	0
rxn05337	0	0
rxn05338	0	0
rxn05339	0	0
rxn05340	0	0
rxn05341	0	0
rxn05342	0	0
rxn05343	0	0
rxn05344	0	0
rxn05345	0	0
rxn05346	0	0
rxn05347	0	0
rxn05348	0	0
rxn05350	0	0
rxn05457	-1000	0
rxn05465	0	0
rxn05733	0	0
rxn05736	0	1000
rxn05740	-1000	1000
rxn05759	-0,5	0
rxn05760	-1,575807765	1000
rxn05763	0	0
rxn05778	0	0
rxn05779	0	0
rxn05794	-1000	0
rxn05853	0	0
rxn05854	0	0
rxn05856	0	0
rxn05871	0	0
rxn05872	0	0
rxn05873	0	0
rxn05874	0	0
rxn05901	0	0
rxn05918	0	0
rxn05927	0	0
rxn05934	0	0
rxn05937	-10,24030777	1000
rxn05938	-16,12246375	0
rxn05939	0,000460107	1000
rxn05940	-10,91708871	0,91422683
rxn05958	0	0

rxn05962	0	0
rxn05979	0	0
rxn05990	0	0
rxn05994	0	0
rxn06005	0	0
rxn06023	0	0
rxn06043	0	0
rxn06044	0	0
rxn06045	0	0
rxn06078	0	0
rxn06080	0	0
rxn06081	0	0
rxn06090	0	0
rxn06096	0	0
rxn06108	-1000	-0,000549042
rxn06109	-5,488345029	-0,000549042
rxn06139	0	0
rxn06140	0	0
rxn06181	0	1000
rxn06182	0	1000
rxn06195	0	0
rxn06196	0	0
rxn06200	0	0
rxn06217	0	0
rxn06218	0	0
rxn06231	0	0
rxn06243	0	0
rxn06280	0	0
rxn06285	0	0
rxn06298	0	0
rxn06300	0	0
rxn06316	0	0
rxn06328	0	0
rxn06347	0	0
rxn06348	0	0
rxn06403	0	0
rxn06412	0	0
rxn06432	0	0
rxn06434	0	0
rxn06435	0	0
rxn06437	0	0
rxn06438	0	0
rxn06439	0	0
rxn06440	0	0
rxn06441	0	0
rxn06443	0	0
rxn06444	0	0
rxn06445	0	0
rxn06446	0	0
rxn06447	0	0

rxn06448	0	0
rxn06449	0	0
rxn06459	0	0
rxn06485	0	0
rxn06489	0	0
rxn06500	0	0
rxn06538	0	0
rxn06556	0	0
rxn06584	0	0
rxn06591	0,001618994	0,001618995
rxn06592	0	0
rxn06595	0	0
rxn06624	0	0
rxn06648	0	0
rxn06664	0	0
rxn06673	0	0
rxn06678	0	0
rxn06694	0	0
rxn06726	0	0
rxn06737	0	0
rxn06741	0	0
rxn06751	0	0
rxn06768	0	0
rxn06799	0	0
rxn06823	0	0
rxn06850	0	0
rxn06860	0	0
rxn06864	0	0
rxn06882	0	0
rxn06883	0	0
rxn06887	0	0
rxn06889	0	1000
rxn06890	0	0
rxn06936	0	0
rxn06937	0,001618994	0,001618995
rxn06947	0	0
rxn06979	0	0
rxn06983	0	0
rxn07056	0	0
rxn07099	0	0
rxn07181	0	0
rxn07241	0	0
rxn07267	0	0
rxn07292	0	0
rxn07437	0	0
rxn07441	0	16,06852057
rxn07456	0	0,234644057
rxn07465	0,006120234	0,006120238
rxn07466	-1000	1000
rxn07476	0	0

rxn07479	0	0
rxn07486	0	0
rxn07489	0	0
rxn07573	0	0
rxn07577	0	0
rxn07578	0	0
rxn07579	0	0
rxn07586	0	0
rxn07587	0	0
rxn07589	0	0
rxn07645	0	0
rxn07679	0	0
rxn07683	0	0
rxn07687	0	0
rxn07846	0	0
rxn07849	0	0
rxn07987	0	0
rxn07989	0	0
rxn07991	0	0
rxn07992	0	0
rxn07993	0	0
rxn07994	0	0
rxn08025	0	0
rxn08035	0	0
rxn08038	0	0
rxn08043	0	0,91422683
rxn08067	-1000	1000
rxn08083	0	0
rxn08084	0	0
rxn08085	0	0
rxn08086	0	0
rxn08087	0	0
rxn08088	0	0
rxn08089	0	0
rxn08126	0	0
rxn08127	0	0
rxn08128	0	0
rxn08129	0	0
rxn08131	0,000202374	0,000202374
rxn08171	0	0
rxn08180	0	0
rxn08194	-1000	1000
rxn08294	0	1000
rxn08295	0	1000
rxn08296	0	1000
rxn08297	0	1000
rxn08298	0	1000
rxn08299	0	1000
rxn08300	0	1000
rxn08306	0	0

rxn08307	0	0
rxn08308	0	0
rxn08309	0	0
rxn08310	0	0
rxn08311	0	0
rxn08312	0	0
rxn08352	0	0
rxn08386	0	0
rxn08390	0	0
rxn08392	0	0
rxn08394	0	0
rxn08396	0	0
rxn08398	0	0
rxn08413	0	0
rxn08433	0	0
rxn08438	0	0
rxn08448	0	0
rxn08449	0	0
rxn08451	0	0
rxn08453	0	0
rxn08454	0	1000
rxn08455	0	0
rxn08456	0	0
rxn08457	0	0
rxn08519	0,045756553	0,045756581
rxn08546	0	0
rxn08547	0	1000
rxn08548	0	0
rxn08549	0	0
rxn08550	0	0
rxn08551	0	0
rxn08552	0	0
rxn08571	0	1000
rxn08582	0	0,5
rxn08605	0	0
rxn08615	-1000	1000
rxn08647	0	0
rxn08668	0	0
rxn08669	0	0
rxn08764	0	0,91422683
rxn08796	0	0
rxn08797	0	1000
rxn08798	0	0
rxn08799	0	1000
rxn08800	0	0
rxn08801	0	1000
rxn08802	0	0
rxn08803	0	0
rxn08804	0	0
rxn08805	0	0

rxn08806	0	0
rxn08807	0	0
rxn08808	0	0
rxn08809	0	0
rxn08810	0	0
rxn08811	0	0
rxn08812	0	0
rxn08813	0	0
rxn08814	0	0
rxn08815	0	0
rxn08816	0	0
rxn08817	0	0
rxn08818	0	0
rxn08819	0	0
rxn08820	0	0
rxn08821	0	0
rxn08822	0	0
rxn08823	0	0
rxn08838	0	0
rxn08839	0	0
rxn08840	0	0
rxn08841	0	0
rxn08842	0	0
rxn08843	0	0
rxn08844	0	0
rxn08845	0	0
rxn08846	0	0
rxn08847	0	0
rxn08848	0	0
rxn08849	0	0
rxn08850	0	0
rxn08851	0	0
rxn08857	0	0
rxn08889	0,000610753	0,000610753
rxn08890	0,004944104	0,004944107
rxn08891	0,000610753	0,000610753
rxn08892	-999,988403	1000
rxn08893	-999,9938961	999,9945069
rxn08894	-999,988403	1000
rxn08897	-0,005493149	-0,005493146
rxn08926	0,000549042	0,000549043
rxn08927	-999,9987794	999,9896236
rxn08928	-999,988403	1000
rxn08929	0,001220598	0,001220599
rxn08930	0	0
rxn08958	0,000610753	0,000610753
rxn09010	0	0
rxn09016	0	9,914666424
rxn09062	0	1000
rxn09063	0	1000

rxn09064	0	1000
rxn09065	0	1000
rxn09066	0	1000
rxn09067	0	1000
rxn09068	0	1000
rxn09101	0	0
rxn09102	0	0
rxn09103	0	0
rxn09104	0	0
rxn09105	0	0
rxn09106	0	0
rxn09107	0	0
rxn09108	0	0
rxn09109	0	0
rxn09110	0	0
rxn09111	0	0
rxn09112	0	0
rxn09113	0	0
rxn09114	0	0
rxn09176	-1000	1000
rxn09177	0	0,000522725
rxn09197	0	0
rxn09198	0	0
rxn09199	0	0
rxn09200	0	0
rxn09201	0	0
rxn09202	0	0
rxn09203	0	0
rxn09205	0	0
rxn09206	0	0
rxn09207	0	0
rxn09208	0	0
rxn09209	0	0
rxn09210	0	0
rxn09211	0	0
rxn09235	0,022515273	0,022515287
rxn09237	0,02324128	0,023241294
rxn09340	0	0
rxn09341	0	9,891425144
rxn09348	0	9,891425144
rxn09355	0	0
rxn09395	0	0
rxn09399	0	0
rxn09412	-1000	1000
rxn09445	0	0
rxn09446	0	0
rxn09447	0	0
rxn09473	0	0
rxn09486	0	0
rxn09502	0	1000

rxn09519	0	0
rxn09531	0	0
rxn09557	0,000202374	0,000202374
rxn09631	0,000202374	0,000202374
rxn09888	0	0
rxn09889	0	0
rxn09952	0	0
rxn09978	0	0
rxn09979	0	0
rxn09988	0	0
rxn09992	0	0
rxn09995	0	0
rxn10003	0	0,000522725
rxn10019	0	0
rxn10020	0	0
rxn10021	0	0
rxn10052	-1000	1000
rxn10054	0	9,891947868
rxn10056	0	0,000405656
rxn10058	0	0,000405656
rxn10060	0	0,000405656
rxn10091	-1000	1000
rxn10107	0	0
rxn10110	0	0
rxn10111	0	0
rxn10192	0	0
rxn10202	0	1000
rxn10203	0	1000
rxn10204	0	1000
rxn10205	0	0
rxn10206	0	0
rxn10207	0	0
rxn10208	0	0
rxn10209	0	0
rxn10210	0	0
rxn10211	0	0
rxn10212	0	0
rxn10213	0	0
rxn10214	0	0
rxn10215	0	0
rxn10216	0	0
rxn10217	0	0
rxn10218	0	0
rxn10219	0	0
rxn10220	0	0
rxn10221	0	0
rxn10222	0	0
rxn10223	0	0
rxn10224	0	0
rxn10225	0	0

rxn10226	0	0
rxn10227	0	0
rxn10228	0	0
rxn10229	0	0
rxn10230	0	0
rxn10231	0	0
rxn10232	0	0
rxn10233	0	0
rxn10234	0	0
rxn10235	0	0
rxn10236	0	0
rxn10237	0	0
rxn10238	0	1000
rxn10239	0	1000
rxn10240	0	1000
rxn10241	0	1000
rxn10242	0	1000
rxn10243	0	1000
rxn10253	0	1000
rxn10254	0	1000
rxn10255	0	1000
rxn10256	0	1000
rxn10257	0	1000
rxn10258	0	1000
rxn10259	0	0
rxn10260	0	0
rxn10261	0	0
rxn10262	0	0
rxn10263	0	0
rxn10264	0	0
rxn10265	0	0
rxn10266	0	0
rxn10267	0	0
rxn10268	0	0
rxn10269	0	0
rxn10270	0	0
rxn10289	0	0
rxn10290	0	0
rxn10291	0	0
rxn10292	0	0
rxn10293	0	0
rxn10294	0	0
rxn10295	0	0
rxn10296	0	0
rxn10297	0	0
rxn10363	0	0
rxn10404	0	0
rxn10405	0	0
rxn10406	0	0
rxn10407	0	0

rxn10408	0	0
rxn10409	0	0
rxn10410	0	0
rxn10785	4,99129E-05	4,9913E-05
rxn10790	0,000202374	1000
rxn10798	-1000	-0,000202374
rxn10951	0,022515273	0,022515287
rxn11007	0,022515273	0,022515287
rxn11513	0	0
rxn11548	0	0
rxn11550	0	0
rxn11567	0	0
rxn11571	0	0
rxn11599	0	0
rxn11641	0	0
rxn11732	0	0
rxn11749	0	0
rxn11759	0	0
rxn11760	0	0
rxn11761	0	0
rxn11765	0	0
rxn11766	0	0
rxn11768	0	0
rxn11772	0	0
rxn11773	0	0
rxn11788	0	0
rxn11808	0	0
rxn11890	0	0
rxn11965	0	0
rxn12049	0	0
rxn12154	0	0
rxn12218	-1000	-0,000202374
rxn12221	0,000202374	1000
rxn12510	0,000522725	0,000522725
rxn12649	-999,9991905	0
rxn12778	0	0
rxn12822	-1000	0
rxn13420	0,000549042	1000
rxn13421	0,000549042	1000
rxn13705	0	0
rxn13741	0	0
rxn13906	-0,006120238	-0,006120234
rxn13936	0,012207798	0,012207805
rxn13974	-16,12246375	0
rxn14043	0	0
rxn14048	-999,9995399	0
rxn14050	0	0
rxn14054	-1000	0
rxn14063	0	0
rxn14070	0	0

rxn14089	-1000	0
rxn14120	-1000	-0,000809497
rxn14132	0	0
rxn14147	0	0
rxn14191	0	0
rxn14198	0	0
rxn14250	0	0
rxn14270	0	0
rxn14279	0	0
rxn14297	0	0
rxn14346	0	0
rxn90002	-9,78883651	1000
rxn90003	0	0
rxn90004	0	0
rxn90005	-0,022920943	-0,022515273
rxn08173	0	499,9997255
Biomass_Bacteria	0,907508	0,907508544
t_Cl	0,004094676	0,004094679
t_Sulfate	0,00341223	0,003412232
t_Cu2+	0,002729784	0,002729786
t_Mg	0,006823553	0,006823557
t_Ca2+	0,004094676	0,004094679
t_NH3	0	0
t_H2O	-21,22472166	6,055418698
t_Biomass	-0,907508544	-0,907508
t_Butyrate	-0,94214122	0
t_D-Lactate	-10,71234705	0
t_Ethanol	-0,637725312	0
t_Formate	-10,97599672	0
t_H2	0	0,5
t_Nitrite	0	0
t_Phosphate	1,205948358	1,598737837
t_Propionate	-0,91422683	0
t_O2	0	0
t_D-Glucose	0	0,5
t_CO2	-10,97559197	0
t_Acetate	-16,29995847	-0,02557539
t_Succinate	-5,487795987	0
t_(S,S)-2,3-Butanediol	0	0
t_H2S	-0,418718138	0
Ex_Cl	-0,004094679	-0,004094676
Ex_Sulfate	-0,003412232	-0,00341223
Ex_Cu2+	-0,002729786	-0,002729784
Ex_Mg	-0,006823557	-0,006823553
Ex_Ca2+	-0,004094679	-0,004094676
Ex_NH3	0	0
Ex_H2O	-6,055418698	21,22472166
Ex_Biomass	0,907508	0,907508544
Ex_Butyrate	0	0,94214122
Ex_D-Lactate	0	10,71234705

Ex_Ethanol	0	0,637725312
Ex_Formate	0	10,97599672
Ex_H2	-0,5	0
Ex_Nitrite	0	0
Ex_Phosphate	-1,598737837	-1,205948358
Ex_Propionate	0	0,91422683
Ex_O2	0	0
Ex_D-Glucose	-0,5	0
Ex_CO2	0	10,97559197
Ex_Acetate	0,02557539	16,29995847
Ex_Succinate	0	5,487795987
Ex_(S,S)-2,3-Butanediol	0	0
Ex_H2S	0	0,418718138
t_Fe2	0,006343481	0,006343485
t_fe3	0,006141107	0,00614111
t_Acetaldehyde	-0,637725312	0
t_Adenosine	0	0,392788756
t_AMP	0	0,392788756
t_Amylotriose	0	0
t_BIOT	0	0
t_Choline	0	0
t_Cytidine	0	0
t_Cytosine	0	0
t_DAlanine	0	0
t_Deoxyadenosine	0	0,392788756
t_Deoxycytidine	0	0,290693137
t_Deoxyguanosine	0	0
t_Deoxyinosine	0	0
t_Deoxyuridine	0	0
t_DRibose	0	0,5
t_Glycerol	0	0
t_GSH	0	0
t_Guanine	0	0
t_H2S2O3	0	0
t_Heme	0,000202374	0,000202374
t_Homocysteine	0	0
t_HYXN	0	0,392788756
t_Inosine	0	0,392788756
t_LACT	0	0,5
t_LAlanine	0,4999997	0,5
t_LArabinose	0	0
t_LArginine	0,026173742	0,260817956
t_LAsparagine	-0,256749741	0,212538501
t_LAspartate	0,030711886	0,5
t_LCysteine	0,081281862	0,5
t_LGlutamate	-0,44214122	0,5
t_LGlutamine	-0,44214122	0,5
t_LHistidine	0,083581487	0,083581537
t_LInositol	0	0
t_LIsoleucine	-0,658037168	0,256189662

t_LLeucine	0,397307002	0,397307241
t_LLysine	-0,166724947	0,302563349
t_LMethionine	-0,282620071	0,136098149
t_LPhenylalanine	-1,824087126	0,163351538
t_LThreonine	-0,414024456	0,5
t_LTryptophan	0,050121667	0,050121697
t_LTyrosine	-1,865832494	0,121606145
t_LValine	-1,613636021	0,373802769
t_Maltose	0	0,5
t_Niacin	0,002068211	0,002068212
t_Ornithine	0	0
t_PPi	0	0
t_Pyridoxol	0	0
t_XAN	0	0
t_5Deoxyadenosine	0	0
t_Acetoacetate	-5,356173523	0
t_Calomide	0	0
t_Cbl	0	0
t_Citrate	0	0
t_CysGly	0	0
t_Glycine	-1,487438566	0,5
t_Glycolaldehyde	0	0
t_LProline	0,194932718	0,194932835
t_Maltohexaose	0	0
t_Methanol	0	0
t_NAcetylDglucosamine	0	0
t_PM	0	0
t_Putrescine	0	0
t_Pyridoxal	0,000202374	0,000202374
t_Riboflavin	0,000404749	0,000404749
t_Salicin	0	0
t_Sorbitol	0	0
t_Spermidine	0	0
t_Sucrose	0	0,5
t_Thiamin	0	0
t_Thymidine	0	0
t_TRHL	0	0
t_Uracil	0	0,290693137
t_Uridine	0	0,290693137
t_Ursin	0	0
t_Mn2+	0,002729784	0,002729786
t_Formaldehyde	0	0
t_Fumarate	-5,487795987	0
t_Oxidized glutathione	0	0
t_Adenine	0	0
t_Nicotinamide	0	0
t_Co2+	0,002729784	0,002729786
t_D-Glutamate	0	0
t_Chorismate	0	0
t_Folate	0,000809497	0,000809498

t_N-Acetyl-D-mannosamine	0	0
t_Siroheme	0	0
t_Menaquinone 7	0	0
t_2-Demethylmenaquinone 8	0	0
t_Menaquinone 8	0	0
t_Ubiquinone-8	0	0
t_2-Oxobutyrate	0	0
t_3MOP	0	0
t_Neu5Ac	0	0
t_Glycerol-3-phosphate	0	0
t_H+	-1000	0,5
t_Nicotinamide ribonucleotide	0	0
t_PAN	0,000522725	0,000522725
t_Pyridoxal phosphate	0	0
t_Zn2+	0,002729784	0,002729786
t_1,2-Diacyl-sn-glycerol dioctadecanoyl	0	0
t_meso-2,6-Diaminopimelate	0	0
t_L-Serine	-1,487438566	0,5
t_D-Fructose	0	0,5
t_beta D-Galactose	0	0,5
t_L-Fucose	0	0
Ex_Fe2	-0,006343485	-0,006343481
Ex_fe3	-0,00614111	-0,006141107
Ex_Acetaldehyde	0	0,637725312
Ex_Adenosine	-0,392788756	0
Ex_AMP	-0,392788756	0
Ex_Amylotriose	0	0
Ex_BIOT	0	0
Ex_Choline	0	0
Ex_Cytidine	0	0
Ex_Cytosine	0	0
Ex_DAlanine	0	0
Ex_Deoxyadenosine	-0,392788756	0
Ex_Deoxycytidine	-0,290693137	0
Ex_Deoxyguanosine	0	0
Ex_Deoxyinosine	0	0
Ex_Deoxyuridine	0	0
Ex_DRibose	-0,5	0
Ex_Glycerol	0	0
Ex_GSH	0	0
Ex_Guanine	0	0
Ex_Heme	-0,000202374	-0,000202374
Ex_Homocysteine	0	0
Ex_HYXN	-0,392788756	0
Ex_Inosine	-0,392788756	0
Ex_LACT	-0,5	0
Ex_LAlanine	-0,5	-0,4999997
Ex_LArabinose	0	0
Ex_LArginine	-0,260817956	-0,026173742
Ex_LAsparagine	-0,212538501	0,256749741

Ex_LAspartate	-0,5	-0,030711886
Ex_LCysteine	-0,5	-0,081281862
Ex_LGlutamate	-0,5	0,44214122
Ex_LGlutamine	-0,5	0,44214122
Ex_LHistidine	-0,083581537	-0,083581487
Ex_LInositol	0	0
Ex_LIsoleucine	-0,256189662	0,658037168
Ex_LLeucine	-0,397307241	-0,397307002
Ex_LLysine	-0,302563349	0,166724947
Ex_LMethionine	-0,136098149	0,282620071
Ex_LPhenylalanine	-0,163351538	1,824087126
Ex_LThreonine	-0,5	0,414024456
Ex_LTryptophan	-0,050121697	-0,050121667
Ex_LTyrosine	-0,121606145	1,865832494
Ex_LValine	-0,373802769	1,613636021
Ex_Maltose	-0,5	0
Ex_Niacin	-0,002068212	-0,002068211
Ex_Ornithine	0	0
Ex_PPi	0	0
Ex_XAN	0	0
Ex_5Deoxyadenosine	0	0
Ex_Acetoacetate	0	5,356173523
Ex_Calomide	0	0
Ex_Cbl	0	0
Ex_Citrate	0	0
Ex_CysGly	0	0
Ex_Glycine	-0,5	1,487438566
Ex_Glycolaldehyde	0	0
Ex_LProline	-0,194932835	-0,194932718
Ex_Maltohexaose	0	0
Ex_Methanol	0	0
Ex_NAcetylDglucosamine	0	0
Ex_PM	0	0
Ex_Putrescine	0	0
Ex_Pyridoxal	-0,000202374	-0,000202374
Ex_Riboflavin	-0,000404749	-0,000404749
Ex_Salicin	0	0
Ex_Sorbitol	0	0
Ex_Spermidine	0	0
Ex_Sucrose	-0,5	0
Ex_Thiamin	0	0
Ex_Thymidine	0	0
Ex_TRHL	0	0
Ex_Uracil	-0,290693137	0
Ex_Uridine	-0,290693137	0
Ex_Ursin	0	0
Ex_Mn2+	-0,002729786	-0,002729784
Ex_Formaldehyde	0	0
Ex_Fumarate	0	5,487795987
Ex_Oxidized glutathione	0	0

Ex_Adenine	0	0
Ex_Nicotinamide	0	0
Ex_Co2+	-0,002729786	-0,002729784
Ex_D-Glutamate	0	0
Ex_Folate	-0,000809498	-0,000809497
Ex_N-Acetyl-D-mannosamine	0	0
Ex_Siroheme	0	0
Ex_Menaquinone 7	0	0
Ex_2-Demethylmenaquinone 8	0	0
Ex_Menaquinone 8	0	0
Ex_Ubiquinone-8	0	0
Ex_Neu5Ac	0	0
Ex_H+	-0,5	1000
Ex_Nicotinamide ribonucleotide	0	0
Ex_PAN	-0,000522725	-0,000522725
Ex_Zn2+	-0,002729786	-0,002729784
Ex_1,2-Diacyl-sn-glycerol dioctadecanoyl	0	0
Ex_L-Serine	-0,5	1,487438566
Ex_D-Fructose	-0,5	0
Ex_beta D-Galactose	-0,5	0
Ex_L-Fucose	0	0
t_Arabinan	0	0
t_Starch	0	0,005
t_octanoate	0	0
t_Melibiose	0	0
t_Amylose	0	0
Ex_Arabinan	0	0
Ex_Starch	-0,005	0
Ex_Melibiose	0	0
Ex_Amylose	0	0
t_Raffinose_Melitose	0	0
t_Isovaleric_acid	0	0
t_H2O2	0	0
Ex_Raffinose_Melitose	0	0
Ex_Isovaleric_acid	0	0
Ex_H2O2	0	0
rxn01207_1	0	0
rxn08972	0	0
rxn08973	0	0
rxn06111	0	999,999451
rxn13726	0	0
rxn13727	0	0
rxn13729	0	0
rxn08974	0	0
rxn10122	0	0
rxn10123	0	0
rxn10124	0	0
rxn12665	0	0
rxn06097	0	0,005
t_Sulfite	0	0

Ex_Sulfite	0	0

rxn ID	minFlux	max Flux
rxn00001	0	1000
rxn00003	-6,595648703	0
rxn00011	-6,595648703	0
rxn00016	0	0
rxn00022	0	1000
rxn00029	0,00101873	0,00101873
rxn00048	0	0,000509365
rxn00060	0,000254683	0,000254683
rxn00062	0	1000
rxn00065	0	0
rxn00066	0	1000
rxn00067	0	0
rxn00070	-1000	0
rxn00076	0	1000
rxn00077	0	0,000510507
rxn00085	-959,4959563	0
rxn00086	-1000	0
rxn00097	-1000	445,2638412
rxn00100	0,000657835	0,000657835
rxn00101	0,046135221	0,171767932
rxn00103	0	1000
rxn00105	-999,9973972	1000
rxn00106	-1000	0
rxn00109	0	0
rxn00114	-0,425820012	0
rxn00119	0,368989262	12,39470307
rxn00122	0,000254683	0,000254683
rxn00124	0,000254683	0,000254683
rxn00126	0,000764048	0,000764048
rxn00127	0	0
rxn00132	0	1000
rxn00133	0	0
rxn00134	0	1000
rxn00137	0	0
rxn00138	0,002602787	1000
rxn00139	-999,7290863	0
rxn00142	0	0
rxn00143	0,000509365	0,000509365
rxn00148	-12,02505597	0
rxn00149	0	12,02505597
rxn00154	0	13,1899155
rxn00157	-13,1899155	0
rxn00159	-1000	1000
rxn00161	-1000	1000
rxn00162	0	0,425820012
rxn00165	0	0,260507082
rxn00171	0	1,308245774
rxn00173	0	13,82351645
rxn00184	-1000	0

rxn00187	0	1000
rxn00189	0	1000
rxn00193	0,031494975	0,031494975
rxn00196	0	0
rxn00205	0	1000
rxn00206	0	0
rxn00211	0	0
rxn00213	0	12,02505597
rxn00214	-1,5	0
rxn00216	0	1000
rxn00225	-13,82351645	0
rxn00231	0	0
rxn00239	0,238807673	12,26386364
rxn00247	0	0,425820012
rxn00250	-0,425855417	-3,54043E-05
rxn00256	0	0
rxn00260	-999,9997099	0,425855417
rxn00262	0	999,9997453
rxn00272	-1000	1000
rxn00275	-2,34579262	0,288627234
rxn00279	0	0,425820012
rxn00283	0,027731841	0,027731841
rxn00293	0,062989949	12,08804592
rxn00297	0	0
rxn00299	0	0,382044316
rxn00300	0	0,000509365
rxn00301	0	12,02505597
rxn00302	0	0,382044316
rxn00303	0	0
rxn00304	-12,2645546	-0,239498628
rxn00307	0	0
rxn00322	0	0
rxn00333	0,000254683	1000
rxn00337	0,039197122	0,039197122
rxn00338	0	0,002602787
rxn00340	0	1000
rxn00342	0	1000
rxn00346	0	0
rxn00350	-0,000254683	-0,000254683
rxn00358	0	0
rxn00360	0	1000
rxn00361	0	1000
rxn00363	0	1000
rxn00364	-12,0257138	12,0257138
rxn00365	0	1000
rxn00368	0	12,02505597
rxn00369	0	12,02505597
rxn00371	0	1000
rxn00379	0	0
rxn00383	0	0

rxn00391	0	999,9997453
rxn00392	0,000254683	1000
rxn00395	0	0
rxn00405	0,046135221	0,171767932
rxn00411	-12,0257138	0
rxn00412	0,17748972	12,20320352
rxn00414	0	0,425820012
rxn00416	0	719,2621046
rxn00420	0	0
rxn00422	-1000	1000
rxn00423	0	0,260507082
rxn00424	-1000	1000
rxn00426	0	0
rxn00433	0	0
rxn00436	0	719,2471183
rxn00437	0	0
rxn00440	0,000254683	719,247373
rxn00453	0	999,9997453
rxn00456	0	999,9997453
rxn00459	0,275159883	12,30021585
rxn00460	-12,42619804	-0,400484237
rxn00461	0,031494975	0,031494975
rxn00470	0	0
rxn00474	0	0
rxn00493	0	0
rxn00499	-13,1899155	0
rxn00506	0	1,308245774
rxn00512	-999,9997453	0
rxn00514	0	0
rxn00517	-12,02505597	0
rxn00527	0	0
rxn00533	3,54043E-05	3,54043E-05
rxn00543	-1,308245774	0
rxn00545	0	1000
rxn00547	0	1
rxn00549	0	1000
rxn00552	-0,06298995	999,9370101
rxn00554	0	12,02505597
rxn00555	0	1000
rxn00556	0	12,02505597
rxn00557	0	1000
rxn00558	-1000	1000
rxn00566	0	1000
rxn00575	0	1000
rxn00577	-1000	0,5
rxn00608	0	0
rxn00611	-0,226690271	0
rxn00615	0	0,226690271
rxn00616	0	0,226690271
rxn00622	0	0

rxn00623	0	0
rxn00624	0	0
rxn00647	0	0
rxn00649	0	0,260507082
rxn00650	-0,000254683	-0,000254683
rxn00653	0	0
rxn00654	0	0
rxn00670	0	0,218733442
rxn00684	-0,382044316	0
rxn00685	0	1000
rxn00686	-0,382044316	0
rxn00687	0	1000
rxn00689	0	0
rxn00690	0	2,418339445
rxn00693	0	0,218733442
rxn00695	-1000	998,5319107
rxn00701	0	13,52505597
rxn00704	-1000	2
rxn00707	0	1000
rxn00708	0	1000
rxn00709	0	1000
rxn00710	0	0
rxn00711	-999,7290863	0
rxn00712	0	12,02505597
rxn00713	0	12,02505597
rxn00714	0	0
rxn00715	0	1000
rxn00717	0	12,0257138
rxn00726	0	0
rxn00727	0	0
rxn00729	0	0
rxn00735	0	0
rxn00737	0	0,218478759
rxn00740	0	1000
rxn00741	0	0
rxn00742	-1000	0,000254683
rxn00743	0	0,226690271
rxn00747	-5,836572636	6,188483333
rxn00748	0	12,02505597
rxn00758	0	0
rxn00762	-0,226690271	0
rxn00763	0	0
rxn00765	0	0
rxn00770	0,270913658	1000
rxn00772	0	1000
rxn00775	0	0
rxn00777	-0,918988884	0,370156845
rxn00778	-1000	1000
rxn00781	0,275159883	12,30021585
rxn00784	0	1,308245774

rxn00785	-0,184653952	0,651280648
rxn00786	-1000	5,836572636
rxn00787	0	0
rxn00790	-0,000254683	-0,000254683
rxn00792	0	0
rxn00796	0	0
rxn00800	0	0,425820012
rxn00808	0	1,5
rxn00816	0	0,5
rxn00817	0	0,5
rxn00818	0	0
rxn00819	0	0
rxn00827	0	0
rxn00829	0,000690955	0,000690955
rxn00830	6,28141E-05	6,28141E-05
rxn00831	0	999,7290863
rxn00832	0	0
rxn00834	-999,7290863	999,9971425
rxn00836	-999,9971425	0
rxn00838	0	0,425820012
rxn00851	0	1000
rxn00855	0	0
rxn00856	0,008138419	0,13377113
rxn00858	0,046135221	0,171767932
rxn00869	0	0
rxn00871	0	6,594957748
rxn00877	0,000690955	6,595648703
rxn00879	0	0
rxn00881	0	0
rxn00882	0	0
rxn00883	0	0
rxn00889	0	0
rxn00890	0	0
rxn00898	0,000690955	6,595648703
rxn00907	-2,41783008	0,000509365
rxn00908	-2,388985859	0,201658299
rxn00909	-0,007702147	0,218988124
rxn00910	-0,226690271	0
rxn00913	0,029248515	999,7583349
rxn00915	-999,7290863	0
rxn00916	-999,7319438	609,524593
rxn00917	0	1000
rxn00918	0	0
rxn00925	0	0
rxn00926	0	1,076429882
rxn00927	-1000	1000
rxn00929	-1000	1000
rxn00931	-1000	1000
rxn00938	0	609,2565368
rxn00942	0	1000

rxn00950	-1000	0,218224076
rxn00952	0	999,9997453
rxn00955	0,000509365	0,000509365
rxn00965	0	0
rxn00977	0	0
rxn00979	0,000254683	0,382298998
rxn00980	0	0
rxn00985	-0,218733442	0
rxn00989	0,000690955	0,126323666
rxn00991	-0,000690955	-0,000690955
rxn01016	0	0
rxn01018	0	0
rxn01019	0	0
rxn01021	0	0
rxn01025	0	0
rxn01053	0	0
rxn01073	0	0
rxn01080	0	0
rxn01089	0	0
rxn01100	-24,29602331	0
rxn01101	0	0
rxn01106	-12,30021585	-0,275159883
rxn01108	-1000	1000
rxn01109	-1000	1000
rxn01114	0	0
rxn01116	-0,920007614	0,369817268
rxn01119	0	0
rxn01123	0	0
rxn01132	-1000	0,505
rxn01133	0	0
rxn01137	0	1,076429882
rxn01138	-1000	1000
rxn01139	0	0
rxn01146	0	0
rxn01153	0	0
rxn01169	0	1000
rxn01171	-1000	1000
rxn01200	0	1000
rxn01201	-0,126323666	-0,000690955
rxn01202	0	0
rxn01203	0	0
rxn01204	0,000690955	0,126323666
rxn01210	0	0
rxn01211	-2,418084763	0,000509365
rxn01213	6,28141E-05	6,28141E-05
rxn01225	0	609,2565368
rxn01226	-999,9707515	1000
rxn01228	0	0
rxn01236	-6,594957748	0
rxn01237	0	0

rxn01241	0	0
rxn01255	0,000254683	0,382298998
rxn01256	0	0
rxn01257	0	0,382044316
rxn01259	0	0
rxn01261	0	0
rxn01265	-999,7316891	0
rxn01270	0	0
rxn01274	0	0
rxn01276	0	0
rxn01278	0	0
rxn01286	0	0
rxn01297	-999,7290863	999,9971425
rxn01299	-1000	1000
rxn01305	0	0
rxn01313	0	0
rxn01314	0	0
rxn01321	0	0
rxn01332	0,000254683	0,382298998
rxn01333	-1000	0,312256739
rxn01334	0	1000
rxn01343	0	1000
rxn01346	0	12,02505597
rxn01347	0	12,02505597
rxn01348	0	1000
rxn01351	0	24,29602331
rxn01354	-12,02505597	0
rxn01358	-1000	1000
rxn01361	0	0
rxn01362	0	0
rxn01366	-12,119322	999,7574212
rxn01367	0	0
rxn01368	0	12,0257138
rxn01370	0	24,29602331
rxn01396	0	0
rxn01406	0	0
rxn01416	0	0
rxn01423	0	0
rxn01426	0	0
rxn01437	0	0
rxn01444	0,029248515	1000
rxn01445	0	999,9707515
rxn01446	-0,029248515	-0,029248515
rxn01452	-999,999309	0
rxn01459	0	0,126068983
rxn01465	0	0
rxn01466	6,28141E-05	6,28141E-05
rxn01476	0	0
rxn01484	0	0
rxn01485	-0,06298995	-0,062989949

rxn01486	0	0
rxn01487	0	0
rxn01492	0	0
rxn01500	-0,000690955	-0,000690955
rxn01503	0	0
rxn01509	0,029248515	24,32527182
rxn01510	0	24,29602331
rxn01513	0,028334856	0,028334856
rxn01517	0	0
rxn01518	0,028334856	1000
rxn01519	0	0
rxn01521	0	999,9716651
rxn01522	0	0
rxn01530	0	0
rxn01539	-719,247373	-0,000254683
rxn01544	-999,9971425	0
rxn01545	-1000	1000
rxn01548	-999,9707515	1000
rxn01549	0	0
rxn01601	0	0,382044316
rxn01602	0	0,382044316
rxn01603	0	0,382044316
rxn01610	0	0
rxn01615	0	0
rxn01626	0	0
rxn01629	-0,00203746	-0,00203746
rxn01641	0	0
rxn01642	0	0
rxn01643	-0,039197122	-0,039197122
rxn01644	0,031494975	0,031494975
rxn01645	-0,007702147	-0,007702147
rxn01646	-1000	609,2565368
rxn01647	0	999,7290863
rxn01648	0	12,0257138
rxn01649	-1000	1000
rxn01650	0	0
rxn01653	0	0
rxn01654	0	0
rxn01667	0	0
rxn01669	0	999,9973972
rxn01670	0	609,2565368
rxn01675	0	0
rxn01679	0	0
rxn01682	0	0
rxn01683	-1000	1000
rxn01684	-1000	1000
rxn01704	0	0
rxn01706	0	0
rxn01715	0	0
rxn01735	0	0

rxn01737	0	0
rxn01739	0,000254683	0,382298998
rxn01741	-0,382298998	-0,000254683
rxn01757	0	0
rxn01763	0	0
rxn01799	-0,028334856	0,33749429
rxn01800	0	0,365829146
rxn01807	0	0
rxn01816	0	1000
rxn01832	0	0
rxn01833	0	0
rxn01834	0	0
rxn01843	0	0
rxn01851	0	0,126068983
rxn01858	0	1,076429882
rxn01859	-1,076429882	0,5
rxn01870	0	0
rxn01892	0	0
rxn01903	0	0
rxn01906	0	0
rxn01937	0	0
rxn01953	0	0
rxn01961	0	999,7290863
rxn01962	0	0
rxn01964	0	0
rxn01967	-1000	0,505
rxn01972	0,031494975	13,85501143
rxn01973	0	0
rxn01974	0,031494975	0,031494975
rxn01977	-1000	1000
rxn01982	0	0
rxn01985	0	1,076429882
rxn01986	-0,057583371	0,808245774
rxn01987	-0,5	0
rxn01990	0	0
rxn01997	0	0
rxn02000	0	0
rxn02003	0	0
rxn02008	0,031494975	0,031494975
rxn02009	0	0
rxn02010	0	0
rxn02011	0,031494975	0,031494975
rxn02015	0	0
rxn02020	0	0
rxn02046	0	0
rxn02056	0	999,9997453
rxn02061	0	0
rxn02085	0	0
rxn02090	0	0
rxn02093	0	0

rxn02102	-1000	0
rxn02103	0	1000
rxn02106	0	0
rxn02122	0	0
rxn02128	0	0
rxn02138	0	0
rxn02144	0	0
rxn02154	0	999,9973972
rxn02155	0,002602787	1000
rxn02160	0	0
rxn02167	0	999,999309
rxn02170	0	0
rxn02171	0,000690955	0,126323666
rxn02175	0,000657835	0,000657835
rxn02185	-6,595648703	0
rxn02186	0,000690955	6,595648703
rxn02190	0	0
rxn02195	0	0
rxn02199	0	0
rxn02200	0	0,382044316
rxn02201	0	0,382044316
rxn02202	0	0
rxn02203	0	0
rxn02209	0	0
rxn02212	0,000254683	0,382298998
rxn02213	0	0
rxn02222	0	0
rxn02228	0	0
rxn02229	0	0
rxn02230	0	0
rxn02236	0	0
rxn02250	0	0
rxn02264	0,000254683	0,000254683
rxn02275	0	0
rxn02283	0	0
rxn02284	-0,031494975	0
rxn02285	-0,031494975	0
rxn02286	0,031494975	0,031494975
rxn02287	-999,9997453	1000
rxn02302	-1000	-0,000254683
rxn02305	0,000254683	0,000254683
rxn02314	0	1000
rxn02315	0	12,02505597
rxn02316	0	12,02505597
rxn02317	-1000	0
rxn02320	0	0
rxn02322	0,000690955	0,000690955
rxn02339	0	0
rxn02341	0,000657835	0,000657835
rxn02346	0	0

rxn02350	0	0
rxn02356	-1000	1000
rxn02358	-1000	1000
rxn02375	0	0
rxn02380	-1000	1000
rxn02400	0	999,7290863
rxn02402	-0,002602787	0
rxn02409	0	0
rxn02432	0	0
rxn02433	0	0
rxn02449	0	0
rxn02454	0	0
rxn02474	-0,000509365	0
rxn02475	0	0,000509365
rxn02476	0,000254683	0,382298998
rxn02484	0,000254683	0,000254683
rxn02495	0	0
rxn02503	0	0,382044316
rxn02504	0	0,382044316
rxn02518	0	0
rxn02521	0	0
rxn02522	0	0
rxn02569	0	0
rxn02571	0	0
rxn02581	0	0
rxn02596	0	0
rxn02597	0	0
rxn02729	0	0
rxn02749	0	0
rxn02751	0	0
rxn02760	0	0
rxn02762	0	0
rxn02774	-999,9997453	0
rxn02775	0	0
rxn02776	0	0
rxn02789	0	0
rxn02795	0	0
rxn02796	0	0
rxn02798	0	0
rxn02811	0	0
rxn02822	0	0
rxn02828	0	0
rxn02853	0	0
rxn02875	0	0
rxn02895	0,000254683	0,000254683
rxn02897	0	0
rxn02914	0	0
rxn02922	0	0
rxn02928	-1000	999,968505
rxn02929	-1000	999,968505

rxn02931	0	0
rxn02936	0	0
rxn02937	0,000254683	0,000254683
rxn02943	0	0
rxn02986	0	0
rxn02988	-0,002602787	0
rxn02993	0	0
rxn03004	0	0,000254683
rxn03005	-0,000254683	0
rxn03030	0,031494975	13,85501143
rxn03039	0	0
rxn03047	0	0
rxn03062	0	0
rxn03075	0	0
rxn03080	0	0,00101873
rxn03084	0,000254683	0,000254683
rxn03086	-13,85501143	-0,031494975
rxn03094	0	0
rxn03095	0	0
rxn03102	0	0
rxn03106	0,000254683	1000
rxn03108	0,000254683	0,000254683
rxn03135	0	0
rxn03136	0	0
rxn03137	0	0
rxn03140	0	0
rxn03141	0	0
rxn03147	0	0
rxn03150	0	0
rxn03164	0,031494975	0,031494975
rxn03167	0	0,382044316
rxn03174	-0,382044316	0
rxn03188	0	0
rxn03194	0	0
rxn03263	0	0
rxn03264	0	0
rxn03269	0	0
rxn03273	0	0
rxn03282	0	0
rxn03333	0	0
rxn03354	0	0
rxn03362	0	0
rxn03372	0	0
rxn03373	0	0
rxn03374	0	0
rxn03379	0	0
rxn03382	0	0
rxn03383	0	0
rxn03387	0	0
rxn03393	0	0

rxn03397	0	0
rxn03405	0	0
rxn03406	0	0
rxn03407	0	0
rxn03408	0,031494975	0,031494975
rxn03409	0	0
rxn03419	0	0,382044316
rxn03421	0	0,382044316
rxn03423	0	0
rxn03445	0	0
rxn03462	0	0
rxn03468	0	0
rxn03481	0	0
rxn03482	0	0
rxn03483	0	0
rxn03491	0	0
rxn03492	0	0
rxn03512	0	0
rxn03513	0	0
rxn03514	0	0
rxn03524	0	0
rxn03535	0	0
rxn03536	0	0
rxn03537	0	0
rxn03538	0	0
rxn03540	0	0
rxn03541	0	0
rxn03548	0	1000
rxn03549	0	0
rxn03552	0	0
rxn03553	0	0
rxn03558	0	0
rxn03598	0	0
rxn03599	0	0
rxn03638	0,062989949	0,06298995
rxn03639	0	0
rxn03642	0,000690955	0,126323666
rxn03838	0	0
rxn03839	0	0
rxn03841	0	0,382044316
rxn03852	0	0
rxn03861	0	0
rxn03869	0	0
rxn03886	0	0
rxn03891	0	0
rxn03901	0,031494975	0,031494975
rxn03902	0	0
rxn03903	0	0
rxn03904	0,031494975	0,031494975
rxn03907	0	0

rxn03908	0	0
rxn03909	0	0
rxn03910	0	0
rxn03933	0	0
rxn03958	0	0
rxn03962	0	0
rxn03964	0	0
rxn03974	-0,028334856	0
rxn03975	-0,028334856	0
rxn03990	0	0
rxn03991	0	0
rxn04045	0	0
rxn04046	0	0
rxn04047	0	0
rxn04048	0	0
rxn04050	0	0
rxn04051	0	1000
rxn04052	0	0
rxn04068	0	0
rxn04113	0	0
rxn04140	0	0
rxn04234	0	0
rxn04308	0	0
rxn04384	0	0
rxn04385	0	0
rxn04413	0	0
rxn04432	0	0
rxn04443	0	0
rxn04482	0	0
rxn04571	0	0
rxn04674	0	0
rxn04676	-0,268981651	1000
rxn04678	-1000	0,268981651
rxn04704	0	0
rxn04726	0	0
rxn04736	0	0
rxn04750	0	0
rxn04792	0	0
rxn04794	0	1000
rxn04840	0	0
rxn04841	0	0
rxn04865	0	0
rxn04866	0	0
rxn04908	0	0
rxn04934	0	0
rxn04938	0	0
rxn04954	-0,226690271	0
rxn04960	0	0
rxn05005	-1000	0
rxn05006	-1000	0

rxn05010	0	0
rxn05011	0	0
rxn05029	0	0
rxn05030	6,28141E-05	6,28141E-05
rxn05039	0	0,000509365
rxn05040	0	0,00101873
rxn05044	0	0
rxn05050	0	0
rxn05054	0	0
rxn05087	0	0
rxn05088	0	0
rxn05108	0	0
rxn05115	0	0
rxn05124	0	0
rxn05233	0	0
rxn05234	0	0
rxn05236	0	0
rxn05239	0	0
rxn05256	0	0
rxn05269	0	0
rxn05289	0	0
rxn05336	0	0
rxn05337	0	0
rxn05338	0	0
rxn05339	0	0
rxn05340	0	0
rxn05341	0	0
rxn05342	0	0
rxn05465	0	0
rxn05733	0	0
rxn05740	-1000	998,5319107
rxn05762	0	0
rxn05778	0	0
rxn05779	0	0
rxn05853	0	0
rxn05871	0	0
rxn05872	0	0
rxn05874	0	0
rxn05878	0	0
rxn05893	-0,1	0
rxn05899	0	0
rxn05901	0	0
rxn05918	0	0
rxn05927	0	0
rxn05934	0	0
rxn05937	-1000	1000
rxn05938	-13,1899155	0
rxn05939	-0,000111923	1000
rxn05940	-1000	0,218733442
rxn05957	0	1000

rxn05958	0	0
rxn05962	0	0
rxn05964	0	0
rxn05965	0	0
rxn05966	0	0
rxn05979	0	0
rxn06005	0	0
rxn06023	0	0
rxn06033	0	0
rxn06043	0	12,02505597
rxn06045	0	12,02505597
rxn06056	0	0
rxn06075	0	0
rxn06078	0	0
rxn06079	0	12,02505597
rxn06080	0	12,02505597
rxn06087	0	0
rxn06089	0	0
rxn06096	0	0
rxn06108	-1000	0
rxn06109	-0,425820012	0
rxn06139	0	12,02505597
rxn06140	0	0
rxn06181	0	1000
rxn06182	0	1000
rxn06190	0	0
rxn06194	0	0
rxn06195	0	0
rxn06196	0	0
rxn06200	0	0
rxn06201	0	0
rxn06217	0	0
rxn06218	0	0
rxn06219	0	0
rxn06231	0	0
rxn06251	0	0
rxn06280	0	0
rxn06285	0	0
rxn06298	0	0
rxn06300	0	0
rxn06312	0	0
rxn06316	0	0
rxn06328	0	0
rxn06347	0	0
rxn06348	0	0
rxn06362	0	0
rxn06365	0	0
rxn06373	0	0
rxn06381	0	0
rxn06425	0	0

rxn06432	0	0
rxn06434	0	0
rxn06435	0	0
rxn06437	0	0
rxn06438	0	0
rxn06439	0	0
rxn06440	0	0
rxn06441	0	0
rxn06443	0	0
rxn06444	0	0
rxn06445	0	0
rxn06446	0	0
rxn06447	0	0
rxn06448	0	0
rxn06449	0	0
rxn06485	0	0
rxn06493	0	0
rxn06500	0	0
rxn06522	0	0
rxn06538	0	0
rxn06556	0	0
rxn06565	0	0
rxn06584	0	0
rxn06591	0,00203746	0,00203746
rxn06592	0	0
rxn06595	0	0
rxn06624	0	0
rxn06641	0	0
rxn06648	0	0
rxn06664	0	0
rxn06671	0	0
rxn06673	0	0
rxn06678	0	0
rxn06701	0	0
rxn06717	0	0
rxn06726	0	0
rxn06737	0	0
rxn06751	0	0
rxn06768	0	0
rxn06798	0	0
rxn06799	0	0
rxn06820	0	0
rxn06831	0	0
rxn06860	0	0
rxn06864	0	0
rxn06882	0	0
rxn06887	0	0
rxn06889	0	1000
rxn06890	0	0
rxn06936	0	0

rxn06937	0,00203746	0,00203746
rxn06947	0	0
rxn06958	0	0
rxn06968	0	0
rxn06979	0	0
rxn07056	0	0
rxn07059	0	0
rxn07099	0	0
rxn07122	0	1000
rxn07189	0	0
rxn07193	0	0
rxn07199	0	0
rxn07251	0	0
rxn07256	0	0
rxn07258	0	0
rxn07264	0	0
rxn07267	0	0
rxn07292	0	0
rxn07437	0	0
rxn07438	0	0
rxn07441	0	13,82351645
rxn07456	0	0,425820012
rxn07465	0,007702147	0,007702147
rxn07466	-1000	1000
rxn07473	0	0
rxn07475	0	0
rxn07489	0	0
rxn07573	0	0
rxn07577	0	0
rxn07584	0	0
rxn07585	0	0
rxn07586	0	0
rxn07587	0	0
rxn07588	-1000	0
rxn07589	-1000	0
rxn07679	0	0
rxn07683	0	0
rxn07687	0	0
rxn07804	0	0
rxn07807	0	0
rxn07832	0	0
rxn07846	0	0
rxn07849	0	0
rxn07987	0	0
rxn07989	0	0
rxn07991	0	0
rxn07992	0	0
rxn07993	0	0
rxn07994	0	0
rxn08025	0	0

rxn08043	0	0
rxn08067	-1000	1000
rxn08083	0	0
rxn08084	0	0
rxn08085	0	0
rxn08086	0	0
rxn08087	0	0
rxn08088	0	0
rxn08089	0	0
rxn08094	0	1000
rxn08126	0	0
rxn08127	0	0
rxn08128	0	0
rxn08129	0	0
rxn08133	0	0
rxn08171	0	0
rxn08180	0	0
rxn08194	-1000	1000
rxn08206	0	0
rxn08207	0	0
rxn08208	0	0
rxn08209	0	0
rxn08294	0	1000
rxn08295	0	1000
rxn08296	0	1000
rxn08297	0	1000
rxn08298	0	1000
rxn08299	0	1000
rxn08300	0	1000
rxn08306	0	0
rxn08307	0	0
rxn08308	0	0
rxn08309	0	0
rxn08310	0	0
rxn08311	0	0
rxn08312	0	0
rxn08352	0	0
rxn08413	0	0
rxn08433	0	0
rxn08434	0	0
rxn08435	0	0
rxn08436	0	0
rxn08437	0	0
rxn08438	0	0
rxn08519	0,057583371	0,057583371
rxn08546	0	0
rxn08547	0	0
rxn08548	0	0
rxn08549	0	0
rxn08550	0	0

rxn08551	0	0
rxn08552	0	0
rxn08582	0	0,5
rxn08605	0	0
rxn08607	0	0
rxn08615	-1000	998,5319107
rxn08647	0	0
rxn08668	0	0
rxn08669	0	0
rxn08796	0	0
rxn08797	0	0
rxn08798	0	0
rxn08799	0	0
rxn08800	0	0
rxn08801	0	0
rxn08802	0	0
rxn08803	0	0
rxn08804	0	0
rxn08805	0	0
rxn08806	0	0
rxn08807	0	0
rxn08808	0	0
rxn08809	0	0
rxn08810	0	0
rxn08811	0	0
rxn08812	0	0
rxn08813	0	0
rxn08814	0	0
rxn08815	0	0
rxn08816	0	0
rxn08817	0	0
rxn08818	0	0
rxn08819	0	0
rxn08820	0	0
rxn08821	0	0
rxn08822	0	0
rxn08823	0	0
rxn08838	0	0
rxn08839	0	0
rxn08840	0	0
rxn08841	0	0
rxn08842	0	0
rxn08843	0	0
rxn08844	0	0
rxn08845	0	0
rxn08846	0	0
rxn08847	0	0
rxn08848	0	0
rxn08849	0	0
rxn08850	0	0

rxn08851	0	0
rxn08857	0	0
rxn08889	0,000768616	0,000768616
rxn08890	0,006222019	0,006222019
rxn08891	0,000768616	0,000768616
rxn08892	-999,9854054	1000
rxn08893	-999,9923184	999,993087
rxn08894	-999,9854054	1000
rxn08897	-0,006912974	-0,006912974
rxn08926	0,000690955	0,000690955
rxn08927	-999,9984639	999,9869415
rxn08928	-999,9854054	1000
rxn08929	0,00153609	0,00153609
rxn08930	0	0
rxn08958	0,000768616	0,000768616
rxn09010	0	0
rxn09062	0	1000
rxn09063	0	1000
rxn09064	0	1000
rxn09065	0	1000
rxn09066	0	1000
rxn09067	0	1000
rxn09068	0	1000
rxn09069	0	0
rxn09101	0	0
rxn09102	0	0
rxn09103	0	0
rxn09104	0	0
rxn09105	0	0
rxn09106	0	0
rxn09107	0	0
rxn09108	0	0
rxn09109	0	0
rxn09110	0	0
rxn09111	0	0
rxn09112	0	0
rxn09113	0	0
rxn09114	0	0
rxn09123	0	0
rxn09124	0	0
rxn09125	0	0
rxn09126	0	0
rxn09127	0	0
rxn09128	0	0
rxn09129	0	0
rxn09130	0	0
rxn09131	0	0
rxn09132	0	0
rxn09133	0	0
rxn09134	0	0

rxn09135	0	0
rxn09136	0	0
rxn09137	0	0
rxn09138	0	0
rxn09139	0	0
rxn09140	0	0
rxn09141	0	0
rxn09142	0	0
rxn09143	0	0
rxn09144	0	0
rxn09145	0	0
rxn09146	0	0
rxn09147	0	0
rxn09148	0	0
rxn09149	0	0
rxn09150	0	0
rxn09151	0	0
rxn09152	0	0
rxn09153	0	0
rxn09154	0	0
rxn09155	0	0
rxn09156	0	0
rxn09157	0	0
rxn09158	0	0
rxn09159	0	0
rxn09160	0	0
rxn09161	0	0
rxn09162	0	0
rxn09163	0	0
rxn09164	0	0
rxn09177	0	0,000657835
rxn09235	0,028334856	0,028334856
rxn09237	0,029248515	0,029248515
rxn09240	0	0
rxn09244	0	0
rxn09264	0	0
rxn09265	0	0
rxn09340	0	0
rxn09341	0	12,02505597
rxn09348	0	12,02505597
rxn09355	0	0
rxn09399	0	0
rxn09454	0	0
rxn09455	0	0
rxn09456	0	0
rxn09473	0	0
rxn09486	0	0
rxn09502	0	1000
rxn09531	0	0
rxn09557	0,000254683	1000

rxn09615	0,000690955	0,000690955
rxn09631	0,000254683	0,000254683
rxn09632	0	719,2471183
rxn09633	0,000254683	0,000254683
rxn09888	0	0
rxn09949	0	0
rxn09952	0	0
rxn09992	0	0
rxn09995	0	0
rxn10003	0	0,000657835
rxn10052	-438,4942365	1000
rxn10054	0	12,0257138
rxn10056	0	0,000510507
rxn10058	0	0,000510507
rxn10060	0	0,000510507
rxn10091	-1000	1000
rxn10107	0	0
rxn10192	0	0
rxn10193	0	0
rxn10202	0	0
rxn10203	0	0
rxn10204	0	0
rxn10205	0	0
rxn10206	0	0
rxn10207	0	0
rxn10208	0	0
rxn10209	0	0
rxn10210	0	0
rxn10211	0	0
rxn10212	0	0
rxn10213	0	0
rxn10214	0	0
rxn10215	0	0
rxn10216	0	0
rxn10217	0	0
rxn10218	0	0
rxn10219	0	0
rxn10220	0	0
rxn10221	0	0
rxn10222	0	0
rxn10223	0	0
rxn10224	0	0
rxn10225	0	0
rxn10238	0	1000
rxn10239	0	1000
rxn10240	0	1000
rxn10241	0	1000
rxn10242	0	1000
rxn10243	0	1000
rxn10253	0	1000

rxn10254	0	1000
rxn10255	0	1000
rxn10256	0	1000
rxn10257	0	1000
rxn10258	0	1000
rxn10259	0	0
rxn10260	0	0
rxn10261	0	0
rxn10262	0	0
rxn10263	0	0
rxn10264	0	0
rxn10265	0	0
rxn10266	0	0
rxn10267	0	0
rxn10268	0	0
rxn10269	0	0
rxn10270	0	0
rxn10289	0	0
rxn10290	0	0
rxn10291	0	0
rxn10292	0	0
rxn10293	0	0
rxn10294	0	0
rxn10295	0	0
rxn10296	0	0
rxn10297	0	0
rxn10298	0	0
rxn10299	0	0
rxn10300	0	0
rxn10301	0	0
rxn10302	0	0
rxn10303	0	0
rxn10304	0	0
rxn10305	0	0
rxn10306	0	0
rxn10363	0	0
rxn10410	0	0
rxn10618	0,000690955	6,595648703
rxn10785	6,28141E-05	6,28141E-05
rxn10790	0,000254683	1000
rxn10798	-1000	-0,000254683
rxn10951	0	0,028334856
rxn11007	0,028334856	0,028334856
rxn11213	0,000254683	0,382298998
rxn11510	0	0
rxn11513	0	0
rxn11547	0	0
rxn11548	0	0
rxn11550	0	0
rxn11567	0	0

rxn11571	0	0
rxn11587	0	0
rxn11599	0	0
rxn11609	0	0
rxn11641	0	0
rxn11642	0	0
rxn11676	0	0
rxn11732	0	0
rxn11735	0	0
rxn11749	0	0
rxn11755	0	0
rxn11759	0	0
rxn11760	0	0
rxn11761	0	0
rxn11765	0	0
rxn11766	0	0
rxn11768	0	0
rxn11772	0	0
rxn11773	0	0
rxn11788	0	0
rxn11809	0	0
rxn11834	0	0
rxn11838	0	0
rxn11878	0	0
rxn11879	0	0
rxn11897	0	0
rxn11899	0	0
rxn11951	0	0
rxn11965	0	0
rxn11977	0	0
rxn11984	0	0
rxn12013	0	0
rxn12033	0	0
rxn12049	0	0
rxn12053	0	0
rxn12054	0	0
rxn12218	-1000	-0,000254683
rxn12221	0,000254683	1000
rxn12510	0,000657835	0,000657835
rxn12649	-1000	0
rxn12778	0	0
rxn12844	0	0
rxn12845	0	0
rxn12846	0	0
rxn12847	0	0
rxn13420	0,000690955	1000
rxn13421	0,000690955	1000
rxn13705	0	0
rxn13713	0,000690955	0,126323666
rxn13741	0	0

rxn13768	0,00138191	13,19129741
rxn13906	-0,007702147	-0,007702147
rxn13936	0,015363179	0,01536318
rxn13953	-1000	-0,000254683
rxn13974	-13,1899155	0
rxn14028	0	0
rxn14043	0	0
rxn14048	-1000	0
rxn14054	-1000	0
rxn14063	0	0
rxn14070	0	0
rxn14089	-1000	0
rxn14094	0	0
rxn14120	-1000	0,381025586
rxn14132	0	0
rxn14142	0	0
rxn14191	0	0
rxn14261	0	0
rxn14270	0	0
rxn14279	0	0
rxn14346	0	0
rxn90002	-12,30021585	11,99580745
rxn90003	0	0
rxn90004	0	0
rxn90005	-0,028845363	-0,028334856
rxn08173	0	500
Biomass_Bacteria	1,142074	1,142074006
t_Cl	0,005153038	0,005153038
t_Sulfate	0,004294198	0,004294198
t_Cu2+	0,003435359	0,003435359
t_Mg	0,008587254	0,008587254
t_Ca2+	0,005153038	0,005153038
t_NH3	-2,208599842	0
t_H2O	-11,85351759	9,919428277
t_Biomass	-1,142074006	-1,142074
t_Butyrate	-6,595648703	-0,000690955
t_D-Lactate	0	0
t_Ethanol	-1,308245774	0
t_Formate	-13,93850505	0
t_L-Lactate	-13,1899155	0
t_Nitrite	0	0,1
t_Phosphate	1,51196664	2,011966651
t_Propionate	-0,218733442	0
t_O2	0	0
t_D-Glucose	0	0,5
t_CO2	-13,93850505	0
t_Acetate	-13,85501143	-0,031494975
t_Succinate	-0,425820012	0
t_H2S	-0,397709	0
Ex_Cl	-0,005153038	-0,005153038

Ex_Sulfate	-0,004294198	-0,004294198
Ex_Cu2+	-0,003435359	-0,003435359
Ex_Mg	-0,008587254	-0,008587254
Ex_Ca2+	-0,005153038	-0,005153038
Ex_NH3	0	2,208599842
Ex_H2O	-9,919428277	11,85351759
Ex_Biomass	1,142074	1,142074006
Ex_Butyrate	0,000690955	6,595648703
Ex_D-Lactate	0	0
Ex_Ethanol	0	1,308245774
Ex_Formate	0	13,93850505
Ex_L-Lactate	0	13,1899155
Ex_Nitrite	-0,1	0
Ex_Phosphate	-2,011966651	-1,51196664
Ex_Propionate	0	0,218733442
Ex_O2	0	0
Ex_D-Glucose	-0,5	0
Ex_CO2	0	13,93850505
Ex_Acetate	0,031494975	13,85501143
Ex_Succinate	0	0,425820012
Ex_H2S	0	0,397709
t_Fe2	0,007983097	0,007983097
t_fe3	0,007728415	0,007728415
t_Acetaldehyde	-1,308245774	0
t_Adenosine	0	0,5
t_AMP	0	0,5
t_Amylotriose	0	0
t_BIOT	0	0
t_Choline	0	0
t_Cytidine	0	0
t_Cytosine	0	0
t_DAlanine	0	0
t_Deoxyadenosine	0	0,5
t_Deoxycytidine	0	0,365829146
t_Deoxyguanosine	0	0
t_Deoxyinosine	0	0
t_Deoxyuridine	0	0
t_DRibose	0	0,5
t_DSerine	0	0
t_Glycerol	0	0
t_GSH	0	0
t_Guanine	0	0
t_H2S2O3	0	0
t_Heme	0,000254683	0,000254683
t_Homocysteine	0	0
t_HYXN	0	0,5
t_Inosine	0	0,5
t_LACT	0	0,5
t_LAlanine	-1,708599842	0,5
t_LArginine	0,374367289	0,5

t_LAsparagine	0,074179988	0,5
t_LAspartate	0,074179988	0,5
t_LCysteine	0,102291	0,5
t_LGlutamate	0,117955684	0,5
t_LGlutamine	0,117955684	0,5
t_LHistidine	0,105185015	0,105185016
t_LInositol	0	0
t_LIsoleucine	0,32240749	0,322407492
t_LLeucine	0,499999997	0,5
t_LLysine	0,380767472	0,380767474
t_LMethionine	-0,047457746	0,171275697
t_LPhenylalanine	0,20557332	0,205573321
t_LThreonine	0,281521241	0,5
t_LTryptophan	0,063076747	0,063076747
t_LTyrosine	0,153037916	0,153037917
t_LValine	0,470420281	0,470420283
t_Maltose	0	0,5
t_Niacin	0	0,002602787
t_Ornithine	0	0
t_PPi	0	0
t_Pyridoxol	0	0
t_XAN	0	0
t_5Deoxyadenosine	0	0
t_Acetoacetate	-0,125632711	0
t_BET	0	0
t_Calomide	0	0
t_Cbl	0	0
t_Citrate	0	0
t_CysGly	0	0
t_Glycine	-1,708599842	0,5
t_Glycolaldehyde	0	0
t_LProline	0,245317495	0,245317497
t_Maltohexaose	0	0
t_Methanol	0	0
t_NAcetylDglucosamine	0	0
t_PM	0	0
t_Putrescine	0	0
t_Pyridoxal	0,000254683	0,000254683
t_Riboflavin	0	0,000509365
t_Salicin	0	0
t_Sorbitol	0	0
t_Spermidine	0	0
t_Sucrose	0	0,5
t_Taurine	0	0
t_Thiamin	0	0
t_Thymidine	0	0
t_Thyminose	0	0,5
t_TRHL	0	0
t_Tyramine	0	0
t_Uracil	0	0,365829146

t_Uridine	0	0,365829146
t_Ursin	0	0
t_Mn2+	0,003435359	0,003435359
t_Formaldehyde	0	0
t_Fumarate	-0,425820012	0
t_Oxidized glutathione	0	0
t_Adenine	0	0
t_Nicotinamide	0	0
t_4-Hydroxybenzoate	0	0
t_Co2+	0,003435359	0,003435359
t_D-Glutamate	0	0
t_Nitrate	0	0
t_Chorismate	0	0
t_Folate	-0,381025586	0,00101873
t_N-Acetyl-D-mannosamine	0	0
t_Siroheme	0	0
t_Selenate	0	0
t_Menaquinone 7	0	0
t_2-Demethylmenaquinone 8	0	0
t_Menaquinone 8	0	0
t_Ubiquinone-8	0	0
t_2-Oxobutyrate	0	0
t_ABEE	0	0
t_Neu5Ac	0	0
t_Glycerol-3-phosphate	0	0
t_H+	-1000	0,5
t_indol	0	0
t_Nicotinamide ribonucleotide	0	0
t_PAN	0,000657835	0,000657835
t_Pyridoxal phosphate	0	0
t_Zn2+	0,003435359	0,003435359
t_1,2-Diacyl-sn-glycerol dioctadecanoyl	0	0
t_meso-2,6-Diaminopimelate	0	0
t_L-Serine	0,239492918	0,5
t_D-Fructose	0	0,5
t_D-Mannose	0	0
t_Oxalate	0	0
t_beta D-Galactose	0	0,5
t_L-Fucose	0	0
Ex_Fe2	-0,007983097	-0,007983097
Ex_fe3	-0,007728415	-0,007728415
Ex_Acetaldehyde	0	1,308245774
Ex_Adenosine	-0,5	0
Ex_AMP	-0,5	0
Ex_Amylotriose	0	0
Ex_BIOT	0	0
Ex_Choline	0	0
Ex_Cytidine	0	0
Ex_Cytosine	0	0
Ex_DAlanine	0	0

Ex_Deoxyadenosine	-0,5	0
Ex_Deoxycytidine	-0,365829146	0
Ex_Deoxyguanosine	0	0
Ex_Deoxyinosine	0	0
Ex_Deoxyuridine	0	0
Ex_DRibose	-0,5	0
Ex_DSerine	0	0
Ex_Glycerol	0	0
Ex_GSH	0	0
Ex_Guanine	0	0
Ex_Heme	-0,000254683	-0,000254683
Ex_Homocysteine	0	0
Ex_HYXN	-0,5	0
Ex_Inosine	-0,5	0
Ex_LACT	-0,5	0
Ex_LAlanine	-0,5	1,708599842
Ex_LArginine	-0,5	-0,374367289
Ex_LAsparagine	-0,5	-0,074179988
Ex_LAspartate	-0,5	-0,074179988
Ex_LCysteine	-0,5	-0,102291
Ex_LGlutamate	-0,5	-0,117955684
Ex_LGlutamine	-0,5	-0,117955684
Ex_LHistidine	-0,105185016	-0,105185015
Ex_LInositol	0	0
Ex_LIsoleucine	-0,322407492	-0,32240749
Ex_LLeucine	-0,5	-0,499999997
Ex_LLysine	-0,380767474	-0,380767472
Ex_LMethionine	-0,171275697	0,047457746
Ex_LPhenylalanine	-0,205573321	-0,20557332
Ex_LThreonine	-0,5	-0,281521241
Ex_LTryptophan	-0,063076747	-0,063076747
Ex_LTyrosine	-0,153037917	-0,153037916
Ex_LValine	-0,470420283	-0,470420281
Ex_Maltose	-0,5	0
Ex_Niacin	-0,002602787	0
Ex_Ornithine	0	0
Ex_PPi	0	0
Ex_XAN	0	0
Ex_5Deoxyadenosine	0	0
Ex_Acetoacetate	0	0,125632711
Ex_BET	0	0
Ex_Calomide	0	0
Ex_Cbl	0	0
Ex_Citrate	0	0
Ex_CysGly	0	0
Ex_Glycine	-0,5	1,708599842
Ex_Glycolaldehyde	0	0
Ex_LProline	-0,245317497	-0,245317495
Ex_Maltohexaose	0	0
Ex_Methanol	0	0

Ex_NAcetylDglucosamine	0	0
Ex_PM	0	0
Ex_Putrescine	0	0
Ex_Pyridoxal	-0,000254683	-0,000254683
Ex_Riboflavin	-0,000509365	0
Ex_Salicin	0	0
Ex_Sorbitol	0	0
Ex_Spermidine	0	0
Ex_Sucrose	-0,5	0
Ex_Taurine	0	0
Ex_Thiamin	0	0
Ex_Thymidine	0	0
Ex_Thyminose	-0,5	0
Ex_TRHL	0	0
Ex_Tyramine	0	0
Ex_Uracil	-0,365829146	0
Ex_Uridine	-0,365829146	0
Ex_Ursin	0	0
Ex_Mn2+	-0,003435359	-0,003435359
Ex_Formaldehyde	0	0
Ex_Fumarate	0	0,425820012
Ex_Oxidized glutathione	0	0
Ex_Adenine	0	0
Ex_Nicotinamide	0	0
Ex_4-Hydroxybenzoate	0	0
Ex_Co2+	-0,003435359	-0,003435359
Ex_D-Glutamate	0	0
Ex_Nitrate	0	0
Ex_Folate	-0,00101873	0,381025586
Ex_N-Acetyl-D-mannosamine	0	0
Ex_Siroheme	0	0
Ex_Selenate	0	0
Ex_Menaquinone 7	0	0
Ex_2-Demethylmenaquinone 8	0	0
Ex_Menaquinone 8	0	0
Ex_Ubiquinone-8	0	0
Ex_ABEE	0	0
Ex_Neu5Ac	0	0
Ex_H+	-0,5	1000
Ex_indol	0	0
Ex_Nicotinamide ribonucleotide	0	0
Ex_PAN	-0,000657835	-0,000657835
Ex_Zn2+	-0,003435359	-0,003435359
Ex_1,2-Diacyl-sn-glycerol dioctadecanoyl	0	0
Ex_L-Serine	-0,5	-0,239492918
Ex_D-Fructose	-0,5	0
Ex_D-Mannose	0	0
Ex_Oxalate	0	0
Ex_beta D-Galactose	-0,5	0
Ex_L-Fucose	0	0

t_Starch	0	0,005
t_octanoate	0	0
t_Melibiose	0	0,5
t_Amylose	0	0
Ex_Starch	-0,005	0
Ex_Melibiose	-0,5	0
Ex_Amylose	0	0
t_Raffinose_Melitose	0	0
t_Isovaleric_acid	0	0
t_H2O2	0	0
t_Nitric_oxide	0	0
Ex_Raffinose_Melitose	0	0
Ex_Isovaleric_acid	0	0
Ex_H2O2	0	0
Ex_Nitric_oxide	0	0
rxn01207_1	0	0
rxn08972	0	0
rxn08973	0	0
rxn06111	0	1000
rxn13726	0	0
rxn13727	0	0
rxn13729	0	0
rxn08974	0	0
rxn10122	0	0
rxn10123	0	0
rxn10124	0	0
rxn12665	0	0
rxn06097	0	0,005
t_Sulfite	0	0
Ex_Sulfite	0	0

rxn ID	minFlux	max Flux
rxn00001	0,883536627	948,0941742
rxn00015	0,005915331	0,005915333
rxn00016	0	0
rxn00020	0	0
rxn00022	0	0
rxn00029	0,000782396	0,000782396
rxn00060	0,000195599	0,000195599
rxn00062	0	947,2106375
rxn00063	0	473,6053188
rxn00065	0	0
rxn00076	0	473,6053188
rxn00077	0	0,000392075
rxn00085	-0,088638023	0
rxn00097	-999,6526236	-52,18228161
rxn00100	0,000505224	0,000505224
rxn00105	-0,088638023	473,6053188
rxn00106	-1000	-52,78936247
rxn00114	0	0
rxn00119	0,283387685	473,8887064
rxn00122	0,000195599	0,000195599
rxn00124	0,000195599	0,000195599
rxn00126	0,000586797	0,000586797
rxn00137	0	0
rxn00139	-0,174159363	-0,000391198
rxn00143	0,000391198	0,000391198
rxn00151	-0,24486802	0
rxn00157	-0,859885421	0
rxn00159	-1000	999,9994693
rxn00161	-1000	999,9994693
rxn00162	0	0,264446327
rxn00173	-0,165336465	947,7097148
rxn00175	0	947,2106375
rxn00176	0	0
rxn00184	-0,57577573	0
rxn00190	0,001998968	0,090636991
rxn00193	0,024188476	0,024188484
rxn00194	0,000335062	0,288222927
rxn00206	0,000195599	0,000195599
rxn00212	0	473,6053188
rxn00213	-474,1720941	-0,307576161
rxn00214	0	0
rxn00221	0	0
rxn00222	0	0
rxn00224	0,000195599	0,000195599
rxn00225	-948,2075202	-0,087600949
rxn00226	0	0
rxn00239	0,205870009	0,450738029
rxn00242	0	0,24486802
rxn00250	-2,71909E-05	-2,71909E-05

rxn00260	2,71909E-05	0,264473518
rxn00272	-1000	1000
rxn00275	-0,810231375	0,016544488
rxn00283	0,021298349	0,021298356
rxn00290	-0,288418526	-0,000530661
rxn00293	0,048376952	0,048376967
rxn00297	0	0
rxn00301	0	0,24486802
rxn00303	0	0,24486802
rxn00304	-0,450738029	-0,205870009
rxn00307	0	0
rxn00313	0	0,206693966
rxn00321	0	0
rxn00333	0,000195599	0,000195599
rxn00337	0,030103807	0,236797773
rxn00340	0	0,264446327
rxn00346	0	0
rxn00350	-0,000195599	-0,000195599
rxn00359	0	0
rxn00360	0	0
rxn00365	0	0
rxn00367	0	0,088638023
rxn00368	0	0
rxn00369	0	0
rxn00391	0	947,2106375
rxn00392	0,000195599	947,2108331
rxn00395	0	0
rxn00405	0,03509728	0,035097291
rxn00411	-0,001035885	-0,000530661
rxn00412	0,136313996	0,22495202
rxn00416	0	0,088638023
rxn00422	-1000	1000
rxn00424	-1000	1000
rxn00436	0	473,6053188
rxn00440	0,000195599	473,6055144
rxn00453	0	0
rxn00459	0,252741815	0,497609834
rxn00461	0,024188476	0,024188484
rxn00462	0	0
rxn00463	0	473,6053188
rxn00469	0	0
rxn00490	0	0
rxn00499	-0,57577573	0
rxn00514	0	0
rxn00533	2,71909E-05	2,71909E-05
rxn00541	0	0
rxn00547	0,048572551	0,048572566
rxn00548	0	0,497805433
rxn00555	0,048376952	0,048376967
rxn00558	-1000	1000

rxn00559	0	0
rxn00562	0	0
rxn00566	0,005617829	0,714571245
rxn00575	0	0
rxn00585	0	0
rxn00611	0	0
rxn00633	0	0
rxn00641	0	0
rxn00646	0	0
rxn00649	0	0
rxn00650	-0,000195599	-0,000195599
rxn00662	0	0
rxn00670	0,000195599	947,2108331
rxn00674	0	947,2106375
rxn00675	0	0
rxn00684	0	0
rxn00685	0	0,57577573
rxn00686	0	0
rxn00687	0	0,57577573
rxn00689	0	0
rxn00692	-0,022543874	-0,022543867
rxn00695	0,307576161	474,1720941
rxn00704	0	0
rxn00707	0	0
rxn00709	0	0,25919929
rxn00710	0	0
rxn00712	0	0,25919929
rxn00713	0	0,24486802
rxn00715	0	0,25919929
rxn00741	0	0
rxn00742	0,000195599	0,000195599
rxn00745	0	0
rxn00747	0	0
rxn00756	0	0
rxn00762	0	0
rxn00770	0,208455773	0,627091944
rxn00775	0	0
rxn00777	0,252937413	0,497805433
rxn00778	0,461393187	1,124897376
rxn00781	0,252741815	0,497609834
rxn00785	-0,497609834	0,000195599
rxn00786	0	0
rxn00790	-0,000195599	-0,000195599
rxn00799	0,000530661	0,479802443
rxn00801	0	0
rxn00808	0	947,2106375
rxn00816	0	0
rxn00827	0	0
rxn00829	0,000530661	0,000530661
rxn00830	4,82419E-05	4,82419E-05

rxn00834	0,205870009	0,205870073
rxn00836	-0,205870073	-0,205870009
rxn00851	0	947,2106375
rxn00853	0,03509728	0,035097291
rxn00855	0	0
rxn00858	0	0
rxn00871	0	0
rxn00879	0	0
rxn00881	0	0
rxn00882	0	0
rxn00883	0	0
rxn00907	0,000391198	0,000391198
rxn00915	-0,24486802	0
rxn00916	0,117231985	0,450738029
rxn00917	0	0,088638023
rxn00925	0	0
rxn00927	-1000	1000
rxn00942	0	1000
rxn00947	0	473,6053188
rxn00952	0	0,293131823
rxn00953	0,000391198	0,293523021
rxn00955	0,000391198	0,000391198
rxn00979	0,000195599	0,000195599
rxn00980	0	0
rxn00983	0	0
rxn00985	-947,2108331	-0,000195599
rxn00986	0	0
rxn00991	-0,000530661	-0,000530661
rxn00999	0	0
rxn01000	0	0
rxn01016	0	0
rxn01018	0	0
rxn01019	0	0
rxn01021	0	0
rxn01022	0	0
rxn01029	0,03509728	0,035097291
rxn01037	0	0
rxn01041	-1000	1000
rxn01042	-1000	1000
rxn01100	-0,497609834	0
rxn01106	-0,497609834	-0,252741815
rxn01108	-1000	1000
rxn01109	-1000	1000
rxn01116	0,252937413	0,497805433
rxn01122	0	0
rxn01123	0	0
rxn01138	-1000	1000
rxn01146	0	0
rxn01171	0	0
rxn01187	0	0,497805433

rxn01200	0	1000
rxn01201	-0,288418526	-0,000530661
rxn01204	0,000530661	0,288418526
rxn01210	0	0
rxn01211	0,000195599	0,000391198
rxn01213	4,82419E-05	4,82419E-05
rxn01225	0	0,24486802
rxn01226	-999,9775368	1000
rxn01228	0	0
rxn01231	0	0
rxn01255	0,000195599	0,000195599
rxn01256	0	0
rxn01265	-0,001998969	-0,001998968
rxn01274	0	0
rxn01299	-1000	1000
rxn01303	0	0
rxn01304	0	0
rxn01308	0	0
rxn01316	0	0
rxn01329	0	0
rxn01332	0,000195599	0,000195599
rxn01334	0	0
rxn01351	0	0,497609834
rxn01352	-0,022463178	-0,022463171
rxn01358	-1000	1000
rxn01366	-0,25919929	0,021761478
rxn01368	0	0
rxn01370	0	0,25919929
rxn01396	0	0
rxn01405	-0,005915333	-0,005915331
rxn01423	0	0
rxn01426	0	0
rxn01446	-0,022463178	-0,022463171
rxn01466	4,82419E-05	4,82419E-05
rxn01478	0	0
rxn01484	0	0
rxn01485	-0,048376967	-0,048376952
rxn01486	0	0
rxn01500	-0,000530661	-0,000530661
rxn01509	0	0,497609834
rxn01510	0	0
rxn01513	0,021761471	0,021761478
rxn01517	0	0
rxn01518	0,021761471	0,021761478
rxn01519	0	0
rxn01539	-473,6055144	-0,000195599
rxn01544	-0,24486802	0
rxn01545	-1000	1000
rxn01548	-999,9775368	1000
rxn01594	0	0

rxn01601	0	0
rxn01602	0	0
rxn01603	0	0
rxn01621	0	0
rxn01629	-0,001564791	-0,001564791
rxn01643	-0,236797773	-0,030103807
rxn01644	0,024188476	0,230882442
rxn01646	-1000	0
rxn01647	0	0
rxn01648	0	0
rxn01649	-1000	1000
rxn01650	0	0
rxn01669	0	0,088638023
rxn01675	0	0
rxn01679	0	0
rxn01683	-1000	1000
rxn01684	-1000	1000
rxn01686	0	0
rxn01706	0	0
rxn01735	0	0
rxn01739	0,000195599	0,000195599
rxn01740	-0,000195599	-0,000195599
rxn01780	0	0
rxn01784	0	0
rxn01799	-0,021761478	0,022463178
rxn01800	0	0,044224656
rxn01807	0	0
rxn01827	0	0
rxn01859	0	0,044224656
rxn01860	0	0
rxn01870	0	0
rxn01892	0	0
rxn01953	0	0
rxn01962	0	0
rxn01967	0	0
rxn01972	0,024188476	0,230882442
rxn01973	0	0
rxn01974	0,024188476	0,230882442
rxn01977	-1000	1000
rxn01982	0	0
rxn01985	0	0
rxn01997	0	0
rxn02000	0	0
rxn02003	0	0
rxn02008	0,024188476	0,024188484
rxn02011	0,024188476	0,024188484
rxn02012	0	0
rxn02015	0	0
rxn02046	0	0
rxn02128	0	0

rxn02138	0	0
rxn02139	0	0
rxn02154	0	473,6053188
rxn02155	0,001998968	473,6073177
rxn02160	0	0
rxn02171	0,000530661	0,288418526
rxn02175	0,000505224	473,605824
rxn02176	0	473,6053188
rxn02199	0	0
rxn02212	0,000195599	0,000195599
rxn02213	0,000195599	0,000195599
rxn02222	0	0
rxn02228	0	0
rxn02264	0,000195599	0,000195599
rxn02284	-0,024188484	0
rxn02285	-0,024188484	0
rxn02286	0,024188476	0,024188484
rxn02302	-0,293327422	-0,000195599
rxn02305	0,000195599	0,000195599
rxn02322	0,000530661	0,000530661
rxn02341	0,000505224	0,000505224
rxn02351	0	0
rxn02377	0	0
rxn02380	-1000	1000
rxn02402	0	0
rxn02409	0	0
rxn02444	0	0
rxn02449	0	0,24486802
rxn02476	0,000195599	0,000195599
rxn02483	0	0
rxn02484	0,000195599	0,000195599
rxn02495	0	0
rxn02518	0	0
rxn02571	0	0
rxn02581	0	0
rxn02632	0	0
rxn02749	0	0
rxn02751	0	0
rxn02775	0	0
rxn02789	0	0
rxn02795	0	0
rxn02796	0	0
rxn02798	0	0
rxn02811	0	0
rxn02834	0	0
rxn02835	0	0
rxn02875	0	0
rxn02884	0	0
rxn02885	0	0
rxn02895	0,000195599	0,000195599

rxn02897	0	0
rxn02900	0	0
rxn02922	0	0
rxn02929	-0,230882442	-0,024188476
rxn02931	0	0
rxn02936	0	0
rxn02937	0,000195599	0,000195599
rxn02943	0	0
rxn03004	0	0,000195599
rxn03005	-0,000195599	0
rxn03030	0,024188476	0,230882442
rxn03036	0	0
rxn03040	0	0
rxn03041	0	0
rxn03047	0	0
rxn03057	0	0
rxn03064	0	0
rxn03080	0	0
rxn03084	0,000195599	0,000195599
rxn03086	-0,230882442	-0,024188476
rxn03094	0	0
rxn03095	0	0
rxn03102	0	0
rxn03108	0,000195599	0,000195599
rxn03123	0	0
rxn03140	0	0
rxn03141	0	0
rxn03150	0	0
rxn03158	0	0
rxn03164	0,024188476	0,024188484
rxn03182	0	0
rxn03263	0	0
rxn03264	0	0
rxn03269	0	0
rxn03273	0	0
rxn03371	0	0
rxn03374	0	0
rxn03380	0	0
rxn03387	0	0
rxn03397	0	0
rxn03402	0	0
rxn03405	0	0
rxn03408	0,024188476	0,024188484
rxn03439	0	0
rxn03462	0	0
rxn03538	0	0
rxn03558	0	0
rxn03587	0	0
rxn03638	0,048376952	0,048376967
rxn03641	0,000530661	0,288418526

rxn03642	0,000530661	0,288418526
rxn03852	0	0
rxn03891	4,82419E-05	4,82419E-05
rxn03900	0	0
rxn03901	0,024188476	0,024188484
rxn03902	0	0
rxn03903	0	0
rxn03904	0,024188476	0,024188484
rxn03916	0	0
rxn03917	0	0
rxn03918	0	0
rxn03919	0	0
rxn03933	0	0
rxn03958	0	0
rxn04045	0	0
rxn04046	0	0
rxn04068	0	0
rxn04142	0	0
rxn04385	0	0
rxn04432	0	0
rxn04443	0	0
rxn04674	0	0
rxn04676	0	1000
rxn04678	-1000	0
rxn04681	0	0
rxn04682	0	0
rxn04704	0	0
rxn04786	0,005915331	0,005915333
rxn04794	0	0,958543564
rxn04822	0	0
rxn04954	-0,000195599	-0,000195599
rxn05028	4,82419E-05	4,82419E-05
rxn05030	4,82419E-05	4,82419E-05
rxn05039	0	0
rxn05124	0	0
rxn05234	0	0
rxn05236	0	0
rxn05247	0	0
rxn05248	0	0
rxn05249	0	0
rxn05250	0	0
rxn05251	0	0
rxn05252	0	0
rxn05269	0	0
rxn05289	0	0
rxn05329	0	0
rxn05330	0	0
rxn05331	0	0
rxn05332	0	0
rxn05333	0	0

rxn05334	0	0
rxn05335	0	0
rxn05336	0	0
rxn05337	0	0
rxn05338	0	0
rxn05339	0	0
rxn05340	0	0
rxn05341	0	0
rxn05342	0	0
rxn05733	0	0
rxn05736	0	473,6053188
rxn05759	-0,481676787	0
rxn05856	0	0
rxn05878	0	0
rxn05901	0	0
rxn05902	-0,1	0
rxn05909	0	0,293131823
rxn05938	-0,859885421	0
rxn05939	0,000444702	0,288332567
rxn05940	-0,958347965	0,000195599
rxn05958	0	0
rxn05962	0	0
rxn05970	0	0
rxn05979	0	0
rxn06005	0	0
rxn06043	0	0
rxn06045	0	0
rxn06071	0,000391198	0,000391198
rxn06078	0	0
rxn06081	0	0
rxn06091	0	0
rxn06108	-0,57630639	-0,000530661
rxn06109	-0,288418526	-0,000530661
rxn06139	0	0
rxn06182	0	0
rxn06195	0	0
rxn06196	0	0
rxn06197	0	0
rxn06209	0	0
rxn06219	0	0
rxn06231	0	0
rxn06244	0	0
rxn06280	0	0
rxn06285	0	0
rxn06298	0	0
rxn06300	0	0
rxn06376	0	0
rxn06403	0	0
rxn06432	0	0
rxn06434	0	0

rxn06435	0	0
rxn06438	0	0
rxn06439	0	0
rxn06440	0	0
rxn06441	0	0
rxn06443	0	0
rxn06444	0	0
rxn06445	0	0
rxn06446	0	0
rxn06447	0	0
rxn06448	0	0
rxn06449	0	0
rxn06485	0	0
rxn06538	0	0
rxn06565	0	0
rxn06584	0	0
rxn06591	0,001564791	0,001564791
rxn06595	0	0
rxn06624	0	0
rxn06648	0	0
rxn06709	0	0
rxn06737	0	0
rxn06751	0	0
rxn06768	0	0
rxn06798	0	0
rxn06799	0	0
rxn06823	0	0
rxn06831	0	0
rxn06836	0	0
rxn06837	-0,864775033	0
rxn06838	0	0
rxn06850	0	0
rxn06882	0	0
rxn06936	0	0
rxn06937	0,001564791	0,001564791
rxn06947	0	0
rxn06958	-0,000391198	-0,000391198
rxn07056	0	0
rxn07059	0	0
rxn07099	0	0
rxn07251	0	0
rxn07292	0	0
rxn07437	0	0
rxn07438	0	0
rxn07465	0,005915331	0,005915333
rxn07466	-1000	1000
rxn07489	0	0
rxn07573	0	0
rxn07577	0	0
rxn07578	0	0

rxn07587	0	0
rxn07846	0	0
rxn07987	0	0
rxn07989	0	0
rxn07991	0	0
rxn07992	0	0
rxn07993	0	0
rxn07994	0	0
rxn08035	0	0
rxn08038	0	0
rxn08040	0	0
rxn08067	-0,25919929	0
rxn08133	0	0
rxn08294	0	0
rxn08295	0	0
rxn08296	0	0
rxn08297	0	0
rxn08298	0	0
rxn08299	0	0
rxn08300	0	0
rxn08306	0	0
rxn08307	0	0
rxn08308	0	0
rxn08309	0	0
rxn08310	0	0
rxn08311	0	0
rxn08312	0	0
rxn08448	0	0
rxn08449	0	0
rxn08451	0	0
rxn08453	0	0
rxn08454	0	473,6053188
rxn08455	0	0
rxn08456	0	0
rxn08457	0	0
rxn08519	0,044224643	0,044224656
rxn08546	0	0
rxn08547	0	0
rxn08548	0	0
rxn08549	0	0
rxn08550	0	0
rxn08551	0	0
rxn08552	0	0
rxn08571	0	947,2106375
rxn08582	0	0
rxn08605	0	0
rxn08615	0,307576161	474,1720941
rxn08796	0	0
rxn08797	0	473,6053188
rxn08798	0	0

rxn08799	0	473,6053188
rxn08800	0	0
rxn08801	0	473,6053188
rxn08802	0	0
rxn08803	0	0
rxn08804	0	0
rxn08805	0	0
rxn08806	0	0
rxn08807	0	0
rxn08808	0	0
rxn08809	0	0
rxn08810	0	0
rxn08811	0	0
rxn08812	0	0
rxn08813	0	0
rxn08814	0	0
rxn08815	0	0
rxn08816	0	0
rxn08817	0	0
rxn08818	0	0
rxn08819	0	0
rxn08820	0	0
rxn08821	0	0
rxn08822	0	0
rxn08823	0	0
rxn08838	0	0
rxn08839	0	0
rxn08840	0	0
rxn08841	0	0
rxn08842	0	0
rxn08843	0	0
rxn08844	0	0
rxn08845	0	0
rxn08846	0	0
rxn08847	0	0
rxn08848	0	0
rxn08849	0	0
rxn08850	0	0
rxn08851	0	0
rxn08857	0	0
rxn08889	0,000590305	0,000590305
rxn08890	0,004778577	0,004778578
rxn08891	0,000590305	0,000590305
rxn08892	-999,9887912	1000
rxn08893	-999,9941005	999,9946908
rxn08894	-999,9887912	1000
rxn08897	-0,005309239	-0,005309238
rxn08926	0,000530661	0,000530661
rxn08927	-999,9988203	999,989971
rxn08928	-999,9887912	1000

rxn08929	0,001179733	0,001179733
rxn08930	0	0
rxn08958	0,000590305	0,000590305
rxn09016	0,022463171	0,267331191
rxn09108	0	0
rxn09109	0	0
rxn09110	0	0
rxn09111	0	0
rxn09112	0	0
rxn09113	0	0
rxn09114	0	0
rxn09177	0	0,000505224
rxn09235	0,021761471	0,021761478
rxn09237	0,022463171	0,022463178
rxn09264	0	0
rxn09265	0	0
rxn09398	-474,1720941	-0,307576161
rxn09399	0	0
rxn09445	0	0
rxn09446	0	0
rxn09447	0	0
rxn09486	0	0
rxn09557	0,000195599	0,000195599
rxn09616	0,000530661	0,000530661
rxn09631	0,000195599	0,000195599
rxn09633	0,000195599	0,000195599
rxn09888	0	0
rxn09949	0	0
rxn09978	0	0
rxn09979	0	0
rxn10003	0	0,000505224
rxn10019	0	0
rxn10020	0	0
rxn10021	0	0
rxn10052	52,78936247	1000
rxn10054	0	0,088638023
rxn10056	0	0,000392075
rxn10058	0	0,000392075
rxn10060	0	0,000392075
rxn10192	0	0
rxn10193	0	0
rxn10194	0	0
rxn10196	0	0
rxn10202	0	473,6053188
rxn10203	0	473,6053188
rxn10204	0	473,6053188
rxn10205	0	0
rxn10206	0	0
rxn10207	0	0
rxn10208	0	0

rxn10209	0	0
rxn10210	0	0
rxn10220	0	0
rxn10221	0	0
rxn10222	0	0
rxn10223	0	0
rxn10224	0	0
rxn10225	0	0
rxn10253	0	0
rxn10254	0	0
rxn10255	0	0
rxn10256	0	0
rxn10257	0	0
rxn10258	0	0
rxn10259	0	0
rxn10260	0	0
rxn10261	0	0
rxn10262	0	0
rxn10263	0	0
rxn10264	0	0
rxn10289	0	0
rxn10290	0	0
rxn10291	0	0
rxn10292	0	0
rxn10293	0	0
rxn10294	0	0
rxn10295	0	0
rxn10296	0	0
rxn10297	0	0
rxn10298	0	0
rxn10299	0	0
rxn10300	0	0
rxn10301	0	0
rxn10302	0	0
rxn10303	0	0
rxn10304	0	0
rxn10305	0	0
rxn10306	0	0
rxn10404	0	0
rxn10405	0	0
rxn10406	0	0
rxn10407	0	0
rxn10408	0	0
rxn10409	0	0
rxn10410	0	0
rxn10476	0	0
rxn10951	0,021761471	0,021761478
rxn11007	0,021761471	0,021761478
rxn11513	0	0
rxn11547	0	0

rxn11548	0	0
rxn11550	0	0
rxn11567	0	0
rxn11571	-0,160558929	0
rxn11599	0	0
rxn11641	0	0
rxn11702	0	0
rxn11728	0	0
rxn11749	0	0
rxn11757	0	0
rxn11760	0	0
rxn11761	0	0
rxn11765	0	0
rxn11766	0	0
rxn11772	0	0
rxn11773	0	0
rxn11788	0	0
rxn11878	0	0
rxn11879	0	0
rxn11934	0	0
rxn11951	0	0
rxn11987	0	0,160558929
rxn12008	-4,82419E-05	-4,82419E-05
rxn12033	0	0
rxn12218	-0,000195599	-0,000195599
rxn12221	0,000195599	0,000195599
rxn12510	0,000505224	0,000505224
rxn12649	-999,9992176	0
rxn12844	0	0
rxn12845	0	0
rxn12846	0	0
rxn12847	0	0
rxn13420	0,000530661	0,288418526
rxn13421	0,000530661	0,288418526
rxn13477	4,82419E-05	4,82419E-05
rxn13936	0,011799086	0,011799089
rxn13974	-0,779396334	0
rxn13994	0	0
rxn13996	0	0
rxn14012	4,82419E-05	4,82419E-05
rxn14057	-0,1	0
rxn14120	-1000	-0,000782395
rxn14132	0	0
rxn14147	0	0,864775033
rxn14180	0	0
rxn14198	0	0,864775033
rxn14219	0	0
rxn14238	0	0
rxn14270	0	0
rxn14297	0	0,864775033

rxn14346	0	0
rxn90002	-0,497609834	0
rxn90003	0	0
rxn90005	-0,022153553	-0,021761471
rxn08173	0	0,287887865
Biomass_Bacteria	0,877125	0,877125275
t_Cl	0,003957588	0,003957589
t_Sulfate	0,00329799	0,003297991
t_Cu2+	0,002638392	0,002638393
t_Mg	0,006595103	0,006595105
t_Ca2+	0,003957588	0,003957589
t_NH3	0	0
t_H2O	-7,100717839	-5,016694196
t_Biomass	-0,877125275	-0,877125
t_D-Lactate	0	0
t_Formate	-0,958543564	0
t_H2	0	0,481676787
t_L-Lactate	-0,57577573	0
t_Phosphate	1,371443705	1,545212299
t_Propionate	-0,000195599	-0,000195599
t_O2	0	0
t_D-Glucose	0	0
t_CO2	-0,958543564	0
t_Acetate	-1,330272804	-0,225519363
t_Succinate	-0,287887865	0
t_(S,S)-2,3-Butanediol	0	0
t_H2S	-0,521439422	0
Ex_Cl	-0,003957589	-0,003957588
Ex_Sulfate	-0,003297991	-0,00329799
Ex_Cu2+	-0,002638393	-0,002638392
Ex_Mg	-0,006595105	-0,006595103
Ex_Ca2+	-0,003957589	-0,003957588
Ex_NH3	0	0
Ex_H2O	5,016694196	7,100717839
Ex_Biomass	0,877125	0,877125275
Ex_D-Lactate	0	0
Ex_Formate	0	0,958543564
Ex_H2	-0,481676787	0
Ex_L-Lactate	0	0,57577573
Ex_Phosphate	-1,545212299	-1,371443705
Ex_Propionate	0,000195599	0,000195599
Ex_O2	0	0
Ex_D-Glucose	0	0
Ex_CO2	0	0,958543564
Ex_Acetate	0,225519363	1,330272804
Ex_Succinate	0	0,287887865
Ex_(S,S)-2,3-Butanediol	0	0
Ex_H2S	0	0,521439422
t_Fe2	0,006131104	0,006131106
t_fe3	0,005935505	0,005935507

t_Acetaldehyde	0	0
t_Adenosine	0	0,173768165
t_AMP	0	0,173768165
t_Choline	0	0
t_Cytidine	0	0
t_Cytosine	0	0
t_DAlanine	0	0
t_Deoxyadenosine	0	0,044224656
t_Deoxycytidine	0	0,044224656
t_Deoxyguanosine	0	0
t_Deoxyinosine	0	0
t_Deoxyuridine	0	0
t_DRibose	0,25513198	0,5
t_DSerine	0	0
t_Glycerol	0	0
t_GSH	0	0
t_Guanine	0	0
t_H2S2O3	0	0
t_Heme	0,000195599	0,000195599
t_Homocysteine	0	0
t_HYXN	0	0,205870073
t_Inosine	0	0,205870073
t_LACT	0	0
t_LAlanine	-0,326775863	0,5
t_LArginine	0,287183005	0,287183095
t_LAsparagine	-0,059023652	0,205422739
t_LAspartate	0,235553673	0,5
t_LCysteine	-0,021439422	0,5
t_LGlutamate	-0,02559905	0,063038993
t_LGlutamine	0,411361977	0,5
t_LHistidine	0,080783213	0,080783238
t_LInositol	0	0
t_LIsoleucine	0,247612387	0,247612465
t_LLeucine	0,384005325	0,384005445
t_LLysine	0,085739509	0,292433567
t_LMethionine	0,131541559	0,1315416
t_LPhenylalanine	0,1578825	0,157882549
t_LThreonine	0,216211313	0,21621138
t_LTryptophan	0,048443614	0,048443629
t_LTyrosine	0,11753475	0,117534787
t_LValine	0,361287788	0,361287901
t_Maltose	0	0
t_Niacin	0,001998968	0,001998969
t_Ornithine	0	0
t_PPi	0	0
t_Pyridoxol	0	0
t_XAN	0	0
t_5Deoxyadenosine	0	0
t_Acetoacetate	-0,287887865	0
t_Calomide	0	0

t_CysGly	0	0
t_Glycine	0,499999844	0,5
t_Glycolaldehyde	0	0
t_LProline	0,18840645	0,188406509
t_Methanol	0	0
t_NAcetylDglucosamine	0	0
t_PM	0	0
t_Putrescine	0	0
t_Pyridoxal	0,000195599	0,000195599
t_Riboflavin	0,000391198	0,000391198
t_Sorbitol	0	0
t_Spermidine	0	0
t_Sucrose	0	0
t_Taurine	0	0
t_Thiamin	0	0
t_Thymidine	0	0
t_Uracil	0	0,24486802
t_Uridine	0	0,280960768
t_Mn2+	0,002638392	0,002638393
t_Fumarate	-0,479271782	0
t_Adenine	0	0
t_Nicotinamide	0	0
t_Co2+	0,002638392	0,002638393
t_D-Glutamate	0	0
t_Chorismate	0	0
t_Folate	0,000782395	0,000782396
t_N-Acetyl-D-mannosamine	0	0
t_Siroheme	0	0
t_Menaquinone 7	0	0
t_2-Demethylmenaquinone 8	0	0
t_Menaquinone 8	0	0
t_Ubiquinone-8	0	0
t_2-Oxobutyrate	0	0
t_Neu5Ac	0	0
t_Glycerol-3-phosphate	0	0
t_H+	-2,959100131	0,5
t_Nicotinamide ribonucleotide	0	0
t_PAN	0,000505224	0,000505224
t_Pyridoxal phosphate	0	0
t_Zn2+	0,002638392	0,002638393
t_1,2-Diacyl-sn-glycerol dioctadecanoyl	0	0
t_meso-2,6-Diaminopimelate	0	0
t_L-Serine	0,206868177	0,5
t_D-Fructose	0,048572551	0,048572566
t_Cholesterol	0	0
t_beta D-Galactose	0	0
Ex_Fe2	-0,006131106	-0,006131104
Ex_fe3	-0,005935507	-0,005935505
Ex_Acetaldehyde	0	0
Ex_Adenosine	-0,173768165	0

Ex_AMP	-0,173768165	0
Ex_Choline	0	0
Ex_Cytidine	0	0
Ex_Cytosine	0	0
Ex_DAlanine	0	0
Ex_Deoxyadenosine	-0,044224656	0
Ex_Deoxycytidine	-0,044224656	0
Ex_Deoxyguanosine	0	0
Ex_Deoxyinosine	0	0
Ex_Deoxyuridine	0	0
Ex_DRibose	-0,5	-0,25513198
Ex_DSerine	0	0
Ex_Glycerol	0	0
Ex_GSH	0	0
Ex_Guanine	0	0
Ex_Heme	-0,000195599	-0,000195599
Ex_Homocysteine	0	0
Ex_HYXN	-0,205870073	0
Ex_Inosine	-0,205870073	0
Ex_LACT	0	0
Ex_LAlanine	-0,5	0,326775863
Ex_LArginine	-0,287183095	-0,287183005
Ex_LAsparagine	-0,205422739	0,059023652
Ex_LAspartate	-0,5	-0,235553673
Ex_LCysteine	-0,5	0,021439422
Ex_LGlutamate	-0,063038993	0,02559905
Ex_LGlutamine	-0,5	-0,411361977
Ex_LHistidine	-0,080783238	-0,080783213
Ex_LInositol	0	0
Ex_LIsoleucine	-0,247612465	-0,247612388
Ex_LLeucine	-0,384005445	-0,384005325
Ex_LLysine	-0,292433567	-0,085739509
Ex_LMethionine	-0,1315416	-0,131541559
Ex_LPhenylalanine	-0,157882549	-0,1578825
Ex_LThreonine	-0,21621138	-0,216211313
Ex_LTryptophan	-0,048443629	-0,048443614
Ex_LTyrosine	-0,117534787	-0,11753475
Ex_LValine	-0,361287901	-0,361287788
Ex_Maltose	0	0
Ex_Niacin	-0,001998969	-0,001998968
Ex_Ornithine	0	0
Ex_PPi	0	0
Ex_XAN	0	0
Ex_5Deoxyadenosine	0	0
Ex_Acetoacetate	0	0,287887865
Ex_Calomide	0	0
Ex_CysGly	0	0
Ex_Glycine	-0,5	-0,499999844
Ex_Glycolaldehyde	0	0
Ex_LProline	-0,188406509	-0,18840645

Ex_Methanol	0	0
Ex_NAcetylDglucosamine	0	0
Ex_PM	0	0
Ex_Putrescine	0	0
Ex_Pyridoxal	-0,000195599	-0,000195599
Ex_Riboflavin	-0,000391198	-0,000391198
Ex_Sorbitol	0	0
Ex_Spermidine	0	0
Ex_Sucrose	0	0
Ex_Taurine	0	0
Ex_Thiamin	0	0
Ex_Thymidine	0	0
Ex_Uracil	-0,24486802	0
Ex_Uridine	-0,280960768	0
Ex_Mn2+	-0,002638393	-0,002638392
Ex_Fumarate	0	0,479271782
Ex_Adenine	0	0
Ex_Nicotinamide	0	0
Ex_Co2+	-0,002638393	-0,002638392
Ex_D-Glutamate	0	0
Ex_Folate	-0,000782396	-0,000782395
Ex_N-Acetyl-D-mannosamine	0	0
Ex_Siroheme	0	0
Ex_Menaquinone 7	0	0
Ex_2-Demethylmenaquinone 8	0	0
Ex_Menaquinone 8	0	0
Ex_Ubiquinone-8	0	0
Ex_Neu5Ac	0	0
Ex_H+	-0,5	2,959100131
Ex_Nicotinamide ribonucleotide	0	0
Ex_PAN	-0,000505224	-0,000505224
Ex_Zn2+	-0,002638393	-0,002638392
Ex_1,2-Diacyl-sn-glycerol dioctadecanoyl	0	0
Ex_L-Serine	-0,5	-0,206868177
Ex_D-Fructose	-0,048572566	-0,048572551
Ex_Cholesterol	0	0
Ex_beta D-Galactose	0	0
t_octanoate	0	0
t_Amylose	0	0
Ex_Amylose	0	0
t_Isovaleric_acid	0	0
t_H2O2	0	0
Ex_Isovaleric_acid	0	0
Ex_H2O2	0	0
rxn01207_1	0	0
rxn08972	0	0
rxn08973	0	0
rxn06111	0	0,57577573
rxn13726	0	0
rxn13727	0	0

rxn13729	0	0
rxn08974	0	0
rxn10122	0	0
rxn10123	0	0
rxn10124	0	0
rxn12665	0	0
t_Sulfite	0	0,1
Ex_Sulfite	-0,1	0

rxn ID	minFlux	max Flux
rxn00001	0	1000
rxn00003	0	0
rxn00011	0	0
rxn00016	0	0
rxn00022	0	0,505
rxn00029	0,00101873	0,00101873
rxn00044	0	0
rxn00048	0	0,000509365
rxn00060	0,000254683	0,000254683
rxn00062	0	1000
rxn00065	0	0
rxn00066	0	499,9957058
rxn00067	0	0
rxn00077	0	0,000510507
rxn00085	-1000	0
rxn00097	-1000	1000
rxn00100	0,000657835	0,000657835
rxn00102	-1000	0,694385566
rxn00103	0	1000
rxn00104	-1000	0
rxn00105	-999,9973972	0
rxn00106	-1000	0
rxn00107	0	0
rxn00109	0	0
rxn00117	-999,5995158	1000
rxn00119	0,368989262	1000
rxn00122	0,000254683	0,000254683
rxn00124	0,000254683	0,000254683
rxn00126	0,000764048	0,000764048
rxn00131	-1000	999,9966332
rxn00132	0	1000
rxn00133	0	0
rxn00137	0	0
rxn00138	0	1000
rxn00139	-999,9971425	0
rxn00142	0	0
rxn00143	0,000509365	0,000509365
rxn00144	0	0
rxn00147	0	1000
rxn00148	-1000	0
rxn00151	-1000	0
rxn00154	0	508,1381613
rxn00157	-508,1381613	0
rxn00159	-1000	1000
rxn00161	-1000	1000
rxn00162	0	1000
rxn00165	0	500,1552553
rxn00171	0	1,303068753
rxn00173	0	0

rxn00175	0	1000
rxn00176	0	0
rxn00179	0	0
rxn00182	-1000	0
rxn00184	-1000	0
rxn00187	0	1000
rxn00189	0	1000
rxn00190	0	1000
rxn00192	0	1000
rxn00193	0,031494975	0,031494975
rxn00196	0	0
rxn00198	0	1000
rxn00199	0	1000
rxn00200	0	0
rxn00202	0	0
rxn00205	0	0
rxn00206	0,008588396	301,4167919
rxn00213	0	0
rxn00214	0	0
rxn00216	0	2,03
rxn00226	0	0
rxn00231	0	0
rxn00237	-1000	1000
rxn00238	-1000	0
rxn00239	0,238807673	1000
rxn00242	0	1000
rxn00250	-0,69442097	999,9999646
rxn00254	0	0
rxn00256	-506,9653518	0
rxn00259	0	0
rxn00260	-1000	0,69442097
rxn00262	0	301,4082035
rxn00272	-1000	1000
rxn00275	-1,747095722	999,8630619
rxn00279	0	1000
rxn00283	0,027731841	0,027731841
rxn00290	-1000	1000
rxn00293	0,062989949	999,6940007
rxn00299	0	0
rxn00300	0	0,000509365
rxn00301	0	999,7611923
rxn00302	0	0
rxn00303	0	1000
rxn00304	-1000	0
rxn00307	0	0
rxn00313	0	0,805078813
rxn00322	0	0
rxn00324	-301,4082035	0
rxn00328	0	0
rxn00333	0	301,4082035

rxn00337	0,039197122	0,844275935
rxn00338	0	0,002602787
rxn00340	0	1000
rxn00342	0	1000
rxn00346	0	0
rxn00347	0	1000
rxn00348	0	0
rxn00350	-0,000254683	-0,000254683
rxn00360	0	0
rxn00363	0	1000
rxn00364	-999,6310107	1000
rxn00365	0	1000
rxn00368	0	1000
rxn00369	0	1000
rxn00371	0	1000
rxn00377	0	0
rxn00383	0,004294198	0,004294198
rxn00388	0	0
rxn00391	0	999,9997453
rxn00392	0,000254683	1000
rxn00405	0	0
rxn00409	-1000	1000
rxn00410	-999,8225103	999,8085005
rxn00411	-1000	0
rxn00412	0	1000
rxn00414	0	0,402539407
rxn00416	0	1000
rxn00420	0	0
rxn00422	-1000	1000
rxn00423	0	500,1552553
rxn00424	-1000	1000
rxn00426	0	0
rxn00433	0	0
rxn00436	0	999,9997453
rxn00437	0	0
rxn00440	0,000254683	1000
rxn00453	0	999,9997453
rxn00456	0	999,9997453
rxn00459	-1,30360447	7,300215853
rxn00460	-1000	0
rxn00461	0,031494975	0,031494975
rxn00469	0	1000
rxn00470	0,046135221	1000
rxn00474	0	0
rxn00490	0	1,610157626
rxn00493	-1,610157626	0
rxn00499	-8,786223562	0
rxn00505	0	1000
rxn00506	0	1,303068753
rxn00508	0	999,9538648

rxn00509	0	999,9538648
rxn00514	0	0
rxn00515	-1000	1000
rxn00517	-1000	0
rxn00527	-1,610157626	0
rxn00533	-999,9999646	3,54043E-05
rxn00539	0	0
rxn00541	-1,303068753	0
rxn00543	-1,303068753	0
rxn00545	0	1000
rxn00546	0	0
rxn00547	0	1
rxn00549	0	1000
rxn00551	0	1000
rxn00554	0	1000
rxn00555	0,062989949	0,06298995
rxn00556	0	1000
rxn00557	0	1000
rxn00558	-1000	1000
rxn00559	0	0
rxn00565	0	0
rxn00566	0	500,2643673
rxn00575	0	0,5
rxn00585	0	0
rxn00611	-0,401371631	0
rxn00615	0	0,401371631
rxn00616	0	0,401371631
rxn00622	0	0
rxn00623	-251,3766277	0
rxn00624	0	0
rxn00642	0	0
rxn00645	0,004294198	251,3809219
rxn00647	0	0
rxn00649	0	500,1552553
rxn00650	-0,000254683	-0,000254683
rxn00653	0	0
rxn00657	0	0
rxn00670	0	0
rxn00674	0	0
rxn00675	0	0
rxn00689	0	0
rxn00690	0	1000
rxn00692	-0,260507082	499,8947482
rxn00693	0,000509365	0,393414802
rxn00695	-1000	1000
rxn00704	0	0
rxn00707	0	1000
rxn00708	0	1000
rxn00709	0	1000
rxn00710	0	0

rxn00711	-999,9971425	0
rxn00712	0	1000
rxn00713	0	1000
rxn00714	0	0
rxn00715	0	1000
rxn00719	0	0
rxn00726	0	0
rxn00727	0	0
rxn00729	0	0
rxn00735	0	0
rxn00737	0,000254683	0,393160119
rxn00741	0	0
rxn00742	-1000	-0,000254683
rxn00743	0	0,401371631
rxn00747	-3,336572636	0,8134214
rxn00748	0	0
rxn00758	0	0
rxn00762	-0,401371631	0
rxn00763	0	0
rxn00765	0	0
rxn00770	0,002857469	1000
rxn00772	0,000509365	0,500509365
rxn00775	0	0
rxn00777	-1,073663777	0,370156845
rxn00778	-0,308245774	1000
rxn00779	0	1000
rxn00781	-1000	7,300215853
rxn00784	0	1,303068753
rxn00785	-0,184653952	1,342674749
rxn00786	-1000	3,336572636
rxn00787	0	0
rxn00789	0	0
rxn00790	-0,000254683	-0,000254683
rxn00792	0	0
rxn00796	0	0
rxn00799	-1000	4,59536395
rxn00800	-0,268565555	815,3147588
rxn00802	0	0,402539407
rxn00816	0	0
rxn00817	0	0
rxn00818	0	0
rxn00819	0	0
rxn00829	0,000690955	0,000690955
rxn00830	6,28141E-05	6,28141E-05
rxn00831	0	999,9971425
rxn00832	0	0
rxn00834	-999,7290863	1000
rxn00836	-999,9971425	0
rxn00838	-0,268565555	815,3147588
rxn00851	0	1000

rxn00855	0	0
rxn00856	0,008138419	999,9620032
rxn00867	0	0
rxn00869	0	0
rxn00871	0	1000
rxn00875	0	1000
rxn00877	0	0
rxn00881	0	0
rxn00882	0	0
rxn00883	0	0
rxn00889	0	0
rxn00890	0	0
rxn00898	0	0
rxn00902	0	0
rxn00907	-999,9994906	0,000509365
rxn00908	-1000	1000
rxn00909	-0,007702147	0,393669484
rxn00910	-0,401371631	0
rxn00913	0,029248515	1000
rxn00915	-999,9707515	0
rxn00916	-999,7319438	1000
rxn00917	0	1000
rxn00918	0	0
rxn00921	0	0
rxn00926	0	815,582815
rxn00929	-1000	1000
rxn00931	-1000	1000
rxn00938	0	999,9971425
rxn00952	0	0,392905437
rxn00955	0,000509365	0,000509365
rxn00973	-1000	1000
rxn00974	-1000	1000
rxn00977	0	0
rxn00980	0	0
rxn00983	0	0
rxn00986	0	0
rxn00990	-1000	1000
rxn00991	-0,000690955	-0,000690955
rxn00992	0	0
rxn00994	-1000	0
rxn00999	0	0
rxn01000	0	1,610157626
rxn01011	0	0
rxn01013	0	0
rxn01016	0	0
rxn01018	0	0
rxn01019	0	0,402539407
rxn01021	0	0
rxn01027	0	0
rxn01037	0	0

rxn01042	0	0
rxn01053	0	0
rxn01069	0	0
rxn01073	0	0
rxn01080	0	0
rxn01100	-1000	0
rxn01101	0	0
rxn01106	-7,300215853	1,30360447
rxn01116	-1,074682507	0,369817268
rxn01123	0	0
rxn01133	0	0
rxn01138	0	1000
rxn01139	0	0
rxn01146	0	0
rxn01169	0	0
rxn01199	0	0
rxn01200	0	1000
rxn01201	-6,590358626	-0,000690955
rxn01202	0	0
rxn01203	0	0
rxn01204	0,000690955	999,9545557
rxn01210	0	0
rxn01211	-999,9997453	0,000509365
rxn01213	6,28141E-05	6,28141E-05
rxn01225	0	999,9707515
rxn01228	0	0
rxn01233	0	0
rxn01236	-1000	0
rxn01237	0	0
rxn01241	-1000	1000
rxn01242	0	0
rxn01249	0	0
rxn01253	0	0
rxn01255	0,000254683	1,610412309
rxn01256	0	1,610157626
rxn01257	0	0
rxn01265	-999,9997453	0
rxn01268	0	1,610157626
rxn01270	-1,610157626	0
rxn01274	0	0
rxn01276	0	0
rxn01277	0	0
rxn01278	0	0
rxn01286	0	0
rxn01297	-999,7319438	999,9971425
rxn01300	0	0
rxn01303	0	0
rxn01304	0	0
rxn01321	0	0
rxn01332	0,000254683	1,610412309

rxn01333	-1000	0,599515007
rxn01334	0	1000
rxn01343	0	1000
rxn01346	0	1000
rxn01347	0	1000
rxn01348	0	1000
rxn01351	0	1000
rxn01352	-1000	-0,029248515
rxn01353	-1000	1000
rxn01354	-1000	0
rxn01358	0	999,9971425
rxn01361	0	0
rxn01362	0	0
rxn01366	-0,33749429	999,9707515
rxn01367	0	0
rxn01368	0	999,6310107
rxn01370	0	1000
rxn01377	0	0
rxn01380	0	0
rxn01387	-1000	0
rxn01388	-1000	1000
rxn01390	0	0
rxn01396	0	0
rxn01423	0	0
rxn01426	0	0
rxn01434	0	0,402539407
rxn01437	0	0
rxn01445	0	999,9707515
rxn01446	-0,029248515	-0,029248515
rxn01459	0	999,9543011
rxn01465	0	0
rxn01466	6,28141E-05	6,28141E-05
rxn01476	0	0
rxn01478	0	0
rxn01484	0	0
rxn01485	-0,06298995	-0,062989949
rxn01486	0	0
rxn01492	0	0
rxn01500	-0,000690955	-0,000690955
rxn01504	0	0
rxn01509	-999,9707515	1000
rxn01510	0	1000
rxn01513	0,028334856	0,028334856
rxn01517	0	1000
rxn01518	0,028334856	1000
rxn01519	0	1000
rxn01521	0	999,9716651
rxn01522	0	0
rxn01530	0	0
rxn01537	0	999,9997453

rxn01539	-1000	-0,000254683
rxn01544	-999,9971425	0
rxn01548	0,029248515	1000
rxn01549	0	0
rxn01603	0	0
rxn01610	0	0
rxn01626	0	0
rxn01629	-0,00203746	-0,00203746
rxn01636	-999,9538648	1000
rxn01637	-1000	-0,046135221
rxn01641	0	0
rxn01642	0	0
rxn01643	-0,844275935	-0,039197122
rxn01644	0,031494975	0,836573788
rxn01645	-0,007702147	-0,007702147
rxn01646	0	999,9971425
rxn01647	0	999,9971425
rxn01648	0	0
rxn01649	0	999,9971425
rxn01650	0	0
rxn01652	0	1000
rxn01653	0	1000
rxn01654	0	0
rxn01667	-1000	0
rxn01669	0	999,9973972
rxn01670	0	999,9971425
rxn01678	0	1000
rxn01679	0	1000
rxn01682	0	0
rxn01684	0	0
rxn01704	0	0
rxn01706	0	1000
rxn01729	0	0
rxn01735	0	0
rxn01737	0	0
rxn01739	0,000254683	1,610412309
rxn01741	-1,610412309	-0,000254683
rxn01757	0	0
rxn01758	-251,3809219	-0,004294198
rxn01775	0	0
rxn01790	0	0
rxn01791	0	0
rxn01799	-0,028334856	0,33749429
rxn01800	0	0,365829146
rxn01807	0	0
rxn01827	0	0
rxn01831	0	0
rxn01834	0	0
rxn01843	0	0
rxn01851	0	999,9543011

rxn01859	0	0,494822979
rxn01870	0	0
rxn01885	0	0
rxn01906	0	0
rxn01917	0,046135221	1000
rxn01937	0	0
rxn01961	0	999,9971425
rxn01962	0	0
rxn01964	0	0
rxn01967	0	0
rxn01972	0,031494975	1000
rxn01973	-999,968505	0
rxn01974	0,031494975	0,836573788
rxn01977	-1000	1000
rxn01982	0	0
rxn01985	0	0
rxn01986	-0,057583371	0,803068753
rxn01987	-0,5	0
rxn01990	0	0
rxn01997	0	0
rxn02000	0	0
rxn02008	0,031494975	0,031494975
rxn02011	0,031494975	0,031494975
rxn02012	0	0
rxn02015	0	0
rxn02020	0	0
rxn02046	0	0
rxn02056	0	999,9997453
rxn02085	0	0
rxn02093	0	0
rxn02106	0	0
rxn02118	0	0
rxn02122	0	0
rxn02128	0	0
rxn02138	0	0
rxn02155	0,002602787	1000
rxn02160	0	0
rxn02161	0	0
rxn02166	0	0
rxn02167	0	0
rxn02171	0,000690955	6,590358626
rxn02175	0,000657835	0,000657835
rxn02185	0	0
rxn02186	0	0
rxn02187	0	0
rxn02190	0	0
rxn02195	0	0
rxn02200	0	0
rxn02201	0	0
rxn02212	0,000254683	1,610412309

rxn02213	0	0
rxn02219	0	0
rxn02222	0	0
rxn02228	0	0
rxn02236	0	0
rxn02264	0,000254683	0,000254683
rxn02275	0	0
rxn02283	0	0
rxn02284	-0,031494975	0
rxn02285	-0,031494975	0
rxn02286	0,031494975	0,031494975
rxn02287	-999,9997453	1000
rxn02288	0	0
rxn02302	-1000	-0,000254683
rxn02303	0	0
rxn02305	0,000254683	1000
rxn02313	0	0
rxn02314	0	1000
rxn02315	0	1000
rxn02316	0	1000
rxn02317	-1000	0
rxn02320	0	0
rxn02322	0,000690955	0,000690955
rxn02339	0	0
rxn02341	0,000657835	0,000657835
rxn02342	0	0
rxn02346	0	0
rxn02350	0	0
rxn02351	0	0
rxn02356	-1000	1000
rxn02358	-1000	1000
rxn02373	-1000	1000
rxn02375	0	0
rxn02380	-1000	1000
rxn02400	0	999,9971425
rxn02402	-0,002602787	0
rxn02409	0	0
rxn02415	0	0
rxn02449	0	1000
rxn02454	0	0
rxn02465	-1000	-0,046135221
rxn02473	0	0
rxn02474	-0,000509365	0
rxn02475	0	0,000509365
rxn02476	0,000254683	1,610412309
rxn02483	0	0
rxn02484	0	999,9997453
rxn02495	0	0
rxn02503	0	0
rxn02504	0	0

rxn02507	0	0
rxn02517	0	0
rxn02518	0	0
rxn02521	0	0
rxn02522	0	0
rxn02525	0	0
rxn02569	0	0
rxn02571	0	0
rxn02581	0	0
rxn02596	0	0
rxn02597	0	0
rxn02625	0	0
rxn02650	0	0
rxn02727	0	0
rxn02729	0	0
rxn02749	0	0
rxn02751	0	0
rxn02760	0	0
rxn02762	0	0
rxn02774	-999,9997453	0
rxn02775	0	0
rxn02776	0	0
rxn02789	0	0
rxn02791	0	0
rxn02792	0	0
rxn02795	0	0
rxn02796	0	0
rxn02811	0	0
rxn02822	0	0
rxn02832	0	0
rxn02834	0	0
rxn02835	0	0
rxn02853	0	0
rxn02875	0	0
rxn02895	0,000254683	0,000254683
rxn02897	0	0
rxn02900	0	0
rxn02922	0	0
rxn02928	-1000	999,968505
rxn02929	-1000	999,968505
rxn02931	0	0
rxn02936	0	0
rxn02937	0,000254683	0,000254683
rxn02939	0	0
rxn02986	0	0
rxn02988	-0,002602787	0
rxn02990	0	0
rxn03004	0	0,000254683
rxn03005	-0,000254683	0
rxn03030	0,031494975	1000

rxn03031	-999,968505	0
rxn03039	0	0
rxn03044	0	0
rxn03047	0	0
rxn03062	0	0
rxn03068	0	0
rxn03075	0	999,9997453
rxn03080	0	0,00101873
rxn03084	0,000254683	0,000254683
rxn03086	-1000	-0,031494975
rxn03087	0	999,968505
rxn03094	0	0
rxn03095	0	0
rxn03102	0	0
rxn03106	0	0
rxn03108	0,000254683	1000
rxn03130	0	0
rxn03135	0	0
rxn03136	0	0
rxn03137	0	0
rxn03140	0	0
rxn03141	0	0
rxn03146	0	0
rxn03147	0	0
rxn03150	0	0
rxn03159	0	0
rxn03164	0,031494975	0,031494975
rxn03167	0	0
rxn03174	0	0
rxn03175	0	0
rxn03194	0	0
rxn03263	0	0
rxn03264	0	0
rxn03273	0	0
rxn03282	0	0
rxn03333	0	0
rxn03354	0	0
rxn03362	0	0
rxn03374	0	0
rxn03384	0	0
rxn03393	0	0
rxn03395	0	0
rxn03397	0	0
rxn03405	0	0
rxn03406	0	0
rxn03407	0	0
rxn03408	0,031494975	0,031494975
rxn03409	0	0
rxn03419	0	0
rxn03421	0	0

rxn03423	0	0
rxn03435	0	0
rxn03436	0	0
rxn03437	0	0
rxn03446	0	0
rxn03462	0	0
rxn03468	0	0
rxn03483	0	0
rxn03492	0	0
rxn03512	0	0
rxn03513	0	0
rxn03514	0	0
rxn03535	0	0
rxn03536	0	0
rxn03537	0	0
rxn03538	0	0
rxn03540	0	0
rxn03548	0	1000
rxn03549	0	0
rxn03552	0	0
rxn03553	0	0
rxn03598	0	0
rxn03638	0,062989949	0,06298995
rxn03641	0,000690955	6,590358626
rxn03642	0,000690955	6,590358626
rxn03838	0	0
rxn03839	0	0
rxn03841	0	0
rxn03852	0	0
rxn03864	0	0
rxn03869	0	0
rxn03870	0	0
rxn03884	0	0
rxn03885	0	0
rxn03886	0	0
rxn03891	0	0
rxn03901	0,031494975	0,031494975
rxn03902	0	0
rxn03903	0	0
rxn03904	0,031494975	0,031494975
rxn03907	0	0
rxn03908	0	0
rxn03909	0	0
rxn03910	0	0
rxn03919	0	0
rxn03933	0	0
rxn03958	0	0
rxn03962	0	0
rxn03964	0	0
rxn03974	-0,028334856	0

rxn03975	-0,028334856	0
rxn03990	0	0
rxn03991	0	0
rxn04016	0	0
rxn04045	0	0
rxn04046	0	0
rxn04047	0	0
rxn04048	0	0
rxn04050	0	0
rxn04051	0	1000
rxn04052	0	0
rxn04068	0	0
rxn04113	0	0
rxn04142	0	0
rxn04234	0	0
rxn04308	0	0
rxn04384	0	0
rxn04385	0	0
rxn04413	0	0
rxn04417	0	0
rxn04418	0	0
rxn04432	0	0
rxn04443	0	0
rxn04482	0	0
rxn04674	0	0
rxn04676	-0,268981651	1000
rxn04678	-1000	0,268981651
rxn04681	0	0
rxn04682	0	0
rxn04703	0	0
rxn04704	0	0
rxn04726	0	0
rxn04736	0	0
rxn04794	0	1000
rxn04822	0	0
rxn04840	0	0
rxn04841	0	0
rxn04865	0	0
rxn04866	0	0
rxn04954	-0,401371631	0
rxn04960	0	0
rxn05005	-1000	0
rxn05006	-1000	0
rxn05010	0	0
rxn05011	0	0
rxn05029	0	0
rxn05030	6,28141E-05	6,28141E-05
rxn05039	0	0,000509365
rxn05040	0	0,00101873
rxn05050	0	0

rxn05054	0	0
rxn05108	0	0
rxn05115	0	0
rxn05124	0	0
rxn05233	0	0
rxn05234	0	0
rxn05236	0	0
rxn05239	0	0
rxn05256	0	0
rxn05269	0	0
rxn05289	0	0
rxn05322	0	0
rxn05323	0	0
rxn05324	0	0
rxn05325	0	0
rxn05326	0	0
rxn05327	0	0
rxn05328	0	0
rxn05329	0	0
rxn05330	0	0
rxn05331	0	0
rxn05332	0	0
rxn05333	0	0
rxn05334	0	0
rxn05335	0	0
rxn05336	0	0
rxn05337	0	0
rxn05338	0	0
rxn05339	0	0
rxn05340	0	0
rxn05341	0	0
rxn05342	0	0
rxn05343	0	0
rxn05344	0	0
rxn05345	0	0
rxn05346	0	0
rxn05347	0	0
rxn05348	0	0
rxn05350	0	0
rxn05351	0	0
rxn05352	0	0
rxn05353	0	0
rxn05354	0	0
rxn05355	0	0
rxn05356	0	0
rxn05357	0	0
rxn05457	0	0
rxn05465	0	0
rxn05733	0	0
rxn05740	-1000	1000

rxn05759	-0,6	0
rxn05760	-1000	1000
rxn05762	0,004294198	500
rxn05778	0	0
rxn05779	0	0
rxn05794	-1000	0
rxn05833	0	0
rxn05853	0	0
rxn05871	0	0
rxn05872	0	0
rxn05874	0	0
rxn05887	0	0,6
rxn05899	0	0
rxn05901	0	0
rxn05918	0	0
rxn05927	0	0
rxn05934	0	0
rxn05937	-1000	1000
rxn05938	-508,1381613	0
rxn05939	-506,886961	1000
rxn05940	-1000	0
rxn05952	0	0
rxn05953	0	0
rxn05958	0	0
rxn05962	0	0
rxn05964	0	0
rxn05970	0	0
rxn05979	0	0
rxn05990	0	0
rxn05994	0	0
rxn05995	0	0
rxn06023	0	0
rxn06025	0	0
rxn06043	0	1000
rxn06044	0	0
rxn06045	0	1000
rxn06071	0,017176793	602,8335838
rxn06075	0	0
rxn06078	0	0
rxn06079	0	1000
rxn06080	0	1000
rxn06081	0	0,401371631
rxn06087	0	0
rxn06090	0	0
rxn06091	0	0
rxn06096	0	0
rxn06108	-1000	0
rxn06109	-4,326883291	506,8868491
rxn06139	0	1000
rxn06140	0	0

rxn06181	0	1000
rxn06182	0	1000
rxn06190	0	0
rxn06195	0	0
rxn06196	0	0
rxn06200	0	0
rxn06201	0	0
rxn06217	0	0
rxn06218	0	0
rxn06219	0	0
rxn06224	0	0
rxn06231	0	0
rxn06280	0	0
rxn06285	0	0
rxn06298	0	0
rxn06299	0	0
rxn06300	0	0
rxn06316	0	0
rxn06328	0	0
rxn06347	0	0
rxn06348	0	0
rxn06362	0	0
rxn06376	0	0
rxn06377	0	1000
rxn06381	0	0
rxn06425	0	0
rxn06432	0	0
rxn06434	0	0
rxn06435	0	0
rxn06437	0	0
rxn06438	0	0
rxn06439	0	0
rxn06440	0	0
rxn06441	0	0
rxn06443	0	0
rxn06444	0	0
rxn06445	0	0
rxn06446	0	0
rxn06447	0	0
rxn06448	0	0
rxn06449	0	0
rxn06485	0	0
rxn06489	0	0
rxn06493	0	1000
rxn06522	0	0
rxn06528	0	0
rxn06538	0	0
rxn06584	0	0
rxn06591	0,00203746	0,00203746
rxn06600	0	1000

rxn06608	0	0
rxn06614	0	0
rxn06624	0	0
rxn06641	0	0
rxn06648	0	0
rxn06664	0	0
rxn06671	0	0
rxn06672	0	1000
rxn06673	0	1000
rxn06699	0	0
rxn06701	0	0
rxn06717	0	0
rxn06726	0	0
rxn06729	0	0
rxn06737	0	0
rxn06751	0	0
rxn06768	0	0
rxn06817	0	0
rxn06831	0	0
rxn06850	0	0
rxn06864	0	0
rxn06874	0	0,1
rxn06882	0	0
rxn06887	0	0
rxn06889	0	1000
rxn06890	0	0
rxn06926	0	0
rxn06936	0	0
rxn06937	0,00203746	0,00203746
rxn06947	0	0
rxn06958	-602,8335838	-0,017176793
rxn06979	0	0
rxn06983	0	0
rxn07056	0	0
rxn07099	0	0
rxn07189	0	0
rxn07193	0	0
rxn07199	0	0
rxn07200	0	0,6
rxn07258	0	0
rxn07267	-0,401371631	0
rxn07292	0	0
rxn07450	0,004294198	251,3809219
rxn07456	0	0,402539407
rxn07465	0,007702147	0,007702147
rxn07466	-1000	1000
rxn07489	0	0
rxn07573	0	0
rxn07577	0	0
rxn07578	0	0

rxn07579	0	0
rxn07584	0	0
rxn07585	0	0
rxn07586	0	0
rxn07587	0	0
rxn07588	-1000	0
rxn07589	-1000	0
rxn07623	0	0
rxn07645	0	0
rxn07679	0	0
rxn07683	0	0
rxn07687	0	0
rxn07849	0	0
rxn07987	0	0
rxn07989	0	0
rxn07991	0	0
rxn07992	0	0
rxn07993	0	0
rxn07994	0	0
rxn08043	0	0
rxn08067	-1000	1000
rxn08083	0	0
rxn08084	0	0
rxn08085	0	0
rxn08086	0	0
rxn08087	0	0
rxn08088	0	0
rxn08089	0	0
rxn08094	0	1000
rxn08126	0	0
rxn08127	0	0
rxn08128	0	0
rxn08129	0	0
rxn08131	0,000254683	0,000254683
rxn08133	0	0
rxn08171	0	0
rxn08180	0	0
rxn08194	-1000	1000
rxn08294	0	1000
rxn08295	0	1000
rxn08296	0	1000
rxn08297	0	1000
rxn08298	0	1000
rxn08299	0	1000
rxn08300	0	1000
rxn08306	0	0
rxn08307	0	0
rxn08308	0	0
rxn08309	0	0
rxn08310	0	0

rxn08311	0	0
rxn08312	0	0
rxn08352	0	0
rxn08386	0	0
rxn08387	0	0
rxn08390	0	0
rxn08391	0	0
rxn08392	0	0
rxn08393	0	0
rxn08394	0	0
rxn08395	0	0
rxn08396	0	0
rxn08397	0	0
rxn08398	0	0
rxn08399	0	0
rxn08413	0	0
rxn08519	0,057583371	0,057583371
rxn08546	0	0
rxn08547	0	0
rxn08548	0	0
rxn08549	0	0
rxn08550	0	0
rxn08551	0	0
rxn08552	0	0
rxn08605	0	0
rxn08607	0	0
rxn08615	-1000	1000
rxn08668	0	0
rxn08669	0	0
rxn08733	0	0
rxn08764	0	0
rxn08796	0	0
rxn08797	0	0
rxn08798	0	0
rxn08799	0	0
rxn08800	0	0
rxn08801	0	0
rxn08802	0	0
rxn08803	0	0
rxn08804	0	0
rxn08805	0	0
rxn08806	0	0
rxn08807	0	0
rxn08808	0	0
rxn08809	0	0
rxn08810	0	0
rxn08811	0	0
rxn08812	0	0
rxn08813	0	0
rxn08814	0	0

rxn08815	0	0
rxn08816	0	0
rxn08817	0	0
rxn08818	0	0
rxn08819	0	0
rxn08820	0	0
rxn08821	0	0
rxn08822	0	0
rxn08823	0	0
rxn08838	0	0
rxn08839	0	0
rxn08840	0	0
rxn08841	0	0
rxn08842	0	0
rxn08843	0	0
rxn08844	0	0
rxn08845	0	0
rxn08846	0	0
rxn08847	0	0
rxn08848	0	0
rxn08849	0	0
rxn08850	0	0
rxn08851	0	0
rxn08857	0	0
rxn08889	0,000768616	0,000768616
rxn08890	0,006222019	0,006222019
rxn08891	0,000768616	0,000768616
rxn08892	-999,9854054	1000
rxn08893	-999,9923184	999,993087
rxn08894	-999,9854054	1000
rxn08897	-0,006912974	-0,006912974
rxn08926	0,000690955	0,000690955
rxn08927	-999,9984639	999,9869415
rxn08928	-999,9854054	1000
rxn08929	0,00153609	0,00153609
rxn08930	0	0
rxn08958	0,000768616	0,000768616
rxn09010	0	0
rxn09062	0	1000
rxn09063	0	1000
rxn09064	0	1000
rxn09065	0	1000
rxn09066	0	1000
rxn09067	0	1000
rxn09068	0	1000
rxn09069	0	0
rxn09101	0	0
rxn09102	0	0
rxn09103	0	0
rxn09104	0	0

rxn09105	0	0
rxn09106	0	0
rxn09107	0	0
rxn09108	0	0
rxn09109	0	0
rxn09110	0	0
rxn09111	0	0
rxn09112	0	0
rxn09113	0	0
rxn09114	0	0
rxn09176	-1000	1000
rxn09177	0	0,000657835
rxn09197	0	0
rxn09198	0	0
rxn09199	0	0
rxn09200	0	0
rxn09201	0	0
rxn09202	0	0
rxn09203	0	0
rxn09205	0	0
rxn09206	0	0
rxn09207	0	0
rxn09208	0	0
rxn09209	0	0
rxn09210	0	0
rxn09211	0	0
rxn09235	0,028334856	0,028334856
rxn09237	0,029248515	0,029248515
rxn09244	0	0
rxn09264	0	0
rxn09265	0	0
rxn09340	0	0
rxn09341	0	999,6310107
rxn09348	0	0
rxn09355	0	0
rxn09399	0	0
rxn09412	-1000	1000
rxn09473	0	0
rxn09486	0	0
rxn09498	-1000	1000
rxn09499	-1000	1000
rxn09502	0	2,03
rxn09519	0	0
rxn09521	0	0
rxn09531	0	0
rxn09557	0,000254683	1000
rxn09615	0,000690955	0,000690955
rxn09631	0,000254683	0,000254683
rxn09632	0	999,9997453
rxn09889	0	0

rxn09949	0	0
rxn09952	0	0
rxn09988	0	0
rxn09995	0	0
rxn10003	0	0,000657835
rxn10052	-1000	1000
rxn10054	0	999,6310107
rxn10056	0	0,000510507
rxn10058	0	0,000510507
rxn10060	0	0,000510507
rxn10091	-1000	1000
rxn10107	0	0
rxn10110	0	0
rxn10111	0	0
rxn10192	0	0
rxn10202	0	0
rxn10203	0	0
rxn10204	0	0
rxn10205	0	0
rxn10206	0	0
rxn10207	0	0
rxn10208	0	0
rxn10209	0	0
rxn10210	0	0
rxn10211	0	0
rxn10212	0	0
rxn10213	0	0
rxn10214	0	0
rxn10215	0	0
rxn10216	0	0
rxn10217	0	0
rxn10218	0	0
rxn10219	0	0
rxn10220	0	0
rxn10221	0	0
rxn10222	0	0
rxn10223	0	0
rxn10224	0	0
rxn10225	0	0
rxn10226	0	0
rxn10227	0	0
rxn10228	0	0
rxn10229	0	0
rxn10230	0	0
rxn10231	0	0
rxn10232	0	0
rxn10233	0	0
rxn10234	0	0
rxn10235	0	0
rxn10236	0	0

rxn10237	0	0
rxn10238	0	1000
rxn10239	0	1000
rxn10240	0	1000
rxn10241	0	1000
rxn10242	0	1000
rxn10243	0	1000
rxn10253	0	1000
rxn10254	0	1000
rxn10255	0	1000
rxn10256	0	1000
rxn10257	0	1000
rxn10258	0	1000
rxn10259	0	0
rxn10260	0	0
rxn10261	0	0
rxn10262	0	0
rxn10263	0	0
rxn10264	0	0
rxn10265	0	0
rxn10266	0	0
rxn10267	0	0
rxn10268	0	0
rxn10269	0	0
rxn10270	0	0
rxn10289	0	0
rxn10290	0	0
rxn10291	0	0
rxn10292	0	0
rxn10293	0	0
rxn10294	0	0
rxn10295	0	0
rxn10296	0	0
rxn10297	0	0
rxn10363	0	0
rxn10410	0	0
rxn10785	6,28141E-05	6,28141E-05
rxn10790	0,000254683	1000
rxn10798	-1000	-0,000254683
rxn10951	0	0,028334856
rxn11007	0,028334856	0,028334856
rxn11213	0,000254683	1,610412309
rxn11547	0	0
rxn11548	0	0
rxn11550	0	0
rxn11567	0	0
rxn11571	0	0
rxn11577	0	0
rxn11587	0	0
rxn11599	0	0

rxn11601	0	0
rxn11609	0	0
rxn11642	0	0
rxn11732	0	0
rxn11749	0	0
rxn11755	0	0
rxn11759	0	0
rxn11760	0	0
rxn11761	0	0
rxn11765	0	0
rxn11766	0	0
rxn11768	0	0
rxn11772	0	0
rxn11773	0	0
rxn11897	0	0
rxn11899	0	0
rxn11946	0	0
rxn11951	0	0
rxn11984	0	0
rxn12013	0	0
rxn12053	0	0
rxn12054	0	0
rxn12218	-1000	-0,000254683
rxn12221	0,000254683	1000
rxn12510	0,000657835	0,000657835
rxn12778	0	0
rxn12822	-1000	0
rxn12844	0	0
rxn12845	0	0
rxn12846	0	0
rxn12847	0	0
rxn13420	0,000690955	6,590358626
rxn13421	0,000690955	6,590358626
rxn13705	0	0
rxn13741	0	0
rxn13906	-0,007702147	-0,007702147
rxn13936	0,015363179	0,01536318
rxn13963	-1000	-0,008588396
rxn13974	-508,1381613	0
rxn14028	0	0
rxn14029	0	0
rxn14043	0	0
rxn14048	-1000	0
rxn14050	0	0
rxn14054	-1000	0
rxn14063	0	0
rxn14070	0	0
rxn14089	-1000	0
rxn14093	0	0
rxn14120	-0,00101873	-0,00101873

rxn14123	0	0
rxn14132	0	0
rxn14160	0	0
rxn14250	0	0
rxn14270	0	0
rxn14279	0	0
rxn14346	0	0
rxn90002	-7,300215853	1000
rxn90003	0	0
rxn90004	0	0
rxn90005	-0,028845363	-0,028334856
rxn08173	0	500
Biomass_Bacteria	1,142074	1,142074006
t_Cl	0,005153038	0,005153038
t_Sulfate	0	0
t_Cu2+	0,003435359	0,003435359
t_Mg	0,008587254	0,008587254
t_Ca2+	0,005153038	0,005153038
t_NH3	-1,610157626	0
t_H2O	-20,46977278	10
t_Biomass	-1,142074006	-1,142074
t_Butyrate	0	0
t_D-Lactate	0	0
t_Ethanol	-1,303068753	0
t_Formate	-21,06926779	0
t_H2	-0,1	0,5
t_L-Lactate	-8,786223562	0
t_Nitrite	0	0
t_Phosphate	1,517143664	2,011966651
t_Propionate	0	0
t_O2	0	0
t_D-Glucose	0	0,5
t_CO2	-21,06926779	0
t_Acetate	-13,17933534	0
t_Succinate	-6,589667672	0
t_H2S	0	0
Ex_Cl	-0,005153038	-0,005153038
Ex_Sulfate	0	0
Ex_Cu2+	-0,003435359	-0,003435359
Ex_Mg	-0,008587254	-0,008587254
Ex_Ca2+	-0,005153038	-0,005153038
Ex_NH3	0	1,610157626
Ex_H2O	-10	20,46977278
Ex_Biomass	1,142074	1,142074006
Ex_Butyrate	0	0
Ex_D-Lactate	0	0
Ex_Ethanol	0	1,303068753
Ex_Formate	0	21,06926779
Ex_H2	-0,5	0,1
Ex_L-Lactate	0	8,786223562

Ex_Nitrite	0	0
Ex_Phosphate	-2,011966651	-1,517143664
Ex_Propionate	0	0
Ex_O2	0	0
Ex_D-Glucose	-0,5	0
Ex_CO2	0	21,06926779
Ex_Acetate	0	13,17933534
Ex_Succinate	0	6,589667672
Ex_H2S	0	0
t_Fe2	0,007983097	0,007983097
t_fe3	0,007728415	0,007728415
t_Acetaldehyde	-1,303068753	0
t_Adenosine	0	0,494822979
t_AMP	0	0,494822979
t_Amylotriose	0	0
t_BIOT	0	0
t_Choline	0	0
t_Cytidine	0	0
t_Cytosine	0	0
t_DAlanine	0	0
t_Deoxyadenosine	0	0,494822979
t_Deoxycytidine	0	0,365829146
t_Deoxyguanosine	0	0
t_Deoxyinosine	0	0
t_Deoxyuridine	0	0
t_DRibose	0	0,5
t_DSerine	0	0
t_Glycerol	0	0
t_GSH	0	0
t_Guanine	0	0
t_H2S2O3	0	0
t_Heme	0,000254683	0,000254683
t_Homocysteine	0	0
t_HYXN	0	0,494822979
t_Inosine	0	0,494822979
t_LACT	0	0
t_LAlanine	-1,110157626	0,5
t_LArabinose	0	0
t_LArginine	-0,074307339	0,328232069
t_LAsparagine	-0,305078813	0,5
t_LAspartate	-1,110157626	0,5
t_LCysteine	0,107094563	0,5
t_LGlutamate	-1,110157626	0,5
t_LGlutamine	-0,305078813	0,5
t_LHistidine	0,105185015	0,105185016
t_LInositol	0	0
t_LIsoleucine	0,32240749	0,322407492
t_LLeucine	0,499999997	0,5
t_LLysine	-0,424311342	0,380767474
t_LMethionine	-0,222139106	0,170766332

t_LPhenylalanine	-1,404584306	0,205573321
t_LThreonine	-1,021292828	0,5
t_LTryptophan	0,063076747	0,063076747
t_LTyrosine	-1,45711971	0,153037917
t_LValine	0,470420281	0,470420283
t_Maltose	0	0,5
t_Niacin	0	0,002602787
t_Ornithine	0	0
t_PPi	0	0
t_Pyridoxol	0	0
t_XAN	0	0
t_(R)3Hydroxybutanoate	0	0
t_5Deoxyadenosine	0	0
t_Acetoacetate	-6,589667672	0
t_BET	0	0
t_Calomide	0	0
t_Cbl	0	0
t_Citrate	0	0
t_CysGly	0	0
t_Glycine	-1,110157626	0,5
t_Glycolaldehyde	0	0
t_LProline	0,245317495	0,245317497
t_Maltohexaose	0	0
t_Methanol	0	0
t_NAcetylDglucosamine	0	0
t_PM	0	0
t_Putrescine	0	0
t_Pyridoxal	0,000254683	0,000254683
t_Riboflavin	0	0,000509365
t_Sorbitol	0	0
t_Spermidine	0	0
t_Sucrose	0	0,5
t_Taurine	0	0
t_Thiamin	0	0
t_Thymidine	0	0
t_Thyminose	0	0,5
t_Uracil	0	0,365829146
t_Uridine	0	0,365829146
t_Mn2+	0,003435359	0,003435359
t_Formaldehyde	0	0
t_Fumarate	-6,589667672	0
t_Oxidized glutathione	0	0
t_Adenine	0	0
t_Nicotinamide	0	0
t_4-Hydroxybenzoate	0	0
t_Co2+	0,003435359	0,003435359
t_D-Glutamate	0	0
t_Nitrate	0	0
t_Chorismate	0	0
t_Folate	0,00101873	0,00101873

t_Siroheme	0	0
t_Menaquinone 7	0	0
t_2-Demethylmenaquinone 8	0	0
t_Menaquinone 8	0	0
t_Ubiquinone-8	0	0
t_2-Oxobutyrate	0	0
t_3MOP	0	0
t_ABEE	0	0
t_Neu5Ac	0	0
t_Glycerol-3-phosphate	0	0
t_H+	-1000	0,5
t_indol	0	0
t_Nicotinamide ribonucleotide	0	0
t_PAN	0,000657835	0,000657835
t_Pyridoxal phosphate	0	0
t_Zn2+	0,003435359	0,003435359
t_1,2-Diacyl-sn-glycerol dioctadecanoyl	0	0
t_meso-2,6-Diaminopimelate	0	0
t_L-Serine	-1,110157626	0,5
t_D-Fructose	0	0,5
t_D-Mannose	0	0
t_D-Mannitol	0	0
t_beta D-Galactose	0	0
t_L-Fucose	0	0
Ex_Fe2	-0,007983097	-0,007983097
Ex_fe3	-0,007728415	-0,007728415
Ex_Acetaldehyde	0	1,303068753
Ex_Adenosine	-0,494822979	0
Ex_AMP	-0,494822979	0
Ex_Amylotriose	0	0
Ex_BIOT	0	0
Ex_Choline	0	0
Ex_Cytidine	0	0
Ex_Cytosine	0	0
Ex_DAlanine	0	0
Ex_Deoxyadenosine	-0,494822979	0
Ex_Deoxycytidine	-0,365829146	0
Ex_Deoxyguanosine	0	0
Ex_Deoxyinosine	0	0
Ex_Deoxyuridine	0	0
Ex_DRibose	-0,5	0
Ex_DSerine	0	0
Ex_Glycerol	0	0
Ex_GSH	0	0
Ex_Guanine	0	0
Ex_Heme	-0,000254683	-0,000254683
Ex_Homocysteine	0	0
Ex_HYXN	-0,494822979	0
Ex_Inosine	-0,494822979	0
Ex_LACT	0	0

Ex_LAlanine	-0,5	1,110157626
Ex_LArabinose	0	0
Ex_LArginine	-0,328232069	0,074307339
Ex_LAsparagine	-0,5	0,305078813
Ex_LAspartate	-0,5	1,110157626
Ex_LCysteine	-0,5	-0,107094563
Ex_LGlutamate	-0,5	1,110157626
Ex_LGlutamine	-0,5	0,305078813
Ex_LHistidine	-0,105185016	-0,105185015
Ex_LInositol	0	0
Ex_LIsoleucine	-0,322407492	-0,32240749
Ex_LLeucine	-0,5	-0,499999997
Ex_LLysine	-0,380767474	0,424311342
Ex_LMethionine	-0,170766332	0,222139106
Ex_LPhenylalanine	-0,205573321	1,404584306
Ex_LThreonine	-0,5	1,021292828
Ex_LTryptophan	-0,063076747	-0,063076747
Ex_LTyrosine	-0,153037917	1,45711971
Ex_LValine	-0,470420283	-0,470420281
Ex_Maltose	-0,5	0
Ex_Niacin	-0,002602787	0
Ex_Ornithine	0	0
Ex_PPi	0	0
Ex_XAN	0	0
Ex_(R)3Hydroxybutanoate	0	0
Ex_5Deoxyadenosine	0	0
Ex_Acetoacetate	0	6,589667672
Ex_BET	0	0
Ex_Calomide	0	0
Ex_Cbl	0	0
Ex_Citrate	0	0
Ex_CysGly	0	0
Ex_Glycine	-0,5	1,110157626
Ex_Glycolaldehyde	0	0
Ex_LProline	-0,245317497	-0,245317495
Ex_Maltohexaose	0	0
Ex_Methanol	0	0
Ex_NAcetylDglucosamine	0	0
Ex_PM	0	0
Ex_Putrescine	0	0
Ex_Pyridoxal	-0,000254683	-0,000254683
Ex_Riboflavin	-0,000509365	0
Ex_Sorbitol	0	0
Ex_Spermidine	0	0
Ex_Sucrose	-0,5	0
Ex_Taurine	0	0
Ex_Thiamin	0	0
Ex_Thymidine	0	0
Ex_Thyminose	-0,5	0
Ex_Uracil	-0,365829146	0

Ex_Uridine	-0,365829146	0
Ex_Mn2+	-0,003435359	-0,003435359
Ex_Formaldehyde	0	0
Ex_Fumarate	0	6,589667672
Ex_Oxidized glutathione	0	0
Ex_Adenine	0	0
Ex_Nicotinamide	0	0
Ex_4-Hydroxybenzoate	0	0
Ex_Co2+	-0,003435359	-0,003435359
Ex_D-Glutamate	0	0
Ex_Nitrate	0	0
Ex_Folate	-0,00101873	-0,00101873
Ex_Siroheme	0	0
Ex_Menaquinone 7	0	0
Ex_2-Demethylmenaquinone 8	0	0
Ex_Menaquinone 8	0	0
Ex_Ubiquinone-8	0	0
Ex_ABEE	0	0
Ex_Neu5Ac	0	0
Ex_H+	-0,5	1000
Ex_indol	0	0
Ex_Nicotinamide ribonucleotide	0	0
Ex_PAN	-0,000657835	-0,000657835
Ex_Zn2+	-0,003435359	-0,003435359
Ex_1,2-Diacyl-sn-glycerol dioctadecanoyl	0	0
Ex_L-Serine	-0,5	1,110157626
Ex_D-Fructose	-0,5	0
Ex_D-Mannose	0	0
Ex_D-Mannitol	0	0
Ex_beta D-Galactose	0	0
Ex_L-Fucose	0	0
t_Arabinan	0	0
t_Starch	0	0,005
t_Melibiose	0	0
t_Amylose	0	0
Ex_Arabinan	0	0
Ex_Starch	-0,005	0
Ex_Melibiose	0	0
Ex_Amylose	0	0
t_Raffinose_Melitose	0	0
t_Isovaleric_acid	0	0
t_H2O2	0	0
t_Nitric_oxide	0	0
Ex_Raffinose_Melitose	0	0
Ex_Isovaleric_acid	0	0
Ex_H2O2	0	0
Ex_Nitric_oxide	0	0
rxn01207_1	0	0
rxn08972	0	0
rxn08973	0	0

rxn06111	0	1000
rxn13726	0	0
rxn13727	0	0
rxn13729	0	0
rxn08974	0	0
rxn10122	0	0
rxn10123	0	0
rxn10124	0	0
rxn12665	0	0
rxn06097	0	0,005
t_Sulfite	0	0
Ex_Sulfite	0	0
t_Nitrogen	0	0,1
Ex_Nitrogen	-0,1	0

rxn ID	minFlux	max Flux
rxn00001	0	1000
rxn00003	-19,7328215	0
rxn00011	-19,7328215	0
rxn00018	0	0
rxn00020	0	0
rxn00022	0	0,505
rxn00029	0,00101873	0,00101873
rxn00060	0,000254683	0,000254683
rxn00062	0	1000
rxn00065	0	0
rxn00067	0	0
rxn00076	0	1000
rxn00077	0	0,000510507
rxn00085	-1000	0
rxn00097	-1000	1000
rxn00100	0,000657835	0,000657835
rxn00103	0	1000
rxn00104	-1000	0
rxn00105	-999,9973972	1000
rxn00106	-1000	0
rxn00109	0	0
rxn00114	-0,462799224	0,236212601
rxn00119	0,368989262	1000
rxn00122	0,000254683	0,000254683
rxn00124	0,000254683	0,000254683
rxn00126	0,008466195	1000
rxn00127	0,007702147	0,007702147
rxn00131	-1000	1000
rxn00132	0	1000
rxn00134	0	1000
rxn00137	0	0
rxn00138	0	1000
rxn00139	-999,9971425	0
rxn00142	0	0
rxn00143	0,000509365	999,9920432
rxn00145	0	0
rxn00148	-12,29442859	0
rxn00151	-12,29442859	0
rxn00154	0	19,7328215
rxn00157	-19,7328215	0
rxn00159	-1000	1000
rxn00161	-1000	1000
rxn00162	0	0,469052079
rxn00165	0	7,271046527
rxn00170	0	20,188766
rxn00171	0	1,302559388
rxn00173	0	20,188766
rxn00178	-7,115281905	0
rxn00179	0	0

rxn00182	-1000	0
rxn00183	0	0
rxn00184	-1000	0
rxn00187	0	1000
rxn00189	0	1000
rxn00190	0	1000
rxn00192	0	0
rxn00193	0,031494975	0,031494975
rxn00194	0,000690955	7,11597286
rxn00196	0	0
rxn00198	0	1000
rxn00199	0	1000
rxn00200	0	0
rxn00202	0	0
rxn00206	0,004548881	201,0591961
rxn00211	0	0
rxn00214	-1,5	0
rxn00216	0	1000
rxn00221	0	1000
rxn00222	0	0
rxn00224	0,000254683	1000
rxn00225	-20,188766	0
rxn00227	0	20,188766
rxn00239	0,238807673	12,56248477
rxn00242	0	12,3236771
rxn00247	0	0,469052079
rxn00250	-1000	999,9999646
rxn00254	0	0
rxn00256	-0,469052079	0
rxn00258	-1000	999,9999646
rxn00260	-201,6362758	0,469087483
rxn00262	0	201,0546472
rxn00272	-1000	1000
rxn00275	-1,049329616	999,8705094
rxn00278	-1000	0
rxn00283	0,027731841	0,027731841
rxn00285	-0,79914459	6,571902795
rxn00290	-6,730279621	-0,000690955
rxn00293	0,062989949	12,35741854
rxn00297	0	0
rxn00301	0	12,3236771
rxn00303	0	12,3236771
rxn00304	-12,29442859	0
rxn00307	0	0
rxn00313	0	0,459919492
rxn00322	0	0
rxn00328	0	0
rxn00333	0,000254683	201,0549019
rxn00337	0,031494975	0,491414467
rxn00338	0	0,002602787

rxn00340	0	0,459919492
rxn00348	0	0
rxn00350	-0,000254683	-0,000254683
rxn00358	0	0
rxn00360	0	0
rxn00362	0	0
rxn00363	0	1000
rxn00364	-12,29577738	0,000657835
rxn00365	0	1000
rxn00368	0	12,29442859
rxn00369	0	12,3236771
rxn00371	0	1000
rxn00383	0,004294198	0,004294198
rxn00391	0	999,9997453
rxn00392	0,000254683	1000
rxn00394	0	0
rxn00405	0	0
rxn00410	-999,8225103	12,4732671
rxn00411	-12,29577738	0
rxn00412	0	1000
rxn00414	0	0,469052079
rxn00422	-1000	1000
rxn00423	0	7,271046527
rxn00424	-1000	1000
rxn00426	0	0
rxn00433	0	0
rxn00436	0	999,9997453
rxn00437	0	0
rxn00440	0,000254683	1000
rxn00452	0	999,9915338
rxn00453	0	1000
rxn00456	0	1000
rxn00459	-0,003500458	12,28754873
rxn00460	-12,69626161	-0,400484237
rxn00461	0,031494975	0,031494975
rxn00462	0	0
rxn00470	0,045698949	0,045698949
rxn00471	0	0,919838985
rxn00474	0	0
rxn00490	0	0,919838985
rxn00493	-0,919838985	0
rxn00503	-0,919838985	0
rxn00504	-0,919838985	0
rxn00506	0	1,302559388
rxn00510	0	0
rxn00512	-201,0546472	0
rxn00514	0	0
rxn00517	-12,29442859	0
rxn00527	-0,919838985	0
rxn00533	-1000	1000

rxn00541	-1,302559388	0
rxn00543	-1,302559388	0
rxn00545	0	5,649181514
rxn00546	0	0
rxn00547	0	1
rxn00551	0	5,649181514
rxn00552	-0,06298995	999,9370101
rxn00553	0	0
rxn00554	0	5,649181514
rxn00555	0	1000
rxn00556	0	5,649181514
rxn00557	0	5,649181514
rxn00558	-1000	1000
rxn00562	0	0
rxn00565	0	0
rxn00566	0	1000
rxn00567	-0,006252855	-0,006252855
rxn00575	0	1000
rxn00579	-0,5	1000
rxn00585	0	0
rxn00598	-0,000254683	-0,000254683
rxn00611	-0,393669484	0
rxn00612	-0,393669484	0
rxn00615	0	0
rxn00616	0	0,393669484
rxn00622	0	0
rxn00634	0	0
rxn00641	0	0
rxn00645	0,004294198	0,004294198
rxn00647	0	0
rxn00649	0	7,271046527
rxn00650	-0,000254683	-0,000254683
rxn00653	0	0
rxn00670	0	20,188766
rxn00677	-1000	1000
rxn00684	0	0
rxn00685	0	999,9989813
rxn00686	0	0
rxn00687	0	999,9989813
rxn00689	0	0
rxn00690	0	14,23056381
rxn00692	-0,260507082	7,010539445
rxn00693	0	0,393414802
rxn00695	-1000	1000
rxn00701	0	1000
rxn00704	-1000	2
rxn00707	0	1000
rxn00708	0	1000
rxn00709	0	1000
rxn00710	0	0

rxn00711	-999,9971425	0
rxn00712	0	12,29442859
rxn00713	0	12,3236771
rxn00715	0	1000
rxn00726	0	0
rxn00727	0	0
rxn00735	0	0
rxn00737	0	1,521038145
rxn00738	0	0
rxn00740	0	1000
rxn00742	-1000	0,000509365
rxn00745	0	0
rxn00747	-5,83160766	0,002602787
rxn00748	0	0
rxn00758	0	0
rxn00763	0	0
rxn00765	0	0
rxn00770	0,002857469	1000
rxn00772	0,000509365	1000
rxn00775	0	0
rxn00776	0	0
rxn00777	-0,848777222	0,364597609
rxn00778	-1000	1000
rxn00781	-0,003500458	12,28754873
rxn00784	0	1,302559388
rxn00785	-0,182171463	0,884435445
rxn00786	0	5,649181514
rxn00787	0	0
rxn00789	0	0
rxn00790	-0,000254683	-0,000254683
rxn00791	0	0
rxn00792	0	0
rxn00796	0	0
rxn00799	-0,53065213	6,723132236
rxn00800	-0,26805619	0,20099589
rxn00802	0	0,229959746
rxn00806	0	0
rxn00808	0	1000
rxn00816	0	0,5
rxn00817	0	0,5
rxn00818	0	0
rxn00819	0	0
rxn00829	0,000690955	0,000690955
rxn00830	6,28141E-05	6,28141E-05
rxn00831	0	999,9971425
rxn00832	0	0
rxn00834	-999,7290863	1000
rxn00836	-999,9971425	0
rxn00838	-0,26805619	0,20099589
rxn00851	0	1000

rxn00869	0	0
rxn00874	0	0
rxn00879	0	0
rxn00881	0	0
rxn00882	0	0
rxn00883	0	0
rxn00889	0	0
rxn00890	0	0
rxn00898	0	0,919838985
rxn00902	0	0
rxn00903	-0,919838985	0
rxn00907	-14,23005445	0,000509365
rxn00908	-7,780097087	0,034105188
rxn00909	0	0,393669484
rxn00910	-0,393669484	0
rxn00913	0	1000
rxn00915	-999,9707515	0
rxn00916	-999,7319438	0,26805619
rxn00917	0	1000
rxn00929	-1000	1000
rxn00931	-1000	1000
rxn00933	0	0
rxn00938	0	999,9971425
rxn00947	0	1000
rxn00950	-1000	0,392905437
rxn00952	0	1000
rxn00955	0,000509365	999,9920432
rxn00957	0,000254683	0,000254683
rxn00962	0,000254683	0,000254683
rxn00973	-1000	1000
rxn00974	-1000	1000
rxn00977	0	0
rxn00979	0,000254683	0,000254683
rxn00980	0	0
rxn00983	0	0
rxn00985	-20,188766	0
rxn00991	-0,000690955	-0,000690955
rxn00992	0	0
rxn01000	0	0,919838985
rxn01007	0	0
rxn01016	0	0
rxn01018	0	0
rxn01019	0,006252855	0,236212601
rxn01021	0	0
rxn01022	0,007702147	0,007702147
rxn01034	0	0
rxn01042	0	0
rxn01056	-1000	1000
rxn01068	0	0
rxn01069	0	0

rxn01073	0	0
rxn01080	0	0
rxn01100	-1000	0
rxn01101	0	0
rxn01103	0	1000
rxn01106	-12,28754873	0,003500458
rxn01114	0	0
rxn01116	-0,848777222	0,364597609
rxn01117	0	0
rxn01119	0	0
rxn01122	0	0
rxn01124	0	0
rxn01133	0	0
rxn01137	0	0,469052079
rxn01138	-1000	1000
rxn01139	0	0
rxn01169	0	1000
rxn01171	0	1000
rxn01192	0,000254683	0,000254683
rxn01199	0	0
rxn01200	0	1000
rxn01201	-7,11597286	-0,000690955
rxn01202	0	0
rxn01203	0	0
rxn01204	0,000690955	7,11597286
rxn01210	0	0
rxn01211	-14,23030913	0,000509365
rxn01213	6,28141E-05	6,28141E-05
rxn01228	0	0
rxn01236	0	0
rxn01237	0	0
rxn01241	0	19,7328215
rxn01242	0	19,7328215
rxn01249	0	0
rxn01255	0,000254683	0,920093667
rxn01256	0	0,919838985
rxn01265	-999,9997453	0
rxn01268	0	0,919838985
rxn01270	-0,919838985	0
rxn01274	0	0
rxn01276	0	0
rxn01278	0	0
rxn01280	0	0
rxn01281	0	0
rxn01286	0	0
rxn01293	0	0
rxn01297	-999,7319438	999,9971425
rxn01303	0	0
rxn01304	0	0
rxn01305	0	0

rxn01313	0	0
rxn01314	0	0
rxn01316	0	0
rxn01329	0	0
rxn01332	0,000254683	0,920093667
rxn01334	0	0,367096093
rxn01343	0	0,367096093
rxn01346	0	0,367096093
rxn01347	0	0,367096093
rxn01348	0	0,367096093
rxn01351	0	1000
rxn01352	-1000	-0,029248515
rxn01354	-12,29442859	0
rxn01355	0	0
rxn01358	0	999,9971425
rxn01361	0	0
rxn01362	0	0
rxn01365	0	0
rxn01366	-0,33749429	1000
rxn01367	0	0
rxn01368	0	12,29577738
rxn01370	0	1000
rxn01387	-1000	0
rxn01388	-1000	1000
rxn01389	0	0
rxn01396	0	0
rxn01406	0,007702147	0,007702147
rxn01423	0	0
rxn01426	0	0
rxn01434	0	0,229959746
rxn01445	0	999,9707515
rxn01446	-0,029248515	-0,029248515
rxn01457	0	0
rxn01459	0	0
rxn01465	0	0
rxn01466	6,28141E-05	6,28141E-05
rxn01480	0	0
rxn01484	0	0
rxn01485	-0,06298995	-0,062989949
rxn01486	0	0
rxn01492	0	0
rxn01500	-0,000690955	-0,000690955
rxn01509	-999,9707515	24,58197731
rxn01510	0	1000
rxn01513	0,028334856	0,028334856
rxn01518	0,028334856	1000
rxn01519	0	0
rxn01521	0	999,9716651
rxn01522	0	0
rxn01539	-1000	-0,000254683

rxn01544	-999,9971425	0
rxn01548	0,029248515	1000
rxn01549	0	0
rxn01562	0	0
rxn01575	-0,919838985	0
rxn01601	0	0
rxn01602	0	0
rxn01603	0	0
rxn01610	0	0
rxn01615	0	0
rxn01620	0	0
rxn01629	-0,00203746	-0,00203746
rxn01636	0,051951804	0,971790789
rxn01637	-0,971790789	-0,051951804
rxn01643	-0,491414467	-0,031494975
rxn01644	0,031494975	0,491414467
rxn01646	0	999,9971425
rxn01647	0	999,9971425
rxn01649	0	999,9971425
rxn01653	0	0
rxn01667	0	0
rxn01669	0	999,9973972
rxn01670	0	999,9971425
rxn01675	0	0
rxn01679	0	0
rxn01682	0	0
rxn01684	0	0
rxn01685	0	0
rxn01697	0	0
rxn01704	0	0
rxn01706	0	0
rxn01710	0	0
rxn01721	0	0
rxn01731	0	0
rxn01735	0	0
rxn01739	0,000254683	0,920093667
rxn01740	-0,920093667	-0,000254683
rxn01741	0	0
rxn01747	0	0
rxn01757	0	0
rxn01758	-0,004294198	-0,004294198
rxn01763	0	0
rxn01790	0	0
rxn01795	0	0
rxn01799	-0,028334856	0,33749429
rxn01800	0	0,365829146
rxn01807	0	0
rxn01812	0	0
rxn01816	0	1000
rxn01834	0	0

rxn01842	0	0
rxn01851	0	0
rxn01857	0	0
rxn01858	0	0,469052079
rxn01859	-0,469052079	0,494313614
rxn01860	0	0
rxn01870	0	0
rxn01871	-19,7328215	0
rxn01902	0	0
rxn01906	0	0
rxn01911	0	0
rxn01912	0	0
rxn01917	0,051951804	0,971790789
rxn01937	0	0
rxn01946	0	0
rxn01953	0	0
rxn01961	0	999,9971425
rxn01962	0	0
rxn01964	0	0
rxn01966	0	0
rxn01967	0	0
rxn01972	0,031494975	20,22026097
rxn01974	0,031494975	0,491414467
rxn01977	-1000	1000
rxn01982	0	0
rxn01985	0	0,469052079
rxn01986	-0,057583371	0,802559388
rxn01987	-0,5	0
rxn01990	0	0
rxn01996	0	0
rxn01997	0	0
rxn02000	0	0
rxn02003	0	0
rxn02008	0,031494975	0,031494975
rxn02009	0	0
rxn02010	0	0
rxn02011	0,031494975	0,031494975
rxn02012	0	0
rxn02014	0	0
rxn02015	0	0
rxn02029	0	0
rxn02056	0	999,9997453
rxn02078	0	0
rxn02093	0	0
rxn02106	0	0
rxn02122	0	0
rxn02128	0	0
rxn02138	0	0
rxn02139	0	0
rxn02143	0,000254683	0,000254683

rxn02144	0,000254683	0,000254683
rxn02154	0	999,9973972
rxn02155	0,002602787	1000
rxn02160	0	0
rxn02166	0	0
rxn02170	0	0
rxn02171	0,000690955	7,11597286
rxn02175	0,000657835	0,000657835
rxn02185	-0,919838985	19,7328215
rxn02186	0	0,919838985
rxn02187	0	0
rxn02195	0	0
rxn02202	0	0
rxn02203	0	0
rxn02209	0	0
rxn02212	0,000254683	0,920093667
rxn02213	0,000254683	0,920093667
rxn02217	0	0
rxn02235	0	0
rxn02236	0	0
rxn02251	0	0
rxn02264	0,000254683	0,000254683
rxn02284	-0,031494975	0
rxn02285	-0,031494975	0
rxn02286	0,031494975	0,031494975
rxn02288	0	0
rxn02296	0	0
rxn02302	-1000	0
rxn02305	0,000254683	0,000254683
rxn02313	0	0
rxn02314	0	1000
rxn02315	0	12,29442859
rxn02316	0	12,29442859
rxn02317	-1000	0
rxn02321	0	0
rxn02322	0,000690955	0,000690955
rxn02339	0	0
rxn02341	0,000657835	0,000657835
rxn02342	0	19,7328215
rxn02346	0	0
rxn02350	0	0
rxn02351	0	0
rxn02356	-1000	1000
rxn02358	-1000	1000
rxn02369	-0,000254683	-0,000254683
rxn02380	-1000	1000
rxn02400	0	999,9971425
rxn02402	-0,002602787	0
rxn02409	0	0
rxn02449	0	12,3236771

rxn02454	0	0
rxn02465	-0,971790789	-0,051951804
rxn02473	0	0
rxn02476	0,000254683	0,920093667
rxn02483	0,000254683	0,000254683
rxn02484	0,000254683	0,000254683
rxn02507	0	0
rxn02518	0	0
rxn02521	0	0
rxn02522	0	0
rxn02569	0	0
rxn02571	0	0
rxn02581	0	0
rxn02596	0	0
rxn02650	0	0
rxn02729	0	0
rxn02749	0	0
rxn02751	0	0
rxn02762	0	0
rxn02774	0	0
rxn02775	0	0
rxn02789	0	0
rxn02794	0	0
rxn02795	0	0
rxn02796	0	0
rxn02811	0	0
rxn02822	0	0
rxn02834	0	0
rxn02835	0	0
rxn02853	0	0
rxn02875	0	0
rxn02895	0,000254683	0,000254683
rxn02897	0	0
rxn02900	0	0
rxn02914	0	0
rxn02928	-1000	999,968505
rxn02929	-1000	999,968505
rxn02931	0	0
rxn02936	0	0
rxn02937	0,000254683	0,000254683
rxn02988	-0,002602787	0
rxn02996	0	0
rxn03004	0	0,000254683
rxn03005	-0,000254683	0
rxn03008	0	0
rxn03024	0	0
rxn03025	0	0
rxn03026	0	0
rxn03030	0,031494975	20,22026097
rxn03037	0	0

rxn03039	0	0
rxn03047	0	0
rxn03052	0	0
rxn03057	0,007702147	0,007702147
rxn03062	0	0
rxn03063	0	0
rxn03064	0	0
rxn03066	0	0
rxn03068	0	0
rxn03071	0	0
rxn03072	0	0
rxn03075	0,000254683	0,000254683
rxn03084	0,000254683	0,000254683
rxn03086	-20,22026097	-0,031494975
rxn03087	0	0
rxn03094	0	0
rxn03095	0	0
rxn03102	0	0
rxn03106	0	0
rxn03107	0	0
rxn03108	0,000254683	0,000254683
rxn03135	0	0
rxn03136	0	0
rxn03137	0	0
rxn03140	0	0
rxn03141	0	0
rxn03145	0	0
rxn03147	0	0
rxn03150	0	0
rxn03164	0,031494975	0,031494975
rxn03175	0	0
rxn03194	0	0,919838985
rxn03202	0	0
rxn03263	0	0
rxn03264	0	0
rxn03269	0	0
rxn03273	0	0
rxn03282	0	0
rxn03292	0	0
rxn03293	0	0
rxn03295	0	0
rxn03296	0	0
rxn03301	0	0
rxn03304	0	0
rxn03313	0	0
rxn03316	0	0
rxn03333	0	0
rxn03354	0	0
rxn03371	0,031264276	0,031264276
rxn03374	0	0

rxn03379	0	0
rxn03382	0	0
rxn03383	0	0
rxn03384	0	0
rxn03387	0	0
rxn03397	0	0
rxn03402	0	0
rxn03405	0	0
rxn03406	0	0
rxn03407	0	0
rxn03408	0,031494975	0,031494975
rxn03409	0	0
rxn03423	0	0
rxn03424	0	0
rxn03435	-0,919838985	0
rxn03436	0	0,919838985
rxn03437	0	0,919838985
rxn03445	0	0
rxn03446	0	0
rxn03462	0	0
rxn03468	0	0
rxn03483	0	0
rxn03491	0	0
rxn03492	0	0
rxn03512	0	0
rxn03513	0	0
rxn03514	0	0
rxn03535	0	0
rxn03536	0	0
rxn03537	0	0
rxn03538	0	0
rxn03540	0	0
rxn03548	0	1000
rxn03549	0	0
rxn03552	0	0
rxn03553	0	0
rxn03594	0	0
rxn03598	0	0
rxn03638	0,062989949	0,06298995
rxn03641	0,000690955	7,11597286
rxn03642	0,000690955	7,11597286
rxn03649	0	0
rxn03650	0	0
rxn03798	-1000	1000
rxn03838	0	0
rxn03852	0	0
rxn03884	0	0
rxn03891	6,28141E-05	6,28141E-05
rxn03901	0,031494975	0,031494975
rxn03902	0	0

rxn03903	0	0
rxn03904	0,031494975	0,031494975
rxn03907	0	0
rxn03908	0	0
rxn03910	0	0
rxn03919	0	0
rxn03933	0	0
rxn03974	-0,028334856	0
rxn03975	-0,028334856	0
rxn03978	0,006252855	0,006252855
rxn03990	0	0
rxn03991	0	0
rxn04045	0	0
rxn04046	0	0
rxn04047	0	0
rxn04048	0	0
rxn04050	0	0
rxn04052	0	0
rxn04068	0	0
rxn04070	0	0
rxn04082	0	0
rxn04113	0	0
rxn04142	0	0
rxn04234	0	0
rxn04286	0	0
rxn04308	0	0
rxn04345	0	0
rxn04346	0	0
rxn04347	0	0
rxn04348	0	0
rxn04349	0	0
rxn04350	0	0
rxn04351	0	0
rxn04352	0	0
rxn04353	0	0
rxn04354	0	0
rxn04355	0	0
rxn04356	0	0
rxn04357	0	0
rxn04358	0	0
rxn04359	0	0
rxn04360	0	0
rxn04384	0	0
rxn04385	0	0
rxn04413	0	0
rxn04432	0	0
rxn04443	0	0
rxn04482	0	0
rxn04673	0	0
rxn04674	0	0

rxn04676	0	1000
rxn04678	-1000	0
rxn04704	0	0
rxn04726	0	0
rxn04736	0	0
rxn04763	0	0
rxn04794	0	1000
rxn04822	0	0
rxn04909	0	0
rxn04928	0	0
rxn04930	0	0
rxn04943	0	0
rxn04954	-0,393669484	0
rxn04960	0	0
rxn05004	0	0
rxn05028	6,28141E-05	6,28141E-05
rxn05029	0	0
rxn05030	6,28141E-05	6,28141E-05
rxn05039	0	0
rxn05050	0	0
rxn05054	0	0
rxn05087	0	0
rxn05088	0	0
rxn05089	0	0
rxn05092	0,007702147	0,007702147
rxn05104	0,007702147	0,007702147
rxn05105	0,007702147	0,007702147
rxn05106	0,007702147	0,007702147
rxn05108	0,007702147	0,007702147
rxn05115	0	0
rxn05234	0	0
rxn05236	0	0
rxn05247	0	0
rxn05248	0	0
rxn05249	0	0
rxn05250	0	0
rxn05251	0	0
rxn05252	0	0
rxn05269	0	0
rxn05289	0	0
rxn05322	0	0
rxn05323	0	0
rxn05324	0	0
rxn05325	0	0
rxn05326	0	0
rxn05327	0	0
rxn05328	0	0
rxn05329	0	0
rxn05330	0	0
rxn05331	0	0

rxn05332	0	0
rxn05333	0	0
rxn05334	0	0
rxn05335	0	0
rxn05336	0	0
rxn05337	0	0
rxn05338	0	0
rxn05339	0	0
rxn05340	0	0
rxn05341	0	0
rxn05342	0	0
rxn05343	0	0
rxn05344	0	0
rxn05345	0	0
rxn05346	0	0
rxn05347	0	0
rxn05348	0	0
rxn05350	0	0
rxn05457	-1000	0
rxn05465	0	0
rxn05733	0	0
rxn05736	0	1000
rxn05740	-1000	1000
rxn05759	-0,5	0
rxn05760	-202,2863982	1000
rxn05778	0	0
rxn05779	0	0
rxn05794	-1000	0
rxn05824	0	0
rxn05853	0	0
rxn05854	0	0
rxn05871	0	0
rxn05872	0	0
rxn05887	0	0,5
rxn05901	0	0
rxn05927	0	0
rxn05934	0	0
rxn05937	-1000	1000
rxn05938	-19,7328215	0
rxn05939	-0,798565559	1000
rxn05940	-1000	1000
rxn05957	0	1000
rxn05958	0	0
rxn05962	0	0
rxn05979	0	0
rxn05990	0	0
rxn05994	0	0
rxn06005	0	0
rxn06023	0	0
rxn06043	0	0

rxn06044	0	0
rxn06045	0	0
rxn06071	0,009097761	402,1183921
rxn06078	0	0
rxn06090	0	0
rxn06096	0	0
rxn06108	-1000	0
rxn06109	-6,572339067	0,798708318
rxn06139	0	0
rxn06140	0	0
rxn06181	0	1000
rxn06182	0	1000
rxn06195	0	0
rxn06196	0	0
rxn06200	0	0
rxn06201	0	0
rxn06209	0	0
rxn06217	0	0
rxn06218	0	0
rxn06224	0	0
rxn06231	0	0
rxn06280	0	0
rxn06285	0	0
rxn06298	0	0
rxn06300	0	0
rxn06316	0	0
rxn06328	0	0
rxn06348	0	0
rxn06381	0	0
rxn06403	0	0
rxn06432	0	0
rxn06434	0	0
rxn06435	0	0
rxn06437	0	0
rxn06438	0	0
rxn06439	0	0
rxn06440	0	0
rxn06441	0	0
rxn06443	0	0
rxn06444	0	0
rxn06445	0	0
rxn06446	0	0
rxn06447	0	0
rxn06448	0	0
rxn06449	0	0
rxn06485	0	0
rxn06489	0	0
rxn06493	0	0
rxn06538	0	0
rxn06556	0	0

rxn06565	0	0
rxn06584	0	0
rxn06591	0,00203746	0,00203746
rxn06592	0	0
rxn06595	0	0
rxn06624	0	0
rxn06648	0	0
rxn06664	0	0
rxn06672	0	1000
rxn06673	0	1000
rxn06678	0	0
rxn06701	0	0
rxn06726	0	0
rxn06751	0	0
rxn06768	0	0
rxn06799	0	0
rxn06820	0	0
rxn06823	0	0
rxn06831	0	0
rxn06850	0	0
rxn06860	0	0
rxn06864	0	0
rxn06874	0	0
rxn06882	0	0
rxn06887	0	0
rxn06889	0	1000
rxn06890	0	0
rxn06926	0	0
rxn06934	0	0
rxn06936	0	0
rxn06937	0,00203746	0,00203746
rxn06947	0	0
rxn06958	-402,1183921	-0,009097761
rxn06979	0	0
rxn06983	0	0
rxn07056	0	0
rxn07059	0	0
rxn07099	0	0
rxn07193	0	0
rxn07200	0	0,5
rxn07221	0	0
rxn07267	0	0
rxn07292	0	0
rxn07312	0	0
rxn07335	0	0
rxn07405	0	0
rxn07437	0	0
rxn07441	0	20,188766
rxn07450	0,004294198	0,004294198
rxn07456	0	0,469052079

rxn07466	-0,029267931	999,9707321
rxn07486	0	0
rxn07489	0	0
rxn07573	0	0
rxn07577	0	0
rxn07578	0	0
rxn07579	0	0
rxn07584	0	0
rxn07585	0	0
rxn07586	0	0
rxn07587	0	0
rxn07804	0	0
rxn07807	0	0
rxn07832	0	0
rxn07846	0	0
rxn07849	0	0
rxn07987	0	0
rxn07989	0	0
rxn07991	0	0
rxn07992	0	0
rxn07993	0	0
rxn07994	0	0
rxn08035	0	0
rxn08038	0	0
rxn08043	0	0,919838985
rxn08067	-1000	1000
rxn08083	0	0
rxn08084	0	0
rxn08085	0	0
rxn08086	0	0
rxn08087	0	0
rxn08088	0	0
rxn08089	0	0
rxn08094	0	1000
rxn08114	0	0
rxn08126	0	0
rxn08127	0	0
rxn08128	0	0
rxn08129	0	0
rxn08171	0	0
rxn08180	0	0
rxn08194	-1000	1000
rxn08294	0	0
rxn08295	0	0
rxn08296	0	0
rxn08297	0	0
rxn08298	0	0
rxn08299	0	0
rxn08300	0	0
rxn08306	0	0

rxn08307	0	0
rxn08308	0	0
rxn08309	0	0
rxn08310	0	0
rxn08311	0	0
rxn08312	0	0
rxn08352	0	0
rxn08386	0	0
rxn08390	0	0
rxn08392	0	0
rxn08394	0	0
rxn08396	0	0
rxn08398	0	0
rxn08433	0	0
rxn08438	0	0
rxn08448	0	0
rxn08449	0	0
rxn08451	0	0
rxn08453	0	0
rxn08454	0	1000
rxn08455	0	0
rxn08456	0	0
rxn08457	0	0
rxn08519	0,057583371	0,057583371
rxn08546	0	0
rxn08547	0	1000
rxn08548	0	0
rxn08549	0	0
rxn08550	0	0
rxn08551	0	0
rxn08552	0	0
rxn08571	0	1000
rxn08582	0	0,5
rxn08605	0	0
rxn08607	0	0
rxn08615	-1000	1000
rxn08647	0	0
rxn08668	0	0
rxn08669	0	0
rxn08764	0	0,919838985
rxn08792	0	0
rxn08793	0	0
rxn08796	0	0
rxn08797	0	1000
rxn08798	0	0
rxn08799	0	1000
rxn08800	0	0
rxn08801	0	1000
rxn08802	0	0
rxn08803	0	0

rxn08804	0	0
rxn08805	0	0
rxn08806	0	0
rxn08807	0	0
rxn08808	0	0
rxn08809	0	0
rxn08810	0	0
rxn08811	0	0
rxn08812	0	0
rxn08813	0	0
rxn08814	0	0
rxn08815	0	0
rxn08816	0	0
rxn08817	0	0
rxn08818	0	0
rxn08819	0	0
rxn08820	0	0
rxn08821	0	0
rxn08822	0	0
rxn08823	0	0
rxn08838	0	0
rxn08839	0	0
rxn08840	0	0
rxn08841	0	0
rxn08842	0	0
rxn08843	0	0
rxn08844	0	0
rxn08845	0	0
rxn08846	0	0
rxn08847	0	0
rxn08848	0	0
rxn08849	0	0
rxn08850	0	0
rxn08851	0	0
rxn08857	0	0
rxn08889	0,000768616	0,000768616
rxn08890	0,006222019	0,006222019
rxn08891	0,000768616	0,000768616
rxn08892	0,013058474	0,013058474
rxn08893	0,0061455	0,0061455
rxn08894	0,00153609	0,00153609
rxn08897	-0,006912974	-0,006912974
rxn08926	0,000690955	0,000690955
rxn08928	0,00153609	0,00153609
rxn08929	0,00153609	0,00153609
rxn08958	0,000768616	0,000768616
rxn09010	0	0
rxn09016	0	12,3236771
rxn09108	0	0
rxn09109	0	0

rxn09110	0	0
rxn09111	0	0
rxn09112	0	0
rxn09113	0	0
rxn09114	0	0
rxn09176	-1000	1000
rxn09177	0	0,000657835
rxn09197	0	0
rxn09198	0	0
rxn09199	0	0
rxn09200	0	0
rxn09201	0	0
rxn09202	0	0
rxn09203	0	0
rxn09205	0	0
rxn09206	0	0
rxn09207	0	0
rxn09208	0	0
rxn09209	0	0
rxn09210	0	0
rxn09211	0	0
rxn09235	0,028334856	0,028334856
rxn09237	0,029248515	0,029248515
rxn09244	0	0
rxn09340	0	0
rxn09341	0	12,29442859
rxn09348	0	999,6310107
rxn09355	0	0
rxn09395	0	0
rxn09398	-1000	0,5
rxn09399	0	0
rxn09445	0	0
rxn09446	0	0
rxn09447	0	0
rxn09486	0	0
rxn09502	0	1000
rxn09523	0	0
rxn09531	0	0
rxn09557	0,000254683	0,000254683
rxn09616	0,000690955	0,000690955
rxn09633	0,000254683	0,000254683
rxn09888	0	0
rxn09889	0	0
rxn09952	0	0
rxn09978	0	0
rxn09979	0	0
rxn10003	0	0,000657835
rxn10019	0	0
rxn10020	0	0
rxn10021	0	0

rxn10052	-1000	1000
rxn10054	0	12,29577738
rxn10056	0	0,000510507
rxn10058	0	0,000510507
rxn10060	0	0,000510507
rxn10091	-1000	1000
rxn10110	0	0
rxn10111	0	0
rxn10192	0	0
rxn10193	0	0
rxn10202	0	1000
rxn10203	0	1000
rxn10204	0	1000
rxn10205	0	0
rxn10206	0	0
rxn10207	0	0
rxn10208	0	0
rxn10209	0	0
rxn10210	0	0
rxn10211	0	0
rxn10212	0	0
rxn10213	0	0
rxn10214	0	0
rxn10215	0	0
rxn10216	0	0
rxn10217	0	0
rxn10218	0	0
rxn10219	0	0
rxn10220	0	0
rxn10221	0	0
rxn10222	0	0
rxn10223	0	0
rxn10224	0	0
rxn10225	0	0
rxn10226	0	0
rxn10227	0	0
rxn10228	0	0
rxn10229	0	0
rxn10230	0	0
rxn10231	0	0
rxn10232	0	0
rxn10233	0	0
rxn10234	0	0
rxn10235	0	0
rxn10236	0	0
rxn10237	0	0
rxn10253	0	0
rxn10254	0	0
rxn10255	0	0
rxn10256	0	0

rxn10257	0	0
rxn10258	0	0
rxn10259	0	0
rxn10260	0	0
rxn10261	0	0
rxn10262	0	0
rxn10263	0	0
rxn10264	0	0
rxn10289	0	0
rxn10290	0	0
rxn10291	0	0
rxn10292	0	0
rxn10293	0	0
rxn10294	0	0
rxn10295	0	0
rxn10296	0	0
rxn10297	0	0
rxn10298	0	0
rxn10299	0	0
rxn10300	0	0
rxn10301	0	0
rxn10302	0	0
rxn10303	0	0
rxn10304	0	0
rxn10305	0	0
rxn10306	0	0
rxn10363	0	0
rxn10404	0	0
rxn10405	0	0
rxn10406	0	0
rxn10407	0	0
rxn10408	0	0
rxn10409	0	0
rxn10410	0	0
rxn10434	0	0
rxn10816	0,000254683	0,000254683
rxn10951	0	0,028334856
rxn11007	0,028334856	0,028334856
rxn11511	0	0
rxn11513	0	0
rxn11547	0	0
rxn11548	0	0
rxn11550	0	0
rxn11564	0	0
rxn11567	0	0
rxn11571	0	0
rxn11587	0	0
rxn11599	0	0
rxn11609	0	0
rxn11641	0	0

rxn11732	0	0
rxn11749	0	0
rxn11757	-999,9971425	0
rxn11759	0	999,9971425
rxn11760	-999,9971425	0
rxn11761	0	0
rxn11765	0	0
rxn11766	0	0
rxn11768	0	0
rxn11772	0	0
rxn11773	0	0
rxn11787	0	0
rxn11788	0	0
rxn11789	0	0
rxn11790	0	0
rxn11791	0	0
rxn11858	0	0
rxn11859	0	0
rxn11878	0	0
rxn11879	0	0
rxn11951	0	0
rxn11965	0	0
rxn12008	-6,28141E-05	-6,28141E-05
rxn12218	-1000	-0,000254683
rxn12221	0,000254683	1000
rxn12239	0,000254683	0,000254683
rxn12510	0,000657835	0,000657835
rxn12649	-999,9989813	0
rxn12778	0	0
rxn12822	-1000	0
rxn13147	0,000254683	0,000254683
rxn13420	0,000690955	7,11597286
rxn13421	0,000690955	7,11597286
rxn13477	6,28141E-05	6,28141E-05
rxn13705	0	0
rxn13719	0	0
rxn13720	0	0
rxn13728	0	0
rxn13741	0	0
rxn13936	0,015363179	0,01536318
rxn13974	-19,7328215	0
rxn13994	-0,031264276	-0,031264276
rxn14012	6,28141E-05	6,28141E-05
rxn14014	0	0
rxn14043	0	0
rxn14048	-1000	0
rxn14050	0	0
rxn14054	-1000	0
rxn14058	-0,003126428	-0,003126428
rxn14063	0	0

rxn14070	0	0
rxn14089	-1000	0
rxn14093	0	0
rxn14120	-1000	-0,00101873
rxn14132	0	0
rxn14144	0	0
rxn14178	-1000	1000
rxn14191	0	0
rxn14250	0	0
rxn14270	0	0
rxn14279	0	0
rxn14346	0	0
rxn90002	-12,28754873	1000
rxn90003	0	0
rxn90004	0	0
rxn90005	-0,028845363	-0,028334856
rxn08173	0	500
Biomass_Bacteria	1,142074	1,142074006
t_Cl	0,005153038	0,005153038
t_Sulfate	0	0
t_Cu2+	0,003435359	0,003435359
t_Mg	0,008587254	0,008587254
t_Ca2+	0,005153038	0,005153038
t_NH3	0	0
t_H2O	-17,50733369	9,280289754
t_Biomass	-1,142074006	-1,142074
t_Butyrate	0	0
t_D-Lactate	0	0
t_Ethanol	-1,302559388	0
t_Formate	-14,23056381	0
t_H2	0	0,5
t_L-Lactate	0	0
t_Nitrite	0	0
t_Phosphate	1,517653029	2,011966651
t_Propionate	-1,52154751	0
t_O2	0	0
t_D-Glucose	0	0,5
t_CO2	-14,23056381	0
t_Acetate	-20,11145787	0
t_Succinate	-7,115281905	0
t_H2S	0	0
Ex_Cl	-0,005153038	-0,005153038
Ex_Sulfate	0	0
Ex_Cu2+	-0,003435359	-0,003435359
Ex_Mg	-0,008587254	-0,008587254
Ex_Ca2+	-0,005153038	-0,005153038
Ex_NH3	0	0
Ex_H2O	-9,280289754	17,50733369
Ex_Biomass	1,142074	1,142074006
Ex_Butyrate	0	0

Ex_D-Lactate	0	0
Ex_Ethanol	0	1,302559388
Ex_Formate	0	14,23056381
Ex_H2	-0,5	0
Ex_L-Lactate	0	0
Ex_Nitrite	0	0
Ex_Phosphate	-2,011966651	-1,517653029
Ex_Propionate	0	1,52154751
Ex_O2	0	0
Ex_D-Glucose	-0,5	0
Ex_CO2	0	14,23056381
Ex_Acetate	0	20,11145787
Ex_Succinate	0	7,115281905
Ex_H2S	0	0
t_Fe2	0,007983097	0,007983097
t_fe3	0,007728415	0,007728415
t_Acetaldehyde	-1,302559388	0
t_Adenosine	0	0,494313614
t_AMP	0	0,494313614
t_Amylotriose	0	0
t_BIOT	0	0
t_Choline	0	0
t_Cytidine	0	0
t_Cytosine	0	0
t_DAlanine	0	0
t_Deoxyadenosine	0	0,494313614
t_Deoxycytidine	0	0,365829146
t_Deoxyguanosine	0	0
t_Deoxyinosine	0	0
t_Deoxyuridine	0	0
t_DRibose	0	0,5
t_Glycerol	0	0
t_GSH	0	0
t_Guanine	0	0
t_H2S2O3	0	0
t_Heme	0,000254683	0,000254683
t_Homocysteine	0	0
t_HYXN	0	0,469052079
t_Inosine	0	0,469052079
t_LACT	0	0,5
t_LAlanine	-0,419838985	0,5
t_LArginine	0,092019466	0,321979214
t_LAsparagine	-0,192445762	0,267473732
t_LAspartate	0,030947921	0,5
t_LCysteine	0,106585198	0,5
t_LGlutamate	-0,419838985	0,5
t_LGlutamine	0,040080508	0,5
t_LHistidine	0,105185015	0,105185016
t_LInositol	0	0
t_LIsoleucine	-0,597431495	0,322407492

t_LLeucine	0,499999997	0,5
t_LLysine	-0,079152021	0,380767474
t_LMethionine	-0,222139106	0,171275697
t_LPhenylalanine	-0,714265665	0,205573321
t_LThreonine	-0,419838985	0,5
t_LTryptophan	0,063076747	0,063076747
t_LTyrosine	-0,766546386	0,153292599
t_LValine	-0,449418704	0,470420283
t_Maltose	0	0,5
t_Niacin	0	0,002602787
t_Ornithine	0	0
t_PPi	0	0
t_Pyridoxol	0	0
t_XAN	0	0
t_(R)3Hydroxybutanoate	0	0
t_1,3Propanediol	0	0
t_5Deoxyadenosine	0	0
t_Acetoacetate	-6,729588666	0
t_Calomide	0	0
t_Cbl	0	0
t_Citrate	0	0
t_CysGly	0	0
t_Dulcose	0	0
t_Glycine	-0,419838985	0,5
t_Glycolaldehyde	0	0
t_LProline	-0,67452149	0,245317497
t_Maltohexaose	0	0
t_Methanol	0	0
t_NAcetylDglucosamine	0	0
t_PM	0	0
t_Putrescine	0	0
t_Pyridoxal	0,000254683	0,000254683
t_Riboflavin	0,000509365	0,000509365
t_Salicin	0	0
t_Sorbitol	0	0
t_Spermidine	0	0
t_Sucrose	0	0,5
t_Taurine	0	0
t_Thiamin	0	0
t_Thyminose	0	0,5
t_TRHL	0	0
t_Uracil	0	0,365829146
t_Uridine	0	0,365829146
t_Ursin	0	0
t_Mn2+	0,003435359	0,003435359
t_Formaldehyde	0	0
t_Fumarate	-7,115281905	0
t_Oxidized glutathione	0	0
t_Adenine	0	0
t_Nicotinamide	0	0

t_4-Hydroxybenzoate	0	0
t_Co2+	0,003435359	0,003435359
t_D-Glutamate	0	0
t_Nitrate	0	0
t_Chorismate	0	0
t_Folate	0,00101873	0,00101873
t_N-Acetyl-D-mannosamine	0	0
t_Siroheme	0	0
t_Pimelate	0	0
t_Menaquinone 7	0	0
t_2-Demethylmenaquinone 8	0	0
t_Menaquinone 8	0	0
t_Ubiquinone-8	0	0
t_2-Oxobutyrate	0	0
t_3MOP	0	0
t_Neu5Ac	0	0
t_Glycerol-3-phosphate	0	0
t_H+	-1000	0,5
t_indol	0	0
t_Nicotinamide ribonucleotide	0	0
t_PAN	0,000657835	0,000657835
t_Pyridoxal phosphate	0	0
t_Zn2+	0,003435359	0,003435359
t_1,2-Diacyl-sn-glycerol dioctadecanoyl	0	0
t_meso-2,6-Diaminopimelate	0	0
t_L-Serine	-0,419838985	0,5
t_D-Fructose	0	0,5
t_D-Mannose	0	0
t_D-Mannitol	0	0
t_beta D-Galactose	0	0,5
t_L-Fucose	0	0
Ex_Fe2	-0,007983097	-0,007983097
Ex_fe3	-0,007728415	-0,007728415
Ex_Acetaldehyde	0	1,302559388
Ex_Adenosine	-0,494313614	0
Ex_AMP	-0,494313614	0
Ex_Amylotriose	0	0
Ex_BIOT	0	0
Ex_Choline	0	0
Ex_Cytidine	0	0
Ex_Cytosine	0	0
Ex_DAlanine	0	0
Ex_Deoxyadenosine	-0,494313614	0
Ex_Deoxycytidine	-0,365829146	0
Ex_Deoxyguanosine	0	0
Ex_Deoxyinosine	0	0
Ex_Deoxyuridine	0	0
Ex_DRibose	-0,5	0
Ex_Glycerol	0	0
Ex_GSH	0	0

Ex_Guanine	0	0
Ex_Heme	-0,000254683	-0,000254683
Ex_Homocysteine	0	0
Ex_HYXN	-0,469052079	0
Ex_Inosine	-0,469052079	0
Ex_LACT	-0,5	0
Ex_LAlanine	-0,5	0,419838985
Ex_LArginine	-0,321979214	-0,092019466
Ex_LAsparagine	-0,267473732	0,192445762
Ex_LAspartate	-0,5	-0,030947921
Ex_LCysteine	-0,5	-0,106585198
Ex_LGlutamate	-0,5	0,419838985
Ex_LGlutamine	-0,5	-0,040080508
Ex_LHistidine	-0,105185016	-0,105185015
Ex_LInositol	0	0
Ex_LIsoleucine	-0,322407492	0,597431495
Ex_LLeucine	-0,5	-0,499999997
Ex_LLysine	-0,380767474	0,079152021
Ex_LMethionine	-0,171275697	0,222139106
Ex_LPhenylalanine	-0,205573321	0,714265665
Ex_LThreonine	-0,5	0,419838985
Ex_LTryptophan	-0,063076747	-0,063076747
Ex_LTyrosine	-0,153292599	0,766546386
Ex_LValine	-0,470420283	0,449418704
Ex_Maltose	-0,5	0
Ex_Niacin	-0,002602787	0
Ex_Ornithine	0	0
Ex_PPi	0	0
Ex_XAN	0	0
Ex_(R)3Hydroxybutanoate	0	0
Ex_5Deoxyadenosine	0	0
Ex_Acetoacetate	0	6,729588666
Ex_Calomide	0	0
Ex_Cbl	0	0
Ex_Citrate	0	0
Ex_CysGly	0	0
Ex_Dulcose	0	0
Ex_Glycine	-0,5	0,419838985
Ex_Glycolaldehyde	0	0
Ex_LProline	-0,245317497	0,67452149
Ex_Maltohexaose	0	0
Ex_Methanol	0	0
Ex_NAcetylDglucosamine	0	0
Ex_PM	0	0
Ex_Putrescine	0	0
Ex_Pyridoxal	-0,000254683	-0,000254683
Ex_Riboflavin	-0,000509365	-0,000509365
Ex_Salicin	0	0
Ex_Sorbitol	0	0
Ex_Spermidine	0	0

Ex_Sucrose	-0,5	0
Ex_Taurine	0	0
Ex_Thiamin	0	0
Ex_Thyminose	-0,5	0
Ex_TRHL	0	0
Ex_Uracil	-0,365829146	0
Ex_Uridine	-0,365829146	0
Ex_Ursin	0	0
Ex_Mn2+	-0,003435359	-0,003435359
Ex_Formaldehyde	0	0
Ex_Fumarate	0	7,115281905
Ex_Oxidized glutathione	0	0
Ex_Adenine	0	0
Ex_Nicotinamide	0	0
Ex_4-Hydroxybenzoate	0	0
Ex_Co2+	-0,003435359	-0,003435359
Ex_D-Glutamate	0	0
Ex_Nitrate	0	0
Ex_Folate	-0,00101873	-0,00101873
Ex_N-Acetyl-D-mannosamine	0	0
Ex_Siroheme	0	0
Ex_Pimelate	0	0
Ex_Menaquinone 7	0	0
Ex_2-Demethylmenaquinone 8	0	0
Ex_Menaquinone 8	0	0
Ex_Ubiquinone-8	0	0
Ex_Neu5Ac	0	0
Ex_H+	-0,5	1000
Ex_indol	0	0
Ex_Nicotinamide ribonucleotide	0	0
Ex_PAN	-0,000657835	-0,000657835
Ex_Zn2+	-0,003435359	-0,003435359
Ex_1,2-Diacyl-sn-glycerol dioctadecanoyl	0	0
Ex_L-Serine	-0,5	0,419838985
Ex_D-Fructose	-0,5	0
Ex_D-Mannose	0	0
Ex_D-Mannitol	0	0
Ex_beta D-Galactose	-0,5	0
Ex_L-Fucose	0	0
t_Starch	0	0,005
t_octanoate	0	0
t_Melibiose	0	0,5
t_Amylose	0	0
Ex_Starch	-0,005	0
Ex_Melibiose	-0,5	0
Ex_Amylose	0	0
t_Raffinose_Melitose	0	0
t_Isovaleric_acid	0	0
t_H2O2	0	0
t_Nitric_oxide	0	0

Ex_Raffinose_Melitose	0	0
Ex_Isovaleric_acid	0	0
Ex_H2O2	0	0
Ex_Nitric_oxide	0	0
rxn01207_1	0	0
rxn08972	0	0
rxn08973	0	0
rxn06111	0	1000
rxn13726	0	0
rxn13727	0	0
rxn13729	0	0
rxn08974	0	0
rxn10122	0	0
rxn10123	0	0
rxn10124	0	0
rxn12665	0	0
rxn06097	0	0,005
t_Sulfite	0	0
Ex_Sulfite	0	0
t_Nitrogen	0	0
Ex_Nitrogen	0	0

rxn ID	minFlux	max Flux
rxn00001	0	455,9453526
rxn00003	-3,750474177	0
rxn00011	-3,750474177	0
rxn00016	0	0
rxn00020	0	0
rxn00022	0	0,505
rxn00029	0,000782701	0,000782702
rxn00056	0	0
rxn00060	0,000195675	0,000195675
rxn00062	0	455,9453526
rxn00065	0	11,79273408
rxn00066	0	0
rxn00076	0	11,79273408
rxn00077	0	0,000392228
rxn00085	-2,807184891	0
rxn00100	0,000505422	0,000505422
rxn00101	0	1,012617419
rxn00105	-11,79273408	11,79273408
rxn00106	-1000	0
rxn00109	0	0
rxn00114	-0,764801722	0,764801722
rxn00119	0,283498504	231,126782
rxn00122	0,000195675	0,000195675
rxn00124	0,000195675	0,000195675
rxn00126	0,000587026	0,000587026
rxn00131	-0,380178161	11,53136677
rxn00133	0	0
rxn00137	0	0
rxn00139	-11,79273408	0
rxn00140	0	11,79273408
rxn00143	0,000391351	0,000391351
rxn00146	0	0,1
rxn00148	-11,79273408	0
rxn00151	-12,17886826	-0,386134181
rxn00154	0	12,67903152
rxn00157	-12,67903152	0
rxn00159	-1000	1000
rxn00161	-1000	1000
rxn00165	0	0,293051234
rxn00171	0	0,497804575
rxn00173	0	13,3911521
rxn00178	-7,552026683	0
rxn00179	0	0
rxn00184	-2,807184891	0
rxn00187	0	455,9453526
rxn00189	0	456,2185929
rxn00190	0,00199975	11,79473383
rxn00192	0	0
rxn00193	0,024197935	0,02419795

rxn00196	0	0
rxn00198	0	911,8907052
rxn00199	0	911,8907052
rxn00202	0	0
rxn00206	0	0
rxn00211	0	0
rxn00213	-461,9937581	11,48453222
rxn00214	-1,5	0
rxn00216	0	458,4753526
rxn00222	0	0
rxn00224	0,000195675	1000
rxn00225	-13,3911521	0
rxn00227	0	13,3911521
rxn00239	0,205950514	11,9986846
rxn00247	0	0,764801722
rxn00250	-0,764828924	-2,72015E-05
rxn00260	2,72015E-05	0,764828924
rxn00262	0	0
rxn00272	-1000	1000
rxn00275	-3,733923069	0,781352831
rxn00279	0	0,764801722
rxn00283	0,021306678	0,021306691
rxn00290	-6,696106918	-0,000530868
rxn00293	0,04839587	0,0483959
rxn00297	0	0
rxn00299	0	0
rxn00301	0	11,79273408
rxn00302	0	0
rxn00303	0	0
rxn00304	-11,79273408	0
rxn00307	0	0
rxn00313	0	0,764801722
rxn00322	0	0
rxn00337	0,030115579	0,794917302
rxn00338	0	0
rxn00340	0	11,79273408
rxn00342	0	12,08723108
rxn00350	-0,000195675	-0,000195675
rxn00358	0	0
rxn00360	0	455,9453526
rxn00361	0	455,9453526
rxn00362	0	455,9453526
rxn00364	-230,8432835	0,000505422
rxn00365	0	455,9453526
rxn00368	0	455,9453526
rxn00369	0	11,79273408
rxn00371	0	1000
rxn00379	0	0
rxn00388	0	0
rxn00391	0	455,9453526

rxn00392	0,000195675	455,9455483
rxn00394	0	1,012617419
rxn00405	0	0
rxn00410	-456,0822256	230,9796508
rxn00411	-11,79273408	0
rxn00412	0	456,2185929
rxn00414	0	0,764801722
rxn00416	0	11,79273408
rxn00422	-1000	1000
rxn00423	0	0,293051234
rxn00424	-1000	1000
rxn00426	0	0
rxn00433	0	0
rxn00436	0	11,79273408
rxn00437	0	0
rxn00440	0,000195675	11,79292976
rxn00459	-0,000195675	11,46042581
rxn00460	-11,79273408	0
rxn00461	0,024197935	0,02419795
rxn00470	0,035446197	7,587472881
rxn00474	0	0
rxn00490	0	0
rxn00493	-0,99560915	0
rxn00499	-12,67903152	0
rxn00500	-12,77903152	0
rxn00506	0	0,497804575
rxn00507	0	0,497804575
rxn00512	0	0
rxn00514	0	0
rxn00517	-11,79273408	0
rxn00527	-0,99560915	0
rxn00533	-999,9999728	2,72015E-05
rxn00543	-0,497804575	0
rxn00545	0	5,481408455
rxn00546	0	0
rxn00547	0	1
rxn00551	0	5,481408455
rxn00552	-0,0483959	456,1701971
rxn00554	0	5,481408455
rxn00555	0	456,2185929
rxn00556	0	5,481408455
rxn00557	0	5,481408455
rxn00558	-1000	1000
rxn00559	0	0
rxn00565	0	0
rxn00566	0	0,714068584
rxn00575	0	455,9453526
rxn00577	-455,4453526	0,5
rxn00608	0	0
rxn00611	-4,559046565	0

rxn00612	-4,559046565	0
rxn00615	0	0
rxn00616	0	4,559046565
rxn00622	0	0
rxn00623	0	0
rxn00624	0	0
rxn00641	0	0
rxn00646	0	0
rxn00647	0	0
rxn00649	0	0,293051234
rxn00650	-0,000195675	-0,000195675
rxn00653	0	0
rxn00670	0	0,283508463
rxn00684	0	0
rxn00685	0	911,8907052
rxn00686	0	0
rxn00687	0	911,8907052
rxn00689	0	0
rxn00690	0	3,06772E-07
rxn00692	-0,022944047	-0,022943726
rxn00693	0,000391351	0,000391351
rxn00695	-23,4813279	461,4578936
rxn00701	0	1,5
rxn00704	-453,9453526	2
rxn00707	0	455,9453526
rxn00709	0	154,0683894
rxn00710	0	0
rxn00711	-11,79273408	0
rxn00712	0	154,0683894
rxn00713	0	11,79273408
rxn00714	0	0
rxn00715	0	228,2319769
rxn00717	0	227,9726763
rxn00726	0	0
rxn00727	0	0
rxn00729	0	0
rxn00735	0	0
rxn00737	0,000195675	0,283704138
rxn00741	0	0
rxn00742	-0,000195675	-0,000195675
rxn00743	0	0
rxn00747	-5,481408455	0
rxn00748	0	0
rxn00758	0	0
rxn00763	0	0
rxn00765	0	0
rxn00770	0,002195425	11,79492951
rxn00772	0,002586776	0,500391351
rxn00775	0	0
rxn00777	0	0

rxn00778	-456,1227759	6,156901694
rxn00781	-0,000195675	11,46042581
rxn00784	0	0,497804575
rxn00785	0,000195675	0,49800025
rxn00786	0	5,481408455
rxn00790	-0,000195675	-0,000195675
rxn00791	0	0
rxn00792	0	0
rxn00796	0	0
rxn00799	-0,55832034	6,637988977
rxn00800	-0,205950641	0,558851208
rxn00802	0	0,764801722
rxn00806	0	0
rxn00808	0	1,5
rxn00816	0	0,5
rxn00817	0	0,5
rxn00818	0	0
rxn00819	0	0
rxn00829	0,000530868	0,000530868
rxn00830	4,82607E-05	4,82608E-05
rxn00832	0	0
rxn00834	0,205950514	0,205950641
rxn00836	-0,764801722	0
rxn00838	-0,205950641	0,558851208
rxn00851	0	455,9453526
rxn00853	0	0
rxn00855	0	0
rxn00856	0,006252837	7,55827952
rxn00869	0	0
rxn00871	0	4,552541895
rxn00872	-4,552541895	0
rxn00874	0	0
rxn00881	0	0
rxn00882	0	0
rxn00883	0	0
rxn00889	0	0
rxn00890	0	0
rxn00898	0	3,750474177
rxn00902	0	0
rxn00903	-3,750474177	0
rxn00907	0,000391044	0,000391351
rxn00909	-4,558459539	0,000587026
rxn00910	-4,559046565	0
rxn00915	-5,896367042	0
rxn00916	-456,0126424	0,205950641
rxn00917	0	456,2185929
rxn00918	0	0
rxn00926	0	0,764801722
rxn00929	-1000	1000
rxn00931	-1000	1000

rxn00938	0	0
rxn00947	0	11,79273408
rxn00955	0,000391351	0,000391351
rxn00973	-1000	1000
rxn00974	-1000	1000
rxn00977	0	0
rxn00980	0	0
rxn00985	-0,283508463	0
rxn00991	-0,000530868	-0,000530868
rxn01000	0	0,99560915
rxn01016	0	0
rxn01018	0	0
rxn01019	0	0,764801722
rxn01021	0	0
rxn01025	0	0
rxn01034	0	0
rxn01049	0	0
rxn01069	0	0
rxn01071	0	0
rxn01073	0	0
rxn01100	-23,25315989	0
rxn01101	0	0
rxn01103	0	23,25315989
rxn01106	-11,46042581	0,000195675
rxn01108	-1000	1000
rxn01109	-1000	1000
rxn01119	0	0
rxn01133	0	0
rxn01138	0	0,37978681
rxn01139	0	0
rxn01146	0	0
rxn01153	0	0
rxn01169	0	458,4753526
rxn01171	0	458,4753526
rxn01199	0	0
rxn01200	0	1000
rxn01201	-7,552557551	-0,000530868
rxn01204	0,000530868	7,552557551
rxn01210	0	0
rxn01211	0,000195369	0,000391351
rxn01213	4,82607E-05	4,82608E-05
rxn01228	0	0
rxn01236	-4,552541895	0
rxn01237	0	0
rxn01255	0,000195675	0,995804825
rxn01256	0	0,99560915
rxn01257	0	0
rxn01265	-0,001999751	-0,00199975
rxn01268	0	0,99560915
rxn01274	0	0

rxn01286	0	0
rxn01300	0	0
rxn01313	0	0
rxn01314	0	0
rxn01329	0	0
rxn01332	0,000195675	0,995804825
rxn01334	0	0,497804575
rxn01343	0	0,497804575
rxn01346	0	0,497804575
rxn01347	0	0,497804575
rxn01348	0	0,497804575
rxn01351	0	23,25315989
rxn01352	-0,022471969	-0,022471955
rxn01354	-11,79273408	0
rxn01358	0	0,37978681
rxn01361	0	0
rxn01362	0	0
rxn01366	-228,2319769	11,65224413
rxn01367	0	0
rxn01368	0	227,9726763
rxn01370	0	23,25315989
rxn01374	0	0
rxn01377	0	0
rxn01379	0	0
rxn01387	-911,8907052	0
rxn01388	-1000	1000
rxn01390	0	0
rxn01396	0	0
rxn01423	0	0
rxn01426	0	0
rxn01434	0	0,764801722
rxn01437	0	0
rxn01446	-0,022471969	-0,022471955
rxn01452	-999,9994691	0
rxn01459	0	7,552361876
rxn01465	0	0
rxn01466	4,82607E-05	4,82608E-05
rxn01484	0	0
rxn01485	-0,0483959	-0,04839587
rxn01486	0	0
rxn01492	0	0
rxn01500	-0,000530868	-0,000530868
rxn01506	0	0
rxn01509	0	23,25315989
rxn01510	0	23,25315989
rxn01513	0,021769981	0,021769994
rxn01517	0	0
rxn01518	0,021769981	0,021769994
rxn01519	0	0
rxn01539	-11,79292976	-0,000195675

rxn01544	0	0
rxn01548	0,022471955	5,918838997
rxn01549	0	0
rxn01562	0	0
rxn01575	-0,283508463	0
rxn01601	0	0
rxn01602	0	0
rxn01603	0	0
rxn01607	0,000530868	0,000530868
rxn01610	0	0
rxn01620	0	0
rxn01629	-0,001565404	-0,001565403
rxn01636	-0,212369499	7,339657184
rxn01637	-7,339657184	0,212369499
rxn01641	0	0
rxn01643	-0,794917302	-0,030115579
rxn01644	0,024197935	0,788999657
rxn01646	0	0
rxn01647	0	0
rxn01648	-455,9453526	0
rxn01649	0	0
rxn01650	0	0
rxn01653	0	0
rxn01654	0	0
rxn01667	0	0
rxn01669	0	11,79273408
rxn01675	0	0
rxn01679	0	0
rxn01682	0	0
rxn01684	0	0
rxn01704	0	0
rxn01706	0	0
rxn01710	0	0
rxn01734	0	0
rxn01735	0	0
rxn01737	0	0
rxn01739	0,000195675	0,995804825
rxn01740	-0,995804825	-0,000195675
rxn01741	0	0
rxn01757	0	0
rxn01772	0	0
rxn01799	-0,021769994	0,259300728
rxn01800	0	0,281070722
rxn01807	0	0
rxn01834	0	0
rxn01840	0	0
rxn01841	0	0
rxn01851	0	7,552361876
rxn01859	0	0,37978681
rxn01860	0	0

rxn01870	0	0
rxn01871	0	0
rxn01895	0	0
rxn01906	0	0
rxn01917	-0,212369499	7,339657184
rxn01937	0	0
rxn01943	0	0
rxn01944	0	0
rxn01953	0	0
rxn01962	0	0
rxn01964	0	0
rxn01967	0	0
rxn01972	0,024197935	7,220507252
rxn01973	-6,813908456	0
rxn01974	0,024197935	0,788999657
rxn01977	-1000	1000
rxn01982	0	0
rxn01985	0	0
rxn01986	0	0,497804575
rxn01991	0	0
rxn01997	0	0
rxn02000	0	0
rxn02003	0	0
rxn02008	0,024197935	0,02419795
rxn02011	0,024197935	0,02419795
rxn02012	0	0
rxn02015	0	0
rxn02020	0	0
rxn02023	0	0
rxn02046	0	0
rxn02056	0	999,9998043
rxn02090	0	0
rxn02093	0	0
rxn02106	0	0
rxn02122	0	0
rxn02128	0	0
rxn02138	0	0
rxn02139	0	0
rxn02144	0	0
rxn02154	0	11,79273408
rxn02155	0,00199975	11,79473383
rxn02160	0	0
rxn02161	0	0
rxn02167	0	999,9994691
rxn02171	0,000530868	7,552557551
rxn02175	0,000505422	0,000505422
rxn02185	-3,750474177	0,283508463
rxn02186	0	3,750474177
rxn02187	0	0
rxn02195	0	0

rxn02200	0	0
rxn02201	0	0
rxn02212	0,000195675	0,995804825
rxn02213	0,000195675	0,995804825
rxn02219	0	0
rxn02222	0	0
rxn02228	0	0
rxn02263	0	0
rxn02264	0,000195675	0,000195675
rxn02283	0	0
rxn02284	-0,02419795	0
rxn02285	-0,02419795	0
rxn02286	0,024197935	0,02419795
rxn02287	-911,8907052	1000
rxn02302	-0,000195675	-0,000195675
rxn02305	0,000195675	0,000195675
rxn02314	0	461,6860616
rxn02315	0	236,7391451
rxn02316	0	461,6860616
rxn02317	-461,6860616	0
rxn02318	0	0
rxn02319	0	0
rxn02320	0	0
rxn02322	0,000530868	0,000530868
rxn02339	0	0
rxn02341	0,000505422	0,000505422
rxn02350	0	0
rxn02351	0	0
rxn02356	-1000	1000
rxn02358	-1000	1000
rxn02364	0	0
rxn02365	0	0
rxn02373	-1000	1000
rxn02375	0	0
rxn02380	-1000	1000
rxn02402	0	0
rxn02409	0	0
rxn02454	0	0
rxn02465	-7,339657184	0,212369499
rxn02473	0	0
rxn02476	0,000195675	0,995804825
rxn02483	0	0
rxn02484	0	0
rxn02495	0	0
rxn02503	0	0
rxn02504	0	0
rxn02507	0	0
rxn02518	0	0
rxn02521	0	0
rxn02522	0	0

rxn02525	0	0
rxn02527	0	1000
rxn02528	-1000	0
rxn02571	0	0
rxn02581	0	0
rxn02596	0	0
rxn02597	0	0
rxn02729	0	0
rxn02749	0	0
rxn02751	0	0
rxn02760	0	0
rxn02762	0	0
rxn02774	-911,8907052	0
rxn02776	0	0
rxn02789	0	0
rxn02795	0	0
rxn02796	0	0
rxn02811	0	0
rxn02821	0	0
rxn02822	0	0
rxn02845	0	0
rxn02853	0	0
rxn02875	0	0
rxn02895	0,000195675	0,000195675
rxn02897	0	0
rxn02900	0	0
rxn02922	0	0
rxn02928	-1000	999,9758021
rxn02929	-1000	999,9758021
rxn02931	0	0
rxn02936	0	0
rxn02937	0,000195675	0,000195675
rxn02988	0	0
rxn02990	0	0
rxn03004	0	0,000195675
rxn03005	-0,000195675	0
rxn03030	0,024197935	7,220507252
rxn03031	-6,813908456	0
rxn03047	0	0
rxn03062	0	0
rxn03068	0	0
rxn03075	0	0
rxn03080	0	0
rxn03084	0,000195675	0,000195675
rxn03086	-7,220507252	-0,024197935
rxn03087	0	6,813908456
rxn03094	0	0
rxn03095	0	0
rxn03102	0	0
rxn03106	0	0

rxn03108	0,000195675	0,000195675
rxn03136	0	0
rxn03137	0	0
rxn03140	0	0
rxn03141	0	0
rxn03150	0	0
rxn03164	0,024197935	0,02419795
rxn03167	0	0
rxn03174	0	0
rxn03194	0	0,283508463
rxn03251	0	0
rxn03263	0	0
rxn03264	0	0
rxn03269	0	0
rxn03273	0	0
rxn03282	0	0
rxn03333	0	0
rxn03335	0	0
rxn03336	0	0
rxn03340	0	0
rxn03341	0	0
rxn03343	0	0
rxn03344	0	0
rxn03354	0	0
rxn03362	0	0
rxn03372	0	0
rxn03373	0	0
rxn03374	0	0
rxn03397	0	0
rxn03400	0	0
rxn03402	0	0
rxn03405	0	0
rxn03406	0	0
rxn03407	0	0
rxn03408	0,024197935	0,02419795
rxn03409	0	0
rxn03419	0	0
rxn03421	0	0
rxn03423	0	0
rxn03435	-0,283508463	0
rxn03436	0	0,283508463
rxn03437	0	0,283508463
rxn03446	0	0
rxn03462	0	0
rxn03465	0	0
rxn03467	0	0
rxn03468	0	0
rxn03481	0	0
rxn03482	0	0
rxn03483	0	0

rxn03492	0	0
rxn03535	0	0
rxn03536	0	0
rxn03537	0	0
rxn03538	0	0
rxn03548	0	911,8907052
rxn03549	0	0
rxn03552	0	0
rxn03553	0	0
rxn03598	0	0
rxn03638	0,04839587	0,0483959
rxn03641	0,000530868	7,552557551
rxn03642	0,000530868	7,552557551
rxn03838	0	0
rxn03841	0	0
rxn03852	0	0
rxn03861	0	0
rxn03870	0	0
rxn03885	0	0
rxn03901	0,024197935	0,02419795
rxn03902	0	0
rxn03903	0	0
rxn03904	0,024197935	0,02419795
rxn03907	0	0
rxn03908	0	0
rxn03909	0	0
rxn03910	0	0
rxn03933	0	0
rxn03958	0	0
rxn03978	0	0,033333333
rxn03990	0	0
rxn03991	0	0
rxn04045	0	0
rxn04050	0	0
rxn04068	0	0
rxn04082	0	0
rxn04113	0	0
rxn04234	0	0
rxn04308	0	0
rxn04385	0	0
rxn04413	0	0
rxn04432	0	0
rxn04443	0	0
rxn04604	0	0
rxn04674	0	0
rxn04676	0	1000
rxn04678	-1000	0
rxn04704	0	0
rxn04726	0	0
rxn04736	0	0

rxn04750	0	0
rxn04786	0,005917644	0,005917648
rxn04794	0	1000
rxn04907	0	0
rxn04954	-4,559046565	0
rxn05005	-1000	0
rxn05006	-1000	0
rxn05010	0	0
rxn05029	0	0
rxn05030	4,82607E-05	4,82608E-05
rxn05039	0	0
rxn05087	0	0
rxn05088	0	0
rxn05115	0	0
rxn05233	0	0
rxn05234	0	0
rxn05236	0	0
rxn05247	0	0
rxn05248	0	0
rxn05249	0	0
rxn05250	0	0
rxn05251	0	0
rxn05252	0	0
rxn05269	0	0
rxn05289	0	0
rxn05322	0	0
rxn05323	0	0
rxn05324	0	0
rxn05325	0	0
rxn05326	0	0
rxn05327	0	0
rxn05328	0	0
rxn05336	0	0
rxn05337	0	0
rxn05338	0	0
rxn05339	0	0
rxn05340	0	0
rxn05341	0	0
rxn05342	0	0
rxn05351	0	0
rxn05352	0	0
rxn05353	0	0
rxn05354	0	0
rxn05355	0	0
rxn05356	0	0
rxn05357	0	0
rxn05457	-11,79273408	0
rxn05733	0	0
rxn05736	0	11,79273408
rxn05740	-23,78952976	460,8908966

rxn05759	-0,5	0
rxn05778	0	0
rxn05779	0	0
rxn05794	-911,8907052	0
rxn05853	0	0
rxn05854	0	0
rxn05871	0	0
rxn05872	0	0
rxn05874	0	0
rxn05878	0	0
rxn05887	0	0,5
rxn05893	-0,133333333	0
rxn05899	0	0
rxn05901	0	0
rxn05927	0	0
rxn05937	-965,8725665	1000
rxn05938	-12,67903152	0
rxn05939	0,000444876	1000
rxn05940	-1000	979,6181162
rxn05962	0	0
rxn05994	0	0
rxn05995	0	0
rxn06023	0	0
rxn06043	0	0
rxn06044	0	0
rxn06045	0	0
rxn06075	0	0
rxn06080	0	0
rxn06090	0	0
rxn06096	0	0
rxn06108	-1000	-58,3735035
rxn06109	-6,814439324	-0,000530868
rxn06139	0	0
rxn06140	0	0
rxn06181	0	455,9453526
rxn06182	0	455,9453526
rxn06195	0	0
rxn06196	0	0
rxn06200	0	0
rxn06201	0	0
rxn06219	0	0
rxn06224	0	0
rxn06231	0	0
rxn06280	0	0
rxn06285	0	0
rxn06298	0	0
rxn06300	0	0
rxn06316	0	0
rxn06328	0	0
rxn06347	0	0

rxn06348	0	0
rxn06394	0	0
rxn06403	0	0
rxn06432	0	0
rxn06434	0	0
rxn06435	0	0
rxn06437	0	0
rxn06438	0	0
rxn06439	0	0
rxn06440	0	0
rxn06441	0	0
rxn06443	0	0
rxn06444	0	0
rxn06445	0	0
rxn06446	0	0
rxn06447	0	0
rxn06448	0	0
rxn06449	0	0
rxn06485	0	0
rxn06538	0	0
rxn06556	0	0
rxn06565	0	0
rxn06581	0	0
rxn06584	0	0
rxn06591	0,001565403	0,001565404
rxn06592	0	0
rxn06624	0	0
rxn06641	0	0
rxn06648	0	0
rxn06664	0	0
rxn06671	0	0
rxn06672	0	1000
rxn06673	0	1000
rxn06678	0	0
rxn06701	0	0
rxn06726	0	0
rxn06729	0	0
rxn06737	0	0
rxn06751	0	0
rxn06752	0	0
rxn06760	0	0
rxn06768	0	0
rxn06823	0	0
rxn06831	0	0
rxn06850	0	0
rxn06860	0	0
rxn06864	0	0
rxn06882	0	0
rxn06883	0	0
rxn06887	0	0

rxn06889	0	911,8907052
rxn06890	0	0
rxn06936	0	0
rxn06937	0,001565403	0,001565404
rxn06947	0	0
rxn06958	0	0
rxn06979	0	0
rxn07056	0	0
rxn07099	0	0
rxn07189	0	0
rxn07199	0	0
rxn07200	0	0,5
rxn07267	0	0
rxn07292	0	0
rxn07456	0	0,764801722
rxn07465	0,005917644	0,005917648
rxn07466	-1000	1000
rxn07489	0	0
rxn07573	0	0
rxn07577	0	0
rxn07579	0	0
rxn07587	0	0
rxn07645	0	0
rxn07679	0	0
rxn07683	0	0
rxn07687	0	0
rxn07804	0	0
rxn07807	0	0
rxn07832	0	0
rxn07846	0	0
rxn07849	0	0
rxn07987	0	0
rxn07989	0	0
rxn07991	0	0
rxn07992	0	0
rxn07993	0	0
rxn07994	0	0
rxn08025	0	0
rxn08040	0	0
rxn08043	0	0,283508463
rxn08083	0	0
rxn08084	0	0
rxn08085	0	0
rxn08086	0	0
rxn08087	0	0
rxn08088	0	0
rxn08089	0	0
rxn08126	0	0
rxn08127	0	0
rxn08128	0	0

rxn08129	0	0
rxn08131	0,000195675	0,000195675
rxn08171	0	0
rxn08180	0	0
rxn08194	-1000	0
rxn08294	0	0
rxn08295	0	0
rxn08296	0	0
rxn08297	0	0
rxn08298	0	0
rxn08299	0	0
rxn08300	0	0
rxn08306	0	0
rxn08307	0	0
rxn08308	0	0
rxn08309	0	0
rxn08310	0	0
rxn08311	0	0
rxn08312	0	0
rxn08352	0	0
rxn08386	0	0
rxn08387	0	0
rxn08390	0	0
rxn08391	0	0
rxn08392	0	0
rxn08393	0	0
rxn08394	0	0
rxn08395	0	0
rxn08396	0	0
rxn08397	0	0
rxn08398	0	0
rxn08399	0	0
rxn08413	0	0
rxn08433	0	0
rxn08438	0	0
rxn08448	0	0
rxn08449	0	0
rxn08451	0	0
rxn08453	0	0
rxn08454	0	11,79273408
rxn08455	0	0
rxn08456	0	0
rxn08457	0	0
rxn08519	0,044241937	0,044241964
rxn08546	0	0
rxn08547	0	11,79273408
rxn08548	0	0
rxn08549	0	0
rxn08550	0	0
rxn08551	0	0

rxn08552	0	0
rxn08582	0	0,5
rxn08605	0	0
rxn08607	0	0
rxn08615	-23,4813279	461,4578936
rxn08668	0	0
rxn08669	0	0
rxn08713	0	0
rxn08764	0	0,283508463
rxn08783	0	0
rxn08796	0	0
rxn08797	0	11,79273408
rxn08798	0	0
rxn08799	0	11,79273408
rxn08800	0	0
rxn08801	0	11,79273408
rxn08802	0	0
rxn08803	0	0
rxn08804	0	0
rxn08805	0	0
rxn08806	0	0
rxn08807	0	0
rxn08808	0	0
rxn08809	0	0
rxn08810	0	0
rxn08811	0	0
rxn08812	0	0
rxn08813	0	0
rxn08814	0	0
rxn08815	0	0
rxn08816	0	0
rxn08817	0	0
rxn08818	0	0
rxn08819	0	0
rxn08820	0	0
rxn08821	0	0
rxn08822	0	0
rxn08823	0	0
rxn08838	0	0
rxn08839	0	0
rxn08840	0	0
rxn08841	0	0
rxn08842	0	0
rxn08843	0	0
rxn08844	0	0
rxn08845	0	0
rxn08846	0	0
rxn08847	0	0
rxn08848	0	0
rxn08849	0	0

rxn08850	0	0
rxn08851	0	0
rxn08857	0	0
rxn08889	0,000590536	0,000590536
rxn08890	0,004780446	0,004780449
rxn08891	0,000590536	0,000590536
rxn08892	-999,9887868	1000
rxn08893	-999,9940982	999,9946887
rxn08894	-999,9887868	1000
rxn08897	-0,005311317	-0,005311314
rxn08926	0,000530868	0,000530868
rxn08927	-999,9988198	999,989967
rxn08928	-999,9887868	1000
rxn08929	0,001180194	0,001180195
rxn08930	0	0
rxn08958	0,000590536	0,000590536
rxn09016	0,022471955	5,918838997
rxn09101	0	0
rxn09102	0	0
rxn09103	0	0
rxn09104	0	0
rxn09105	0	0
rxn09106	0	0
rxn09107	0	0
rxn09108	0	0
rxn09109	0	0
rxn09110	0	0
rxn09111	0	0
rxn09112	0	0
rxn09113	0	0
rxn09114	0	0
rxn09177	0	0,000505422
rxn09197	0	0
rxn09198	0	0
rxn09199	0	0
rxn09200	0	0
rxn09201	0	0
rxn09202	0	0
rxn09203	0	0
rxn09205	0	0
rxn09206	0	0
rxn09207	0	0
rxn09208	0	0
rxn09209	0	0
rxn09210	0	0
rxn09211	0	0
rxn09235	0,021769981	0,021769994
rxn09237	0,022471955	0,022471969
rxn09240	0	0
rxn09264	0	0

rxn09265	0	0
rxn09398	-461,9937581	11,48453222
rxn09399	0	0
rxn09412	-1000	1000
rxn09445	0	0
rxn09446	0	0
rxn09447	0	0
rxn09473	0	0
rxn09486	0	0
rxn09502	0	458,4753526
rxn09519	0	0
rxn09531	0	0
rxn09557	0,000195675	911,8909009
rxn09631	0,000195675	0,000195675
rxn09889	0	0
rxn09949	0	0
rxn09952	0	0
rxn09978	0	0
rxn09979	0	0
rxn09988	0	0
rxn09992	0	0
rxn09995	0	0
rxn10003	0	0,000505422
rxn10019	0	0
rxn10020	0	0
rxn10021	0	0
rxn10054	0	230,8432835
rxn10056	0	0,000392228
rxn10058	0	0,000392228
rxn10060	0	0,000392228
rxn10091	0	1000
rxn10107	0	0
rxn10110	0	0
rxn10111	0	0
rxn10192	0	0
rxn10193	0	0
rxn10202	0	11,79273408
rxn10203	0	11,79273408
rxn10204	0	11,79273408
rxn10205	0	0
rxn10206	0	0
rxn10207	0	0
rxn10208	0	0
rxn10209	0	0
rxn10210	0	0
rxn10211	0	0
rxn10212	0	0
rxn10213	0	0
rxn10214	0	0
rxn10215	0	0

rxn10216	0	0
rxn10217	0	0
rxn10218	0	0
rxn10219	0	0
rxn10220	0	0
rxn10221	0	0
rxn10222	0	0
rxn10223	0	0
rxn10224	0	0
rxn10225	0	0
rxn10226	0	0
rxn10227	0	0
rxn10228	0	0
rxn10229	0	0
rxn10230	0	0
rxn10231	0	0
rxn10232	0	0
rxn10233	0	0
rxn10234	0	0
rxn10235	0	0
rxn10236	0	0
rxn10237	0	0
rxn10253	0	0
rxn10254	0	0
rxn10255	0	0
rxn10256	0	0
rxn10257	0	0
rxn10258	0	0
rxn10259	0	0
rxn10260	0	0
rxn10261	0	0
rxn10262	0	0
rxn10263	0	0
rxn10264	0	0
rxn10265	0	0
rxn10266	0	0
rxn10267	0	0
rxn10268	0	0
rxn10269	0	0
rxn10270	0	0
rxn10289	0	0
rxn10290	0	0
rxn10291	0	0
rxn10292	0	0
rxn10293	0	0
rxn10294	0	0
rxn10295	0	0
rxn10296	0	0
rxn10297	0	0
rxn10298	0	0

rxn10299	0	0
rxn10300	0	0
rxn10301	0	0
rxn10302	0	0
rxn10303	0	0
rxn10304	0	0
rxn10305	0	0
rxn10306	0	0
rxn10363	0	0
rxn10404	0	0
rxn10405	0	0
rxn10406	0	0
rxn10407	0	0
rxn10408	0	0
rxn10409	0	0
rxn10410	0	0
rxn10785	4,82607E-05	4,82608E-05
rxn10951	0,021769981	0,021769994
rxn11007	0,021769981	0,021769994
rxn11547	0	0
rxn11548	0	0
rxn11550	0	0
rxn11567	0	0
rxn11571	0	0
rxn11587	0	0
rxn11599	0	0
rxn11609	0	0
rxn11703	0	0
rxn11732	0	0
rxn11749	0	0
rxn11759	0	0
rxn11760	0	0
rxn11761	0	0
rxn11765	0	0
rxn11766	0	0
rxn11768	0	0
rxn11772	0	0
rxn11773	0	0
rxn11810	-1000	0
rxn11811	0	1000
rxn11835	0	0
rxn11878	0	0
rxn11951	0	0
rxn11965	0	0
rxn11984	0	0
rxn12013	0	0
rxn12033	0	0
rxn12053	0	0
rxn12054	0	0
rxn12218	-1000	-0,000195675

rxn12221	0,000195675	1000
rxn12510	0,000505422	0,000505422
rxn12649	-999,9992173	0
rxn13420	0,000530868	1000
rxn13421	0,000530868	1000
rxn13705	0	0
rxn13717	0	0
rxn13718	0	0
rxn13734	0	0
rxn13735	0	0
rxn13741	0	0
rxn13906	-0,005917648	-0,005917644
rxn13936	0,0118037	0,011803707
rxn13974	-12,67903152	0
rxn13975	0	0
rxn13977	0	0
rxn13983	0	0
rxn13995	0	0
rxn14028	0	0
rxn14048	-999,9995551	0
rxn14050	0	0
rxn14054	-911,8907052	0
rxn14059	0	0
rxn14063	0	0
rxn14070	0	0
rxn14089	-911,8907052	0
rxn14093	0	0
rxn14120	-1000	-0,000782701
rxn14122	0	0
rxn14132	0	0
rxn14156	0	0
rxn14172	0	0
rxn14173	-0,1	0
rxn14178	-979,3346077	1000
rxn14182	0	0
rxn14191	0	0
rxn14244	0	0
rxn14248	0	0
rxn14278	0	0
rxn14346	0	0
rxn90002	-11,46042581	11,79273408
rxn90003	0	0
rxn90004	0	0
rxn90005	-0,022162223	-0,021769981
rxn08173	29,18648632	499,9997346
Biomass_Bacteria	0,877468	0,877468538
t_Cl	0,003959136	0,003959138
t_Sulfate	0,00329928	0,003299282
t_Cu2+	0,002639424	0,002639425
t_Mg	0,006597682	0,006597686

t_Ca2+	0,003959136	0,003959138
t_NH3	-2,807184891	0
t_H2O	-17,21629312	10
t_Biomass	-0,877468538	-0,877468
t_Butyrate	-4,552541895	0
t_D-Lactate	-12,67903152	0
t_Ethanol	-0,497804575	0
t_Formate	-13,62781691	0
t_H2	0	0,5
t_L-Lactate	-12,67903152	0
t_Nitrite	0	0
t_Phosphate	1,166029494	1,54581702
t_Propionate	-0,283508463	0
t_O2	0	0
t_D-Glucose	0	0,5
t_CO2	-13,62781691	0
t_Acetate	-20,49626294	-0,024728803
t_Succinate	-6,813908456	0
t_H2S	-0,42101735	0
Ex_Cl	-0,003959138	-0,003959136
Ex_Sulfate	-0,003299282	-0,00329928
Ex_Cu2+	-0,002639425	-0,002639424
Ex_Mg	-0,006597686	-0,006597682
Ex_Ca2+	-0,003959138	-0,003959136
Ex_NH3	0	2,807184891
Ex_H2O	-10	17,21629312
Ex_Biomass	0,877468	0,877468538
Ex_Butyrate	0	4,552541895
Ex_D-Lactate	0	12,67903152
Ex_Ethanol	0	0,497804575
Ex_Formate	0	13,62781691
Ex_H2	-0,5	0
Ex_L-Lactate	0	12,67903152
Ex_Nitrite	0	0
Ex_Phosphate	-1,54581702	-1,166029494
Ex_Propionate	0	0,283508463
Ex_O2	0	0
Ex_D-Glucose	-0,5	0
Ex_CO2	0	13,62781691
Ex_Acetate	0,024728803	20,49626294
Ex_Succinate	0	6,813908456
Ex_H2S	0	0,42101735
t_Fe2	0,006133501	0,006133505
t_fe3	0,005937826	0,00593783
t_Acetaldehyde	-0,497804575	0
t_Adenosine	0	0,37978681
t_AMP	0	0,37978681
t_Amylotriose	0	0
t_BIOT	0	0
t_Choline	0	0

t_Cytidine	0	0
t_Cytosine	0	0
t_DAlanine	0	0
t_Deoxyadenosine	0	0,37978681
t_Deoxycytidine	0	0,281070722
t_Deoxyguanosine	0	0
t_Deoxyinosine	0	0
t_Deoxyuridine	0	0
t_DRibose	0,002195425	0,5
t_DSerine	0	0
t_Glycerol	0	0
t_GSH	0	0
t_Guanine	0	0
t_H2S2O3	0	0
t_Heme	0,000195675	0,000195675
t_Homocysteine	0	0
t_HYXN	0	0,37978681
t_Inosine	0	0,37978681
t_LACT	0	0,5
t_LAlanine	-3,250474177	0,5
t_LArabinose	0	0
t_LArginine	-0,437618544	0,5
t_LAsparagine	-0,264801722	0,5
t_LAspartate	-0,264801722	0,5
t_LCysteine	0,07898265	0,5
t_LGlutamate	-3,250474177	0,5
t_LGlutamine	-1,375237088	0,5
t_LHistidine	0,080814803	0,080814852
t_LInositol	0	0
t_LIsoleucine	-0,035799246	0,247709368
t_LLeucine	0,38415549	0,384155726
t_LLysine	-0,472253891	0,292548011
t_LMethionine	0,131201648	0,131201728
t_LPhenylalanine	-0,83766491	0,157944337
t_LThreonine	0,216491537	0,5
t_LTryptophan	0,048462558	0,048462587
t_LTyrosine	-0,878028438	0,117580784
t_LValine	-3,389045107	0,361429291
t_Maltose	0	0,5
t_Niacin	0,00199975	0,001999751
t_Ornithine	0	0
t_PPi	0	0
t_Pyridoxol	0	0
t_XAN	0	0
t_5Deoxyadenosine	0	0
t_Acetoacetate	-6,69557605	0
t_Calomide	0	0
t_Cbl	0	0
t_Citrate	0	0
t_CysGly	0	0

t_Glycine	0,499999693	0,5
t_Glycolaldehyde	0	0
t_LProline	0,188480126	0,188480242
t_Maltohexaose	0	0
t_Methanol	0	0
t_NAcetylDglucosamine	0	0
t_PM	0	0
t_Putrescine	0	0
t_Pyridoxal	0,000195675	0,000195675
t_Riboflavin	0,000391351	0,000391351
t_Salicin	0	0
t_Sorbitol	0	0
t_Spermidine	0	0
t_Sucrose	0	0,5
t_Taurine	0	0
t_Thiamin	0	0
t_Thymidine	0	0
t_TRHL	0	0
t_Uracil	0	0,281070722
t_Uridine	0	0,281070722
t_Ursin	0	0
t_Mn2+	0,002639424	0,002639425
t_Formaldehyde	0	0
t_Fumarate	-6,813908456	0
t_Oxidized glutathione	0	0
t_Adenine	0	0
t_Nicotinamide	0	0
t_Co2+	0,002639424	0,002639425
t_D-Glutamate	0	0
t_Nitrate	0	0,1
t_Chorismate	0	0
t_Folate	0,000782701	0,000782702
t_N-Acetyl-D-mannosamine	0	0
t_Siroheme	0	0
t_Selenate	0	0
t_Menaquinone 7	0	0
t_2-Demethylmenaquinone 8	0	0
t_Menaquinone 8	0	0
t_Ubiquinone-8	0	0
t_2-Oxobutyrate	0	0
t_3MOP	0	0
t_ABEE	0	0
t_Neu5Ac	0	0
t_Glycerol-3-phosphate	0	0
t_H+	-1000	-88,10929482
t_indol	0	0
t_Nicotinamide ribonucleotide	0	0
t_PAN	0,000505422	0,000505422
t_Pyridoxal phosphate	0	0
t_Zn2+	0,002639424	0,002639425

t_1,2-Diacyl-sn-glycerol dioctadecanoyl	0	0
t_meso-2,6-Diaminopimelate	0	0
t_L-Serine	0,206948766	0,5
t_D-Fructose	0	0,5
t_D-Mannose	0	0
t_D-Mannitol	0	0
t_beta D-Galactose	0	0,5
t_L-Fucose	0	0
Ex_Fe2	-0,006133505	-0,006133501
Ex_fe3	-0,00593783	-0,005937826
Ex_Acetaldehyde	0	0,497804575
Ex_Adenosine	-0,37978681	0
Ex_AMP	-0,37978681	0
Ex_Amylotriose	0	0
Ex_BIOT	0	0
Ex_Choline	0	0
Ex_Cytidine	0	0
Ex_Cytosine	0	0
Ex_DAlanine	0	0
Ex_Deoxyadenosine	-0,37978681	0
Ex_Deoxycytidine	-0,281070722	0
Ex_Deoxyguanosine	0	0
Ex_Deoxyinosine	0	0
Ex_Deoxyuridine	0	0
Ex_DRibose	-0,5	-0,002195425
Ex_DSerine	0	0
Ex_Glycerol	0	0
Ex_GSH	0	0
Ex_Guanine	0	0
Ex_Heme	-0,000195675	-0,000195675
Ex_Homocysteine	0	0
Ex_HYXN	-0,37978681	0
Ex_Inosine	-0,37978681	0
Ex_LACT	-0,5	0
Ex_LAlanine	-0,5	3,250474177
Ex_LArabinose	0	0
Ex_LArginine	-0,5	0,437618544
Ex_LAsparagine	-0,5	0,264801722
Ex_LAspartate	-0,5	0,264801722
Ex_LCysteine	-0,5	-0,07898265
Ex_LGlutamate	-0,5	3,250474177
Ex_LGlutamine	-0,5	1,375237088
Ex_LHistidine	-0,080814852	-0,080814803
Ex_LInositol	0	0
Ex_LIsoleucine	-0,247709368	0,035799246
Ex_LLeucine	-0,384155726	-0,38415549
Ex_LLysine	-0,292548011	0,472253891
Ex_LMethionine	-0,131201728	-0,131201648
Ex_LPhenylalanine	-0,157944337	0,83766491
Ex_LThreonine	-0,5	-0,216491537

Ex_LTryptophan	-0,048462587	-0,048462558
Ex_LTyrosine	-0,117580784	0,878028438
Ex_LValine	-0,361429291	3,389045107
Ex_Maltose	-0,5	0
Ex_Niacin	-0,001999751	-0,00199975
Ex_Ornithine	0	0
Ex_PPi	0	0
Ex_XAN	0	0
Ex_5Deoxyadenosine	0	0
Ex_Acetoacetate	0	6,69557605
Ex_Calomide	0	0
Ex_Cbl	0	0
Ex_Citrate	0	0
Ex_CysGly	0	0
Ex_Glycine	-0,5	-0,499999693
Ex_Glycolaldehyde	0	0
Ex_LProline	-0,188480242	-0,188480126
Ex_Maltohexaose	0	0
Ex_Methanol	0	0
Ex_NAcetylDglucosamine	0	0
Ex_PM	0	0
Ex_Putrescine	0	0
Ex_Pyridoxal	-0,000195675	-0,000195675
Ex_Riboflavin	-0,000391351	-0,000391351
Ex_Salicin	0	0
Ex_Sorbitol	0	0
Ex_Spermidine	0	0
Ex_Sucrose	-0,5	0
Ex_Taurine	0	0
Ex_Thiamin	0	0
Ex_Thymidine	0	0
Ex_TRHL	0	0
Ex_Uracil	-0,281070722	0
Ex_Uridine	-0,281070722	0
Ex_Ursin	0	0
Ex_Mn2+	-0,002639425	-0,002639424
Ex_Formaldehyde	0	0
Ex_Fumarate	0	6,813908456
Ex_Oxidized glutathione	0	0
Ex_Adenine	0	0
Ex_Nicotinamide	0	0
Ex_Co2+	-0,002639425	-0,002639424
Ex_D-Glutamate	0	0
Ex_Nitrate	-0,1	0
Ex_Folate	-0,000782702	-0,000782701
Ex_N-Acetyl-D-mannosamine	0	0
Ex_Siroheme	0	0
Ex_Selenate	0	0
Ex_Menaquinone 7	0	0
Ex_2-Demethylmenaquinone 8	0	0

Ex_Menaquinone 8	0	0
Ex_Ubiquinone-8	0	0
Ex_ABEE	0	0
Ex_Neu5Ac	0	0
Ex_H+	88,10929482	1000
Ex_indol	0	0
Ex_Nicotinamide ribonucleotide	0	0
Ex_PAN	-0,000505422	-0,000505422
Ex_Zn2+	-0,002639425	-0,002639424
Ex_1,2-Diacyl-sn-glycerol dioctadecanoyl	0	0
Ex_L-Serine	-0,5	-0,206948766
Ex_D-Fructose	-0,5	0
Ex_D-Mannose	0	0
Ex_D-Mannitol	0	0
Ex_beta D-Galactose	-0,5	0
Ex_L-Fucose	0	0
t_Arabinan	0	0
t_Starch	0	0,005
t_octanoate	0	0
t_Melibiose	0	0,5
t_Amylose	0	0
Ex_Arabinan	0	0
Ex_Starch	-0,005	0
Ex_Melibiose	-0,5	0
Ex_Amylose	0	0
t_Raffinose_Melitose	0	0
t_Isovaleric_acid	0	0
t_H2O2	0	0
t_Nitric_oxide	0	0
Ex_Raffinose_Melitose	0	0
Ex_Isovaleric_acid	0	0
Ex_H2O2	0	0
Ex_Nitric_oxide	0	0
rxn01207_1	0	0
rxn08972	0	0
rxn08973	0	0
rxn06111	58,37297263	999,9994691
rxn13726	0	0
rxn13727	0	0
rxn13729	0	0
rxn08974	0	0
rxn10122	0	0
rxn10123	0	0
rxn10124	0	0
rxn12665	0	0
rxn06097	0	0,005
t_Sulfite	0	0
Ex_Sulfite	0	0

rxn ID	minFlux	max Flux
rxn00001	0	1000
rxn00014	0	0
rxn00016	0	0
rxn00020	0	0
rxn00022	0	1000
rxn00029	0,000780563	0,000780564
rxn00044	0	0
rxn00060	0,000195141	0,000195141
rxn00062	0	1000
rxn00063	0	1000
rxn00067	0	0
rxn00076	0	1000
rxn00077	0	0,000391157
rxn00085	-1000	0
rxn00097	-1000	1000
rxn00100	0,000504041	0,000504041
rxn00102	-1000	0,565957783
rxn00104	-1000	0
rxn00105	-983,1853255	1000
rxn00106	-1000	0
rxn00109	0	0
rxn00119	0,282724064	1000
rxn00122	0,000195141	0,000195141
rxn00124	0,000195141	0,000195141
rxn00126	0,006486901	0,006486904
rxn00127	0,005901479	0,005901481
rxn00132	0	1000
rxn00134	0	1000
rxn00137	0	0
rxn00139	-999,9978106	0
rxn00142	0	0
rxn00143	0,000390282	0,000390282
rxn00148	-1000	0
rxn00151	-1000	0
rxn00157	-7,830176578	0
rxn00159	-1000	1000
rxn00161	-1000	1000
rxn00162	0	1000
rxn00165	0	0,293616437
rxn00171	0	1,114930992
rxn00173	0	8,292356885
rxn00184	-1000	0
rxn00187	0	1000
rxn00189	0	1000
rxn00190	0,001994287	983,1873198
rxn00193	0,024131833	0,024131842
rxn00200	0	0
rxn00202	0	0
rxn00206	0,006775675	200,8235844

rxn00212	0	999,7172759
rxn00213	-1000	999,6931441
rxn00214	0	0
rxn00216	0	1000
rxn00222	0	0
rxn00225	-8,292356885	0
rxn00231	0	0
rxn00239	0,182977346	633,3138199
rxn00247	0	1000
rxn00250	-0,565984911	999,9999729
rxn00254	0	0
rxn00259	0	0
rxn00260	-203,4272197	0,565984911
rxn00262	0	200,457924
rxn00283	0,021248474	0,021248482
rxn00293	0,048263666	999,7655396
rxn00301	0	633,1196373
rxn00303	0	0
rxn00304	-633,2769634	0
rxn00313	0	1,043461203
rxn00328	0	0
rxn00333	0	0
rxn00337	0,024131833	1,067593035
rxn00338	0	0,001994288
rxn00340	0	1000
rxn00342	0	1000
rxn00347	0	4,217993467
rxn00350	-0,000195141	-0,000195141
rxn00359	0	0
rxn00360	0	0
rxn00363	0	1000
rxn00364	-999,7172759	633,1086967
rxn00365	0	1000
rxn00367	0	633,1086967
rxn00368	0	1000
rxn00369	0	633,1196373
rxn00371	0	1000
rxn00374	0	0
rxn00379	0	0
rxn00383	0,003290267	0,003290268
rxn00388	0	0
rxn00391	0	999,9998049
rxn00392	0,000195141	1000
rxn00405	0,035015091	0,035015104
rxn00410	-999,8640052	999,8532707
rxn00411	-1000	0
rxn00412	0	1000
rxn00414	0	0
rxn00416	0	983,1853255
rxn00423	0	0

rxn00426	0	0
rxn00433	0	0
rxn00436	0	629,4759251
rxn00437	0	0
rxn00440	0,000195141	629,4761203
rxn00459	-0,197170968	7,312279882
rxn00460	-1000	0
rxn00461	0,024131833	0,024131842
rxn00463	0	999,7172759
rxn00474	0	0
rxn00490	0	2,086922405
rxn00493	-2,086922405	0
rxn00499	-7,830176578	0
rxn00514	0	0
rxn00517	-1000	0
rxn00527	-2,087117546	-0,000195141
rxn00533	2,71272E-05	2,71272E-05
rxn00541	-1,81878E-07	0
rxn00543	-1,114930992	0
rxn00545	0	3,160282965
rxn00547	0	1
rxn00551	0	3,160282965
rxn00552	-0,048263683	-0,048263666
rxn00554	0	3,160282965
rxn00556	0	3,160282965
rxn00557	0	3,160282965
rxn00558	-1000	1000
rxn00575	0	0,5
rxn00600	0,001561127	0,001561127
rxn00601	0	0
rxn00602	0	0
rxn00605	0	1000
rxn00608	0	0
rxn00611	0	0
rxn00615	0	0
rxn00621	0	0
rxn00622	0	0
rxn00641	0	0
rxn00645	0,003290267	0,003290268
rxn00647	0	0
rxn00649	0	0
rxn00650	-0,000195141	-0,000195141
rxn00654	0	0
rxn00670	0	0
rxn00684	0	0
rxn00685	0	622,6298024
rxn00686	0	0
rxn00687	0	622,6298024
rxn00689	0	0
rxn00690	0	1,81878E-07

rxn00692	-0,022881365	-0,022881175
rxn00693	0,000390282	0,000390282
rxn00695	-1000	1000
rxn00704	-1000	0
rxn00707	0	1000
rxn00708	0	1000
rxn00709	0	1000
rxn00710	0	0
rxn00711	-999,9978106	0
rxn00712	0	1000
rxn00713	0	633,2489334
rxn00715	0	1000
rxn00741	0	0
rxn00743	0	0
rxn00747	-3,339024736	0,001994288
rxn00758	0	0
rxn00763	0	0
rxn00765	0	0
rxn00770	0,002189428	1000
rxn00772	0,000390282	0,500390282
rxn00776	0	0
rxn00777	-1,111053632	0,357288411
rxn00778	-0,621222755	1000
rxn00781	-0,197170968	7,312279882
rxn00784	0	1,114930992
rxn00785	-0,178546635	1,599085589
rxn00786	0	3,160282965
rxn00789	0	0
rxn00790	-0,000195141	-0,000195141
rxn00791	0	0
rxn00792	0	0
rxn00800	-0,205387989	0,173361254
rxn00802	0	0
rxn00816	0	0
rxn00817	0	0
rxn00818	0	0
rxn00819	0	0
rxn00829	0,000529418	0,000529418
rxn00830	4,81289E-05	4,81289E-05
rxn00831	0	999,9978106
rxn00834	0,205387914	0,205387989
rxn00836	-999,9978106	0
rxn00838	-0,205387989	0,173361254
rxn00851	0	1000
rxn00853	0,035015091	0,035015104
rxn00855	0	0
rxn00858	0	0
rxn00867	0	0
rxn00869	0	0
rxn00871	0	0

rxn00889	0	0
rxn00890	0	0
rxn00892	0	0
rxn00902	0	0
rxn00907	0,0003901	0,000390282
rxn00913	0	1000
rxn00915	-999,9775894	0
rxn00916	-999,7946121	0,205387989
rxn00917	0	1000
rxn00925	0	0
rxn00947	0	1000
rxn00955	0,000390282	0,000390282
rxn00963	0,000195141	2,087117546
rxn00966	-2,086922405	0
rxn00973	-1000	1000
rxn00974	-1000	1000
rxn00977	0	0
rxn00980	0	0
rxn00983	0	0
rxn00985	0	0
rxn00990	0,000529418	0,526514833
rxn00991	-0,000529418	-0,000529418
rxn01000	0	2,086922405
rxn01008	0	0
rxn01016	0	0
rxn01018	0	0
rxn01021	0	0
rxn01022	0,005901479	0,005901481
rxn01029	0,035015091	0,035015104
rxn01049	0	0
rxn01069	0	0
rxn01073	0	0
rxn01100	-1000	0
rxn01106	-7,312279882	0,197170968
rxn01116	-1,111053632	0,357288411
rxn01119	0	0
rxn01133	0	0
rxn01134	-1000	0,505
rxn01138	-0,385041006	999,9915188
rxn01139	0	0
rxn01146	0	0
rxn01153	0	0
rxn01169	0	1000
rxn01171	0	1000
rxn01176	0	0
rxn01182	0	0
rxn01200	0	1000
rxn01210	0	0
rxn01211	0,000194959	0,000390282
rxn01213	4,81289E-05	4,81289E-05

rxn01228	0	0
rxn01244	0	0
rxn01245	0	0
rxn01255	0,000195141	2,087117546
rxn01256	0	2,086922405
rxn01265	-999,9998049	0
rxn01268	0	2,086922405
rxn01270	-2,086922405	0
rxn01274	0	0
rxn01294	0	0
rxn01300	0	0
rxn01303	0	0
rxn01304	0	0
rxn01305	0	0
rxn01322	0	0
rxn01329	0	0
rxn01332	0,000195141	2,087117546
rxn01334	0	0,780948325
rxn01343	0	0,780948325
rxn01346	0	0,780948325
rxn01347	0	0,780948325
rxn01348	0	0,780948325
rxn01351	0	1000
rxn01352	-1000	-0,022410568
rxn01354	-1000	0
rxn01358	0	999,9978106
rxn01361	0	0
rxn01362	0	0
rxn01366	-0,258592325	1000
rxn01367	0	0
rxn01368	0	629,4759251
rxn01370	0	1000
rxn01377	0	0
rxn01379	0	0
rxn01380	0	0
rxn01388	-1000	1000
rxn01390	0	0
rxn01396	0	0
rxn01406	0,005901479	0,005901481
rxn01423	0	0
rxn01426	0	0
rxn01427	0	0
rxn01439	0	0
rxn01445	0	999,9775894
rxn01446	-0,022410576	-0,022410568
rxn01462	0	0
rxn01465	0	0
rxn01466	4,81289E-05	4,81289E-05
rxn01484	0	0
rxn01485	-0,048263683	-0,048263666

rxn01486	0	0
rxn01492	0	0
rxn01500	-0,000529418	-0,000529418
rxn01509	-999,9775894	636,7648367
rxn01510	0	1000
rxn01513	0,021710512	0,021710519
rxn01517	0	0
rxn01518	0,021710512	1000
rxn01519	0	0
rxn01521	0	999,9782895
rxn01539	-629,4761203	-0,000195141
rxn01544	-999,9978106	0
rxn01548	0,022410568	1000
rxn01549	0	0
rxn01571	0	0
rxn01583	0	0
rxn01590	0	0
rxn01601	0	0
rxn01602	0	0
rxn01603	0	0
rxn01613	0	0
rxn01615	0	0
rxn01620	0	0
rxn01627	0,001561127	0,001561127
rxn01629	0	0
rxn01635	0	0
rxn01637	0	0
rxn01641	0	0
rxn01643	-1,067593035	-0,024131833
rxn01644	0,024131833	1,067593035
rxn01646	0	0
rxn01647	0	999,9978106
rxn01648	0	0
rxn01649	0	999,9978106
rxn01650	0	0
rxn01667	0	0
rxn01669	0	983,1853255
rxn01670	0	0
rxn01675	0	0
rxn01679	0	0
rxn01682	0	0
rxn01684	0	0
rxn01704	0	0
rxn01706	0	0
rxn01739	0,000195141	2,087117546
rxn01740	-2,087117546	-0,000195141
rxn01741	0	0
rxn01750	0	0
rxn01757	0	0
rxn01758	-0,003290268	-0,003290267

rxn01761	0	0
rxn01790	0	0
rxn01799	-0,021710519	0,258592325
rxn01800	0	0,280302845
rxn01807	0	0
rxn01843	0	0
rxn01859	0	0,378749243
rxn01860	0	0
rxn01870	0	0
rxn01885	0	0
rxn01896	0	0
rxn01901	0	0
rxn01906	0	0
rxn01933	0	0
rxn01946	0,000195141	2,087117546
rxn01951	0	0
rxn01961	0	999,9978106
rxn01962	0	0
rxn01964	0	0
rxn01966	-1000	1000
rxn01967	-1000	1000
rxn01972	0,024131833	8,316488718
rxn01973	0	0
rxn01974	0,024131833	1,067593035
rxn01977	-1000	1000
rxn01982	0	0
rxn01985	0	0
rxn01986	-0,044121096	0,614930992
rxn01987	-0,5	0
rxn01997	0	0
rxn02003	0	0
rxn02004	0	1000
rxn02007	0	0
rxn02008	0,024131833	0,024131842
rxn02009	0	0
rxn02010	0	0
rxn02011	0,024131833	0,024131842
rxn02093	0	0
rxn02128	0	0
rxn02138	0	0
rxn02139	0	0
rxn02154	0	999,9980057
rxn02155	0,001994287	1000
rxn02160	0	0
rxn02161	0	0
rxn02169	0,000529418	0,526514833
rxn02173	0	0
rxn02175	0,000504041	1000
rxn02176	0	999,999496
rxn02195	0	0

rxn02209	0	0
rxn02212	0,000195141	2,087117546
rxn02213	0,000195141	2,087117546
rxn02219	0	0
rxn02222	0	0
rxn02228	0	0
rxn02236	0	0
rxn02246	0	0
rxn02264	0,000195141	0,000195141
rxn02275	0	0
rxn02283	0	0
rxn02284	-0,024131842	0
rxn02285	-0,024131842	0
rxn02286	0,024131833	0,024131842
rxn02297	0	0
rxn02305	0,000195141	0,000195141
rxn02312	0	0
rxn02314	0	1000
rxn02315	0	1000
rxn02316	0	1000
rxn02317	-1000	0
rxn02319	0	0
rxn02320	0	0
rxn02322	0,000529418	0,000529418
rxn02339	0	0
rxn02341	0,000504041	0,000504041
rxn02351	0	0
rxn02380	-1000	1000
rxn02400	0	999,9978106
rxn02402	-0,001994288	0
rxn02409	0	0
rxn02449	0	0
rxn02454	0	0
rxn02476	0,000195141	2,087117546
rxn02483	0	0
rxn02484	0	0
rxn02495	0	0
rxn02503	0	0
rxn02508	0	0
rxn02518	0	0
rxn02521	0	0
rxn02522	0	0
rxn02525	0	0
rxn02571	0	0
rxn02581	0	0
rxn02596	0	0
rxn02733	0	0
rxn02749	0	0
rxn02751	0	0
rxn02762	0	0

rxn02768	-0,526514833	-0,000529418
rxn02789	0	0
rxn02798	0	0
rxn02810	0,000529418	0,526514833
rxn02811	0	0
rxn02821	0	0
rxn02822	0	0
rxn02875	0	0
rxn02895	0,000195141	0,000195141
rxn02897	0	0
rxn02914	0	0
rxn02922	0	0
rxn02928	-1000	999,9758682
rxn02929	-1000	999,9758682
rxn02931	0	0
rxn02936	0	0
rxn02937	0,000195141	0,000195141
rxn02950	0	0
rxn02988	-0,001994288	0
rxn03003	0	0
rxn03004	0	0,000195141
rxn03005	-0,000195141	0
rxn03030	0,024131833	8,316488718
rxn03036	0	0
rxn03039	0	0
rxn03047	0	0
rxn03057	0,005901479	0,005901481
rxn03075	0,000195141	0,000195141
rxn03084	0,000195141	0,000195141
rxn03086	-8,316488718	-0,024131833
rxn03087	0	0
rxn03089	0	0
rxn03102	0	0
rxn03108	0,000195141	0,000195141
rxn03136	0	0
rxn03140	0	0
rxn03141	0	0
rxn03146	0	0
rxn03147	0	0
rxn03164	0,024131833	0,024131842
rxn03167	0	0
rxn03182	0	0
rxn03188	0	0
rxn03264	0	0
rxn03269	0	0
rxn03273	0	0
rxn03282	0	0
rxn03333	0	0
rxn03373	0	0
rxn03395	0	0

rxn03397	0	0
rxn03402	0	0
rxn03405	0	0
rxn03406	0	0
rxn03407	0	0
rxn03408	0,024131833	0,024131842
rxn03409	0	0
rxn03439	0	0
rxn03445	0	0
rxn03446	0	0
rxn03462	0	0
rxn03465	0	0
rxn03467	0	0
rxn03481	0	0
rxn03482	0	0
rxn03483	0	0
rxn03514	0	0
rxn03548	0	622,6298024
rxn03549	0	0
rxn03608	0	0
rxn03634	0	0
rxn03638	0,048263666	0,048263683
rxn03663	0	0
rxn03667	0	0
rxn03838	0	0
rxn03839	0	0
rxn03869	0	0
rxn03870	0	0
rxn03884	0	0
rxn03887	0	0
rxn03891	0	0
rxn03900	0	0
rxn03901	0,024131833	0,024131842
rxn03902	0	0
rxn03903	0	0
rxn03904	0,024131833	0,024131842
rxn03907	0	0
rxn03908	0	0
rxn03909	0	0
rxn03910	0	0
rxn03933	0	0
rxn03958	0	0
rxn04016	0	0
rxn04045	0	0
rxn04048	0	0
rxn04096	0	0
rxn04113	0	0
rxn04142	0	0
rxn04301	0	0
rxn04308	0	0

rxn04417	0	0
rxn04418	0	0
rxn04432	0	0
rxn04437	0	0
rxn04443	0	0
rxn04476	0	0
rxn04674	0	0
rxn04676	0	1000
rxn04678	-1000	0
rxn04702	-2,087117546	-0,000195141
rxn04704	0	0
rxn04726	0	0
rxn04736	0	0
rxn04794	0	1000
rxn04809	0	0
rxn04810	0	0
rxn04811	0	0
rxn04830	0	0
rxn04831	0	0
rxn04832	0	0
rxn04833	0	0
rxn04872	0	0
rxn04873	0	0
rxn04886	0	0
rxn04887	0	0
rxn04894	0	0
rxn04895	0	0
rxn04896	0	0
rxn04903	0	0
rxn04916	0	0
rxn04919	0	0
rxn04954	-0,000585423	-0,000585422
rxn04960	0	0
rxn05030	4,81289E-05	4,81289E-05
rxn05037	0	0
rxn05039	0	0
rxn05050	0	0
rxn05104	0,005901479	0,005901481
rxn05108	0,005901479	0,005901481
rxn05124	0	0
rxn05234	0	0
rxn05236	0	0
rxn05239	0	0
rxn05247	0	0
rxn05248	0	0
rxn05249	0	0
rxn05250	0	0
rxn05251	0	0
rxn05252	0	0
rxn05256	0	0

rxn05269	0	0
rxn05289	0	0
rxn05322	0	0
rxn05323	0	0
rxn05324	0	0
rxn05325	0	0
rxn05326	0	0
rxn05327	0	0
rxn05328	0	0
rxn05336	0	0
rxn05337	0	0
rxn05338	0	0
rxn05339	0	0
rxn05340	0	0
rxn05341	0	0
rxn05342	0	0
rxn05343	0	0
rxn05344	0	0
rxn05345	0	0
rxn05346	0	0
rxn05347	0	0
rxn05348	0	0
rxn05350	0	0
rxn05465	0	0
rxn05733	0	0
rxn05736	0	1000
rxn05740	-1000	1000
rxn05759	-0,5	0
rxn05760	-146,4602699	1000
rxn05762	0,003485408	2,090407813
rxn05778	0	0
rxn05779	0	0
rxn05871	0	0
rxn05872	0	0
rxn05874	0	0
rxn05878	0	0
rxn05901	0	0
rxn05938	-7,830176578	0
rxn05940	-1000	220,1895319
rxn05958	0	0
rxn05962	0	0
rxn05979	0	0
rxn05990	0	0
rxn05994	0	0
rxn06023	0	0
rxn06031	0	0
rxn06043	0	0
rxn06045	0	0
rxn06071	0,01355135	401,6471688
rxn06080	0	0

rxn06096	0	0
rxn06108	-622,6298024	0
rxn06109	-4,2019801	0
rxn06139	0	0
rxn06140	0	0
rxn06190	0	0
rxn06195	0	0
rxn06196	0	0
rxn06201	0	0
rxn06206	0	0
rxn06217	0	0
rxn06218	0	0
rxn06219	0	0
rxn06227	0	0
rxn06280	0	0
rxn06285	0	0
rxn06293	0	0
rxn06298	0	0
rxn06299	0	0
rxn06300	0	0
rxn06316	0	0
rxn06328	0	0
rxn06373	0	0
rxn06394	0	0
rxn06403	0	0
rxn06432	0	0
rxn06434	0	0
rxn06435	0	0
rxn06437	0	0
rxn06438	0	0
rxn06439	0	0
rxn06440	0	0
rxn06441	0	0
rxn06443	0	0
rxn06444	0	0
rxn06445	0	0
rxn06446	0	0
rxn06447	0	0
rxn06448	0	0
rxn06449	0	0
rxn06459	0	0
rxn06485	0	0
rxn06489	0	0
rxn06522	0	0
rxn06525	0	1000
rxn06526	-1000	0
rxn06538	0	0
rxn06565	0	0
rxn06584	0	0
rxn06595	0	0

rxn06621	0	0
rxn06624	0	0
rxn06648	0	0
rxn06664	0	0
rxn06671	0	0
rxn06701	0	0
rxn06726	0	0
rxn06737	0	0
rxn06751	0	0
rxn06752	0	0
rxn06760	0	0
rxn06768	0	0
rxn06798	0	0
rxn06799	0	0
rxn06823	0	0
rxn06831	0	0
rxn06850	0	0
rxn06864	0	0
rxn06865	0	0
rxn06882	0	0
rxn06887	0	0
rxn06889	0	622,6298024
rxn06936	0	0
rxn06937	0	0
rxn06947	0	0
rxn06958	-401,6471688	-0,01355135
rxn06979	0	0
rxn07056	0	0
rxn07099	0	0
rxn07181	0	0
rxn07193	0	0
rxn07199	0	0
rxn07267	0	0
rxn07292	0	0
rxn07430	0	0
rxn07431	0	0
rxn07432	0	0
rxn07433	0	0
rxn07434	0	0
rxn07435	0	0
rxn07437	0	0
rxn07438	0	0
rxn07441	0	8,292356885
rxn07450	0,003290267	0,003290268
rxn07456	0	0
rxn07466	-0,022425453	999,9775746
rxn07573	0	0
rxn07577	0	0
rxn07579	0	0
rxn07586	0	0

rxn07587	0	0
rxn07645	0	0
rxn07804	0	0
rxn07807	0	0
rxn07832	0	0
rxn07846	0	0
rxn07849	0	0
rxn07987	0	0
rxn07989	0	0
rxn07991	0	0
rxn07992	0	0
rxn07993	0	0
rxn07994	0	0
rxn08025	0	0
rxn08044	0	0
rxn08067	-1000	1000
rxn08083	0	0
rxn08084	0	0
rxn08085	0	0
rxn08086	0	0
rxn08087	0	0
rxn08088	0	0
rxn08089	0	0
rxn08126	0	0
rxn08127	0	0
rxn08128	0	0
rxn08129	0	0
rxn08131	0,000195141	0,000195141
rxn08206	0	0
rxn08207	0	0
rxn08208	0	0
rxn08209	0	0
rxn08294	0	0
rxn08295	0	0
rxn08296	0	0
rxn08297	0	0
rxn08298	0	0
rxn08299	0	0
rxn08300	0	0
rxn08306	0	0
rxn08307	0	0
rxn08308	0	0
rxn08309	0	0
rxn08310	0	0
rxn08311	0	0
rxn08312	0	0
rxn08352	0	0
rxn08386	0	0
rxn08390	0	0
rxn08392	0	0

rxn08394	0	0
rxn08396	0	0
rxn08398	0	0
rxn08448	0	0
rxn08449	0	0
rxn08451	0	0
rxn08453	0	0
rxn08454	0	1000
rxn08455	0	0
rxn08456	0	0
rxn08457	0	0
rxn08519	0,04412108	0,044121096
rxn08546	0	0
rxn08547	0	0
rxn08548	0	0
rxn08549	0	0
rxn08550	0	0
rxn08551	0	0
rxn08552	0	0
rxn08582	0	0
rxn08605	0	0
rxn08607	0	0
rxn08615	-1000	1000
rxn08647	0	0
rxn08668	0	0
rxn08669	0	0
rxn08796	0	0
rxn08797	0	1000
rxn08798	0	0
rxn08799	0	1000
rxn08800	0	0
rxn08801	0	1000
rxn08802	0	0
rxn08803	0	0
rxn08804	0	0
rxn08805	0	0
rxn08806	0	0
rxn08807	0	0
rxn08808	0	0
rxn08809	0	0
rxn08810	0	0
rxn08811	0	0
rxn08812	0	0
rxn08813	0	0
rxn08814	0	0
rxn08815	0	0
rxn08816	0	0
rxn08817	0	0
rxn08818	0	0
rxn08819	0	0

rxn08820	0	0
rxn08821	0	0
rxn08822	0	0
rxn08823	0	0
rxn08838	0	0
rxn08839	0	0
rxn08840	0	0
rxn08841	0	0
rxn08842	0	0
rxn08843	0	0
rxn08844	0	0
rxn08845	0	0
rxn08846	0	0
rxn08847	0	0
rxn08848	0	0
rxn08849	0	0
rxn08850	0	0
rxn08851	0	0
rxn08857	0	0
rxn08889	0,000588923	0,000588923
rxn08890	0,004767387	0,004767389
rxn08891	0,000588923	0,000588923
rxn08892	0,010005562	0,010005565
rxn08893	0,004708757	0,004708759
rxn08894	0,00117697	0,001176971
rxn08897	-0,005296807	-0,005296805
rxn08926	0,000529418	0,000529418
rxn08928	0,00117697	0,001176971
rxn08929	0,00117697	0,001176971
rxn08958	0,000588923	0,000588923
rxn09010	0	0
rxn09016	0	633,1308426
rxn09069	0	0
rxn09101	0	0
rxn09102	0	0
rxn09103	0	0
rxn09104	0	0
rxn09105	0	0
rxn09106	0	0
rxn09107	0	0
rxn09108	0	0
rxn09109	0	0
rxn09110	0	0
rxn09111	0	0
rxn09112	0	0
rxn09113	0	0
rxn09114	0	0
rxn09176	-1000	1000
rxn09235	0,021710512	0,021710519
rxn09237	0,022410568	0,022410576

rxn09240	0	0
rxn09264	0	0
rxn09265	0	0
rxn09340	0	0
rxn09341	0	999,7172759
rxn09348	0	0
rxn09355	0	0
rxn09398	-1000	999,6931441
rxn09399	0	0
rxn09445	0	0
rxn09446	0	0
rxn09447	0	0
rxn09486	0	0
rxn09502	0	1000
rxn09519	0	0
rxn09557	0,000195141	0,000195141
rxn09615	0,000529418	0,000529418
rxn09632	0	629,4759251
rxn09888	0	0
rxn09949	0	0
rxn09952	0	0
rxn09978	0	0
rxn09979	0	0
rxn09988	0	0
rxn09992	0	0
rxn09995	0	0
rxn10003	0,000504041	0,000504041
rxn10019	0	0
rxn10020	0	0
rxn10021	0	0
rxn10052	-1000	1000
rxn10054	0	999,7172759
rxn10056	0	0,000391157
rxn10058	0	0,000391157
rxn10060	0	0,000391157
rxn10075	0,005901479	0,005901481
rxn10107	0	0
rxn10191	0	0
rxn10192	0	0
rxn10193	0	0
rxn10202	0	1000
rxn10203	0	1000
rxn10204	0	1000
rxn10205	0	0
rxn10206	0	0
rxn10207	0	0
rxn10208	0	0
rxn10209	0	0
rxn10210	0	0
rxn10211	0	0

rxn10212	0	0
rxn10213	0	0
rxn10214	0	0
rxn10215	0	0
rxn10216	0	0
rxn10217	0	0
rxn10218	0	0
rxn10219	0	0
rxn10220	0	0
rxn10221	0	0
rxn10222	0	0
rxn10223	0	0
rxn10224	0	0
rxn10225	0	0
rxn10253	0	0
rxn10254	0	0
rxn10255	0	0
rxn10256	0	0
rxn10257	0	0
rxn10258	0	0
rxn10259	0	0
rxn10260	0	0
rxn10261	0	0
rxn10262	0	0
rxn10263	0	0
rxn10264	0	0
rxn10265	0	0
rxn10266	0	0
rxn10267	0	0
rxn10268	0	0
rxn10269	0	0
rxn10270	0	0
rxn10289	0	0
rxn10290	0	0
rxn10291	0	0
rxn10292	0	0
rxn10293	0	0
rxn10294	0	0
rxn10295	0	0
rxn10296	0	0
rxn10297	0	0
rxn10298	0	0
rxn10299	0	0
rxn10300	0	0
rxn10301	0	0
rxn10302	0	0
rxn10303	0	0
rxn10304	0	0
rxn10305	0	0
rxn10306	0	0

rxn10363	0	0
rxn10404	0	0
rxn10405	0	0
rxn10406	0	0
rxn10407	0	0
rxn10408	0	0
rxn10409	0	0
rxn10410	0	0
rxn10563	-2,087117546	-0,000195141
rxn10785	4,81289E-05	4,81289E-05
rxn10790	0,000195141	0,000195141
rxn10798	-0,000195141	-0,000195141
rxn10816	0,000195141	0,000195141
rxn10951	0,021710512	0,021710519
rxn11007	0,021710512	0,021710519
rxn11510	0	0
rxn11547	0	0
rxn11548	0	0
rxn11550	0	0
rxn11567	0	0
rxn11571	0	0
rxn11587	0	0
rxn11599	0	0
rxn11609	0	0
rxn11702	0	0
rxn11731	0	0
rxn11732	-0,526514833	-0,000529418
rxn11749	0	0
rxn11756	0	0
rxn11757	-999,9978106	0
rxn11759	0	999,9978106
rxn11760	-999,9978106	0
rxn11761	0	0
rxn11765	0	0
rxn11766	0	0
rxn11768	0	0
rxn11772	0	0
rxn11773	0	0
rxn11808	0	0
rxn11946	0	0
rxn11951	0	0
rxn12013	0	0
rxn12033	0	0
rxn12154	0	0
rxn12218	-0,000195141	-0,000195141
rxn12221	0,000195141	0,000195141
rxn12239	0,000195141	0,000195141
rxn12510	0,000504041	0,000504041
rxn12649	-999,9992194	0
rxn12676	-0,000195141	-0,000195141

rxn12778	0	0
rxn12822	-1000	0
rxn13147	0,000195141	0,000195141
rxn13207	0,000195141	0,000195141
rxn13208	0,000195141	0,000195141
rxn13420	0,000529418	0,526514833
rxn13421	0,000529418	0,526514833
rxn13687	0	0
rxn13768	-4,174235092	-0,000390282
rxn13936	0,011771455	0,011771459
rxn13963	-4,180815626	-0,006970816
rxn13974	-7,830176578	0
rxn14029	0	0
rxn14048	-0,001646884	-0,001646884
rxn14054	-622,6298024	0
rxn14070	0	0
rxn14089	-622,6298024	0
rxn14093	0	0
rxn14120	-1000	-0,000780563
rxn14132	0	0
rxn14178	-220,1895319	1000
rxn14322	0	0
rxn14346	0	0
rxn90002	-7,312279882	1000
rxn90003	0	0
rxn90004	0	0
rxn90005	-0,022101676	-0,021710512
rxn08173	0	311,3149012
Biomass_Bacteria	0,875071	0,875071318
t_Cl	0,00394832	0,003948322
t_Sulfate	0	0
t_Cu2+	0,002632214	0,002632215
t_Mg	0,006579659	0,006579661
t_Ca2+	0,00394832	0,003948322
t_NH3	0	0
t_H2O	-16,62129424	3,4562184
t_Biomass	-0,875071318	-0,875071
t_D-Lactate	0	0
t_Ethanol	-1,114930992	0
t_Formate	-8,230599005	0
t_H2	0	0,5
t_L-Lactate	-7,830176578	0
t_Phosphate	1,162844224	1,54159389
t_Propionate	0	0
t_O2	0	0
t_D-Glucose	0	0,5
t_CO2	-8,230599005	0
t_Acetate	-8,316293577	0
t_Succinate	-4,2019801	0
t_H2S	0	0

Ex_Cl	-0,003948322	-0,00394832
Ex_Sulfate	0	0
Ex_Cu2+	-0,002632215	-0,002632214
Ex_Mg	-0,006579661	-0,006579659
Ex_Ca2+	-0,003948322	-0,00394832
Ex_NH3	0	0
Ex_H2O	-3,4562184	16,62129424
Ex_Biomass	0,875071	0,875071318
Ex_D-Lactate	0	0
Ex_Ethanol	0	1,114930992
Ex_Formate	0	8,230599005
Ex_H2	-0,5	0
Ex_L-Lactate	0	7,830176578
Ex_Phosphate	-1,54159389	-1,162844224
Ex_Propionate	0	0
Ex_O2	0	0
Ex_D-Glucose	-0,5	0
Ex_CO2	0	8,230599005
Ex_Acetate	0	8,316293577
Ex_Succinate	0	4,2019801
Ex_H2S	0	0
t_Fe2	0,006116746	0,006116749
t_fe3	0,005921605	0,005921608
t_Acetaldehyde	-1,114930992	0
t_Adenosine	0	0,378749243
t_AMP	0	0,378749243
t_Amylotriose	0	0
t_BIOT	0	0
t_Choline	0	0
t_Cytidine	0	0
t_Cytosine	0	0
t_DAlanine	0	0
t_Deoxyadenosine	0	0,378749243
t_Deoxycytidine	0	0,280302845
t_Deoxyguanosine	0	0
t_Deoxyinosine	0	0
t_Deoxyuridine	0	0
t_DRibose	0	0,5
t_GLUM	0	0
t_Glycerol	0	0
t_GSH	0	0
t_Guanine	0	0
t_H2S2O3	0	0
t_Heme	0,000195141	0,000195141
t_Homocysteine	0	0
t_HYXN	0	0,378749243
t_Inosine	0	0,378749243
t_LACT	0	0
t_LAlanine	0,482128243	0,482128418
t_LArabinose	0	0

t_LArginine	0,286510496	0,286510601
t_LAsparagine	-0,543461203	0,5
t_LAspartate	-1,586922405	0,5
t_LCysteine	0,082057158	0,082057188
t_LGlutamate	-0,025985415	0,5
t_LGlutamine	-0,025985415	0,5
t_LHistidine	0,080594039	0,080594068
t_LInositol	0	0
t_LIsoleucine	0,247032543	0,247032633
t_LLeucine	0,383106084	0,383106223
t_LLysine	-0,751712531	0,291748778
t_LMethionine	0,130843241	0,130843289
t_LPhenylalanine	-1,929409625	0,157512837
t_LThreonine	0,21570482	0,21570508
t_LTryptophan	0,048330171	0,048330189
t_LTyrosine	-1,969662891	0,117259557
t_LValine	0,360441745	0,360441876
t_Maltose	0	0,5
t_Niacin	0	0,001994288
t_PPi	0	0
t_Pyridoxol	0	0
t_XAN	0	0
t_5Deoxyadenosine	0	0
t_Acetoacetate	-0,525985415	0
t_Citrate	0	0
t_CysGly	0	0
t_Glycine	0,499999818	0,5
t_Glycolaldehyde	0	0
t_LProline	0,187965251	0,187965319
t_Maltohexaose	0	0
t_Methanol	0	0
t_NAcetylDglucosamine	0	0
t_PM	0	0
t_Putrescine	0	0
t_Pyridoxal	0,000195141	0,000195141
t_Riboflavin	0,000390282	0,000390282
t_Sorbitol	0	0
t_Spermidine	0	0
t_Sucrose	0	0,5
t_Thiamin	0	0
t_Thymidine	0	0
t_Thyminose	0	0,5
t_TRHL	0	0
t_Uracil	0	0,280302845
t_Uridine	0	0,280302845
t_Mn2+	0,002632214	0,002632215
t_Formaldehyde	0	0
t_Fumarate	-4,2019801	0
t_Adenine	0	0
t_Nicotinamide	0	0

t_4-Hydroxybenzoate	0	0
t_Co2+	0,002632214	0,002632215
t_D-Glutamate	0	0
t_Chorismate	0	0
t_Folate	0,000780563	0,000780564
t_N-Acetyl-D-mannosamine	0	0
t_Siroheme	0	0
t_Selenate	0	0
t_Menaquinone 7	0	0
t_2-Demethylmenaquinone 8	0	0
t_Menaquinone 8	0	0
t_Ubiquinone-8	0	0
t_2-Oxobutyrate	0	0
t_3MOP	0	0
t_Neu5Ac	0	0
t_Glycerol-3-phosphate	0	0
t_H+	-1000	0,5
t_indol	0	0
t_Nicotinamide ribonucleotide	0	0
t_PAN	0,000504041	0,000504041
t_Pyridoxal phosphate	0	0
t_Zn2+	0,002632214	0,002632215
t_1,2-Diacyl-sn-glycerol dioctadecanoyl	0	0
t_meso-2,6-Diaminopimelate	0	0
t_L-Serine	0,206383563	0,5
t_D-Fructose	0	0,5
t_D-Mannose	0	0
t_L-Rhamnose	0	0
t_beta D-Galactose	0	0
t_L-Fucose	0	0
Ex_Fe2	-0,006116749	-0,006116746
Ex_fe3	-0,005921608	-0,005921605
Ex_Acetaldehyde	0	1,114930992
Ex_Adenosine	-0,378749243	0
Ex_AMP	-0,378749243	0
Ex_Amylotriose	0	0
Ex_BIOT	0	0
Ex_Choline	0	0
Ex_Cytidine	0	0
Ex_Cytosine	0	0
Ex_DAlanine	0	0
Ex_Deoxyadenosine	-0,378749243	0
Ex_Deoxycytidine	-0,280302845	0
Ex_Deoxyguanosine	0	0
Ex_Deoxyinosine	0	0
Ex_Deoxyuridine	0	0
Ex_DRibose	-0,5	0
Ex_GLUM	0	0
Ex_Glycerol	0	0
Ex_GSH	0	0

Ex_Guanine	0	0
Ex_Heme	-0,000195141	-0,000195141
Ex_Homocysteine	0	0
Ex_HYXN	-0,378749243	0
Ex_Inosine	-0,378749243	0
Ex_LACT	0	0
Ex_LAlanine	-0,482128418	-0,482128243
Ex_LArabinose	0	0
Ex_LArginine	-0,286510601	-0,286510496
Ex_LAsparagine	-0,5	0,543461203
Ex_LAspartate	-0,5	1,586922405
Ex_LCysteine	-0,082057188	-0,082057158
Ex_LGlutamate	-0,5	0,025985415
Ex_LGlutamine	-0,5	0,025985415
Ex_LHistidine	-0,080594068	-0,080594039
Ex_LInositol	0	0
Ex_LIsoleucine	-0,247032633	-0,247032543
Ex_LLeucine	-0,383106223	-0,383106084
Ex_LLysine	-0,291748778	0,751712531
Ex_LMethionine	-0,130843289	-0,130843241
Ex_LPhenylalanine	-0,157512837	1,929409625
Ex_LThreonine	-0,21570508	-0,21570482
Ex_LTryptophan	-0,048330189	-0,048330171
Ex_LTyrosine	-0,117259557	1,969662891
Ex_LValine	-0,360441876	-0,360441745
Ex_Maltose	-0,5	0
Ex_Niacin	-0,001994288	0
Ex_PPi	0	0
Ex_XAN	0	0
Ex_5Deoxyadenosine	0	0
Ex_Acetoacetate	0	0,525985415
Ex_Citrate	0	0
Ex_CysGly	0	0
Ex_Glycine	-0,5	-0,499999818
Ex_Glycolaldehyde	0	0
Ex_LProline	-0,187965319	-0,187965251
Ex_Maltohexaose	0	0
Ex_Methanol	0	0
Ex_NAcetylDglucosamine	0	0
Ex_PM	0	0
Ex_Putrescine	0	0
Ex_Pyridoxal	-0,000195141	-0,000195141
Ex_Riboflavin	-0,000390282	-0,000390282
Ex_Sorbitol	0	0
Ex_Spermidine	0	0
Ex_Sucrose	-0,5	0
Ex_Thiamin	0	0
Ex_Thymidine	0	0
Ex_Thyminose	-0,5	0
Ex_TRHL	0	0

Ex_Uracil	-0,280302845	0
Ex_Uridine	-0,280302845	0
Ex_Mn2+	-0,002632215	-0,002632214
Ex_Formaldehyde	0	0
Ex_Fumarate	0	4,2019801
Ex_Adenine	0	0
Ex_Nicotinamide	0	0
Ex_4-Hydroxybenzoate	0	0
Ex_Co2+	-0,002632215	-0,002632214
Ex_D-Glutamate	0	0
Ex_Folate	-0,000780564	-0,000780563
Ex_N-Acetyl-D-mannosamine	0	0
Ex_Siroheme	0	0
Ex_Selenate	0	0
Ex_Menaquinone 7	0	0
Ex_2-Demethylmenaquinone 8	0	0
Ex_Menaquinone 8	0	0
Ex_Ubiquinone-8	0	0
Ex_Neu5Ac	0	0
Ex_H+	-0,5	1000
Ex_indol	0	0
Ex_Nicotinamide ribonucleotide	0	0
Ex_PAN	-0,000504041	-0,000504041
Ex_Zn2+	-0,002632215	-0,002632214
Ex_1,2-Diacyl-sn-glycerol dioctadecanoyl	0	0
Ex_L-Serine	-0,5	-0,206383563
Ex_D-Fructose	-0,5	0
Ex_D-Mannose	0	0
Ex_L-Rhamnose	0	0
Ex_beta D-Galactose	0	0
Ex_L-Fucose	0	0
t_Arabinan	0	0
t_Starch	0	0,005
t_octanoate	0	0
t_Melibiose	0	0
t_Amylose	0	0
Ex_Arabinan	0	0
Ex_Starch	-0,005	0
Ex_Melibiose	0	0
Ex_Amylose	0	0
t_Raffinose_Melitose	0	0
t_Isovaleric_acid	0	0
t_H2O2	0	0
Ex_Raffinose_Melitose	0	0
Ex_Isovaleric_acid	0	0
Ex_H2O2	0	0
rxn01207_1	0	0
rxn08972	0	0
rxn08973	0	0
rxn06111	0	622,6298024

rxn13726	0	0
rxn13727	0	0
rxn13729	0	0
rxn08974	0	0
rxn10122	0	0
rxn10123	0	0
rxn10124	0	0
rxn12665	0	0
rxn06097	0	0,005
t_Sulfite	0	0
Ex_Sulfite	0	0
